# The development of a new parameter for tracking post-transcriptional regulation allows the detailed map of the *Pseudomonas aeruginosa* Crc regulon

**DOI:** 10.1038/s41598-018-34741-9

**Published:** 2018-11-14

**Authors:** Fernando Corona, Jose Antonio Reales-Calderón, Concha Gil, José Luis Martínez

**Affiliations:** 1 Departamento de Biotecnología Microbiana, Centro Nacional de Biotecnología, CSIC, Madrid, Spain; 2 Departamento de Microbiología y Parasitología, Facultad de Farmacia, Universidad Complutense de Madrid and Instituto Ramón y Cajal de Investigaciones Sanitarias (IRYCIS), Madrid, Spain; 3 Present Address: Singapore Immunology Network (SIgN), A*STAR; 8A Biomedical Grove, Level 4, Immunos (Biopolis), Singapore, 138648 Singapore

## Abstract

Bacterial physiology is regulated at different levels, from mRNA synthesis to translational regulation and protein modification. Herein, we propose a parameter, dubbed post-transcriptional variation (PTV), that allows extracting information on post-transcriptional regulation from the combined analysis of transcriptomic and proteomic data. We have applied this parameter for getting a deeper insight in the regulon of the *Pseudomonas aeruginosa* post-transcriptional regulator Crc. *P. aeruginosa* is a free-living microorganism, and part of its ecological success relies on its capability of using a large number of carbon sources. The hierarchical assimilation of these sources when present in combination is regulated by Crc that, together with Hfq (the RNA-binding chaperon in the complex), impedes their translation when catabolite repression is triggered. Most studies on Crc regulation are based either in transcriptomics or in proteomics data, which cannot provide information on post-transcriptional regulation when analysed independently. Using the PTV parameter, we present a comprehensive map of the Crc post-transcriptional regulon. In addition of controlling the use of primary and secondary carbon sources, Crc regulates as well cell respiration, c-di-GMP mediated signalling, and iron utilization. Thus, besides controlling the hyerarchical assimilation of carbon sources, Crc is an important element for keeping bacterial homeostasis and, consequently, metabolic robustness.

## Introduction

*P. aeruginosa* is an important opportunistic pathogen able to live in different ecosystems as well as to infect immunocompromised patients. The capacity of *P. aeruginosa* for growing in different environments relies on a plethora of regulatory mechanisms, which integrate extracellular and intracellular signals to optimize the bacterial physiology in each situation^1^. In particular, bacterial metabolism is subjected to several layers of regulation that include transcriptional regulation and mRNA degradation, translational regulation and, once the protein is synthetized, other events of protein modification and activation. Whereas methods for studying at a global level transcriptional regulation and even mRNA degradation are well established^2–4^, and proteomics allows tracking changes at the protein level, simple and standardized methodologies for linking transcriptomics and proteomics in the aim of defining translational changes are not so frequent.

Free-living, metabolically versatile, environmental bacteria, as *P. aeruginosa*, harbour global regulation systems that allow cells to selectively assimilate preferred nutrients among the mixture of carbon sources that can be present in the habitats that these organisms can colonize. Carbon catabolite repression is one of the aformentioned regulatory systems and regulates the hierarchical acquisition of carbon sources in complex media^5,6^. Catabolite repression control in *P. aeruginosa* is regulated at the post-transcriptional level through the activity of three elements, the proteins Crc and Hfq and the small RNA CrcZ. Hfq is an RNA chaperone with multiple regulatory functions, from riboregulation to mRNA translational repression^7^. The current model of the Crc/Hfq regulatory system supports that Hfq binds the target RNAs, which contain A-rich binding motifs, and Crc facilitates formation of a more stable complex at these targets^8,9^. Consistent with this model, the analysis of the transcriptomes of *hfq* and *crc P. aeruginosa* mutants has shown that they present a large degree of overlapping^9^. However, the finding that some mRNAs behave differently in both mutants, including some transcripts showing inverse regulation in the *crc* and *hfq* mutants^9^, may suggest, as raised by Kambara *et al*. that Crc might still have regulatory activities going beyond “just influencing the degree with which Hfq occupies a particular transcript”^10^. The CrcZ small RNA acts as a molecular switch of carbon catabolite repression control^11^. When the preferred carbon source of *P. aeruginosa* (dicarboxylic acids) is consumed, the small RNA CrcZ is overexpressed. In this situation, CrcZ sequestrates the Crc/Hfq complex, allowing the translation of their target mRNAs^11–13^. Best known mRNA targets of Crc/Hfq encode enzymes, transporters and local regulators that are involved in the uptake and catabolism of secondary carbon sources. Apart from its role in carbon catabolite repression, Crc/Hfq also modulate, biofilm formation^14^, pyocyanin production^15,16^, quorum sensing^17^, rhamnolipid production^18^, type III secretion^19^, and the secretion of proteinaceous virulence factors, such as ToxA^20^. A *crc* mutant is also more susceptible to different injuries, including antibiotics^21^. In addition, while *P. aeruginosa* can inhibit the growth of unicellular predators as *Dictytostelium discoideum*, this bacterivorous amoeba can grow well using a *P. aeruginosa crc* defective mutant as food resource^21^. Hence, the activity of Crc goes beyond regulating the use of carbon sources and have profound implications on the ecological behaviour of this microorganism.

Although the phenotypic effects caused by the inactivation of Crc in the assimilation of secondary carbon sources are clear, several of the molecular mechanisms that underlie the regulatory pathways between Crc and a given phenotype still remains unknown and it is not clear whether these changes are due to a direct regulation by Crc or are indirect effects. First step in defining such regulation will be a precise delimitation of the post-transcriptional effect associated to Crc activity. Different transcriptomic and proteomic assays have been performed to describe the regulon of Crc in *Pseudomonas*^21–24^. However, since Crc is a post-transcriptional regulator, neither the transcriptomic data nor the proteomic data by themselves, when analysed independently, can describe targets of the post-transcriptional action of Crc. Indeed, transcriptomic is insufficient to describe the direct targets of Crc regulation, since the information is at the mRNA level and the effect of Crc is at the level of translation. Further, even proteomic information alone is not enough to track the Crc-associated post-transcriptional repression, since changes in the expression levels of a protein most frequently reflect changes in the levels of its encoding mRNA, which does not necessarily involve post-transcriptional regulation. This is particularly relevant in the case of global regulators, as Crc, which regulate the expression of a large number of proteins, including transcriptional regulators. This regulation of regulators may produce indirect effects in the regulation of a large set of genes. In addition, by controlling different catabolic pathways, Crc/Hfq control the quantity of the pathways’ metabolites, and these metabolites may act as transcriptional effectors further modifying the regulatory networks in an indirect (yet reproducible) way. Some proposed targets of the post-transcriptional action of Crc have been analysed by specific experiments, performing transcriptional fusions with reporter genes^11,23^. However, these experiments are time-consuming and only allow the simultaneous analysis of a small set of genes. At the time of writing the current article, a novel elegant approach, based in the use of ChIP-seq for analysing the binding of nascent RNAs to Hfq may help to fill this gap^10^. Nevertheless, and while the analysis of the differential recovery (and hence enrichment) of mRNAs bound to this regulator is useful for defining its potential targets, this methodology does not allow to get a direct picture of the effect of this potential binding on the actual translational regulation of the messengers, which is only formally provided when more specific assays, as the translation fusions described in the same article, are made.

To perform a global analysis of the post-transcriptional effect of Crc on *P. aeruginosa* physiology, we have compared the transcriptomes and proteomes of a Crc deficient mutant with those of its parental wild-type strain and have defined a novel parameter, dubbed as post-transcriptional variation parameter, to track specifically post-transcriptional regulation at a global scale. Using this parameter, we have been able to describe more precisely the network of Crc-associated post-transcriptional regulation in *P. aeruginosa*. In addition to the specific application to the study of Crc, the post-transcriptional variation parameter defined in our work, is a simple tool for identifying the elements of translational regulons at a global scale.

## Results and Discussion

The transcriptomes and proteomes of the wild type strain PAO1 and its isogenic Δ*crc* counterpart (FCP001) were obtained by Illumina-based RNA-Seq and iTRAQ respectively. The experiments were done in LB medium during the mid-exponential phase of growth. In this complex and rich medium, the CrcZ levels, which is an antagonist of Crc/Hfq, are lower in comparison with other growing condition. Thereby, the catabolite repression mediated by Crc is the highest^25^.

### Effect of Crc on the transcriptome of *P. aeruginosa*

**Figure 1:**
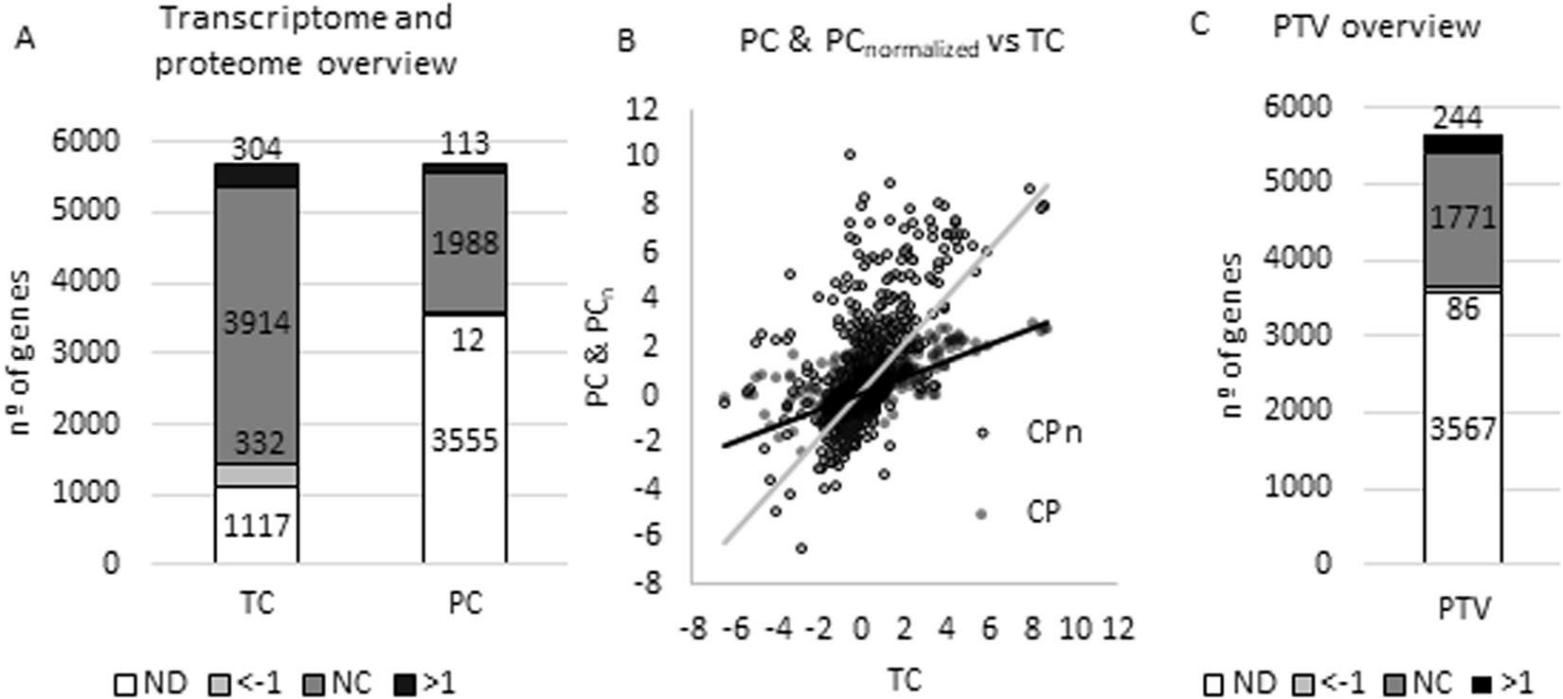
Global analysis of the post-transcriptional changes associated to the inactivation of Crc. (**A**) Detected genes. Number of genes whose transcripts or proteins were detected in the transcriptome and proteome analysis. The transcripts/proteins were classified depending on their changes in the level of expression between the ∆*crc* mutant and the wild-type strain. Black, transcripts/proteins presenting an increasing expression in the ∆*crc* mutant as compared with the wild type strain (log_2_ fold change >1). Pale grey transcripts/proteins presenting a lower expression in the ∆*crc* mutant as compared with the wild type strain (log_2_ fold change <−1). Dark grey transcripts/proteins not showing relevant changes in their expression levels (log_2_ fold change between −1 and 1, NC). ND No detected. (**B**) PC & PC_normalized_ vs TC. All the PC values of each of the genes were plotted against the TC values and the slope is represented. The new PC_normalized values_ present a slope = 1 when represented against TC. (**C**) Genes with assigned PTV. Number of genes that have an assigned PTV. Black, genes presenting an increasing PTV in the ∆*crc* mutant as compared with the wild type strain (log_2_ fold change >1). Pale grey genes presenting a lower PTV in the ∆*crc* mutant as compared with the wild type strain (log_2_ fold change <−1). Dark grey genes not showing relevant changes in their PTVs (log_2_ fold change between −1 and 1, NC). ND No detected.

RNA-seq experiments provide the RPKM parameter (reads *per* kilobase million), which is an absolute value of the expression of each transcript that represents the number of reads that aligns with each gene divided by the base pairs of such gene and normalized by the longitude of the genome. RPKM values were obtained using Rockhopper^26^ and are shown in Supplementary Material, Table S1. A RPKM threshold of 10 was set to define the expressed transcripts of the strains. Genes presenting a level of expression below this threshold in the two analysed strains were discarded in the subsequent analysis. After this approach, 4450 transcripts were detected in both strains (Fig. 1).

**Figure 2:**
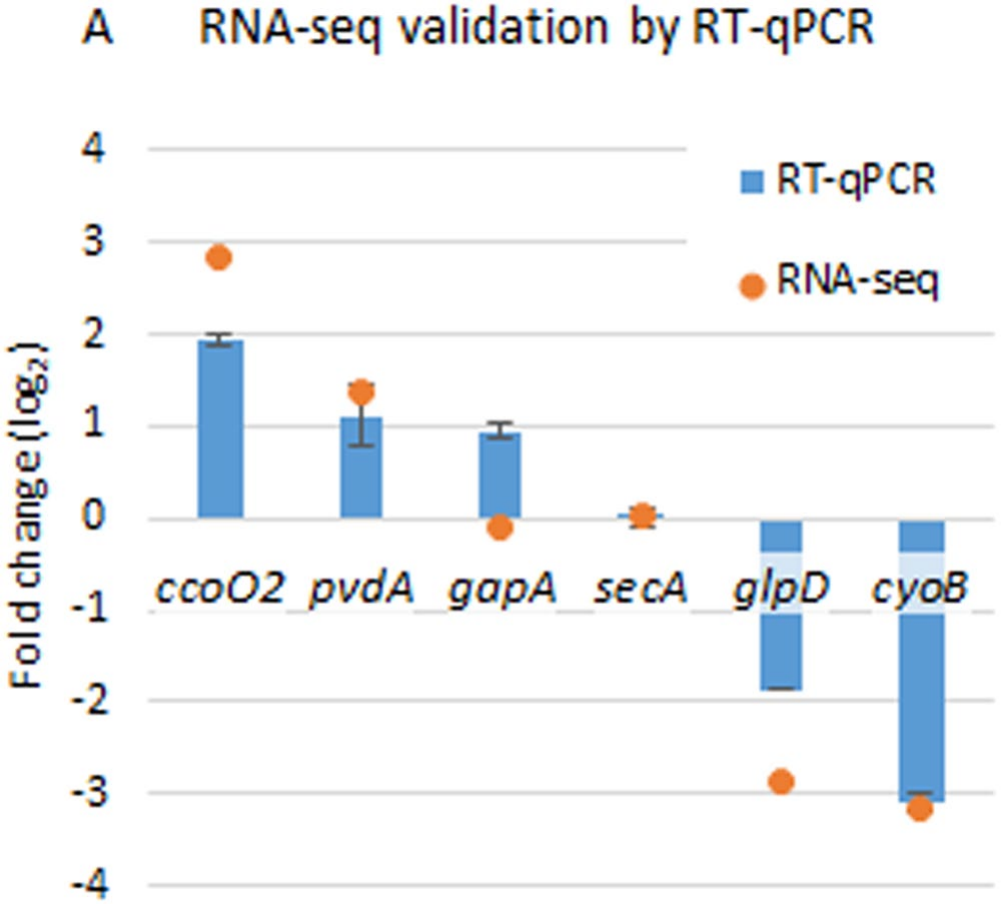
Validation of RNA-seq analysis by RT-qPCR. The results represent the fold change of the selected genes by RT-qPCR of tree biological samples (log_2_ of fold change value calculated by the 2^−ΔΔCt^ method^35^), and the result of RNA-seq (log_2_ of RPKM^Δ*crc*^/RPKM^PAO1^).

To describe the relative expression of a given gene (fold change) in the mutant *∆crc* strain compared to the wild type the transcript change parameter (TC) was determined for each of the transcripts. TC was defined as the log_2_ of the value of the RPKM of a messenger in the Δ*crc* strain divided by the RPKM of the same mRNA in the wild type strain. TC values over 1 or below −1 were defined as thresholds of changes of gene expression. In this way, 304 genes were overexpressed in the Δ*crc* mutant and 114 were repressed comparing to the wild type strain. To confirm the reliability of our data, the expression levels of a set of genes presenting different changes in their expression as determined by RNA-seq, were analysed by real time RT-PCR as described in Methods. As shown in Fig. 2, the changes in the levels of expression when Crc is absent were similar when they were analysed either by RNA-seq or by real time RT-PCR.

### Effect of Crc on the proteome of *P. aeruginosa*

Three independent biological replicates were used to perform the proteomic analysis of the *P. aeruginosa* Δ*crc* mutant compared to its parental strain PAO1 as described in Methods. The results of the analysis of each replicate is shown in Supplementary Material, Table S2 and allow identifying and quantifying 2113 proteins. Only those changes in the level of expression that were detected in at least two of the three biological replicates were considered for further analyses.

Previous analysis on the effect of Crc in *P. aeruginosa* proteome using 2D gels allowed the identification of 66 proteins^21^. Among them, 14 were expressed at higher level and 6 at lower level in the *∆crc* mutant using a two-fold threshold^21^. Among the more than 2000 proteins identified in the current analysis, 113 proteins were more abundant and 12 were found in less quantity in the Δ*crc* mutant compared to the wild type strain using the same threshold (Fig. 1). To further analyse the Crc regulon, a protein change parameter (PC) was defined as the log_2_ of the relative quantity of each of proteins of the Δ*crc* mutant comparing to the wild type strain.

### The post-transcriptional variation parameter, a simple tool for analysing translational regulons

As said before, a combined analysis of proteomic and transcriptomic data is needed to define post-transcriptional regulation. To compare these two parameters, the PC value of each gene was normalized as a function of the TC values. To note here that all transcripts with RPKM values above 10 in one of the strains and all proteins detected in at least two of the three replicates were used in the analysis irrespectively on whether or not their levels were different in the Δ*crc* mutant and in the wild-type strain. Thereby, PC values were plotted against TC values. Then, PC_normalized_ values were obtained dividing each PC value by the slope of the linear regression of the PC vs TC curve (Fig. 1B). Finally, we defined the post-transcriptional variation parameter (PTV) as PC_normalized_ − TC. A threshold of |1| was set to define significant changes of post-transcriptional regulation. PC, TC, and PTV values for each gene are listed in Supplementary Material (Table S3).

The PTV parameter reveals the post-transcriptional action of Crc when the wild type and the Δ*crc* strains are compared. Crc is a post-transcriptional repressor that acts concertedly with Hfq on their RNA targets^8,9,12,13,27^. Consequently, a PTV value >1 for a given gene indicates that such gene lacks post-transcriptional repression in the Δ*crc* mutant, being this gene a candidate for the post-transcriptional action of Crc. A PTV value between −1 and 1 reveals an absence of substantial post-transcriptional repression caused by Crc. PTV values <−1 indicate that these genes are under a translational regulation opposite to the one caused by Crc. Using this novel PTV parameter, we found that 244 out of 2101 analysed genes are potential targets of post-transcriptional regulation by Crc, while 86 genes showed the opposite regulation (Fig. 1C).

It is important to notice that the analysis of translational regulation using the PTV parameter is independent of the phenotype to a certain degree, since the phenotype is mainly determined by the protein levels themselves, independently on whether these levels are regulated at the transcriptional or at the post-transcriptional level. For example, a protein could be found at the same levels in the Δ*crc* mutant comparing to the wild type strain, and therefore, the Δ*crc* mutant could present the same phenotype as the parental strain. However, the transcript of this protein could be found repressed in the Δ*crc* mutant, and so, the analysis by PTV parameter reveals a post-transcriptional regulation by Crc.

**Figure 3:**
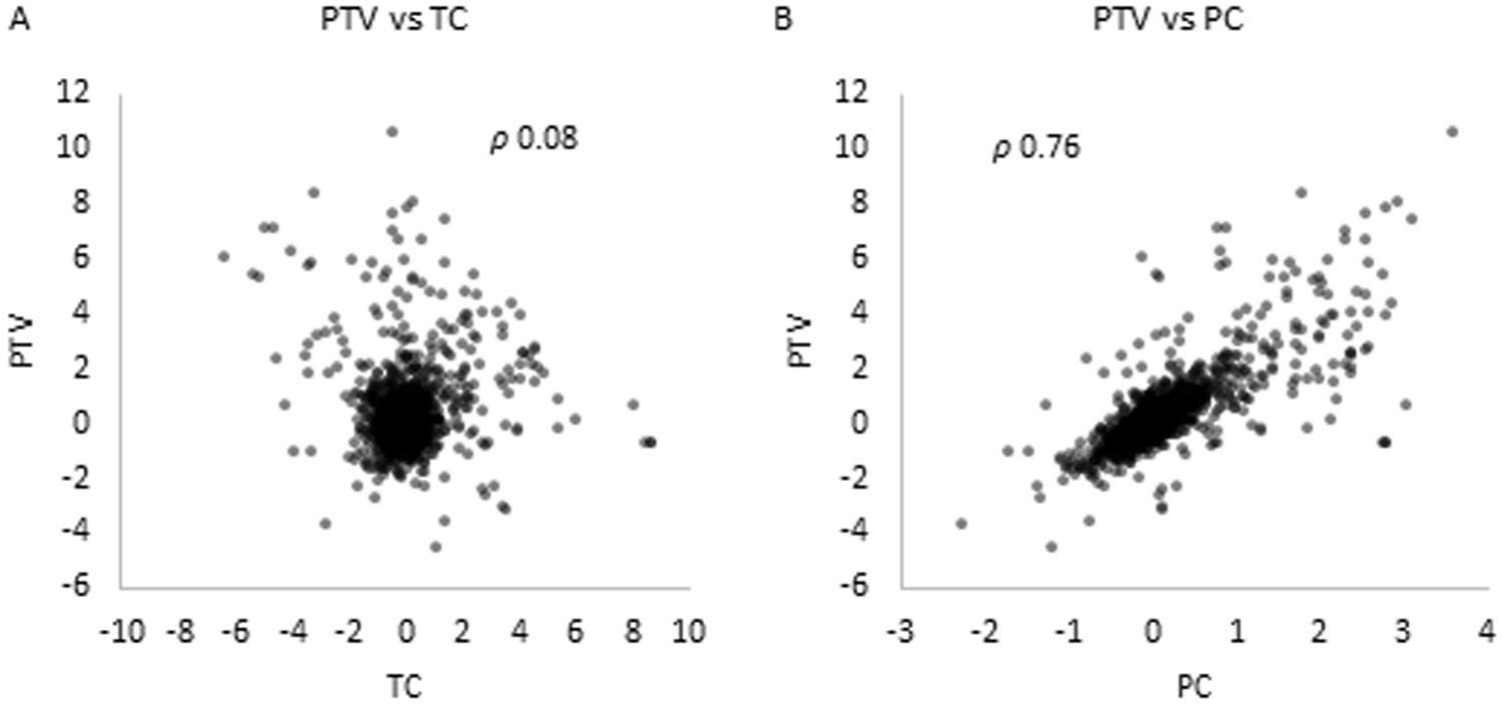
Relationship between PTV and either TC or PC. (**A**) PTV vs TC. The values of PTV are plotted against TC. The Spearman coefficient is indicated. (**B**) PTV vs PC. The values of PTV are plotted against PC. The Spearman coefficient is indicated.

Since it is not always possible to perform both proteomic and transcriptomic experiments, in order to address which one of these analysis is a better indicator of post-transcriptional regulation, a correlation analysis was done by calculating the Spearman coefficient between either the proteomic or the transcriptomic data with the PTV parameter. The PTV parameter presents a better correlation with the proteomic data (PC) than with the transcriptomic data (TC) with a Spearman coefficient of 0.76 for PC data and 0.08 for TC values (Fig. 3). Thus, the proteomic changes are more representative of the post-transcriptional action of Crc than the transcriptional ones.

**Figure 4:**
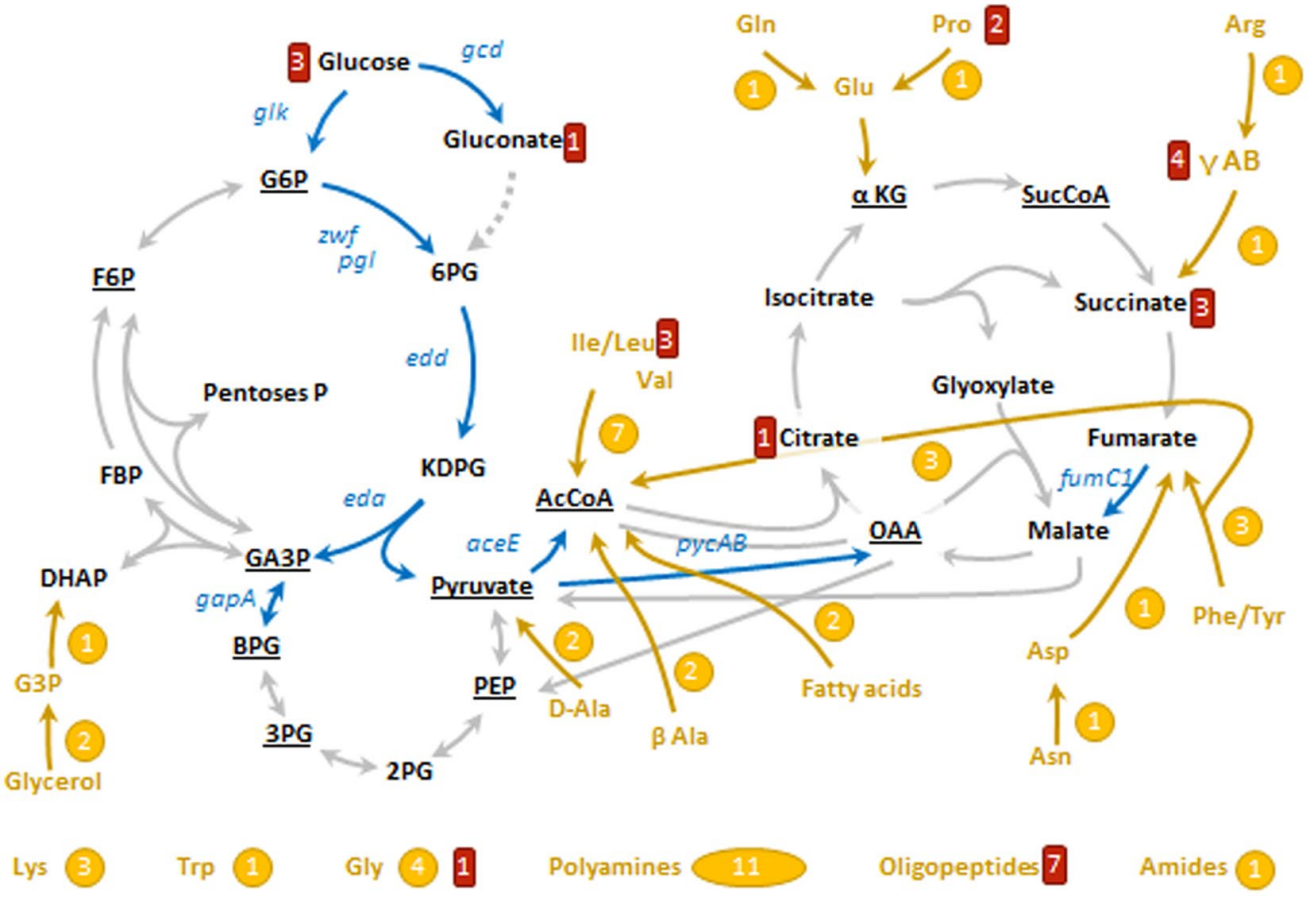
Targets of Crc on bacterial metabolism and on the transport of carbon sources. The diagram represents schematically the main pathways of central carbon metabolism, some pathways of carbon sources catabolism, some of which elements are regulated by Crc, and transporters repressed by Crc. In black, key metabolites within central carbon metabolism, underlined the 12 precursors of biomolecules^30^. In blue, genes encoding enzymes of central carbon metabolism which translation repress the protein Crc (PTV > 1), including Zwf (glucose-6-P 1-dehydrogenase), PgI (6-phosphogluconolactonase), Edd (6-phosphogluconate dehydratase), Eda (KDPG aldolase), Glk (glucokinase), Gcd (glucose dehydrogenase), GapA (GA3P dehydrogenase), AceE (pyruvate dehydrogenase), PycAB (pyruvate carboxylase), and FumC1 (fumarase). In grey, other pathways of central carbon metabolism that are not controlled by Crc. In yellow, secondary carbon sources which degradation is controlled by Crc, including a simplified pathway indicating its connection to central carbon metabolism and the number of enzymes which production is controlled by Crc. In red, the number of proteins related with the transport of such carbon source. This scheme has been done with the information of the databases Pseudomonas Genome database^61^, MetaCyc^62^ and TransportDB^63^ and the information of the bibliography, specially refs^38,64^. G6P, glucose-6-P; F6P, fructose-6-P; FBP, fructose-1,6-P_2_; GA3P, glyceraldehyde-3-P; DHAP, dihydroxyacetone-P; BPG, 1,3-biphosphoglycerate; 2PG, 2-P-glycerate; 3PG, 3-P-glycerate; PEP, phosphoenolpyruvate; AcCoA, acetyl-coenzyme A; 6PG, 6-P-gluconate; KDPG, 2-keto-3-deoxy-6-P-gluconate; αKG, α-ketoglutarate; SucCoA, succinyl-coenzyme A; OAA, oxaloacetate; E4P, erythrose-4-P; R5P, ribulose-5-P; G3P, glycerol-3-P; D-Ala, D-alanine; β-Ala, β-alanine; γ-AB, γ-aminobutyrate.

The PTV analysis has revealed a total of 244 genes which are candidates for being Crc/Hfq targets. Among them, most of the genes that have been already described to be post-transcriptionally regulated by this system, by using translational fusions were found to present changes in their PTVs above the defined threshold. In particular, we found that the following genes known to be under Crc-mediated posttranscriptional control *zwf* ^28^, *estA*^23^, *bkdA1*, *bkdA2*, *bkdB*^16^, *amiE*^9^, *liuR*^29^ and *phhA*^10^, also present changes in their PTVs in our study. The following genes, also post-transcriptionally regulated by Crc were not detected, either at the protein of mRNA level and their PTVs could not be determined: *popD*^19^, *aroP2*, *bkdR*^23^ and *rbsB*^10^. Information on the PT, PC and PTV values of genes belonging to the central carbon metabolism, secondary carbon sources catabolism and carbon sources transporters, iron assimilation and bacterial auto-aggregation, which are discussed below, is shown in Table 1. The table highlight as well genes recently proposed to be Crc targets^10^ and information of genes forming part of operons that present a similar Crc-mediated regulation. Genes related to central carbon metabolism and acquisition of secondary carbon sources are outlined in Fig. 4.

**Table 1:** Transcriptomic, proteomic and post-transcriptional changes for selected genes.

Category	Name	ID	Description	TC	PC	PTV
Catabolism	***bauC***	**PA0130**	3-Oxopropanoate dehydrogenase	−1,02	1,03	3,94
Catabolism	***bauA***	**PA0132**	Beta-alanine: pyruvate transaminase	−1,36	1,41	5,37
Other	***siaD***	**PA0169**	SiaD	1,27	1,05	1,72
Other	***siaC***	**PA0170**	Hypothetical protein	1,39	0,96	1,33
Other	***siaB***	**PA0171**	Hypothetical protein	1,5	1,44	2,6
Catabolism	*gabT*	PA0266	Delta-aminovalerate aminotransferase	−1,69	−0,21	1,1
Catabolism	***spuA***	**PA0297**	Probable glutamine amidotransferase	−0,39	0,6	2,11
Catabolism	***spuB***	**PA0298**	Glutamylpolyamine synthetase	−0,51	0,53	2,01
Catabolism	***spuC***	**PA0299**	Polyamine:pyruvate transaminase	−0,57	0,31	1,46
Transport	***spuD***	**PA0300**	Polyamine transport protein	−0,02	0,49	1,4
Catabolism	***gcdG***	**PA0446**	Conserved hypothetical protein	1,55	1,43	2,52
Catabolism	***gcdH***	**PA0447**	Glutaryl-CoA dehydrogenase	2,64	1,09	0,48
Iron	*fiuA*	PA0470	Ferrichrome receptor FiuA	−0,24	1,05	3,22
Transport	***agtA***	**PA0603**	AgtA	−6,4	−0,12	6,07
Transport	***agtB***	**PA0604**	AgtB	−5,35	0,05	5,5
Transport	***agtC***	**PA0605**	AgtC	−5,14	0,08	5,36
Iron	*hemO*	PA0672	Heme oxygenase	0,51	2,55	6,75
Catabolism	—	PA0744	Probable enoyl-CoA hydratase/isomerase	1,86	1,28	1,78
Transport	***tctC***	**PA0754**	Hypothetical protein	0,06	2,8	7,9
Transport	***opdH***	**PA0755**	Cis-aconitate porin OpdH	−0,5	2,54	7,72
Catabolism	***putA***	**PA0782**	Proline dehydrogenase PutA	−3,29	1,79	8,38
Transport	***putP***	**PA0783**	Sodium/proline symporter PutP	−0,79	1,58	5,3
Transport	**—**	**PA0789**	Probable amino acid permease	0,38	0,86	2,08
Iron	***pirA***	**PA0931**	Ferric enterobactin receptor PirA	0,29	0,97	2,48
Transport	***tolQ***	**PA0969**	TolQ protein	0,41	1,03	2,52
Transport	***tolR***	**PA0970**	TolR protein	0,33	0,62	1,43
Transport	*gntP*	PA1051	Probable transporter	−2,46	0,33	3,4
Transport	***braG***	**PA1070**	Branched-chain amino acid transport protein BraG	0,94	0,79	1,32
Transport	***braF***	**PA1071**	Branched-chain amino acid transport protein BraF	1,06	1,07	2
Transport	***braC***	**PA1074**	Branched-chain amino acid transport protein BraC	0,96	1,47	3,21
Transport	*fadL*	PA1288	Probable outer membrane protein precursor	−0,13	0,57	1,74
Catabolism	*gbuA*	PA1421	Guanidinobutyrase	−3,52	−0,37	2,45
Transport	*kdpB*	PA1634	Potassium-transporting ATPase	3,46	1,19	−0,06
Catabolism	*pauD* *2*	PA1742	Glutamine amidotransferase class I	−0,68	0,55	2,23
Transport	*oppD*	PA1808	NppC	0,66	0,65	1,18
Catabolism	*ldcA*	PA1818	Lysine decarboxylase	1,28	1,49	2,96
Transport	*modA*	PA1863	Molybdate-binding periplasmic protein precursor ModA	0,21	0,76	1,94
Catabolism	***dhcA***	**PA1999**	Dehydrocarnitine CoA transferase	4,29	2,37	2,46
Catabolism	***dhcB***	**PA2000**	Dehydrocarnitine CoA transferase	4,07	1,99	1,61
Catabolism	***atoB***	**PA2001**	Acetyl-CoA acetyltransferase	3,42	2,44	3,51
Transport	***atoE***	**PA2002**	Conserved hypothetical protein	3,7	2,84	4,37
Catabolism	***maiA***	**PA2007**	Maleylacetoacetate isomerase	3,66	1,86	1,62
Catabolism	***fahA***	**PA2008**	Fumarylacetoacetase	3,24	2,58	4,09
Catabolism	***hmgA***	**PA2009**	Homogentisate 1-2dioxygenase	3,36	1,68	1,43
Catabolism	***liuC***	**PA2013**	Putative 3-methylglutaconyl-CoA hydratase	2,27	1,76	2,74
Catabolism	***liuB***	**PA2014**	Methylcrotonyl-CoA carboxylase	2,37	1,32	1,39
Catabolism	***liuA***	**PA2015**	Putative isovaleryl-CoA dehydrogenase	2,16	2,06	3,69
Catabolism	*pauA4*	PA2040	Glutamylpolyamine synthetase	−1	0,16	1,47
Catabolism	*kynU*	PA2080	Kynureninase KynU	0,48	0,54	1,05
Transport	*opdO*	PA2113	Pyroglutamate porin OpdO	7,97	3,03	0,65
Transport	—	PA2204	Probable binding protein component of ABC transporter	2,01	1,33	1,77
Other	***pslA***	**PA2231**	PslA	1,56	1,16	1,74
Other	***pslD***	**PA2234**	PslD	2,03	1,44	2,06
Other	***pslE***	**PA2235**	PslE	1,93	1,2	1,49
Other	***pslF***	**PA2236**	PslF	2,03	1,08	1,05
Other	***pslH***	**PA2238**	PslH	2,19	1,23	1,3
Catabolism	***bkdA1***	**PA2247**	2-oxoisovalerate dehydrogenase (alpha subunit)	3,61	1,96	5,58
Catabolism	***bkdA2***	**PA2248**	2-oxoisovalerate dehydrogenase (beta subunit)	3,98	2,79	7,95
Catabolism	***bkdB***	**PA2249**	Branched-chain alpha-keto acid dehydrogenase (lipoamide component)	3,99	2,17	6,18
Catabolism	***lpdV***	**PA2250**	Lipoamide dehydrogenase-Val	4,12	2,35	6,69
Transport	**—**	**PA2252**	Probable AGCS sodium/alanine/glycine symporter	−3,32	0,89	5,85
Catabolism	***ansA***	**PA2253**	L-asparaginase I	−2,19	0,31	3,07
CCM/catabolism	***gcd***	**PA2290**	Glucose dehydrogenase	0,8	0,7	1,2
Transport	***oprB2***	**PA2291**	Probable glucose-sensitive porin	1,37	2,56	5,91
Iron	*pvdA*	PA2386	L-ornithine N5-oxygenase	1,39	3,11	7,46
Iron	*pvdF*	PA2396	Pyoverdine synthetase F	0,58	2,01	5,14
Iron	*fpvA*	PA2398	Ferripyoverdine receptor	1,2	1,11	1,96
Iron	*pvdD*	PA2399	Pyoverdine synthetase D	1,14	0,93	1,52
Catabolism	***gcvT2***	**PA2442**	Glycine cleavage system protein T2	2,63	1,7	2,2
Catabolism	***glyA2***	**PA2444**	Serine hydroxymethyltransferase	2,37	1,15	0,9
Catabolism	***gcvP2***	**PA2445**	Glycine cleavage system protein P2	2,51	1,98	3,13
Catabolism	***gcvH2***	**PA2446**	Glycine cleavage system protein H2	2,68	2,37	4,08
Catabolism	—	**PA2552**	Probable acyl-CoA dehydrogenase	3,27	1,72	1,61
Catabolism	—	**PA2553**	Probable acyl-CoA thiolase	4,09	2,36	2,63
Transport	*oprQ*	PA2760	OprQ	0,33	0,89	2,21
Catabolism	*pauB3*	PA2776	FAD-dependent oxidoreductase	−1,55	0,2	2,13
CCM/catabolism	***eda***	**PA3181**	2-keto-3-deoxy-6-phosphogluconate aldolase	−0,29	1,6	4,84
CCM/catabolism	***pgl***	**PA3182**	6-phosphogluconolactonase	−0,64	1,71	5,53
CCM/catabolism	***zwf***	**PA3183**	Glucose-6-phosphate 1-dehydrogenase	−1,18	1,63	5,83
Transport	***oprB***	**PA3186**	Glucose/carbohydrate outer membrane porin OprB precursor	2,35	2,76	5,5
Transport	***gltK***	**PA3187**	Probable ATP-binding component of ABC transporter	−0,22	2,29	6,75
Transport	***gltG***	**PA3188**	Probable permease of ABC sugar transporter	−0,46	3,56	10,6
Transport	***gltB***	**PA3190**	Probable binding protein component of ABC sugar transporter	0,2	2,92	8,1
CCM/catabolism	***glk***	**PA3193**	Glucokinase	0,02	1,11	3,15
CCM/catabolism	***edd***	**PA3194**	Phosphogluconate dehydratase	−1,07	1,11	4,22
CCM	***gapA***	**PA3195**	Glyceraldehyde 3-phosphate dehydrogenase	−0,1	2,08	6,02
Transport	—	PA3271	Probable two-component sensor	−2,75	−0,32	1,85
Catabolism	*pauA5*	PA3356	Glutamylpolyamine synthetase	−0,85	0,13	1,21
Catabolism	*amiE*	PA3366	Aliphatic amidase	1,95	1,72	2,95
Catabolism	—	PA3579	Probable carbohydrate kinase	−1,76	−0,23	1,1
Catabolism	*glpK*	PA3582	Glycerol kinase	−1,93	1,44	6,02
Catabolism	*glpD*	PA3584	Glycerol-3-phosphate dehydrogenase	−2,87	0,16	3,31
Transport	—	PA3690	Probable metal-transporting P-type ATPase	−0,46	1,02	3,36
Transport	—	PA3760	N-Acetyl-D-Glucosamine phosphotransferase system transporter	−0,5	0,44	1,76
Transport	—	PA3779	Hypothetical protein	0	0,89	2,54
Transport	—	PA3838	Probable ATP-binding component of ABC transporter	−0,44	0,49	1,84
Iron	*fecA*	PA3901	Fe(III) dicitrate transport protein FecA	−0,44	1,36	4,31
Catabolism	—	PA3925	Probable acyl-CoA thiolase	0,33	0,85	2,1
Catabolism	—	PA4198	Probable AMP-binding enzyme	−0,83	0,88	3,32
CCM	*fumC1*	PA4470	Fumarate hydratase	−0,24	1,3	3,71
Transport	—	PA4496	Probable binding protein component of ABC transporter	1,45	1,72	3,44
Transport	—	**PA4500**	Probable binding protein component of ABC transporter	3,37	2,33	3,27
Transport	***opdP***	**PA4501**	Glycine-glutamate dipeptide porin OpdP	4,52	2,52	2,66
Transport	—	**PA4502**	Probable binding protein component of ABC transporter	4,52	2,58	2,83
Transport	—	**PA4503**	Dipeptide ABC transporter permease DppB	4,43	2,3	2,12
Transport	—	**PA4504**	Dipeptide ABC transporter permease DppC	4,85	2,37	1,88
Transport	—	**PA4505**	Dipeptide ABC transporter ATP-binding protein DppD	4,61	2,35	2,08
Transport	—	**PA4506**	Dipeptide ABC transporter ATP-binding protein DppF	4,55	2,16	1,59
Iron	***piuB***	**PA4513**	Probable oxidoreductase	0,66	0,84	1,73
Iron	***piuA***	**PA4514**	Probable outer membrane receptor for iron transport	0,87	1,99	4,8
Iron	*piuC*	PA4515	Conserved hypothetical protein	0,73	0,93	1,91
Iron	*chtA*	PA4675	ChtA	−0,11	1,03	3,03
Iron/transport	***phuT***	**PA4708**	Heme-transport protein	0,22	1,91	5,22
Iron/transport	***phuS***	**PA4709**	PhuS	0,29	1,98	5,33
Iron/transport	*phuR*	PA4710	Heme/Hemoglobin uptake outer membrane receptor PhuR precursor	−0,46	2,31	7,03
CCM	*aceE*	PA5015	Pyruvate dehydrogenase	0,5	0,56	1,09
Transport	—	**PA5152**	Probable ATP-binding component of ABC transporter	−0,09	1,2	3,51
Transport	—	**PA5153**	Amino acid (lysine/arginine/ornithine/histidine/octopine) ABC transporter periplasmic binding protein	0,8	1,32	2,95
Transport	***dctP***	**PA5167**	DctP	2,07	2,44	4,87
Transport	***dctQ***	**PA5168**	DctQ	1,98	2	3,72
Transport	—	PA5217	Probable binding protein component of ABC iron transporter	−0,01	0,83	2,37
Catabolism	***dadX***	**PA5302**	Catabolic alanine racemase	1,34	1,41	2,67
Catabolism	***dadA***	**PA5304**	D-amino acid dehydrogenase	0,99	1,32	2,76
Catabolism	*pauB4*	PA5309	FAD-dependent oxidoreductase	−0,73	0,14	1,14
Catabolism	*pauC*	PA5312	Aldehyde dehydrogenase	−1,49	0,2	2,07
Transport	***pstB***	**PA5366**	ATP-binding component of ABC phosphate transporter	−0,44	0,24	1,13
Transport	***pstA***	**PA5367**	Membrane protein component of ABC phosphate transporter	0	0,5	1,42
Transport	***pstS***	**PA5369**	Phosphate ABC transporter	−0,22	0,28	1,02
Catabolism	*aspA*	PA5429	Aspartate ammonia-lyase	−0,05	0,6	1,76
CCM	***pycB***	**PA5435**	Probable transcarboxylase subunit	1,69	1,05	1,29
CCM	***pycA***	**PA5436**	Probable biotin carboxylase subunit of a transcarboxylase	1,09	0,88	1,4
Transport	—	PA5504	D-methionine ABC transporter membrane protein	−0,19	0,29	1,01
Catabolism	***pauA7***	**PA5508**	Glutamylpolyamine synthetase homologue	−4,03	0,8	6,32
Transport	—	**PA5510**	Probable transporter	−2,56	0,44	3,82
Catabolism	—	PA5523	Probable aminotransferase	−0,55	0,33	1,49
Iron	*tonB1*	PA5531	TonB1	0,28	1,18	3,08
Transport	—	PA5545	Conserved hypothetical protein	−0,19	0,86	2,64

ID: Gene code as annotated in http://pseudomonas.com, TC: transcript change; PC: protein change; PTV: post-transcriptional variation parameter. CCM: Central carbon metabolism. Underlined, genes that have been described as potential Crc targets in^10^. Highlighted in bold contiguous genes, transcribed in the same strand, which can form operons.

### Potential Crc targets in central carbon metabolism, catabolism of secondary carbon sources and transporters

Central carbon metabolism is defined as the set of metabolic reactions that are needed to produce energy and the precursors of biomass^30^. Since these reactions are required independently of the carbon source present in the medium, central carbon metabolism is not a subject of catabolite repression control *a priori*. Although the assimilation of glucose generally defines the central carbon metabolism, glucose is a secondary carbon source in *P. aeruginosa*^31^. There are two pathways that allow the assimilation of glucose, the phosphorylative branch and the oxidative branch^32^. According to their PTV, and consistent with previous findings^33^, Crc/Hfq seems to control the translation of two enzymes belonging to these pathways, glucokinase (PTV^Glk^ = 3.15) and glucose dehydrogenase (PTV^Gcd^ = 1.20). Once glucose is incorporated in the central carbon metabolism in the form of glucose-6-P, this intermediate is not processed through the Embden-Meyerhof-Parnas (EMP) pathway due to the fact that *P. aeruginosa* (as other *Pseudomonas* species) lacks the key glycolytic enzyme 6-phosphofructo-1-kinase. Glucose-6-P is processed by the enzymes glucose-6-P dehydrogenase (Zwf) and phosphogluconolactonase (Pgl), which yield 6-P-gluconate. In agreement with previous findings^28,33^, both enzymes are strongly repressed by Crc (PTV^Zwf^ = 5.83, PTV^Pgl^ = 5.53)^33^. Zwf is a key enzyme of the bacterial physiology because its activity produces NADPH, an essential cofactor for detoxifying systems implied in oxidative stress defence^34^. Under an oxidative stress challenge the enzyme Zwf is overproduced. Conversely, its inactivation leads to increased susceptibility to oxidative stress in several bacterial species^35–37^. Thus, Zwf regulation by Crc could go beyond the mere assimilation of glucose and it is likely to result in alterations in the cellular redox state.

6-P-gluconate is processed through the Entner-Doudoroff (ED) pathway, composed by two enzymes, 6-P-gluconate dehydratase (Edd), and 2-keto-3-deoxy-6-P-gluconate dehydrogenase (Eda). Both enzymes seem to be repressed by Crc (PTV^Edd^ = 4.84, and PTV^Eda^ = 4.22). In glucose-grown *Pseudomonas* species, the ED pathway enzymes act in concert with Zwf and components of the (incomplete) EMP in a cyclic operation, known as EDEMP cycle. A fraction of the pool of trioses-P is converted into hexoses-P, and processed again through Zwf in order to maintain appropriate NADPH levels^38^. It is thus possible that Crc/Hfq is affecting the availability of reducing power necessary to cope with oxidative stress.

Other enzymes from central carbon metabolism that seem to be repressed by Crc are glyceraldehyde-3-P dehydrogenase (GapA), that belongs to the EMP pathway (PTV^GapA^ = 6.02), fumarase (FumC1), which belongs to the tricarboxylic acid cycle (PTV^FumC1^ = 3.71) and components of the pyruvate dehydrogenase complex (AceE), which mediates the transformation of pyruvate into acetyl-CoA under aerobic conditions (PTV^AceE^ = 1.09). The enzyme pyruvate carboxylase (PycAB), which catalyses the anaplerotic reaction from pyruvate to oxaloacetate, is also likely repressed by Crc (PTV^PycA^ = 1.40, PTV^PycB^ = 1.09). Altogether, these findings indicate that the Crc-dependent regulatory pattern is compatible with a fine-tune modulation of glycolytic fluxes around the pyruvate/acetyl-CoA node (Fig. 4).

Since Crc modulates catabolite repression in *Pseudomonas*, the assimilation of secondary carbon sources is tightly controlled by this protein. For example, it has been described that the aliphatic amidase AmiE is repressed by Crc/Hfq, and actually *amiE* is a prototype gene used for molecular studies involving Crc^11,33^. In agreement with these findings, AmiE presents a PTV of 2.95. It has also been described that Crc modulates the translation of the proteins of the *bkd* operon, which controls the assimilation of branched amino acids^16^. This regulation is confirmed through the analysis of the PTV parameter (PTV^BkdA1^ = 5.58, PTV^BkdA2^ = 7.95, PTV^BkdB^ = 6.18, PTV^LpdV^ = 6.69). Glycerol is another secondary carbon source whose assimilation seems to be controlled by Crc at the level of glycerol kinase (Glpk) and glycerol phosphate dehydrogenase (GlpD) (PTV^GlpK^ = 6.02; PTV^GlpD^ = 2.31).

The PTV analysis suggests that amino acids assimilation is globally controlled by Crc. A feature described as well for *P. putida*^22^. Crc/Hfq controls at least one enzyme of the catabolic assimilation of Ala, Val, Leu, Ile, Gly, Gln, Pro, Arg, Phe, Tyr, Asp, Asn, Lys and Trp. For instance, in addition to the aforementioned proteins of the *bkd* operon, Crc represses the bifunctional enzyme proline dehydrogenase, which converts proline in glutamate (PTV^PutA^ = 8.38). The global control of Crc includes enzymes that take part in the degradation of non-proteinogenic amino acids such β-alanine, δ-aminovalerate, γ-aminobutyrate and D-amino acids. Other substrates whose assimilation seems to be controlled by Crc are fatty acids and polyamines (Table 1).

Crc also controls the production of carbon sources transporters (Table 1). The transporters controlled by Crc belong to all functional and structural families, for example, Crc controls the production of the *P. aeruginosa* ABC transporter for glucose GltG (PTV^GltG^ = 10.80) and the proline transporter PutP (PTV^PutP^ = 5.30). In agreement with previous reports^39^, Crc does not only control the secondary carbon sources transporters, but also the transporters of primary carbon sources as succinate, DctP and DctQ (PTV^DctP^ = 4.87, PTV^DctQ^ = 3.72)^39^. The outer membrane porins are also likely controlled by Crc, including hexoses porins as OprB1 (PTV^OprB1^ = 5.50) and OprB2 (PTV^OprB2^ = 5.91).

It has been described that the regulation over the enzymes and transporters caused by Crc includes the post-transcriptional control of the local regulators, which is known as multi-tier regulation^40^. In agreement with this information, our analysis shows that the regulators PhhR (PTV^PhhR^ = 1.50), DhcR (PTV^DhcR^ = 1.39) and LiuR (PTV^LiuR^ = 1.35) seem to be repressed by Crc, as well as its cognates targets, respectively the operons *phhBCD, dhcAB-atoB, and liuABC* (Table 1). In line with this type or regulation, we found that several genes, presenting increasing PTV values in the *∆crc* mutant, are linked in operons (Table 1), further supporting the internal consistency of our results.

### Crc post-transcriptional regulation goes beyond the use of carbon sources

Iron is a critical element for all living organisms. Although it is an essential co-factor, at high concentrations it is a toxic compound that drives the Fenton reaction^41^. Consequently, the success of a microorganism as *P. aeruginosa*, able of colonizing environments with different iron availability, largely relies on the use of well controlled systems of iron assimilation^42^. *Pseudomonas* has multiple systems for the uptake of iron including self-produced siderophores, such as pyoverdine and pyochelin, as well as systems that are involved in the uptake of the *heme* group and also of xenosiderophores from other species^43^.

According to our analysis, Crc controls the translation of several proteins belonging to the different mechanisms of iron uptake (Table 1). Pyoverdine is the main siderophore of *P. aeruginosa*^44^. The proteins related to pyoverdine production and iron uptake whose translation seems to be controlled by Crc are PvdA, PvdF and PvdD, and the receptor of ferripyoverdine FpvA (PTV^PvdA^ = 7.46, PTV^PvdF^ = 5.14, PTV^PvdD^ = 1.52, PTV^FpvA^ = 1.96). Crc/Hfq also control the translation of the xenosiderophores’ receptors related proteins PiuABC, ChtA, FiuA, PirA and FecA. The proteins PhuT, PhuS and HemO involved *heme* group iron uptake^45^ are also regulated post-transcriptionally by Crc/Hfq. In most of these systems TonB is the protein that energizes the iron uptake coupling its entrance with proton symport^46^ and its expression seems also to be modulated by Crc (PTV^TonB^ = 3.08)^46^. There are more systems involved in iron uptake in *Pseudomonas* besides the Crc-regulated ones, but these genes were not detected at the transcriptional level in our assay, so it was not possible to determine if they are also targets of Crc/Hfq. In addition, our studies have been performed under conditions where iron availability is not restricted, and it would be possible that more elements of the iron homeostasis would be detected when bacteria grow under iron-restricted conditions^43^. The recent finding that the Crc/Hfq system has an important role in keeping iron homeostasis in *P. putida* further supports the linkage between iron and carbon metabolism in *Pseudomonas*^24^.

**Figure 5:**
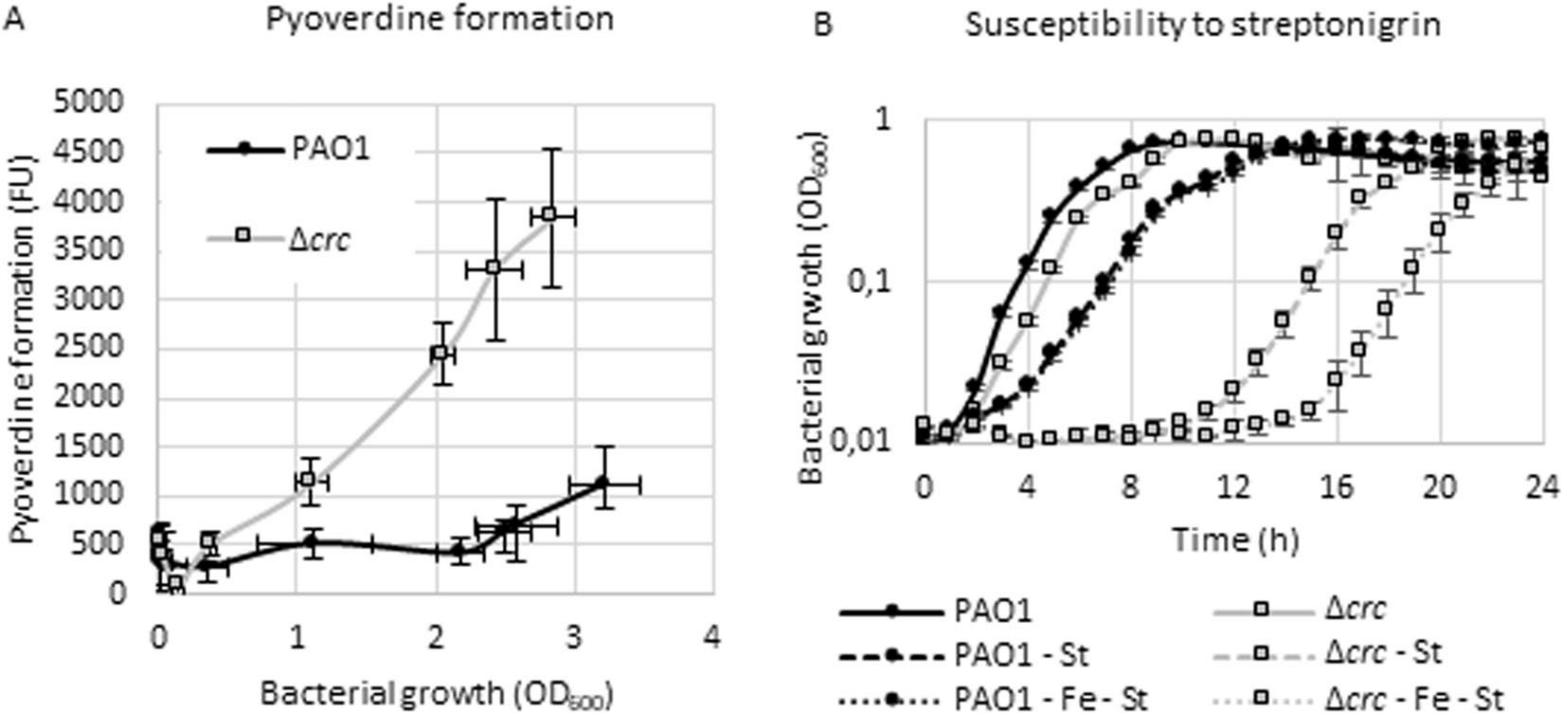
Effect of Crc on iron uptake. (**A**) Pyoverdine formation. Pyoverdine formation by the wild type strain and the Δ*crc* mutant was measured during growth in LB medium. The fluorescence was measured according to reference^60^ and the values are plotted against the bacterial growth. Error bars represent the standard deviation of three biological replicates. (FU: fluorescence units). (**B**) Susceptibility to streptonigrin. The wild type strain and the Δ*crc* mutant strain were grown in LB medium, LB supplemented with 5 µg/ml of streptonigrin (LB - St) and LB supplemented with 5 µg/ml of streptonigrin and 1 mM of FeCl_3_. (LB-Fe-St). Bacterial growth was measured recording the OD_600_ every 10 min, although average values corresponding to three biological replicates each hour are represented in the graph. Error bars represent the standard deviation.

Fur is the main regulator of iron homeostasis in *Pseudomonas*^47^. Our data indicate that the part of the Crc regulon related with iron uptake presents some overlap with the Fur regulon. Nevertheless, no changes on the levels of Fur were detected in the *∆crc* mutant, suggesting the effect of Crc does not simply consists on the regulation of this repressor. To address if the observed post-transcriptional changes observed in the Δ*crc* mutant (and given that same effect is also observed at the protein level; Table 1), have functional consequences, the production of the siderophore pyoverdine was measured in the wild-type PAO1 strains and in the Δ*crc* defective mutant. As shown (Fig. 5A), the Δ*crc* mutant produces more pyoverdine than the parental strain, at least during the first eight hours of growth. To figure out if this overproduction leads to a higher intracellular iron concentration in the mutant, the susceptibility to streptonigrin was determined. Streptonigrin is an antibiotic that acts depending on the intracellular concentration of iron^48^. As shown in Fig. 5B, the Δ*crc* mutant is more susceptible to streptonigrin, and the addition of extracellular iron increases this susceptibility in the Δ*crc* mutant much more than in the parental strain, indicating that the Δ*crc* mutant is able of accumulating higher iron levels than the wild-type strain. Whether or not the accumulation of intracellular iron in *P. aeruginosa* is functionally linked with the optimization of the uptake and degradation of the available carbon sources remains to be established.

Other potential Crc targets are a group of proteins involved in bacterial auto-aggregation, which expression is triggered in response to environmental conditions^49^, leading to a global physiological response called *SDS-stress response*^50^. This response is mediated by the proteins SiaABC and SiaD. SiaBC and SiaD are post-transcriptionally repressed by Crc (PTV^SiaB^ = 2.60, PTV^SiaC^ = 1.33, PTV^SiaD^ = 1.72) (Table 1). SiaD is a di-GMP cyclase, di-GMP is a signal molecule that is involved in multiple cellular regulatory processes. For example, the levels of c-di-GMP controls the transcription of the *cup* and the *psl* operons that encode the proteins involved in the fimbria and exopolysaccharide synthesis respectively. Both of them are transcriptionally overexpressed in the Δ*crc* mutant (Supplementary Data Table S3). In agreement with these findings, previous work showed that a Δ*crc* mutant produces bacterial clumps^21^.

**Figure 6:**
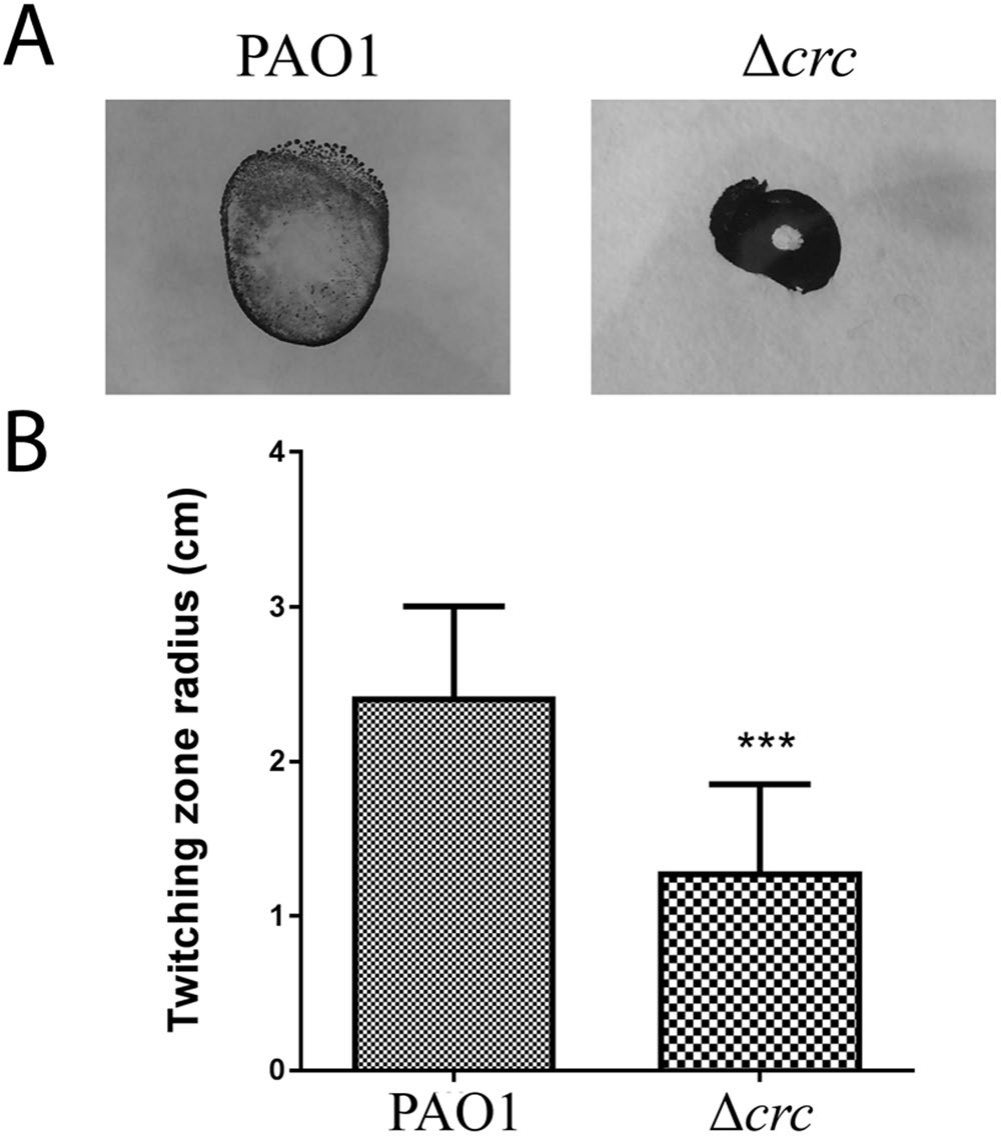
Twitching motility. (**A**) Twitching motility of the wild-type strain and the Δ*crc* mutant was assayed using the subsurface agar method and visualized with Coomassie brilliant blue R250. (**B**) Quantification of the radius (cm) of three independent replicates. The differences were statistically significant (***p < 0.001).

The remaining effects of Crc in the transcriptome or in the proteome are likely fully indirect. These effects are reflected when the PTV parameter is <−1 or in the cases when the proteomic changes are due just to transcriptomic changes. For example, the pili formation-related proteins PilO, N, M, A, X, Y1, E, Z, T, U, G, H and J, have a PTV associated <−1. Pili are the responsible of twitching motility in *P. aeruginosa*^51^. According to these results, the Crc mutants should present lower twitching motility than the parental strain^14^. Consistent with this statement, the twitching motility of the Δ*crc* mutant is half of that shown by the parental strain (Fig. 6).

Regulation of cell respiration, including anaerobic respiration and fermentation processes seem to depend on Crc. *P. aeruginosa* presents five terminal oxidases (Cco1, Cco2, Cyo, Cio and Cox genes) which are tightly regulated according to oxygen availability or the presence of nutrients in the media^52^. Crc inversely modulates the production of the two main terminal oxidases of the electron transport chain, encoded by the operons *cco1* and *cco2*, repressing the translation of Cco1 proteins and promoting the translation of Cco2 ones (Table 1). The transcripts of *cyoABCDE* and *cioAB* presented a lower expression level in the Δ*crc* mutant, althought the encoded proteins were not detected in our analysis (Supplementary Material, Table S3). Other proteins involved in respiration-related processes and presenting PTV parameter <−1 are AcrABC, responsible for the anaerobic fermentation of arginine^53^ are HcnC, that contributes to the biogenesis of cyanide when *P. aeruginosa* grows in microaerophilic conditions and it is also a *P. aeruginosa* important virulence factor^54^. Crc regulation over catabolic enzymes includes membrane dehydrogenases such as DadA, Gcd, PutA and GlpD, that give electrons directly to the electron transport chain; and other dehydrogenases of the metabolism such GapA, that contributes with electrons through the oxidation and reduction of NAD(H). It makes sense that the production of the electron chain components is tightly coordinated with the oxidation of substrates, in this case under regulation by Crc.

## Conclusions

Different approaches have been implemented to identify the post-transcriptional targets of Crc/Hfq in *P. aeruginosa*. This include phenotypic^14^, enzymatic^16,33^ and proteomic^21^ approaches that measure regulation at the proteomic level. Transcriptomic assays have also been used as proxy for studying this system^23^, despite they do not fully take into consideration post-transcriptional regulation. More specific studies on the post-transcriptional effect of Crc/Hfq include the use of translational fusions^9,16,19,23,28,29^. Very recently, the analysis of the binding of nascent mRNAs to Hfq using Chip-Seq approaches^10^, provides information on potential Hfq/Crc mRNA targets, although the functional consequences of the binding og Hfq/Crc to such potential targets requires additional studies.

By the analysis of post-transcriptional regulation using the PTV parameter here defined, we have described 244 genes as putative Crc targets out of a total of over 2000 genes identified by combined transcriptomic and proteomic analysis. This method goes beyond the mere qualitative comparison among proteomic and transcriptomic data^22^, and allow a direct quantification of post-transcriptional regulation in a simpler manner than already available methods^55^. To note here that post-transcriptional fusions, while very valuable, are indirect methods for measuring post-transcriptional regulation and that Chip-Seq analyses provide useful information on potential mRNA targets, not on the post-transcriptional effect of such binding.

Using this parameter, we found several already known targets of Crc/Hfq, which validates the use of PTV for describing translational regulation. In addition, a comprehensive set of potential Crc targets is described. Our results indicate that carbon catabolite repression control in *P. aeruginosa* also involves the regulation of other elements of bacterial physiology, as iron uptake, which should require to be tightly regulated in a free-living organism. This regulation includes the expression of genes at all the main pathways of iron uptake, such as siderophores biosynthesis and uptake, xenosiderophores uptake and group *heme* uptake. Recent work has also shown that CrcZ and Hfq participate in the a riboregulation mechanism that includes the sRNAs PrrF1-2, which are involved in the regulation of iron metabolism, in *P. aeruginosa*^13,56^, although the effect of this regulation in bacterial iron homeostasis has not been explored in detail until now.

It is well established that Crc/Hfq has a major role in catabolite repression control. The current work contributes to widening the regulon of Crc assigning potential directs targets of Crc in *P. aeruginosa* central carbon metabolism, iron uptake and c-di-GMP regulatory network. Crc is considered as a main player in keeping *P. aeruginosa* metabolic homeostasis. In addition, Crc, in cooperation with Hfq, regulates directly or indirectly several of the processes that allows *P. aeruginosa* to colonize a variety of different habitats, each one presenting different physicochemical and nutritional characteristics^15,18^. One of the challenges for understanding *P. aeruginosa* global regulatory processes involved in the adaptation of this microorganism for growing in different habitats, would then be to decipher how all these processes are connected in the Crc/Hfq node.

## Methods

### Bacterial strains and growth conditions

The strains used in this study were *P. aeruginosa* PAO1 (wild-type) conserved by P.V. Phibbs^57^ and FCP001 (Δ*crc* mutant)^20^. The strains were grown in lysogeny broth (LB-Lennox, Pronadisa) at 37 °C with shaking at 250 r.p.m. unless otherwise indicated.

### RNA-seq and real time RT-PCR validation

For RNA-seq analysis, bacterial cells were grown to mid-exponential phase in LB at OD_600_ ≈ 0.6. 10 ml of *RNA protect TM bacterial reagent* (Qiagen) were added to 10 ml of cell culture and incubated 10 min at room temperature. The following steps were the same for RNA samples of RT-PCR and RNA-seq. Briefly, samples were centrifuged (7000 r.p.m. 20 min, 4 °C) and the pellets were resuspended in TE buffer, treated with lysozyme (10 µg/ml) and sonicated [3 cycles of 20 s (Labsonic U Braunn) each one followed by 20 s of incubation on ice]. RNA was extracted using the commercial system *RNA easy minikit* (Qiagen). The DNA of the samples was removed with *RNase-Free DNase Set* (Qiagen) and with *Turbo DNA-free* (Ambion) according to manufacturer’s instructions. Contamination with DNA was checked with RplU primers by PCR^20^. After that, RNA samples for RNA-seq analysis were processed by Parque Científico de Madrid, Unidad Genómica Antonia Martin Gallardo. The samples were treated with *Ribo-Zero Magnetic Kit Bacteria* (Epicentre), which eliminates ribosomal RNA. cDNAs libraries were prepared with *Truseq RNA sample preparation kit v2* (Illumina). Quality of samples and libraries were determined using a *Bioanalyser* (Agilent). The samples were sequenced in a *Genome Analyzer IIx* (Illumina) in single read (1 × 75) format. Sequences were demultiplexed with Casava software (Illumina) and RPKM values were extracted with Rockhopper software^26^. All RPKM = 0 were adjusted to a value of 1 to avoid getting ∞ values when calculating fold changes. The RNA-seq analysis was validated by real time RT-PCR. The samples for RT-PCR were obtained in the same way as described above with the exception of the *RNA protect* addition. RNA samples were converted in cDNA with the *High Capacity cDNA Reverse Transcriptase Kit* (Applied Biosystems). RT-qPCR was performed with 3 biological and 2 technical replicates of the samples. 50 ng of cDNA per well was used with the commercial system *Power-green PCR master mix* (Applied Biosystems) per well. The primers used were ccoO2_F (5′-GCTGGTGGAGAACAAGCTC-3′), ccoO2_R (5′-CGTTTGTTCTTGATCGAGGT-3′) for *ccoO2*, pvdA_F (5′-GTTCACGATCTCATCGGTGT-3′), pvdA_R (5′-ACACCAGGTCCTTGAGGAAG-3′) for *pvdA*, gapA_F (5′-CTACACCAACGACCAGAACC-3′), gapA_R (5′-GACCAGCGACACGTTGAT-3′) for *gapA*, secA_F (5′-AGATCTACGGTCTCGACGTG-3′), secA_R (5′-ACAGCTTGGAAACGTACTCG-3′) for *secA*, glpD_F (5′-GACTACACCCTGTCGCTCTC-3′), glpD_R (5′-GACGCTCTGCATCTGCTC-3′) for *glpD*, and cyoB_F (5′-ACCTGGACATGCACTTCTTC-3′), cyoB_R (5′-CCCAGACCATCGACTTGTAG-3′) for *cyoB*. The RpoN primers^20^ were used for normalization in order to compare the expression levels of each gene among different strains. Fold change was calculated using the 2^−ΔΔCt^ method^58^. Results were presented as the average and standard deviations from the analysis three different replicate biological samples.

### Protein extraction

Three independent cultures of strains PAO1 and FCP001 (20 ml of each) were grown in LB medium to reach mid-exponential growth phase (OD_600_ of 0.6), moment at which the cells were pelleted by centrifugation and cell pellets were frozen at −80 °C until protein extraction.

Cell pellets were resuspended in 1 ml of PBS supplemented with 1/1000 Protease Inhibitor Cocktail (Complete, Mini, EDTA-free Protease Inhibitor Cocktail Tablets) and lysed by sonication (LabSonic U). Cell debris were removed by centrifugation (14000 *g*, 10 min, 4 °C).

### Sample preparation and protein digestion

The proteomic analysis was performed in the proteomics facility of The Spanish National Center for Biotechnology (CNB-CSIC) and at the proteomic facility of the Complutense University-Scientific Park of Madrid (UCM-PCM), both belongs to ProteoRed, PRB2-ISCIII.

Forty µg of protein from each condition were precipitated with methanol/chloroform and resuspended in 0.5 M Triethylammonium bicarbonate (TEAB). The amount of protein in each of the samples was quantified using RC/DC protein assay (BioRad) prior to iTRAQ labelling.

Protein pellets were resuspended and denatured in 20 µl 6 M guanidine hydrochloride/100 mM HEPES, pH 7.5, (SERVA Electrophoresis GmbH), reduced with 2 µL of 50 mM Tris(2-carboxyethyl) phosphine (TCEP, AB SCIEX), pH 8.0, at 60 °C for 60 min and followed by 2 µL of 200 mM cysteine-blocking reagent (methyl methanethiosulfonate (MMTS, Pierce) for 10 min at room temperature. Samples were diluted up to 120 µL to reduce guanidine concentration with 50 mM TEAB. Digestions were initiated by adding 3 µL (1 µg/µL) sequence grade-modified trypsin (Sigma-Aldrich) to each sample in a ratio 1/20 (w/w), which were then incubated at 37 °C overnight on a shaker. Sample digestions were evaporated to dryness in a vacuum concentrator.

### iTRAQ labelling

Digested samples were labelled at room temperature for 2 h with a half unit of iTRAQ Reagent Multi-plex kit (AB SCIEX, Foster City, CA, USA) previously reconstituted with 80 μl of 70% ethanol/50 mM TEAB. The iTRAQ labelling was performed in a 2-plex design for each condition swapping the isobaric tags to compensate for possible variation in label efficiency or accuracy due to tag-specific artefacts, In the first labelling (R1), tags 114 and 115 were used for the PAO1 and Δ*crc* labelling, in the second labelling (R2), tags 115 and 114 were used for labelling the opposite strain and in the last one (R3) tags 116 and 117 were used to label the PAO1 and Δ*crc*, respectively. Finally, samples were combined and labelling reaction was stopped by addition of 100 µl of 50% ACN and further evaporation in a vacuum concentrator was carried out. Labelling reactions were done in triplicate with the different biological samples.

The digested, labelled and pooled peptide mixture was desalted using a Sep-PAK C18 Cartridge (Waters), following manufacture indications; the cleaned tryptic peptides were evaporated to dryness and stored at −20 °C for further analysis.

### Offline high pH reversed-phase (bRP-LC) peptide fractionation

bRP-LC C18 fractionation of the iTRAQ labelled peptides was performed on the SmartLine (Knauer, Germany) HPLC system using the Waters, XBridge C18 column (100 × 2.1 mm, 5 μm particle). The composition of mobile phase (A) was 10 mM ammonium hydroxide (pH 9.4), whereas the composition of mobile phase (B) was 80% methanol, 10 mM ammonium hydroxide (pH 9.3). The dried-up peptide pellet was dissolved in 100 μL of buffer A, injected in the sample loop and then fractionated using a linear gradient of 0–100% buffer B at 150 μL/min for 90 min. Thirty fractions were collected over the elution profile and pooled into 5 fractions using the fraction mixing strategy n + 1 (i.e. fractions 1 + 6 + 11 + 16 + 21 + 26). The peptide fractions were dried, desalted using a SEP-PAK C18 Cartridge (Waters) and stored at −20 °C until the LC−MS analysis.

### Liquid chromatography and mass spectrometer analysis

A 1.5 µg aliquot of each peptide fraction was subjected to 2D-nano LC ESI-MSMS analysis using a nano liquid chromatography system (Eksigent Technologies nanoLC Ultra 1D plus, AB SCIEX, Foster City, CA) coupled to high speed Triple TOF 5600 mass spectrometer (AB SCIEX, Foster City, CA) with a Nanospray III Source. The analytical column used was a silica-based reversed phase column C18 ChromXP 75 µm × 15 cm, 3 µm particle size and 120 Å pore size (Eksigent Technologies, AB SCIEX, Foster City, CA). The trap column was a C18 ChromXP (Eksigent Technologies, AB SCIEX, Foster City, CA), 3 µm particle diameter, 120 Å pore size, switched on-line with the analytical column. The loading pump delivered a solution of 0.1% formic acid in water at 2 µl/min. The nano-pump provided a flow-rate of 300 nl/min and was operated under gradient elution conditions, using 0.1% formic acid in water as mobile phase A, and 0.1% formic acid in acetonitrile as mobile phase B. Gradient elution was performed using a 120 minutes gradient ranging from 2% to 90% mobile phase B. Injection volume was 5 µl.

Data acquisition was performed with a TripleTOF 5600 System (AB SCIEX, Concord, ON). Data was acquired using an ionspray voltage floating (ISVF) 2800 V, curtain gas (CUR) 20, interface heater temperature (IHT) 150, ion source gas 1 (GS1) 20, declustering potential (DP) 85 V. All data was acquired using information-dependent acquisition (IDA) mode with Analyst TF 1.5 software (AB SCIEX, USA). For IDA parameters, 0.25 s MS survey scan in the mass range of 350–1250 Da were followed by 30 MS/MS scans of 150 ms in the mass range of 100–1800 (total cycle time: 4.04 s). Switching criteria were set to ions greater than mass to charge ratio (m/z) 350 and smaller than m/z 1250 with charge state of 2–5 and an abundance threshold of more than 90 counts (cps). Former target ions were excluded for 20 s. IDA rolling collision energy (CE) parameters script was used for automatically controlling the CE.

### Data analysis

MS and MS/MS data were processed using Analyst® TF 1.6 Software (AB SCIEX). Raw data file conversion tools generated mgf files which were also searched against the *P. aeruginosa* (PAO1) database (http://pseudomonas.com), containing 5563 protein sequences and their corresponding reversed entries using the Mascot Server v. 2.5.0 (Matrix Science, London, UK). Search parameters were set as follows: enzyme, trypsin; allowed missed cleavages, 1; fixed modifications, iTRAQ4plex (N-term and K) and beta-methylthiolation of cysteine; variable modifications, oxidation of methionine. Peptide mass tolerance was set to ±25 ppm for precursors and 0.05 Da for fragment masses. The confidence interval for protein identification was set to ≥95% (p < 0.05) and only peptides with an individual ion score above the 1% False Discovery Rates (FDR) threshold were considered correctly identified. Only proteins having at least two quantitated peptides of different primary sequence (unique peptides) were considered in the quantitation.

The mass spectrometry proteomics data have been deposited to the ProteomeXchange Consortium via the PRIDE^59^ partner repository with the dataset identifier PXD009809.

### Measurement of pyoverdine production

Pyoverdine production was measured by fluorometry following the protocol described by Hoegy and coworkers adapting the method to microtiter plates^60^. Bacterial strains were grown in a 20 ml flask with LB media, starting with an OD_600_ = 0,01. Each hour OD_600_ was measured and 1 ml of the culture was centrifuged (7000 r.p.m., 3 min, RT). The supernatant was centrifuged twice. 10 µl of supernatant from the last centrifugation was mixed with 90 µl of 50 mM of Tris-HCl buffer at pH 8. The fluorescence was measured at λ_ex_ 400 nm and λ_em_ 447 nm.

### Susceptibility test to streptonigrin

In a microtiter plate the bacterial strains were inoculated in 150 µl of media at starting OD_600_ = 0.01. The media were LB supplemented with 5 µg/ml of streptonigrin (Sigma), and LB supplemented with 5 µg/ml of streptonigrin and 1 mM of FeCl_3_. The OD_600_ was recorded each 10 min.

## Electronic supplementary material

Supplementary Information
