# Supplementary material for: The development of a new parameter for tracking post-transcriptional regulation allows the detailed map of the Pseudomonas aeruginosa Crc regulon
Source: Sci Rep. 2018 Nov 14;8:16793. doi: 10.1038/s41598-018-34741-9 (PMC6235884; doi:10.1038/s41598-018-34741-9)
Supplement: Supplementary file 1 — Supplementary Information [file 41598_2018_34741_MOESM1_ESM.pdf]

The development of a new parameter for tracking post-transcriptional regulation allows the detailed map of the *Pseudomonas aeruginosa* Crc regulon.

Fernando Corona, Jose Antonio Reales-Calderón, Concha Gil, José Luis Martínez

Supplementary information.

Table S1. RPKM values of all *P. aeruginosa genes*

Transcription Start	Translation Start	Translation Stop	Transcription Stop	Strand	Name	Synonym	RPKM PAO1	RPKM *Δ crc*
456	483	2027	2027	+	dnaA	PA0001	529	559
2028	2056	3159	3159	+	dnaN	PA0002	717	651
3160	3169	4278		+	recF	PA0003	486	504
	4275	6695	6713	+	gyrB	PA0004	665	579
7802	7791	7018	7006	-	lptA	PA0005	140	176
8353	8339	7803	7803	-	-	PA0006	172	132
8463	8671	10377	10433	+	-	PA0007	25	33
	12488	10434	10434	-	glyS	PA0008	349	295
13435	13435	12488		-	glyQ	PA0009	380	341
13436	13540	14091	14234	+	tag	PA0010	39	32
14235	14235	15122	15123	+	-	PA0011	130	126
15181	15207	15473	15473	+	-	PA0012	123	100
15480	15620	16273	16284	+	-	PA0013	71	94
16898	16607	16335	16285	-	-	PA0014	26	34
17223	17217	16900	16899	-	-	PA0015	99	95
18765	18739	17366	17224	-	trkA	PA0016	153	150
	20071	18767	18767	-	-	PA0017	120	116
21066	21012	20068		-	fmt	PA0018	111	120
21619	21573	21067	21067	-	def	PA0019	600	660
21620			21665	?	-	predicted RN	380	514
21672	21712	22737	22737	+	-	PA0020	391	207
	22872	23960		+	-	PA0021	5	4
23997	24001	24558	24558	+	-	PA0022	139	132
25589	25545	24568	24559	-	qor	PA0023	58	45
25715	25736	26653	26710	+	hemF	PA0024	227	256
26711	26711	27535	27535	+	aroE	PA0025	98	108
27536	27646	28632		+	plcB	PA0026	124	31
	28613	29899		+	-	PA0027	54	17
	29896	30498		+	-	PA0028	38	11

	32055	30502		-	-	PA0029	9	4
	32983	32060		-	-	PA0030	6	4
	34511	33000		-	betC	PA0031	15	10
	34624	35538		+	-	PA0032	28	20
36277	36270	35905	35539	-	-	PA0033	49	29
	36901	36278		-	-	PA0034	22	20
	37893	37087	36902	-	trpA	PA0035	62	34
39098	39098	37890		-	trpB	PA0036	94	45
39099	39202	40089	40189	+	trpI	PA0037	44	29
40190	40190	40405	40550	+	-	PA0038	114	56
40551			40560	?	-	predicted RN	455	481
40561	40589	40816	40845	+	-	PA0039	752	1098
40987	41113	42801	42913	+	-	PA0040	37	38
	42914	53521		+	-	PA0041	29	24
56460	56546	56941	57042	+	-	PA0042	71	80
	58594	57212		-	-	PA0043	11	8
	58786	60159		+	exoT	PA0044	69	17
60655	60656	61342	61342	+	-	PA0045	387	251
61343	61373	61726	61768	+	-	PA0046	508	335
61858	61879	62388	62388	+	-	PA0047	295	198
62805	62786	62403	62389	-	-	PA0048	105	150
65023	64729	63068	62899	-	-	PA0049	15	112
	65339	65479		+	-	PA0050	8	11
	66303	68135		+	phzH	PA0051	3	2
	68616	68188		-	-	PA0052	9	4
68857	69272	69526	69542	+	-	PA0053	126	104
70091	70091	69543	69543	-	-	PA0054	69	48
70667	70636	70130	70093	-	-	PA0055	118	102
	71622	70702		-	-	PA0056	12	10
	71730	72617		+	-	PA0057	7	9
	72680	73384		+	-	PA0058	6	5
	73468	73923		+	osmC	PA0059	8	7

74001	74034	74267	74267	+	-	PA0060	96	74
74772	74716	74279	74268	-	-	PA0061	52	38
	75189	74773		-	-	PA0062	18	18
75201	75281	76408	76415	+	-	PA0063	61	49
77411	77399	76416	76416	-	-	PA0064	87	75
	78097	77432	77412	-	-	PA0065	90	95
78640	78632	78090		-	-	PA0066	119	112
78706	78710	80755		+	prlC	PA0067	223	163
	80752	81027	81047	+	-	PA0068	210	169
81048	81116	82174	82388	+	-	PA0069	52	37
83379	83318	82404	82392	-	-	PA0070	791	824
	85092	83380	83380	-	-	PA0071	107	107
	86284	85085		-	-	PA0072	35	43
	87003	86284		-	-	PA0073	56	57
90098	90098	87000		-	ppkA	PA0074	78	70
90843	90834	90106	90099	-	pppA	PA0075	82	99
	91524	90844	90844	-	-	PA0076	94	101
94918	94826	91521		-	icmF1	PA0077	109	115
96401	96397	95048	95014	-	-	PA0078	107	103
97738	97738	96404	96402	-	-	PA0079	149	146
98262	98218	97754	97739	-	-	PA0080	150	158
99756	99756	98263	98263	-	fha1	PA0081	111	104
99952	100124	101158	101158	+	-	PA0082	180	180
101159	101247	101765	101765	+	-	PA0083	526	429
101766	101778	103274	103274	+	-	PA0084	517	464
103282	103350	103838	103838	+	hcp1	PA0085	1397	1207
103839	104006	104851	104852	+	-	PA0086	163	191
104853	104853	105362		+	-	PA0087	110	110
	105359	107218		+	-	PA0088	88	86
	107182	108228		+	-	PA0089	68	63
	108221	110929	110975	+	clpV1	PA0090	143	135
110976	110976	112907	112908	+	vgrG1	PA0091	102	104

	113306	113022	112966	-	-	PA0092	164	168
114604	114595	113303		-	-	PA0093	79	79
115072	115045	114611	114611	-	-	PA0094	131	119
	115299	117524		+	-	PA0095	55	24
	117552	118001		+	-	PA0096	21	10
	117931	119130		+	-	PA0097	18	8
	119127	120164		+	-	PA0098	17	8
	120164	121324	121324	+	-	PA0099	40	21
121325	121346	122266		+	-	PA0100	83	46
	122248	123495		+	-	PA0101	26	13
123866	123871	124599	124681	+	-	PA0102	231	199
	124810	126381		+	-	PA0103	27	16
126447	126518	127114	127118	+	-	PA0104	67	46
	127378	128502		+	coxB	PA0105	19	12
	128512	130104		+	coxA	PA0106	9	5
	130115	130669		+	-	PA0107	6	4
	130680	131567		+	colII	PA0108	5	3
	131792	131583		-	-	PA0109	15	13
	131808	132602		+	-	PA0110	2	2
	132577	133155		+	-	PA0111	2	1
	133220	134293		+	-	PA0112	3	3
	134319	135233		+	-	PA0113	14	8
135234	135259	135894	135897	+	senC	PA0114	99	66
136391	136386	135934	135902	-	-	PA0115	92	58
136518	136518	136991	137247	+	-	PA0116	76	63
	137248	137976	137976	+	-	PA0117	29	22
	138001	138588		+	-	PA0118	34	27
	138818	140167		+	-	PA0119	36	16
	140216	140902		+	-	PA0120	26	27
	141003	141737		+	-	PA0121	20	19
141817	141917	142327	142358	+	-	PA0122	80	45
143267	143267	142359	142359	-	-	PA0123	36	35

	143848	143567	143268	-	-	PA0124	79	70
144095	144072	143845		-	-	PA0125	75	75
144892	144868	144248	144248	-	-	PA0126	246	249
	144969	145469		+	-	PA0127	32	19
145539	145542	145883	145883	+	-	PA0128	222	282
	147392	145965		-	gabP	PA0129	37	31
149102	149054	147561	147553	-	-	PA0130	85	42
	149425	149138		-	-	PA0131	50	21
	150771	149425		-	-	PA0132	59	23
	150906	151823		+	-	PA0133	32	21
	153306	151936		-	-	PA0134	17	16
	153696	153836		+	-	PA0135	2	0
	154417	155988		+	-	PA0136	2	2
	155988	157085		+	-	PA0137	3	2
	157108	158034		+	-	PA0138	8	6
158171	158199	158762	158762	+	ahpC	PA0139	2404	1700
158763	158907	160472	160492	+	ahpF	PA0140	132	141
161502	161448	160552	160497	-	-	PA0141	118	154
	161906	163255		+	-	PA0142	26	19
163361	163363	164415	164442	+	nuh	PA0143	115	90
165099	165069	164443	164443	-	-	PA0144	71	56
165763	165737	165219	165100	-	-	PA0145	50	34
	165976	167073		+	-	PA0146	7	6
	167146	168108		+	-	PA0147	14	7
168152	168213	169163	169163	+	-	PA0148	90	55
169164	169361	169906		+	-	PA0149	25	28
	169903	170898		+	-	PA0150	28	29
	171047	173434		+	-	PA0151	5	4
	173811	174638		+	pcaQ	PA0152	38	20
	174773	175492		+	pcaH	PA0153	6	4
	175503	176108		+	pcaG	PA0154	16	7
176299	176315	177154	177306	+	pcaR	PA0155	56	50

177307	177307	178458		+	-	PA0156	48	47
	178455	179525		+	-	PA0157	50	43
	179522	182569	182569	+	-	PA0158	66	52
182586	182768	183706	183708	+	-	PA0159	59	50
183810	183822	184007	184043	+	-	PA0160	37	97
184226	184287	184439	184476	+	-	PA0161	91	274
184557	184594	185928	185928	+	opdC	PA0162	119	172
186831	186754	185957	185929	-	-	PA0163	50	44
	186832	188448		+	-	PA0164	59	15
188765	189120	189956	189961	+	-	PA0165	299	215
	190198	191604		+	-	PA0166	5	3
191686	191697	192362	192362	+	-	PA0167	106	56
192363	192368	192958	192964	+	-	PA0168	58	44
193672	193672	192965	192965	-	-	PA0169	55	133
194179	194179	193799	193673	-	-	PA0170	53	139
194756	194748	194206	194182	-	-	PA0171	49	139
196814	196748	194757	194757	-	-	PA0172	25	52
	198060	197011		-	-	PA0173	1	2
	198682	198080		-	-	PA0174	2	2
	199530	198688		-	-	PA0175	3	4
	201639	199600		-	aer2	PA0176	7	6
	202160	201675	201675	-	-	PA0177	7	9
	204066	202147		-	-	PA0178	7	7
	204459	204094	204094	-	-	PA0179	27	13
	205829	204657		-	cttP	PA0180	24	19
	206954	206022		-	-	PA0181	38	21
	207071	207823		+	-	PA0182	14	10
	209533	207923		-	atsA	PA0183	11	8
	210460	209621		-	-	PA0184	7	5
	212073	210457		-	-	PA0185	5	4
	212340	213401		+	-	PA0186	8	8
	213819	214634		+	-	PA0187	2	0

	214631	215512		+	-	PA0188	4	4
	216908	215550		-	-	PA0189	4	2
	217156	217881		+	-	PA0190	8	4
	218822	217905		-	-	PA0191	8	6
	219172	221544		+	-	PA0192	3	2
	221585	222487		+	-	PA0193	5	3
	222555	223454		+	-	PA0194	5	3
223742	224101	225219	225294	+	pntAA	PA0195	19	55
	225295	225603		+	pntAB	PA0195.1	24	33
	225603	227039		+	pntB	PA0196	12	29
	227382	228194		+	tonB2	PA0197	1	2
	228223	228942		+	exbB1	PA0198	4	3
	228944	229345		+	exbD1	PA0199	14	10
229747	229738	229526	229487	-	-	PA0200	212	42
229924	229954	230535	230542	+	-	PA0201	56	39
	232000	230543		-	-	PA0202	7	10
	233100	232066		-	-	PA0203	3	10
	233932	233123		-	-	PA0204	5	6
	234849	233929		-	-	PA0205	11	9
	235987	234875		-	-	PA0206	9	14
	236218	237111		+	-	PA0207	19	10
	237232	238896		+	mdcA	PA0208	27	18
	238896	239777		+	-	PA0209	2	10
	239779	240078		+	mdcC	PA0210	2	14
	240071	240934		+	mdcD	PA0211	5	16
	240931	241737		+	mdcE	PA0212	5	14
	241753	242445		+	-	PA0213	6	14
	242442	243374		+	-	PA0214	10	13
	243431	243835		+	-	PA0215	19	12
	243841	244605		+	-	PA0216	7	13
	245619	244690		-	-	PA0217	23	14
	246868	245948		-	-	PA0218	24	13

	247219	248709		+	-	PA0219	4	3
	248767	250200		+	-	PA0220	6	2
	250231	251613		+	-	PA0221	4	5
	251777	252835		+	-	PA0222	1	2
	253794	252913		-	-	PA0223	12	9
	254636	253854		-	-	PA0224	12	11
254637	254791	255330	255480	+	-	PA0225	58	39
	255481	256332		+	-	PA0226	10	11
	256329	257111		+	-	PA0227	7	6
	257108	258313		+	pcaF	PA0228	9	8
	258463	259761		+	pcaT	PA0229	7	4
	259784	261163		+	pcaB	PA0230	17	10
	261179	261970		+	pcaD	PA0231	19	12
261981	261981	262382	262382	+	pcaC	PA0232	22	17
	262557	263498		+	-	PA0233	33	18
	263689	264522		+	-	PA0234	9	13
	266310	264964		-	pcaK	PA0235	5	6
266607	266616	267395	267486	+	-	PA0236	38	44
	267638	268681		+	-	PA0237	1	3
	268704	269519		+	-	PA0238	4	4
	269669	270547		+	-	PA0239	15	13
	271838	270573		-	-	PA0240	8	9
	273229	271904		-	-	PA0241	3	3
	273775	275679		+	-	PA0242	3	3
275754	275772	276440	276479	+	-	PA0243	51	42
	277334	276480		-	-	PA0244	8	5
	277777	277331		-	aroQ2	PA0245	11	8
	279399	277894		-	-	PA0246	31	23
	280758	279574		-	pobA	PA0247	8	9
280772	280936	281802		+	-	PA0248	38	22
	282245	281799		-	-	PA0249	27	29
282781	282757	282323	282246	-	-	PA0250	41	24

	283553	282912		-	-	PA0251	7	4
	284105	283818		-	-	PA0252	18	19
284713	284673	284197	284106	-	-	PA0253	39	34
	286204	284714		-	-	PA0254	13	11
287071	287024	286338	286205	-	-	PA0255	46	37
	288120	287188		-	-	PA0256	22	10
289175	289175	288384	288384	-	-	PA0257	34	24
289392	289390	289205	289176	-	-	PA0258	106	65
291004	291004	289562	289562	-	-	PA0259	101	73
	293304	291154	291044	-	-	PA0260	70	54
	293798	293301		-	-	PA0261	34	27
	296861	293802	293802	-	-	PA0262	24	16
	297561	297043		-	hcpC	PA0263	10	6
298892			298816	-	-	PA0263.1	60	92
299497	299497	299081	299081	-	-	PA0264	163	65
299498	299522	300973	301004	+	gabD	PA0265	314	116
301085			301160	?	-	predicted RN	500	196
301161	301218	302498	302522	+	gabT	PA0266	573	177
302657	302824	304023	304176	+	-	PA0267	54	43
	305598	304177		-	-	PA0268	18	14
	305725	306162		+	-	PA0269	36	19
306174	306174	306581	306581	+	-	PA0270	35	21
	306615	306899		+	-	PA0271	24	25
	307828	306896		-	-	PA0272	32	20
	309092	307878		-	-	PA0273	1	1
	310025	309255		-	-	PA0274	6	2
	310820	310134		-	-	PA0275	41	14
	310896	311411		+	-	PA0276	13	11
312278	312209	311451	311444	-	-	PA0277	216	296
	313133	312381		-	-	PA0278	7	5
	313227	313925	313925	+	-	PA0279	23	16
314930	314927	313938	313926	-	cysA	PA0280	35	56

315800	315800	314931	314931	-	cysW	PA0281	33	41
316790	316629	315811	315801	-	cysT	PA0282	29	31
317797	317789	316791	316791	-	sbp	PA0283	33	40
318169	318148	317966	317899	-	-	PA0284	66	88
320634	320594	318312	318191	-	-	PA0285	77	52
320747	320766	321944	321970	+	desA	PA0286	253	236
	322175	323560		+	gpuP	PA0287	3	2
	323616	324572		+	gpuA	PA0288	9	7
	324625	325587		+	gpuR	PA0289	23	13
	325700	326671		+	-	PA0290	19	10
326848	327284	328666	328674	+	oprE	PA0291	674	604
329909	329907	328801	328791	-	aguA	PA0292	143	127
330933	330871	329993	329993	-	aguB	PA0293	57	64
	331699	331034		-	aguR	PA0294	33	29
332886	332823	331762	331719	-	-	PA0295	121	57
334733	334455	333079	333079	-	spuI	PA0296	327	204
334734	334734	335486	335526	+	spuA	PA0297	150	105
335527	335527	336885	336885	+	spuB	PA0298	159	112
336886	336951	338321	338367	+	spuC	PA0299	268	181
338383			338410	?	-	predicted RN	351	384
338411	338437	339540	339591	+	spuD	PA0300	485	478
339749	339959	341056	341056	+	spuE	PA0301	148	112
341110	341111	342265	342285	+	spuF	PA0302	157	110
342286	342292	343173	343255	+	spuG	PA0303	186	139
343256	343256	344125	344126	+	spuH	PA0304	153	124
	346690	344303		-	-	PA0305	21	14
	346801	347853		+	-	PA0306	37	24
	349051	348440	347854	-	-	PA0307	36	32
	349050	350069	350088	+	-	PA0308	62	51
350857	350841	350089	350089	-	-	PA0309	65	70
350890	350890	351588	351609	+	-	PA0310	66	38
	352164	351610		-	-	PA0311	16	10

352284	352430	352924	352926	+	-	PA0312	92	61
353673	353619	352927	352927	-	-	PA0313	65	56
354538	354461	353691	353680	-	-	PA0314	76	74
354652	354754	355191	355206	+	-	PA0315	282	135
356580	356477	355248	355248	-	serA	PA0316	762	824
356633	356681	358075	358075	+	-	PA0317	292	255
358094	358167	358832	358832	+	-	PA0318	125	78
358833	358931	359920	359981	+	-	PA0319	97	85
	359982	360332		+	-	PA0320	25	23
	361447	360413		-	-	PA0321	6	9
	362857	361463		-	-	PA0322	5	4
	364321	363278		-	-	PA0323	7	6
	365157	364369		-	-	PA0324	8	4
	366083	365154		-	-	PA0325	9	7
	367129	366080		-	-	PA0326	4	2
367457	367457	368422		+	-	PA0327	19	7
	370405	368462	368462	-	-	PA0328	31	7
371072	371038	370706	370706	-	-	PA0329	183	173
371864	371833	371162	371162	-	rpiA	PA0330	236	222
372082	372091	373605	373605	+	ilvA1	PA0331	179	157
373722	373725	374192	374242	+	-	PA0332	93	75
374243	374243	375514	375950	+	-	PA0333	47	43
377189	377189	375951	375951	-	-	PA0334	38	26
377954	377892	377239	377190	-	-	PA0335	106	50
378096	378096	378575	378597	+	ygdP	PA0336	593	491
378598	378598	380877	380877	+	ptsP	PA0337	249	232
380878	380903	382033	382036	+	-	PA0338	41	39
	382792	382037	382037	-	-	PA0339	32	17
382892	382914	383717	383717	+	-	PA0340	40	40
383722	383727	384527	384545	+	lgt	PA0341	115	114
384715	384733	385527	385553	+	thyA	PA0342	215	202
386385	386351	385554	385554	-	-	PA0343	95	90

	387765	386386		-	-	PA0344	21	13
	389143	387758		-	-	PA0345	21	12
389262	389334	389696	389732	+	-	PA0346	39	40
	390884	389733		-	glpQ	PA0347	14	17
	391094	392188		+	-	PA0348	6	6
	392185	393225		+	-	PA0349	5	5
393298	393308	393814	393821	+	folA	PA0350	100	106
394441	394303	393830	393830	-	-	PA0351	85	55
395832	395827	394442	394442	-	-	PA0352	101	123
398047	397895	396057	396057	-	ilvD	PA0353	160	113
399425	399420	398224	398223	-	-	PA0354	134	115
	400032	399493		-	pfpI	PA0355	9	6
400191	400248	401072	401072	+	-	PA0356	128	133
401073	401131	401943	401973	+	mutM	PA0357	117	103
401998	402020	402598	402657	+	-	PA0358	57	131
402663	402681	403025	403025	+	-	PA0359	406	144
403033	403295	404281	404281	+	-	PA0360	63	42
406189	406119	404386	404282	-	-	PA0361	50	43
406498	406498	406247	406205	-	fdx1	PA0362	259	257
407110	407098	406619	406536	-	coaD	PA0363	269	239
	408845	407250		-	-	PA0364	11	11
	409449	408901		-	-	PA0365	15	17
	410934	409504		-	-	PA0366	17	7
411073	411224	411871	411899	+	-	PA0367	61	41
413327	413327	412329	412308	-	-	PA0368	42	34
413877	413654	413364	413345	-	-	PA0369	35	37
	414529	413933	413930	-	-	PA0370	91	88
	416016	414529		-	-	PA0371	81	82
417446	417406	416009		-	-	PA0372	95	91
417508	417527	418894		+	ftsY	PA0373	217	223
	418891	419562		+	ftsE	PA0374	265	273
	419562	420569	420682	+	ftsX	PA0375	204	244

420683	420683	421537	421537	+	rpoH	PA0376	1024	1096
421538	421602	422207	422207	+	-	PA0377	41	48
422972	422943	422212	422208	-	-	PA0378	53	61
422980	422980	423357	423451	+	-	PA0379	80	67
423460	423460	423660	423689	+	-	PA0380	217	278
423690	423719	424516	424523	+	thiG	PA0381	266	282
424582	424670	425341	425503	+	micA	PA0382	199	215
	425504	426850		+	-	PA0383	21	23
	427120	426863		-	-	PA0384	27	27
427515	427505	427182	427121	-	-	PA0385	61	59
428860	428740	427586	427516	-	-	PA0386	42	48
	429454	428861	428861	-	-	PA0387	254	267
429870	429870	429451		-	-	PA0388	250	273
430622	430622	430002	429925	-	-	PA0389	178	171
431772	431769	430630	430629	-	metX	PA0390	186	197
434185	433825	431858	431852	-	-	PA0391	109	46
434821	434819	434226	434205	-	-	PA0392	252	259
435651	435651	434830	434825	-	proC	PA0393	186	178
436569	436355	435663	435652	-	-	PA0394	142	160
436570	436570	437604	437604	+	pilT	PA0395	181	91
437727	437782	438930	438937	+	pilU	PA0396	193	48
	439837	438938	438938	-	-	PA0397	34	18
439989	439991	440395	440404	+	-	PA0398	128	95
440623	440638	442011		+	-	PA0399	142	109
	442008	443192	443364	+	-	PA0400	145	114
	444690	443419	443413	-	-	PA0401	141	132
445691	445691	444687		-	pyrB	PA0402	188	185
446250	446227	445715	445692	-	pyrR	PA0403	102	77
	446773	446339	446281	-	-	PA0404	70	63
447342	447342	446773		-	-	PA0405	154	120
448403	448350	447391	447343	-	tonB3	PA0406	51	34
449571	449384	448431	448431	-	gshB	PA0407	252	102

449581	449639	450046	450046	+	pilG	PA0408	1061	593
450047	450093	450458	450508	+	pilH	PA0409	710	401
450509	450509	451045	451070	+	pilI	PA0410	246	170
451085	451130	453178	453178	+	pilJ	PA0411	426	243
453179	453239	454114	454125	+	pilK	PA0412	130	81
454126	454126	461544		+	chpA	PA0413	170	105
	461537	462568		+	chpB	PA0414	107	74
	462565	463071	463072	+	chpC	PA0415	111	71
463079	463079	463873		+	chpD	PA0416	42	24
	463949	464560		+	chpE	PA0417	22	14
	465983	464568		-	-	PA0418	42	36
466832	466740	466018	466009	-	-	PA0419	88	121
468250	468236	466833	466833	-	bioA	PA0420	115	128
468405	468410	469900	469971	+	-	PA0421	143	168
470081	470081	470650	470650	+	-	PA0422	181	151
470651	470662	471237	471242	+	pasP	PA0423	1119	937
471987	471749	471306	471306	-	mexR	PA0424	96	85
472005	472024	473175	473175	+	mexA	PA0425	307	516
473176	473191	476331	476332	+	mexB	PA0426	302	492
476333	476333	477790	477802	+	oprM	PA0427	303	487
479805	479805	477886	477847	-	-	PA0428	455	370
481193	481126	480056	479829	-	-	PA0429	320	290
482084	482068	481196	481196	-	metF	PA0430	203	190
482705	482665	482111	482085	-	-	PA0431	252	235
484263	484115	482706	482706	-	sahH	PA0432	839	724
	484404	484838		+	-	PA0433	11	17
	484964	487156		+	-	PA0434	5	13
	487167	488645		+	-	PA0435	3	9
488725	488730	489350	489356	+	-	PA0436	146	83
	490658	489387	489387	-	codA	PA0437	107	98
491938	491898	490648		-	codB	PA0438	42	45
	493357	492080		-	-	PA0439	4	2

	494721	493354		-	-	PA0440	7	5
	496255	494816		-	dht	PA0441	6	6
	496478	496362		-	-	PA0442	1	4
	496871	498361		+	-	PA0443	4	2
	498420	499703		+	-	PA0444	9	9
	501120	500104		-	-	PA0445	19	17
502718	502599	501376	501121	-	-	PA0446	30	90
503938	503900	502719	502719	-	gcdH	PA0447	34	202
504039	504121	505029	505046	+	-	PA0448	50	42
505047	505047	505586	505591	+	-	PA0449	202	179
	507251	505629		-	-	PA0450	14	17
	507631	508962		+	-	PA0451	4	2
	508959	509753		+	-	PA0452	4	5
	510499	509825		-	-	PA0453	24	10
	512790	510589		-	-	PA0454	22	20
514438	514427	513051	513044	-	dbpA	PA0455	80	63
514638	514775	514984	515021	+	-	PA0456	13435	8795
	515135	515659		+	-	PA0457	36	26
516027	516027	515653		-	-	PA0457.1	23	24
	517462	516029		-	-	PA0458	29	20
	518083	520635		+	-	PA0459	7	3
520711	520737	521315	521414	+	-	PA0460	91	110
	522517	521630	521630	-	-	PA0461	115	116
	522465	523169	523247	+	-	PA0462	55	35
523250	523254	523943		+	creB	PA0463	79	46
	523943	525367		+	creC	PA0464	30	28
	525469	526827		+	creD	PA0465	8	7
	526877	527179		+	-	PA0466	8	4
527944	527944	527324		-	-	PA0467	33	28
	528913	527963		-	-	PA0468	39	23
528914	528999	529856	529992	+	-	PA0469	62	31
	532437	530029		-	fiuA	PA0470	13	10

	533512	532541	532438	-	-	PA0471	15	16
534058	534027	533509		-	-	PA0472	23	25
534181	534196	534945	535017	+	-	PA0473	52	53
	535085	535489		+	-	PA0474	2	3
	535539	536108		+	-	PA0475	17	16
	537869	536142		-	-	PA0476	11	8
	539143	538217		-	-	PA0477	19	8
	539231	539707		+	-	PA0478	29	20
	540735	539785	539785	-	-	PA0479	16	10
	540839	541636		+	-	PA0480	5	3
541637	541679	542122	542127	+	-	PA0481	31	39
544393	544347	542170	542170	-	glcB	PA0482	218	149
	544654	545097	545097	+	-	PA0483	47	17
	545129	545644	545644	+	-	PA0484	14	9
547250	547230	546334	546230	-	-	PA0485	58	95
548406	548406	547432	547271	-	-	PA0486	47	32
549293	549226	548468	548407	-	-	PA0487	55	25
	549614	549294		-	-	PA0488	14	13
	550381	549656		-	-	PA0489	5	5
	550813	550520		-	-	PA0490	26	23
	551793	550867		-	-	PA0491	23	14
	551911	552669		+	-	PA0492	19	8
552670	552746	552994	553004	+	-	PA0493	29	17
	553005	554381		+	-	PA0494	15	12
554382	554383	555261		+	-	PA0495	27	28
	555251	556228		+	-	PA0496	17	15
	557276	556275		-	-	PA0497	12	15
	558361	557354		-	-	PA0498	20	12
	559078	558377		-	-	PA0499	12	7
559585	559644	560702	560702	+	bioB	PA0500	324	450
560790	560808	562013		+	bioF	PA0501	93	149
	562006	562728		+	-	PA0502	73	111

	562721	563545	563548	+	-	PA0503	54	89
563549	563549	564235	564323	+	bioD	PA0504	33	65
564324	564344	564574	564574	+	-	PA0505	109	75
564795	564914	566719	566742	+	-	PA0506	49	43
	566932	568728		+	-	PA0507	7	3
	569002	570780		+	-	PA0508	32	18
	572592	571111		-	nirN	PA0509	4	16
	573422	572583		-	-	PA0510	4	21
	574596	573433		-	nirJ	PA0511	5	17
	575105	574590		-	-	PA0512	4	25
	575523	575080		-	-	PA0513	3	13
	576040	575516		-	nirL	PA0514	3	18
	576489	576037		-	-	PA0515	7	20
	577676	576498		-	nirF	PA0516	7	36
	578032	577673		-	nirC	PA0517	5	27
	578343	578029		-	nirM	PA0518	22	71
	580100	578394	578394	-	nirS	PA0519	80	84
	580316	581098		+	nirQ	PA0520	19	12
	581133	581660		+	-	PA0521	4	3
	581668	581925		+	-	PA0522	2	0
	582015	582455		+	norC	PA0523	2	5
	582455	583855		+	norB	PA0524	4	2
	583857	585695		+	-	PA0525	6	11
	585896	585702		-	-	PA0526	67	79
586677	586663	585980	585897	-	dnr	PA0527	260	195
586867			586990	+	rsmY	PA0527.1	154	214
	587895	587017		-	-	PA0528	14	10
	587997	588698		+	-	PA0529	24	6
	588924	590105		+	-	PA0530	12	6
	590105	590821		+	-	PA0531	6	5
	591402	590896		-	-	PA0532	17	9
	591445	592935		+	-	PA0533	23	21

593252	593273	594562	594568	+	-	PA0534	21	31
594570	594580	595134	595205	+	-	PA0535	36	46
596273	596270	595245	595245	-	-	PA0536	104	101
596882	596882	596274	596274	-	-	PA0537	222	192
597543	597479	596970	596885	-	dsbB	PA0538	65	41
	597708	598538		+	-	PA0539	7	9
	598608	598994		+	-	PA0540	22	17
599091	599107	599565	599565	+	-	PA0541	174	228
600232	600176	599757	599754	-	-	PA0542	233	213
	600426	601394		+	-	PA0543	8	4
	602141	601398		-	-	PA0544	27	18
	602346	603650		+	-	PA0545	37	73
604911	604896	603706	603695	-	metK	PA0546	885	714
605913	605913	604912	604912	-	-	PA0547	335	342
606127	606160	608157		+	tktA	PA0548	504	459
	608157	609221		+	-	PA0549	119	110
610029	609999	609202		-	-	PA0550	50	43
610197	610214	611275	611275	+	epd	PA0551	140	150
611276	611281	612444	612444	+	pgk	PA0552	390	355
612453	612517	612717	612876	+	-	PA0553	248	231
612877	612877	613218	613299	+	-	PA0554	317	342
613300			613329	?	-	predicted RN	577	574
613330	613338	614402	614402	+	fda	PA0555	830	663
614403	614468	614950	614951	+	-	PA0556	91	84
	615608	614952		-	-	PA0557	25	22
	615607	616374		+	-	PA0558	26	17
617561	617549	616371		-	-	PA0559	48	52
617649	617662	618150	618164	+	-	PA0560	55	40
618165	618176	619330	619330	+	-	PA0561	39	35
620134	620006	619332	619331	-	-	PA0562	58	54
620549	620488	620135	620135	-	-	PA0563	787	657
	621577	620666		-	-	PA0564	23	12

	621695	622033		+	-	PA0565	18	18
622693	622538	622023		-	-	PA0566	45	36
622726	622726	622884	622893	+	-	PA0567	67	61
623352	623352	622894	622894	-	-	PA0568	40	40
623851	623838	623368	623358	-	-	PA0569	37	35
	624199	623852	623852	-	-	PA0570	36	25
624816	624803	624189		-	-	PA0571	39	34
	627765	624994	624994	-	-	PA0572	46	9
	628670	628335		-	-	PA0573	25	9
629956	629884	628763	628671	-	-	PA0574	73	48
630510			630434	-	-	PA0574.1	23	41
	634264	630527		-	-	PA0575	6	6
636240	636224	634371	634360	-	rpoD	PA0576	1343	1348
638380	638298	636304	636244	-	dnaG	PA0577	177	213
638899	638830	638381	638381	-	-	PA0578	415	621
639155	639115	638900	638900	-	rpsU	PA0579	3468	5642
639156			639223	?	-	predicted RN	655	1269
639301	639316	640341	640341	+	gcp	PA0580	141	184
641057	640989	640420	640392	-	-	PA0581	99	89
641065	641073	641426		+	folB	PA0582	74	97
	641417	641959		+	-	PA0583	66	80
	643164	641932		-	cca	PA0584	38	31
	643714	643208		-	-	PA0585	4	4
	645361	643808		-	-	PA0586	13	11
	646629	645358		-	-	PA0587	20	13
	648652	646730	646730	-	-	PA0588	40	29
649306	649263	648931	648791	-	-	PA0589	76	72
	650158	649307	649307	-	apaH	PA0590	96	86
650538	650538	650158		-	-	PA0591	144	127
651421	651381	650575	650551	-	ksgA	PA0592	124	155
	652483	651497	651478	-	pdxA	PA0593	75	75
	653772	652480		-	surA	PA0594	369	374

656577	656527	653753		-	ostA	PA0595	613	597
656635	656654	657670		+	-	PA0596	110	104
	657667	658341	658341	+	-	PA0597	87	84
658342	658343	659101		+	-	PA0598	58	64
	660163	659102		-	-	PA0599	34	29
	660315	662708	662708	+	-	PA0600	26	27
662709	662754	663386	663410	+	-	PA0601	42	36
663510	663515	664549	664555	+	-	PA0602	98	53
664770			664782	?	-	predicted RN	575	2
	664783	665892	665892	+	-	PA0603	677	8
665893			665938	?	-	predicted RN	589	23
	665948	666994		+	-	PA0604	600	15
	667108	668355	668355	+	-	PA0605	176	5
	668461	669291		+	-	PA0606	153	6
669401	669415	670089		+	rpe	PA0607	182	211
	670089	670907	670944	+	-	PA0608	110	148
670945	670980	672458	672473	+	trpE	PA0609	109	138
673091	673091	672777	672609	-	prtN	PA0610	150	133
673961	673961	673191	673092	-	prtR	PA0611	135	89
674096	674419	674619	674619	+	ptrB	PA0612	168	160
674621	674667	675026	675051	+	-	PA0613	219	187
675380	675390	675839	675839	+	-	PA0614	122	85
675840	675861	676376		+	-	PA0615	202	137
	676373	676930	676970	+	-	PA0616	165	94
677083	677083	677409		+	-	PA0617	270	185
	677406	678293		+	-	PA0618	256	177
	678286	678819	678820	+	-	PA0619	271	192
678821	678821	680896		+	-	PA0620	327	226
	680893	681351	681364	+	-	PA0621	297	216
681366	681394	682554	682554	+	-	PA0622	710	472
682555	682567	683070	683071	+	-	PA0623	797	503
683072			683081	?	-	predicted RN	628	429

683082	683085	683429	683455	+	-	PA0624	496	304
683471	683599	685836	685836	+	-	PA0625	305	181
685837	685846	686718		+	-	PA0626	188	133
	686693	686899	686956	+	-	PA0627	165	117
686957	686957	687946	687978	+	-	PA0628	175	131
687979	687979	688608		+	-	PA0629	135	87
	688605	688967		+	-	PA0630	138	98
	688964	689221	689221	+	-	PA0631	111	86
689222	689236	689466	689466	+	-	PA0632	180	121
689467	689537	690031	690032	+	-	PA0633	680	374
690042	690043	690390	690390	+	-	PA0634	510	259
690391	690420	690674	690676	+	-	PA0635	389	198
690704	690721	692556		+	-	PA0636	219	136
	692549	692890	692897	+	-	PA0637	165	96
692898	692898	693593	693593	+	-	PA0638	145	74
693594	693596	694366	694366	+	-	PA0639	126	74
694368	694421	695023	695023	+	-	PA0640	184	123
695024	695082	698696	698696	+	-	PA0641	126	81
698706	698932	699720	699743	+	-	PA0642	126	82
699744	699744	700835		+	-	PA0643	171	113
	700835	701170		+	-	PA0644	219	124
	701151	701381	701476	+	-	PA0645	179	114
701477	701477	702529		+	-	PA0646	246	132
	702529	702831		+	-	PA0647	275	159
	702828	703058	703077	+	-	PA0648	253	153
703230	703477	704082	704082	+	trpG	PA0649	145	132
704083	704084	705133		+	trpD	PA0650	135	115
	705130	705966	705977	+	trpC	PA0651	164	135
706786	706672	706028	706025	-	vfr	PA0652	838	277
706935	706944	707366	707390	+	-	PA0653	162	141
707452	707686	708480	708491	+	speD	PA0654	596	702
709183	709182	708535	708528	-	-	PA0655	187	112

709620	709620	709282		-	-	PA0656	33	19
709621	709699	711180	711219	+	-	PA0657	48	33
712080	712020	711220	711220	-	-	PA0658	84	63
713163	713157	712081	712081	-	-	PA0659	205	153
714247	714247	713279	713171	-	-	PA0660	78	53
714692	714686	714264	714248	-	-	PA0661	130	80
714948	714977	716011		+	argC	PA0662	180	166
	716011	716730		+	-	PA0663	171	129
	716731	717153	717165	+	-	PA0664	211	155
717214	717231	717581	717591	+	-	PA0665	326	271
718726	718726	717635	717635	-	-	PA0666	130	106
720072	720072	718729	718727	-	-	PA0667	296	253
720073			720188	?	-	predicted RN	500	681
720242			720251	?	-	predicted RN	258	386
720344			720354	?	-	predicted RN	360	404
720355	720357	721556	722095	+	tyrZ	PA0668	477	395
722096			723631	+	-	PA0668.1	131	118
723696			723772	+	-	PA0668.2	4629	4940
723801			723876	+	-	PA0668.3	3221	2967
724103			726993	+	-	PA0668.4	148	140
727136			727255	+	-	PA0668.5	9	14
	730703	727608		-	-	PA0669	7	8
	732094	730679		-	-	PA0670	11	11
	732653	732102		-	-	PA0671	8	8
732919	732959	733555	733595	+	hemO	PA0672	30	44
	733993	733673		-	-	PA0673	14	11
	734159	734875		+	vreA	PA0674	2	4
	734872	735417		+	vreI	PA0675	2	4
	735487	736446		+	vreR	PA0676	4	8
	737097	736456		-	-	PA0677	1	2
	737530	737081		-	-	PA0678	0	0
	737677	738108		+	-	PA0679	0	3

	738485	738111		-	-	PA0680	2	2
	738719	739168		+	-	PA0681	1	4
	739176	740141		+	-	PA0682	0	3
	740203	741348		+	-	PA0683	1	0
	741335	741928		+	-	PA0684	1	1
	741925	744336		+	-	PA0685	4	2
	744333	745742		+	-	PA0686	2	2
	745742	746956		+	-	PA0687	3	5
747396	747470	748576	748630	+	-	PA0688	18	39
748647	748662	749774	749802	+	-	PA0689	40	50
	749957	762499		+	-	PA0690	7	7
	762891	763493		+	-	PA0691	1	1
	763657	765291		+	-	PA0692	1	1
	765315	767162		+	exbB2	PA0693	1	2
	767173	767592		+	exbD2	PA0694	3	1
	767601	768347		+	-	PA0695	0	0
	768415	770121		+	-	PA0696	4	3
	770156	770818		+	-	PA0697	1	5
	770847	771326		+	-	PA0698	5	3
	771331	772275		+	-	PA0699	4	3
	772275	772700		+	-	PA0700	2	4
	772710	773696		+	-	PA0701	4	5
	775321	774416		-	-	PA0702	8	8
	776716	775400		-	-	PA0703	9	11
	778181	776787		-	-	PA0704	15	16
779358	779208	778309	778264	-	migA	PA0705	143	162
780112	780101	779463	779370	-	cat	PA0706	78	83
	780973	780194		-	toxR	PA0707	7	7
	782113	781259		-	-	PA0708	28	29
	782229	782525		+	-	PA0709	14	17
	782570	782965		+	gloA2	PA0710	11	9
	783515	783009		-	-	PA0711	5	3

	783833	783576		-	-	PA0712	21	24
	784173	784466		+	-	PA0713	47	6
	784698	785174		+	-	PA0714	5	4
785498			785570	+	phrD	PA0714.1	37	37
785880	785969	786925	786925	+	-	PA0715	166	194
786926	786928	788253	788797	+	-	PA0716	106	143
	789144	789356		+	-	PA0717	10	13
	789360	789650		+	-	PA0718	18	12
	789654	790031		+	-	PA0719	11	9
	790166	790600	790600	+	-	PA0720	46	31
790601	790617	790709	790721	+	-	PA0721	32	29
790722	790722	790973	790973	+	-	PA0722	87	74
790974	790986	791234	791330	+	coaB	PA0723	99	113
	791370	792632		+	-	PA0724	7	3
	792637	792993		+	-	PA0725	7	3
	792997	794271		+	-	PA0726	9	7
	794501	795793		+	-	PA0727	13	10
	795793	796776		+	-	PA0728	11	11
796869	797251	797598	797682	+	-	PA0729	335	363
797794			797721	-	-	PA0729.1	48	80
798831	798827	797925	797917	-	-	PA0730	64	68
800021	800016	799279	799261	-	-	PA0731	46	68
	801139	800096		-	-	PA0732	31	22
	801967	801275		-	-	PA0733	21	17
802239	802239	801967		-	-	PA0734	37	35
	803075	802260	802240	-	-	PA0735	33	33
804231	804148	803072		-	-	PA0736	39	38
	804656	805111		+	-	PA0737	3	6
	805228	805473		+	-	PA0738	8	6
	806702	805788		-	-	PA0739	15	12
	806805	808781		+	sdsA1	PA0740	5	3
809457	809457	808816		-	-	PA0741	14	6

	809882	809574		-	-	PA0742	21	2
	811171	810275		-	-	PA0743	14	22
812386	812386	811283	811241	-	-	PA0744	16	58
813314	813263	812445	812443	-	-	PA0745	29	90
814492	814492	813329	813316	-	-	PA0746	15	47
816036	816012	814504	814493	-	-	PA0747	26	36
816901	817082	817903	817946	+	-	PA0749	26	39
817960	818003	818698	818716	+	ung	PA0750	155	182
	819862	818825		-	-	PA0751	31	36
	821372	819855		-	-	PA0752	14	17
	821853	821383		-	-	PA0753	7	12
	822885	821902		-	-	PA0754	23	24
	824198	822915		-	opdH	PA0755	17	12
824396	824409	825080		+	-	PA0756	37	36
	825073	826455	826455	+	-	PA0757	34	31
827337	827299	826463	826456	-	-	PA0758	172	113
828348	828344	827400	827338	-	-	PA0759	92	89
828612	828617	828871	828873	+	-	PA0760	211	305
830897	830892	829276	829090	-	nadB	PA0761	122	136
830973	831301	831882	831913	+	algU	PA0762	470	602
831914	831914	832498	832506	+	mucA	PA0763	361	475
832507	832507	833457		+	mucB	PA0764	179	252
	833454	833909	833909	+	mucC	PA0765	173	286
833910	833949	835373	835383	+	mucD	PA0766	458	754
835493	835523	837322	837327	+	lepA	PA0767	347	425
837328	837328	838182	838239	+	lepB	PA0768	203	245
838257	838351	838728		+	-	PA0769	230	254
	838725	839414		+	rnc	PA0770	221	227
	839407	840324	840324	+	era	PA0771	269	284
840325	840385	841086		+	recO	PA0772	49	53
	841079	841825	841873	+	pdxJ	PA0773	110	142
	842881	841913	841892	-	-	PA0774	105	147

843633	843618	842878		-	-	PA0775	140	233
	844295	843723		-	-	PA0776	3	3
	845179	844427		-	-	PA0777	21	22
845728	845682	845278	845214	-	icp	PA0778	111	124
848193	848192	845793	845793	-	-	PA0779	232	282
848356	848383	849135	849255	+	pruR	PA0780	30	31
	851319	849256		-	-	PA0781	5	4
851629	851783	854965	855101	+	putA	PA0782	517	53
855103	855277	856797	856802	+	putP	PA0783	365	210
857943	857871	856942	856863	-	-	PA0784	68	44
	857998	858636		+	azoR1	PA0785	10	10
	858646	858951		+	-	PA0786	9	6
	859007	860170		+	-	PA0787	24	28
	863300	860175		-	-	PA0788	8	7
863891	864095	865510	865525	+	-	PA0789	522	677
	866451	865636		-	-	PA0790	28	33
	866558	867346		+	-	PA0791	29	39
868808	868808	867324		-	prpD	PA0792	16	29
870248	870130	868943	868809	-	-	PA0793	26	55
872985	872855	870249	870249	-	-	PA0794	46	86
874137	874113	872986	872986	-	prpC	PA0795	160	317
875102	875102	874206	874139	-	prpB	PA0796	103	161
875857	875835	875113	875110	-	-	PA0797	31	52
	875953	876603		+	pmtA	PA0798	10	11
878963	878608	876617	876604	-	-	PA0799	59	46
879027	879027	879560		+	-	PA0800	14	33
	879557	881077		+	-	PA0801	31	41
	881077	881400	881485	+	-	PA0802	38	52
	881486	881926		+	-	PA0803	14	19
	881958	882779		+	-	PA0804	25	23
883581	883216	882989	882974	-	-	PA0805	721	495
	883777	884172		+	-	PA0806	7	6

884338	884799	885566	885634	+	ampDh3	PA0807	52	36
	885635	886108		+	-	PA0808	17	19
	887476	886160		-	-	PA0809	15	10
	888284	887583		-	-	PA0810	14	12
	889562	888315		-	-	PA0811	13	9
	890854	889559		-	-	PA0812	12	7
	892389	891142		-	-	PA0813	12	11
	892820	892386		-	-	PA0814	6	2
892864	893041	893994		+	-	PA0815	45	22
	894851	893967		-	-	PA0816	27	30
	894952	895377		+	-	PA0817	19	13
	895668	895396		-	-	PA0818	20	10
	895824	896117		+	-	PA0819	12	8
896401	896416	897228	897250	+	-	PA0820	122	100
898439	898423	897335	897251	-	-	PA0821	47	54
898886	898886	898440	898440	-	-	PA0822	58	63
	899141	898908	898887	-	-	PA0823	50	63
	899656	899138		-	-	PA0824	16	17
	900165	899830		-	-	PA0825	9	6
	901046	900408		-	-	PA0826	23	22
901872			901520	-	ssrA	PA0826.2	131612	173918
901881	901779	901746		-	-	PA0826.1	151826	200084
	902812	901934		-	-	PA0827	22	15
	902900	903583		+	-	PA0828	4	5
	903692	904633		+	-	PA0829	5	4
	905601	904768		-	-	PA0830	25	21
905701	905726	906745	906835	+	oruR	PA0831	70	66
906836	906847	907488	907555	+	-	PA0832	82	75
907561	907594	908307	908307	+	-	PA0833	367	575
909296	909291	908377	908363	-	-	PA0834	78	121
911532	911532	909418	909418	-	pta	PA0835	52	95
912790	912779	911595	911533	-	ackA	PA0836	111	115

912791			912861	+	-	PA0836.1	375	279
913071	913086	913571	913623	+	slyD	PA0837	581	618
913624	913777	914259	914262	+	-	PA0838	152	181
	914412	915002		+	-	PA0839	16	11
	915043	916155		+	-	PA0840	30	18
916307	916346	917305	917308	+	-	PA0841	24	39
	918529	917309		-	-	PA0842	7	13
	919240	918617		-	plcR	PA0843	2	3
	921450	919258		-	plcH	PA0844	1	1
	923804	921792		-	-	PA0845	2	3
924938	924922	924182	924020	-	-	PA0846	67	88
	927214	925007		-	-	PA0847	10	9
	927147	927746	927859	+	-	PA0848	21	39
927862	927880	928830	928865	+	trxB2	PA0849	31	58
	929084	929503	929503	+	-	PA0850	14	21
	930476	929514		-	-	PA0851	23	29
931844	931822	930653	930651	-	cbpD	PA0852	92	66
	932725	932102	932090	-	-	PA0853	42	43
934115	934112	932718		-	fumC2	PA0854	48	51
935274	935260	934241	934116	-	-	PA0855	54	52
935427	935427	935975	935975	+	-	PA0856	496	576
935976	935989	936294	936349	+	bolA	PA0857	311	409
936369	936459	937397	937397	+	-	PA0858	248	323
937433	937437	938039		+	-	PA0859	82	117
	938032	939822	939822	+	-	PA0860	41	53
	940117	942573		+	-	PA0861	31	24
942587	942648	943430	943440	+	-	PA0862	85	62
	944441	943482		-	-	PA0863	9	10
	945512	944724		-	-	PA0864	8	6
945788	945834	946907	946907	+	hpd	PA0865	397	2082
946908	947205	948623	948775	+	aroP2	PA0866	34	228
948776	948776	949159	949159	+	mliC	PA0867	307	361

949160	949280	949693	949715	+	-	PA0868	51	77
950700	950648	949716	949716	-	pbpG	PA0869	87	139
	952161	950962	950948	-	phhC	PA0870	333	1775
952519	952514	952158		-	phhB	PA0871	604	2701
952520			952600	?	-	predicted RN	294	1113
953428	953407	952619	952603	-	phhA	PA0872	546	1747
953429			953438	?	-	predicted RN	215	622
953638	953691	955250	955252	+	phhR	PA0873	52	37
955865	955722	955456	955450	-	-	PA0874	35	96
	958186	955991		-	-	PA0875	7	9
958187	958406	959350	959350	+	-	PA0876	57	85
960452	960380	959484	959351	-	-	PA0877	29	33
	960497	961324		+	-	PA0878	3	1
	961370	962530		+	-	PA0879	3	4
	962545	962925		+	-	PA0880	1	3
	962964	964316		+	-	PA0881	1	2
	964372	965574		+	-	PA0882	2	3
	965585	966412		+	-	PA0883	1	2
	966526	967521		+	-	PA0884	5	5
	967570	968208		+	-	PA0885	3	3
	968205	969488		+	-	PA0886	3	3
969646	969670	971625	971650	+	acsA	PA0887	450	46
971858			971972	+	-	PA0887.1	61	48
971973	972166	972945	972946	+	aotJ	PA0888	547	518
972954	973064	973753	973753	+	aotQ	PA0889	158	144
973761	973770	974468	974481	+	aotM	PA0890	172	151
974482	974482	975594	975594	+	-	PA0891	130	105
975595	975613	976377	976409	+	aotP	PA0892	249	186
976410	976410	977399	977419	+	argR	PA0893	128	103
	977743	977420		-	-	PA0894	35	24
977907	977910	979130	979131	+	aruC	PA0895	398	441
979132			979144	?	-	predicted RN	226	359

979145	979329	980345	980347	+	aruF	PA0896	152	201
980357	980357	981379	981379	+	aruG	PA0897	109	137
981380	981422	982888		+	aruD	PA0898	126	154
	982885	984231	984231	+	aruB	PA0899	161	215
984235	984245	984535	984535	+	-	PA0900	334	363
984536	984549	985547	985547	+	aruE	PA0901	137	189
985618	985692	986696	986794	+	-	PA0902	70	51
986804	986818	989442	989529	+	alaS	PA0903	343	418
989539			989580	?	-	predicted RN	396	500
989590	989590	990828	990992	+	lysC	PA0904	352	424
990993			991005	?	-	predicted RN	547	580
991006	991013	991198	991259	+	rsmA	PA0905	721	808
991260			991350	+	-	PA0905.1	573	1959
991468			991544	+	-	PA0905.2	844	2856
991656			991732	+	-	PA0905.3	258	834
992713	992543	991830	991733	-	-	PA0906	53	47
992714	992714	993244	993310	+	-	PA0907	54	39
993374	993409	993783		+	-	PA0908	38	45
	993776	994051	994089	+	-	PA0909	53	51
994133	994143	994646	994673	+	-	PA0910	99	107
994690	994699	995151	995171	+	-	PA0911	81	78
	995711	995172	995172	-	-	PA0912	26	22
995852	996038	997486	997486	+	mgtE	PA0913	122	108
	997928	997536	997487	-	-	PA0914	47	48
998397	998382	997921		-	-	PA0915	49	67
999795	999766	998444	998444	-	-	PA0916	162	193
999992	1000013	1001917	1001938	+	kup	PA0917	54	83
	1002520	1001972	1001972	-	-	PA0918	32	36
1003965	1003964	1002681	1002565	-	-	PA0919	41	59
1006614	1006611	1003966	1003966	-	-	PA0920	28	43
1006848	1006860	1007219	1007219	+	-	PA0921	169	208
1007578	1007548	1007234	1007220	-	-	PA0922	148	150

1007803			1007727	-	-	PA0922.1	4	13
1007839	1007950	1008999	1009005	+	dinB	PA0923	50	65
	1010934	1009006		-	-	PA0924	11	16
1011454	1011454	1011047	1010935	-	-	PA0925	70	86
1011947	1011947	1011522	1011455	-	-	PA0926	76	81
1012972	1012972	1011983	1011948	-	ldhA	PA0927	29	37
1015879	1015752	1012975	1012974	-	gacS	PA0928	39	45
1015938	1015938	1016657		+	-	PA0929	43	51
	1016657	1017994	1018229	+	-	PA0930	24	29
	1018230	1020458		+	pirA	PA0931	9	11
1020693	1020708	1021607	1021607	+	cysM	PA0932	224	329
1021608	1021616	1022968	1022968	+	ygcA	PA0933	157	214
1022971	1023053	1025296	1025296	+	relA	PA0934	216	284
1025363	1025401	1026231	1026273	+	-	PA0935	89	125
1027329	1027212	1026274	1026274	-	lpxO2	PA0936	145	216
1027375	1027445	1027984	1028113	+	-	PA0937	245	358
1028116	1028172	1029500	1029565	+	-	PA0938	379	734
	1030151	1029825		-	-	PA0939	18	30
	1030411	1030157		-	-	PA0940	25	16
	1030656	1030423		-	-	PA0941	42	17
1030769	1030772	1031332	1031385	+	-	PA0942	52	44
	1032102	1031386	1031386	-	-	PA0943	445	573
	1032763	1032095		-	purN	PA0944	275	393
1033883	1033824	1032763		-	purM	PA0945	346	535
1033990	1034038	1035075	1035086	+	-	PA0946	117	108
1035265	1035277	1035981	1035981	+	-	PA0947	265	470
1036577	1036577	1036158	1035982	-	-	PA0948	105	167
	1037175	1036579	1036578	-	wrbA	PA0949	174	251
1037525	1037525	1037172		-	-	PA0950	207	349
1037578	1037650	1038885	1039009	+	-	PA0951	48	59
1039623	1039623	1039240	1039014	-	-	PA0952	199	153
1039941	1039968	1040432		+	-	PA0953	71	70

	1040432	1040707	1040723	+	-	PA0954	71	82
1041680	1041680	1040724	1040724	-	-	PA0955	202	283
1043410	1043404	1041689	1041681	-	proS	PA0956	423	569
1043418	1043512	1043919	1043977	+	-	PA0957	44	43
1045381	1045314	1043983	1043978	-	oprD	PA0958	1558	1259
1045816	1045832	1046461		+	-	PA0959	84	138
	1046462	1046671	1046671	+	-	PA0960	88	133
1047431	1046911	1046720	1046713	-	-	PA0961	120	185
1048054	1048019	1047549	1047471	-	-	PA0962	496	558
1048288	1048459	1050234	1050300	+	aspS	PA0963	649	924
1050301	1050301	1051047	1051058	+	pmpR	PA0964	1046	1329
1051059	1051132	1051656	1051672	+	ruvC	PA0965	224	319
1051673	1051673	1052278	1052288	+	ruvA	PA0966	263	339
1052289	1052289	1053347	1053399	+	ruvB	PA0967	155	213
1053400	1053400	1053846	1053847	+	-	PA0968	308	629
1053848	1053848	1054543	1054565	+	tolQ	PA0969	557	739
1054566	1054566	1055006	1055008	+	tolR	PA0970	534	673
1055009	1055009	1056052		+	tolA	PA0971	332	504
	1056049	1057347	1057347	+	tolB	PA0972	622	825
1057348	1057400	1057906	1057915	+	oprL	PA0973	4762	5768
1057916	1057916	1058740	1058740	+	-	PA0974	789	1200
1058785	1058812	1059606	1059606	+	-	PA0975	69	145
1059621	1059622	1060296	1060355	+	-	PA0976	105	133
1060356			1060431	+	-	PA0976.1	316	837
1061008	1060833	1060510	1060432	-	-	PA0977	64	148
	1062034	1061207		-	-	PA0978	14	13
	1062369	1062061		-	-	PA0979	18	20
	1062601	1062885		+	-	PA0980	13	10
	1062921	1063544		+	-	PA0981	29	21
	1065103	1064555		-	-	PA0982	32	18
1065104	1065138	1065425	1065781	+	-	PA0983	29	31
1065943	1065970	1066296	1066320	+	-	PA0984	44	61

1067823	1067817	1066321	1066321	-	pyoS5	PA0985	45	29
	1068193	1068456		+	-	PA0986	5	4
	1068489	1069331		+	-	PA0987	4	6
1069765	1069769	1070173	1070173	+	-	PA0988	73	59
1071238	1070854	1070294	1070174	-	-	PA0989	26	30
	1071877	1071239		-	-	PA0990	10	10
	1072462	1072839		+	-	PA0991	29	30
1072840	1073285	1073902	1073919	+	cupC1	PA0992	51	48
	1073960	1074673		+	cupC2	PA0993	3	1
	1074784	1077303		+	cupC3	PA0994	10	10
1077916	1077904	1077383	1077304	-	ogt	PA0995	50	24
	1078462	1080015		+	pqsA	PA0996	39	2
	1080009	1080860		+	pqsB	PA0997	44	2
	1080853	1081899		+	pqsC	PA0998	31	3
	1081942	1082955		+	pqsD	PA0999	46	10
	1082949	1083854	1083854	+	pqsE	PA1000	33	17
	1083972	1085564		+	phnA	PA1001	32	16
	1085542	1086144		+	phnB	PA1002	84	74
1087552	1087095	1086097		-	mvfR	PA1003	145	148
1087716	1087843	1088901	1088901	+	nadA	PA1004	192	284
1090555	1090443	1089010	1088902	-	-	PA1005	95	92
1090603	1090606	1090857	1090857	+	-	PA1006	98	160
1090858	1090891	1091964	1092024	+	-	PA1007	59	110
1092509	1092498	1092025	1092025	-	bcp	PA1008	394	468
1093092	1093067	1092510	1092510	-	-	PA1009	272	371
1093237	1093251	1094129	1094146	+	dapA	PA1010	607	808
1094147	1094147	1095337		+	-	PA1011	509	685
	1095276	1096034	1096045	+	-	PA1012	210	303
1096046			1096061	?	-	predicted RN	399	557
1096062	1096063	1096773	1096866	+	purC	PA1013	615	709
1096867			1096956	+	-	PA1013.1	178	319
1097296	1097296	1098288	1098315	+	-	PA1014	151	225

1099668	1099128	1098328	1098316	-	-	PA1015	75	79
	1099669	1100820		+	-	PA1016	21	22
	1100930	1103077		+	pauA	PA1017	6	8
	1103061	1103927		+	-	PA1018	7	7
	1104009	1105364		+	mucK	PA1019	4	4
	1105888	1107000		+	-	PA1020	12	13
	1107000	1107761		+	-	PA1021	10	11
	1107763	1108908		+	-	PA1022	10	12
	1108949	1109866		+	-	PA1023	16	16
	1109882	1110868		+	-	PA1024	12	12
	1110947	1112197		+	-	PA1025	3	8
1113064	1113050	1112574	1112235	-	-	PA1026	38	53
	1113129	1114718		+	-	PA1027	12	9
	1114774	1116060		+	-	PA1028	8	8
1116127	1116213	1116500	1116631	+	-	PA1029	105	89
1116632	1116635	1117390	1117531	+	-	PA1030	60	69
1117532			1117609	+	-	PA1030.1	15	32
1117999	1118158	1119519	1119673	+	-	PA1031	110	156
1122224	1122217	1119674	1119674	-	quiP	PA1032	72	101
1123002	1122981	1122340	1122318	-	-	PA1033	51	43
1123348	1123348	1123148	1123148	-	-	PA1034	288	416
1123851	1123850	1123356	1123350	-	-	PA1035	160	123
1124031	1124165	1124725	1124725	+	-	PA1036	57	31
1125518	1125465	1124845	1124726	-	-	PA1037	38	52
	1125865	1125548	1125520	-	-	PA1038	36	40
1126338	1126338	1125865		-	-	PA1039	40	47
1126848	1126838	1126341	1126339	-	-	PA1040	60	57
	1126969	1127601		+	-	PA1041	15	12
1127708	1127717	1128016		+	-	PA1042	76	92
	1128009	1128818	1128856	+	-	PA1043	53	65
1129357	1129321	1128857	1128857	-	-	PA1044	44	49
1129451	1129451	1131595	1131673	+	-	PA1045	67	82

1131674	1131674	1133947	1134005	+	-	PA1046	27	35
1134008	1134024	1135202	1135306	+	-	PA1047	66	72
1135318	1135408	1136289	1136351	+	-	PA1048	256	252
1136384	1136388	1137035	1137042	+	pdxH	PA1049	150	213
1137043	1137043	1138155	1138241	+	-	PA1050	42	84
	1138314	1139672	1139672	+	-	PA1051	88	16
	1139714	1140859		+	-	PA1052	54	28
1141265	1141268	1141732	1141732	+	-	PA1053	1278	1386
1141733	1142061	1144862		+	-	PA1054	41	52
	1144862	1145200		+	-	PA1055	53	53
	1145197	1146696		+	-	PA1056	41	47
	1146693	1147217		+	-	PA1057	72	90
	1147202	1147471		+	-	PA1058	44	60
	1147468	1147815	1147823	+	-	PA1059	55	59
1147824	1147928	1148833	1148837	+	-	PA1060	25	37
1148838	1148844	1149941	1149941	+	-	PA1061	26	50
1149947	1149952	1150473		+	-	PA1062	40	77
	1150470	1150721	1150749	+	-	PA1063	81	103
1151422	1151415	1150750	1150750	-	-	PA1064	168	159
	1151435	1151908		+	-	PA1065	11	15
	1152575	1151919	1151919	-	-	PA1066	11	15
	1152624	1153538		+	-	PA1067	14	8
1153590	1153637	1155547	1155547	+	-	PA1068	94	90
1155578	1155584	1157878	1157882	+	-	PA1069	110	107
1158655	1158655	1157954	1157930	-	braG	PA1070	83	159
	1159425	1158658	1158656	-	braF	PA1071	89	185
	1160675	1159422		-	braE	PA1072	90	186
1161843	1161595	1160672		-	braD	PA1073	104	217
1163065	1162977	1161856	1161847	-	braC	PA1074	596	1060
1163276	1163276	1163593	1163648	+	-	PA1075	105	135
1163657	1163660	1164022	1164042	+	-	PA1076	284	300
1164256	1164275	1164682	1164682	+	flgB	PA1077	164	203

1164684	1164688	1165128	1165128	+	flgC	PA1078	147	194
1165129	1165141	1165854	1165856	+	flgD	PA1079	196	198
1165881	1165882	1167270	1167270	+	flgE	PA1080	275	258
1167466	1167488	1168237	1168240	+	flgF	PA1081	85	120
1168256	1168284	1169069	1169099	+	flgG	PA1082	194	187
1169115	1169115	1169810	1169821	+	flgH	PA1083	109	122
1169822	1169822	1170931	1170931	+	flgI	PA1084	66	78
1170932	1170942	1172144	1172162	+	flgJ	PA1085	91	93
1172163	1172163	1174214	1174239	+	flgK	PA1086	119	110
1174240	1174240	1175559	1175613	+	flgL	PA1087	144	157
1175614	1175614	1176375	1176379	+	-	PA1088	109	133
1176380	1176380	1176982		+	-	PA1089	89	112
	1176958	1177620		+	-	PA1090	51	71
	1177613	1182697	1182704	+	fgtA	PA1091	69	96
1182825	1183058	1184524	1184524	+	fliC	PA1092	2379	2928
1184525	1184603	1184974	1184974	+	-	PA1093	707	953
1184975	1185060	1186484	1186605	+	fliD	PA1094	538	704
1186606	1186606	1186986	1187008	+	-	PA1095	655	783
1187009	1187009	1187305	1187377	+	-	PA1096	596	715
1187513	1187587	1189059	1189059	+	fleQ	PA1097	349	309
1189060	1189172	1190380	1190380	+	fleS	PA1098	113	123
1190381	1190385	1191806	1191824	+	fleR	PA1099	112	87
1192047	1192053	1192382	1192392	+	fliE	PA1100	194	214
1192405	1192405	1194201	1194201	+	fliF	PA1101	122	118
1194202	1194207	1195223	1195224	+	fliG	PA1102	219	224
1195225	1195225	1196031		+	-	PA1103	154	182
	1196021	1197376	1197382	+	fliI	PA1104	88	103
1197383	1197390	1197833	1197835	+	fliJ	PA1105	70	56
1198674	1198551	1197838	1197836	-	-	PA1106	42	41
	1199946	1198750		-	-	PA1107	7	5
	1201220	1200063		-	-	PA1108	3	3
	1201310	1202089		+	-	PA1109	21	21

1202810	1202804	1202094	1202090	-	-	PA1110	51	55
	1202917	1203540		+	-	PA1111	2	1
	1203633	1204781		+	-	PA1112	12	10
1205330			1205031	-	-	PA1112.1	199	142
	1205771	1207537		+	-	PA1113	10	12
	1207875	1207540		-	-	PA1114	19	20
	1210303	1207979		-	-	PA1115	33	26
1210554	1210558	1211397	1211406	+	-	PA1116	77	129
1211808	1211781	1211407	1211407	-	-	PA1117	73	105
	1212571	1211888		-	-	PA1118	31	26
	1213223	1212717	1212594	-	yfiB	PA1119	58	97
	1214502	1213195		-	tpbB	PA1120	21	28
1215120	1215074	1214502		-	yfiR	PA1121	31	41
1215271	1215284	1215727	1215727	+	-	PA1122	50	57
1216637	1216440	1216120	1215728	-	-	PA1123	36	70
1216671	1216671	1218167		+	dgt	PA1124	27	26
1218168	1218181	1218933	1218939	+	-	PA1125	24	31
1219527	1219527	1218940	1218940	-	-	PA1126	72	95
1220654	1220610	1219621	1219528	-	-	PA1127	110	81
	1220745	1221656		+	-	PA1128	21	18
	1221691	1222098		+	-	PA1129	16	20
	1223072	1222095		-	rhlC	PA1130	10	10
	1224328	1223060		-	-	PA1131	3	1
1224491	1224641	1225330	1225330	+	-	PA1132	49	85
1225756	1225690	1225340	1225331	-	-	PA1133	18	21
	1226278	1225757		-	-	PA1134	6	6
	1227302	1226427		-	-	PA1135	15	14
	1227474	1228205		+	-	PA1136	12	7
	1229198	1228212		-	-	PA1137	5	8
	1229344	1230219		+	-	PA1138	15	9
	1230310	1231158		+	-	PA1139	28	26
1232146	1232092	1231256	1231243	-	-	PA1140	61	116

	1233131	1232229		-	-	PA1141	21	19
1234032	1234013	1233297	1233132	-	-	PA1142	38	37
	1234748	1234095		-	-	PA1143	3	3
	1236150	1234816		-	-	PA1144	2	2
	1236644	1237546		+	-	PA1145	9	12
	1238732	1237551		-	-	PA1146	5	5
	1240324	1238834		-	-	PA1147	10	16
	1242500	1240584		-	toxA	PA1148	5	3
	1243118	1242750		-	-	PA1149	5	5
1243591	1243594	1245663	1245663	+	pys2	PA1150	107	92
1245664	1245665	1245928	1245928	+	imm2	PA1151	760	680
1245929	1246444	1246788	1247931	+	-	PA1152	44	64
	1248486	1247932		-	-	PA1153	12	13
	1249552	1249019		-	-	PA1154	10	13
1251154	1251154	1249907	1249840	-	nrdB	PA1155	705	740
1254698	1254309	1251418	1251155	-	nrdA	PA1156	651	537
1255006	1255042	1255752	1255752	+	-	PA1157	68	107
1255753	1256094	1257452	1257593	+	-	PA1158	24	39
1257636	1257772	1257981	1257986	+	-	PA1159	1800	2550
1258480	1258470	1258087	1257988	-	-	PA1160	113	153
	1259291	1258488	1258488	-	rrmA	PA1161	65	83
1260457	1260442	1259291		-	dapE	PA1162	98	120
	1263166	1260557		-	ndvB	PA1163	13	23
1263357	1263378	1264190		+	-	PA1164	80	108
1265008	1264919	1264191		-	pcpS	PA1165	46	61
	1265935	1265147		-	-	PA1166	4	2
	1266111	1266782		+	-	PA1167	14	12
	1267282	1267602		+	-	PA1168	3	6
	1267680	1269737		+	-	PA1169	4	7
1270705	1270515	1269781	1269738	-	-	PA1170	33	75
1270874	1270972	1272168	1272199	+	-	PA1171	66	89
	1272796	1272200		-	napC	PA1172	25	30

	1273298	1272807		-	napB	PA1173	8	10
	1275798	1273309		-	napA	PA1174	5	6
	1276124	1275795		-	napD	PA1175	5	9
	1276608	1276117		-	napF	PA1176	6	4
	1276784	1276617	1276617	-	napE	PA1177	14	12
1276975	1277006	1277608	1277608	+	oprH	PA1178	1970	2100
1277610	1277688	1278365		+	phoP	PA1179	416	437
	1278362	1279708	1279809	+	phoQ	PA1180	99	121
1279810	1279810	1283172	1283175	+	-	PA1181	18	41
1284184	1284177	1283176	1283176	-	-	PA1182	16	28
	1284513	1285823		+	dctA	PA1183	4	5
	1286788	1285898		-	-	PA1184	18	15
	1286990	1287619		+	-	PA1185	4	2
	1287616	1288635		+	-	PA1186	3	2
	1288737	1289876		+	-	PA1187	6	5
	1289965	1291146		+	-	PA1188	9	10
	1291645	1291148		-	-	PA1189	14	21
	1292412	1291813		-	-	PA1190	11	11
	1293164	1292571		-	-	PA1191	13	7
1293250	1293267	1294091	1294137	+	-	PA1192	196	297
1294138	1294138	1294809	1294810	+	-	PA1193	104	146
	1296229	1294811		-	-	PA1194	12	17
	1297098	1296334		-	-	PA1195	8	5
	1297309	1298709		+	-	PA1196	47	36
1298715	1298722	1299492	1299556	+	-	PA1197	35	50
1299558	1299558	1300175	1300175	+	-	PA1198	413	564
1300216	1300258	1300791	1300807	+	-	PA1199	204	129
1300811	1300811	1301473	1301480	+	-	PA1200	70	46
	1302395	1301481		-	-	PA1201	31	29
1302621	1302696	1303313	1303344	+	-	PA1202	41	28
	1303457	1303864		+	-	PA1203	37	23
1303865	1303892	1304449	1304452	+	-	PA1204	46	30

1304476	1304476	1305423		+	-	PA1205	29	22
1305424	1305583	1306056	1306058	+	-	PA1206	75	90
1306059	1306059	1307900	1308066	+	kefB	PA1207	71	80
	1308067	1309566		+	-	PA1208	14	15
	1310518	1309583		-	-	PA1209	14	10
	1311386	1310688		-	-	PA1210	7	9
	1312139	1311504		-	-	PA1211	2	2
	1313365	1312136		-	-	PA1212	1	2
	1314321	1313362		-	-	PA1213	2	1
	1315916	1314318		-	-	PA1214	3	2
	1317202	1315916		-	-	PA1215	2	2
	1318150	1317404		-	-	PA1216	9	6
	1319514	1318147		-	-	PA1217	8	9
	1320389	1319511		-	-	PA1218	4	5
	1321000	1320386		-	-	PA1219	2	3
	1322256	1320994		-	-	PA1220	3	1
	1324109	1322253		-	-	PA1221	2	1
1326045	1325950	1324793	1324512	-	-	PA1222	46	65
	1326939	1326046		-	-	PA1223	19	15
	1327024	1327803		+	-	PA1224	32	27
	1328439	1327813	1327813	-	-	PA1225	29	23
	1328545	1329153		+	-	PA1226	27	26
1329155	1329192	1330040	1330051	+	-	PA1227	92	69
1330467	1330467	1330117	1330108	-	-	PA1228	263	216
	1331383	1330577		-	-	PA1229	16	12
	1331517	1331714		+	-	PA1230	1	3
	1331727	1332635		+	-	PA1231	2	2
	1332635	1334674		+	-	PA1232	2	3
	1334671	1334928		+	-	PA1233	18	21
1335535	1335453	1334950	1334929	-	-	PA1234	27	29
	1335645	1336427		+	-	PA1235	21	15
	1337941	1336430		-	-	PA1236	3	3

	1339082	1337931		-	-	PA1237	3	2
	1340527	1339079		-	-	PA1238	1	1
	1341794	1340910		-	-	PA1239	3	3
	1342648	1341851		-	-	PA1240	8	3
1342802	1342813	1343373	1343452	+	-	PA1241	36	36
	1343453	1345225		+	-	PA1242	9	7
1348059	1347824	1345248	1345226	-	-	PA1243	31	45
1348626	1348401	1348060	1348060	-	-	PA1244	354	508
1349235			1349415	?	-	predicted RN	159	64
	1349416	1350660		+	-	PA1245	40	17
	1350747	1352528		+	aprD	PA1246	9	4
	1352525	1353823	1353823	+	aprE	PA1247	10	4
	1353827	1355272		+	aprF	PA1248	8	3
1355607			1355630	?	-	predicted RN	23	13
	1355631	1357070	1357070	+	aprA	PA1249	15	10
1357198	1357317	1357712	1357724	+	aprI	PA1250	92	118
	1359350	1357725	1357725	-	-	PA1251	20	24
	1359590	1360594		+	-	PA1252	6	5
	1362278	1360698		-	-	PA1253	2	2
	1363288	1362371		-	-	PA1254	1	2
	1364473	1363439		-	-	PA1255	2	0
	1365221	1364499		-	-	PA1256	3	4
	1365862	1365218		-	-	PA1257	4	6
	1366538	1365873		-	-	PA1258	2	2
	1368280	1366538		-	-	PA1259	4	3
	1369160	1368339		-	-	PA1260	2	2
	1370092	1369418		-	-	PA1261	7	7
	1371699	1370257		-	-	PA1262	2	2
1371738	1371738	1372682	1372969	+	-	PA1263	44	50
	1373845	1372979		-	-	PA1264	7	10
	1373945	1374847		+	-	PA1265	2	2
	1376097	1374844		-	-	PA1266	2	1

	1377442	1376327		-	-	PA1267	2	2
	1378383	1377439		-	-	PA1268	2	2
1379200	1379168	1378500	1378437	-	-	PA1269	40	42
	1379413	1381452		+	-	PA1270	5	6
1381525	1381804	1383654	1383717	+	-	PA1271	89	149
1383718	1383718	1384329	1384360	+	cobO	PA1272	127	193
1384361	1384361	1385668	1385668	+	cobB	PA1273	60	82
1385669	1385670	1386326		+	-	PA1274	54	88
	1386323	1387261		+	cobD	PA1275	43	64
	1387254	1388249		+	cobC	PA1276	55	87
	1388242	1389714		+	cobQ	PA1277	62	94
	1389707	1390228		+	cobP	PA1278	73	119
	1390225	1391280		+	cobU	PA1279	64	91
	1391277	1391861	1391863	+	-	PA1280	47	74
1391864	1391864	1392601		+	cobV	PA1281	26	37
	1394069	1392564		-	-	PA1282	10	11
	1394188	1394748		+	-	PA1283	22	19
	1396599	1394779		-	-	PA1284	6	8
1396731	1396731	1397180	1397253	+	-	PA1285	59	70
	1397254	1398453		+	-	PA1286	9	10
1398610	1398618	1399172	1399230	+	-	PA1287	60	50
1400510	1400505	1399231	1399231	-	-	PA1288	1266	1153
	1401190	1400711		-	-	PA1289	16	9
	1402005	1401412		-	-	PA1290	15	18
	1402117	1402956		+	-	PA1291	27	25
1404125	1403835	1402981	1402957	-	-	PA1292	72	72
1405207	1405197	1404136	1404126	-	-	PA1293	112	98
1405334	1405338	1406462		+	rnd	PA1294	111	146
	1406459	1406752	1406760	+	-	PA1295	61	82
1406761	1407007	1407939	1407948	+	-	PA1296	31	42
1408948	1408931	1407951	1407949	-	-	PA1297	25	32
1408999	1408999	1409274		+	-	PA1298	27	24

1409348	1409351	1409800	1409805	+	-	PA1299	92	165
1409806	1409949	1410476		+	-	PA1300	16	36
	1410473	1411456		+	-	PA1301	14	17
	1411585	1414140		+	-	PA1302	9	8
1414699	1414686	1414147	1414141	-	-	PA1303	29	38
1416901	1416814	1414763	1414762	-	-	PA1304	48	65
1416963	1416963	1417436	1417436	+	-	PA1305	106	164
1417437	1417500	1417961	1417961	+	-	PA1306	67	85
1417963	1417965	1418738	1418744	+	-	PA1307	88	97
1418765	1418765	1419289	1419297	+	-	PA1308	65	85
	1420158	1419298		-	-	PA1309	31	24
	1420326	1421441		+	phnW	PA1310	4	5
	1421477	1422304		+	phnX	PA1311	7	9
	1423266	1422355		-	-	PA1312	13	11
	1423374	1424732		+	-	PA1313	2	3
	1424746	1425132		+	-	PA1314	15	16
	1425754	1425140		-	-	PA1315	29	20
	1425912	1427453		+	-	PA1316	12	12
	1428080	1429075	1429075	+	cyoA	PA1317	82	10
	1429082	1431058		+	cyoB	PA1318	70	8
1431062	1431062	1431691		+	cyoC	PA1319	58	5
	1431691	1432026		+	cyoD	PA1320	53	9
1432037	1432037	1432927		+	cyoE	PA1321	30	5
	1433166	1435364		+	-	PA1322	3	3
	1435493	1435825	1435825	+	-	PA1323	22	22
	1435885	1436397		+	-	PA1324	12	11
1436491			1436618	+	-	PA1324.1	31	24
	1436663	1437070		+	-	PA1325	31	18
	1437067	1438614		+	ilvA2	PA1326	14	7
	1438706	1440676		+	-	PA1327	7	6
	1441547	1440639		-	-	PA1328	21	10
	1441632	1442063		+	-	PA1329	2	0

	1442069	1442848	1442848	+	-	PA1330	22	19
1444650	1444451	1442904	1442849	-	-	PA1331	28	39
	1445271	1444720	1444720	-	-	PA1332	22	13
	1445611	1445405		-	-	PA1333	76	19
	1446918	1445806		-	-	PA1334	6	4
	1448503	1447226		-	-	PA1335	19	27
	1450449	1448548		-	-	PA1336	24	29
1451633	1451633	1450545	1450533	-	ansB	PA1337	94	240
1453469	1453392	1451719	1451662	-	ggt	PA1338	83	114
	1454204	1453470	1453470	-	-	PA1339	234	241
	1454869	1454201		-	-	PA1340	178	182
1455671	1455612	1454866		-	-	PA1341	130	140
1456825	1456723	1455815	1455815	-	-	PA1342	421	444
1457123	1457175	1457633	1457686	+	-	PA1343	54	66
1457729	1457729	1458523	1458706	+	-	PA1344	38	40
	1458707	1460296		+	-	PA1345	4	3
	1461854	1460307		-	-	PA1346	1	1
	1462630	1461938		-	-	PA1347	5	6
	1462696	1463403		+	-	PA1348	6	6
	1463586	1463936		+	-	PA1349	4	1
	1464111	1464860		+	-	PA1350	7	5
	1464878	1466101		+	-	PA1351	1	2
	1467320	1466109		-	-	PA1352	2	2
	1467901	1467488		-	-	PA1353	5	4
	1468363	1467938		-	-	PA1354	7	7
	1468510	1468890		+	-	PA1355	1	2
	1468913	1470019		+	-	PA1356	2	1
1470086	1470088	1470564	1470579	+	-	PA1357	84	71
1470988	1470978	1470580	1470580	-	-	PA1358	69	70
	1471672	1471016		-	-	PA1359	27	39
	1471671	1472564		+	-	PA1360	39	44
1474052	1473980	1472547		-	-	PA1361	52	64

	1474391	1474714		+	-	PA1362	10	9
1474715	1474727	1475467		+	-	PA1363	15	29
	1475464	1476306		+	-	PA1364	13	17
	1476384	1478825		+	-	PA1365	16	20
1480214	1479791	1479021	1478953	-	-	PA1366	64	53
	1481051	1480215		-	-	PA1367	18	21
	1482758	1481334		-	-	PA1368	18	15
1483112	1483123	1483875	1483897	+	-	PA1369	112	94
1483898	1483898	1485763	1486095	+	-	PA1370	58	62
	1486967	1486266	1486266	-	-	PA1371	99	110
1489128	1489095	1486960		-	-	PA1372	156	165
	1491083	1489815		-	fabF2	PA1373	25	29
	1491192	1491698		+	-	PA1374	21	16
1491699	1491913	1493055	1493089	+	pdxB	PA1375	49	56
1494830	1494823	1493090	1493090	-	aceK	PA1376	36	31
1494956	1494959	1495492	1495592	+	-	PA1377	44	43
	1495635	1495997		+	-	PA1378	20	25
	1496920	1496087		-	-	PA1379	6	3
	1497012	1497977		+	-	PA1380	13	14
	1498338	1498829		+	-	PA1381	10	11
	1498813	1501092	1501109	+	-	PA1382	27	33
1501430	1501611	1503416	1503567	+	-	PA1383	40	40
	1503568	1504581		+	galE	PA1384	9	10
	1504605	1505729		+	-	PA1385	6	11
	1505749	1507017		+	-	PA1386	10	13
	1507164	1508720		+	-	PA1387	19	18
	1508737	1509465		+	-	PA1388	9	13
	1509562	1511046		+	-	PA1389	10	13
	1511232	1512266		+	-	PA1390	7	10
	1512260	1514035		+	-	PA1391	5	6
	1514036	1515280		+	-	PA1392	7	14
	1515927	1515337		-	cysC	PA1393	14	18

	1516433	1516687		+	-	PA1394	22	40
1516688	1516703	1517119	1517145	+	-	PA1395	37	43
	1518768	1517146	1517146	-	-	PA1396	24	25
	1518914	1519546		+	-	PA1397	29	39
1519547	1519627	1519968	1519983	+	-	PA1398	46	48
	1520904	1519984		-	-	PA1399	16	13
	1521109	1524396		+	-	PA1400	3	4
	1524831	1524424		-	-	PA1401	15	12
	1525574	1524909		-	-	PA1402	7	9
	1525667	1526299		+	-	PA1403	17	19
	1526657	1526430		-	-	PA1404	10	13
1526813	1527191	1528783	1528802	+	-	PA1405	32	32
	1529561	1528803		-	-	PA1406	16	16
	1530538	1529597		-	-	PA1407	23	26
	1533028	1530605		-	-	PA1408	4	4
	1533238	1534278		+	aphA	PA1409	4	8
	1534289	1535380		+	-	PA1410	5	9
	1535586	1536497		+	-	PA1411	20	23
	1537650	1536457		-	-	PA1412	3	5
	1537755	1538627		+	-	PA1413	17	9
1538683	1538683	1538916	1538916	+	-	PA1414	583	846
1539748	1539704	1538976	1538917	-	-	PA1415	33	42
	1541131	1539749		-	-	PA1416	23	13
	1542746	1541145		-	-	PA1417	21	5
	1544213	1542822		-	-	PA1418	36	8
	1545818	1544307		-	-	PA1419	19	5
1546256	1546256	1545837		-	-	PA1420	56	4
	1547230	1546271		-	gbuA	PA1421	126	10
1547387	1547437	1548330	1548333	+	gbuR	PA1422	34	51
	1549587	1548334		-	bdlA	PA1423	17	23
	1549883	1550494		+	-	PA1424	22	20
	1550984	1552600		+	-	PA1425	13	17

	1552641	1552997		+	-	PA1426	19	25
	1553112	1553675		+	-	PA1427	21	26
	1554415	1554846		+	-	PA1428	19	30
	1555012	1557720		+	-	PA1429	38	52
1558043	1558171	1558890		+	lasR	PA1430	332	317
1559136	1559122	1558880		-	rsaL	PA1431	650	503
1559248	1559254	1559859	1559859	+	lasI	PA1432	1721	1921
	1561918	1559966	1559860	-	-	PA1433	137	140
	1562632	1561919		-	-	PA1434	16	25
	1562813	1563970		+	-	PA1435	3	2
	1563967	1567077		+	-	PA1436	4	3
	1567181	1567870		+	-	PA1437	21	17
	1567848	1569293		+	-	PA1438	9	17
1569720	1569709	1569302	1569294	-	-	PA1439	61	67
1570323	1570308	1569721	1569721	-	-	PA1440	357	444
1570468	1570496	1571779	1571791	+	-	PA1441	170	203
1571939	1572023	1572544	1572544	+	-	PA1442	138	191
1572545	1572552	1573523	1573523	+	fliM	PA1443	160	186
1573524	1573551	1574024	1574025	+	fliN	PA1444	132	151
1574026	1574026	1574478		+	fliO	PA1445	90	119
	1574475	1575242	1575243	+	fliP	PA1446	89	111
1575288	1575290	1575559		+	fliQ	PA1447	78	93
	1575559	1576335	1576337	+	fliR	PA1448	29	40
	1576338	1577474		+	flhB	PA1449	15	20
	1577547	1578806		+	-	PA1450	17	23
	1578839	1580182		+	-	PA1451	19	28
1580291	1580321	1582444	1582527	+	flhA	PA1452	72	73
1582528	1582528	1583817	1583817	+	flhF	PA1453	170	150
1583818	1583956	1584798		+	fleN	PA1454	277	302
	1584795	1585538	1585593	+	fliA	PA1455	266	305
1585636	1585640	1586014	1586014	+	cheY	PA1456	353	422
1586015	1586034	1586822	1586822	+	cheZ	PA1457	349	407

1586823	1587023	1589284	1589284	+	-	PA1458	210	248
1589285	1589338	1590444	1590503	+	-	PA1459	143	175
1590533	1590533	1591273	1591285	+	motC	PA1460	86	131
1591286	1591286	1592176	1592188	+	motD	PA1461	75	107
1592271	1592271	1593059	1593150	+	-	PA1462	118	155
1593151	1593151	1594041	1594086	+	-	PA1463	118	150
1594087	1594087	1594566	1594596	+	-	PA1464	369	468
1594597	1594597	1595004	1595007	+	-	PA1465	143	192
1595826	1595718	1595032	1595008	-	-	PA1466	33	56
	1595827	1596798		+	-	PA1467	18	16
	1596889	1597305		+	-	PA1468	13	18
	1597365	1598087		+	-	PA1469	17	16
	1598221	1598958		+	-	PA1470	10	11
	1599320	1599024		-	-	PA1471	23	33
	1599982	1599428		-	-	PA1472	13	21
	1600418	1600083	1599983	-	-	PA1473	27	39
1601970	1601893	1600415		-	-	PA1474	44	70
1602083	1602179	1602880		+	ccmA	PA1475	69	149
	1602877	1603548	1603580	+	ccmB	PA1476	49	106
1603609	1603671	1604429		+	ccmC	PA1477	95	183
	1604426	1604602		+	-	PA1478	87	131
	1604599	1605087		+	ccmE	PA1479	147	208
	1605088	1607061	1607061	+	ccmF	PA1480	110	172
1607062	1607065	1607607		+	ccmG	PA1481	102	185
	1607604	1608071		+	ccmH	PA1482	112	174
	1608068	1609291	1609514	+	cycH	PA1483	90	154
	1610544	1609738	1609738	-	-	PA1484	21	25
	1610745	1612112		+	-	PA1485	1	2
	1612126	1613226		+	-	PA1486	6	6
1614649	1614649	1613234		-	-	PA1487	14	10
	1615908	1614670		-	-	PA1488	18	11
	1617085	1615895		-	-	PA1489	19	10

	1618265	1617489		-	-	PA1490	18	20
	1618612	1619847		+	-	PA1491	12	10
	1619907	1620263		+	-	PA1492	23	18
1621352	1621343	1620345	1620341	-	cysP	PA1493	226	155
	1623127	1621472		-	-	PA1494	25	25
	1623877	1623251		-	-	PA1495	8	12
	1624715	1623864		-	-	PA1496	16	21
	1625695	1624781		-	-	PA1497	5	5
	1627495	1626062		-	pykF	PA1498	5	4
	1628762	1627497		-	-	PA1499	2	3
	1629949	1629059		-	-	PA1500	6	4
	1630806	1630024		-	-	PA1501	17	10
	1632677	1630902		-	gcl	PA1502	5	3
	1633006	1633437		+	-	PA1503	6	4
1634514	1634492	1633842	1633732	-	-	PA1504	107	144
1634682	1634728	1635723	1635765	+	moaA2	PA1505	184	88
	1635908	1636237		+	-	PA1506	29	12
	1637696	1636329		-	-	PA1507	11	9
	1638639	1638379		-	-	PA1508	21	26
	1639794	1638652	1638640	-	-	PA1509	44	59
	1641500	1639791		-	-	PA1510	29	32
	1644025	1641497		-	-	PA1511	21	15
	1644725	1644207		-	hcpA	PA1512	7	4
1646536	1646488	1645211	1644917	-	-	PA1513	39	45
1647100	1647046	1646537	1646537	-	-	PA1514	36	44
	1648099	1647101		-	alc	PA1515	20	26
	1648632	1648117	1648100	-	-	PA1516	38	48
1649604	1649555	1648629		-	-	PA1517	36	44
	1649928	1650308		+	-	PA1518	30	25
	1651898	1650549		-	-	PA1519	3	5
1652206	1652273	1653052	1653052	+	-	PA1520	138	181
	1654410	1653106		-	-	PA1521	23	20

	1655319	1654480		-	-	PA1522	19	20
	1657745	1655346		-	xdhB	PA1523	11	8
	1659192	1657738		-	xdhA	PA1524	17	9
	1660546	1659413		-	alkB2	PA1525	7	8
1660702	1660727	1661386	1661388	+	-	PA1526	120	140
1661389	1661412	1664900	1664992	+	-	PA1527	99	147
1665026	1665065	1665934	1665934	+	zipA	PA1528	276	334
1665935	1666025	1668409	1668454	+	lig	PA1529	106	134
1668455	1668455	1668832	1668839	+	-	PA1530	116	168
1668840			1669799	+	ffs	PA1530.1	30	35
	1669952	1669086		-	-	PA1531	19	20
1669978	1669989	1672034	1672034	+	dnaX	PA1532	132	169
1672035	1672080	1672406	1672406	+	-	PA1533	686	758
1672407	1672485	1673081	1673087	+	recR	PA1534	129	159
	1673204	1674352	1674352	+	-	PA1535	33	33
	1674904	1674365		-	-	PA1536	27	25
	1675865	1674978		-	-	PA1537	7	9
	1677445	1675862		-	-	PA1538	7	5
	1677559	1678407		+	-	PA1539	21	25
	1678590	1678261		-	-	PA1540	13	16
	1678952	1678584		-	-	PA1541	9	7
1680436	1680197	1679361	1678953	-	-	PA1542	35	74
1681064	1680985	1680437	1680437	-	apt	PA1543	92	146
1681822	1681805	1681071	1681071	-	anr	PA1544	452	644
1682046	1682051	1682518	1682566	+	-	PA1545	136	179
1683963	1683949	1682567	1682567	-	hemN	PA1546	427	684
	1684760	1684077		-	-	PA1547	14	21
	1684962	1684753		-	-	PA1548	36	91
	1687376	1684941		-	-	PA1549	21	40
1687923	1687912	1687373		-	-	PA1550	86	162
1689399	1689339	1687924	1687924	-	-	PA1551	95	169
	1690513	1689557	1689534	-	ccoP1	PA1552	952	875

1690700	1690695	1690510		-	ccoQ1	PA1552.1	1471	1519
1691312	1691312	1690701	1690701	-	ccoO1	PA1553	1171	1088
1692785	1692754	1691327	1691313	-	ccoN1	PA1554	1134	1101
	1694045	1693119	1693115	-	ccoP2	PA1555	74	631
1694227	1694227	1694042		-	ccoQ2	PA1555.1	287	988
1694851	1694841	1694233	1694231	-	ccoO2	PA1556	91	650
1696359	1696279	1694852	1694852	-	ccoN2	PA1557	251	850
	1696467	1697099		+	-	PA1558	11	14
1697159	1697188	1697919		+	-	PA1559	236	306
	1697916	1698344	1698377	+	-	PA1560	205	265
1699974	1699947	1698382	1698382	-	aer	PA1561	135	219
	1703052	1700320		-	acnA	PA1562	31	33
1703123	1703379	1704437	1704519	+	-	PA1563	60	93
1704522	1704522	1704761	1704792	+	-	PA1564	89	99
1706103	1706103	1704793	1704793	-	-	PA1565	30	23
	1707536	1706172		-	-	PA1566	12	6
	1709401	1708004		-	-	PA1567	5	8
	1709765	1709412		-	-	PA1568	5	9
	1711191	1709821		-	-	PA1569	3	2
1711474	1711569	1712450	1712511	+	-	PA1570	40	32
1712703	1712698	1712519	1712516	-	-	PA1571	96	121
1712879	1712908	1714053	1714053	+	-	PA1572	44	38
1714057	1714064	1714660	1714771	+	-	PA1573	26	35
1715082	1715059	1714778	1714778	-	-	PA1574	182	207
	1715705	1715154		-	-	PA1575	11	10
1715805	1715809	1716675	1716676	+	-	PA1576	44	40
1717053	1717023	1716682	1716682	-	-	PA1577	54	81
	1717139	1717894		+	-	PA1578	10	10
1718312	1718386	1718994	1718999	+	-	PA1579	315	188
1720434	1720395	1719109	1719089	-	gltA	PA1580	1246	872
1720640	1720744	1721130		+	sdhC	PA1581	3303	4560
	1721124	1721492	1721495	+	sdhD	PA1582	2470	3054

1721496	1721496	1723268	1723268	+	sdhA	PA1583	1853	2062
1723269	1723280	1723987	1724010	+	sdhB	PA1584	2637	2866
1724011			1724243	?	-	predicted RN	2042	2657
1724244	1724244	1727075	1727117	+	sucA	PA1585	1461	1719
1727118	1727118	1728347	1728415	+	sucB	PA1586	1289	1576
1728416	1728416	1729852	1730114	+	lpdG	PA1587	1425	1795
1730118	1730181	1731347		+	sucC	PA1588	3954	5673
	1731347	1732234	1732234	+	sucD	PA1589	3444	4821
1732235			1732258	?	-	predicted RN	937	1195
1732347	1732545	1733858	1733877	+	braB	PA1590	71	102
1733970	1734004	1734768	1734826	+	-	PA1591	30	105
1735068	1735063	1734827	1734827	-	-	PA1592	635	945
1735069			1735078	?	-	predicted RN	357	514
1735227	1735236	1735709		+	-	PA1593	53	83
	1735706	1736152	1736179	+	-	PA1594	42	65
1736180	1736189	1737418	1737526	+	-	PA1595	25	43
1737534	1737536	1739440	1739452	+	htpG	PA1596	1033	1449
1739484	1739508	1740233	1740243	+	-	PA1597	81	110
1741077	1741065	1740244	1740244	-	-	PA1598	24	28
	1741146	1741928		+	-	PA1599	25	18
	1743240	1741939		-	-	PA1600	33	6
	1745536	1743290	1743290	-	-	PA1601	67	6
	1746024	1745533		-	-	PA1602	119	10
	1746653	1746231		-	-	PA1603	18	24
	1747597	1746692		-	-	PA1604	35	25
	1747596	1748630		+	-	PA1605	10	6
	1748699	1749175		+	-	PA1606	11	9
1749792	1749789	1749349	1749288	-	-	PA1607	125	135
1751673	1751520	1749895	1749795	-	-	PA1608	31	49
1752902	1752891	1751674	1751674	-	fabB	PA1609	856	1472
1753482	1753418	1752903	1752903	-	fabA	PA1610	747	1411
1753483			1753496	?	-	predicted RN	464	669

	1755562	1753607	1753497	-	-	PA1611	32	47
1756317	1756317	1755559		-	-	PA1612	24	33
1758686	1758597	1756489	1756318	-	-	PA1613	34	43
1758748	1758748	1759770	1759770	+	gpsA	PA1614	122	159
1759771	1759784	1760125		+	-	PA1615	143	166
	1760122	1760586	1760712	+	-	PA1616	94	108
1760713	1760713	1762380	1762380	+	-	PA1617	17	24
1762381	1762433	1762870	1762886	+	-	PA1618	53	48
	1762928	1763752		+	-	PA1619	14	18
	1763793	1764434		+	-	PA1620	3	1
1764521	1764536	1765348		+	-	PA1621	25	35
	1765345	1766205		+	-	PA1622	16	25
1766273	1766285	1766947	1766955	+	-	PA1623	90	74
1766956	1766956	1767762	1767927	+	-	PA1624	47	57
	1767928	1768995		+	-	PA1625	19	20
	1770321	1769047		-	-	PA1626	19	19
	1770984	1770337		-	-	PA1627	22	27
	1772651	1771122		-	-	PA1628	9	12
	1773440	1772655		-	-	PA1629	13	16
1773596	1773682	1774548	1774602	+	-	PA1630	32	34
	1774603	1775757		+	-	PA1631	16	12
1775921	1775945	1776034	1776044	+	kdpF	PA1632	18	128
1776045	1776045	1777739	1777750	+	kdpA	PA1633	15	102
1777751	1777751	1779823	1779823	+	kdpB	PA1634	7	77
1779824	1779877	1780428	1780527	+	kdpC	PA1635	7	56
1780528	1780536	1783193	1783193	+	kdpD	PA1636	12	45
1783194	1783294	1783986	1784070	+	kdpE	PA1637	28	45
1784104	1784108	1785016	1785093	+	-	PA1638	36	52
1785124	1785127	1785732	1785735	+	-	PA1639	93	91
1786786	1786773	1785736	1785736	-	-	PA1640	65	68
	1786888	1787166		+	-	PA1641	19	19
1787237	1787250	1788284		+	selD	PA1642	99	121

	1788284	1789393	1789856	+	-	PA1643	63	84
1789857	1789857	1790465	1790465	+	-	PA1644	80	93
1791079	1790879	1790472	1790469	-	-	PA1645	52	54
	1791080	1793038		+	-	PA1646	8	8
	1794740	1793019		-	-	PA1647	16	22
	1795887	1794883		-	-	PA1648	8	5
	1796138	1796899		+	-	PA1649	10	11
1797010	1797058	1797951	1797951	+	-	PA1650	19	30
1799186	1799154	1797958	1797952	-	-	PA1651	33	59
	1799385	1800020		+	-	PA1652	23	37
1800598	1800547	1800071	1800021	-	-	PA1653	37	53
1800623	1800629	1801795	1801850	+	-	PA1654	80	112
1801851	1801851	1802453	1802633	+	-	PA1655	82	120
	1803626	1805182	1805182	+	-	PA1656	49	66
1805183	1805218	1805724	1805749	+	-	PA1657	61	93
1805753	1805753	1807228	1807232	+	-	PA1658	51	78
1807241	1807241	1807648		+	-	PA1659	24	51
	1808193	1809773		+	-	PA1660	16	30
	1809737	1810744	1810744	+	-	PA1661	11	29
	1810751	1813384	1813384	+	-	PA1662	14	28
	1813395	1814906		+	-	PA1663	10	26
	1814995	1815135		+	-	PA1664	10	32
1815153	1815153	1816346	1816346	+	-	PA1665	12	24
	1816352	1816858		+	-	PA1666	14	37
	1816855	1818186		+	-	PA1667	19	35
1818189	1818189	1819058		+	-	PA1668	14	28
1819074	1819074	1822601		+	-	PA1669	11	22
	1822601	1823329		+	stp1	PA1670	9	21
	1823326	1824315	1824315	+	stk1	PA1671	10	19
1824940	1824723	1824343	1824316	-	-	PA1672	53	43
1824942	1824969	1825430	1825494	+	-	PA1673	609	1021
1826040	1826040	1825495	1825495	-	folE2	PA1674	384	569

1826714	1826675	1826118	1826043	-	-	PA1675	77	133
	1827052	1826732	1826732	-	-	PA1676	32	28
1827822	1827821	1827225	1827210	-	-	PA1677	198	114
1827980	1827992	1828906	1828911	+	-	PA1678	98	151
1829787	1829710	1828931	1828931	-	-	PA1679	69	83
	1830771	1829788		-	-	PA1680	8	8
1830867	1830879	1831970	1831993	+	aroC	PA1681	176	177
1832010	1832014	1833165		+	-	PA1682	37	44
	1833162	1833779		+	-	PA1683	86	109
	1833776	1834321	1834321	+	-	PA1684	111	148
1834336	1834393	1835142	1835166	+	masA	PA1685	85	132
1836326	1836218	1835325	1835315	-	alkA	PA1686	47	55
1836332	1836367	1837227	1837227	+	speE	PA1687	161	215
1837228	1837388	1838257	1838259	+	-	PA1688	70	123
1838260	1838260	1840362	1840369	+	-	PA1689	120	156
	1841517	1840468		-	pscU	PA1690	9	8
	1842302	1841514		-	pscT	PA1691	8	7
	1842565	1842299		-	-	PA1692	26	21
	1843221	1842568	1842566	-	pscR	PA1693	42	33
	1844147	1843218		-	pscQ	PA1694	20	14
	1845253	1844144		-	pscP	PA1695	16	10
	1845717	1845241		-	pscO	PA1696	32	11
	1847036	1845714		-	-	PA1697	43	20
	1847227	1848093		+	popN	PA1698	43	20
	1848074	1848352		+	-	PA1699	37	17
	1848339	1848710		+	-	PA1700	40	17
	1848707	1849072		+	-	PA1701	25	9
1849077	1849077	1849406		+	-	PA1702	25	12
	1849403	1851523		+	pcrD	PA1703	26	10
	1851520	1851954		+	pcrR	PA1704	17	7
	1851982	1852278		+	pcrG	PA1705	25	8
	1852288	1853172		+	pcrV	PA1706	35	10

1853181	1853181	1853684		+	pcrH	PA1707	51	18
	1853665	1854837	1854839	+	popB	PA1708	107	47
1854849	1854849	1855736	1855752	+	popD	PA1709	89	37
1855862	1855862	1856299	1856300	+	exsC	PA1710	202	135
1856305	1856308	1856553	1856553	+	exsE	PA1711	132	85
1856554	1856562	1856975	1856988	+	exsB	PA1712	78	59
1857269	1857273	1858109	1858110	+	exsA	PA1713	108	64
1858135	1858207	1859037	1859070	+	exsD	PA1714	120	77
1859071	1859071	1859493		+	pscB	PA1715	52	27
	1859493	1861295		+	pscC	PA1716	40	22
	1861297	1862595		+	pscD	PA1717	26	14
	1862558	1862761	1862761	+	pscE	PA1718	57	28
1862762	1862764	1863021	1863022	+	pscF	PA1719	126	50
1863024	1863024	1863371		+	pscG	PA1720	54	31
	1863368	1863799		+	pscH	PA1721	58	26
	1863799	1864137		+	pscI	PA1722	72	34
	1864134	1864880		+	pscJ	PA1723	59	34
	1864889	1865515		+	pscK	PA1724	24	15
	1865494	1866138		+	pscL	PA1725	27	18
1868704	1868535	1866241	1866203	-	bglX	PA1726	29	40
1870997	1870763	1868706	1868705	-	mucR	PA1727	26	44
1871018	1871116	1871457	1871457	+	-	PA1728	55	55
1871458	1871555	1872202	1872382	+	-	PA1729	46	49
	1872602	1874014		+	-	PA1730	7	7
	1874017	1874970		+	-	PA1731	7	9
	1874967	1875767		+	-	PA1732	7	8
	1875849	1876580	1876580	+	-	PA1733	13	11
	1877409	1876588		-	-	PA1734	17	11
	1877522	1878409		+	-	PA1735	17	25
	1878980	1880185		+	-	PA1736	7	11
	1880199	1882343		+	-	PA1737	8	13
1883578	1883509	1882595	1882344	-	-	PA1738	46	43

	1883661	1884632		+	-	PA1739	7	6
	1884655	1885653		+	-	PA1740	15	11
1886255	1886033	1885662	1885654	-	-	PA1741	70	56
1886260	1886278	1887000	1887002	+	-	PA1742	251	157
	1887297	1887058		-	-	PA1743	6	7
	1887543	1887325		-	-	PA1744	7	5
	1888186	1887698		-	-	PA1745	4	4
1888369	1888407	1888913	1888953	+	-	PA1746	49	60
	1889173	1888985		-	-	PA1747	16	34
1890023	1890012	1889323	1889313	-	-	PA1748	70	93
1890196	1890196	1890681	1890681	+	-	PA1749	163	189
1891887	1891815	1890739	1890739	-	-	PA1750	372	445
1892064	1892133	1892510		+	-	PA1751	49	53
	1892510	1893457	1893463	+	-	PA1752	53	73
1893605	1893620	1894117	1894224	+	-	PA1753	45	78
1894228	1894249	1895223	1895223	+	cysB	PA1754	197	185
1895641	1895627	1895280	1895264	-	-	PA1755	41	51
1896495	1896495	1895692	1895684	-	cysH	PA1756	57	105
1896609	1896630	1897247	1897247	+	thrH	PA1757	138	203
1898651	1898651	1897290	1897285	-	pabB	PA1758	77	86
1898661	1898816	1901521	1901523	+	-	PA1759	44	67
1901524	1901524	1904247	1904440	+	-	PA1760	23	56
1904479	1904479	1904916	1904994	+	-	PA1761	19	36
	1904995	1905756		+	-	PA1762	10	21
	1905797	1906822		+	-	PA1763	9	10
	1906842	1908440		+	-	PA1764	5	6
	1908454	1909641		+	-	PA1765	5	7
1910681	1910681	1909698	1909687	-	-	PA1766	286	491
1912216	1912211	1910685	1910683	-	-	PA1767	327	546
1912847	1912756	1912217	1912217	-	-	PA1768	201	452
1913928	1913871	1913047	1913037	-	-	PA1769	109	168
1913930			1914034	?	-	predicted RN	355	670

1914035	1914037	1916412	1916415	+	ppsA	PA1770	554	767
1916423	1916482	1917492	1917593	+	estX	PA1771	59	95
1917594	1917599	1918087	1918087	+	-	PA1772	237	259
1918093	1918275	1919273	1919321	+	cmaX	PA1773	57	77
1919322	1919322	1919576	1919578	+	crfX	PA1774	203	474
1919579	1919579	1920403	1920567	+	cmpX	PA1775	192	397
1920568	1920568	1921065	1921136	+	sigX	PA1776	1257	2276
1921137	1921174	1922226	1922226	+	oprF	PA1777	13099	20254
1923043	1923032	1922295	1922227	-	cobA	PA1778	22	31
	1925770	1923044		-	-	PA1779	3	3
	1926123	1925797		-	nirD	PA1780	4	4
	1928626	1926176		-	nirB	PA1781	3	3
1928886			1928666	-	-	PA1781.1	1	1
	1930489	1928894		-	-	PA1782	2	2
	1931775	1930564		-	nasA	PA1783	1	1
	1932824	1932129		-	-	PA1784	5	7
	1933632	1933054		-	-	PA1785	5	6
	1934857	1933649		-	-	PA1786	3	6
1937754	1937644	1935035	1935020	-	acnB	PA1787	1080	1457
1938021	1938026	1938493	1938504	+	-	PA1788	61	133
1939432	1939412	1938549	1938533	-	-	PA1789	248	205
1939557	1939583	1940206	1940211	+	-	PA1790	73	185
1941597	1941586	1940255	1940255	-	-	PA1791	96	158
1942446	1942442	1941720	1941720	-	-	PA1792	163	227
1942987	1942944	1942447	1942447	-	ppiB	PA1793	1233	1641
1943030	1943067	1944737	1944746	+	glnS	PA1794	416	625
1944747	1944747	1946129	1946135	+	cysS	PA1795	351	551
1947060	1947041	1946187	1946181	-	folD	PA1796	213	355
1947320			1947396	+	-	PA1796.1	999	3570
1947448			1947523	+	-	PA1796.2	266	851
1947578			1947662	+	-	PA1796.3	220	531
1947729			1947804	+	-	PA1796.4	86	219

	1950334	1948502		-	-	PA1797	20	18
	1951725	1950439	1950335	-	parS	PA1798	45	67
1952442	1952433	1951726		-	parR	PA1799	49	101
1952625	1952665	1953975	1953975	+	tig	PA1800	2734	3785
1953976	1954069	1954710	1954814	+	clpP	PA1801	981	1104
1954815	1954815	1956095	1956095	+	clpX	PA1802	1451	1734
1956096	1956227	1958623	1958623	+	lon	PA1803	577	617
1958624	1958759	1959031	1959082	+	hupB	PA1804	9666	8607
1959083			1959159	+	-	PA1804.1	2017	2088
1959160	1959263	1961128	1961140	+	ppiD	PA1805	416	496
1962032	1962024	1961227	1961221	-	fabI	PA1806	46	70
1963657	1963656	1962046	1962033	-	-	PA1807	36	54
1964681	1964677	1963658	1963658	-	-	PA1808	30	49
1965765	1965764	1964682	1964682	-	-	PA1809	35	59
	1967618	1965771	1965766	-	-	PA1810	52	69
1969490	1969444	1967615		-	-	PA1811	36	43
1971242	1971239	1969635	1969635	-	mltD	PA1812	191	243
1972106	1972093	1971317	1971244	-	-	PA1813	35	32
1972110	1972195	1972956	1972956	+	-	PA1814	53	71
1972957	1972959	1973405	1973406	+	rnhA	PA1815	97	171
1973449	1973470	1974210	1974220	+	dnaQ	PA1816	110	164
1974632	1974627	1974238	1974238	-	-	PA1817	61	93
1974794	1974821	1977076	1977099	+	ldcA	PA1818	73	177
1977100	1977100	1978455		+	-	PA1819	43	83
1979960	1979941	1978439		-	nhaB	PA1820	39	40
1980116	1980165	1980977	1980977	+	-	PA1821	252	213
1980978	1981017	1982705		+	fimL	PA1822	171	182
	1982705	1983541		+	-	PA1823	138	146
1984298	1984288	1983509		-	-	PA1824	42	67
	1984389	1985033		+	-	PA1825	26	42
	1985935	1985030		-	-	PA1826	13	19
	1986067	1986828		+	-	PA1827	4	3

1987756	1987756	1986989	1986972	-	-	PA1828	60	49
1988941	1988851	1987781	1987757	-	-	PA1829	44	35
1989434	1989423	1989109	1989085	-	-	PA1830	596	646
1989435			1989446	?	-	predicted RN	355	323
1990245	1990194	1989484	1989447	-	-	PA1831	66	68
1990402	1990428	1991453	1991487	+	-	PA1832	94	114
1991512	1991512	1992504	1992583	+	-	PA1833	82	111
1992584	1992584	1993474		+	-	PA1834	18	25
	1993898	1993461		-	-	PA1835	27	15
	1994021	1994602		+	-	PA1836	20	24
	1995164	1994667	1994661	-	-	PA1837	98	188
1996850	1996806	1995148		-	cysI	PA1838	125	281
1998549	1998549	1997509	1997250	-	-	PA1839	46	88
1998587	1998587	1998949	1998949	+	-	PA1840	97	165
1998950	1998963	1999460	1999461	+	-	PA1841	108	146
1999874	1999874	1999512	1999479	-	-	PA1842	87	104
2003611	2003593	1999889	1999875	-	metH	PA1843	77	88
2004332	2004332	2003868		-	-	PA1844	26	33
2005133	2004892	2004374	2004333	-	-	PA1845	33	44
2005254	2005254	2007542	2007664	+	cti	PA1846	55	71
2007665	2007665	2008249	2008263	+	-	PA1847	291	279
	2009477	2008308		-	-	PA1848	7	8
	2009724	2009482		-	-	PA1849	6	3
	2010751	2009747		-	-	PA1850	11	10
	2011000	2012205		+	-	PA1851	9	16
2012583	2012530	2012255	2012243	-	-	PA1852	418	1003
2012584			2012673	?	-	predicted RN	179	14
2012769	2012815	2013678		+	-	PA1853	40	53
	2014823	2013666		-	-	PA1854	14	18
	2015136	2014915		-	-	PA1855	4	7
2016927	2016581	2015139	2015137	-	-	PA1856	27	25
2017888	2017851	2016928	2016928	-	-	PA1857	58	84

	2018794	2017967		-	str	PA1858	12	14
	2019690	2018803		-	-	PA1859	19	20
	2019776	2020603		+	-	PA1860	7	6
2021710	2021710	2020625		-	modC	PA1861	24	31
2022398	2022398	2021712	2021711	-	modB	PA1862	34	55
2023199	2023166	2022411	2022399	-	modA	PA1863	44	51
	2023931	2023281		-	-	PA1864	7	2
2024065	2024065	2025744		+	-	PA1865	20	20
	2025737	2028013		+	-	PA1866	20	16
	2028454	2028981		+	xphA	PA1867	5	4
	2028968	2031298		+	xqhA	PA1868	6	3
2031407	2031466	2031705	2031987	+	-	PA1869	60	49
	2031988	2032413		+	-	PA1870	3	2
	2032695	2033951		+	lasA	PA1871	14	12
	2034857	2034066		-	-	PA1872	31	25
	2035940	2034918		-	-	PA1873	5	3
	2036441	2043847		+	-	PA1874	6	6
	2043847	2045124		+	-	PA1875	3	3
	2045114	2047285		+	-	PA1876	3	3
	2047275	2048462		+	-	PA1877	1	1
	2049148	2048570		-	-	PA1878	10	13
	2049791	2049237		-	-	PA1879	12	15
2052144	2052144	2049949		-	-	PA1880	6	9
	2052610	2052149		-	-	PA1881	9	11
	2052941	2053264		+	-	PA1882	12	9
	2053277	2053675		+	-	PA1883	3	11
	2054223	2053672		-	-	PA1884	20	16
	2054309	2054842		+	-	PA1885	10	8
	2054911	2057274		+	polB	PA1886	13	20
	2057946	2057278		-	-	PA1887	9	10
	2059339	2057930		-	-	PA1888	3	5
2059411	2059569	2060552	2060565	+	-	PA1889	21	35

2061207	2061189	2060566	2060566	-	-	PA1890	47	53
	2061667	2061278		-	-	PA1891	4	6
	2062401	2061664		-	-	PA1892	3	9
	2064827	2062398		-	-	PA1893	5	12
	2065545	2064853	2064828	-	-	PA1894	11	31
	2066767	2065493		-	-	PA1895	8	18
	2067955	2066786		-	-	PA1896	7	15
2068942	2068728	2067961	2067956	-	-	PA1897	11	24
	2069490	2070203		+	qscR	PA1898	8	9
	2070685	2071173		+	phzA2	PA1899	1	0
	2071209	2071697		+	phzB2	PA1900	4	4
	2071721	2072938		+	phzC2	PA1901	12	10
	2072935	2073558		+	phzD2	PA1902	3	2
	2073555	2075438		+	phzE2	PA1903	4	2
	2075452	2076288		+	phzF2	PA1904	3	4
	2076311	2076958		+	phzG2	PA1905	6	6
	2077583	2077047		-	-	PA1906	15	21
	2079266	2077626		-	-	PA1907	2	3
	2080474	2079263		-	-	PA1908	4	3
	2081812	2080661		-	-	PA1909	3	4
	2084267	2081853		-	femA	PA1910	3	4
	2085426	2084476		-	femR	PA1911	5	8
2086020	2085929	2085423		-	femI	PA1912	9	27
2086708	2086701	2086021	2086021	-	-	PA1913	37	119
	2088034	2086808		-	-	PA1914	2	3
	2090149	2088602		-	-	PA1915	14	16
	2091445	2090213		-	-	PA1916	1	2
	2091837	2091490		-	-	PA1917	1	4
	2093250	2091850		-	-	PA1918	2	2
	2094142	2093444		-	nrdG	PA1919	9	10
	2096356	2094329		-	nrdD	PA1920	6	4
	2097323	2096514		-	-	PA1921	1	0

	2097491	2099452		+	-	PA1922	2	1
	2099452	2103297		+	-	PA1923	1	0
	2103294	2103770		+	-	PA1924	2	3
	2103770	2104096		+	-	PA1925	6	4
2104178	2104597	2106447	2106579	+	-	PA1926	62	78
	2106580	2108880		+	metE	PA1927	8	10
	2109511	2108942		-	rimJ	PA1928	13	15
	2109854	2109558		-	-	PA1929	14	20
	2110226	2111521		+	-	PA1930	6	4
	2111691	2112203		+	-	PA1931	8	5
	2112200	2113189		+	-	PA1932	10	8
	2113186	2115390	2115572	+	-	PA1933	55	46
2115865	2115885	2116265	2116846	+	-	PA1934	38	40
	2117734	2117030		-	-	PA1935	16	25
	2117897	2118097		+	-	PA1936	34	47
	2118585	2118893		+	-	PA1937	20	26
	2118926	2119747		+	-	PA1938	13	22
2122575	2122222	2120225	2119786	-	-	PA1939	106	137
2123718	2123718	2122579	2122579	-	-	PA1940	25	49
2125810	2125792	2123897	2123719	-	-	PA1941	25	42
	2126334	2126104		-	-	PA1942	11	10
	2127493	2126396		-	-	PA1943	12	16
	2127684	2129171		+	-	PA1944	13	17
	2129307	2130635		+	-	PA1945	6	9
2130794	2130854	2131813	2131834	+	rbsB	PA1946	158	99
2131835	2131835	2133367	2133384	+	rbsA	PA1947	23	51
2133389	2133391	2134389	2134389	+	rbsC	PA1948	23	64
2134390	2134393	2135406	2135447	+	rbsR	PA1949	31	85
2135448	2135460	2136386	2136392	+	rbsK	PA1950	38	91
	2137785	2136520		-	-	PA1951	12	29
	2138598	2137846		-	-	PA1952	0	2
	2139292	2138612		-	-	PA1953	3	2

	2140376	2139354		-	-	PA1954	3	4
	2141002	2140433		-	-	PA1955	1	3
	2141487	2140999		-	-	PA1956	1	2
	2142317	2141790		-	-	PA1957	15	22
2142934	2142889	2142314		-	-	PA1958	32	54
2143067	2143173	2144006	2144013	+	bacA	PA1959	63	95
2144014	2144077	2144799	2144809	+	-	PA1960	32	39
	2145745	2144810		-	-	PA1961	19	24
	2145894	2146502		+	azoR2	PA1962	7	10
2146602	2146609	2146875	2146949	+	-	PA1963	77	63
2148529	2148526	2146961	2146960	-	-	PA1964	122	148
2148837	2148854	2149189	2149456	+	-	PA1965	42	58
2149467	2149472	2149843	2149843	+	-	PA1966	46	80
2150523	2150364	2149864	2149844	-	-	PA1967	48	57
2150524	2150524	2150781	2150787	+	-	PA1968	61	74
2150819	2150828	2151220	2151224	+	-	PA1969	118	137
2151754	2151526	2151287	2151231	-	-	PA1970	30	38
	2153068	2151755		-	braZ	PA1971	24	15
	2153594	2155297		+	-	PA1972	1	3
	2155374	2157701		+	pqqF	PA1973	15	12
	2157968	2159167		+	-	PA1974	2	1
	2159270	2160433		+	-	PA1975	3	2
	2160363	2163008		+	ercS'	PA1976	2	2
	2163873	2163010		-	-	PA1977	4	2
	2164548	2163883		-	erbR	PA1978	22	4
	2165213	2165863		+	eraS	PA1979	1	1
	2165876	2166553		+	eraR	PA1980	2	3
	2167227	2166580		-	-	PA1981	2	0
	2169152	2167281		-	exaA	PA1982	1	2
	2169464	2169901		+	exaB	PA1983	0	0
2169988	2169988	2171508	2171508	+	exaC	PA1984	22	83
2171852			2171864	?	-	predicted RN	41	17

	2171865	2171936		+	pqqA	PA1985	46	9
2171989	2171989	2172903	2172903	+	pqqB	PA1986	16	4
	2172913	2173665		+	pqqC	PA1987	13	4
	2173662	2173940		+	pqqD	PA1988	21	8
	2173912	2175057	2175057	+	pqqE	PA1989	12	6
	2175062	2176888		+	pqqH	PA1990	5	2
	2176974	2178137		+	-	PA1991	25	34
	2178121	2179815		+	ercS	PA1992	11	26
	2181056	2179848		-	-	PA1993	11	24
	2181744	2181181	2181118	-	-	PA1994	30	36
2182097	2182097	2181741		-	-	PA1995	35	49
2182451	2182394	2182116	2182098	-	ppiC1	PA1996	40	68
	2184407	2182452		-	-	PA1997	20	22
	2185395	2184475		-	dhcR	PA1998	20	10
2185478	2185527	2186225	2186259	+	dhcA	PA1999	19	370
2186260	2186260	2186916	2186926	+	dhcB	PA2000	19	319
2186992	2187065	2188246	2188246	+	atoB	PA2001	29	311
2188247	2188459	2189883	2190083	+	-	PA2002	7	91
2190890	2190854	2190084	2190084	-	bdhA	PA2003	9	49
	2192282	2190891		-	-	PA2004	5	10
2194019	2193989	2192544	2192454	-	-	PA2005	21	47
2195470	2195410	2194058	2194058	-	-	PA2006	11	111
	2196132	2195494	2195477	-	maiA	PA2007	41	509
2197427	2197427	2196129		-	fahA	PA2008	59	558
2198802	2198730	2197432	2197428	-	hmgA	PA2009	63	647
2198869	2198891	2199694	2199761	+	-	PA2010	27	62
2200684	2200664	2199762	2199762	-	liuE	PA2011	37	135
	2202652	2200685	2200685	-	liuD	PA2012	24	111
2203446	2203446	2202649		-	liuC	PA2013	18	87
2205189	2205067	2203460	2203447	-	liuB	PA2014	23	109
2206372	2206353	2205190	2205190	-	liuA	PA2015	61	273
2206814	2206806	2206402	2206384	-	liuR	PA2016	87	125

	2206999	2207928		+	-	PA2017	8	10
	2211306	2208169		-	-	PA2018	38	25
	2212512	2211322		-	-	PA2019	40	26
2212661	2212677	2213309	2213314	+	-	PA2020	77	87
	2213539	2213315		-	-	PA2021	13	9
	2213693	2215054		+	-	PA2022	6	10
2215081	2215102	2215941	2215954	+	galU	PA2023	498	823
	2216543	2216121		-	-	PA2024	12	14
2216681	2216688	2218043	2218043	+	gor	PA2025	96	87
2219142	2219099	2218098	2218069	-	-	PA2026	81	13
	2219645	2219253		-	-	PA2027	6	6
	2219778	2220251	2220251	+	-	PA2028	24	27
	2220275	2220574		+	-	PA2029	29	25
	2220906	2220649		-	-	PA2030	22	27
	2221157	2220903		-	-	PA2031	14	17
2221227	2221337	2222761	2222895	+	-	PA2032	41	60
	2222896	2223804		+	-	PA2033	5	6
	2223804	2224478		+	-	PA2034	5	3
	2226144	2224486		-	-	PA2035	5	5
	2226879	2227400		+	-	PA2036	6	7
	2227541	2229001		+	-	PA2037	23	33
	2229440	2229126	2229006	-	-	PA2038	24	40
2230473	2230183	2229425		-	-	PA2039	24	34
2230689	2230689	2232065	2232076	+	-	PA2040	222	101
	2232222	2233592		+	-	PA2041	31	18
2233942	2234080	2235309	2235315	+	-	PA2042	183	225
2235364	2235430	2236332	2236490	+	-	PA2043	33	30
2238377	2238366	2236492	2236492	-	-	PA2044	112	110
2238819	2238801	2238541	2238378	-	-	PA2045	32	46
	2239267	2238860		-	-	PA2046	1	3
	2241291	2240302		-	-	PA2047	10	10
	2241705	2242112		+	-	PA2048	3	15

2244181	2243603	2242170	2242113	-	-	PA2049	25	30
	2244492	2244998		+	-	PA2050	1	2
	2244995	2245948		+	-	PA2051	1	4
	2246456	2245986		-	cynS	PA2052	9	10
	2247158	2246496		-	cynT	PA2053	1	4
	2247273	2248160		+	cynR	PA2054	14	11
	2249582	2248167		-	-	PA2055	4	3
	2249693	2250595		+	-	PA2056	33	27
	2251275	2253815		+	-	PA2057	2	3
	2253819	2255627		+	-	PA2058	1	2
	2255629	2256702		+	-	PA2059	3	2
	2256704	2257720		+	-	PA2060	1	1
	2257722	2259332		+	-	PA2061	3	3
	2259478	2260659		+	-	PA2062	6	8
2260831	2260862	2262085	2262104	+	-	PA2063	51	85
	2263082	2262105		-	pcoB	PA2064	13	20
	2264977	2263079		-	pcoA	PA2065	1	4
	2265764	2265126		-	-	PA2066	4	5
	2266429	2265761		-	-	PA2067	3	3
	2267594	2266431		-	-	PA2068	2	1
	2269362	2267638		-	-	PA2069	2	3
	2272184	2269542		-	-	PA2070	3	3
	2272460	2274568		+	fusA2	PA2071	17	17
	2274740	2277334		+	-	PA2072	1	1
	2277552	2278982		+	-	PA2073	8	5
	2278982	2279794		+	-	PA2074	4	2
	2281578	2279917		-	-	PA2075	2	4
2282241	2282480	2283382	2283418	+	-	PA2076	36	45
	2285323	2283419		-	-	PA2077	6	10
	2287237	2285363		-	-	PA2078	2	3
2289084	2288929	2287523	2287387	-	-	PA2079	26	46
2290335	2290335	2289085	2289085	-	kynU	PA2080	87	120

2290982	2290980	2290339	2290336	-	kynB	PA2081	114	140
2291019	2291113	2291589	2291790	+	-	PA2082	42	43
	2291791	2293065		+	-	PA2083	11	12
	2293153	2294985		+	-	PA2084	4	6
	2295013	2295522		+	-	PA2085	3	3
	2295533	2296435		+	-	PA2086	3	2
	2296432	2297088		+	-	PA2087	2	1
	2297072	2297920		+	-	PA2088	2	3
	2298012	2300663		+	-	PA2089	5	6
	2300676	2301755		+	-	PA2090	0	2
	2301752	2303035		+	-	PA2091	0	1
	2303022	2304215		+	-	PA2092	1	2
	2304318	2304827		+	-	PA2093	5	7
	2304824	2305780		+	-	PA2094	3	1
	2306627	2305782		-	-	PA2095	4	4
	2307810	2306776		-	-	PA2096	7	7
	2307957	2309432		+	-	PA2097	4	10
	2309443	2310372		+	-	PA2098	3	7
	2310357	2311112		+	-	PA2099	2	8
	2312766	2311333		-	-	PA2100	32	31
	2312899	2313789		+	-	PA2101	16	15
	2313791	2314249		+	-	PA2102	15	18
	2314512	2315690		+	-	PA2103	22	22
	2315709	2316626	2316626	+	-	PA2104	31	36
2316627	2316708	2317403		+	-	PA2105	38	47
	2317400	2318134	2318225	+	-	PA2106	30	36
	2318624	2318226		-	-	PA2107	4	9
	2318795	2320567		+	-	PA2108	3	37
2321125	2321062	2320586	2320568	-	-	PA2109	3	651
	2322071	2321130	2321126	-	-	PA2110	3	1057
	2322781	2322068		-	-	PA2111	6	2416
2323553	2323521	2322778		-	-	PA2112	5	1753

2324790	2324783	2323554	2323554	-	opdO	PA2113	5	1255
2324791			2324807	?	-	predicted RN	2	1535
2326142	2326079	2324808	2324808	-	-	PA2114	30	1882
2326269	2326334	2327287	2327382	+	-	PA2115	14	96
2327388	2327394	2328191		+	-	PA2116	16	638
2329156	2329156	2328176		-	-	PA2117	20	323
2329346	2329348	2330424	2330958	+	ada	PA2118	16	47
2332110	2332059	2330959	2330959	-	-	PA2119	110	109
	2332820	2332392		-	-	PA2120	6	9
	2333873	2332968		-	-	PA2121	10	6
	2333999	2335135		+	-	PA2122	5	5
	2335172	2336104		+	-	PA2123	11	12
	2336209	2337846		+	-	PA2124	1	1
	2337868	2339316		+	-	PA2125	2	10
2340397	2339987	2339352	2339317	-	-	PA2126	6	73
2342128	2341640	2340414	2340398	-	-	PA2127	52	151
2342403	2342493	2343044	2343044	+	cupA1	PA2128	20	338
2343045	2343132	2343878		+	cupA2	PA2129	2	66
	2343862	2346480		+	cupA3	PA2130	2	18
	2346477	2347838		+	cupA4	PA2131	0	12
	2347828	2348541		+	cupA5	PA2132	0	9
	2348538	2349395		+	-	PA2133	1	8
	2349488	2350060		+	-	PA2134	1	5
	2350089	2351453		+	-	PA2135	0	1
	2352430	2351906		-	-	PA2136	7	4
	2352532	2353068		+	-	PA2137	1	2
	2353086	2355608		+	-	PA2138	1	1
	2355684	2355806		+	-	PA2139	0	5
	2355918	2356157		+	-	PA2140	0	0
	2356168	2356716		+	-	PA2141	0	0
	2356713	2357573		+	-	PA2142	1	0
	2358024	2358311		+	-	PA2143	4	3

2358364	2360802	+	glgP	PA2144	9	8
2361207	2360809	-	-	PA2145	1	3
2361706	2361873	+	-	PA2146	1	0
2361954	2364083	+	katE	PA2147	2	4
2364311	2364802	+	-	PA2148	0	0
2364816	2365058	+	-	PA2149	1	0
2365081	2365962	+	-	PA2150	1	1
2366106	2368100	+	-	PA2151	2	3
2368111	2371413	+	-	PA2152	2	3
2371410	2373608	+	glgB	PA2153	1	1
2374605	2373610	-	-	PA2154	1	1
2375807	2374602	-	-	PA2155	1	1
2376541	2375804	-	-	PA2156	1	1
2377476	2376538	-	-	PA2157	7	3
2378727	2377480	-	-	PA2158	1	3
2379210	2378794	-	-	PA2159	1	2
2381460	2379310	-	-	PA2160	1	1
2381778	2381473	-	-	PA2161	0	1
2384555	2381775	-	-	PA2162	1	1
2386602	2384548	-	-	PA2163	1	1
2388346	2386595	-	-	PA2164	0	1
2389887	2388346	-	-	PA2165	1	2
2390255	2390620	+	-	PA2166	11	6
2390949	2392046	+	-	PA2167	1	2
2392043	2392819	+	-	PA2168	1	0
2392945	2393397	+	-	PA2169	0	1
2393424	2393633	+	-	PA2170	1	0
2393708	2394178	+	-	PA2171	3	4
2394182	2395258	+	-	PA2172	3	3
2395285	2395635	+	-	PA2173	4	4
2396252	2395944	-	-	PA2174	5	5
2396883	2396536	-	-	PA2175	5	3

	2397486	2396896		-	-	PA2176	1	2
	2397569	2399668		+	-	PA2177	3	4
	2400280	2399672		-	-	PA2178	1	1
	2401605	2400655		-	-	PA2179	2	1
	2402965	2401589		-	-	PA2180	1	1
	2403151	2404284		+	-	PA2181	0	1
	2404655	2404386		-	-	PA2182	3	10
	2405233	2404949		-	-	PA2183	5	4
	2405739	2405230		-	-	PA2184	1	3
	2405993	2406877		+	katN	PA2185	4	4
	2406961	2407131		+	-	PA2186	0	2
	2407236	2407661		+	-	PA2187	3	2
	2408847	2407681		-	-	PA2188	2	2
	2409107	2409661		+	-	PA2189	5	3
	2409837	2410181		+	-	PA2190	3	1
	2411480	2410344		-	exoY	PA2191	18	8
	2411709	2412122		+	-	PA2192	2	1
	2412546	2412860		+	hcnA	PA2193	55	98
	2412857	2414251	2414251	+	hcnB	PA2194	38	82
	2414254	2415507	2415507	+	hcnC	PA2195	34	89
2415629	2415661	2416245	2416295	+	-	PA2196	40	52
2416376	2416376	2417413		+	-	PA2197	60	50
	2417410	2417754	2417754	+	-	PA2198	39	33
2417755	2417760	2418635	2418722	+	-	PA2199	94	81
	2418723	2420318		+	-	PA2200	16	16
2420374	2420443	2421327	2421342	+	-	PA2201	28	26
2422020	2422020	2421343	2421343	-	-	PA2202	12	30
2422818	2422738	2422022	2422021	-	-	PA2203	4	28
2423720	2423625	2422819	2422819	-	-	PA2204	45	180
	2424400	2423924		-	-	PA2205	13	17
	2425429	2424482		-	-	PA2206	11	11
	2427017	2425497		-	-	PA2207	1	2

	2427544	2427014		-	-	PA2208	0	2
	2428669	2427572		-	-	PA2209	1	2
	2428852	2430177		+	-	PA2210	2	1
	2430170	2431129		+	-	PA2211	2	4
	2431126	2432139		+	-	PA2212	2	5
	2432312	2433562		+	-	PA2213	1	1
	2433748	2435070		+	-	PA2214	1	0
	2435101	2436276		+	-	PA2215	2	6
	2436304	2437299		+	-	PA2216	1	3
	2437428	2439011		+	-	PA2217	1	3
	2440253	2439150		-	-	PA2218	9	12
	2441555	2440347		-	opdE	PA2219	4	5
	2441771	2442691		+	-	PA2220	38	36
	2443161	2444366		+	-	PA2221	4	7
2445533	2445533	2444886		-	-	PA2222	18	19
	2446564	2445545		-	-	PA2223	17	27
	2447325	2446597		-	-	PA2224	7	13
	2447989	2447573		-	-	PA2225	5	13
2448533	2448533	2448033		-	-	PA2226	17	22
	2449545	2448568		-	vqsM	PA2227	21	34
	2450765	2449554		-	-	PA2228	5	10
2451477	2451707	2452426	2452463	+	-	PA2229	26	27
2452464	2452469	2453140	2453441	+	-	PA2230	31	50
2453539	2453667	2455103		+	pslA	PA2231	38	112
	2455103	2456569		+	pslB	PA2232	47	166
	2456569	2457480	2457480	+	pslC	PA2233	39	137
2457481	2457510	2458280	2458284	+	pslD	PA2234	52	203
2458295	2458295	2460283		+	pslE	PA2235	59	225
	2460283	2461470		+	pslF	PA2236	32	131
	2461460	2462788	2462791	+	pslG	PA2237	30	137
2462792	2462797	2464005		+	pslH	PA2238	27	123
	2463996	2465099	2465099	+	pslI	PA2239	18	94

2465100	2465102	2466538	2466539	+	pslJ	PA2240	16	78
2466540	2466540	2467949	2468016	+	pslK	PA2241	9	42
2468018	2468032	2469099	2469135	+	pslL	PA2242	26	101
	2469286	2471019		+	pslM	PA2243	2	8
	2471075	2472076		+	pslN	PA2244	13	11
	2472104	2472409		+	pslO	PA2245	16	23
	2472903	2472442		-	bkdR	PA2246	40	20
2473034	2473213	2474445		+	bkdA1	PA2247	75	917
	2474442	2475494		+	bkdA2	PA2248	56	881
	2475495	2476781	2476781	+	bkdB	PA2249	47	749
2476782	2476785	2478179	2478186	+	lpdV	PA2250	47	808
2479168	2479130	2478312	2478196	-	-	PA2251	28	43
2479257	2479299	2480744	2480744	+	-	PA2252	509	52
2480779	2480844	2481830	2481834	+	ansA	PA2253	155	34
	2482045	2483031		+	pvcA	PA2254	4	1
	2483049	2483924		+	pvcB	PA2255	4	2
	2483976	2485478		+	pvcC	PA2256	4	2
	2485471	2486118		+	pvcD	PA2257	5	2
	2487293	2486355		-	ptxR	PA2258	12	6
	2487856	2488878		+	ptxS	PA2259	26	21
	2488950	2489732		+	-	PA2260	3	1
	2489725	2490675		+	-	PA2261	4	2
	2490738	2492045		+	-	PA2262	3	3
	2492064	2493050		+	-	PA2263	2	1
2493186	2493218	2493934	2493936	+	-	PA2264	95	112
2493937	2493937	2495712	2495723	+	-	PA2265	83	96
2495724	2495724	2497043	2497115	+	-	PA2266	55	70
	2498012	2497116		-	-	PA2267	32	33
	2498099	2499169		+	-	PA2268	12	10
	2500338	2499133		-	-	PA2269	21	18
2500472	2500485	2501075	2501082	+	-	PA2270	51	59
	2501083	2501598		+	-	PA2271	24	20

	2501720	2503417	2503417	+	pbpC	PA2272	23	30
	2503895	2503425		-	soxR	PA2273	34	20
	2503973	2504350		+	-	PA2274	6	7
	2505436	2504375		-	-	PA2275	1	5
	2505645	2506535		+	-	PA2276	9	9
	2506614	2506964		+	arsR	PA2277	20	27
	2506978	2508261		+	arsB	PA2278	14	17
	2508293	2508763		+	arsC	PA2279	30	31
2508764	2508775	2509467	2509467	+	-	PA2280	44	61
2510603	2510334	2509480	2509468	-	-	PA2281	51	68
	2510622	2511050		+	-	PA2282	8	14
	2511057	2512514		+	-	PA2283	6	8
	2512511	2513182		+	-	PA2284	9	8
	2513179	2513841		+	-	PA2285	12	15
	2513842	2515386		+	-	PA2286	11	13
	2515373	2516008	2516008	+	-	PA2287	30	28
2516313	2516308	2516021	2516017	-	-	PA2288	66	58
2518582	2518561	2516429	2516429	-	-	PA2289	34	65
2521188	2521104	2518693	2518690	-	gcd	PA2290	144	251
2522724	2522616	2521258	2521258	-	-	PA2291	71	184
	2523239	2522958		-	-	PA2292	11	11
	2524201	2523236		-	-	PA2293	4	4
	2525052	2524198		-	-	PA2294	4	2
	2525846	2525049		-	-	PA2295	2	1
	2527277	2525871		-	-	PA2296	6	3
	2527657	2527412		-	-	PA2297	8	7
	2529467	2527743		-	-	PA2298	11	9
	2530213	2529464		-	-	PA2299	17	17
	2531840	2530389		-	chiC	PA2300	5	3
	2532601	2532059		-	-	PA2301	12	11
	2539043	2532669	2532669	-	ambE	PA2302	12	18
	2540082	2539063	2539063	-	ambD	PA2303	10	16

2541167	2541167	2540079		-	ambC	PA2304	15	18
2544946	2544946	2541197	2541168	-	ambB	PA2305	37	45
2545665	2545659	2545042	2544947	-	ambA	PA2306	63	97
	2546635	2545769		-	-	PA2307	4	3
	2547500	2546652		-	-	PA2308	1	0
	2548530	2547508		-	-	PA2309	2	2
	2549458	2548571		-	-	PA2310	3	1
2549728	2549748	2549906	2549960	+	-	PA2311	2	7
	2549961	2550542		+	-	PA2312	3	4
	2550626	2551240		+	-	PA2313	21	24
	2552675	2551422		-	-	PA2314	1	1
	2553850	2552675		-	-	PA2315	1	1
	2553965	2554858		+	-	PA2316	16	14
	2554962	2556260		+	-	PA2317	6	5
	2556293	2556658		+	-	PA2318	19	18
	2556948	2557964		+	-	PA2319	34	33
	2559949	2558918		-	gntR	PA2320	40	17
	2560144	2560665		+	-	PA2321	30	9
	2560762	2562114		+	-	PA2322	16	8
2562428	2562447	2564072	2564176	+	-	PA2323	75	54
	2564290	2565549		+	-	PA2324	1	2
	2565580	2566815		+	-	PA2325	1	0
	2568284	2566875		-	-	PA2326	1	1
	2569693	2568929		-	-	PA2327	8	9
	2570889	2569690		-	-	PA2328	6	10
	2571691	2570855		-	-	PA2329	6	11
2572804	2572755	2571688		-	-	PA2330	9	17
2573469	2573365	2572805	2572805	-	-	PA2331	20	22
	2573470	2574375		+	-	PA2332	33	25
	2575992	2574376		-	-	PA2333	9	7
	2577000	2576089		-	-	PA2334	3	4
	2577150	2579519		+	-	PA2335	2	1

	2579542	2580882		+	-	PA2336	2	1
	2581034	2581939		+	mtlR	PA2337	12	10
	2582097	2583407		+	-	PA2338	2	4
	2583483	2584415		+	-	PA2339	3	2
	2584426	2585259		+	-	PA2340	5	4
	2585299	2586411		+	-	PA2341	2	5
	2586434	2587909		+	mtlD	PA2342	2	2
	2587906	2589414		+	mtlY	PA2343	1	1
	2589455	2590387		+	mtlZ	PA2344	14	17
	2591665	2590430		-	-	PA2345	11	9
	2592084	2593319		+	-	PA2346	2	2
	2593330	2594547		+	-	PA2347	2	2
	2594565	2595953		+	-	PA2348	1	3
	2595985	2596779		+	-	PA2349	2	2
	2596776	2597885		+	-	PA2350	1	2
	2597869	2598522		+	-	PA2351	3	1
2598523	2598700	2599827	2599892	+	-	PA2352	65	47
	2601047	2599893		-	-	PA2353	31	31
	2602349	2601219		-	-	PA2354	1	1
	2603530	2602346		-	-	PA2355	2	0
	2604705	2603560		-	msuD	PA2356	1	1
	2605275	2604715		-	msuE	PA2357	1	1
	2605824	2605435		-	-	PA2358	25	8
	2607022	2605937		-	-	PA2359	4	3
	2608232	2607132		-	-	PA2360	2	3
	2612044	2608229		-	-	PA2361	3	2
	2612799	2612041		-	-	PA2362	3	4
2614148	2614148	2612817		-	-	PA2363	7	7
	2614684	2614208		-	-	PA2364	15	9
	2614893	2615438		+	-	PA2365	10	4
2615461	2615461	2616945		+	-	PA2366	6	7
2617019	2617019	2617516	2617516	+	-	PA2367	5	5

	2617529	2617954		+	-	PA2368	2	1
	2617938	2619731		+	-	PA2369	1	2
	2619695	2620711		+	-	PA2370	1	1
	2620713	2623262		+	-	PA2371	2	4
	2623284	2623856		+	-	PA2372	18	20
	2624204	2626210		+	-	PA2373	7	12
	2626221	2626757		+	-	PA2374	2	5
	2627175	2626780		-	-	PA2375	3	4
	2627452	2628093		+	-	PA2376	10	8
	2628225	2629499		+	-	PA2377	5	3
	2632231	2629916	2629762	-	-	PA2378	93	53
2632725	2632698	2632228		-	-	PA2379	179	105
2633284	2633199	2632963	2632942	-	-	PA2380	44	52
	2633494	2633736		+	-	PA2381	29	16
	2634954	2633803		-	lldA	PA2382	8	9
	2635971	2635051		-	-	PA2383	12	9
	2636336	2636013		-	-	PA2384	6	21
	2638805	2636517		-	pvdQ	PA2385	1	2
2640391	2640259	2638928	2638806	-	pvdA	PA2386	8	21
2640988	2640871	2640392	2640392	-	fpvI	PA2387	57	69
2640993	2641035	2642030	2642030	+	fpvR	PA2388	104	133
2642031	2642131	2643306		+	pvdR	PA2389	18	29
	2643306	2645297		+	pvdT	PA2390	13	20
	2645303	2646727		+	opmQ	PA2391	13	18
	2648410	2646776		-	pvdP	PA2392	3	6
	2648626	2649972		+	-	PA2393	3	4
	2649995	2651278		+	pvdN	PA2394	3	5
	2651307	2652161		+	pvdO	PA2395	3	7
2653142	2653057	2652230	2652162	-	pvdF	PA2396	16	24
	2653435	2655084		+	pvdE	PA2397	7	10
2655144	2655187	2657634	2657639	+	fpvA	PA2398	38	87
	2665144	2657798		-	pvdD	PA2399	5	10

	2671629	2665156		-	pvdJ	PA2400	5	9
	2687178	2671729		-	-	PA2402	3	7
2687442	2687497	2688708		+	-	PA2403	9	22
	2688705	2689244		+	-	PA2404	11	26
	2689241	2689570		+	-	PA2405	9	27
	2689567	2690127		+	-	PA2406	6	15
2690128	2690160	2691113		+	-	PA2407	7	25
	2691110	2691865		+	-	PA2408	5	24
	2691862	2692767		+	-	PA2409	5	19
	2692764	2693681	2693692	+	-	PA2410	11	38
	2694545	2693781		-	-	PA2411	3	17
2694841	2694764	2694546		-	-	PA2412	5	30
	2696251	2694842		-	pvdH	PA2413	1	5
	2697749	2696430		-	-	PA2414	2	3
	2698167	2697742		-	-	PA2415	5	3
	2698526	2700163		+	treA	PA2416	3	3
	2701105	2700167		-	-	PA2417	15	16
	2701206	2702066		+	-	PA2418	4	3
	2702164	2702844		+	-	PA2419	9	7
	2702926	2704344		+	-	PA2420	3	3
	2704377	2705297		+	-	PA2421	4	3
	2705773	2706087		+	-	PA2422	7	8
	2706387	2707181		+	-	PA2423	37	35
	2720694	2707666		-	pvdL	PA2424	2	3
	2721531	2720767		-	pvdG	PA2425	1	2
2722109	2722175	2722738	2722754	+	pvdS	PA2426	7	19
	2723222	2722755		-	-	PA2427	2	3
	2724223	2723309		-	-	PA2428	5	4
	2724485	2724730		+	-	PA2429	5	6
	2725771	2724767		-	-	PA2430	5	5
	2728141	2725967		-	-	PA2431	5	4
	2728542	2729456		+	bexR	PA2432	15	12

	2729571	2729840		+	-	PA2433	14	5
	2730521	2729976		-	-	PA2434	9	4
	2732503	2730518		-	-	PA2435	15	7
2733025	2732966	2732532	2732504	-	-	PA2436	93	28
	2734160	2733120		-	-	PA2437	4	3
	2735182	2734157		-	-	PA2438	5	4
	2737194	2735194		-	-	PA2439	3	3
	2737882	2738844		+	-	PA2440	7	27
	2738841	2739716	2739716	+	-	PA2441	23	140
2740910	2740883	2739762	2739717	-	gcvT2	PA2442	50	305
2742353	2742353	2740977	2740930	-	sdaA	PA2443	27	176
2743799	2743657	2742401	2742354	-	glyA2	PA2444	42	217
2746679	2746679	2743800	2743800	-	gcvP2	PA2445	121	687
2747101	2747073	2746690	2746680	-	gcvH2	PA2446	151	968
	2748255	2747332		-	-	PA2447	6	5
	2748378	2750120		+	-	PA2448	8	8
2751666	2751663	2750128	2750121	-	-	PA2449	55	70
2752781	2752662	2751745	2751685	-	-	PA2450	33	42
	2753465	2752866		-	-	PA2451	5	7
	2754445	2753519		-	-	PA2452	3	3
2754591	2754602	2754823	2754826	+	-	PA2453	439	267
2754831	2754877	2755728		+	-	PA2454	74	54
	2755725	2756252	2756252	+	-	PA2455	63	48
2756281	2756309	2756650	2756869	+	-	PA2456	114	81
	2756918	2757898		+	-	PA2457	19	18
2757960	2758061	2759182	2759446	+	-	PA2458	35	50
	2760087	2759482		-	-	PA2459	27	44
2760794	2760619	2760323	2760088	-	-	PA2460	45	59
2761357	2761351	2760872	2760852	-	-	PA2461	75	134
2778804	2778804	2761921	2761358	-	-	PA2462	47	79
2780667	2780614	2778917	2778805	-	-	PA2463	43	60
2781460	2781451	2780927	2780696	-	-	PA2464	57	49

	2782646	2781498		-	-	PA2465	0	1
	2785226	2782764		-	foxA	PA2466	4	4
	2786356	2785370		-	foxR	PA2467	15	12
2786928	2786883	2786365	2786357	-	foxI	PA2468	33	35
	2787886	2786972		-	-	PA2469	16	11
	2788011	2789072		+	gtdA	PA2470	2	1
	2789085	2789783		+	-	PA2471	3	2
	2789861	2791207		+	-	PA2472	0	0
	2791220	2791864		+	-	PA2473	2	2
	2791906	2792817		+	-	PA2474	1	2
	2794133	2792799		-	-	PA2475	5	6
	2794976	2794206		-	dsbG	PA2476	31	25
	2795809	2794973		-	-	PA2477	11	7
	2797572	2795809		-	-	PA2478	12	12
	2797735	2798415		+	-	PA2479	40	28
	2798412	2799734	2799734	+	-	PA2480	64	41
	2800785	2799910		-	-	PA2481	153	81
2801447	2801435	2800782		-	-	PA2482	212	95
2802551	2802551	2801550	2801516	-	-	PA2483	89	69
2803342	2803316	2802702	2802640	-	-	PA2484	55	53
	2803345	2803623		+	-	PA2485	16	17
	2803640	2803834		+	-	PA2486	14	13
	2804132	2803860		-	-	PA2487	8	5
	2804230	2804994		+	-	PA2488	12	12
	2805021	2805836		+	-	PA2489	16	10
	2805917	2806291		+	-	PA2490	13	8
2807376	2807369	2806350	2806337	-	-	PA2491	87	76
2807450	2807469	2808512	2808525	+	mexT	PA2492	34	33
	2808743	2809987		+	mexE	PA2493	1	1
	2810009	2813197		+	mexF	PA2494	4	3
	2813194	2814612		+	oprN	PA2495	6	6
	2815282	2814767		-	-	PA2496	16	16

	2816219	2815341		-	-	PA2497	12	11
	2816347	2816979		+	-	PA2498	6	3
	2816997	2817452		+	-	PA2499	5	5
	2817449	2818675	2818675	+	-	PA2500	23	24
2818886	2818886	2818719	2818676	-	-	PA2501	222	156
	2820489	2818990	2818887	-	-	PA2502	26	37
	2820448	2821701	2821704	+	-	PA2503	40	36
	2822322	2821705		-	-	PA2504	11	12
	2823920	2822574		-	opdT	PA2505	3	2
	2824283	2824501		+	-	PA2506	1	2
	2825591	2824659		-	catA	PA2507	1	2
	2825926	2825636		-	catC	PA2508	1	2
	2827079	2825958		-	catB	PA2509	1	1
	2827241	2828113		+	catR	PA2510	12	7
	2829123	2828122		-	-	PA2511	6	7
	2829440	2830834		+	antA	PA2512	4	3
	2830831	2831322		+	antB	PA2513	3	2
	2831341	2832363		+	antC	PA2514	2	1
	2833130	2832369		-	xylL	PA2515	2	1
	2834168	2833155		-	xylZ	PA2516	1	1
	2834690	2834202		-	xylY	PA2517	0	0
	2836054	2834687		-	xylX	PA2518	1	0
	2837127	2836171		-	xylS	PA2519	7	4
	2840489	2837334		-	czcA	PA2520	3	2
	2841966	2840512		-	czcB	PA2521	2	2
	2843305	2842019		-	czcC	PA2522	0	0
	2843818	2844492		+	-	PA2523	10	6
	2844489	2845907		+	-	PA2524	13	9
	2847779	2846283		-	-	PA2525	13	21
	2850886	2847776		-	-	PA2526	11	18
	2854014	2850883		-	-	PA2527	20	22
2855325	2855291	2854011		-	-	PA2528	30	35

2855546	2855548	2856981		+	-	PA2529	67	62
	2856981	2858306	2858314	+	-	PA2530	55	43
	2858503	2859627		+	-	PA2531	9	7
2860280	2860274	2859777	2859683	-	tpx	PA2532	195	160
2861831	2861758	2860409	2860323	-	-	PA2533	45	95
	2862825	2861914		-	-	PA2534	19	13
	2862944	2863939		+	-	PA2535	24	27
2865106	2865105	2864170	2864073	-	-	PA2536	82	118
2865736	2865736	2865107	2865107	-	-	PA2537	66	103
	2866200	2865748	2865737	-	-	PA2538	32	58
2867506	2867506	2866193		-	-	PA2539	29	44
2869391	2869302	2867542	2867507	-	-	PA2540	46	64
2870049	2870027	2869392	2869392	-	-	PA2541	81	106
	2873827	2870162		-	-	PA2542	21	24
2875659	2875563	2873824		-	-	PA2543	34	38
	2876410	2875697		-	-	PA2544	18	14
2876550	2876555	2877367	2877373	+	xthA	PA2545	160	116
	2877908	2877477		-	-	PA2546	25	25
	2877999	2878916		+	-	PA2547	10	6
	2878976	2880370		+	-	PA2548	4	3
	2880520	2881563		+	-	PA2549	23	14
	2882983	2881754		-	-	PA2550	14	12
2882984	2883157	2884089	2884188	+	-	PA2551	58	69
2885361	2885333	2884206	2884206	-	-	PA2552	17	164
2886569	2886552	2885362	2885362	-	-	PA2553	10	187
2887356	2887337	2886570	2886570	-	-	PA2554	9	132
2889045	2889024	2887357	2887357	-	-	PA2555	6	88
	2890287	2889202		-	-	PA2556	10	15
2892143	2892050	2890356	2890288	-	-	PA2557	9	30
2892248	2892271	2892963	2893285	+	-	PA2558	35	35
2893905	2893828	2893286	2893286	-	-	PA2559	66	58
2894754	2894739	2894452	2894303	-	-	PA2560	56	141

	2894908	2896614		+	-	PA2561	14	16
	2897253	2896741		-	-	PA2562	42	31
	2897610	2899097		+	-	PA2563	9	10
	2899934	2899107		-	-	PA2564	8	7
	2900349	2899924		-	-	PA2565	4	5
	2901559	2900372		-	-	PA2566	4	6
	2902218	2903981		+	-	PA2567	28	24
2904447	2904447	2904040		-	-	PA2568	40	31
	2904907	2904566		-	-	PA2569	35	22
	2905550	2905182		-	lecA	PA2570	2	3
2906051			2905965	-	-	PA2570.1	164	150
	2907578	2906166		-	-	PA2571	7	4
	2907656	2908999		+	-	PA2572	10	4
	2910617	2909010		-	-	PA2573	16	9
	2911877	2910729		-	alkB1	PA2574	6	5
2912222	2912225	2912827	2912836	+	-	PA2575	180	133
	2913789	2912890		-	-	PA2576	18	16
2913828	2913922	2914359	2914359	+	-	PA2577	62	30
2914360	2914374	2914934	2914965	+	-	PA2578	30	31
2915904	2915832	2914966	2914966	-	kynA	PA2579	199	283
	2916748	2916158		-	-	PA2580	17	21
2917150	2917172	2918212	2918212	+	-	PA2581	156	144
2918676			2918603	-	-	PA2581.1	190	461
2919505	2919500	2918967	2918967	-	-	PA2582	311	258
	2922569	2919591		-	-	PA2583	33	33
2923297			2923222	-	-	PA2583.1	78	155
2923927	2923927	2923367	2923316	-	pgsA	PA2584	124	179
	2925787	2923961	2923928	-	uvrC	PA2585	48	57
2926567	2926432	2925788		-	gacA	PA2586	166	177
2927970	2927921	2926773	2926570	-	pqsH	PA2587	130	93
	2928539	2929567		+	-	PA2588	38	23
	2930797	2929583		-	-	PA2589	6	5

	2933462	2930808		-	-	PA2590	3	2
2934398	2934388	2933582	2933463	-	-	PA2591	81	74
2934528	2934541	2935644	2935701	+	-	PA2592	66	59
	2935851	2936423		+	qteE	PA2593	13	10
	2937449	2936490		-	-	PA2594	15	12
	2938541	2937585		-	-	PA2595	9	7
	2939539	2938553		-	-	PA2596	4	5
	2940797	2939646		-	-	PA2597	5	3
	2941884	2940802		-	-	PA2598	7	7
	2942838	2941894		-	-	PA2599	9	6
	2943915	2942848		-	-	PA2600	6	4
	2945151	2944255		-	-	PA2601	19	11
	2945264	2945869		+	-	PA2602	12	6
	2945866	2947449		+	-	PA2603	21	18
2947672			2947583	-	-	PA2603.1	53	48
2947776	2947803	2948471	2948482	+	-	PA2604	344	262
2948576	2948582	2948977		+	-	PA2605	76	53
	2948974	2949333		+	-	PA2606	69	48
	2949333	2949638		+	-	PA2607	55	53
	2949635	2949970		+	-	PA2608	60	51
	2949967	2950950	2950973	+	-	PA2609	56	55
2950974	2951038	2952012	2952012	+	-	PA2610	35	31
2953414	2953414	2952017	2952013	-	cysG	PA2611	129	139
2954696	2954696	2953416	2953415	-	serS	PA2612	278	274
2956143	2956143	2954818	2954707	-	-	PA2613	116	137
2956779	2956779	2956153	2956144	-	lolA	PA2614	208	239
2959397	2959240	2956805	2956780	-	ftsK	PA2615	150	150
2959420	2959468	2960418	2960418	+	trxB1	PA2616	425	320
2960419	2960456	2961136	2961190	+	aat	PA2617	65	77
2961191	2961191	2961898	2961963	+	-	PA2618	35	37
2961967	2962003	2962221	2962248	+	infA	PA2619	873	1484
2964601	2964579	2962303	2962293	-	clpA	PA2620	252	255

2965073	2964843	2964607	2964602	-	-	PA2621	501	466
2965115			2965151	?	-	predicted RN	270	204
2965152	2965201	2965473	2965485	+	cspD	PA2622	255	140
2966826	2966802	2965546	2965527	-	icd	PA2623	1826	1192
2966954	2967161	2969386	2969405	+	idh	PA2624	994	541
	2969473	2969943		+	-	PA2625	39	30
2969978	2969987	2971114		+	trmU	PA2626	218	227
	2971111	2971731	2971733	+	-	PA2627	154	160
	2971734	2972624		+	-	PA2628	26	22
2972677	2972699	2974069	2974070	+	purB	PA2629	268	274
2974075	2974129	2975298		+	-	PA2630	127	120
	2975291	2975716	2975716	+	-	PA2631	175	156
2975717	2975723	2976343		+	-	PA2632	51	37
	2976340	2977161		+	-	PA2633	34	22
2977378	2977756	2979351	2979351	+	aceA	PA2634	262	226
	2979530	2981548		+	-	PA2635	5	3
	2981627	2982181		+	-	PA2636	6	4
2982699	2982781	2983194	2983195	+	nuoA	PA2637	428	469
2983196	2983205	2983882	2983882	+	nuoB	PA2638	770	717
2983883	2983963	2985744	2985744	+	nuoD	PA2639	393	356
2985745	2985746	2986246		+	nuoE	PA2640	407	379
	2986243	2987589	2987589	+	nuoF	PA2641	381	384
2987696			2987706	?	-	predicted RN	393	379
2987707	2987721	2990438		+	nuoG	PA2642	388	374
	2990435	2991430	2991441	+	nuoH	PA2643	349	350
2991442	2991442	2991990	2991991	+	nuoI	PA2644	347	378
2991995	2992002	2992502	2992502	+	nuoJ	PA2645	288	335
2992503	2992548	2992856		+	nuoK	PA2646	265	280
	2992853	2994700	2994700	+	nuoL	PA2647	267	310
2994701	2994728	2996257	2996257	+	nuoM	PA2648	242	266
2996258	2996265	2997725	2997732	+	nuoN	PA2649	190	237
	2997840	2998649		+	-	PA2650	18	19

2999923	2999914	2998856	2998844	-	-	PA2651	86	49
3001872	3001759	3000074	3000074	-	-	PA2652	88	56
3003231	3003198	3001873	3001873	-	-	PA2653	92	75
3003379	3003457	3005601	3005605	+	-	PA2654	61	41
3005914	3005914	3005606	3005606	-	-	PA2655	28	22
	3007342	3006005		-	-	PA2656	24	27
	3008010	3007339		-	-	PA2657	55	41
	3008324	3008010		-	-	PA2658	128	86
3008676	3008632	3008324		-	-	PA2659	193	129
3008842	3008847	3009929	3009951	+	-	PA2660	60	50
3009956	3009956	3010849	3011099	+	-	PA2661	37	35
	3012293	3011100		-	-	PA2662	12	9
	3012537	3012280		-	ppyR	PA2663	14	8
	3013773	3012592		-	fhp	PA2664	4	2
	3013928	3015481		+	-	PA2665	19	17
3015553	3015582	3015938	3016021	+	-	PA2666	64	71
3016205	3016246	3016599	3016604	+	-	PA2667	801	654
	3016884	3016675		-	-	PA2668	51	40
	3017818	3017234		-	-	PA2669	6	5
	3018804	3017818		-	-	PA2670	8	5
	3019927	3018848		-	-	PA2671	3	4
	3020507	3019917		-	-	PA2672	2	1
	3020929	3020504		-	-	PA2673	5	1
	3021339	3020929		-	-	PA2674	6	4
	3021742	3021308		-	-	PA2675	5	2
	3022983	3021796		-	-	PA2676	3	2
	3024715	3022988		-	-	PA2677	5	3
	3025508	3024705		-	-	PA2678	13	4
	3026852	3026100		-	-	PA2679	61	7
	3027078	3028052		+	-	PA2680	5	4
	3028075	3029004		+	-	PA2681	12	7
	3029187	3030428		+	-	PA2682	11	8

	3031398	3030436		-	-	PA2683	25	22
3035751	3035702	3031749	3031515	-	-	PA2684	50	44
	3037932	3035752	3035752	-	-	PA2685	81	66
	3037886	3038803		+	pfeR	PA2686	24	22
	3038803	3040143		+	pfeS	PA2687	12	10
	3040242	3042482		+	pfeA	PA2688	3	5
	3042502	3043416		+	-	PA2689	7	4
	3044766	3043750		-	-	PA2690	18	20
	3046152	3044947		-	-	PA2691	10	5
3046368	3046421	3046945	3047115	+	-	PA2692	41	38
	3047116	3047616		+	-	PA2693	20	20
	3047970	3047644		-	-	PA2694	19	21
	3049154	3048051		-	-	PA2695	25	24
	3050161	3049268		-	-	PA2696	7	3
	3050612	3050331		-	-	PA2697	9	4
	3051349	3050669		-	-	PA2698	15	12
	3051957	3053795		+	-	PA2699	1	1
	3053844	3055151		+	opdB	PA2700	3	1
	3055154	3056743		+	-	PA2701	3	2
	3056890	3057366	3057366	+	-	PA2702	21	19
	3057376	3057609		+	-	PA2703	25	16
	3058921	3057902		-	-	PA2704	12	8
3060264	3060216	3059035	3058924	-	-	PA2705	50	28
	3060660	3060265		-	-	PA2706	43	30
3061610	3061570	3060725	3060661	-	-	PA2707	121	66
	3062818	3061733		-	-	PA2708	5	4
3062928	3062939	3063913	3063971	+	cysK	PA2709	102	53
	3063972	3064586		+	-	PA2710	39	29
	3064785	3065876		+	-	PA2711	9	7
	3066296	3067159		+	-	PA2712	15	42
3067686	3067686	3067207		-	-	PA2713	34	28
	3068040	3070349		+	-	PA2714	7	9

	3070366	3070704		+	-	PA2715	10	6
	3071954	3070719		-	-	PA2716	5	4
	3072911	3072081		-	cpo	PA2717	6	2
	3073144	3073632		+	-	PA2718	25	13
	3073732	3074418		+	-	PA2719	7	6
3075276	3075218	3074580	3074419	-	-	PA2720	49	46
	3075411	3075890		+	-	PA2721	3	3
	3075922	3076314		+	-	PA2722	4	3
	3076344	3076622		+	-	PA2723	37	21
	3076697	3077236		+	-	PA2724	5	5
3077259	3077312	3079195	3079196	+	-	PA2725	43	33
3079197	3079197	3079835		+	-	PA2726	25	19
3079850	3079850	3083482	3083482	+	-	PA2727	22	18
	3083485	3086145		+	-	PA2728	19	14
	3086142	3087491		+	-	PA2729	14	10
3087767			3087988	?	-	predicted RN	319	609
3089636	3089613	3088660	3088551	-	-	PA2730	178	166
3090220	3090108	3089644	3089637	-	-	PA2731	206	208
	3093661	3090221	3090221	-	-	PA2732	163	159
3094667	3094185	3093658		-	-	PA2733	233	218
3096052	3096052	3094757	3094757	-	-	PA2734	295	275
3096053			3096065	?	-	predicted RN	387	362
3098508	3098508	3096130	3096117	-	-	PA2735	313	272
3099208	3098979	3098626	3098514	-	-	PA2736	159	191
3099382			3099306	-	-	PA2736.1	182	419
3099733	3099730	3099473	3099383	-	-	PA2737	484	366
3099734			3099781	?	-	predicted RN	1010	653
3100115	3100112	3099810	3099782	-	himA	PA2738	1376	946
3102503	3102494	3100116	3100116	-	pheT	PA2739	293	255
3103641	3103545	3102529	3102504	-	pheS	PA2740	393	350
3104015	3103999	3103643	3103642	-	rplT	PA2741	11743	10596
3104278	3104217	3104023	3104023	-	rpmI	PA2742	13864	12585

	3104830	3104279	3104279	-	infC	PA2743	6409	6355
3106918	3106752	3104830		-	thrS	PA2744	657	602
3106994			3106919	-	-	PA2744.1	48	35
3107937	3107865	3107002	3106995	-	-	PA2745	37	33
	3108057	3108383		+	-	PA2746	13	8
3109742	3109257	3108970	3108462	-	-	PA2747	29	33
	3110525	3109743		-	-	PA2748	30	22
	3110901	3111614	3111614	+	endA	PA2749	29	30
3111615	3111633	3112151	3112630	+	-	PA2750	39	35
	3112878	3113777		+	-	PA2751	6	4
	3114683	3114240		-	-	PA2752	12	14
3114792	3114819	3115193	3115303	+	-	PA2753	129	41
	3115304	3115633		+	-	PA2754	54	40
3116647	3116654	3117124	3117126	+	eco	PA2755	267	146
3117641	3117611	3117177	3117169	-	-	PA2756	131	93
3118209	3118193	3117750	3117671	-	-	PA2757	61	47
	3118296	3119183		+	-	PA2758	19	9
3119917	3119708	3119394	3119322	-	-	PA2759	176	28
3119959	3120073	3121350	3121350	+	-	PA2760	1462	1841
3121351			3121366	?	-	predicted RN	437	614
3121396	3121462	3121878	3121920	+	-	PA2761	95	49
	3122265	3121921		-	-	PA2762	52	27
	3122586	3122377		-	-	PA2763	12	7
3123490	3123599	3124294	3124341	+	-	PA2764	57	42
3125306	3125241	3124342	3124342	-	-	PA2765	194	169
	3126164	3125583		-	-	PA2766	24	17
	3126252	3127220		+	-	PA2767	10	8
	3127226	3127708		+	-	PA2768	25	21
3128270	3128270	3127860	3127709	-	-	PA2769	69	50
3129078	3129071	3128292	3128271	-	-	PA2770	127	78
3129417	3129729	3130754	3130754	+	-	PA2771	42	45
3131508	3131159	3130761	3130759	-	-	PA2772	46	61

3132228	3132030	3131509	3131509	-	-	PA2773	40	43
3132816	3132816	3132229	3132229	-	-	PA2774	91	83
3133269	3133258	3132821	3132817	-	-	PA2775	115	121
3133525			3133449	-	-	PA2775.1	6	7
3133698	3133710	3134993	3135043	+	-	PA2776	90	31
	3136000	3135044		-	-	PA2777	14	13
	3136963	3136082		-	-	PA2778	23	30
3137495	3137442	3137044	3136964	-	-	PA2779	46	64
	3137850	3138194		+	-	PA2780	16	6
	3138191	3138532		+	-	PA2781	22	10
	3139011	3139670		+	-	PA2782	6	1
	3139748	3141547		+	-	PA2783	6	1
	3141719	3142276		+	-	PA2784	7	3
	3142285	3142503		+	-	PA2785	8	4
	3142618	3143085		+	-	PA2786	2	3
	3143152	3144390		+	cpg2	PA2787	4	2
	3146007	3144412		-	-	PA2788	10	5
	3146251	3147330		+	-	PA2789	14	13
	3147765	3148250		+	-	PA2790	27	16
	3148629	3148339		-	-	PA2791	20	18
3149399	3149319	3148723	3148630	-	-	PA2792	41	39
3150447	3150434	3149400	3149400	-	-	PA2793	88	89
	3152202	3150886		-	-	PA2794	16	11
3152490	3152504	3153502	3153502	+	-	PA2795	68	68
3153503	3153582	3154505	3154592	+	tal	PA2796	111	65
	3155075	3154593	3154593	-	-	PA2797	247	297
3156298	3156256	3155072		-	-	PA2798	319	417
3156319	3156503	3156802	3156859	+	-	PA2799	51	40
3157564	3157564	3156860	3156860	-	-	PA2800	473	438
3157609	3157667	3158071	3158071	+	-	PA2801	76	56
3158803	3158792	3158073	3158072	-	-	PA2802	84	69
	3158926	3159669		+	-	PA2803	4	2

	3159666	3160247		+	-	PA2804	4	2
3160293	3160321	3160584	3160584	+	-	PA2805	248	117
3161535	3161481	3160651	3160634	-	-	PA2806	55	65
	3162216	3161599		-	-	PA2807	3	0
	3162579	3162388		-	ptrA	PA2808	11	17
	3162705	3163385		+	copR	PA2809	16	12
	3163382	3164713		+	copS	PA2810	14	16
	3165542	3164763	3164752	-	-	PA2811	86	80
3166471	3166471	3165539		-	-	PA2812	111	101
3167281	3167167	3166547	3166472	-	-	PA2813	43	32
	3167953	3167282		-	-	PA2814	9	3
	3168106	3170553		+	-	PA2815	15	8
	3170682	3171062		+	-	PA2816	25	13
3171097	3171121	3171531	3171539	+	-	PA2817	94	82
	3173175	3171598		-	arr	PA2818	26	25
3173244	3173244	3173723		+	-	PA2819	143	285
3173674			3173599	-	-	PA2819.1	271	602
3173834			3173759	-	-	PA2819.2	881	2122
3173912			3173837	-	-	PA2819.3	940	2334
3174090	3174131	3174904	3174965	+	-	PA2820	68	48
3174966	3174966	3175628	3175635	+	-	PA2821	66	50
	3176120	3175638	3175636	-	-	PA2822	94	74
3177089	3177007	3176117		-	-	PA2823	108	84
	3177090	3179450		+	-	PA2824	27	18
	3179960	3179469	3179451	-	ospR	PA2825	50	40
3180553	3180442	3179957		-	-	PA2826	60	37
3180978	3180952	3180554	3180554	-	-	PA2827	98	58
3181083	3181137	3182348	3182348	+	-	PA2828	153	169
3182349	3182400	3182852	3182972	+	-	PA2829	41	51
3182978	3182986	3183861	3183871	+	htpX	PA2830	278	277
3183997	3184002	3185129	3185129	+	-	PA2831	75	40
3185834	3185817	3185161	3185130	-	tpm	PA2832	57	31

	3186319	3185873		-	-	PA2833	9	8
	3186449	3187408		+	-	PA2834	24	17
	3187546	3189138		+	-	PA2835	2	1
	3189150	3190214		+	-	PA2836	2	2
	3190211	3191650		+	-	PA2837	2	1
	3191687	3192658		+	-	PA2838	2	3
	3192749	3193519		+	-	PA2839	6	7
3195746	3195589	3193886	3193881	-	-	PA2840	136	73
	3195900	3196691		+	-	PA2841	28	16
	3197466	3196717	3196717	-	-	PA2842	35	24
3197620	3197642	3198988	3199028	+	-	PA2843	49	38
	3200237	3199029		-	-	PA2844	18	8
	3200553	3200320		-	-	PA2845	8	5
	3200650	3201504		+	-	PA2846	17	9
	3202255	3201506		-	-	PA2847	18	7
	3202293	3203312		+	-	PA2848	9	4
3203380	3203398	3203853	3203966	+	ohrR	PA2849	77	55
3203997	3203998	3204426	3204463	+	ohr	PA2850	74	74
3205102	3205080	3204514	3204502	-	efp	PA2851	2564	2141
	3206253	3205123		-	-	PA2852	22	22
3206408			3206497	+	-	PA2852.1	20	43
3206852	3206915	3207166	3207195	+	oprI	PA2853	38225	36090
3208273	3208261	3207290	3207278	-	-	PA2854	259	246
3208617	3208617	3208339	3208277	-	-	PA2855	78	76
3209288	3209278	3208673	3208644	-	tesA	PA2856	78	50
	3209289	3209972		+	-	PA2857	30	25
	3209981	3212473		+	-	PA2858	19	14
3212474	3212525	3213031	3213043	+	greB	PA2859	55	57
3213484	3213484	3213044	3213044	-	-	PA2860	148	136
	3213538	3214086		+	ligT	PA2861	3	5
	3214282	3215217		+	lipA	PA2862	10	3
	3215423	3216289		+	lipH	PA2863	2	1

3216903	3216890	3216456	3216451	-	-	PA2864	66	41
	3218419	3216989		-	-	PA2865	33	25
3218420	3218666	3219469	3219580	+	mttC	PA2866	26	27
3219581	3219608	3221080	3221080	+	-	PA2867	198	194
	3221206	3221565		+	-	PA2868	27	33
	3221652	3222140		+	-	PA2869	14	10
	3222150	3223727		+	-	PA2870	16	10
3224540	3224533	3223736	3223728	-	-	PA2871	54	39
	3225384	3224614		-	-	PA2872	23	14
	3227387	3225381		-	-	PA2873	21	9
	3228337	3227384		-	-	PA2874	24	11
3229483	3229254	3228337		-	-	PA2875	60	38
3230193	3230182	3229484	3229484	-	pyrF	PA2876	80	79
	3231172	3230279		-	-	PA2877	17	13
	3231218	3231859		+	-	PA2878	10	11
	3232772	3231882		-	-	PA2879	28	16
	3232819	3233334		+	-	PA2880	12	8
	3234254	3233343		-	-	PA2881	8	4
	3235371	3234256		-	-	PA2882	4	2
3235787	3235794	3235961	3236024	+	-	PA2883	105	57
3236194	3236194	3236958		+	-	PA2884	55	57
3237683	3237555	3236959		-	atuR	PA2885	105	72
	3237836	3239638		+	atuA	PA2886	10	11
	3239711	3240589		+	atuB	PA2887	8	14
	3240591	3242207		+	atuC	PA2888	6	10
	3242324	3243484		+	atuD	PA2889	10	14
	3243505	3244299		+	atuE	PA2890	4	7
	3244345	3246330		+	atuF	PA2891	5	7
	3246382	3247206		+	atuG	PA2892	4	7
	3247369	3249195		+	atuH	PA2893	10	11
3249974	3249631	3249209	3249196	-	-	PA2894	53	44
	3250741	3249977	3249977	-	-	PA2895	27	31

3251650	3251322	3250738		-	-	PA2896	37	30
3253102	3253090	3251651	3251651	-	-	PA2897	107	101
	3253680	3253312		-	-	PA2898	4	3
	3254404	3253766		-	-	PA2899	24	17
	3255407	3254598	3254596	-	-	PA2900	123	116
	3255766	3255407		-	-	PA2901	220	196
3256620	3256611	3255763		-	-	PA2902	94	94
	3258360	3256681	3256626	-	cobJ	PA2903	50	56
	3259105	3258353		-	cobI	PA2904	69	63
	3259728	3259102		-	cobH	PA2905	43	49
3261413	3261185	3259725		-	-	PA2906	57	69
	3261522	3262769		+	cobL	PA2907	27	22
	3262762	3263862		+	cbiD	PA2908	24	21
	3263859	3264587		+	-	PA2909	16	12
	3265211	3264642		-	-	PA2910	6	4
	3265848	3268004		+	-	PA2911	16	17
	3268057	3268830		+	-	PA2912	9	10
	3268827	3269798		+	-	PA2913	7	6
	3269795	3270826		+	-	PA2914	8	8
	3271693	3270827		-	-	PA2915	21	12
	3272406	3271813		-	-	PA2916	1	2
	3273293	3272457		-	-	PA2917	12	18
	3273504	3274277		+	-	PA2918	12	7
	3274328	3274585		+	-	PA2919	3	2
	3276234	3274597		-	-	PA2920	13	8
	3277308	3276319		-	-	PA2921	23	16
	3277409	3278578		+	-	PA2922	1	1
	3278638	3279423		+	hisJ	PA2923	1	1
	3279479	3280168		+	hisQ	PA2924	1	1
	3280165	3280878		+	hisM	PA2925	5	4
	3280899	3281666		+	hisP	PA2926	5	3
	3281988	3283319		+	-	PA2927	11	11

	3284598	3283375		-	-	PA2928	18	15
	3285621	3285007		-	-	PA2929	7	7
3285668	3285687	3286613	3286623	+	-	PA2930	50	36
	3287214	3286624	3286624	-	cifR	PA2931	27	34
	3287340	3288449		+	morB	PA2932	8	4
	3288515	3289693		+	-	PA2933	1	2
	3289715	3290674		+	cif	PA2934	8	3
	3291173	3290694		-	-	PA2935	2	2
	3291283	3291864		+	-	PA2936	3	3
	3291960	3292268		+	-	PA2937	2	1
	3293781	3292324		-	-	PA2938	5	3
	3294282	3295892		+	-	PA2939	5	5
	3297118	3295979		-	-	PA2940	6	6
	3297884	3297240		-	-	PA2941	22	23
3298933	3298921	3297905	3297885	-	-	PA2942	54	41
3299022			3299269	+	-	PA2942.1	37	32
	3299492	3300586	3300586	+	-	PA2943	25	27
	3304361	3300615		-	cobN	PA2944	34	28
3305705	3305564	3304437	3304362	-	-	PA2945	87	70
3305899	3305919	3307013	3307084	+	-	PA2946	89	57
	3307085	3307513		+	-	PA2947	30	27
	3307510	3308262		+	cobM	PA2948	22	14
	3308391	3309338		+	-	PA2949	28	24
3310637	3310607	3309411	3309375	-	-	PA2950	656	772
	3311721	3310792	3310774	-	etfA	PA2951	1264	909
3312660	3312470	3311721		-	etfB	PA2952	1430	1075
3312736	3312791	3314446	3314447	+	-	PA2953	307	257
	3315064	3314495	3314473	-	-	PA2954	41	36
	3315689	3315057		-	-	PA2955	45	47
3316799	3316650	3315754	3315690	-	-	PA2956	70	66
3317460	3317438	3316800	3316800	-	-	PA2957	231	281
	3317524	3318657		+	-	PA2958	34	28

3318663			3318859	+	rgsA	PA2958.1	164	136
3319673	3319659	3318883	3318860	-	-	PA2959	149	123
3320032	3320030	3319674	3319674	-	pilZ	PA2960	252	232
	3321050	3320064	3320040	-	holB	PA2961	108	92
3321701	3321675	3321043		-	tmk	PA2962	75	69
3322758	3322753	3321704	3321704	-	-	PA2963	66	68
	3323574	3322759	3322759	-	pabC	PA2964	191	167
3324946	3324818	3323574		-	fabF1	PA2965	562	529
3325214	3325183	3324947	3324947	-	acpP	PA2966	11996	10982
3325215			3325254	?	-	predicted RN	6204	6770
3326122	3326122	3325379	3325255	-	fabG	PA2967	1481	1612
3327091	3327083	3326145	3326124	-	fabD	PA2968	538	743
3328202	3328169	3327189	3327101	-	plsX	PA2969	221	247
3328398	3328385	3328203	3328203	-	rpmF	PA2970	6703	6321
3328935	3328935	3328399	3328399	-	-	PA2971	1814	2661
3328936	3329045	3329623	3329635	+	-	PA2972	47	46
	3330670	3329690	3329690	-	-	PA2973	238	270
	3331355	3330663		-	-	PA2974	153	160
3332304	3332304	3331348		-	rluC	PA2975	167	188
3332377	3332881	3336054	3336060	+	rne	PA2976	440	316
	3337231	3336212	3336212	-	murB	PA2977	122	122
	3337692	3337228		-	ptpA	PA2978	173	177
	3338456	3337692		-	kdsB	PA2979	155	176
3338678	3338641	3338456		-	-	PA2980	240	238
	3339677	3338679	3338679	-	lpxK	PA2981	115	95
	3340117	3339677		-	-	PA2982	240	161
3340749	3340749	3340114		-	-	PA2983	181	160
	3343047	3340822		-	-	PA2984	6	4
	3343178	3343711		+	-	PA2985	37	22
3345100	3345100	3343799	3343757	-	-	PA2986	63	66
	3345796	3345113	3345101	-	-	PA2987	114	116
	3347039	3345789		-	-	PA2988	59	74

	3346977	3347741	3348066	+	-	PA2989	41	45
3348073	3348073	3348795	3348795	+	-	PA2990	156	84
3350267	3350232	3348838	3348796	-	sth	PA2991	410	232
	3350638	3350411	3350300	-	-	PA2992	111	97
	3351663	3350635		-	-	PA2993	100	98
3352879	3352879	3351656		-	nqrF	PA2994	224	214
	3353498	3352890	3352880	-	nqrE	PA2995	275	249
	3354172	3353498		-	nqrD	PA2996	303	273
	3354954	3354169		-	nqrC	PA2997	232	190
3356158	3356158	3354947		-	nqrB	PA2998	302	259
3357554	3357499	3356162	3356159	-	nqrA	PA2999	349	309
3359268	3359167	3357758	3357741	-	aroP1	PA3000	124	103
3360718	3360654	3359269	3359269	-	-	PA3001	1591	1475
3360719			3360731	?	-	predicted RN	848	885
3360800	3360875	3364321	3364321	+	mfd	PA3002	105	112
3364322	3364332	3364961	3364961	+	-	PA3003	127	134
3365746	3365744	3365007	3365000	-	-	PA3004	129	106
3366782	3366754	3365756	3365756	-	nagZ	PA3005	105	94
3367681	3367671	3366970	3366969	-	psrA	PA3006	182	125
3367889	3367903	3368517	3368518	+	lexA	PA3007	271	135
3368519	3368529	3369014	3369014	+	-	PA3008	181	115
3369269	3369269	3369036	3369019	-	-	PA3009	111	149
3370099	3369993	3369475	3369450	-	-	PA3010	137	103
3372717	3372706	3370100	3370100	-	topA	PA3011	299	243
3373253	3373171	3372797	3372734	-	-	PA3012	66	38
3374459	3374429	3373254	3373254	-	foaB	PA3013	251	169
3376767	3376607	3374460	3374460	-	faoA	PA3014	269	164
	3377035	3377853		+	-	PA3015	30	16
	3378304	3377870		-	-	PA3016	20	22
3378442	3378512	3378949	3378978	+	-	PA3017	81	62
3379670	3379665	3378979	3378979	-	-	PA3018	164	184
3381742	3381737	3379815	3379815	-	-	PA3019	135	136

	3381963	3383891		+	-	PA3020	39	35
3383919	3383948	3384334	3384334	+	-	PA3021	176	157
3385189	3385184	3384378	3384341	-	-	PA3022	81	74
	3386195	3385287		-	-	PA3023	13	9
	3387829	3386270		-	-	PA3024	12	8
	3389430	3387850		-	-	PA3025	11	8
	3391031	3389436		-	-	PA3026	12	10
3391107	3391208	3392239	3392267	+	-	PA3027	62	65
	3393485	3392268	3392268	-	moeA2	PA3028	101	59
3394036	3394021	3393482		-	moaB2	PA3029	154	82
	3394088	3394684		+	mobA	PA3030	24	14
3394704	3394816	3395037	3395052	+	-	PA3031	771	490
3395190			3395266	+	-	PA3031.1	2	7
	3396728	3395325		-	snr1	PA3032	4	5
3397380	3397374	3397096	3396912	-	-	PA3033	60	51
3397944	3397938	3397381	3397381	-	-	PA3034	74	50
	3398104	3398700		+	-	PA3035	12	10
	3398717	3399649		+	-	PA3036	11	10
	3399649	3400515		+	-	PA3037	11	5
3400622	3400684	3401949	3402065	+	-	PA3038	79	35
	3403516	3402134		-	-	PA3039	4	2
3403712	3403812	3404141	3404141	+	-	PA3040	85	50
3404142	3404145	3404525	3404538	+	-	PA3041	41	35
3404539	3404539	3404862	3404862	+	-	PA3042	31	22
	3404979	3406310		+	-	PA3043	36	18
	3408546	3406321		-	-	PA3044	6	4
	3409183	3408560		-	-	PA3045	6	4
3410040	3409953	3409609	3409609	-	-	PA3046	329	249
3410068	3410264	3411694	3411762	+	-	PA3047	61	46
3414318	3413940	3411763	3411763	-	-	PA3048	74	64
3414351	3414401	3414613	3414627	+	rmf	PA3049	121	95
3415730	3415729	3414701	3414701	-	pyrD	PA3050	140	144

	3416067	3415786		-	-	PA3051	35	27
	3416061	3417041		+	-	PA3052	53	32
	3417113	3418120		+	-	PA3053	16	8
	3418288	3420192		+	-	PA3054	19	19
3420785	3420785	3420309	3420305	-	-	PA3055	78	56
3421424	3421299	3420823	3420800	-	-	PA3056	53	37
	3421425	3421655		+	-	PA3057	16	22
	3423066	3421696		-	pelG	PA3058	7	9
	3424591	3423068		-	pelF	PA3059	3	3
	3425577	3424588		-	pelE	PA3060	5	6
	3426922	3425555		-	pelD	PA3061	3	3
	3427446	3426928		-	pelC	PA3062	3	5
	3431067	3427486		-	pelB	PA3063	2	2
	3433891	3431045		-	pelA	PA3064	3	3
	3435359	3434373		-	-	PA3065	4	2
	3435944	3435372		-	-	PA3066	24	13
	3436430	3435987	3435987	-	-	PA3067	16	19
3441616	3441440	3436578	3436563	-	gdhB	PA3068	214	206
	3442330	3441713		-	-	PA3069	10	6
3442724	3442823	3443803	3443803	+	-	PA3070	115	94
3443804	3443814	3444752		+	-	PA3071	40	35
	3444749	3445243		+	-	PA3072	19	19
	3445236	3446258		+	-	PA3073	18	18
	3446255	3448015		+	-	PA3074	19	21
	3448012	3449643		+	-	PA3075	22	19
	3449697	3450776		+	-	PA3076	27	30
	3450838	3451509		+	-	PA3077	25	24
	3451506	3452801		+	-	PA3078	25	24
3455256	3455253	3452872	3452850	-	-	PA3079	30	32
3456542	3456363	3455266	3455262	-	-	PA3080	34	34
3457910	3457910	3456543	3456543	-	-	PA3081	78	33
3460056	3459933	3457969	3457945	-	gbt	PA3082	136	51

3463055	3463037	3460380	3460380	-	pepN	PA3083	141	114
3463888	3463856	3463056	3463056	-	-	PA3084	113	102
3464152	3464152	3463889	3463889	-	-	PA3085	155	152
	3465096	3464236	3464153	-	-	PA3086	54	49
	3466073	3465093		-	-	PA3087	57	59
3466981	3466960	3466073		-	-	PA3088	114	90
	3467085	3468050		+	-	PA3089	7	2
	3468108	3468965		+	-	PA3090	18	11
	3470427	3468988		-	-	PA3091	54	15
	3472803	3470764		-	fadH1	PA3092	16	8
3473015	3473018	3474136		+	-	PA3093	56	50
	3474133	3475170	3475456	+	-	PA3094	44	32
3475533			3475457	-	-	PA3094.1	239	677
3475724			3475648	-	-	PA3094.2	1112	2421
3475816			3475741	-	-	PA3094.3	826	2084
3476480	3476480	3475956		-	xcpZ	PA3095	24	19
	3477630	3476482		-	xcpY	PA3096	39	25
	3478628	3477627		-	xcpX	PA3097	31	23
	3479338	3478625		-	xcpW	PA3098	50	32
	3479724	3479335		-	xcpV	PA3099	45	28
3480241	3480239	3479721		-	xcpU	PA3100	78	48
3480696	3480692	3480246	3480242	-	xcpT	PA3101	175	104
	3481914	3480697	3480697	-	xcpS	PA3102	51	32
3483510	3483422	3481914		-	xcpR	PA3103	72	45
3483642	3483642	3484349	3484349	+	xcpP	PA3104	92	71
3484350	3484354	3486330	3486370	+	xcpQ	PA3105	76	78
	3487184	3486417	3486410	-	-	PA3106	169	195
3488392	3488392	3487181		-	metZ	PA3107	185	231
3490003	3489914	3488409	3488393	-	purF	PA3108	330	366
3490681	3490678	3490136	3490004	-	-	PA3109	80	90
3491411	3491411	3490752	3490682	-	-	PA3110	144	143
	3492704	3491415	3491412	-	folC	PA3111	268	312

3493659	3493573	3492701		-	accD	PA3112	383	528
3494515	3494477	3493842	3493840	-	trpF	PA3113	166	117
3495430	3495424	3494567	3494517	-	truA	PA3114	96	70
3498316	3498205	3495446	3495432	-	fimV	PA3115	444	259
3499485	3499384	3498374	3498341	-	-	PA3116	85	87
3500620	3500598	3499486	3499486	-	asd	PA3117	208	160
3501808	3501750	3500668	3500634	-	leuB	PA3118	65	44
	3502372	3501809		-	-	PA3119	18	24
3503291	3503280	3502642	3502373	-	leuD	PA3120	57	42
	3504716	3503292		-	leuC	PA3121	47	31
	3504879	3505772		+	-	PA3122	28	24
3506253	3506242	3505865	3505773	-	-	PA3123	60	46
	3507233	3506322		-	-	PA3124	45	37
	3507345	3508673		+	-	PA3125	12	9
3509213	3509166	3508717	3508707	-	ibpA	PA3126	248	341
	3509324	3510127		+	-	PA3127	27	16
3510144	3510144	3510890	3510890	+	-	PA3128	22	17
3511892	3511864	3510905	3510891	-	-	PA3129	76	78
	3512394	3511957	3511914	-	-	PA3130	67	37
3513096	3513041	3512394		-	-	PA3131	74	55
	3514011	3513154		-	-	PA3132	8	10
	3514565	3514023		-	-	PA3133	13	11
3514798			3514723	-	-	PA3133.1	211	423
3514920			3514845	-	-	PA3133.2	437	902
3515089			3515014	-	-	PA3133.3	1036	2084
3515213			3515138	-	-	PA3133.4	1013	2249
3516921	3516900	3515416	3515404	-	gltX	PA3134	391	327
	3517858	3516938		-	-	PA3135	21	15
	3517928	3518995		+	-	PA3136	5	6
	3519045	3520544		+	-	PA3137	14	12
3522597	3522560	3520548	3520545	-	uvrB	PA3138	86	60
3522683	3522748	3523944	3524011	+	-	PA3139	379	654

3524012			3524087	+	-	PA3139.1	58	112
	3524490	3524161		-	-	PA3140	15	16
3526774	3526678	3524681	3524675	-	wbpM	PA3141	262	230
	3527734	3527429	3527150	-	-	PA3142	118	84
3528212	3528212	3527670		-	-	PA3143	82	93
3528387	3528350	3528231	3528213	-	-	PA3144	181	220
3529506	3529447	3528428	3528405	-	wbpL	PA3145	242	320
3530458	3530458	3529508	3529508	-	wbpK	PA3146	307	388
3531750	3531708	3530467	3530459	-	wbpJ	PA3147	352	412
	3532815	3531751	3531751	-	wbpI	PA3148	474	497
3533933	3533933	3532812		-	wbpH	PA3149	359	437
3535215	3535081	3533948	3533934	-	wbpG	PA3150	382	439
	3535971	3535216	3535216	-	hisF2	PA3151	351	380
	3536579	3535971		-	hisH2	PA3152	191	239
	3537811	3536576		-	wzx	PA3153	102	129
3539124	3539124	3537808		-	wzy	PA3154	129	168
3540209	3540207	3539128	3539127	-	wbpE	PA3155	658	642
	3540785	3540210	3540210	-	wbpD	PA3156	618	639
3542729	3542671	3540782		-	-	PA3157	109	125
3543757	3543690	3542740	3542740	-	wbpB	PA3158	669	588
3545185	3545074	3543764	3543764	-	wbpA	PA3159	924	771
3545186			3545228	?	-	predicted RN	574	507
3546965	3546927	3545881	3545229	-	wzz	PA3160	209	226
3547528			3547624	?	-	predicted RN	317	190
3547998	3547973	3547689	3547625	-	himD	PA3161	361	221
3549919	3549789	3548110	3548083	-	rpsA	PA3162	9326	7340
3549920			3549944	?	-	predicted RN	4356	4546
3552978	3550746	3550057	3549945	-	cmk	PA3163	220	203
3554156	3554088	3552979	3552979	-	hisC2	PA3165	190	175
	3555254	3554157	3554157	-	pheA	PA3166	260	224
3556412	3556339	3555254		-	serC	PA3167	350	323
3559278	3559198	3556427	3556427	-	gyrA	PA3168	507	410

3560515	3560511	3559435	3559317	-	-	PA3169	128	106
3560605	3560621	3561955	3562033	+	-	PA3170	91	73
3562034	3562106	3562804		+	ubiG	PA3171	95	74
	3562801	3563481	3563545	+	-	PA3172	63	54
3563546	3563546	3564286	3564291	+	-	PA3173	86	69
	3564445	3565173		+	-	PA3174	6	4
	3565189	3566124		+	-	PA3175	6	3
	3566163	3567377		+	gltS	PA3176	4	3
	3567606	3568529		+	-	PA3177	38	24
3569027	3569013	3568636	3568602	-	-	PA3178	63	53
3570272	3570266	3569106	3569106	-	-	PA3179	210	206
3570282	3570414	3570851	3570939	+	-	PA3180	30	37
3571603	3571603	3570941	3570941	-	-	PA3181	105	94
	3572337	3571621	3571604	-	pgl	PA3182	101	71
3573799	3573793	3572324		-	zwf	PA3183	211	93
3573800			3573816	?	-	predicted RN	78	47
3573838	3573980	3574837	3574845	+	-	PA3184	77	66
3575709	3575697	3574846	3574846	-	-	PA3185	121	108
3575713			3575854	?	-	predicted RN	16	24
3577290	3577276	3575912	3575886	-	oprB	PA3186	39	199
3578512	3578480	3577320	3577297	-	-	PA3187	14	12
	3579358	3578513	3578513	-	-	PA3188	11	8
3580383	3580283	3579351		-	-	PA3189	9	5
3581665	3581646	3580384	3580384	-	-	PA3190	27	30
3583615	3583592	3582171	3581666	-	-	PA3191	21	30
3584344	3584344	3583616	3583616	-	gltR	PA3192	70	74
3585476	3585374	3584379	3584345	-	glk	PA3193	83	84
3587371	3587303	3585477	3585477	-	edd	PA3194	99	47
3587420	3587433	3588437	3588437	+	gapA	PA3195	120	103
3589271	3589271	3588741	3588438	-	-	PA3196	54	62
3590356	3590350	3589352	3589272	-	-	PA3197	48	44
3591363	3591242	3590490	3590382	-	-	PA3198	37	37

	3592044	3591415	3591415	-	-	PA3199	66	49
3593092	3592928	3592041		-	-	PA3200	53	44
3593132	3593144	3593731	3593732	+	-	PA3201	269	217
3593733	3593733	3594032	3594032	+	-	PA3202	224	201
3594098	3594208	3594570	3594580	+	-	PA3203	149	135
3594581	3594581	3595258	3595386	+	-	PA3204	64	58
3595387	3595387	3595827	3595862	+	-	PA3205	33	77
	3595942	3597279		+	-	PA3206	19	16
3597280	3597319	3597798		+	-	PA3207	57	45
3598344	3598339	3597779		-	-	PA3208	92	77
3598807	3598775	3598404	3598345	-	-	PA3209	32	51
3598915	3598933	3600387	3600453	+	trkH	PA3210	45	39
3600454	3600454	3601599		+	-	PA3211	96	72
	3601596	3602390	3602390	+	-	PA3212	50	53
3602391	3602392	3603330		+	-	PA3213	59	52
	3603327	3603971	3603993	+	-	PA3214	71	63
3605062	3605007	3603994	3603994	-	-	PA3215	51	48
	3605150	3605467		+	-	PA3216	12	8
3605605	3605684	3607075	3607244	+	cyaB	PA3217	32	35
	3607617	3608090		+	-	PA3218	2	1
	3608929	3608117		-	-	PA3219	2	1
	3609076	3609840		+	-	PA3220	36	25
3609899	3609905	3610240	3610243	+	csaA	PA3221	41	303
3610244	3610250	3611149	3611253	+	-	PA3222	14	158
	3611895	3611254	3611254	-	azoR3	PA3223	10	50
3612543	3612359	3612042	3612032	-	-	PA3224	130	109
3612544	3612559	3613488	3613494	+	-	PA3225	54	54
3613495	3613495	3614322		+	-	PA3226	48	51
	3614304	3614867	3614870	+	ppiA	PA3227	169	158
3614928	3614930	3616762	3616779	+	-	PA3228	58	77
	3617044	3617310		+	-	PA3229	11	9
3618467	3618467	3617343	3617343	-	-	PA3230	29	25

	3618884	3618726		-	-	PA3231	9	4
	3619619	3618993		-	-	PA3232	10	6
	3621415	3619616		-	-	PA3233	12	5
	3623160	3621505		-	-	PA3234	33	8
	3623468	3623157		-	-	PA3235	89	13
	3624543	3623686		-	-	PA3236	9	4
	3624837	3625058		+	-	PA3237	6	2
3625172	3625201	3626562	3626565	+	-	PA3238	50	28
	3626566	3627369		+	-	PA3239	43	28
	3627509	3628366		+	-	PA3240	27	14
	3629599	3628430		-	-	PA3241	36	30
3630616	3630605	3629667	3629600	-	-	PA3242	52	59
3630763	3630765	3631556	3631606	+	minC	PA3243	161	96
3631617	3631618	3632433		+	minD	PA3244	781	678
	3632430	3632684	3632684	+	minE	PA3245	932	749
3632685	3632788	3633423	3633435	+	rluA	PA3246	62	66
3633532	3633592	3634881	3634881	+	-	PA3247	101	85
3635491	3635475	3634924	3634882	-	-	PA3248	46	28
	3636257	3635541		-	-	PA3249	7	5
	3636621	3637679		+	-	PA3250	6	2
	3637734	3638543		+	-	PA3251	1	2
	3638540	3639379		+	-	PA3252	4	2
	3639366	3640163		+	-	PA3253	4	2
	3640166	3641161	3641161	+	-	PA3254	17	14
3641162	3641233	3641811	3641871	+	-	PA3255	108	64
3642870	3642834	3641872	3641872	-	-	PA3256	71	42
3642872	3642964	3645060	3645071	+	prc	PA3257	228	139
	3645099	3646904		+	-	PA3258	13	11
3647404	3647397	3646918	3646905	-	-	PA3259	51	34
3648114	3648066	3647755	3647578	-	-	PA3260	92	63
	3648170	3648916		+	-	PA3261	36	29
3650497	3650466	3649705	3649692	-	-	PA3262	689	554

3650798			3650722	-	-	PA3262.1	557	1354
3650890			3650815	-	-	PA3262.2	665	1874
3651046	3651056	3651976	3651976	+	-	PA3263	146	94
	3652063	3652998		+	-	PA3264	29	22
	3653138	3653452		+	-	PA3265	23	27
3653526	3653667	3653876	3653897	+	capB	PA3266	4332	2606
3655985	3655811	3653937	3653926	-	-	PA3267	68	64
3658160	3658151	3655986	3655986	-	-	PA3268	96	92
3659102	3659102	3658248	3658248	-	-	PA3269	36	20
3659846	3659756	3659169	3659103	-	-	PA3270	140	82
	3660022	3663501		+	-	PA3271	54	8
	3667855	3663509		-	-	PA3272	12	9
	3668523	3667924		-	-	PA3273	9	1
	3668474	3668761		+	-	PA3274	17	7
	3669169	3668840		-	-	PA3275	9	7
	3669588	3669166		-	-	PA3276	29	25
	3670633	3669821		-	-	PA3277	22	12
	3671058	3670759		-	-	PA3278	98	43
	3672549	3671227		-	oprP	PA3279	2	1
	3674324	3673008		-	oprO	PA3280	5	3
	3675160	3674570		-	-	PA3281	10	8
	3675897	3675163		-	-	PA3282	9	6
	3676748	3675894		-	-	PA3283	20	14
3677114	3677081	3676767	3676749	-	-	PA3284	84	40
3678333	3678331	3677720	3677475	-	-	PA3285	110	95
3679528	3679386	3678334	3678334	-	-	PA3286	175	165
	3679946	3680461		+	-	PA3287	19	15
3680968	3680968	3680465	3680462	-	-	PA3288	39	41
	3681048	3681461		+	-	PA3289	14	12
	3684148	3681500	3681500	-	-	PA3290	24	22
	3684717	3684163		-	-	PA3291	12	9
	3685762	3684905		-	-	PA3292	8	9

	3686574	3685759		-	-	PA3293	10	10
	3688650	3686584		-	-	PA3294	18	17
3689327	3689316	3688879	3688762	-	-	PA3295	203	111
	3689521	3690951		+	phoA	PA3296	4	3
3695061	3695004	3691024	3691009	-	-	PA3297	72	64
	3695481	3695179		-	-	PA3298	6	6
3697412	3697199	3695511	3695511	-	fadD1	PA3299	293	199
	3699122	3697434		-	fadD2	PA3300	27	31
3699351	3699369	3700319		+	-	PA3301	80	62
	3700316	3700786	3700789	+	-	PA3302	217	144
	3700937	3702112		+	-	PA3303	9	9
	3702950	3702093		-	-	PA3304	19	26
3703156			3703022	-	-	PA3304.1	20	15
	3705161	3703167		-	-	PA3305	30	22
3705521			3705309	-	phrS	PA3305.1	3207	2084
3706545	3706492	3705890	3705522	-	-	PA3306	41	31
	3706854	3706546		-	-	PA3307	34	14
3707090	3707090	3709942	3709959	+	hepA	PA3308	188	164
3710188	3710225	3710680	3710691	+	-	PA3309	978	1120
3712502	3712404	3710749	3710723	-	-	PA3310	44	52
	3712560	3714911		+	-	PA3311	9	7
	3715805	3714915		-	-	PA3312	33	27
	3715804	3716811		+	-	PA3313	161	143
	3716808	3717611		+	-	PA3314	78	62
	3717605	3718438		+	-	PA3315	28	32
	3718435	3719205		+	-	PA3316	31	31
	3719329	3720057		+	-	PA3317	43	31
	3720123	3720623		+	-	PA3318	6	3
	3722759	3720681		-	plcN	PA3319	3	3
	3723445	3722990		-	-	PA3320	4	1
	3723534	3724448		+	-	PA3321	17	17
3725257	3725154	3724456	3724449	-	-	PA3322	53	43

	3725580	3726455		+	-	PA3323	7	6
	3726452	3728230		+	-	PA3324	5	5
	3728240	3729127		+	-	PA3325	15	8
3730269	3730076	3729471	3729441	-	-	PA3326	161	43
	3730557	3737615		+	-	PA3327	9	1
	3737612	3738778		+	-	PA3328	9	1
	3738775	3740103	3740103	+	-	PA3329	8	3
	3740105	3741019		+	-	PA3330	9	2
	3741012	3742268		+	-	PA3331	12	2
	3742265	3742690	3742690	+	-	PA3332	14	2
	3742694	3743686	3743686	+	fabH2	PA3333	11	2
	3743700	3743939		+	-	PA3334	20	1
3744057	3744057	3744809	3744809	+	-	PA3335	9	2
	3744901	3746067		+	-	PA3336	11	7
3747076	3747076	3746078	3746078	-	rfaD	PA3337	85	46
	3747456	3747166		-	-	PA3338	31	17
	3747525	3749711		+	plpD	PA3339	24	24
3752021	3751767	3749719	3749712	-	-	PA3340	30	28
3752509	3752478	3752044	3752037	-	-	PA3341	194	93
	3753612	3752596		-	-	PA3342	14	8
	3754932	3753763		-	-	PA3343	35	18
3757159	3757159	3755021	3754933	-	recQ	PA3344	162	159
3757620	3757593	3757243	3757201	-	-	PA3345	116	139
3759374	3759374	3757659		-	-	PA3346	26	15
3759712	3759681	3759376	3759375	-	-	PA3347	150	74
3760896	3760820	3759996	3759996	-	-	PA3348	192	167
3761960	3761829	3760897	3760897	-	-	PA3349	386	337
3761961	3761961	3762659	3762787	+	-	PA3350	115	74
3762794	3762804	3763127	3763181	+	flgM	PA3351	773	535
3763182	3763182	3763652	3763652	+	-	PA3352	322	260
3763653	3763681	3764472	3764475	+	-	PA3353	141	119
	3765087	3764476		-	-	PA3354	31	28

	3766433	3765129		-	-	PA3355	25	11
3767966	3767858	3766617	3766596	-	-	PA3356	135	75
3767968	3768184	3769530	3769586	+	dsdA	PA3357	43	31
	3769587	3770465		+	-	PA3358	14	14
	3771480	3770470		-	-	PA3359	14	17
	3772561	3771503		-	-	PA3360	5	14
	3773029	3773376		+	lecB	PA3361	6	6
	3773948	3773430		-	-	PA3362	7	14
	3774581	3773991	3773949	-	amiR	PA3363	14	46
3775747	3775735	3774578		-	amiC	PA3364	13	50
3776958	3776876	3775761	3775748	-	-	PA3365	11	80
3778033	3777999	3776959	3776959	-	amiE	PA3366	38	147
3778133			3778034	-	amiL	PA3366.1	40	132
	3778266	3778604		+	-	PA3367	34	36
3778730			3778654	-	-	PA3368.1	31	55
	3778704	3779369		+	-	PA3368	6	7
	3779562	3779840		+	-	PA3369	28	21
	3779930	3780076		+	-	PA3370	25	22
	3780128	3780313		+	-	PA3371	10	20
	3781132	3780362		-	-	PA3372	20	16
	3781680	3781123		-	-	PA3373	36	34
	3782843	3781680		-	-	PA3374	9	8
	3783549	3782833		-	-	PA3375	1	3
	3784414	3783599		-	-	PA3376	3	1
	3785295	3784411		-	-	PA3377	2	1
	3786392	3785292		-	-	PA3378	2	1
	3787021	3786392		-	-	PA3379	2	0
	3787479	3787021		-	-	PA3380	0	0
	3788211	3787489		-	-	PA3381	0	2
	3789026	3788232		-	phnE	PA3382	0	1
	3790097	3789093		-	-	PA3383	3	2
	3790986	3790150		-	phnC	PA3384	4	1

3791245	3791347	3791673	3791739	+	amrZ	PA3385	1392	564
	3791827	3792192		+	-	PA3386	43	20
	3792290	3793060		+	rhlG	PA3387	9	4
	3793113	3793808		+	-	PA3388	29	21
	3794117	3793815		-	-	PA3390	16	7
	3794178	3794591		+	-	PA3389	5	4
	3794819	3796966		+	nosR	PA3391	2	1
	3797009	3798919		+	nosZ	PA3392	3	1
	3798916	3800202		+	nosD	PA3393	3	2
	3800199	3801113		+	nosF	PA3394	3	1
	3801104	3801931		+	nosY	PA3395	3	3
	3801948	3802484		+	nosL	PA3396	7	10
3803376	3803343	3802567	3802547	-	fpr	PA3397	401	408
3803377	3803617	3804543	3804543	+	-	PA3398	44	38
3804889	3804886	3804545	3804544	-	-	PA3399	47	39
	3806073	3804940		-	-	PA3400	17	16
3807242	3807242	3806070		-	-	PA3401	16	13
3808287	3808217	3807246	3807243	-	-	PA3402	44	36
3808874	3808803	3808318	3808318	-	-	PA3403	29	43
	3810613	3809258		-	-	PA3404	2	1
	3811941	3810610		-	hasE	PA3405	1	0
	3813740	3811938		-	hasD	PA3406	0	0
	3814575	3813958		-	hasAp	PA3407	14	12
	3817336	3814661		-	hasR	PA3408	4	2
	3818532	3817546		-	-	PA3409	5	5
	3819112	3818597		-	-	PA3410	13	13
	3819407	3819189		-	-	PA3411	7	10
	3819796	3819527		-	-	PA3412	4	2
3819970	3820006	3820272	3820307	+	-	PA3413	208	71
3820308	3820308	3820892		+	-	PA3414	47	24
	3822497	3821385		-	-	PA3415	6	3
	3823516	3822515		-	-	PA3416	2	0

	3824606	3823509		-	-	PA3417	1	1
	3825773	3824748		-	ldh	PA3418	4	3
	3826019	3826831		+	-	PA3419	20	8
	3829321	3826838		-	-	PA3420	1	1
	3830841	3829471		-	-	PA3421	3	1
	3832655	3830898		-	-	PA3422	3	2
	3832940	3833683		+	-	PA3423	35	22
	3833774	3835180		+	-	PA3424	5	4
	3835215	3835559		+	-	PA3425	8	11
	3836349	3835579		-	-	PA3426	11	10
	3837377	3836466		-	-	PA3427	22	10
	3837786	3837493		-	-	PA3428	4	2
	3838781	3837885		-	-	PA3429	5	5
	3839557	3838778		-	-	PA3430	8	8
	3840372	3839686	3839558	-	-	PA3431	23	21
3840763	3840748	3840359		-	-	PA3432	48	45
	3840844	3841737		+	-	PA3433	20	14
	3843290	3842274		-	-	PA3434	20	20
3844109	3844105	3843653	3843474	-	-	PA3435	220	155
	3844385	3844942		+	-	PA3436	8	24
3845025	3845053	3845757	3845774	+	folM	PA3437	55	30
3845775	3845775	3846335	3846335	+	folE1	PA3438	94	59
3846336	3846337	3846708	3846798	+	folX	PA3439	129	71
3846808	3846837	3847148	3847361	+	-	PA3440	353	310
3847973	3847933	3847718	3847510	-	-	PA3441	87	88
	3848798	3847974		-	-	PA3442	15	16
	3849583	3848795		-	-	PA3443	5	0
	3850752	3849604		-	-	PA3444	1	0
	3851801	3850830		-	-	PA3445	3	1
3852555	3852513	3851920	3851907	-	-	PA3446	10	5
	3853416	3852667		-	-	PA3447	2	1
	3854237	3853413		-	-	PA3448	2	1

	3855242	3854241		-	-	PA3449	1	0
3856145	3856131	3855493	3855395	-	-	PA3450	26	21
	3856337	3856546		+	-	PA3451	13	6
3856709	3856749	3858320	3858320	+	mqoA	PA3452	578	201
3859214	3859209	3858544	3858321	-	-	PA3453	76	71
	3860522	3859338		-	-	PA3454	10	7
	3862165	3860675		-	-	PA3455	43	19
	3862309	3864273		+	-	PA3456	31	28
	3864374	3865126		+	-	PA3457	21	26
	3865754	3865281		-	-	PA3458	57	50
3865897	3865897	3867666		+	-	PA3459	29	13
	3867707	3869464		+	-	PA3460	8	6
	3869461	3870657		+	-	PA3461	8	7
	3870795	3873554		+	-	PA3462	4	4
3873601	3873606	3873836	3873934	+	-	PA3463	33	36
	3875979	3874654		-	-	PA3464	7	6
	3876196	3877911	3877911	+	-	PA3465	55	37
3879376	3879373	3878033	3877912	-	-	PA3466	89	72
	3879559	3880926		+	-	PA3467	3	2
3881020	3881093	3882421	3882440	+	-	PA3468	61	36
3882441	3882441	3882983		+	-	PA3469	69	46
	3882980	3883438	3883559	+	-	PA3470	61	35
3885270	3885254	3883560	3883560	-	-	PA3471	141	61
3885438	3885711	3886307	3886327	+	-	PA3472	59	60
	3887227	3886328	3886328	-	-	PA3473	38	20
	3888084	3887224		-	-	PA3474	26	26
	3888984	3888178		-	pheC	PA3475	33	26
3889816	3889744	3889139	3889112	-	rhlI	PA3476	250	170
3890659	3890650	3889925	3889914	-	rhlR	PA3477	243	118
	3892055	3890775		-	rhlB	PA3478	12	4
	3893008	3892121	3892121	-	rhlA	PA3479	19	4
3893998	3893998	3893432	3893412	-	-	PA3480	273	273

3895205	3895193	3894099	3893999	-	-	PA3481	141	93
3895309	3895324	3897357	3897390	+	metG	PA3482	281	199
3897391	3897391	3898191	3898192	+	-	PA3483	108	93
3898193	3898278	3899504		+	-	PA3484	47	36
	3899501	3899938	3900232	+	-	PA3485	36	27
	3900233	3902659		+	-	PA3486	20	17
	3902663	3905962		+	pldA	PA3487	25	19
3906391	3906391	3907089		+	-	PA3488	19	13
3907184	3907268	3907852		+	-	PA3489	49	48
	3907849	3908415		+	-	PA3490	47	34
	3908412	3910736	3910738	+	-	PA3491	44	38
	3910739	3911773		+	-	PA3492	30	20
	3911773	3912417		+	-	PA3493	34	25
	3912410	3913132		+	-	PA3494	34	24
	3913129	3913767	3913872	+	nth	PA3495	58	62
3913875	3913876	3914055	3914077	+	-	PA3496	272	587
	3915337	3914117		-	-	PA3497	14	17
3916763	3916763	3915807		-	-	PA3498	16	11
	3917212	3916778		-	-	PA3499	8	4
	3918228	3917209		-	-	PA3500	6	5
3918469	3918469	3918251	3918251	-	-	PA3501	1	6
	3918788	3918471		-	-	PA3502	9	6
	3919431	3918796		-	-	PA3503	3	2
	3921258	3919774		-	-	PA3504	7	4
	3922073	3921270		-	-	PA3505	5	5
	3923766	3922084		-	-	PA3506	2	3
	3924556	3923759		-	-	PA3507	4	3
	3925376	3924543		-	-	PA3508	6	3
	3926238	3925369		-	-	PA3509	3	2
	3926777	3926247		-	-	PA3510	7	3
	3927552	3926791		-	-	PA3511	4	2
	3928334	3927558		-	-	PA3512	4	0

	3929338	3928331		-	-	PA3513	5	1
	3930230	3929379		-	-	PA3514	5	1
	3931816	3930743		-	-	PA3515	19	19
	3933317	3931866		-	-	PA3516	6	5
	3934750	3933317		-	-	PA3517	5	3
	3935767	3934781		-	-	PA3518	2	2
	3936837	3935800		-	-	PA3519	2	3
	3937432	3937238		-	-	PA3520	6	4
	3939495	3938020		-	-	PA3521	2	1
	3942653	3939492		-	-	PA3522	3	2
	3943807	3942650		-	-	PA3523	3	3
3944169	3944184	3944570	3944599	+	gloA1	PA3524	465	280
3945879	3945879	3944662	3944644	-	argG	PA3525	513	439
3946982	3946962	3945997	3945880	-	-	PA3526	60	45
3947092	3947095	3948141		+	pyrC	PA3527	160	138
	3948138	3948812	3948916	+	rnt	PA3528	164	145
3949661	3949593	3948991	3948973	-	-	PA3529	1798	1145
3949662			3949676	?	-	predicted RN	615	483
3949845	3949853	3950074	3950086	+	-	PA3530	35	86
3950280	3950284	3950760	3950762	+	bfrB	PA3531	789	337
	3951978	3950827		-	-	PA3532	29	17
3952412	3952387	3952061	3952058	-	-	PA3533	1002	461
	3954608	3952500		-	-	PA3534	7	2
	3954907	3957894		+	-	PA3535	26	25
3958825	3958755	3958288	3957895	-	-	PA3536	62	48
3958995	3959033	3959950	3959950	+	argF	PA3537	144	80
3959951	3959955	3961037	3961037	+	-	PA3538	130	81
3961038	3961144	3961923	3961930	+	-	PA3539	96	67
	3962825	3964135		+	algD	PA3540	6	3
	3964275	3965759		+	alg8	PA3541	4	4
	3965842	3967011		+	alg44	PA3542	3	2
	3967025	3968452		+	algK	PA3543	3	2

	3968449	3969921		+	algE	PA3544	3	1
	3969942	3971573		+	algG	PA3545	20	7
	3971586	3973010		+	algX	PA3546	1	1
	3973014	3974117		+	algL	PA3547	2	1
	3974359	3975921		+	algI	PA3548	3	2
	3975936	3977111		+	algJ	PA3549	3	1
	3977184	3977834		+	algF	PA3550	5	6
	3978031	3979476		+	algA	PA3551	5	4
3979834	3979860	3981008		+	arnB	PA3552	95	70
	3981005	3982024		+	arnC	PA3553	69	52
	3982021	3984009		+	arnA	PA3554	91	77
	3984006	3984893		+	arnD	PA3555	41	44
	3984890	3986539		+	arnT	PA3556	36	32
	3986536	3986883		+	arnE	PA3557	35	31
	3986880	3987293		+	arnF	PA3558	37	35
	3987290	3988684	3988685	+	-	PA3559	76	59
3990595	3990595	3988838	3988838	-	fruA	PA3560	67	69
	3991541	3990597	3990596	-	fruK	PA3561	77	74
3994442	3994411	3991541		-	fruI	PA3562	85	79
3994639	3994739	3995728	3995840	+	fruR	PA3563	55	42
	3995841	3996518		+	-	PA3564	32	19
	3997726	3996806		-	-	PA3565	17	8
3997785	3997822	3998115	3998136	+	-	PA3566	71	38
3998137	3998137	3999150	3999207	+	-	PA3567	57	35
	4001094	3999208		-	-	PA3568	9	5
	4002092	4001196		-	mmsB	PA3569	2	5
	4003601	4002108		-	mmsA	PA3570	7	10
	4003733	4004656		+	mmsR	PA3571	16	9
4004767	4004783	4004959	4004965	+	-	PA3572	504	328
	4006145	4004967		-	-	PA3573	42	34
4006487	4006510	4007148	4007148	+	nalD	PA3574	87	69
4007294	4007507	4008037	4008135	+	-	PA3575	39	58

	4008136	4008558		+	-	PA3576	40	23
	4009051	4008938		-	-	PA3577	15	13
	4010327	4009542		-	-	PA3578	39	9
4012007	4012007	4010523	4010523	-	-	PA3579	60	18
4012728	4012541	4012071	4012008	-	-	PA3580	73	20
4012776	4012806	4013645	4013645	+	glpF	PA3581	78	19
4013670	4013685	4015202	4015202	+	glpK	PA3582	133	35
4015399	4015408	4016163	4016281	+	glpR	PA3583	94	45
	4016441	4017979		+	glpD	PA3584	50	7
4018018	4018112	4018441	4018457	+	glpM	PA3585	20	49
	4019444	4018458		-	-	PA3586	9	16
	4019743	4020663		+	metR	PA3587	39	16
	4021919	4020669		-	-	PA3588	7	3
	4023177	4021972		-	-	PA3589	1	1
	4024732	4023200		-	-	PA3590	5	3
	4025526	4024729		-	-	PA3591	2	0
	4026773	4025586		-	-	PA3592	1	1
	4028539	4026812		-	-	PA3593	2	3
	4028653	4029540		+	-	PA3594	15	9
	4029759	4031168		+	-	PA3595	4	3
	4032238	4031165		-	-	PA3596	5	3
	4033650	4032328		-	-	PA3597	7	3
	4034665	4033850		-	-	PA3598	7	3
	4034793	4035578		+	-	PA3599	30	19
	4035758	4035606		-	-	PA3600	26	10
4036042	4036021	4035758		-	-	PA3601	25	11
4036192	4036265	4037875	4037896	+	-	PA3602	88	47
4038387	4038316	4037945	4037937	-	dgkA	PA3603	78	86
4039048	4039041	4038388	4038388	-	erdR	PA3604	158	148
4039061	4039174	4040100	4040103	+	-	PA3605	43	32
4040869	4040820	4040104	4040104	-	-	PA3606	65	70
4041034	4041079	4042170	4042172	+	potA	PA3607	67	57

4042175	4042175	4043068		+	potB	PA3608	64	41
	4043061	4043831	4043894	+	potC	PA3609	54	40
4043895	4043920	4044984	4045005	+	potD	PA3610	100	75
4045147	4045160	4045570	4045588	+	-	PA3611	222	217
4045589	4045589	4045810	4045891	+	-	PA3612	115	144
4048339	4048339	4045934	4045934	-	-	PA3613	81	88
4048457			4048518	?	-	predicted RN	179	48
	4048519	4049922	4049922	+	-	PA3614	78	28
4049923	4050005	4051075	4051096	+	-	PA3615	74	39
4051561	4051558	4051097	4051097	-	-	PA3616	171	99
4052630	4052604	4051564	4051564	-	recA	PA3617	557	302
	4053244	4052738		-	-	PA3618	59	24
	4053392	4054399		+	-	PA3619	11	5
4054506	4054525	4057092	4057148	+	mutS	PA3620	128	88
4057152	4057159	4057482	4057487	+	fdxA	PA3621	302	283
4057658			4057543	-	rsmZ	PA3621.1	29	55
4058913	4058913	4057909	4057901	-	rpoS	PA3622	241	169
4059956	4059911	4059018	4058914	-	-	PA3623	258	172
4060624	4060592	4059957	4059957	-	pcm	PA3624	317	256
	4061374	4060625	4060625	-	surE	PA3625	256	243
	4062429	4061362		-	-	PA3626	143	131
4062899	4062899	4062426		-	ygbB	PA3627	210	210
4063821	4063821	4062970	4062900	-	-	PA3628	109	67
4065022	4064987	4063875	4063847	-	adhC	PA3629	240	118
	4065119	4066027		+	-	PA3630	18	12
4066079	4066103	4067329		+	-	PA3631	99	82
	4067295	4067543	4067545	+	-	PA3632	176	173
4068308	4068308	4067604	4067551	-	ygbP	PA3633	93	94
4068665	4068612	4068328	4068321	-	-	PA3634	214	209
4070011	4069966	4068677	4068671	-	eno	PA3635	1056	1014
4070857	4070857	4070012	4070012	-	kdsA	PA3636	659	709
4072558	4072488	4070860	4070858	-	pyrG	PA3637	666	798

4072559			4072603	?	-	predicted RN	290	793
4074056	4073987	4072659	4072652	-	-	PA3638	54	63
4075036	4075007	4074057	4074057	-	accA	PA3639	567	615
4075037			4075130	?	-	predicted RN	383	484
4078713	4078678	4075157	4075131	-	dnaE	PA3640	215	181
4078755			4078803	?	-	predicted RN	478	321
4080295	4080223	4078808	4078808	-	-	PA3641	508	392
	4081045	4080440	4080398	-	rnhB	PA3642	102	89
4082184	4082181	4081045		-	lpxB	PA3643	135	156
	4082961	4082185	4082185	-	lpxA	PA3644	559	543
4083441	4083398	4082958		-	fabZ	PA3645	678	730
	4084505	4083444	4083444	-	lpxD	PA3646	416	408
4085040	4085011	4084505		-	-	PA3647	698	655
4087472	4087455	4085062	4085041	-	opr86	PA3648	412	365
4088903	4088878	4087526	4087473	-	-	PA3649	165	179
	4090094	4088904	4088904	-	dxr	PA3650	203	201
	4090906	4090091		-	cdsA	PA3651	248	257
4091655	4091655	4090900		-	uppS	PA3652	476	432
4092230	4092228	4091671	4091656	-	frr	PA3653	1266	930
4093166	4092968	4092231	4092231	-	pyrH	PA3654	543	430
4094166	4094036	4093167	4093167	-	tsf	PA3655	4005	3372
4095023	4094907	4094167	4094167	-	rpsB	PA3656	6882	6330
4095155	4095172	4095957	4095961	+	map	PA3657	479	371
4095973	4096124	4098826	4098834	+	glnD	PA3658	49	39
4098844	4098844	4100052	4100065	+	-	PA3659	75	47
4101849	4101849	4100104	4100104	-	-	PA3660	34	14
	4102087	4102446		+	-	PA3661	18	18
4102702	4102768	4103061	4103061	+	-	PA3662	170	71
4103566	4103429	4103076	4103062	-	-	PA3663	59	41
4103712	4103732	4104079	4104079	+	-	PA3664	187	125
4104080	4104092	4104715	4104715	+	-	PA3665	115	82
4104740	4104745	4105779	4105779	+	dapD	PA3666	383	244

4105780	4105874	4107079		+	-	PA3667	61	47
	4107076	4107498	4107504	+	-	PA3668	44	37
4108485	4108485	4107505	4107505	-	-	PA3669	23	23
	4110336	4108489		-	-	PA3670	21	19
	4111081	4110347		-	-	PA3671	26	19
	4112001	4111078		-	-	PA3672	30	29
4112251	4112285	4114789	4114916	+	plsB	PA3673	59	50
4114917	4114933	4115331	4115344	+	-	PA3674	155	82
4115461	4115468	4116148	4116187	+	-	PA3675	158	133
	4119265	4116188		-	-	PA3676	29	34
	4120373	4119270		-	-	PA3677	11	23
4120438	4120469	4121107	4121107	+	-	PA3678	59	54
4122404	4122391	4121114	4121111	-	-	PA3679	46	44
4123213	4123205	4122420	4122405	-	-	PA3680	81	69
	4123956	4123261		-	-	PA3681	21	20
	4124785	4123961		-	-	PA3682	8	6
4125724	4125724	4124858		-	-	PA3683	46	35
4126163	4126090	4125743	4125725	-	-	PA3684	115	86
4126906	4126844	4126164	4126164	-	-	PA3685	136	103
4127655	4127595	4126948	4126941	-	adk	PA3686	620	275
	4130392	4127756		-	ppc	PA3687	26	14
4130599	4130599	4130943		+	-	PA3688	11	9
	4131422	4130952		-	-	PA3689	31	17
	4131536	4133758		+	-	PA3690	33	24
4133998	4134138	4134542	4134542	+	-	PA3691	59	29
4134544	4134591	4135376	4135376	+	lptF	PA3692	55	34
	4135973	4135452	4135444	-	-	PA3693	64	51
	4136293	4135970		-	-	PA3694	111	108
	4137195	4136290		-	-	PA3695	44	37
4138006	4137938	4137192		-	-	PA3696	45	26
	4139302	4138007		-	-	PA3697	50	32
4139862	4139862	4139311	4139303	-	-	PA3698	71	44

4140832	4140672	4139959	4139863	-	-	PA3699	65	54
4142389	4142389	4140884	4140869	-	lysS	PA3700	538	472
4143670	4143665	4142570	4142390	-	prfB	PA3701	597	521
4144887	4144831	4143788	4143673	-	wspR	PA3702	58	47
	4145946	4144939	4144888	-	wspF	PA3703	59	47
	4148252	4145943		-	wspE	PA3704	74	54
	4148938	4148249		-	wspD	PA3705	76	57
	4150199	4148931		-	wspC	PA3706	64	50
4150711	4150711	4150196		-	wspB	PA3707	92	74
4152467	4152344	4150716	4150712	-	wspA	PA3708	103	78
	4154230	4152611		-	-	PA3709	9	4
	4156012	4154339		-	-	PA3710	7	5
	4156184	4157089	4157089	+	-	PA3711	23	18
	4157821	4157126		-	-	PA3712	41	14
4158042	4158143	4160005	4160328	+	spdH	PA3713	109	44
	4160329	4160970		+	-	PA3714	17	9
4161029	4161091	4161894	4161972	+	-	PA3715	67	51
4163749	4163679	4161973	4161973	-	-	PA3716	290	204
4163865	4163890	4164231	4164329	+	-	PA3717	52	46
	4165571	4164330		-	-	PA3718	6	4
	4165880	4165719		-	armR	PA3719	23	6
	4166313	4165888		-	-	PA3720	24	7
4166493	4166518	4167159	4167168	+	nalC	PA3721	69	85
4167542	4167522	4167172	4167169	-	-	PA3722	169	279
	4168817	4167711		-	-	PA3723	7	5
	4170483	4168987	4168987	-	lasB	PA3724	22	12
4172528	4172485	4170770	4170690	-	recJ	PA3725	78	59
4173068	4173068	4172529	4172529	-	-	PA3726	103	87
4173867	4173867	4173175	4173069	-	-	PA3727	58	52
4179233	4179126	4173886	4173868	-	-	PA3728	96	76
4181377	4181342	4179276	4179276	-	-	PA3729	287	186
4182066	4182019	4181378	4181378	-	-	PA3730	106	80

4182770	4182770	4182075	4182075	-	-	PA3731	224	155
4183342	4183228	4182785	4182771	-	-	PA3732	321	203
	4183710	4184939		+	-	PA3733	31	25
	4185522	4186727		+	-	PA3734	6	5
4188242	4188190	4186781	4186781	-	thrC	PA3735	248	169
4189682	4189547	4188243	4188243	-	hom	PA3736	234	179
4190489	4190489	4189761	4189733	-	dsbC	PA3737	313	257
4191526	4191512	4190616	4190490	-	xerD	PA3738	44	31
	4191727	4193562		+	-	PA3739	12	7
4193659	4193675	4194361	4194403	+	-	PA3740	38	26
4195007	4194856	4194431	4194431	-	-	PA3741	72	54
4195386	4195358	4195008	4195008	-	rplS	PA3742	4877	4147
4195387			4195399	?	-	predicted RN	4606	4157
4196164	4196158	4195400	4195400	-	trmD	PA3743	2359	2713
4196692	4196692	4196165	4196165	-	rimM	PA3744	2994	3589
4196975	4196959	4196708	4196693	-	rpsP	PA3745	3715	4000
4196976			4196998	?	-	predicted RN	1935	2442
4198555	4198542	4197169	4196999	-	ffh	PA3746	382	334
4198841	4198843	4199643	4199655	+	-	PA3747	142	103
4199656	4199656	4200948	4201111	+	-	PA3748	43	39
	4201252	4202565		+	-	PA3749	7	5
	4203322	4202573		-	-	PA3750	34	31
4204542	4204505	4203324	4203323	-	purT	PA3751	86	61
	4204737	4204543	4204543	-	-	PA3752	94	56
4205295	4205258	4204734		-	-	PA3753	115	79
4205923	4205907	4205296	4205296	-	-	PA3754	125	56
4206032	4206048	4206602	4206602	+	-	PA3755	64	30
4206603	4206665	4207165	4207192	+	-	PA3756	181	145
	4207408	4208151		+	-	PA3757	14	10
	4208168	4209259		+	-	PA3758	18	10
	4209256	4210278		+	-	PA3759	15	11
	4210295	4212823		+	-	PA3760	17	12

	4212848	4214560		+	-	PA3761	13	11
4215142	4215079	4214762	4214751	-	-	PA3762	149	33
4219458	4219440	4215544	4215544	-	purL	PA3763	279	264
4219736	4219841	4221199	4221212	+	-	PA3764	62	46
	4221797	4221213		-	-	PA3765	31	24
4223179	4223179	4221923	4221923	-	-	PA3766	92	14
	4224116	4223568	4223387	-	-	PA3767	80	77
4225516	4225498	4224107		-	-	PA3768	113	116
4227300	4227237	4225660	4225642	-	guaA	PA3769	483	390
4227301			4227315	?	-	predicted RN	671	539
4228801	4228786	4227317	4227316	-	guaB	PA3770	740	668
	4229050	4230027		+	-	PA3771	9	6
	4230162	4231058		+	-	PA3772	1	1
	4231071	4232228		+	-	PA3773	0	0
	4232258	4233400		+	-	PA3774	1	1
	4234167	4233385		-	-	PA3775	2	2
	4234275	4235183		+	-	PA3776	7	7
4235210	4235220	4236599	4236605	+	xseA	PA3777	67	58
4236606	4236606	4237541		+	-	PA3778	31	28
	4237693	4238733		+	-	PA3779	17	17
	4238808	4239302		+	-	PA3780	12	10
	4239299	4240579		+	-	PA3781	11	12
	4242295	4241342		-	-	PA3782	31	24
	4242416	4243045		+	-	PA3783	23	17
4243708	4243652	4243080	4243046	-	-	PA3784	43	52
4244192	4244185	4243709	4243709	-	-	PA3785	36	66
4244714	4244704	4244315	4244284	-	-	PA3786	50	44
4244861	4244876	4245724	4245809	+	-	PA3787	43	35
	4245810	4246205		+	-	PA3788	31	23
4247638	4247638	4246223		-	-	PA3789	28	26
4249975	4249874	4247703	4247639	-	oprC	PA3790	45	59
4250593	4250456	4249995	4249976	-	-	PA3791	39	37

4250755	4250828	4252606	4252611	+	leuA	PA3792	108	71
4253009	4253009	4252677	4252677	-	-	PA3793	178	125
4253569	4253518	4253063	4253013	-	-	PA3794	107	78
	4253699	4254649	4254649	+	-	PA3795	38	30
	4255324	4254737		-	-	PA3796	44	43
	4256252	4255458	4255333	-	-	PA3797	83	56
4257426	4257388	4256240		-	-	PA3798	83	54
4259219	4259177	4257696	4257696	-	-	PA3799	222	199
4260398	4260397	4259255	4259255	-	-	PA3800	437	360
4261043	4261043	4260399	4260399	-	-	PA3801	429	362
4262368	4262359	4261070	4261044	-	hisS	PA3802	317	301
	4263493	4262378	4262374	-	gcpE	PA3803	314	317
	4264533	4263490		-	-	PA3804	221	207
4265305	4265288	4264530		-	pilF	PA3805	252	240
4266445	4266445	4265306	4265306	-	-	PA3806	902	727
4266910	4266901	4266470	4266446	-	ndk	PA3807	3544	2646
4266911			4267076	?	-	predicted RN	1331	1597
4267345	4267345	4267145	4267116	-	-	PA3808	484	404
4267715	4267710	4267372	4267346	-	fdx2	PA3809	441	437
4269582	4269576	4267717	4267716	-	hscA	PA3810	233	182
4270140	4270140	4269619	4269619	-	hscB	PA3811	443	356
4270498	4270471	4270148	4270141	-	iscA	PA3812	927	740
4270885	4270885	4270499	4270499	-	iscU	PA3813	720	537
4270886			4270919	?	-	predicted RN	619	588
4272135	4272135	4270921	4270920	-	iscS	PA3814	629	464
4272146			4272156	?	-	predicted RN	481	386
4272724	4272656	4272165	4272158	-	iscR	PA3815	246	250
	4273577	4272801	4272737	-	cysE	PA3816	156	99
4274367	4274350	4273577		-	-	PA3817	117	85
4274493	4274502	4275317	4275323	+	-	PA3818	227	257
4276011	4275970	4275422	4275417	-	-	PA3819	169	158
4277084	4277074	4276154	4276111	-	secF	PA3820	650	491

4278999	4278947	4277085	4277085	-	secD	PA3821	545	420
4279345	4279345	4279007	4279004	-	-	PA3822	1016	799
4280519	4280507	4279389	4279346	-	tgt	PA3823	266	235
4281566	4281563	4280520	4280520	-	queA	PA3824	107	128
4281665			4281751	+	-	PA3824.1	47	85
	4283658	4282078		-	-	PA3825	12	7
4283742	4283788	4284285	4284401	+	-	PA3826	71	79
	4285484	4284417	4284402	-	-	PA3827	153	136
4286629	4286595	4285477		-	-	PA3828	148	154
	4287710	4286787		-	-	PA3829	10	5
	4288606	4287794		-	-	PA3830	16	11
4288726	4288943	4290430		+	pepA	PA3831	307	230
	4290427	4290855	4290866	+	holC	PA3832	134	117
4290867	4290867	4291235	4291237	+	-	PA3833	99	94
4291339	4291356	4294208	4294208	+	valS	PA3834	303	247
4294640	4294605	4294255	4294218	-	-	PA3835	40	35
4297202	4297250	4298227	4298227	+	-	PA3836	196	203
4298228	4298311	4299201	4299203	+	-	PA3837	95	96
4299204	4299204	4299998	4300006	+	-	PA3838	101	82
	4300117	4301949	4301949	+	-	PA3839	43	30
	4302041	4303051	4303051	+	-	PA3840	38	29
	4304502	4303141		-	exoS	PA3841	70	12
	4304690	4305040		+	-	PA3842	49	14
	4305064	4305426		+	-	PA3843	27	5
	4306256	4305645		-	-	PA3844	10	6
	4306380	4307276		+	-	PA3845	24	17
	4307808	4307266		-	-	PA3846	33	17
	4307912	4308382		+	-	PA3847	27	24
4308456	4308456	4309811		+	-	PA3848	35	23
4309867	4309904	4310908	4310908	+	-	PA3849	178	135
4311871	4311862	4310951	4310909	-	-	PA3850	128	88
4312706	4312703	4311951	4311895	-	-	PA3851	57	27

4313644	4313642	4312707	4312707	-	-	PA3852	50	32
4314452	4314452	4313763	4313746	-	-	PA3853	63	57
	4314795	4314481	4314468	-	-	PA3854	64	60
4315555	4315489	4314788		-	-	PA3855	47	38
	4316038	4315556	4315556	-	-	PA3856	44	18
4316844	4316825	4316109	4316039	-	pcs	PA3857	82	40
	4317962	4316937		-	-	PA3858	28	16
4318236	4318259	4318906	4318921	+	-	PA3859	97	64
	4320820	4318922		-	-	PA3860	15	9
4321098	4321434	4322627	4322635	+	rhl	PA3861	409	350
	4322788	4323735		+	dauB	PA3862	35	28
4323758	4323758	4324885		+	dauA	PA3863	31	20
4324934	4324948	4325580	4325603	+	dauR	PA3864	58	46
	4326395	4325604		-	-	PA3865	34	26
4329999	4329991	4327697	4327305	-	-	PA3866	158	59
	4330321	4330896		+	-	PA3867	13	9
	4331452	4332462		+	-	PA3868	6	2
	4332653	4333036		+	-	PA3869	13	8
	4334346	4333357		-	moaA1	PA3870	9	7
	4335221	4334403		-	-	PA3871	3	5
	4335959	4335276		-	narI	PA3872	4	8
	4336702	4335962		-	narJ	PA3873	2	4
	4338249	4336708		-	narH	PA3874	5	9
	4342046	4338261		-	narG	PA3875	4	5
	4343528	4342122		-	narK2	PA3876	3	4
	4344835	4343540		-	narK1	PA3877	4	4
	4345010	4346878		+	narX	PA3878	44	65
	4346875	4347534	4347597	+	narL	PA3879	98	101
4347619	4347619	4348014	4348099	+	-	PA3880	147	76
	4348186	4348650		+	-	PA3881	51	24
	4348666	4349415		+	-	PA3882	28	18
4349502	4349502	4350332		+	-	PA3883	27	23

	4350353	4350739		+	-	PA3884	12	14
	4350834	4351490		+	tpbA	PA3885	6	6
	4351595	4352494		+	-	PA3886	45	23
4354192	4354102	4352828	4352495	-	nhaP	PA3887	88	40
	4354544	4355266		+	-	PA3888	11	6
	4355266	4356201		+	-	PA3889	8	7
	4356201	4356863		+	-	PA3890	13	11
	4356876	4358039		+	-	PA3891	10	16
4359075	4359075	4358167	4358106	-	-	PA3892	19	83
	4361497	4359302	4359076	-	-	PA3893	16	66
4363090	4362984	4361494		-	-	PA3894	25	120
4363167	4363230	4364183	4364183	+	-	PA3895	35	35
4364184	4364278	4365255	4365272	+	-	PA3896	40	32
	4366175	4365273		-	-	PA3897	10	6
	4366301	4367182		+	-	PA3898	20	17
	4367283	4367792		+	-	PA3899	13	9
	4367789	4368742		+	-	PA3900	9	7
4368813	4368837	4371191	4371191	+	fecA	PA3901	88	65
4371792	4371748	4371287	4371192	-	-	PA3902	79	57
4371953	4371969	4373552	4373556	+	prfC	PA3903	250	214
4373938	4373938	4374333		+	-	PA3904	215	172
	4374330	4374857		+	-	PA3905	152	135
	4374850	4375233		+	-	PA3906	176	142
	4375230	4376015		+	-	PA3907	155	126
	4376012	4376731	4376762	+	-	PA3908	190	149
	4379650	4377311		-	eddB	PA3909	4	2
	4381249	4379687		-	eddA	PA3910	2	2
	4382016	4381501		-	-	PA3911	12	29
	4382900	4382010		-	-	PA3912	22	33
	4383907	4382912		-	-	PA3913	51	54
	4385245	4384022		-	moeA1	PA3914	4	2
	4385799	4385242		-	moaB1	PA3915	6	6

4386352	4386352	4385900	4385800	-	moaE	PA3916	61	51
	4386608	4386357	4386353	-	moaD	PA3917	93	57
4387104	4387087	4386605		-	moaC	PA3918	114	72
	4388727	4387336		-	-	PA3919	26	11
	4389231	4391609		+	-	PA3920	5	3
	4394361	4391641		-	-	PA3921	18	15
	4396061	4394694		-	-	PA3922	8	4
4398047	4398047	4396122	4396122	-	-	PA3923	15	7
	4399941	4398259		-	-	PA3924	9	4
4400097	4400123	4401298	4401317	+	-	PA3925	62	78
	4402536	4401355		-	-	PA3926	8	6
	4402638	4403426		+	-	PA3927	19	5
	4403877	4403707		-	-	PA3928	26	16
	4404898	4403891		-	cioB	PA3929	25	11
	4406368	4404902	4404902	-	cioA	PA3930	18	4
	4406806	4407585		+	-	PA3931	3	5
	4407760	4408734		+	-	PA3932	6	5
	4408924	4410885	4410885	+	-	PA3933	17	10
4413038	4413029	4410993	4410886	-	-	PA3934	57	44
	4414025	4413192		-	tauD	PA3935	6	4
	4414950	4414132		-	-	PA3936	4	1
	4415731	4414940		-	-	PA3937	1	3
	4416809	4415796		-	-	PA3938	3	4
	4417934	4417014		-	-	PA3939	11	4
4418164	4418303	4418584	4418584	+	-	PA3940	2686	2047
	4419304	4418708	4418647	-	-	PA3941	76	60
4420203	4420170	4419301		-	tesB	PA3942	93	64
	4421799	4420204		-	-	PA3943	20	17
	4422380	4421802		-	-	PA3944	33	28
	4422997	4422407	4422407	-	-	PA3945	29	15
	4426850	4423212		-	rocS1	PA3946	6	4
	4428130	4426952		-	rocR	PA3947	12	8

4428282	4428412	4429041	4429041	+	rocA1	PA3948	132	82
4429042	4429401	4430576	4430598	+	-	PA3949	66	55
4431948	4431948	4430599	4430599	-	-	PA3950	114	94
4432540	4432529	4432026	4431949	-	-	PA3951	75	60
	4433170	4432595		-	-	PA3952	34	27
	4433411	4433992		+	-	PA3953	9	5
	4434150	4435244		+	-	PA3954	3	3
4435327	4435328	4435984	4436041	+	-	PA3955	41	37
	4436051	4436485		+	-	PA3956	53	22
	4437328	4436492		-	-	PA3957	12	6
4437373	4437390	4438529	4438569	+	-	PA3958	47	30
4438602	4438619	4439353	4439353	+	-	PA3959	15	31
4439354	4439377	4439784	4439794	+	-	PA3960	18	40
4442376	4442311	4439795	4439795	-	-	PA3961	51	44
4442453	4442453	4442869	4442884	+	-	PA3962	79	60
	4442958	4443854		+	-	PA3963	20	16
	4443811	4444596		+	-	PA3964	6	4
4444950	4444978	4445487	4445594	+	-	PA3965	53	40
4445618	4445689	4445898	4445898	+	-	PA3966	513	451
4445946	4445999	4446391	4446391	+	-	PA3967	173	153
4447062	4446994	4446425	4446392	-	-	PA3968	63	50
	4447133	4448218		+	-	PA3969	30	19
4450446	4450392	4448893	4448219	-	amn	PA3970	66	35
	4450878	4450447		-	-	PA3971	13	10
	4452539	4450890		-	-	PA3972	24	17
4453183	4453183	4452536		-	-	PA3973	40	13
	4455676	4453289		-	ladS	PA3974	37	30
4455887	4455887	4456684	4456691	+	thiD	PA3975	155	135
4456695	4456695	4457324	4457325	+	thiE	PA3976	96	70
4457335	4457362	4458645	4458645	+	hemL	PA3977	225	196
4458806	4458854	4459402	4459402	+	-	PA3978	236	189
4459762	4459750	4459418	4459403	-	-	PA3979	185	213

4459868	4459879	4461219	4461375	+	-	PA3980	234	213
4461380	4461388	4462410		+	-	PA3981	330	240
	4462400	4462882		+	-	PA3982	230	232
	4462879	4463718	4463720	+	-	PA3983	246	280
4463816	4463904	4465439	4465455	+	lnt	PA3984	98	95
4466250	4466250	4465489		-	-	PA3985	39	26
	4466754	4466323		-	-	PA3986	13	9
4466911	4466925	4469546	4469608	+	leuS	PA3987	417	303
4469609	4469613	4470236	4470236	+	-	PA3988	291	245
4470237	4470274	4471311	4471311	+	holA	PA3989	108	104
4471312	4471403	4471555	4471626	+	-	PA3990	24	45
	4472198	4471671		-	-	PA3991	15	11
	4472131	4473477	4473622	+	-	PA3992	81	49
4473623	4473623	4474639		+	-	PA3993	40	36
	4475849	4474959		-	-	PA3994	4	3
	4475955	4476848		+	-	PA3995	22	18
	4477977	4476994	4476990	-	lis	PA3996	303	326
	4478627	4477974		-	lipB	PA3997	284	307
4478978	4478908	4478627		-	-	PA3998	508	476
4480179	4480139	4478979	4478979	-	dacC	PA3999	519	492
4480187			4480204	?	-	predicted RN	486	468
	4481233	4480205	4480205	-	-	PA4000	228	225
4482260	4482252	4481230		-	sltB1	PA4001	156	168
4483365	4483364	4482261	4482261	-	rodA	PA4002	108	104
4485336	4485336	4483396	4483396	-	pbpA	PA4003	83	71
4485823	4485816	4485349	4485337	-	-	PA4004	154	131
4486180	4486180	4485824	4485824	-	-	PA4005	416	350
	4486847	4486203	4486181	-	nadD	PA4006	227	197
4488112	4488112	4486847		-	proA	PA4007	224	195
	4488410	4489633		+	-	PA4008	9	3
	4490498	4489641		-	-	PA4009	10	8
4490540	4490562	4491281		+	-	PA4010	102	108

	4491278	4492591	4492591	+	-	PA4011	82	72
4493214	4493195	4492602	4492592	-	-	PA4012	116	92
	4493925	4493215	4493215	-	-	PA4013	81	59
4494403	4494335	4493925		-	-	PA4014	69	53
	4494481	4494936		+	-	PA4015	53	29
	4495126	4496865	4496865	+	-	PA4016	34	23
4496876	4496912	4497553	4497553	+	-	PA4017	53	41
4497848	4497848	4497573	4497559	-	-	PA4018	77	59
4498487	4498479	4497850	4497849	-	-	PA4019	82	60
4499864	4499843	4498488	4498488	-	mpl	PA4020	110	85
	4501932	4500001	4500001	-	-	PA4021	33	29
4502229	4502271	4503791	4503791	+	-	PA4022	57	234
4503792	4504001	4505449	4505507	+	-	PA4023	14	32
4505508	4505508	4506902	4506912	+	eutB	PA4024	20	49
4506913	4506913	4507734	4507852	+	-	PA4025	18	34
4507864	4507897	4508358	4508407	+	-	PA4026	128	63
	4508408	4509301		+	-	PA4027	14	11
	4509776	4509997		+	-	PA4028	2	4
4510948	4510971	4511636	4511636	+	-	PA4029	106	110
4511637	4511639	4512469	4512534	+	-	PA4030	62	51
4512536	4512549	4513076	4513076	+	ppa	PA4031	1462	1078
4513939	4513884	4513168	4513077	-	-	PA4032	69	83
	4514079	4514348		+	-	PA4033	13	5
	4514695	4515384		+	aqpZ	PA4034	12	5
	4515550	4516551		+	-	PA4035	45	25
	4516570	4518870		+	-	PA4036	13	10
	4519275	4520201		+	-	PA4037	4	3
	4520198	4520932		+	-	PA4038	5	2
	4520942	4522744		+	-	PA4039	7	3
	4522746	4523753		+	-	PA4040	9	4
	4525154	4523985		-	-	PA4041	7	1
4525309	4525312	4525554		+	xseB	PA4042	199	146

	4525551	4526438	4526537	+	ispA	PA4043	99	71
4526540	4526547	4528430	4528432	+	dxs	PA4044	252	201
	4529579	4528782	4528443	-	-	PA4045	74	58
	4529998	4529579		-	-	PA4046	150	130
4530615	4530612	4529995		-	ribA	PA4047	256	217
4531387	4531387	4530743	4530618	-	-	PA4048	70	47
4532136	4532136	4531390	4531388	-	-	PA4049	64	64
	4532660	4532145	4532137	-	pgpA	PA4050	126	145
4533639	4533621	4532653		-	thiL	PA4051	158	156
	4534119	4533640	4533640	-	nusB	PA4052	541	493
4534688	4534592	4534116		-	ribE	PA4053	645	643
4534689			4534701	?	-	predicted RN	421	443
4535807	4535807	4534710	4534705	-	ribB	PA4054	152	208
4536839	4536493	4535834	4535808	-	ribC	PA4055	161	234
	4538042	4536921	4536844	-	ribD	PA4056	106	83
4538507	4538503	4538039		-	nrdR	PA4057	170	126
4538684	4538689	4539159		+	-	PA4058	82	84
	4539156	4539533	4539533	+	-	PA4059	65	57
4539534	4539539	4539817	4539877	+	-	PA4060	48	51
4539885	4539889	4540758	4540758	+	-	PA4061	210	166
4541139	4541139	4540783	4540760	-	-	PA4062	48	27
	4541272	4541862		+	-	PA4063	11	10
	4541951	4542655		+	-	PA4064	16	7
	4542658	4543923		+	-	PA4065	17	10
	4543941	4544459		+	-	PA4066	29	22
4544599	4544607	4545305	4545305	+	oprG	PA4067	2816	4274
4545306			4545319	?	-	predicted RN	391	850
	4546675	4545746	4545381	-	-	PA4068	139	102
4547698	4547552	4546668		-	-	PA4069	183	114
	4548771	4547821		-	-	PA4070	11	5
	4549510	4548932		-	-	PA4071	0	1
	4550975	4549485		-	-	PA4072	6	2

	4552630	4551143		-	-	PA4073	5	2
	4552859	4553527		+	-	PA4074	15	9
	4554360	4553524		-	-	PA4075	23	14
4554462	4554467	4554841		+	-	PA4076	49	46
	4554838	4555044	4555103	+	-	PA4077	33	26
	4555254	4558229		+	-	PA4078	5	2
4558299	4558302	4558991	4559005	+	-	PA4079	127	80
	4559650	4559006		-	-	PA4080	50	33
	4561440	4560295		-	cupB6	PA4081	6	3
	4564596	4561540		-	cupB5	PA4082	4	3
	4565424	4564684		-	cupB4	PA4083	1	0
	4567955	4565421		-	cupB3	PA4084	2	0
	4569023	4568277		-	cupB2	PA4085	4	1
	4569623	4569054		-	cupB1	PA4086	11	12
	4570096	4570920		+	-	PA4087	0	0
	4570929	4572314		+	-	PA4088	0	1
	4572311	4573072		+	-	PA4089	2	1
4573276	4573285	4573575	4573576	+	-	PA4090	97	46
	4573741	4575303		+	hpaA	PA4091	5	4
	4575332	4575844		+	hpaC	PA4092	7	7
	4576324	4575908		-	-	PA4093	7	9
	4577331	4576372		-	-	PA4094	31	19
	4577650	4578183		+	-	PA4095	1	1
	4578254	4579513		+	-	PA4096	1	0
	4579510	4580568		+	-	PA4097	1	2
	4580583	4581308		+	-	PA4098	5	3
	4581393	4582697		+	-	PA4099	1	0
	4582852	4584531		+	-	PA4100	3	1
	4585149	4585889		+	bfmR	PA4101	16	8
	4585886	4587190		+	bfmS	PA4102	9	5
	4587320	4587949		+	-	PA4103	3	3
	4587981	4588490		+	-	PA4104	3	3

	4589498	4588743		-	-	PA4105	2	1
	4590321	4589491		-	-	PA4106	3	1
	4590863	4590396		-	-	PA4107	2	2
	4591187	4592431		+	-	PA4108	26	5
	4593880	4592990		-	ampR	PA4109	30	27
	4594029	4595222		+	ampC	PA4110	9	9
	4595287	4595685		+	-	PA4111	35	14
	4600102	4595849		-	-	PA4112	11	10
	4601420	4600230		-	-	PA4113	28	26
4602178	4602083	4601574	4601421	-	-	PA4114	68	41
4602364	4602503	4603888	4603897	+	-	PA4115	100	39
4604006	4604006	4604593		+	bphO	PA4116	58	35
4604594	4604621	4606807	4606870	+	bphP	PA4117	62	40
4606877	4606885	4607454	4607495	+	-	PA4118	82	61
	4608384	4607578		-	aph	PA4119	42	27
	4609347	4608436		-	-	PA4120	8	5
	4609567	4610226		+	-	PA4121	4	2
	4610237	4611016		+	-	PA4122	6	3
	4611013	4612473		+	hpcC	PA4123	5	2
	4612605	4613528		+	hpcB	PA4124	11	5
	4613539	4613931		+	hpcD	PA4125	8	7
	4614024	4615328		+	-	PA4126	3	1
	4615346	4616149		+	hpcG	PA4127	5	5
	4616162	4616968		+	-	PA4128	3	2
	4617538	4617050		-	-	PA4129	12	10
	4619208	4617535		-	-	PA4130	10	7
4619314	4619314	4621035	4621035	+	-	PA4131	9	3
	4621133	4622548		+	-	PA4132	23	12
	4622812	4624239	4624239	+	-	PA4133	36	61
	4624323	4624565		+	-	PA4134	22	20
4625017	4625003	4624581	4624569	-	-	PA4135	88	85
	4626297	4625089		-	-	PA4136	13	10

	4626662	4627918		+	-	PA4137	8	10
	4629185	4627947		-	tyrS	PA4138	28	33
	4629944	4630243		+	-	PA4139	27	21
	4630375	4632168		+	-	PA4140	4	2
4632452	4632477	4632776	4632782	+	-	PA4141	107	57
	4632873	4634129		+	-	PA4142	5	2
	4634139	4636298		+	-	PA4143	3	1
	4636298	4637713		+	-	PA4144	3	2
	4637809	4638699		+	-	PA4145	32	17
	4638887	4639291		+	-	PA4146	11	6
	4641379	4639502		-	acoR	PA4147	11	15
	4641711	4642511		+	-	PA4148	5	3
	4642508	4643548		+	-	PA4149	12	7
	4643571	4644545		+	-	PA4150	10	5
	4644578	4645597		+	acoB	PA4151	6	4
	4645594	4646706		+	-	PA4152	2	3
	4646720	4647811		+	-	PA4153	7	5
	4648563	4647895		-	-	PA4154	19	15
	4650306	4648999		-	-	PA4155	3	1
	4652458	4650374		-	-	PA4156	2	2
4652630	4652714	4653502	4653508	+	-	PA4157	39	43
	4654306	4653509		-	fepC	PA4158	13	10
	4654432	4655337		+	fepB	PA4159	3	2
	4655368	4656390		+	fepD	PA4160	4	4
	4656387	4657418		+	fepG	PA4161	7	5
	4658145	4657429		-	-	PA4162	11	6
4658351	4658450	4660159	4660159	+	-	PA4163	87	73
4660161	4660221	4660547	4660562	+	-	PA4164	68	53
	4662053	4660563		-	-	PA4165	24	15
	4662185	4662643		+	-	PA4166	11	4
	4663511	4662693		-	-	PA4167	5	3
	4663854	4666262		+	fpvB	PA4168	12	6

	4666755	4666327		-	-	PA4169	22	17
	4666836	4667768		+	-	PA4170	12	7
	4667904	4668467		+	-	PA4171	5	4
	4668490	4669290		+	-	PA4172	1	1
	4669720	4669322		-	-	PA4173	0	0
	4669861	4670784		+	-	PA4174	18	10
	4671319	4672707		+	piv	PA4175	52	18
4673322	4673319	4673038	4672770	-	ppiC2	PA4176	174	84
	4673462	4673863		+	-	PA4177	7	6
	4673963	4674706		+	-	PA4178	15	13
	4674944	4676239		+	-	PA4179	94	6
	4677925	4676282	4676282	-	-	PA4180	52	23
	4678210	4678929		+	-	PA4181	15	9
	4678957	4679595	4679595	+	-	PA4182	24	19
	4680066	4679599		-	-	PA4183	27	20
	4681114	4680092		-	-	PA4184	19	22
	4681421	4682149		+	-	PA4185	33	26
	4682242	4683561		+	-	PA4186	27	18
	4683697	4685025		+	-	PA4187	4	4
	4685090	4686001		+	-	PA4188	3	3
	4685994	4687484		+	-	PA4189	3	2
4687621	4687652	4688848	4688866	+	pqsL	PA4190	49	30
	4689893	4688889		-	-	PA4191	14	9
	4690645	4689902		-	-	PA4192	6	6
	4691346	4690642		-	-	PA4193	4	5
	4692041	4691343		-	-	PA4194	7	4
4692993	4692956	4692126	4692042	-	-	PA4195	12	9
	4693756	4693112		-	bfiR	PA4196	25	28
	4696035	4693759		-	bfiS	PA4197	23	18
	4696281	4697903		+	-	PA4198	39	22
	4698021	4699802		+	-	PA4199	37	23
	4700000	4700866		+	-	PA4200	31	27

4701964	4701946	4700906	4700867	-	ddlA	PA4201	69	38
4703130	4703097	4702042	4702026	-	-	PA4202	52	36
	4703205	4704059		+	-	PA4203	18	14
	4704139	4705305		+	ppgL	PA4204	15	10
	4705956	4706402		+	mexG	PA4205	14	25
	4706410	4707522		+	mexH	PA4206	6	13
	4707535	4710624		+	mexI	PA4207	8	11
	4710621	4712084		+	opmD	PA4208	7	9
	4713099	4712095		-	phzM	PA4209	6	4
	4713796	4714284		+	phzA1	PA4210	3	0
	4714314	4714802		+	phzB1	PA4211	5	2
	4714826	4716043		+	phzC1	PA4212	10	12
	4716040	4716663		+	phzD1	PA4213	2	2
	4716660	4718543		+	phzE1	PA4214	4	1
	4718557	4719393		+	phzF1	PA4215	2	2
	4719419	4720063		+	phzG1	PA4216	8	5
	4720301	4721509		+	phzS	PA4217	11	4
	4722858	4721614		-	-	PA4218	1	1
	4724035	4722851		-	-	PA4219	1	0
	4724639	4724358		-	-	PA4220	2	1
	4726801	4724639		-	fptA	PA4221	5	5
	4728617	4726893		-	-	PA4222	5	3
	4730326	4728614		-	-	PA4223	3	2
	4731372	4730323		-	pchG	PA4224	2	1
	4736798	4731369		-	pchF	PA4225	3	2
	4741111	4736795		-	pchE	PA4226	3	2
	4742195	4741305		-	pchR	PA4227	13	14
	4742424	4744067		+	pchD	PA4228	3	2
	4744064	4744819		+	pchC	PA4229	1	1
	4744819	4745124		+	pchB	PA4230	2	2
	4745121	4746551		+	pchA	PA4231	9	9
4747153	4747137	4746640	4746635	-	ssb	PA4232	802	544

4748545	4748542	4747154	4747154	-	-	PA4233	116	110
4748751	4748756	4751593	4751593	+	uvrA	PA4234	199	157
4752134	4752129	4751665	4751664	-	bfrA	PA4235	294	199
4753751	4753708	4752260	4752243	-	katA	PA4236	156	137
4754379	4754379	4753990	4753966	-	rplQ	PA4237	6582	5485
4755424	4755424	4754423	4754380	-	rpoA	PA4238	7554	6423
4756072	4756067	4755447	4755425	-	rpsD	PA4239	8174	6731
4756473	4756473	4756084	4756073	-	rpsK	PA4240	6916	5815
4756474			4756488	?	-	predicted RN	8357	7187
4756969	4756848	4756492	4756489	-	rpsM	PA4241	8770	7885
4757123	4757095	4756979	4756972	-	rpmJ	PA4242	9320	8148
	4758452	4757124	4757124	-	secY	PA4243	8222	7229
4758887	4758887	4758453		-	rplO	PA4244	10999	8815
4759067	4759067	4758891	4758889	-	rpmD	PA4245	13969	11260
4759573	4759570	4759070	4759068	-	rpsE	PA4246	11552	9228
4759924	4759924	4759574	4759574	-	rplR	PA4247	9575	7704
4760468	4760468	4759935	4759925	-	rplF	PA4248	10499	9114
4760888	4760872	4760480	4760469	-	rpsH	PA4249	12265	11514
4760889			4760911	?	-	predicted RN	10529	9663
4761367	4761367	4761062	4760912	-	rpsN	PA4250	7878	6867
4761920	4761920	4761381	4761368	-	rplE	PA4251	11644	9627
4762254	4762254	4761940	4761921	-	rplX	PA4252	10999	8794
4762635	4762635	4762267	4762255	-	rplN	PA4253	10689	8521
4762927	4762925	4762659	4762636	-	rpsQ	PA4254	10518	8546
	4763119	4762928	4762928	-	rpmC	PA4255	9247	7602
4763537	4763532	4763119		-	rplP	PA4256	11277	8999
4764242	4764230	4763544	4763538	-	rpsC	PA4257	10750	8686
4764587	4764575	4764243	4764243	-	rplV	PA4258	10121	8138
4764879	4764863	4764588	4764588	-	rpsS	PA4259	11100	8790
4765701	4765701	4764880	4764880	-	rplB	PA4260	9154	7530
	4766012	4765713	4765702	-	rplW	PA4261	11038	9330
4766611	4766611	4766009		-	rplD	PA4262	11809	9962

4767271	4767260	4766625	4766619	-	rplC	PA4263	11230	10084
4767724	4767654	4767343	4767272	-	rpsJ	PA4264	16053	13801
4769034	4769004	4767811	4767785	-	tufA	PA4265	12500	9552
4771155	4771155	4769035	4769035	-	fusA1	PA4266	10244	8356
4771707	4771656	4771186	4771165	-	rpsG	PA4267	10891	9022
4771708			4771724	?	-	predicted RN	11734	9561
4772141	4772127	4771756	4771725	-	rpsL	PA4268	10548	8916
4776478	4776478	4772279	4772149	-	rpoC	PA4269	2645	2415
4780767	4780617	4776544	4776479	-	rpoB	PA4270	2249	2118
4780833			4780768	-	-	PA4270.1	6019	5830
4781234	4781207	4780839	4780834	-	rplL	PA4271	17692	15491
	4781786	4781286	4781242	-	rplJ	PA4272	16722	15119
4781786			4781978	+	-	PA4272.1	12466	10951
	4782680	4781985	4781979	-	rplA	PA4273	8424	7239
4783140	4783111	4782680		-	rplK	PA4274	10958	9352
4783761	4783761	4783228	4783141	-	nusG	PA4275	2967	2996
4784183	4784139	4783771	4783762	-	secE	PA4276	3274	3601
4784259			4784184	-	-	PA4276.1	2313	2427
4785592	4785509	4784316	4784260	-	tufB	PA4277	10587	8292
4785668			4785593	-	-	PA4277.1	1913	3202
4785761			4785688	-	-	PA4277.2	2366	5004
4785872			4785788	-	-	PA4277.3	2597	6226
4786725	4786725	4786021	4785873	-	-	PA4278	80	67
	4787480	4786734	4786726	-	-	PA4279	73	67
4788420	4788415	4787477		-	birA	PA4280	131	120
4788693			4788574	-	-	PA4280.1	3	8
4791724			4788836	-	-	PA4280.2	54	52
4792026			4791951	-	-	PA4280.3	1251	1128
4792131			4792055	-	-	PA4280.4	1630	1780
4793731			4792196	-	-	PA4280.5	52	53
	4794404	4795633		+	sbcD	PA4281	33	24
4795634	4795642	4799277	4799298	+	-	PA4282	36	35

	4801464	4799299	4799299	-	recD	PA4283	48	48
	4805198	4801461		-	recB	PA4284	54	57
4808710	4808710	4805195		-	recC	PA4285	60	77
4809467	4809434	4808751	4808711	-	-	PA4286	82	99
	4810437	4809547		-	-	PA4287	6	6
	4810538	4811341		+	-	PA4288	11	7
	4811396	4812601		+	-	PA4289	24	6
	4814214	4812598		-	-	PA4290	72	3
	4814213	4814899	4814899	+	-	PA4291	69	125
4814900	4815043	4816512	4816512	+	-	PA4292	155	191
	4819348	4816580		-	pprA	PA4293	3	3
	4819864	4819358		-	-	PA4294	5	1
	4819928	4820410		+	fppA	PA4295	6	3
4820411	4820532	4821359	4821384	+	pprB	PA4296	34	32
	4823055	4821385		-	tadG	PA4297	10	6
	4823364	4823080		-	-	PA4298	2	0
	4824124	4823387		-	tadD	PA4299	4	1
	4825032	4824121		-	tadC	PA4300	2	2
	4825926	4825042		-	tadB	PA4301	4	3
	4827188	4825923		-	tadA	PA4302	4	3
	4828369	4827185		-	tadZ	PA4303	9	7
	4829629	4828379		-	rcpA	PA4304	5	4
	4830554	4829643		-	rcpC	PA4305	7	3
	4830964	4831182		+	flp	PA4306	23	8
4831312	4831372	4833270	4833331	+	pctC	PA4307	164	112
4834864	4834864	4833374	4833366	-	-	PA4308	40	32
4835193	4835264	4837153	4837207	+	pctA	PA4309	45	50
4837343	4837390	4839279	4839280	+	pctB	PA4310	148	153
	4839597	4840712		+	-	PA4311	10	7
	4840705	4841466		+	-	PA4312	40	52
	4841463	4842464	4842518	+	-	PA4313	61	75
4843565	4843552	4842701	4842647	-	purU1	PA4314	485	548

4843778	4843812	4844186	4844186	+	mvaT	PA4315	1848	1668
4845718	4845713	4844271	4844187	-	sbcB	PA4316	72	53
4845915	4845938	4846675	4846675	+	-	PA4317	381	430
4846676	4846713	4847510		+	-	PA4318	126	164
	4847507	4848487		+	-	PA4319	74	85
	4848474	4850045		+	-	PA4320	76	84
	4850042	4851313		+	-	PA4321	54	64
	4851310	4852317		+	-	PA4322	143	166
	4852314	4853645	4853645	+	-	PA4323	81	87
4854009	4854009	4853650	4853646	-	-	PA4324	110	97
4854492	4854492	4854070	4854020	-	-	PA4325	64	57
4854978	4854978	4854595	4854493	-	-	PA4326	39	31
	4855072	4855878		+	-	PA4327	16	13
4855948	4855963	4856877	4856959	+	-	PA4328	83	64
4856960	4856960	4858411	4858414	+	pykA	PA4329	286	223
4859328	4859263	4858490	4858422	-	-	PA4330	79	56
	4860255	4859329		-	-	PA4331	31	26
	4861711	4860248		-	-	PA4332	66	65
	4861653	4863176	4863180	+	-	PA4333	553	457
	4864529	4863273		-	-	PA4334	19	21
4864993	4864973	4864665	4864654	-	-	PA4335	85	70
4865603	4865578	4864994	4864994	-	-	PA4336	84	75
	4866293	4865682		-	-	PA4337	7	6
	4867667	4866387		-	-	PA4338	14	12
4868846	4868846	4867767		-	-	PA4339	24	18
4869503	4869493	4868921	4868847	-	-	PA4340	62	50
	4870328	4869558		-	-	PA4341	1	3
	4871912	4870428		-	-	PA4342	4	4
	4873232	4871913		-	-	PA4343	2	1
	4874490	4873270		-	-	PA4344	7	5
	4875245	4874655		-	-	PA4345	26	20
	4875641	4875306		-	-	PA4346	9	14

	4876792	4875638		-	-	PA4347	25	40
4877648	4877606	4876821	4876820	-	-	PA4348	577	563
	4878587	4877691		-	-	PA4349	16	15
	4878790	4879545		+	-	PA4350	16	39
	4879545	4880321		+	-	PA4351	42	99
4881199	4881178	4880318		-	-	PA4352	258	259
	4881900	4881328		-	-	PA4353	30	17
	4882053	4882355		+	-	PA4354	41	35
	4882404	4883570		+	-	PA4355	14	11
4883583	4883607	4884659	4884718	+	xenB	PA4356	104	67
	4884961	4884719		-	-	PA4357	24	80
4887261	4887261	4884961		-	-	PA4358	35	75
4887569	4887506	4887279	4887262	-	-	PA4359	77	128
4888589	4888195	4887722	4887722	-	-	PA4360	142	160
	4890101	4889112		-	-	PA4361	18	16
	4890286	4891344		+	-	PA4362	10	8
	4892247	4891345		-	iciA	PA4363	15	12
	4892345	4892746		+	-	PA4364	4	3
	4892748	4893350		+	-	PA4365	4	1
4893529	4893697	4894278	4894291	+	sodB	PA4366	3056	1753
	4894459	4896522		+	bifA	PA4367	36	27
4897425	4897425	4896523		-	-	PA4368	28	21
4898173	4897999	4897427	4897426	-	-	PA4369	61	33
4898176			4898191	?	-	predicted RN	303	425
4898192	4898193	4899533	4899548	+	icmP	PA4370	491	515
4899646	4899696	4901117	4901118	+	-	PA4371	63	91
4901127	4901127	4902191	4902196	+	-	PA4372	163	179
4902202	4902202	4903296	4903296	+	-	PA4373	98	118
4903347	4903466	4904596	4904646	+	-	PA4374	44	36
4904647	4904647	4907703		+	-	PA4375	24	23
4907741	4907841	4909037	4909037	+	pncB2	PA4376	54	51
	4909404	4909162	4909141	-	-	PA4377	50	38

4910107	4910105	4909401		-	inaA	PA4378	55	53
4910870	4910791	4910108	4910108	-	-	PA4379	49	50
	4912151	4910871		-	-	PA4380	22	23
4913040	4912824	4912141		-	-	PA4381	57	50
	4913041	4913835		+	-	PA4382	10	7
	4914510	4914127		-	-	PA4383	30	20
4914739	4914739	4915425	4915425	+	-	PA4384	31	24
4917174	4917124	4915481	4915452	-	groEL	PA4385	9871	8778
4917538	4917468	4917175	4917175	-	groES	PA4386	8192	7761
4917539			4917559	?	-	predicted RN	3998	4599
4918145	4918130	4917663	4917560	-	-	PA4387	92	90
4918936	4918933	4918199	4918158	-	-	PA4388	75	59
4918937	4919042	4919800	4919805	+	speA	PA4389	222	187
4920874	4920866	4919859	4919859	-	-	PA4390	120	136
	4921982	4920981		-	-	PA4391	20	18
	4921981	4922364		+	-	PA4392	35	26
4922365	4922407	4924191	4924246	+	ampG	PA4393	34	29
4925166	4925083	4924247	4924247	-	-	PA4394	182	162
4925831	4925795	4925316	4925235	-	-	PA4395	880	911
	4925899	4926999		+	-	PA4396	47	25
	4927036	4927947		+	panE	PA4397	14	11
	4927998	4930094	4930094	+	-	PA4398	19	19
4930095	4930099	4930677	4930705	+	-	PA4399	44	36
4931723	4931695	4930748	4930748	-	-	PA4400	109	68
4932381	4932381	4931746	4931746	-	-	PA4401	55	58
4933865	4933719	4932502	4932382	-	argJ	PA4402	102	124
4936650	4936616	4933866	4933866	-	secA	PA4403	537	540
4937675	4937673	4936750	4936667	-	-	PA4404	110	89
4937752	4937820	4938215	4938275	+	-	PA4405	99	124
4939193	4939187	4938276	4938276	-	lpxC	PA4406	1467	1919
4939277			4939194	-	-	PA4406.1	1177	1423
4940513	4940484	4939300	4939278	-	ftsZ	PA4407	948	1065

4940514			4940526	?	-	predicted RN	700	888
4941788	4941788	4940535	4940527	-	ftsA	PA4408	376	446
4942676	4942673	4941810	4941791	-	ftsQ	PA4409	167	217
	4943636	4942677	4942677	-	ddlB	PA4410	225	261
	4945075	4943633		-	murC	PA4411	225	326
	4946141	4945068		-	murG	PA4412	151	188
	4947330	4946131		-	ftsW	PA4413	104	135
4948676	4948676	4947330		-	murD	PA4414	186	256
	4949772	4948690	4948677	-	mraY	PA4415	186	248
	4951148	4949772		-	murF	PA4416	168	215
	4952604	4951141		-	murE	PA4417	197	266
	4954343	4952604		-	ftsI	PA4418	170	229
	4954633	4954340		-	ftsL	PA4419	192	238
4955573	4955571	4954630		-	-	PA4420	267	364
4956081	4956029	4955574	4955574	-	-	PA4421	459	590
4956328			4956536	+	rnpB	PA4421.1	87674	103075
4957595	4957582	4956734	4956537	-	-	PA4422	73	75
4957695	4957709	4959523		+	-	PA4423	169	161
	4959520	4959897	4959897	+	-	PA4424	153	205
4959898	4959926	4960519		+	-	PA4425	258	296
	4960516	4961094	4961094	+	-	PA4426	336	346
4961569	4961558	4961151	4961145	-	sspB	PA4427	337	315
4962272	4962187	4961570	4961570	-	sspA	PA4428	248	264
	4963055	4962273	4962273	-	-	PA4429	1623	1564
	4964266	4963055		-	-	PA4430	2084	1994
4964917	4964859	4964266		-	-	PA4431	1873	1911
4965506	4965501	4965109	4965086	-	rpsI	PA4432	8407	8325
4966067	4965944	4965516	4965507	-	rplM	PA4433	7616	7967
4966068			4966098	?	-	predicted RN	3988	5158
4966099	4966190	4967227	4967312	+	-	PA4434	65	63
	4968458	4967313		-	-	PA4435	23	18
4968459	4968712	4969611	4969769	+	-	PA4436	35	45

	4969770	4970732		+	-	PA4437	12	15
4971909	4971891	4970797	4970797	-	-	PA4438	285	306
4973342	4973332	4971986	4971981	-	trpS	PA4439	226	205
4974094	4974029	4973400	4973345	-	-	PA4440	105	74
4974120			4974147	?	-	predicted RN	234	448
4974148	4974174	4974620	4974645	+	-	PA4441	367	596
4976606	4976595	4974694	4974691	-	cysN	PA4442	120	208
4977551	4977524	4976607	4976607	-	cysD	PA4443	167	278
4977859	4977869	4978972		+	mltB1	PA4444	62	53
4979764	4979717	4978959		-	-	PA4445	84	81
4979812	4979834	4981003	4981009	+	algW	PA4446	66	105
4982103	4982101	4981046	4981036	-	hisC1	PA4447	110	101
4983551	4983426	4982104	4982104	-	hisD	PA4448	154	131
4984187	4984187	4983552	4983552	-	hisG	PA4449	410	410
4985490	4985470	4984205	4984188	-	murA	PA4450	679	648
4985750	4985730	4985491	4985491	-	-	PA4451	588	716
4985843			4985782	-	-	PA4451.1	70	79
	4986155	4985847	4985844	-	-	PA4452	359	444
4986809	4986799	4986152		-	-	PA4453	725	901
	4987284	4986811	4986811	-	-	PA4454	354	433
	4988082	4987285		-	-	PA4455	256	336
4988959	4988891	4988082		-	-	PA4456	377	539
4989303	4989305	4990285		+	-	PA4457	368	362
	4990285	4990824	4990832	+	-	PA4458	232	245
4990833	4990833	4991405		+	-	PA4459	365	389
	4991392	4991919		+	-	PA4460	708	783
	4991919	4992644	4992778	+	-	PA4461	428	466
4992781	4992870	4994363	4994363	+	rpoN	PA4462	302	318
4994364			4994397	?	-	predicted RN	1356	876
4994398	4994441	4994749	4994749	+	-	PA4463	1496	1101
4994757	4994763	4995227	4995228	+	ptsN	PA4464	490	504
4995229	4995229	4996089	4996118	+	-	PA4465	256	318

4996119	4996119	4996391	4996402	+	-	PA4466	179	237
	4997420	4996488		-	-	PA4467	9	9
	4998050	4997439		-	sodM	PA4468	13	9
	4998512	4998063		-	-	PA4469	9	6
	4999916	4998540		-	fumC1	PA4470	13	11
	5000304	4999909		-	-	PA4471	6	2
5001821	5001801	5000452	5000432	-	pmbA	PA4472	68	58
5001822	5001822	5002424	5002424	+	-	PA4473	166	189
5003897	5003897	5002455	5002425	-	-	PA4474	121	117
5004797	5004748	5003900	5003898	-	-	PA4475	77	82
5008628	5008628	5004798	5004798	-	-	PA4476	34	35
5010133	5010100	5008643	5008629	-	cafA	PA4477	95	75
5010749	5010739	5010134	5010134	-	-	PA4478	70	66
	5011322	5010828	5010828	-	mreD	PA4479	94	100
5012335	5012314	5011322		-	mreC	PA4480	151	172
5013465	5013431	5012394	5012345	-	mreB	PA4481	527	601
5013558	5013671	5013961	5013961	+	gatC	PA4482	358	332
5013962	5013974	5015428	5015494	+	gatA	PA4483	395	351
5015535	5015535	5016980	5016980	+	gatB	PA4484	455	420
5016982	5017040	5017417	5017417	+	-	PA4485	55	71
5017438	5017449	5017835	5017908	+	-	PA4486	73	86
5018755	5018751	5017960	5017960	-	-	PA4487	75	86
	5020405	5018756	5018756	-	-	PA4488	52	62
5024957	5024952	5020402		-	-	PA4489	104	124
	5025617	5024979	5024979	-	-	PA4490	99	121
5027383	5027383	5025614		-	-	PA4491	123	140
5028241	5028231	5027422	5027384	-	-	PA4492	123	141
5028979	5028954	5028394	5028377	-	roxR	PA4493	166	167
5030312	5030248	5028980	5028980	-	roxS	PA4494	85	80
5030507	5030525	5031235	5031235	+	-	PA4495	127	103
5031479	5031488	5033101	5033101	+	-	PA4496	50	139
5033159	5033194	5034792	5034792	+	-	PA4497	29	71

5036083	5036077	5034860	5034849	-	-	PA4498	148	346
5036238	5036245	5036808	5036831	+	-	PA4499	60	139
5036939	5037074	5038675	5038675	+	-	PA4500	222	2292
5038676	5038901	5040355	5040360	+	opdP	PA4501	30	713
5040361	5040405	5042000	5042000	+	-	PA4502	49	1121
5042001			5042063	?	-	predicted RN	73	1492
5042064	5042068	5043078	5043089	+	-	PA4503	52	1109
5043090	5043090	5044001	5044001	+	-	PA4504	25	721
5044006	5044054	5045028		+	-	PA4505	34	828
	5045028	5045999	5045999	+	-	PA4506	35	818
5046682	5046664	5046032	5046030	-	-	PA4507	55	128
	5046806	5047279		+	-	PA4508	23	29
	5048213	5047284		-	-	PA4509	17	16
	5048887	5048210		-	-	PA4510	26	23
5049752	5049639	5048884		-	-	PA4511	38	32
5049760	5049769	5050668	5050668	+	lpxO1	PA4512	151	92
5053615	5053400	5050848	5050669	-	-	PA4513	24	38
5055890	5055877	5053616	5053616	-	-	PA4514	68	124
5056089	5056095	5056775	5056777	+	-	PA4515	40	68
5056778	5056778	5057593	5057715	+	-	PA4516	50	62
	5057716	5059518		+	-	PA4517	22	21
	5059999	5059574		-	-	PA4518	30	30
5061533	5061278	5060115	5060110	-	speC	PA4519	239	330
	5063751	5061730		-	-	PA4520	26	32
	5064777	5063941		-	-	PA4521	31	24
	5065340	5064774		-	ampD	PA4522	38	24
5068031	5067746	5065491	5065354	-	-	PA4523	125	41
5068032	5068032	5068880	5068891	+	nadC	PA4524	72	67
5069007			5068932	-	-	PA4524.1	2151	1807
5069541	5069531	5069082	5069008	-	pilA	PA4525	6412	5365
5069542			5069557	?	-	predicted RN	1712	1370
5069756	5069763	5071463	5071976	+	pilB	PA4526	188	109

5071991			5072168	?	-	predicted RN	528	105
	5072695	5073567		+	pilD	PA4528	136	38
	5073564	5074175		+	coaE	PA4529	155	85
	5074172	5074372	5074372	+	-	PA4530	113	60
5074623	5074618	5074409	5074373	-	-	PA4531	73	65
	5075413	5074724	5074646	-	-	PA4532	31	40
	5075880	5075410		-	-	PA4533	49	57
5076315	5076302	5075877		-	-	PA4534	60	80
5076403	5076435	5077064		+	-	PA4535	61	30
	5077061	5077510		+	-	PA4536	25	16
5077530	5077536	5077706	5077706	+	-	PA4537	90	86
5077765	5077770	5079077	5079077	+	ndh	PA4538	117	74
5080442	5080208	5079117	5079078	-	-	PA4539	65	76
	5080757	5082394		+	-	PA4540	3	2
	5082443	5086696		+	-	PA4541	13	10
5087000			5086925	-	-	PA4541.1	72	264
5087087			5087011	-	-	PA4541.2	289	1180
5087172			5087097	-	-	PA4541.3	455	2133
5089972	5089972	5087408	5087391	-	clpB	PA4542	269	317
	5090856	5090128	5090094	-	-	PA4543	74	87
5091889	5091815	5090853		-	rluD	PA4544	169	201
5091916	5091961	5092986	5092986	+	comL	PA4545	226	272
5092987	5093378	5094970	5094970	+	pilS	PA4546	37	43
5094971	5094985	5096322	5096322	+	pilR	PA4547	68	64
5097550	5097462	5096368	5096323	-	-	PA4548	96	49
	5097597	5098106		+	fimT	PA4549	5	3
	5098212	5098718		+	fimU	PA4550	169	35
	5098709	5099266		+	pilV	PA4551	84	21
	5099263	5100087		+	pilW	PA4552	107	28
	5100084	5100671	5100671	+	pilX	PA4553	102	31
5100678	5100683	5104168	5104169	+	pilY1	PA4554	143	44
5104170	5104170	5104517		+	pilY2	PA4555	152	53

	5104514	5104939	5104958	+	pilE	PA4556	114	44
5105968	5105930	5104986	5104982	-	lytB	PA4557	185	248
	5106456	5106016	5106016	-	-	PA4558	346	400
	5106958	5106449		-	lspA	PA4559	348	404
5109805	5109782	5106951		-	ileS	PA4560	382	417
5110747	5110744	5109806	5109806	-	ribF	PA4561	198	239
5112512	5112379	5110841	5110748	-	-	PA4562	32	39
5112632			5112654	?	-	predicted RN	801	1442
5112655	5112663	5112938	5112966	+	rpsT	PA4563	2996	4136
5113469	5113469	5113005	5113002	-	-	PA4564	151	170
5114669	5114599	5113481	5113480	-	proB	PA4565	252	264
5115946	5115890	5114670	5114670	-	obg	PA4566	301	272
5116012			5116031	?	-	predicted RN	536	622
5116289	5116289	5116032	5116032	-	rpmA	PA4567	6443	5777
5116803	5116624	5116313	5116290	-	rplU	PA4568	8471	8037
5116855	5116865	5117833	5117843	+	ispB	PA4569	259	301
	5117971	5118195		+	-	PA4570	11	9
5118462	5118538	5120565	5120622	+	-	PA4571	125	176
5121254	5121252	5120635	5120623	-	fklB	PA4572	322	248
5121893	5121642	5121337	5121260	-	-	PA4573	34	49
5122398	5122387	5121899	5121898	-	-	PA4574	178	289
	5122902	5122561		-	-	PA4575	35	26
5125508	5125506	5123053	5123037	-	-	PA4576	171	162
5125975	5125931	5125605	5125531	-	-	PA4577	189	95
5126137	5126166	5126654	5126655	+	-	PA4578	1179	1002
5126656	5126713	5128569		+	-	PA4579	90	72
5129172	5129117	5128563		-	-	PA4580	71	63
	5130768	5129173		-	rtcR	PA4581	25	20
5131082			5131155	+	-	PA4581.1	217	216
5131156	5131428	5132573	5132628	+	-	PA4582	32	27
	5132629	5133843	5133843	+	-	PA4583	40	29
5133844	5133880	5134692		+	-	PA4584	40	34

	5134689	5135714	5135815	+	rtcA	PA4585	41	37
	5135816	5136193		+	-	PA4586	22	25
5136295	5136317	5137357	5137377	+	ccpR	PA4587	45	58
5138816	5138754	5137417	5137417	-	gdhA	PA4588	52	39
	5140441	5139050		-	-	PA4589	7	10
	5141189	5140701		-	pra	PA4590	23	22
	5143063	5141786		-	-	PA4591	8	8
	5144534	5143053		-	-	PA4592	11	11
	5145720	5144527		-	-	PA4593	11	9
	5146403	5145717		-	-	PA4594	19	13
5148578	5148571	5146907	5146902	-	-	PA4595	536	434
	5149064	5149582		+	-	PA4596	38	27
	5151072	5149633		-	oprJ	PA4597	10	12
	5154209	5151078		-	mexD	PA4598	11	8
	5155400	5154237		-	mexC	PA4599	9	5
5155548	5155561	5156124	5156367	+	nfxB	PA4600	51	42
	5156368	5160615		+	morA	PA4601	25	25
5160717	5160738	5161991	5161994	+	glyA3	PA4602	748	751
5162457	5162449	5162069	5161995	-	-	PA4603	38	17
5163534	5163476	5162472	5162458	-	-	PA4604	66	28
5163782	5163739	5163536	5163536	-	-	PA4605	275	109
5165869	5165854	5163788	5163788	-	-	PA4606	363	127
5166697	5166697	5166191	5166167	-	-	PA4607	135	78
5166698			5166710	?	-	predicted RN	98	63
5166809	5166900	5167277	5167277	+	-	PA4608	75	54
5168656	5168645	5167284	5167278	-	radA	PA4609	66	75
	5168755	5169189		+	-	PA4610	47	28
5169512	5169505	5169251	5169245	-	-	PA4611	290	146
	5170131	5169580		-	-	PA4612	11	8
5171764	5171726	5170185	5170132	-	katB	PA4613	15	24
5172123	5172253	5172666	5172666	+	mscL	PA4614	286	186
5173612	5173547	5172771	5172718	-	-	PA4615	88	54

	5174652	5173654		-	-	PA4616	79	10
5174653	5174980	5176104	5176104	+	-	PA4617	45	44
5177651	5177651	5176680	5176105	-	-	PA4618	95	53
5178902	5178902	5177655	5177652	-	-	PA4619	102	43
	5179444	5178905	5178903	-	-	PA4620	124	37
5182363	5182268	5179437		-	-	PA4621	107	37
	5182579	5183790		+	-	PA4622	14	14
	5184339	5183950		-	-	PA4623	9	10
	5186348	5184642		-	-	PA4624	12	17
5192875	5192875	5186411		-	-	PA4625	11	21
	5193196	5194167		+	hprA	PA4626	31	24
5194168	5194277	5195275	5195291	+	-	PA4627	73	64
5195370	5195370	5196833	5196878	+	lysP	PA4628	108	90
5197186	5197186	5197764		+	-	PA4629	26	32
5197875	5197881	5198324	5198325	+	-	PA4630	57	70
5199501	5199359	5198334	5198334	-	-	PA4631	124	158
5200331	5200324	5199503	5199502	-	-	PA4632	360	390
5200438	5200487	5202625	5202625	+	-	PA4633	61	59
5203313	5203242	5202628	5202626	-	-	PA4634	59	55
	5203668	5204372		+	-	PA4635	11	7
5204894	5204928	5206088	5206090	+	-	PA4636	150	199
5206566	5206487	5206209	5206147	-	-	PA4637	152	167
5207034	5207007	5206720	5206569	-	-	PA4638	47	51
5207631	5207622	5207035	5207035	-	-	PA4639	250	338
5207907	5208464	5209987	5210166	+	mqoB	PA4640	400	279
5210996	5210996	5210706	5210683	-	-	PA4642	117	162
5211567	5211564	5211079	5211026	-	-	PA4643	68	68
5212106	5212105	5211632	5211632	-	-	PA4644	155	115
5212675	5212664	5212107	5212107	-	-	PA4645	289	231
5212822	5212833	5213471	5213473	+	upp	PA4646	146	141
5213474	5213474	5214757	5214763	+	uraA	PA4647	122	110
	5215091	5215639		+	-	PA4648	6	7

	5215670	5216203		+	-	PA4649	6	7
	5216203	5216745		+	-	PA4650	3	4
	5216764	5217552		+	-	PA4651	4	6
	5217569	5219941		+	-	PA4652	3	3
	5219938	5220885		+	-	PA4653	8	5
	5222260	5220887		-	-	PA4654	5	1
	5223562	5222540	5222417	-	hemH	PA4655	75	70
5224889	5224476	5223559		-	-	PA4656	40	39
5224890	5224890	5225873	5225883	+	-	PA4657	44	35
	5226026	5226982		+	-	PA4658	20	19
	5226992	5227891		+	-	PA4659	18	18
	5227888	5229333	5229455	+	phr	PA4660	77	59
5230006	5229980	5229459	5229459	-	pagL	PA4661	1532	1218
	5230911	5230114	5230012	-	murI	PA4662	117	156
	5231659	5230901		-	moeB	PA4663	158	190
5232484	5232483	5231653		-	hemK	PA4664	198	232
5233584	5233567	5232485	5232485	-	prfA	PA4665	227	244
5234865	5234853	5233585	5233585	-	hemA	PA4666	167	224
5234997	5234997	5236769	5236772	+	-	PA4667	134	137
5236774	5236774	5237391	5237392	+	-	PA4668	95	90
5237393	5237393	5238241	5238276	+	ipk	PA4669	1202	2660
5238277			5238351	+	-	PA4669.1	2877	5950
5238352	5238408	5239349	5239357	+	prs	PA4670	1556	2660
5239361	5239466	5240080	5240095	+	-	PA4671	5625	5554
5240096			5240111	?	-	predicted RN	3337	3108
5240112	5240122	5240706	5240746	+	-	PA4672	367	491
5240747	5240747	5241847	5241858	+	-	PA4673	392	523
5242026			5242102	+	-	PA4673.1	0	13
5242849	5242559	5242254	5242249	-	-	PA4674	83	116
5243163	5243178	5245406	5245406	+	-	PA4675	169	157
5246125	5246123	5245476	5245407	-	-	PA4676	200	178
5247423	5247423	5246185	5246137	-	-	PA4677	41	39

	5248074	5247622	5247424	-	rimI	PA4678	43	53
5248950	5248772	5248071		-	-	PA4679	71	70
	5249568	5249032		-	-	PA4680	4	2
	5250618	5249599		-	-	PA4681	4	4
	5251666	5250620		-	-	PA4682	7	5
	5252473	5251871		-	-	PA4683	27	27
5252756	5252758	5254056		+	-	PA4684	113	141
	5254046	5254741		+	-	PA4685	12	148
	5254738	5257584	5257584	+	-	PA4686	160	164
5257687	5257696	5258703	5258703	+	hitA	PA4687	260	270
5258704	5258724	5260262	5260262	+	hitB	PA4688	82	96
5263928	5262649	5260343	5260276	-	-	PA4689	55	55
5264219			5264100	-	-	PA4690.1	2	3
5267252			5264362	-	-	PA4690.2	59	54
5267554			5267479	-	-	PA4690.3	1093	1194
5267659			5267583	-	-	PA4690.4	1680	1738
5269259			5267724	-	-	PA4690.5	48	54
	5270411	5269803		-	-	PA4691	18	17
	5271424	5270411		-	-	PA4692	24	20
5272365	5272300	5271485	5271425	-	pssA	PA4693	345	386
5273513	5273471	5272455	5272438	-	ilvC	PA4694	757	554
5274005	5274005	5273514	5273514	-	ilvH	PA4695	374	339
5275977	5275732	5274008	5274006	-	ilvI	PA4696	331	290
5276282	5276283	5276735	5276782	+	-	PA4697	169	73
	5277172	5276843	5276836	-	-	PA4698	171	197
5277967	5277951	5277172		-	-	PA4699	173	178
5280292	5280292	5277968	5277968	-	mrcB	PA4700	90	116
5280503	5280505	5282067	5282070	+	-	PA4701	148	99
	5282159	5282506		+	-	PA4702	12	15
	5282732	5283004		+	-	PA4703	9	7
	5283109	5283906		+	cbpA	PA4704	44	14
5283960			5284110	+	prrF1	PA4704.1	30	29

5284172			5284319	+	prrF2	PA4704.2	17	19
5285257	5285257	5284370	5284320	-	-	PA4705	29	29
	5286035	5285268	5285258	-	-	PA4706	23	25
	5287018	5286035		-	-	PA4707	15	19
	5287962	5287069		-	phuT	PA4708	12	14
	5289036	5287972		-	-	PA4709	9	10
5289178	5289217	5291511	5291613	+	phuR	PA4710	10	8
5291614	5291614	5291961	5292041	+	-	PA4711	48	57
	5292042	5292554		+	-	PA4712	16	18
	5293007	5292558		-	-	PA4713	13	11
5293247	5293257	5293706	5293716	+	-	PA4714	73	56
5295005	5295000	5293765	5293765	-	-	PA4715	114	156
	5295170	5296024		+	-	PA4716	34	23
5296050	5296092	5296985	5296991	+	-	PA4717	60	64
5297610	5297483	5297007	5296992	-	-	PA4718	82	93
5297732	5297897	5299192		+	-	PA4719	122	126
	5299189	5300280	5300280	+	trmA	PA4720	165	146
	5301021	5300314	5300281	-	-	PA4721	62	70
5302336	5302193	5301021		-	-	PA4722	69	61
5302337			5302376	?	-	predicted RN	460	654
5302377	5302386	5302832	5302832	+	dksA	PA4723	941	908
5302833	5302900	5303781	5303850	+	-	PA4724	65	57
5303851	5303851	5304027		+	-	PA4724.1	42	49
	5304011	5306962		+	cbrA	PA4725	29	26
5306989	5306989	5308425	5308432	+	cbrB	PA4726	81	64
5308433			5308493	+	-	PA4726.1	41	33
5308587			5308993	+	crcZ	PA4726.11	21454	72
5308964			5308743	-	-	PA4726.2	25151	88
5309053	5309326	5310729		+	pcnB	PA4727	183	305
	5310726	5311214	5311238	+	folK	PA4728	90	142
5311357	5311464	5312264		+	panB	PA4729	236	266
	5312261	5313112	5313112	+	panC	PA4730	166	190

5313113	5313206	5313586	5313592	+	panD	PA4731	129	138
5313663	5313676	5315340	5315340	+	pgi	PA4732	184	134
5315482	5315534	5317471	5317557	+	acsB	PA4733	32	31
5317565	5317569	5318450	5318556	+	-	PA4734	62	46
5318557	5318575	5321841	5321841	+	-	PA4735	62	61
5321842	5321846	5322139	5322205	+	-	PA4736	131	118
5322235	5322235	5322450	5322457	+	-	PA4737	72	94
5322729	5322707	5322510	5322503	-	-	PA4738	71	42
5323138	5323101	5322757	5322757	-	-	PA4739	62	38
5325644	5325479	5323374	5323374	-	pnp	PA4740	1060	815
5326009	5325922	5325653	5325645	-	rpsO	PA4741	10671	9903
5326932	5326932	5326018	5326013	-	truB	PA4742	382	385
5327426	5327324	5326935	5326933	-	rbfA	PA4743	320	302
5329976	5329949	5327427	5327427	-	infB	PA4744	1287	1066
5331458	5331458	5329977	5329977	-	nusA	PA4745	1034	924
5332086	5331961	5331503	5331459	-	-	PA4746	322	484
5332163			5332087	-	-	PA4746.1	507	1032
5332339			5332254	-	-	PA4746.2	1214	1701
5332743	5332743	5332354	5332340	-	secG	PA4747	1497	1929
5333502	5333501	5332746	5332744	-	tpiA	PA4748	1441	2191
5334920	5334904	5333567	5333506	-	glmM	PA4749	399	418
5335781	5335772	5334921	5334921	-	folP	PA4750	177	228
5337762	5337701	5335782	5335782	-	ftsH	PA4751	401	385
5338598	5338523	5337900	5337900	-	ftsJ	PA4752	142	156
5338605	5338618	5338932	5338933	+	-	PA4753	515	448
5339390	5339380	5338973	5338934	-	-	PA4754	316	263
	5339867	5339391	5339391	-	greA	PA4755	698	594
5343085	5343085	5339864		-	carB	PA4756	402	339
5343755	5343755	5343105	5343086	-	-	PA4757	206	224
5344932	5344903	5343767	5343756	-	carA	PA4758	356	355
5345060			5344950	-	-	PA4758.1	129	120
5345892	5345892	5345086	5345061	-	dapB	PA4759	333	387

5347180	5347082	5345949	5345893	-	dnaJ	PA4760	464	475
5349154	5349111	5347198	5347182	-	dnaK	PA4761	1769	1648
5349155			5349178	?	-	predicted RN	1364	1312
5349810	5349761	5349201	5349179	-	grpE	PA4762	799	811
5349838	5349929	5351605	5351674	+	recN	PA4763	67	38
5352108	5352079	5351675	5351675	-	fur	PA4764	808	638
5352109			5352123	?	-	predicted RN	608	820
5352124	5352177	5352707	5352707	+	omlA	PA4765	531	615
	5353080	5352775	5352767	-	-	PA4766	91	112
5353668	5353507	5353073		-	-	PA4767	187	198
5353783	5353783	5354262	5354284	+	smpB	PA4768	408	471
5355058	5355058	5354285	5354285	-	-	PA4769	165	116
5355075	5355387	5357075	5357075	+	lldP	PA4770	95	28
5357082	5357230	5358375	5358441	+	lldD	PA4771	68	31
5358442	5358442	5361258		+	-	PA4772	45	23
5361562	5361586	5362068	5362068	+	-	PA4773	207	199
5362093	5362146	5363195	5363197	+	-	PA4774	130	107
5363198	5363198	5364058	5364064	+	-	PA4775	64	64
5364065	5364071	5364736	5364759	+	pmrA	PA4776	45	41
	5364760	5366193		+	pmrB	PA4777	20	21
5366252	5366257	5366655	5366655	+	cueR	PA4778	238	225
	5368080	5367187		-	-	PA4779	17	16
5369154	5369063	5368170	5368081	-	-	PA4780	56	39
	5370368	5369187		-	-	PA4781	12	10
5370721	5370721	5370476	5370369	-	-	PA4782	67	84
5371646	5371645	5370755	5370722	-	-	PA4783	33	53
5371647	5371800	5372267	5372590	+	-	PA4784	32	28
	5373868	5372591		-	-	PA4785	6	6
	5374123	5375478		+	-	PA4786	16	11
5376626	5376591	5375590	5375479	-	-	PA4787	111	103
	5376852	5377709		+	-	PA4788	2	2
5377776	5377791	5378096		+	-	PA4789	60	54

	5378093	5378842	5378907	+	-	PA4790	36	41
	5378908	5379528		+	-	PA4791	27	27
	5380448	5379513		-	-	PA4792	13	14
5380572	5380580	5381143	5381144	+	-	PA4793	107	48
	5381145	5381627		+	-	PA4794	41	39
	5381617	5381994		+	-	PA4795	47	76
	5381991	5382458	5382795	+	-	PA4796	48	72
	5383812	5382796		-	-	PA4797	18	16
	5383947	5384675		+	-	PA4798	29	28
	5385276	5384698		-	-	PA4799	13	11
	5386073	5385273		-	-	PA4800	14	18
5386844	5386737	5386369	5386074	-	-	PA4801	37	40
	5387000	5387722		+	-	PA4802	12	11
5387857			5387766	-	-	PA4802.1	56	51
	5388607	5387990		-	-	PA4803	28	14
	5390099	5388702		-	-	PA4804	15	8
	5391554	5390148		-	-	PA4805	3	3
	5391836	5392519		+	-	PA4806	19	18
	5394448	5392523		-	selB	PA4807	35	37
5395870	5395851	5394445		-	selA	PA4808	62	46
	5396859	5395930		-	fdhE	PA4809	26	30
	5397618	5396992	5396992	-	fdnI	PA4810	76	72
5398619	5398619	5397690	5397690	-	fdnH	PA4811	72	81
5401806	5401708	5398628	5398620	-	fdnG	PA4812	179	166
	5402945	5402016		-	lipC	PA4813	2	3
	5403305	5405350		+	fadH2	PA4814	1	1
5405490	5405559	5406008	5406164	+	-	PA4815	45	39
	5406165	5407400		+	-	PA4816	18	15
	5407376	5407870		+	-	PA4817	9	9
	5409309	5407873		-	-	PA4818	4	3
	5410280	5409306		-	-	PA4819	2	2
	5410639	5410277		-	-	PA4820	4	1

	5410876	5412237		+	-	PA4821	18	14
	5414236	5412569		-	-	PA4822	7	4
	5414488	5414279		-	-	PA4823	0	1
	5415315	5414539		-	-	PA4824	2	2
	5418124	5415413		-	mgtA	PA4825	6	2
5418863	5418569	5418351	5418295	-	-	PA4826	26	28
	5419738	5418899		-	-	PA4827	21	14
	5419863	5420318		+	-	PA4828	7	7
	5421726	5420323		-	lpd3	PA4829	13	9
	5421899	5422438		+	-	PA4830	14	16
5422468	5422506	5423066	5423070	+	-	PA4831	44	39
	5423868	5423071		-	-	PA4832	17	14
	5424099	5424716		+	-	PA4833	34	24
	5425615	5424761		-	-	PA4834	5	5
	5426898	5425597		-	-	PA4835	6	3
	5427686	5426895		-	-	PA4836	5	3
	5429842	5427716		-	-	PA4837	5	2
	5429984	5431138		+	-	PA4838	18	15
5433291	5433291	5431381	5431350	-	speA	PA4839	125	202
5433971	5433912	5433541	5433292	-	-	PA4840	100	239
5434652	5434652	5434116	5434016	-	-	PA4841	81	74
5435880	5435726	5434656	5434653	-	-	PA4842	164	136
5436042	5436079	5437707	5437707	+	-	PA4843	99	113
	5437785	5439758		+	-	PA4844	7	8
5439759	5439918	5441693	5441826	+	dipZ	PA4845	47	43
5441837	5441837	5442280	5442280	+	aroQ1	PA4846	387	614
5442281	5442304	5442774	5442774	+	accB	PA4847	1283	1393
5442775	5442792	5444141	5444155	+	accC	PA4848	1171	1334
	5444252	5445118		+	-	PA4849	19	29
5445178	5445199	5446083	5446083	+	prmA	PA4850	111	127
5446084	5446179	5447444	5447500	+	-	PA4851	123	130
5447628	5447648	5448646		+	-	PA4852	400	525

	5448643	5448966	5448966	+	fis	PA4853	528	559
5448967	5449046	5450653	5450653	+	purH	PA4854	314	323
5450654	5450757	5452046	5452053	+	purD	PA4855	215	249
5452054	5452150	5454978	5455035	+	retS	PA4856	71	80
	5455434	5456027		+	-	PA4857	20	16
	5456452	5457717		+	-	PA4858	6	6
	5458500	5459453		+	-	PA4859	2	0
	5459450	5460529		+	-	PA4860	2	1
	5460526	5461383		+	-	PA4861	4	1
	5461520	5462218		+	-	PA4862	5	4
	5462302	5462754		+	-	PA4863	33	28
	5462763	5463605		+	ureD	PA4864	17	13
	5463607	5463909	5463909	+	ureA	PA4865	24	14
	5463918	5464436	5464436	+	-	PA4866	25	20
	5464453	5464758		+	ureB	PA4867	12	18
	5464821	5466521	5466521	+	ureC	PA4868	23	22
5466542	5466790	5468007	5468007	+	-	PA4869	39	31
5468388	5468283	5468017	5468008	-	-	PA4870	43	30
5468409	5468409	5469023		+	-	PA4871	43	31
5469992	5469978	5469115	5469106	-	-	PA4872	85	53
5470177	5470186	5471451	5471482	+	-	PA4873	59	45
5472063	5472042	5471626	5471606	-	-	PA4874	112	74
	5472439	5472735		+	-	PA4875	16	15
5472736	5472824	5473168	5473211	+	osmE	PA4876	59	42
	5473619	5473212		-	-	PA4877	9	8
	5473766	5474578		+	-	PA4878	26	18
	5476649	5474580		-	-	PA4879	15	13
	5476945	5477478		+	-	PA4880	39	29
	5477754	5478095		+	-	PA4881	10	11
	5478976	5478209		-	-	PA4882	7	2
	5479587	5478973		-	-	PA4883	0	0
	5480264	5479641		-	-	PA4884	2	3

	5480402	5481091		+	irlR	PA4885	34	19
	5481070	5482461		+	-	PA4886	22	24
5483770	5483768	5482452		-	-	PA4887	36	63
	5485100	5483988	5483839	-	desB	PA4888	72	26
5486211	5486197	5485097		-	-	PA4889	103	38
5486296	5486356	5486985	5487002	+	desT	PA4890	75	54
	5487215	5487718		+	ureE	PA4891	17	8
	5487715	5488386		+	ureF	PA4892	6	6
	5488397	5489011		+	ureG	PA4893	11	7
	5489041	5489613		+	-	PA4894	9	3
	5490652	5489630		-	-	PA4895	5	5
	5491181	5490645		-	-	PA4896	6	7
	5491346	5494315		+	-	PA4897	6	6
	5495713	5494460		-	opdK	PA4898	2	2
	5497290	5495821		-	-	PA4899	5	8
	5498653	5497313		-	-	PA4900	3	2
	5500326	5498740		-	mdlC	PA4901	4	3
	5500427	5501323		+	-	PA4902	13	11
	5502672	5501338		-	-	PA4903	3	3
	5503051	5504106		+	vanA	PA4904	7	3
	5504121	5505074		+	vanB	PA4905	7	6
5505847	5505784	5505071		-	-	PA4906	79	66
5506622	5506609	5505848	5505848	-	-	PA4907	167	183
	5507898	5506966		-	-	PA4908	2	3
	5508685	5507969		-	-	PA4909	8	7
	5509554	5508682		-	-	PA4910	1	2
	5510828	5509551		-	-	PA4911	1	1
	5511753	5510839		-	-	PA4912	7	4
	5513112	5511988		-	-	PA4913	6	6
	5513732	5514670		+	-	PA4914	15	7
	5514727	5516352		+	-	PA4915	19	13
5517094	5517094	5516399	5516353	-	-	PA4916	62	29

5517739	5517711	5517109	5517095	-	-	PA4917	113	25
5517804	5517821	5518480	5518480	+	-	PA4918	647	61
5518481	5518483	5519682	5519682	+	pncB1	PA4919	244	82
5519683	5519711	5520538	5520557	+	nadE	PA4920	263	152
	5520672	5521595		+	-	PA4921	43	30
5522238	5522110	5521664	5521645	-	azu	PA4922	1953	2298
5522374	5522386	5522973	5522973	+	-	PA4923	153	139
5523712	5523681	5522986	5522981	-	-	PA4924	79	63
	5524719	5523868		-	-	PA4925	8	9
	5525905	5524970		-	-	PA4926	14	11
	5528397	5525905		-	-	PA4927	8	8
5530934	5530902	5528659	5528650	-	-	PA4928	105	102
	5530973	5533015		+	-	PA4929	17	16
5534161	5534093	5533017	5533016	-	alr	PA4930	88	86
5535684	5535556	5534162	5534162	-	dnaB	PA4931	170	169
5536152	5536131	5535685	5535685	-	rplI	PA4932	5390	4471
5537037	5537022	5536153	5536153	-	-	PA4933	4475	3669
5537289	5537289	5537059	5537043	-	rpsR	PA4934	8367	6464
5537835	5537738	5537319	5537290	-	rpsF	PA4935	9534	7714
	5538699	5537953	5537951	-	-	PA4936	128	116
5541484	5541410	5538696		-	rnr	PA4937	190	139
5541614			5541700	+	-	PA4937.1	856	2405
5541830			5541916	+	-	PA4937.2	453	1173
5543365	5543365	5542073	5542045	-	purA	PA4938	627	601
5543366			5543415	?	-	predicted RN	436	449
5544601	5544601	5543417	5543416	-	-	PA4939	276	303
5544848	5544821	5544636	5544602	-	-	PA4940	331	444
	5545786	5544917	5544917	-	hflC	PA4941	746	741
5547082	5546988	5545786		-	hflK	PA4942	788	814
5548385	5548384	5547083	5547083	-	-	PA4943	787	780
5548659	5548645	5548397	5548395	-	hfq	PA4944	3127	2659
5548660			5548711	?	-	predicted RN	1557	1598

5549722	5549722	5548751	5548712	-	miaA	PA4945	422	395
	5551681	5549780	5549723	-	mutL	PA4946	186	206
5553116	5553108	5551681		-	amiB	PA4947	142	171
	5553584	5553117	5553117	-	-	PA4948	83	89
5555080	5555080	5553572		-	-	PA4949	75	81
5555137	5555162	5556247	5556247	+	-	PA4950	81	72
5556826	5556823	5556281	5556248	-	orn	PA4951	183	149
5556935	5556935	5557954	5557957	+	-	PA4952	127	128
5559020	5559001	5557958	5557958	-	motB	PA4953	94	83
5559875	5559872	5559021	5559021	-	motA	PA4954	132	110
	5560013	5561524		+	-	PA4955	42	31
5561594	5561599	5562414	5562417	+	rhdA	PA4956	98	107
5562418	5562418	5563287	5563287	+	psd	PA4957	143	142
5563964	5563965	5565428	5565469	+	-	PA4958	90	49
5565475	5565493	5567568	5567599	+	fimX	PA4959	154	138
5568944	5568944	5567655	5567655	-	-	PA4960	217	207
5569090	5569090	5570628		+	-	PA4961	137	117
	5570625	5571161		+	-	PA4962	33	31
5571929	5571929	5571219	5571206	-	-	PA4963	176	219
5574486	5574486	5572222	5572142	-	parC	PA4964	192	142
	5575018	5574494	5574487	-	-	PA4965	106	87
	5576028	5575015		-	-	PA4966	120	86
5577917	5577917	5576028		-	parE	PA4967	231	185
5578560	5578549	5577929	5577918	-	-	PA4968	113	78
5579499	5579499	5578681	5578564	-	cpdA	PA4969	122	62
	5580111	5579653	5579643	-	-	PA4970	118	111
5580740	5580719	5580102		-	aspP	PA4971	242	205
5580885	5580962	5581708	5581714	+	-	PA4972	91	100
	5583645	5581762		-	thiC	PA4973	37	34
5584101	5584101	5585549	5585549	+	-	PA4974	201	179
5586369	5586319	5585615	5585550	-	-	PA4975	34	61
	5587551	5586370		-	aruH	PA4976	16	34

	5589253	5587574		-	aruI	PA4977	6	21
	5591385	5589295		-	-	PA4978	6	27
	5592612	5591452		-	-	PA4979	7	36
5593456	5593424	5592633	5592613	-	-	PA4980	12	25
	5595095	5593683		-	-	PA4981	4	5
	5598275	5595279		-	-	PA4982	5	10
5599104	5599042	5598308	5598284	-	-	PA4983	38	44
	5599807	5599160	5599160	-	-	PA4984	31	18
	5600975	5599884		-	-	PA4985	16	9
	5601149	5603095		+	-	PA4986	14	9
5603096	5603173	5603772	5603787	+	-	PA4987	47	47
5605133	5605098	5603821	5603821	-	waaA	PA4988	55	52
	5606018	5605134		-	-	PA4989	14	9
	5606103	5606435		+	-	PA4990	19	8
5606482	5606496	5607671		+	-	PA4991	100	80
	5607668	5608480	5608648	+	-	PA4992	81	71
	5608649	5609569		+	-	PA4993	26	20
	5610802	5609594		-	-	PA4994	6	6
	5612140	5610851		-	-	PA4995	17	12
5613693	5613692	5612268	5612141	-	rfaE	PA4996	235	183
5615544	5615544	5613733	5613706	-	msbA	PA4997	192	168
5615647	5615652	5616302	5616309	+	-	PA4998	175	140
5616310	5616310	5617515	5617533	+	waaL	PA4999	102	100
5618491	5618418	5617534	5617534	-	wapR	PA5000	271	278
5619607	5619535	5618579	5618570	-	-	PA5001	195	215
5621026	5621026	5619608	5619608	-	-	PA5002	122	138
	5621926	5621030	5621027	-	-	PA5003	190	190
	5623056	5621920		-	-	PA5004	349	334
5624797	5624797	5623040		-	-	PA5005	491	444
	5626379	5624901	5624806	-	-	PA5006	115	132
	5627134	5626376		-	-	PA5007	122	128
	5627865	5627131		-	-	PA5008	133	139

	5628671	5627865		-	waaP	PA5009	127	141
	5629789	5628668		-	waaG	PA5010	198	202
	5630853	5629786		-	waaC	PA5011	150	163
5631930	5631887	5630850		-	waaF	PA5012	200	196
5632910	5632870	5631947	5631938	-	ilvE	PA5013	579	533
5632911			5632925	?	-	predicted RN	503	533
5636092	5635874	5632926	5632926	-	glnE	PA5014	95	100
5636101	5636156	5638804	5638804	+	aceE	PA5015	1149	1626
5638805	5638949	5640592	5640592	+	aceF	PA5016	825	1284
5640864	5641010	5643709	5643789	+	-	PA5017	50	42
5643803	5643803	5644450	5644450	+	msrA	PA5018	129	81
5645383	5645290	5644454	5644451	-	-	PA5019	122	118
	5645593	5647395		+	-	PA5020	18	5
5647604	5647814	5649430	5649450	+	-	PA5021	48	46
5649473	5649522	5652878	5652885	+	-	PA5022	54	56
	5652945	5654405		+	-	PA5023	19	10
5654406	5654811	5655527	5655614	+	-	PA5024	9	28
	5655648	5656925		+	metY	PA5025	30	25
	5657016	5657468		+	-	PA5026	11	11
5657561	5657581	5658396	5658411	+	-	PA5027	141	58
5659197	5659185	5658418	5658416	-	-	PA5028	163	110
	5660181	5659276		-	-	PA5029	31	22
5660284	5660357	5661673	5661700	+	-	PA5030	23	167
	5662630	5661701		-	-	PA5031	5	11
	5662742	5663815		+	-	PA5032	3	2
	5663889	5664869		+	-	PA5033	44	18
5666103	5666057	5664990	5664990	-	hemE	PA5034	128	151
5667667	5667667	5666234	5666109	-	gltD	PA5035	64	54
5672272	5672141	5667696	5667668	-	gltB	PA5036	68	49
5674027	5674021	5672366	5672360	-	-	PA5037	209	221
5675183	5675134	5674028	5674028	-	aroB	PA5038	117	114
5675702	5675702	5675184	5675184	-	aroK	PA5039	229	197

5677858	5677858	5675714	5675703	-	pilQ	PA5040	406	244
	5678436	5677912	5677859	-	pilP	PA5041	350	167
	5679056	5678433		-	pilO	PA5042	456	210
	5679649	5679053		-	pilN	PA5043	540	223
5680738	5680713	5679649		-	pilM	PA5044	505	184
5680746	5680898	5683366	5683368	+	ponA	PA5045	97	87
5684763	5684739	5683471	5683457	-	-	PA5046	843	1147
	5686281	5684842	5684764	-	-	PA5047	80	101
5687100	5687045	5686278		-	-	PA5048	133	150
5687350	5687320	5687105	5687104	-	rpmE	PA5049	5933	6474
5687351			5687368	?	-	predicted RN	2364	2747
5687432	5687496	5689715	5689847	+	priA	PA5050	34	32
5689964	5689964	5691727	5691754	+	argS	PA5051	256	234
5691762	5691762	5692457	5692554	+	-	PA5052	111	102
5692574	5692576	5693109	5693109	+	hslV	PA5053	207	240
5693135	5693138	5694481	5694492	+	hslU	PA5054	311	310
5694516	5694576	5694947	5694947	+	-	PA5055	121	112
	5695366	5697045	5697045	+	phaC1	PA5056	29	25
	5697198	5698055		+	phaD	PA5057	23	18
	5698359	5700041		+	phaC2	PA5058	12	8
	5700097	5700714		+	-	PA5059	17	15
5701697	5701687	5700758	5700752	-	phaF	PA5060	371	242
5702114	5702114	5701698	5701698	-	-	PA5061	209	167
5702541	5702533	5702258	5702193	-	-	PA5062	158	88
5702669	5702669	5703439	5703439	+	ubiE	PA5063	269	221
5703440	5703454	5704080		+	-	PA5064	116	90
	5704077	5705678	5705793	+	ubiB	PA5065	71	70
5705794	5705794	5706198		+	hisI	PA5066	251	187
	5706191	5706526	5706551	+	hisE	PA5067	253	251
5706552	5706552	5706800	5706813	+	tatA	PA5068	365	336
5706814	5706814	5707239		+	tatB	PA5069	200	221
	5707236	5708039		+	tatC	PA5070	121	159

	5708036	5708743	5708954	+	-	PA5071	40	48
5708955	5708955	5710898	5711007	+	-	PA5072	46	44
5711008	5711008	5711469	5711473	+	-	PA5073	90	83
	5712211	5711477	5711474	-	-	PA5074	143	129
5713229	5713166	5712204		-	-	PA5075	138	139
5714169	5714032	5713232	5713232	-	-	PA5076	244	268
	5716855	5714270	5714270	-	mdoH	PA5077	186	170
5718425	5718425	5716848		-	-	PA5078	385	335
	5719319	5718882	5718426	-	-	PA5079	93	91
5720332	5720287	5719316		-	-	PA5080	147	156
	5720469	5720945		+	-	PA5081	17	15
	5721849	5720950		-	-	PA5082	20	22
	5722242	5721892		-	-	PA5083	11	10
	5723517	5722267		-	-	PA5084	8	4
	5723582	5724538		+	-	PA5085	39	24
	5725242	5724601	5724601	-	-	PA5086	33	29
5726348	5726348	5725479	5725479	-	-	PA5087	28	25
	5727238	5726357		-	-	PA5088	23	19
	5729476	5727239		-	-	PA5089	9	8
	5731845	5729473		-	-	PA5090	8	8
	5732916	5732116		-	hutG	PA5091	16	12
	5734117	5732909		-	hutI	PA5092	14	11
	5735646	5734114		-	-	PA5093	7	7
	5736473	5735643		-	-	PA5094	11	7
	5737321	5736470		-	-	PA5095	11	7
	5738314	5737346		-	-	PA5096	7	4
	5739835	5738432		-	-	PA5097	4	4
	5741427	5739898		-	hutH	PA5098	4	2
	5742966	5741524		-	-	PA5099	3	1
	5744753	5743074		-	hutU	PA5100	9	4
	5745876	5745079		-	-	PA5101	9	3
	5746833	5745895		-	-	PA5102	4	3

	5747871	5746849		-	-	PA5103	10	8
	5748603	5748013		-	-	PA5104	24	25
5749430	5749352	5748600		-	hutC	PA5105	45	35
	5749453	5750814		+	-	PA5106	10	5
	5751530	5750961	5750913	-	blc	PA5107	90	93
5751856	5751784	5751527		-	-	PA5108	158	156
5752463	5752459	5751857	5751857	-	-	PA5109	202	171
5753489	5753474	5752464	5752464	-	fbp	PA5110	541	542
5754246	5754144	5753614	5753586	-	gloA3	PA5111	141	149
5756298	5756238	5754298	5754298	-	estA	PA5112	120	156
	5757741	5756347	5756299	-	-	PA5113	83	83
5761408	5761343	5757738		-	-	PA5114	60	62
	5762066	5761482		-	-	PA5115	10	5
	5762150	5762575		+	-	PA5116	24	8
5764478	5764476	5762659	5762652	-	typA	PA5117	450	333
5766167	5766147	5764693	5764502	-	thiI	PA5118	190	209
5766310	5766484	5767893	5767915	+	glnA	PA5119	691	818
5767975	5768041	5768445		+	-	PA5120	57	69
5770774	5770649	5768442		-	-	PA5121	33	43
5770937	5770937	5771458		+	-	PA5122	117	46
	5771455	5772027		+	-	PA5123	43	21
	5772298	5773374	5773374	+	ntrB	PA5124	23	29
	5773377	5774807		+	ntrC	PA5125	32	30
	5776087	5775620		-	-	PA5126	38	31
	5776086	5776547	5776605	+	-	PA5127	83	111
5777103	5777097	5776606	5776606	-	secB	PA5128	1635	1286
5777388	5777388	5777134	5777104	-	grx	PA5129	383	310
5777816	5777809	5777390	5777389	-	-	PA5130	268	303
5777961	5778134	5779681	5779702	+	pgm	PA5131	268	251
	5779995	5780813		+	-	PA5132	13	19
5780953	5780963	5782249	5782249	+	-	PA5133	117	118
5782250	5782278	5783588		+	-	PA5134	209	189

	5783588	5784361	5784429	+	-	PA5135	60	62
5784430	5784430	5785911	5785911	+	-	PA5136	91	92
5786852	5786703	5785948	5785914	-	-	PA5137	72	59
5787610	5787605	5786853	5786853	-	-	PA5138	215	135
5788504	5788442	5787696	5787613	-	-	PA5139	234	89
5789384	5789384	5788614	5788614	-	hisF1	PA5140	194	176
5790295	5790132	5789395	5789385	-	hisA	PA5141	231	229
	5791086	5790445	5790376	-	hisH1	PA5142	286	263
5791690	5791676	5791083		-	hisB	PA5143	386	367
	5791837	5792235		+	-	PA5144	8	12
	5792505	5793611		+	-	PA5145	22	17
5793692	5793705	5795957		+	-	PA5146	130	130
	5795954	5797021	5797034	+	mutY	PA5147	128	131
5797048	5797065	5797337	5797364	+	-	PA5148	294	260
5797365	5797365	5798447	5798554	+	-	PA5149	69	58
5798560			5798635	+	-	PA5149.1	20	35
5799479	5799430	5798693	5798636	-	-	PA5150	43	36
	5799589	5800278		+	-	PA5151	22	19
5800496	5800527	5801300	5801314	+	-	PA5152	249	234
5801315	5801315	5802067	5802067	+	-	PA5153	207	378
5802105	5802129	5802824		+	-	PA5154	43	101
	5802821	5803513	5803567	+	-	PA5155	15	35
5803574	5803867	5804802	5804806	+	-	PA5156	47	43
5804807	5805206	5805676	5805679	+	-	PA5157	31	36
	5805680	5807158		+	-	PA5158	18	20
	5807173	5808357		+	-	PA5159	23	27
	5808368	5809897		+	-	PA5160	10	17
5809970			5810045	+	-	PA5160.1	8	33
5810148	5810281	5811339		+	rmlB	PA5161	306	257
	5811336	5812244		+	rmlD	PA5162	262	205
	5812241	5813122		+	rmlA	PA5163	410	331
	5813122	5813667	5813685	+	rmlC	PA5164	340	289

5813698	5813727	5815565		+	-	PA5165	44	34
	5815562	5816950	5816978	+	-	PA5166	56	55
5817105	5817191	5818186	5818186	+	-	PA5167	45	189
5818187	5818202	5818834		+	-	PA5168	19	75
	5818831	5820114	5820564	+	-	PA5169	10	78
5820904	5820910	5822358	5822358	+	arcD	PA5170	986	902
5822359	5822380	5823636	5823715	+	arcA	PA5171	239	1520
5823716	5823716	5824726	5824726	+	arcB	PA5172	203	2193
5824727	5824787	5825719	5825726	+	arcC	PA5173	109	1333
5826030	5826135	5828039	5828039	+	-	PA5174	269	393
	5828907	5828086	5828066	-	cysQ	PA5175	93	84
5829470	5829470	5828904		-	-	PA5176	199	173
5829527	5829596	5830261	5830307	+	-	PA5177	64	55
5830800	5830750	5830313	5830313	-	-	PA5178	314	251
	5830924	5831805		+	-	PA5179	27	21
	5831902	5832741		+	-	PA5180	14	9
	5832749	5835070		+	-	PA5181	14	8
5835480			5835082	-	-	PA5181.1	38	27
5835481	5835482	5835895	5835903	+	-	PA5182	83	71
5835982	5835994	5836401	5836419	+	-	PA5183	57	45
5836465	5836910	5837467		+	-	PA5184	71	52
	5837892	5837449		-	-	PA5185	40	20
	5839078	5837915		-	-	PA5186	12	8
	5840895	5839105		-	-	PA5187	11	6
	5842132	5840897		-	-	PA5188	12	9
5842188	5842275	5843183	5843197	+	-	PA5189	47	41
5843909	5843800	5843198	5843198	-	-	PA5190	91	80
	5844054	5844410		+	-	PA5191	43	35
5846009	5846009	5844468	5844456	-	pckA	PA5192	877	740
5846010			5846019	?	-	predicted RN	483	502
5847103	5847018	5846125	5846021	-	yrfI	PA5193	195	172
5847121	5847131	5847934	5847934	+	-	PA5194	188	185

5848486	5848367	5847972	5847935	-	-	PA5195	54	44
5848501	5848577	5849011	5849142	+	-	PA5196	43	39
	5849143	5850048		+	rimK	PA5197	24	19
5850049	5850239	5851162	5851162	+	-	PA5198	74	58
5852652	5852558	5851239	5851174	-	amgS	PA5199	50	49
5853398	5853396	5852653	5852653	-	amgR	PA5200	207	173
5853563	5853585	5855924	5855935	+	-	PA5201	265	239
5855936	5855936	5856325	5856531	+	-	PA5202	130	126
5856532	5856532	5858115	5858116	+	gshA	PA5203	120	114
5859580	5859456	5858158	5858158	-	argA	PA5204	134	56
	5859793	5860494		+	-	PA5205	30	26
5861632	5861621	5860467		-	argE	PA5206	49	32
	5862989	5861721	5861721	-	-	PA5207	19	35
5863714	5863711	5863034	5862990	-	-	PA5208	228	175
5863715	5863825	5865189	5865226	+	-	PA5209	72	53
5865227	5865227	5867011	5867190	+	-	PA5210	102	60
	5867233	5867685		+	-	PA5211	20	21
5867748	5867817	5868146	5868180	+	-	PA5212	82	76
	5871057	5868181		-	gcvP1	PA5213	15	17
5871648	5871620	5871231	5871189	-	gcvH1	PA5214	669	656
5872775	5872749	5871667	5871660	-	gcvT1	PA5215	259	311
5874586	5874519	5872900	5872782	-	-	PA5216	39	38
5875595	5875585	5874587	5874587	-	-	PA5217	148	147
	5876567	5875653		-	-	PA5218	19	14
	5876664	5877857		+	-	PA5219	17	14
	5877896	5878717		+	-	PA5220	35	29
5879999	5879971	5878754	5878718	-	-	PA5221	83	80
5880486	5880482	5880000	5880000	-	-	PA5222	95	104
5881671	5881671	5880487	5880487	-	ubiH	PA5223	164	157
5883020	5883013	5881679	5881672	-	pepP	PA5224	299	248
5883595	5883577	5883023	5883021	-	-	PA5225	192	140
5883664	5883664	5883975		+	-	PA5226	250	151

	5883972	5884286	5884319	+	-	PA5227	217	140
5884320			5884502	+	ssrS	PA5227.1	4685	6456
5884503	5884508	5885119	5885376	+	-	PA5228	97	79
5885471	5885486	5885944	5885953	+	-	PA5229	70	105
5887078	5887078	5885954	5885954	-	-	PA5230	48	93
	5889832	5887082	5887079	-	-	PA5231	70	118
5890951	5890902	5889829		-	-	PA5232	294	257
5891593	5891528	5891109	5890961	-	-	PA5233	93	65
	5891683	5892660		+	-	PA5234	32	20
5892907	5892910	5894256	5894256	+	glpT	PA5235	229	77
	5895261	5894293	5894257	-	-	PA5236	131	92
5896729	5896727	5895261		-	-	PA5237	171	138
	5898794	5896806		-	-	PA5238	11	9
5900253	5900124	5898865	5898855	-	rho	PA5239	699	539
5900727	5900695	5900369	5900348	-	trxA	PA5240	2520	1925
5900858	5900879	5902399		+	ppx	PA5241	222	173
5904596	5904596	5902386		-	ppk	PA5242	278	251
5905655	5905627	5904614	5904597	-	hemB	PA5243	440	350
5905866	5905871	5906461	5906500	+	-	PA5244	102	103
5906623	5906626	5907294	5907294	+	-	PA5245	243	176
5907331	5907390	5907863		+	-	PA5246	64	57
5908396	5908330	5907848		-	-	PA5247	81	73
5910302	5910298	5908397	5908397	-	-	PA5248	65	80
5911203	5911032	5910403	5910306	-	-	PA5249	45	28
5912020	5912020	5911262	5911206	-	-	PA5250	154	170
	5912605	5912027	5912025	-	-	PA5251	82	87
5914522	5914521	5912605		-	-	PA5252	125	131
5914985			5915042	?	-	predicted RN	328	222
5916106	5916101	5915043	5915043	-	algP	PA5253	956	619
	5916227	5916856		+	-	PA5254	38	32
5917497	5917400	5916918	5916857	-	algQ	PA5255	701	452
5918289	5918171	5917680	5917504	-	dsbH	PA5256	86	96

	5919588	5918350	5918350	-	-	PA5257	148	141
5920715	5920715	5919585		-	-	PA5258	142	137
	5921497	5920742	5920716	-	hemD	PA5259	76	73
5922438	5922435	5921494		-	hemC	PA5260	198	150
5923294	5923290	5922544	5922441	-	algR	PA5261	145	113
5924404	5924371	5923295	5923295	-	algZ	PA5262	78	41
5924562	5924596	5925990	5926133	+	argH	PA5263	204	158
5927335	5927105	5926134	5926134	-	-	PA5264	65	52
	5930605	5927336		-	-	PA5265	22	12
	5932677	5930602		-	-	PA5266	6	5
	5933395	5932877		-	hcpB	PA5267	10	9
5934700	5934668	5933688	5933396	-	corA	PA5268	60	41
5934735	5934770	5935045	5935045	+	-	PA5269	115	75
5935046	5935064	5935945	5935959	+	-	PA5270	73	61
5935988	5935988	5936221	5936222	+	-	PA5271	105	76
	5936370	5939222		+	cyaA	PA5272	29	30
5939263	5939263	5939976		+	-	PA5273	37	24
5940455	5940424	5940020	5940020	-	rnk	PA5274	466	488
5941133	5941082	5940747	5940474	-	-	PA5275	88	59
5941328	5941336	5941476	5941486	+	lppL	PA5276	336	336
5941487	5941487	5942734	5942744	+	lysA	PA5277	281	269
5942745	5942745	5943575	5943595	+	dapF	PA5278	275	266
5943606	5943606	5944307	5944307	+	-	PA5279	269	228
5944308	5944327	5945238		+	sss	PA5280	76	65
	5945235	5945933	5945962	+	-	PA5281	34	33
	5947132	5945963		-	-	PA5282	6	3
	5947295	5948671		+	-	PA5283	39	26
	5949610	5948696		-	-	PA5284	12	13
5950365	5950351	5950034	5950024	-	-	PA5285	1724	1789
5950366			5950395	?	-	predicted RN	1202	1518
5950866	5950854	5950429	5950396	-	-	PA5286	369	586
5952446	5952443	5951115	5951045	-	amtB	PA5287	63	48

5952854	5952821	5952483	5952458	-	glnK	PA5288	758	395
5953250	5953261	5953521	5953561	+	-	PA5289	136	135
	5953562	5955055		+	-	PA5290	28	21
5955056	5955180	5957165	5957183	+	-	PA5291	112	57
	5958257	5957208		-	pchP	PA5292	29	15
	5959325	5958408		-	-	PA5293	18	10
	5959474	5960940		+	-	PA5294	15	10
5962567	5962528	5960852		-	-	PA5295	116	91
5962701	5962716	5964725	5964725	+	rep	PA5296	158	186
	5964859	5966577		+	poxB	PA5297	9	9
5966688	5966706	5967278	5967285	+	-	PA5298	453	622
5969354	5969145	5967286	5967286	-	-	PA5299	66	76
5969827	5969765	5969355	5969355	-	cycB	PA5300	1040	1219
5970601	5970535	5969987	5969963	-	-	PA5301	416	355
5971849	5971759	5970686	5970686	-	dadX	PA5302	190	483
	5972203	5971850	5971850	-	-	PA5303	644	1618
5973516	5973476	5972178		-	dadA	PA5304	579	1153
5974177	5974177	5973833	5973809	-	-	PA5305	177	111
5974401	5974381	5974190	5974178	-	-	PA5306	225	170
	5976969	5974402	5974402	-	-	PA5307	38	26
5977115	5977123	5977611	5977611	+	lrp	PA5308	164	94
5977618	5977789	5979108	5979193	+	-	PA5309	58	35
	5979194	5980783		+	-	PA5310	25	29
	5981950	5980787		-	-	PA5311	24	10
5983617	5983590	5982097	5982062	-	-	PA5312	255	91
5983796	5983820	5985154	5985177	+	-	PA5313	59	26
5985219	5985219	5985581	5985634	+	-	PA5314	93	35
5985871	5985871	5985716	5985688	-	rpmG	PA5315	6516	5334
	5986119	5985883	5985872	-	rpmB	PA5316	8525	7570
5986120			5986170	+	-	PA5316.1	6315	7126
5986171	5986475	5988055	5988080	+	-	PA5317	59	33

5988081	5988081	5988629		+	-	PA5318	37	21
5989354	5989320	5988646	5988633	-	radC	PA5319	54	40
5989420	5989460	5990668	5990668	+	coaC	PA5320	165	151
5990669	5990676	5991131	5991952	+	dut	PA5321	249	228
5992383	5992383	5993774	5993774	+	algC	PA5322	356	304
5993775	5993791	5994696	5994696	+	argB	PA5323	349	327
5995861	5995811	5994741	5994697	-	-	PA5324	62	73
	5996036	5996986		+	-	PA5325	5	5
	5998265	5997057		-	-	PA5326	4	3
	5999676	5998348		-	-	PA5327	4	3
	6000143	5999754		-	-	PA5328	2	2
6000287	6000317	6000781		+	-	PA5329	71	54
6001378	6001378	6000761		-	-	PA5330	261	374
6002068	6002040	6001399	6001379	-	pyrE	PA5331	407	868
6002114	6002121	6002900	6002900	+	crc	PA5332	652	0
6003333	6003330	6002959	6002949	-	-	PA5333	276	260
6004102	6004096	6003377	6003377	-	rph	PA5334	340	281
6004235	6004277	6005140	6005140	+	-	PA5335	186	181
6005198	6005199	6005810	6005867	+	gmk	PA5336	187	175
6005870	6005899	6006165	6006181	+	rpoZ	PA5337	1441	1272
6006182			6006230	?	-	predicted RN	968	884
6006231	6006231	6008336	6008395	+	spoT	PA5338	346	344
6008397	6008398	6008778	6008780	+	-	PA5339	1283	1064
6008783	6008833	6009564	6009564	+	-	PA5340	329	339
6010191	6010191	6009571	6009565	-	-	PA5341	40	43
	6011058	6010258	6010192	-	-	PA5342	42	31
6011918	6011909	6011058		-	-	PA5343	87	58
6012047	6012047	6012979		+	oxyR	PA5344	256	180
	6012976	6015051	6015134	+	recG	PA5345	108	85
6015141	6015141	6016550	6016621	+	-	PA5346	213	125
6017020	6017014	6016622	6016622	-	-	PA5347	188	221
6017502	6017422	6017150	6017150	-	-	PA5348	659	1583

6018778	6018778	6017624	6017509	-	-	PA5349	104	70
6019080	6018997	6018830	6018779	-	rubA2	PA5350	141	105
6019366	6019348	6019181	6019181	-	rubA1	PA5351	534	455
	6019883	6019482		-	-	PA5352	1	1
	6021114	6019888		-	glcF	PA5353	2	1
	6022203	6021124		-	glcE	PA5354	4	3
	6023702	6022203		-	glcD	PA5355	7	6
	6023907	6024662	6024662	+	glcC	PA5356	36	13
6024726	6024742	6025278	6025304	+	-	PA5357	52	46
6025305	6025305	6026195	6026195	+	ubiA	PA5358	74	78
6026764	6026674	6026219	6026196	-	-	PA5359	63	48
6026779	6026779	6027468	6027541	+	phoB	PA5360	74	80
	6027542	6028873		+	phoR	PA5361	22	18
6028965	6028976	6030316	6030321	+	-	PA5362	115	91
6031365	6031250	6030351	6030322	-	-	PA5363	92	52
6032270	6032268	6031366	6031366	-	-	PA5364	167	112
6033115	6033115	6032387	6032387	-	phoU	PA5365	85	55
6034059	6034044	6033211	6033139	-	pstB	PA5366	90	67
6035736	6035736	6034060	6034060	-	pstA	PA5367	41	40
6038135	6037789	6035756	6035737	-	pstC	PA5368	42	42
6039226	6039183	6038212	6038169	-	pstS	PA5369	97	83
6039703			6039584	-	-	PA5369.1	1	6
6042736			6039846	-	-	PA5369.2	61	56
6043038			6042963	-	-	PA5369.3	1205	1265
6043143			6043067	-	-	PA5369.4	1639	1884
6044743			6043208	-	-	PA5369.5	55	52
6046701	6046626	6045310	6044744	-	-	PA5370	49	47
6046872	6046914	6047318	6047323	+	-	PA5371	190	166
	6049049	6047364		-	betA	PA5372	25	16
6050657	6050657	6049185	6049050	-	betB	PA5373	67	35
6051469	6051311	6050718	6050700	-	betI	PA5374	67	46
	6051644	6053194		+	betT1	PA5375	8	5

6054593	6054590	6053412	6053195	-	-	PA5376	36	28
6055433	6055433	6054594	6054594	-	-	PA5377	34	24
	6056413	6055475		-	-	PA5378	47	28
	6056877	6058253		+	sdaB	PA5379	4	2
6058355	6058673	6059776	6059778	+	gbdR	PA5380	48	51
6060339	6060200	6059823	6059779	-	-	PA5381	29	33
	6061233	6060340		-	-	PA5382	27	19
	6061341	6062408		+	-	PA5383	1	2
	6063347	6062352		-	-	PA5384	1	0
	6063832	6063353		-	cdhB	PA5385	1	1
	6064848	6063883		-	cdhA	PA5386	2	2
	6065783	6064899		-	cdhC	PA5387	2	2
	6066794	6065856		-	-	PA5388	5	1
	6067005	6068015		+	cdhR	PA5389	11	7
	6069099	6067945		-	-	PA5390	10	9
	6069830	6069156		-	-	PA5391	4	4
	6070266	6069844		-	-	PA5392	8	6
	6071618	6070266		-	-	PA5393	4	4
6073384	6073384	6071912		-	cls	PA5394	20	15
	6073538	6074008		+	-	PA5395	3	4
	6074230	6075207		+	-	PA5396	27	12
	6075334	6075864		+	-	PA5397	10	4
	6075880	6077940		+	dgcA	PA5398	6	4
	6078044	6080005		+	dgcB	PA5399	5	2
	6080370	6081356		+	-	PA5400	4	1
	6081392	6082165		+	-	PA5401	5	3
	6082290	6082859	6082859	+	-	PA5402	68	28
6082860	6082867	6083073	6083079	+	-	PA5403	102	70
	6083105	6083509	6083509	+	-	PA5404	31	10
	6083523	6083756		+	-	PA5405	30	9
	6083753	6084085		+	-	PA5406	134	117
	6084082	6084372		+	-	PA5407	97	71

6084549	6084545	6084369		-	-	PA5408	76	41
	6085110	6084550		-	-	PA5409	46	18
	6086674	6085385		-	gbcA	PA5410	11	7
	6087099	6088199		+	gbcB	PA5411	21	14
6090819	6090706	6088193		-	-	PA5412	64	49
6093100	6093086	6092046	6092040	-	ltaA	PA5413	196	173
6093808	6093808	6093167	6093136	-	-	PA5414	144	117
6093984	6094024	6095277	6095328	+	glyA1	PA5415	12	54
	6095363	6096613		+	soxB	PA5416	4	3
	6096707	6097027		+	soxD	PA5417	2	2
	6097024	6100041		+	soxA	PA5418	4	3
	6100135	6100770		+	soxG	PA5419	5	3
	6100820	6101677		+	purU2	PA5420	7	4
	6101884	6103083		+	fdhA	PA5421	8	7
6103121	6103167	6104123	6104123	+	-	PA5422	69	52
6104798	6104737	6104189	6104169	-	-	PA5423	61	62
6105044	6105044	6104799	6104799	-	-	PA5424	201	167
6106241	6106241	6105159	6105074	-	purK	PA5425	232	225
6106858	6106763	6106272	6106242	-	purE	PA5426	379	428
6107028	6107111	6108139	6108175	+	adhA	PA5427	132	130
6109246	6109084	6108176	6108176	-	-	PA5428	66	60
6109250	6109260	6110684	6110754	+	aspA	PA5429	1233	1090
6112273	6112262	6111048	6110760	-	-	PA5430	61	65
	6113774	6112299		-	-	PA5431	11	7
	6113902	6114351		+	-	PA5432	9	12
6114352	6114360	6115034	6115052	+	-	PA5433	29	35
6116492	6116312	6115059	6115053	-	mtr	PA5434	40	54
6118325	6118316	6116493	6116493	-	-	PA5435	161	521
6119822	6119747	6118332	6118332	-	-	PA5436	222	472
6119927	6119961	6120896	6121109	+	-	PA5437	61	53
6122055	6122053	6121172	6121171	-	-	PA5438	213	167
	6122139	6123605		+	-	PA5439	35	33

6123674	6123686	6125080	6125088	+	-	PA5440	168	286
6128004	6127997	6125796	6125786	-	-	PA5441	182	171
6130946	6130911	6128056	6128007	-	-	PA5442	14	45
6131088	6131088	6133274	6133311	+	uvrD	PA5443	191	147
6133312	6133312	6133740	6133740	+	-	PA5444	62	44
6133741	6133838	6135331	6135491	+	-	PA5445	526	27
6135671			6135682	?	-	predicted RN	451	2205
6135683	6135709	6135912	6135926	+	-	PA5446	1082	5457
6135927			6135942	?	-	predicted RN	531	2502
	6137113	6135968	6135943	-	wbpZ	PA5447	73	196
	6138241	6137114		-	wbpY	PA5448	35	41
	6139607	6138225		-	wbpX	PA5449	26	43
	6140869	6139604		-	wzt	PA5450	43	63
	6141666	6140869		-	wzm	PA5451	46	74
6143108	6143105	6141666		-	wbpW	PA5452	50	60
	6144080	6143109	6143109	-	gmd	PA5453	70	104
6145078	6144991	6144077		-	rmd	PA5454	75	69
6145258	6145399	6147027		+	-	PA5455	91	113
	6147021	6148322		+	-	PA5456	71	95
	6148319	6149182		+	-	PA5457	43	60
	6149179	6150321		+	-	PA5458	26	37
	6150315	6151151	6151151	+	-	PA5459	40	63
6151152	6151314	6151526	6151585	+	-	PA5460	27	20
6151587	6151622	6151939	6151939	+	-	PA5461	687	1100
6152050	6152064	6152357	6152357	+	-	PA5462	197	201
6152380	6152386	6152721		+	-	PA5463	136	120
	6152718	6154676	6154783	+	-	PA5464	63	53
6155270	6155203	6154784	6154784	-	-	PA5465	53	36
	6155271	6156215		+	-	PA5466	17	7
	6156212	6156574		+	-	PA5467	29	15
	6156854	6158158		+	-	PA5468	19	7
	6158179	6158943		+	-	PA5469	34	21

	6159563	6158949		-	-	PA5470	54	35
6160752	6160699	6159560		-	-	PA5471	65	57
6160955	6160953	6160912	6160835	-	-	PA5471.1	294	258
6161883	6161867	6161067	6161053	-	-	PA5472	208	146
	6162221	6163969		+	-	PA5473	19	11
6164030	6164038	6165411	6165416	+	-	PA5474	79	37
6166008	6165977	6165417	6165417	-	-	PA5475	222	76
	6167409	6166120		-	citA	PA5476	13	7
	6167783	6168823		+	-	PA5477	41	37
6170147	6170071	6168842	6168841	-	-	PA5478	80	77
6171510	6171510	6170176	6170176	-	gltP	PA5479	443	345
6171676	6171733	6171927	6172222	+	-	PA5480	164	123
6172690	6172690	6172223		-	-	PA5481	20	5
	6172873	6172712		-	-	PA5482	38	10
6173336	6173348	6174697		+	algB	PA5483	64	47
	6174694	6176481	6176515	+	-	PA5484	61	43
6177324	6177295	6176516	6176516	-	ampDh2	PA5485	52	47
6178035	6177918	6177325	6177325	-	-	PA5486	48	42
	6180051	6178036	6178036	-	-	PA5487	88	97
6180926	6180926	6180048		-	-	PA5488	227	223
6181671	6181577	6180942	6180927	-	dsbA	PA5489	712	570
6182360	6182350	6181745	6181696	-	cc4	PA5490	867	959
6182770	6182689	6182396	6182368	-	-	PA5491	336	541
6182864	6182873	6183520	6183734	+	-	PA5492	235	191
6186565	6186525	6183784	6183751	-	polA	PA5493	132	96
6186590	6186603	6186893	6186926	+	-	PA5494	498	332
6186927	6186927	6187877	6187885	+	thrB	PA5495	194	150
6188855	6188855	6188166		-	nrdJb	PA5496	32	61
6191123	6191076	6188872	6188856	-	nrdJa	PA5497	114	93
6192123	6192109	6191186	6191124	-	-	PA5498	46	48
6192179	6192179	6192682		+	np20	PA5499	126	89
	6192682	6193491		+	znuC	PA5500	71	66

	6193484	6194272	6194298	+	znuB	PA5501	48	48
6194307	6194314	6195102	6195256	+	-	PA5502	65	79
6195290	6195309	6196316		+	-	PA5503	171	187
	6196316	6196993	6197009	+	-	PA5504	204	188
6197057	6197070	6197852	6197868	+	-	PA5505	479	388
6198055	6198055	6198912	6198916	+	-	PA5506	187	6
6198917	6198917	6199570		+	-	PA5507	235	9
	6199567	6200898	6200954	+	-	PA5508	130	8
6200955	6200966	6201634	6201735	+	-	PA5509	86	8
6201736	6201736	6203085	6203103	+	-	PA5510	65	10
	6204447	6203104	6203104	-	mifR	PA5511	65	53
	6206210	6204444		-	mifS	PA5512	23	19
6206286	6206292	6207170	6207240	+	poxA	PA5513	29	36
	6207241	6208029		+	-	PA5514	24	16
6208574	6208542	6208042	6208030	-	-	PA5515	62	36
6208575	6208575	6209441	6209441	+	pdxY	PA5516	80	69
6210063	6210063	6209479	6209442	-	-	PA5517	56	58
6212109	6211768	6210065	6210064	-	-	PA5518	37	37
6212722	6212713	6212147	6212128	-	-	PA5519	72	92
	6212790	6213533		+	-	PA5520	45	29
6214370	6214339	6213581	6213547	-	-	PA5521	96	67
6215801	6215801	6214434	6214433	-	-	PA5522	32	24
6217204	6217155	6215803	6215802	-	-	PA5523	47	32
6217252	6217313	6218095	6218095	+	-	PA5524	44	31
6218096	6218100	6218840	6218840	+	-	PA5525	60	60
6219186	6219069	6218857	6218841	-	-	PA5526	156	125
6219700	6219688	6219287	6219287	-	-	PA5527	183	192
6220749	6220739	6219885	6219707	-	-	PA5528	419	324
6221033	6221101	6222858	6222993	+	-	PA5529	55	35
6223107	6223174	6224481	6224638	+	-	PA5530	41	1693
6225956	6225925	6224897	6224885	-	tonB1	PA5531	101	135
	6226979	6226221		-	-	PA5532	19	12

6226980	6227082	6227450	6227543	+	-	PA5533	102	83
	6228244	6227603		-	-	PA5534	12	6
	6229443	6228241		-	-	PA5535	11	6
	6229862	6229458		-	-	PA5536	6	1
	6229975	6230391		+	-	PA5537	31	13
	6231562	6230369		-	amiA	PA5538	5	4
	6231662	6232558		+	-	PA5539	1	2
	6232555	6233115		+	-	PA5540	4	4
	6233118	6234455		+	pyrQ	PA5541	10	7
	6235744	6234500		-	-	PA5542	8	10
	6236229	6235831		-	-	PA5543	11	13
	6238250	6236226		-	-	PA5544	11	12
6239295	6239284	6238325	6238251	-	-	PA5545	64	56
6240869	6240632	6239448	6239337	-	-	PA5546	61	49
6240870	6240870	6241493	6241692	+	-	PA5547	68	38
6241800	6241851	6243056	6243068	+	-	PA5548	53	51
6244961	6244946	6243111	6243098	-	glmS	PA5549	161	171
6245802	6245735	6244962	6244962	-	glmR	PA5550	62	93
6246312	6246312	6245803	6245803	-	-	PA5551	103	108
6247810	6247690	6246326	6246313	-	glmU	PA5552	203	199
6248236	6248236	6247811	6247811	-	atpC	PA5553	1949	1767
6249654	6249654	6248278	6248237	-	atpD	PA5554	4546	3890
6250595	6250545	6249685	6249655	-	atpG	PA5555	4416	3955
6252146	6252140	6250596	6250596	-	atpA	PA5556	4404	3834
6252695	6252695	6252159	6252154	-	atpH	PA5557	5392	4988
6253177	6253177	6252707	6252696	-	atpF	PA5558	5970	5366
6253510	6253492	6253235	6253178	-	atpE	PA5559	9297	8397
6254411	6254411	6253542	6253512	-	atpB	PA5560	1268	1402
6254868	6254808	6254428	6254412	-	atpI	PA5561	250	420
6255844	6255844	6254972	6254874	-	spoOJ	PA5562	308	245
6256650	6256642	6255854	6255845	-	soj	PA5563	282	214
	6257305	6256661	6256661	-	gidB	PA5564	330	294

6259211	6259197	6257305		-	gidA	PA5565	312	284
	6260054	6259671		-	-	PA5566	11	5
6261827	6261757	6260390	6260270	-	-	PA5567	70	66
6263804	6263564	6261828	6261828	-	-	PA5568	607	596
6264212	6264212	6263805	6263805	-	rnpA	PA5569	2727	2797
6264400	6264361	6264227	6264213	-	rpmH	PA5570	6241	5834

Protein AC	Mass	pI	Prot score	Num pept	Coverage	Ratio Acrc/PAO1 R1	Log2_Ratio	ZSCORE	pvalue	rank	FDR	Ratio Acrc/PAO1 R2	Log2_Ratio	ZSCORE	pvalue	rank	FDR	Ratio Acrc/PAO1 R3	Log2_Ratio	ZSCORE	pvalue	rank	FDR	MEDIA Acrc/PAO1	Number of replicates	Log2_Ratio	SD
**PA0001**	61669	8,61	777,40	21	34,94	1,11	0,16	0,47	0,64	1417	**0,98**	1,16	0,21	0,70	0,48	1090	**0,96**	1,11	0,15	0,46	0,64	1322	**1,00**	1,13	3,00	0,17	0,03
**PA0002**	42635	5,13	1570,38	31	57,65	0,93	-0,10	-0,44	0,66	1447	**0,99**	0,97	-0,04	-0,24	0,81	1793	**0,98**	0,92	-0,13	-0,49	0,62	1287	**1,00**	0,94	3,00	-0,09	0,03
**PA0003**	43295	8,22	146,86	3	12,50	1,02	0,03	0,02	0,98	2142	**1,00**	0,77	-0,37	-1,49	0,14	500	**0,59**	0,78	-0,36	-1,28	0,20	567	**0,73**	0,86	3,00	-0,22	0,14
**PA0004**	97522	5,62	2723,06	59	50,24	0,84	-0,25	-0,96	0,34	805	**0,90**	0,82	-0,28	-1,14	0,26	678	**0,82**	0,83	-0,27	-0,97	0,33	780	**0,87**	0,83	3,00	-0,27	0,01
**PA0005**	30654	9,74	382,57	11	43,93	0,89	-0,17	-0,67	0,50	1121	**0,98**	1,00	0,00	-0,08	0,93	2036	**0,99**	0,85	-0,24	-0,87	0,39	869	**0,92**	0,91	3,00	-0,13	0,08
**PA0006**	20295	4,71	193,91	4	25,68	1,04	0,05	0,10	0,92	1989	**1,00**	1,12	0,16	0,51	0,61	1361	**0,97**	1,05	0,07	0,17	0,87	1787	**1,00**	1,07	3,00	0,10	0,05
**PA0008**	78625	5,11	2766,04	52	56,04	0,93	-0,11	-0,47	0,64	1397	**0,99**	0,89	-0,16	-0,69	0,49	1099	**0,96**	0,87	-0,20	-0,74	0,46	1006	**0,94**	0,90	3,00	-0,16	0,03
**PA0009**	37331	4,99	634,60	14	34,19	0,93	-0,11	-0,46	0,64	1418	**0,98**	0,90	-0,16	-0,69	0,49	1101	**0,96**	0,83	-0,27	-0,98	0,33	774	**0,87**	0,88	3,00	-0,18	0,05
**PA0011**	36591	9,42	243,18	6	19,80	0,97	-0,04	-0,21	0,83	1825	**0,99**	1,05	0,07	0,15	0,88	1941	**0,98**	0,93	-0,10	-0,39	0,70	1432	**1,00**	0,99	3,00	-0,02	0,06
**PA0016**	52438	5,23	446,53	13	27,80	1,05	0,07	0,15	0,88	1937	**0,99**	1,11	0,15	0,48	0,63	1420	**0,96**	1,05	0,08	0,20	0,84	1730	**1,00**	1,07	3,00	0,10	0,04
**PA0017**	50133	8,33	414,85	10	26,15	1,06	0,09	0,23	0,82	1793	**0,99**	1,02	0,03	0,00	1,00	2160	**1,00**	1,02	0,02	0,02	0,98	2039	**1,00**	1,03	3,00	0,05	0,03
**PA0018**	34586	6,45	543,02	12	42,55	1,01	0,01	-0,04	0,97	2112	**1,00**	1,15	0,20	0,64	0,52	1176	**0,96**	0,99	-0,01	-0,08	0,93	1921	**1,00**	1,05	3,00	0,07	0,08
**PA0019**	21076	4,97	425,36	9	46,80	1,03	0,04	0,06	0,95	2079	**1,00**	1,07	0,10	0,27	0,79	1745	**0,98**	0,99	-0,02	-0,12	0,91	1859	**1,00**	1,03	3,00	0,04	0,04
**PA0020**	40013	8,92	795,21	17	39,60	0,59	-0,77	-2,78	0,01	171	**0,07**	0,55	-0,86	-3,31	0,00	154	**0,01**	0,51	-0,96	-3,32	0,00	144	**0,01**	0,55	3,00	-0,86	0,04
**PA0023**	36940	5,94	253,54	8	21,38	0,72	-0,47	-1,74	0,08	362	**0,49**	0,80	-0,33	-1,31	0,19	564	**0,72**	0,82	-0,29	-1,03	0,30	733	**0,86**	0,78	3,00	-0,36	0,05
**PA0024**	36325	5,59	426,73	14	36,56	1,06	0,08	0,21	0,83	1823	**0,99**	1,16	0,22	0,71	0,47	1069	**0,96**	1,15	0,20	0,62	0,54	1155	**0,96**	1,12	3,00	0,17	0,06
**PA0025**	30659	5,57	309,25	8	31,56	1,04	0,06	0,13	0,90	1964	**0,99**	0,97	-0,05	-0,28	0,78	1723	**0,98**	0,96	-0,06	-0,26	0,79	1634	**1,00**	0,99	3,00	-0,02	0,05
**PA0035**	29427	5,17	252,99	6	22,95	0,86	-0,22	-0,87	0,38	903	**0,93**	0,92	-0,13	-0,57	0,57	1282	**0,96**	0,86	-0,22	-0,79	0,43	945	**0,94**	0,88	3,00	-0,19	0,03
**PA0036**	46523	5,85	358,06	10	18,30	0,80	-0,32	-1,21	0,23	626	**0,79**	1,00	-0,01	-0,12	0,90	1988	**0,98**	0,78	-0,37	-1,30	0,19	559	**0,72**	0,86	3,00	-0,22	0,12
**PA0040**	64700	8,35	156,38	4	11,90	1,22	0,28	0,91	0,36	858	**0,91**	1,12	0,16	0,50	0,62	1387	**0,96**	1,08	0,10	0,30	0,77	1582	**1,00**	1,14	3,00	0,18	0,07
**PA0045**	26300	9,16	334,66	8	37,56	0,73	-0,45	-1,67	0,09	386	**0,53**	0,97	-0,05	-0,29	0,78	1708	**0,98**	1,39	0,47	1,54	0,12	446	**0,57**	1,03	3,00	0,04	0,33
**PA0047**	18172	5,33	209,33	3	21,30	1,04	0,05	0,10	0,92	1994	**1,00**	0,93	-0,10	-0,47	0,64	1431	**0,96**	0,89	-0,17	-0,64	0,53	1135	**0,95**	0,95	3,00	-0,07	0,08
**PA0055**	19430	5,63	226,04	5	39,42	0,78	-0,36	-1,35	0,18	532	**0,73**	0,89	-0,16	-0,70	0,48	1089	**0,96**	0,94	-0,09	-0,38	0,71	1454	**1,00**	0,87	3,00	-0,20	0,08
**PA0067**	79122	5,13	1489,39	38	38,96	0,95	-0,08	-0,35	0,72	1595	**0,99**	0,88	-0,19	-0,81	0,42	968	**0,94**	0,87	-0,19	-0,72	0,47	1044	**0,93**	0,90	3,00	-0,15	0,04
**PA0070**	34567	8,93	1625,04	27	56,54	0,94	-0,09	-0,41	0,68	1498	**0,99**	1,03	0,04	0,05	0,96	2080	**1,00**	1,08	0,11	0,32	0,75	1548	**1,00**	1,01	3,00	0,02	0,07
**PA0071**	65338	5,81	181,51	6	9,90	0,95	-0,07	-0,34	0,74	1616	**0,99**	0,84	-0,25	-1,04	0,30	756	**0,85**	1,00	0,01	-0,03	0,97	2004	**1,00**	0,93	3,00	-0,10	0,08
**PA0074**	115517	5,33	374,20	11	12,85	0,97	-0,04	-0,23	0,82	1796	**0,99**	0,93	-0,11	-0,51	0,61	1377	**0,96**	0,87	-0,20	-0,74	0,46	1018	**0,93**	0,92	3,00	-0,12	0,05
**PA0075**	26413	5,83	145,14	6	30,17	1,23	0,30	0,98	0,33	794	**0,90**	0,87	-0,21	-0,87	0,38	898	**0,93**	0,71	-0,49	-1,72	0,08	368	**0,47**	0,94	3,00	-0,09	0,27
**PA0076**	24267	4,67	277,45	5	25,12	0,95	-0,07	-0,32	0,75	1646	**0,99**	0,76	-0,40	-1,59	0,11	457	**0,53**	0,84	-0,24	-0,88	0,38	855	**0,91**	0,85	3,00	-0,23	0,10
**PA0077**	126134	6,02	638,29	19	16,18	0,94	-0,08	-0,37	0,71	1578	**0,98**	0,96	-0,05	-0,29	0,77	1696	**0,98**	0,78	-0,36	-1,27	0,20	568	**0,74**	0,90	3,00	-0,16	0,10
**PA0078**	50520	7,81	415,01	10	31,29	0,97	-0,04	-0,23	0,82	1797	**0,99**	0,85	-0,23	-0,96	0,34	821	**0,89**	1,02	0,03	0,04	0,97	1997	**1,00**	0,95	3,00	-0,08	0,09
**PA0079**	49778	5,74	783,67	23	38,39	0,95	-0,07	-0,34	0,74	1624	**0,99**	0,87	-0,21	-0,87	0,38	894	**0,93**	0,73	-0,46	-1,62	0,10	406	**0,53**	0,85	3,00	-0,24	0,11
**PA0080**	17542	6,11	188,11	4	26,60	1,06	0,09	0,22	0,82	1805	**0,99**	0,90	-0,16	-0,69	0,49	1113	**0,96**	1,10	0,14	0,42	0,67	1382	**1,00**	1,02	3,00	0,03	0,11
**PA0081**	54810	5,21	205,59	5	9,00	0,67	-0,57	-2,08	0,04	267	**0,30**	0,81	-0,31	-1,26	0,21	606	**0,75**	0,56	-0,84	-2,91	0,00	179	**0,04**	0,68	3,00	-0,56	0,12
**PA0082**	37248	4,41	411,04	9	32,00	0,78	-0,36	-1,33	0,18	548	**0,73**	0,84	-0,25	-1,02	0,31	782	**0,86**	0,75	-0,42	-1,49	0,14	472	**0,60**	0,79	3,00	-0,34	0,05
**PA0083**	20935	5,13	273,28	8	26,33	0,71	-0,49	-1,79	0,07	343	**0,47**	0,72	-0,48	-1,90	0,06	355	**0,35**	0,73	-0,45	-1,60	0,11	419	**0,54**	0,72	3,00	-0,47	0,01
**PA0084**	60434	5,19	1154,96	21	50,80	0,77	-0,38	-1,41	0,16	507	**0,68**	0,69	-0,54	-2,12	0,03	299	**0,25**	0,73	-0,45	-1,58	0,11	424	**0,55**	0,73	3,00	-0,46	0,04
**PA0085**	19611	6,29	902,89	18	51,29	0,79	-0,35	-1,30	0,19	564	**0,74**	0,65	-0,63	-2,44	0,01	248	**0,13**	0,71	-0,50	-1,74	0,08	365	**0,46**	0,71	3,00	-0,49	0,07
**PA0086**	31109	4,67	179,60	3	13,77	0,91	-0,14	-0,58	0,56	1239	**0,98**	0,82	-0,29	-1,16	0,25	655	**0,81**	0,79	-0,34	-1,22	0,22	602	**0,77**	0,84	3,00	-0,25	0,06
**PA0088**	71296	6,96	224,54	8	12,45	0,93	-0,11	-0,46	0,65	1428	**0,98**	0,92	-0,13	-0,57	0,57	1264	**0,97**	0,76	-0,40	-1,40	0,16	522	**0,64**	0,87	3,00	-0,20	0,09
**PA0090**	104625	5,42	1512,40	39	42,52	0,95	-0,08	-0,35	0,73	1601	**0,99**	0,90	-0,15	-0,65	0,52	1156	**0,96**	0,87	-0,21	-0,76	0,45	984	**0,94**	0,91	3,00	-0,14	0,04
**PA0094**	17310	6,31	117,10	3	21,53	0,93	-0,11	-0,47	0,64	1398	**0,99**	0,91	-0,13	-0,59	0,56	1232	**0,97**	0,87	-0,19	-0,71	0,48	1047	**0,94**	0,90	3,00	-0,15	0,03
**PA0100**	34980	4,30	96,30	2	6,20	0,70	-0,51	-1,88	0,06	303	**0,43**	0,85	-0,23	-0,94	0,35	837	**0,90**	0,80	-0,32	-1,15	0,25	636	**0,81**	0,78	3,00	-0,35	0,08
**PA0102**	27844	6,19	383,52	9	32,37	0,77	-0,38	-1,42	0,16	500	**0,67**	0,69	-0,53	-2,09	0,04	308	**0,26**	0,67	-0,58	-2,04	0,04	295	**0,29**	0,71	3,00	-0,50	0,05

**PA0125**	8852	5,48	297,51	6	48,00	1,09	0,12	0,35	0,73	1603	**0,99**	1,08	0,10	0,29	0,77	1702	**0,98**	0,92	-0,13	-0,48	0,63	1295	**1,00**	1,03	3,00	0,04	0,10
**PA0126**	24509	7,66	624,48	11	39,41	0,93	-0,11	-0,45	0,65	1432	**0,99**	0,90	-0,16	-0,69	0,49	1110	**0,96**	0,95	-0,08	-0,32	0,75	1553	**1,00**	0,92	3,00	-0,11	0,03
**PA0128**	14227	5,07	264,76	7	33,39	0,78	-0,36	-1,34	0,18	542	**0,73**	0,79	-0,35	-1,39	0,16	534	**0,67**	0,93	-0,10	-0,41	0,69	1404	**1,01**	0,83	3,00	-0,26	0,09
**PA0130**	57161	5,70	942,28	20	37,82	1,77	0,83	2,82	0,00	168	**0,06**	2,27	1,19	4,32	0,00	107	**0,00**	2,07	1,05	3,50	0,00	134	**0,01**	2,04	3,00	1,03	0,25
**PA0132**	50887	6,06	684,55	15	31,89	2,19	1,13	3,90	0,00	97	**0,00**	2,66	1,41	5,16	0,00	81	**0,00**	3,12	1,64	5,51	0,00	73	**0,00**	2,66	3,00	1,41	0,46
**PA0139**	22782	5,89	1266,39	26	73,48	1,14	0,19	0,58	0,56	1246	**0,98**	1,16	0,21	0,69	0,49	1102	**0,96**	1,19	0,25	0,78	0,44	966	**0,93**	1,16	3,00	0,22	0,02
**PA0140**	60498	5,26	913,07	21	36,10	1,22	0,29	0,93	0,35	835	**0,91**	1,19	0,25	0,85	0,40	918	**0,93**	1,20	0,27	0,84	0,40	893	**0,92**	1,21	3,00	0,27	0,01
**PA0141**	37239	8,41	210,05	6	24,93	0,88	-0,18	-0,72	0,47	1063	**0,97**	0,72	-0,47	-1,85	0,06	374	**0,37**	0,93	-0,11	-0,43	0,67	1380	**1,00**	0,84	3,00	-0,25	0,11
**PA0143**	40205	7,03	392,66	10	29,42	0,83	-0,26	-1,00	0,32	772	**0,89**	0,78	-0,37	-1,46	0,14	508	**0,61**	0,75	-0,42	-1,49	0,14	469	**0,60**	0,79	3,00	-0,35	0,04
**PA0148**	38008	5,08	298,92	10	25,84	0,73	-0,45	-1,65	0,10	396	**0,54**	0,82	-0,29	-1,19	0,23	637	**0,79**	0,85	-0,23	-0,84	0,40	899	**0,92**	0,80	3,00	-0,32	0,06
**PA0155**	31513	7,15	199,50	6	22,83	1,01	0,02	-0,01	1,00	2167	**1,00**	0,92	-0,13	-0,57	0,57	1268	**0,97**	1,22	0,29	0,93	0,35	818	**0,89**	1,05	3,00	0,07	0,16
**PA0156**	42590	6,12	166,31	4	14,33	1,06	0,08	0,21	0,84	1832	**0,99**	1,00	0,00	-0,09	0,93	2025	**0,99**	0,93	-0,10	-0,41	0,69	1403	**1,01**	1,00	3,00	0,00	0,06
**PA0157**	40034	8,72	246,36	7	17,31	0,87	-0,20	-0,79	0,43	986	**0,94**	0,78	-0,36	-1,44	0,15	514	**0,63**	0,95	-0,08	-0,31	0,75	1557	**1,00**	0,87	3,00	-0,21	0,08
**PA0158**	117979	5,79	130,54	4	4,70	1,12	0,17	0,50	0,62	1354	**0,99**	0,96	-0,06	-0,31	0,76	1676	**0,98**	0,96	-0,06	-0,28	0,78	1612	**1,00**	1,01	3,00	0,02	0,09
**PA0162**	51466	4,97	1011,39	19	34,99	1,24	0,30	0,99	0,32	787	**0,89**	1,06	0,08	0,22	0,83	1826	**0,98**	1,24	0,31	1,00	0,32	759	**0,87**	1,18	3,00	0,24	0,10
**PA0165**	33573	5,08	342,44	9	22,60	0,82	-0,29	-1,11	0,27	689	**0,84**	0,80	-0,31	-1,26	0,21	601	**0,74**	0,87	-0,20	-0,74	0,46	1019	**0,93**	0,83	3,00	-0,27	0,04
**PA0167**	25906	8,55	86,35	2	10,90	1,05	0,07	0,16	0,87	1913	**0,99**	0,91	-0,14	-0,62	0,53	1200	**0,96**	0,94	-0,08	-0,34	0,73	1513	**1,00**	0,97	3,00	-0,05	0,07
**PA0169**	27877	9,26	76,06	2	18,70	1,77	0,83	2,82	0,00	166	**0,06**	2,38	1,25	4,57	0,00	99	**0,00**	2,07	1,05	3,49	0,00	136	**0,01**	2,07	3,00	1,05	0,31
**PA0170**	14878	4,59	143,53	4	52,00	1,92	0,94	3,23	0,00	130	**0,02**	2,09	1,06	3,86	0,00	124	**0,00**	1,81	0,86	2,84	0,00	182	**0,05**	1,94	3,00	0,96	0,14
**PA0171**	21033	6,52	187,93	6	44,52	2,51	1,33	4,58	0,00	80	**0,00**	2,39	1,26	4,59	0,00	97	**0,00**	3,25	1,70	5,70	0,00	66	**0,00**	2,72	3,00	1,44	0,46
**PA0195**	41116	5,73	194,93	5	10,84	1,45	0,53	1,79	0,07	340	**0,47**	1,18	0,24	0,80	0,43	983	**0,94**	1,70	0,77	2,54	0,01	220	**0,10**	1,44	3,00	0,53	0,26
**PA0236**	28727	7,96	188,11	5	15,92	0,76	-0,39	-1,44	0,15	493	**0,66**	1,11	0,16	0,49	0,63	1411	**0,96**	0,97	-0,04	-0,20	0,85	1736	**1,00**	0,95	3,00	-0,07	0,18
**PA0250**	16856	5,58	139,99	3	27,10	0,99	-0,02	-0,14	0,89	1959	**0,99**	0,54	-0,88	-3,39	0,00	150	**0,01**	1,29	0,37	1,19	0,23	621	**0,78**	0,94	3,00	-0,09	0,38
**PA0265**	56719	5,60	1446,09	35	58,32	0,93	-0,10	-0,42	0,67	1482	**0,99**	0,89	-0,17	-0,74	0,46	1042	**0,95**	0,82	-0,28	-1,02	0,31	742	**0,85**	0,88	3,00	-0,18	0,06
**PA0266**	48782	6,05	2205,78	51	65,18	0,95	-0,08	-0,36	0,72	1590	**0,99**	0,84	-0,25	-1,01	0,31	785	**0,86**	0,81	-0,31	-1,12	0,26	656	**0,83**	0,87	3,00	-0,21	0,07
**PA0269**	17195	5,43	70,86	2	12,40	1,01	0,02	-0,02	0,98	2148	**1,00**	1,16	0,21	0,69	0,49	1100	**0,96**	1,13	0,17	0,52	0,60	1253	**0,99**	1,10	3,00	0,14	0,08
**PA0277**	28811	6,08	685,55	11	29,68	1,04	0,06	0,14	0,89	1958	**0,99**	0,88	-0,18	-0,76	0,45	1023	**0,95**	0,98	-0,03	-0,14	0,89	1820	**1,00**	0,97	3,00	-0,04	0,08
**PA0280**	39124	5,77	168,64	6	14,10	0,99	-0,01	-0,13	0,90	1966	**0,99**	1,02	0,03	0,01	0,99	2150	**1,00**	1,16	0,22	0,68	0,49	1078	**0,95**	1,06	3,00	0,08	0,09
**PA0285**	89486	5,90	289,58	8	13,81	1,08	0,11	0,29	0,77	1688	**0,99**	0,99	-0,01	-0,15	0,88	1952	**0,98**	0,92	-0,12	-0,45	0,65	1351	**1,00**	1,00	3,00	0,00	0,08
**PA0291**	53095	8,67	2137,74	43	60,42	1,23	0,30	0,99	0,32	788	**0,89**	1,20	0,26	0,87	0,38	885	**0,93**	1,22	0,29	0,92	0,36	820	**0,89**	1,22	3,00	0,28	0,02
**PA0292**	42928	4,84	460,70	9	27,33	0,61	-0,72	-2,61	0,01	189	**0,10**	0,99	-0,02	-0,18	0,86	1893	**0,98**	0,93	-0,10	-0,41	0,69	1405	**1,01**	0,84	3,00	-0,25	0,20
**PA0293**	34030	5,69	249,89	9	28,78	1,08	0,11	0,31	0,76	1667	**0,99**	1,37	0,45	1,59	0,11	459	**0,53**	1,12	0,16	0,48	0,63	1297	**1,00**	1,19	3,00	0,25	0,16
**PA0295**	40490	5,66	123,87	4	18,10	0,61	-0,71	-2,57	0,01	191	**0,12**	0,82	-0,28	-1,16	0,25	658	**0,81**	0,92	-0,12	-0,47	0,64	1310	**1,00**	0,78	3,00	-0,35	0,16
**PA0296**	53411	4,82	792,67	19	47,10	0,96	-0,06	-0,27	0,78	1730	**0,99**	0,98	-0,03	-0,20	0,84	1861	**0,98**	0,97	-0,04	-0,21	0,83	1709	**1,01**	0,97	3,00	-0,04	0,01
**PA0297**	27607	5,97	91,17	2	13,20	1,10	0,14	0,42	0,68	1495	**0,98**	1,78	0,83	3,00	0,00	177	**0,03**	1,67	0,74	2,46	0,01	231	**0,12**	1,52	3,00	0,60	0,36
**PA0298**	54621	5,22	536,81	15	29,30	1,47	0,55	1,86	0,06	310	**0,44**	1,43	0,51	1,82	0,07	385	**0,39**	1,43	0,51	1,69	0,09	381	**0,50**	1,44	3,00	0,53	0,02
**PA0299**	54253	5,71	1018,69	23	37,22	1,19	0,25	0,81	0,42	974	**0,94**	1,26	0,33	1,13	0,26	686	**0,82**	1,28	0,36	1,16	0,24	632	**0,80**	1,24	3,00	0,31	0,05
**PA0300**	46316	6,97	1135,28	25	44,64	1,28	0,36	1,17	0,24	648	**0,80**	1,62	0,70	2,51	0,01	230	**0,11**	1,30	0,37	1,21	0,23	606	**0,77**	1,40	3,00	0,49	0,19
**PA0301**	44748	5,51	330,89	10	21,30	0,39	-1,35	-4,83	0,00	74	**0,00**	0,74	-0,44	-1,75	0,08	402	**0,43**	0,82	-0,29	-1,05	0,29	710	**0,85**	0,65	3,00	-0,63	0,23
**PA0302**	45966	6,02	550,13	12	22,40	1,02	0,03	0,04	0,97	2114	**1,00**	1,14	0,18	0,59	0,56	1233	**0,97**	1,10	0,13	0,39	0,69	1422	**1,01**	1,09	3,00	0,12	0,06
**PA0308**	39126	6,32	118,94	3	12,10	1,15	0,20	0,63	0,53	1162	**0,99**	1,13	0,17	0,54	0,59	1314	**0,97**	1,02	0,03	0,03	0,97	2009	**1,00**	1,10	3,00	0,14	0,07
**PA0309**	27870	6,75	435,99	8	26,55	0,85	-0,24	-0,91	0,36	863	**0,92**	0,96	-0,06	-0,33	0,74	1646	**0,98**	0,95	-0,07	-0,28	0,78	1598	**1,00**	0,92	3,00	-0,12	0,06
**PA0314**	30870	9,11	511,40	13	49,98	1,25	0,32	1,03	0,30	746	**0,88**	1,11	0,15	0,48	0,63	1422	**0,96**	1,17	0,23	0,72	0,47	1045	**0,93**	1,18	3,00	0,24	0,07
**PA0315**	17228	6,58	357,37	8	50,43	0,91	-0,14	-0,56	0,57	1269	**0,98**	0,92	-0,11	-0,52	0,60	1347	**0,97**	0,95	-0,07	-0,31	0,76	1564	**1,00**	0,93	3,00	-0,11	0,02
**PA0316**	48120	6,08	1403,15	30	56,85	1,06	0,08	0,21	0,83	1821	**0,99**	1,11	0,15	0,45	0,65	1455	**0,97**	1,02	0,02	0,02	0,98	2024	**1,00**	1,06	3,00	0,09	0,05
**PA0317**	55617	5,13	953,32	25	37,50	0,91	-0,13	-0,54	0,59	1287	**0,99**	1,02	0,03	0,03	0,98	2128	**0,99**	1,02	0,03	0,03	0,97	2006	**1,00**	0,98	3,00	-0,02	0,06
**PA0318**	25361	5,36	278,90	7	34,13	0,76	-0,39	-1,46	0,14	474	**0,66**	0,71	-0,49	-1,94	0,05	345	**0,33**	0,73	-0,45	-1,57	0,12	428	**0,56**	0,73	3,00	-0,44	0,03
**PA0319**	38497	5,07	74,82	3	13,70	0,98	-0,03	-0,18	0,85	1876	**0,99**	1,13	0,18	0,58	0,56	1248	**0,97**	1,10	0,13	0,40	0,69	1420	**1,00**	1,07	3,00	0,10	0,08

**PA0328**	71867	4,84	536,34	16	21,56	0,71	-0,50	-1,84	0,07	318	**0,45**	0,84	-0,25	-1,03	0,30	763	**0,85**	0,73	-0,45	-1,59	0,11	423	**0,55**	0,76	3,00	-0,40	0,07
**PA0329**	13495	9,26	108,20	2	22,75	0,95	-0,08	-0,36	0,72	1586	**0,99**	0,83	-0,28	-1,13	0,26	684	**0,82**	0,83	-0,27	-0,99	0,32	768	**0,87**	0,87	3,00	-0,21	0,07
**PA0330**	25770	5,38	1060,39	19	67,35	1,09	0,13	0,36	0,72	1585	**0,98**	1,06	0,08	0,22	0,83	1827	**0,98**	1,09	0,12	0,36	0,72	1470	**1,00**	1,08	3,00	0,11	0,02
**PA0331**	59103	6,05	1051,23	24	36,92	0,94	-0,08	-0,37	0,71	1566	**0,98**	0,95	-0,07	-0,37	0,71	1590	**0,97**	1,02	0,03	0,03	0,98	2014	**1,00**	0,97	3,00	-0,04	0,04
**PA0335**	25341	4,78	326,07	8	38,84	1,02	0,03	0,03	0,97	2125	**1,00**	0,83	-0,28	-1,13	0,26	683	**0,82**	0,87	-0,20	-0,75	0,45	1002	**0,93**	0,91	3,00	-0,14	0,10
**PA0337**	88159	5,53	589,49	13	20,28	1,00	0,01	-0,06	0,95	2076	**1,00**	1,03	0,05	0,07	0,94	2052	**0,99**	0,99	-0,02	-0,12	0,91	1866	**1,00**	1,01	3,00	0,01	0,02
**PA0341**	31407	9,61	94,73	3	9,03	1,03	0,04	0,07	0,94	2050	**1,00**	0,92	-0,13	-0,57	0,57	1271	**0,97**	0,92	-0,13	-0,49	0,63	1289	**1,00**	0,95	3,00	-0,07	0,07
**PA0342**	32198	6,04	299,60	9	25,80	0,89	-0,17	-0,69	0,49	1089	**0,97**	0,94	-0,08	-0,41	0,68	1523	**0,97**	1,00	-0,01	-0,08	0,94	1932	**1,00**	0,94	3,00	-0,09	0,06
**PA0350**	18912	5,45	189,43	5	31,50	0,85	-0,23	-0,90	0,37	874	**0,91**	0,79	-0,34	-1,35	0,18	552	**0,70**	0,91	-0,13	-0,51	0,61	1263	**0,99**	0,85	3,00	-0,23	0,06
**PA0353**	70472	5,71	1085,50	21	27,58	0,62	-0,70	-2,54	0,01	197	**0,12**	0,82	-0,28	-1,15	0,25	665	**0,82**	0,77	-0,38	-1,36	0,18	540	**0,67**	0,73	3,00	-0,44	0,11
**PA0354**	46855	6,48	257,65	6	16,47	0,95	-0,07	-0,34	0,73	1608	**0,99**	0,87	-0,21	-0,87	0,38	892	**0,93**	1,04	0,06	0,14	0,89	1828	**1,00**	0,95	3,00	-0,07	0,09
**PA0356**	31822	5,33	312,13	7	21,20	0,98	-0,03	-0,20	0,84	1846	**0,99**	1,05	0,07	0,17	0,87	1910	**0,98**	0,89	-0,17	-0,62	0,53	1147	**0,96**	0,97	3,00	-0,04	0,08
**PA0357**	31220	9,43	135,09	5	18,72	0,94	-0,09	-0,40	0,69	1520	**0,99**	0,90	-0,16	-0,68	0,49	1117	**0,96**	0,82	-0,29	-1,03	0,30	731	**0,86**	0,89	3,00	-0,18	0,06
**PA0363**	19201	8,04	736,28	16	81,80	0,81	-0,30	-1,13	0,26	676	**0,83**	0,88	-0,18	-0,78	0,43	997	**0,94**	0,94	-0,08	-0,34	0,73	1504	**1,00**	0,88	3,00	-0,19	0,07
**PA0370**	22692	9,06	166,98	4	18,93	0,91	-0,14	-0,57	0,57	1265	**0,98**	0,90	-0,16	-0,69	0,49	1104	**0,96**	0,75	-0,42	-1,48	0,14	477	**0,61**	0,85	3,00	-0,23	0,09
**PA0371**	56998	5,67	688,27	16	29,38	0,95	-0,08	-0,36	0,72	1587	**0,99**	0,98	-0,03	-0,23	0,82	1814	**0,98**	1,00	0,00	-0,06	0,95	1963	**1,00**	0,97	3,00	-0,04	0,03
**PA0372**	55271	6,17	668,57	17	31,39	0,89	-0,16	-0,66	0,51	1128	**0,98**	1,06	0,08	0,20	0,84	1854	**0,98**	0,94	-0,08	-0,34	0,74	1517	**1,00**	0,96	3,00	-0,05	0,08
**PA0373**	52253	4,81	853,39	15	30,10	1,05	0,07	0,18	0,86	1886	**0,99**	1,17	0,23	0,76	0,45	1026	**0,94**	1,08	0,11	0,32	0,75	1544	**1,00**	1,10	3,00	0,14	0,06
**PA0374**	25750	10,15	416,69	11	42,58	1,00	0,01	-0,06	0,95	2077	**1,00**	0,99	-0,01	-0,15	0,88	1948	**0,98**	0,87	-0,20	-0,72	0,47	1036	**0,93**	0,96	3,00	-0,07	0,07
**PA0375**	38105	5,40	104,79	4	10,70	0,94	-0,10	-0,42	0,68	1491	**0,98**	1,12	0,17	0,52	0,60	1350	**0,97**	0,99	-0,01	-0,10	0,92	1900	**1,00**	1,02	3,00	0,02	0,10
**PA0381**	30032	5,01	990,58	22	60,20	0,90	-0,15	-0,59	0,55	1217	**0,99**	0,91	-0,13	-0,60	0,55	1221	**0,97**	0,93	-0,10	-0,40	0,69	1421	**1,00**	0,92	3,00	-0,13	0,02
**PA0382**	26992	7,88	188,74	7	23,07	0,86	-0,22	-0,85	0,39	920	**0,93**	1,08	0,11	0,29	0,77	1693	**0,98**	1,04	0,05	0,12	0,90	1853	**1,00**	0,99	3,00	-0,01	0,12
**PA0387**	22762	5,85	580,41	14	64,03	1,00	0,00	-0,09	0,93	2029	**1,00**	1,05	0,07	0,15	0,88	1944	**0,98**	1,08	0,11	0,33	0,75	1537	**1,00**	1,04	3,00	0,06	0,04
**PA0388**	16636	6,60	534,16	13	49,69	1,05	0,07	0,15	0,88	1938	**0,99**	0,98	-0,03	-0,22	0,82	1820	**0,98**	0,94	-0,08	-0,34	0,74	1524	**1,00**	0,99	3,00	-0,02	0,05
**PA0389**	24754	6,06	289,88	7	22,30	0,99	-0,01	-0,12	0,90	1977	**0,99**	0,85	-0,23	-0,95	0,34	829	**0,89**	1,01	0,02	0,00	1,00	2059	**1,00**	0,95	3,00	-0,07	0,09
**PA0390**	44245	5,64	623,49	11	30,55	0,89	-0,16	-0,65	0,52	1137	**0,99**	0,98	-0,03	-0,21	0,83	1831	**0,98**	0,99	-0,02	-0,12	0,91	1858	**1,00**	0,95	3,00	-0,07	0,05
**PA0393**	29269	4,78	490,58	10	28,20	1,10	0,14	0,41	0,68	1505	**0,99**	0,96	-0,06	-0,32	0,75	1653	**0,98**	1,10	0,13	0,39	0,69	1425	**1,00**	1,05	3,00	0,07	0,08
**PA0394**	25879	6,22	195,85	5	22,86	1,15	0,20	0,62	0,54	1179	**0,99**	0,92	-0,13	-0,57	0,57	1281	**0,96**	1,18	0,24	0,76	0,45	983	**0,94**	1,08	3,00	0,11	0,14
**PA0395**	40971	6,38	492,24	13	32,10	0,53	-0,91	-3,28	0,00	128	**0,02**	0,57	-0,82	-3,15	0,00	169	**0,02**	0,52	-0,95	-3,27	0,00	150	**0,01**	0,54	3,00	-0,89	0,02
**PA0396**	45238	6,33	246,28	7	19,89	0,51	-0,98	-3,51	0,00	116	**0,01**	0,44	-1,18	-4,50	0,00	101	**0,00**	0,44	-1,19	-4,10	0,00	110	**0,00**	0,46	3,00	-1,11	0,04
**PA0399**	51342	5,47	1477,96	27	52,22	1,02	0,03	0,01	0,99	2161	**0,99**	0,85	-0,23	-0,96	0,34	820	**0,89**	0,86	-0,22	-0,79	0,43	948	**0,94**	0,91	3,00	-0,14	0,09
**PA0400**	43809	6,16	467,73	10	25,38	0,98	-0,03	-0,17	0,87	1902	**0,99**	0,89	-0,16	-0,70	0,48	1093	**0,96**	0,93	-0,11	-0,44	0,66	1362	**1,00**	0,93	3,00	-0,10	0,04
**PA0401**	45506	5,56	803,63	17	26,44	0,97	-0,05	-0,25	0,80	1764	**0,99**	1,05	0,07	0,15	0,88	1943	**0,98**	0,99	-0,01	-0,09	0,93	1908	**1,00**	1,00	3,00	0,00	0,04
**PA0402**	38571	6,33	843,49	19	48,07	0,92	-0,12	-0,51	0,61	1342	**0,99**	1,03	0,04	0,04	0,97	2110	**0,99**	0,98	-0,03	-0,16	0,87	1798	**1,00**	0,97	3,00	-0,04	0,05
**PA0403**	19143	5,56	266,41	7	37,19	0,87	-0,20	-0,80	0,43	977	**0,95**	0,88	-0,19	-0,79	0,43	988	**0,94**	0,86	-0,21	-0,77	0,44	970	**0,93**	0,87	3,00	-0,20	0,01
**PA0407**	37451	5,68	549,96	17	47,30	0,96	-0,06	-0,30	0,77	1683	**0,99**	0,99	-0,02	-0,17	0,86	1903	**0,98**	1,00	0,00	-0,05	0,96	1975	**1,00**	0,98	3,00	-0,03	0,02
**PA0408**	16818	6,71	348,83	11	43,10	0,54	-0,90	-3,23	0,00	129	**0,02**	0,64	-0,63	-2,46	0,01	240	**0,13**	0,55	-0,85	-2,94	0,00	176	**0,04**	0,58	3,00	-0,79	0,06
**PA0409**	14850	5,35	827,00	16	78,50	0,54	-0,89	-3,21	0,00	135	**0,02**	0,57	-0,81	-3,13	0,00	171	**0,02**	0,55	-0,87	-2,99	0,00	172	**0,03**	0,55	3,00	-0,86	0,01
**PA0410**	20590	6,14	245,61	4	28,38	0,75	-0,42	-1,54	0,12	442	**0,60**	0,68	-0,56	-2,19	0,03	290	**0,21**	0,65	-0,62	-2,15	0,03	278	**0,23**	0,69	3,00	-0,53	0,05
**PA0411**	76087	4,65	1323,20	23	31,52	0,59	-0,77	-2,77	0,01	173	**0,07**	0,51	-0,98	-3,77	0,00	129	**0,00**	0,49	-1,03	-3,53	0,00	132	**0,01**	0,53	3,00	-0,92	0,05
**PA0413**	275166	4,43	2590,61	60	23,59	0,64	-0,63	-2,31	0,02	231	**0,20**	0,64	-0,64	-2,50	0,01	232	**0,12**	0,59	-0,76	-2,64	0,01	212	**0,08**	0,62	3,00	-0,68	0,03
**PA0415**	18047	4,91	531,42	10	61,90	0,54	-0,88	-3,17	0,00	137	**0,02**	0,61	-0,71	-2,74	0,01	206	**0,07**	0,58	-0,77	-2,68	0,01	207	**0,07**	0,58	3,00	-0,79	0,03
**PA0418**	52869	5,56	195,59	7	14,61	0,98	-0,03	-0,17	0,87	1904	**0,99**	0,78	-0,36	-1,43	0,15	518	**0,63**	1,03	0,05	0,11	0,91	1877	**1,00**	0,93	3,00	-0,10	0,13
**PA0419**	27454	6,15	395,01	8	36,45	1,06	0,08	0,21	0,84	1833	**0,99**	1,13	0,18	0,57	0,57	1278	**0,97**	1,47	0,55	1,81	0,07	343	**0,42**	1,22	3,00	0,28	0,22
**PA0420**	54928	5,86	757,34	18	38,94	0,76	-0,40	-1,50	0,13	460	**0,64**	0,80	-0,32	-1,30	0,19	572	**0,74**	0,81	-0,31	-1,10	0,27	664	**0,84**	0,79	3,00	-0,34	0,03
**PA0421**	58785	9,76	963,25	25	41,92	1,01	0,01	-0,05	0,96	2098	**0,99**	0,95	-0,08	-0,40	0,69	1540	**0,97**	1,11	0,15	0,45	0,65	1338	**1,00**	1,02	3,00	0,03	0,08
**PA0423**	23357	6,09	902,35	23	67,90	1,44	0,53	1,78	0,07	345	**0,47**	1,27	0,35	1,20	0,23	630	**0,79**	1,39	0,48	1,56	0,12	434	**0,56**	1,37	3,00	0,45	0,09
**PA0425**	44305	8,75	1250,57	22	48,92	1,58	0,66	2,22	0,03	249	**0,23**	1,51	0,59	2,12	0,03	301	**0,24**	1,51	0,60	1,97	0,05	306	**0,33**	1,53	3,00	0,62	0,04

**PA0426**	118765	5,51	1381,54	35	21,42	1,62	0,70	2,36	0,02	225	**0,17**	1,48	0,56	2,00	0,05	326	**0,30**	1,48	0,57	1,87	0,06	327	**0,38**	1,53	3,00	0,61	0,08
**PA0427**	54486	5,52	1637,06	34	48,32	1,54	0,63	2,12	0,03	261	**0,29**	1,58	0,66	2,36	0,02	259	**0,15**	1,64	0,71	2,36	0,02	248	**0,15**	1,59	3,00	0,67	0,05
**PA0428**	75782	9,98	1344,31	31	42,41	0,98	-0,03	-0,20	0,84	1847	**0,99**	0,94	-0,09	-0,42	0,67	1498	**0,97**	1,01	0,01	-0,02	0,98	2032	**1,00**	0,97	3,00	-0,04	0,03
**PA0429**	42852	6,50	388,40	9	24,74	1,00	0,01	-0,06	0,95	2069	**1,00**	1,30	0,37	1,29	0,20	575	**0,73**	1,07	0,09	0,25	0,80	1649	**1,00**	1,12	3,00	0,17	0,15
**PA0430**	34234	6,13	765,44	17	53,83	1,14	0,19	0,59	0,56	1223	**0,99**	1,06	0,09	0,23	0,82	1810	**0,98**	1,13	0,18	0,55	0,58	1220	**0,98**	1,11	3,00	0,15	0,04
**PA0432**	56779	5,71	2526,25	58	69,75	1,01	0,01	-0,05	0,96	2090	**1,00**	0,92	-0,11	-0,52	0,60	1340	**0,97**	0,97	-0,04	-0,19	0,85	1756	**1,00**	0,97	3,00	-0,05	0,04
**PA0437**	48578	5,66	396,98	11	23,46	0,97	-0,05	-0,24	0,81	1779	**0,99**	1,09	0,13	0,37	0,71	1577	**0,97**	1,04	0,05	0,11	0,91	1871	**1,00**	1,03	3,00	0,04	0,06
**PA0446**	46119	5,36	470,76	11	25,55	3,14	1,65	5,72	0,00	59	**0,00**	2,10	1,07	3,89	0,00	123	**0,00**	2,85	1,51	5,06	0,00	85	**0,00**	2,70	3,00	1,43	0,54
**PA0447**	46009	5,84	710,70	15	47,70	2,63	1,39	4,81	0,00	76	**0,00**	1,72	0,79	2,83	0,00	194	**0,05**	2,06	1,04	3,47	0,00	139	**0,01**	2,14	3,00	1,09	0,46
**PA0449**	21282	9,86	286,57	8	25,10	0,83	-0,26	-1,00	0,32	777	**0,89**	1,10	0,14	0,42	0,67	1501	**0,97**	0,95	-0,08	-0,32	0,75	1551	**1,00**	0,96	3,00	-0,06	0,13
**PA0455**	52971	8,57	839,44	20	33,20	1,00	0,01	-0,06	0,95	2082	**1,00**	0,96	-0,05	-0,29	0,77	1699	**0,98**	1,02	0,03	0,04	0,97	1992	**1,00**	1,00	3,00	-0,01	0,03
**PA0456**	8566	8,09	452,37	9	67,63	0,90	-0,16	-0,63	0,53	1165	**0,99**	0,85	-0,24	-0,98	0,33	804	**0,88**	0,96	-0,06	-0,25	0,80	1646	**1,00**	0,90	3,00	-0,15	0,06
**PA0459**	102588	6,32	223,13	5	8,78	0,91	-0,14	-0,57	0,57	1256	**0,98**	0,79	-0,35	-1,39	0,17	536	**0,67**	0,82	-0,28	-1,01	0,31	748	**0,86**	0,84	3,00	-0,25	0,06
**PA0460**	20301	5,67	415,23	7	33,99	1,08	0,11	0,29	0,77	1696	**0,99**	0,87	-0,20	-0,83	0,41	938	**0,93**	1,06	0,09	0,25	0,80	1652	**1,00**	1,00	3,00	0,01	0,11
**PA0462**	28225	10,04	65,48	2	9,80	1,00	0,00	-0,09	0,93	2009	**1,00**	0,88	-0,19	-0,79	0,43	986	**0,94**	1,00	0,00	-0,06	0,95	1961	**1,00**	0,96	3,00	-0,06	0,07
**PA0463**	26612	5,46	71,78	3	6,27	1,01	0,02	-0,02	0,98	2138	**1,00**	0,79	-0,33	-1,34	0,18	554	**0,71**	0,94	-0,09	-0,38	0,70	1448	**1,00**	0,91	3,00	-0,13	0,11
**PA0469**	32878	4,94	309,81	8	22,23	0,70	-0,51	-1,86	0,06	307	**0,44**	0,76	-0,40	-1,57	0,12	466	**0,54**	0,57	-0,81	-2,80	0,01	192	**0,05**	0,68	3,00	-0,56	0,10
**PA0470**	92574	5,46	78,69	2	3,10	1,45	0,53	1,79	0,07	342	**0,47**	3,48	1,80	6,61	0,00	48	**0,00**	1,27	0,35	1,12	0,26	653	**0,83**	2,07	3,00	1,05	1,23
**PA0473**	29037	5,74	227,96	5	16,16	1,54	0,62	2,11	0,04	263	**0,29**	2,01	1,01	3,65	0,00	135	**0,00**	1,80	0,85	2,82	0,00	187	**0,05**	1,78	3,00	0,83	0,23
**PA0482**	84657	5,47	1566,69	40	43,53	0,70	-0,51	-1,85	0,06	313	**0,44**	0,76	-0,39	-1,55	0,12	474	**0,55**	0,76	-0,40	-1,41	0,16	516	**0,63**	0,74	3,00	-0,43	0,03
**PA0486**	37562	4,96	228,06	7	18,84	0,77	-0,38	-1,40	0,16	513	**0,69**	0,85	-0,23	-0,95	0,34	830	**0,89**	0,92	-0,12	-0,45	0,65	1347	**1,00**	0,85	3,00	-0,24	0,08
**PA0487**	28275	8,82	138,13	3	20,60	1,08	0,12	0,33	0,74	1632	**0,99**	1,13	0,18	0,57	0,57	1275	**0,96**	0,76	-0,40	-1,43	0,15	509	**0,62**	0,99	3,00	-0,01	0,21
**PA0500**	42535	5,75	680,35	17	42,22	1,06	0,08	0,21	0,83	1828	**0,99**	0,98	-0,02	-0,18	0,85	1886	**0,98**	1,09	0,13	0,38	0,71	1458	**1,00**	1,05	3,00	0,06	0,06
**PA0501**	43023	5,65	93,95	3	13,00	1,08	0,11	0,30	0,77	1684	**0,99**	1,02	0,03	0,01	1,00	2152	**1,00**	1,07	0,10	0,28	0,78	1602	**1,00**	1,06	3,00	0,08	0,03
**PA0502**	26904	4,50	141,86	4	15,80	0,97	-0,04	-0,22	0,82	1809	**0,99**	1,09	0,12	0,36	0,72	1607	**0,97**	1,07	0,10	0,29	0,77	1595	**1,00**	1,04	3,00	0,06	0,06
**PA0503**	30454	9,06	187,10	4	15,55	1,18	0,24	0,77	0,44	1004	**0,95**	1,16	0,22	0,72	0,47	1063	**0,96**	1,11	0,16	0,47	0,64	1307	**1,00**	1,15	3,00	0,21	0,04
**PA0504**	23939	5,36	131,46	4	15,05	1,25	0,32	1,03	0,30	749	**0,88**	0,94	-0,08	-0,41	0,68	1515	**0,97**	1,08	0,11	0,31	0,76	1563	**1,00**	1,09	3,00	0,12	0,15
**PA0506**	70197	5,62	868,85	23	27,47	0,86	-0,21	-0,83	0,41	939	**0,94**	0,90	-0,15	-0,66	0,51	1134	**0,97**	0,93	-0,11	-0,43	0,67	1371	**1,00**	0,90	3,00	-0,16	0,03
**PA0508**	67755	5,29	581,95	18	27,14	0,85	-0,24	-0,92	0,36	848	**0,91**	0,83	-0,27	-1,09	0,28	722	**0,83**	0,88	-0,19	-0,70	0,48	1054	**0,94**	0,85	3,00	-0,23	0,02
**PA0527**	27207	6,21	91,58	3	12,47	0,88	-0,19	-0,73	0,46	1040	**0,97**	0,84	-0,25	-1,02	0,31	777	**0,86**	1,45	0,53	1,75	0,08	361	**0,45**	1,06	3,00	0,08	0,34
**PA0536**	37160	5,39	175,57	4	13,80	1,19	0,25	0,79	0,43	989	**0,95**	1,11	0,15	0,45	0,65	1457	**0,97**	1,40	0,49	1,60	0,11	415	**0,54**	1,23	3,00	0,30	0,15
**PA0537**	23942	8,63	604,38	13	45,91	0,93	-0,10	-0,42	0,67	1483	**0,98**	1,08	0,12	0,33	0,74	1634	**0,98**	1,16	0,22	0,67	0,50	1091	**0,95**	1,06	3,00	0,08	0,12
**PA0541**	19123	8,46	202,42	5	22,24	0,93	-0,10	-0,43	0,67	1465	**0,99**	1,05	0,07	0,17	0,87	1911	**0,98**	1,30	0,38	1,23	0,22	591	**0,77**	1,09	3,00	0,13	0,19
**PA0542**	17300	8,53	88,34	3	20,37	0,88	-0,18	-0,71	0,48	1076	**0,97**	0,86	-0,21	-0,89	0,37	868	**0,93**	0,98	-0,03	-0,15	0,88	1810	**1,00**	0,91	3,00	-0,14	0,06
**PA0546**	46129	5,26	909,47	23	41,88	0,78	-0,35	-1,31	0,19	559	**0,74**	0,87	-0,20	-0,85	0,39	915	**0,93**	0,85	-0,24	-0,87	0,38	866	**0,91**	0,83	3,00	-0,26	0,04
**PA0548**	76243	5,16	1766,94	37	43,60	0,98	-0,03	-0,17	0,86	1897	**0,99**	0,88	-0,18	-0,78	0,44	1002	**0,94**	0,88	-0,18	-0,68	0,50	1080	**0,95**	0,91	3,00	-0,13	0,06
**PA0551**	39906	5,61	193,42	5	13,90	1,20	0,26	0,84	0,40	932	**0,93**	1,13	0,18	0,58	0,56	1247	**0,97**	0,96	-0,05	-0,24	0,81	1668	**1,00**	1,10	3,00	0,14	0,12
**PA0552**	44985	5,28	1646,91	31	60,96	0,85	-0,23	-0,89	0,37	886	**0,92**	0,93	-0,10	-0,48	0,63	1419	**0,96**	0,94	-0,10	-0,38	0,70	1444	**1,00**	0,91	3,00	-0,14	0,05
**PA0555**	41327	5,34	1314,22	32	59,59	0,89	-0,17	-0,68	0,50	1105	**0,98**	0,82	-0,29	-1,17	0,24	643	**0,81**	0,86	-0,22	-0,79	0,43	946	**0,94**	0,86	3,00	-0,22	0,04
**PA0559**	45279	6,11	109,91	4	13,30	1,12	0,16	0,47	0,64	1405	**0,99**	1,01	0,01	-0,05	0,96	2077	**1,00**	1,05	0,07	0,17	0,87	1783	**1,00**	1,06	3,00	0,08	0,05
**PA0562**	24741	5,41	119,78	3	14,00	0,77	-0,37	-1,39	0,16	514	**0,69**	0,84	-0,26	-1,05	0,29	748	**0,84**	1,11	0,15	0,46	0,65	1336	**1,00**	0,91	3,00	-0,14	0,18
**PA0571**	23149	5,14	135,54	4	18,18	0,94	-0,09	-0,40	0,69	1512	**0,99**	0,82	-0,28	-1,14	0,25	677	**0,81**	0,94	-0,09	-0,35	0,73	1502	**1,00**	0,90	3,00	-0,15	0,07
**PA0576**	75266	4,90	1706,75	48	42,71	0,89	-0,16	-0,66	0,51	1129	**0,98**	0,97	-0,04	-0,24	0,81	1800	**0,98**	0,89	-0,16	-0,60	0,55	1170	**0,96**	0,92	3,00	-0,12	0,05
**PA0577**	80412	6,79	378,84	10	13,82	1,03	0,04	0,07	0,95	2057	**1,00**	1,07	0,09	0,26	0,80	1758	**0,98**	1,15	0,21	0,64	0,52	1121	**0,96**	1,08	3,00	0,12	0,06
**PA0578**	18129	5,91	177,66	6	29,50	1,03	0,05	0,09	0,93	2022	**1,00**	1,10	0,13	0,40	0,69	1534	**0,97**	1,21	0,28	0,89	0,37	841	**0,91**	1,12	3,00	0,16	0,09
**PA0579**	10111	10,78	115,12	3	38,50	1,13	0,18	0,56	0,58	1278	**0,98**	0,90	-0,15	-0,66	0,51	1142	**0,96**	0,83	-0,28	-1,00	0,32	760	**0,87**	0,95	3,00	-0,07	0,16
**PA0580**	38347	5,84	396,80	9	24,43	0,84	-0,25	-0,94	0,35	826	**0,91**	0,99	-0,02	-0,16	0,87	1923	**0,98**	0,95	-0,08	-0,32	0,75	1550	**1,00**	0,93	3,00	-0,11	0,07
**PA0582**	13480	4,91	62,83	3	14,50	1,08	0,11	0,32	0,75	1640	**0,99**	0,87	-0,20	-0,85	0,40	925	**0,93**	1,09	0,13	0,37	0,71	1460	**1,00**	1,02	3,00	0,02	0,13

**PA0583**	20989	5,53	248,81	6	35,80	1,24	0,31	1,00	0,32	779	**0,89**	1,23	0,29	1,00	0,32	795	**0,86**	1,17	0,23	0,72	0,47	1039	**0,93**	1,21	3,00	0,28	0,03
**PA0588**	80391	5,58	320,87	7	11,46	0,72	-0,46	-1,71	0,09	371	**0,51**	0,95	-0,07	-0,36	0,72	1605	**0,97**	0,81	-0,30	-1,09	0,28	674	**0,84**	0,83	3,00	-0,27	0,12
**PA0590**	34626	6,72	166,36	7	22,00	1,12	0,16	0,49	0,63	1383	**0,98**	0,82	-0,29	-1,16	0,24	650	**0,81**	0,91	-0,14	-0,54	0,59	1231	**0,98**	0,95	3,00	-0,08	0,15
**PA0591**	13758	5,71	144,04	4	29,35	1,07	0,10	0,29	0,78	1706	**0,99**	0,93	-0,11	-0,51	0,61	1365	**0,96**	0,97	-0,05	-0,22	0,83	1696	**1,00**	0,99	3,00	-0,02	0,08
**PA0592**	31671	6,84	650,62	12	43,85	1,08	0,11	0,31	0,75	1651	**0,99**	1,04	0,06	0,14	0,89	1960	**0,98**	1,02	0,03	0,04	0,97	1989	**1,00**	1,05	3,00	0,07	0,03
**PA0593**	36298	6,16	182,48	5	19,14	0,79	-0,35	-1,30	0,19	565	**0,75**	1,29	0,36	1,26	0,21	603	**0,74**	0,91	-0,14	-0,54	0,59	1235	**0,98**	0,99	3,00	-0,01	0,26
**PA0594**	51241	5,65	2119,29	40	66,40	1,13	0,18	0,56	0,58	1274	**0,98**	1,11	0,16	0,49	0,63	1405	**0,96**	1,05	0,07	0,19	0,85	1746	**1,00**	1,10	3,00	0,14	0,04
**PA0595**	109959	5,41	1880,45	45	40,13	0,93	-0,10	-0,44	0,66	1452	**0,99**	0,88	-0,19	-0,81	0,42	956	**0,94**	0,90	-0,15	-0,57	0,57	1209	**0,97**	0,90	3,00	-0,15	0,03
**PA0596**	39734	5,27	719,25	20	42,53	1,02	0,03	0,04	0,97	2108	**1,00**	1,03	0,04	0,05	0,96	2101	**0,99**	1,09	0,13	0,38	0,70	1441	**1,01**	1,05	3,00	0,07	0,04
**PA0603**	42687	6,03	748,15	17	47,72	0,95	-0,07	-0,33	0,74	1625	**0,99**	0,88	-0,19	-0,81	0,42	957	**0,94**	0,94	-0,09	-0,37	0,71	1465	**1,00**	0,92	3,00	-0,12	0,04
**PA0604**	42892	5,87	373,06	10	26,00	0,41	-1,28	-4,57	0,00	81	**0,00**	1,29	0,37	1,27	0,20	594	**0,74**	1,41	0,50	1,62	0,10	408	**0,53**	1,04	3,00	0,05	0,54
**PA0605**	48051	9,53	240,84	7	19,59	1,08	0,11	0,32	0,75	1649	**0,99**	1,06	0,09	0,24	0,81	1791	**0,98**	1,02	0,03	0,05	0,96	1974	**1,00**	1,06	3,00	0,08	0,03
**PA0607**	25469	5,16	766,52	14	44,20	0,93	-0,10	-0,43	0,67	1474	**0,98**	1,12	0,16	0,51	0,61	1362	**0,97**	1,21	0,27	0,86	0,39	876	**0,92**	1,09	3,00	0,12	0,14
**PA0609**	56958	4,99	416,78	11	19,06	1,17	0,23	0,73	0,46	1035	**0,97**	1,11	0,15	0,46	0,64	1441	**0,97**	1,10	0,13	0,39	0,70	1428	**1,01**	1,13	3,00	0,17	0,04
**PA0622**	43628	5,27	463,01	9	31,23	0,62	-0,69	-2,49	0,01	206	**0,14**	0,52	-0,93	-3,56	0,00	139	**0,01**	0,72	-0,47	-1,66	0,10	393	**0,51**	0,62	3,00	-0,68	0,10
**PA0623**	19513	5,06	159,94	5	21,48	0,82	-0,28	-1,06	0,29	724	**0,87**	0,76	-0,40	-1,58	0,12	465	**0,54**	0,55	-0,87	-3,02	0,00	169	**0,03**	0,71	3,00	-0,49	0,15
**PA0634**	13629	5,07	76,61	2	20,00	0,61	-0,72	-2,61	0,01	188	**0,10**	0,93	-0,11	-0,49	0,62	1394	**0,96**	0,58	-0,80	-2,75	0,01	200	**0,06**	0,70	3,00	-0,51	0,20
**PA0649**	23323	5,69	173,08	4	21,40	1,03	0,05	0,08	0,94	2038	**1,00**	1,24	0,31	1,04	0,30	759	**0,85**	1,10	0,14	0,41	0,68	1394	**1,00**	1,12	3,00	0,17	0,10
**PA0650**	39804	5,51	439,66	10	27,94	0,96	-0,06	-0,30	0,76	1675	**0,99**	1,07	0,09	0,25	0,80	1762	**0,98**	0,89	-0,17	-0,65	0,52	1119	**0,96**	0,97	3,00	-0,04	0,09
**PA0651**	32307	4,93	386,82	11	33,17	0,97	-0,05	-0,25	0,80	1768	**0,99**	0,99	-0,02	-0,18	0,86	1892	**0,98**	0,97	-0,04	-0,18	0,86	1765	**1,00**	0,98	3,00	-0,04	0,01
**PA0652**	26601	8,25	552,43	14	46,14	0,65	-0,63	-2,28	0,02	236	**0,21**	0,58	-0,79	-3,06	0,00	174	**0,03**	0,64	-0,64	-2,22	0,03	268	**0,20**	0,62	3,00	-0,69	0,04
**PA0654**	32104	4,71	492,23	12	42,17	0,85	-0,24	-0,91	0,36	865	**0,91**	0,87	-0,20	-0,85	0,40	922	**0,93**	0,87	-0,21	-0,76	0,45	982	**0,94**	0,86	3,00	-0,21	0,01
**PA0655**	24776	5,39	132,14	5	34,96	0,96	-0,06	-0,31	0,76	1668	**0,99**	1,04	0,06	0,13	0,90	1981	**0,98**	0,90	-0,16	-0,59	0,55	1182	**0,96**	0,96	3,00	-0,05	0,07
**PA0659**	39311	4,52	119,31	2	6,10	0,69	-0,53	-1,94	0,05	292	**0,39**	0,74	-0,43	-1,69	0,09	423	**0,46**	0,65	-0,62	-2,17	0,03	275	**0,22**	0,69	3,00	-0,53	0,05
**PA0660**	36418	5,86	293,30	6	23,27	1,00	0,00	-0,07	0,94	2047	**1,00**	1,03	0,04	0,06	0,95	2071	**0,99**	1,03	0,04	0,08	0,93	1926	**1,00**	1,02	3,00	0,03	0,02
**PA0662**	38188	6,22	613,99	17	43,51	0,78	-0,35	-1,32	0,19	554	**0,74**	0,93	-0,10	-0,48	0,63	1426	**0,96**	0,88	-0,19	-0,69	0,49	1073	**0,95**	0,86	3,00	-0,21	0,08
**PA0663**	29487	9,69	39,57	2	4,60	0,81	-0,31	-1,16	0,25	655	**0,82**	0,83	-0,27	-1,10	0,27	713	**0,83**	0,85	-0,23	-0,83	0,41	910	**0,92**	0,83	3,00	-0,27	0,02
**PA0664**	15547	5,54	327,62	7	49,69	0,74	-0,43	-1,60	0,11	419	**0,57**	0,65	-0,61	-2,38	0,02	254	**0,15**	0,74	-0,44	-1,56	0,12	435	**0,56**	0,71	3,00	-0,49	0,05
**PA0665**	13335	4,20	211,52	4	25,00	1,14	0,19	0,60	0,55	1215	**0,98**	1,06	0,08	0,20	0,84	1859	**0,98**	1,18	0,24	0,75	0,45	998	**0,93**	1,13	3,00	0,17	0,06
**PA0666**	39869	5,28	76,02	2	7,95	1,23	0,30	0,96	0,34	809	**0,90**	1,17	0,23	0,76	0,44	1020	**0,94**	1,15	0,20	0,64	0,52	1133	**0,95**	1,19	3,00	0,24	0,04
**PA0668**	48137	5,61	1684,13	35	60,98	0,92	-0,12	-0,51	0,61	1338	**0,99**	0,85	-0,23	-0,97	0,33	810	**0,88**	0,87	-0,19	-0,72	0,47	1042	**0,94**	0,88	3,00	-0,18	0,03
**PA0672**	23093	5,03	683,69	16	65,19	5,65	2,50	8,69	0,00	7	**0,00**	3,62	1,85	6,81	0,00	46	**0,00**	8,27	3,05	10,27	0,00	18	**0,00**	5,85	3,00	2,55	2,33
**PA0705**	36204	8,31	167,50	6	21,25	1,10	0,14	0,42	0,67	1489	**0,98**	1,11	0,14	0,44	0,66	1478	**0,97**	1,05	0,07	0,16	0,87	1791	**1,00**	1,09	3,00	0,12	0,03
**PA0715**	41407	9,46	185,69	6	16,97	1,04	0,05	0,10	0,92	1995	**1,00**	1,06	0,08	0,20	0,84	1852	**0,98**	1,20	0,27	0,85	0,39	885	**0,92**	1,10	3,00	0,14	0,09
**PA0729**	13599	4,77	51,09	2	13,90	1,36	0,45	1,49	0,14	466	**0,64**	1,05	0,07	0,17	0,86	1902	**0,98**	0,96	-0,06	-0,27	0,79	1620	**1,00**	1,12	3,00	0,17	0,21
**PA0744**	41848	5,26	140,52	3	7,10	2,01	1,01	3,47	0,00	117	**0,01**	2,70	1,44	5,25	0,00	75	**0,00**	2,56	1,36	4,54	0,00	96	**0,00**	2,43	3,00	1,28	0,36
**PA0745**	31700	6,02	181,40	5	21,24	1,70	0,77	2,61	0,01	187	**0,10**	1,71	0,78	2,80	0,01	203	**0,05**	1,77	0,82	2,74	0,01	202	**0,06**	1,73	3,00	0,79	0,04
**PA0746**	44191	5,44	71,55	3	10,30	1,82	0,86	2,95	0,00	157	**0,04**	1,66	0,74	2,64	0,01	217	**0,08**	2,20	1,14	3,79	0,00	121	**0,00**	1,89	3,00	0,92	0,27
**PA0750**	28247	7,81	343,33	8	42,10	1,09	0,12	0,35	0,73	1599	**0,99**	0,95	-0,07	-0,38	0,71	1573	**0,97**	0,90	-0,15	-0,55	0,58	1222	**0,98**	0,98	3,00	-0,03	0,10
**PA0754**	37870	5,58	930,08	21	48,90	6,05	2,60	9,03	0,00	9	**0,00**	6,36	2,67	9,85	0,00	11	**0,00**	8,47	3,08	10,38	0,00	22	**0,00**	6,96	3,00	2,80	1,32
**PA0755**	49973	7,81	208,50	5	10,76	6,52	2,71	9,41	0,00	13	**0,00**	4,87	2,28	8,41	0,00	1	**0,00**	6,02	2,59	8,72	0,00	9	**0,00**	5,80	3,00	2,54	0,85
**PA0756**	25759	5,40	73,22	3	21,50	1,24	0,31	1,00	0,32	775	**0,89**	1,18	0,23	0,77	0,44	1014	**0,94**	1,45	0,53	1,75	0,08	362	**0,46**	1,29	3,00	0,36	0,14
**PA0758**	32389	4,95	478,56	12	34,90	0,65	-0,62	-2,27	0,02	239	**0,21**	0,68	-0,55	-2,14	0,03	297	**0,24**	0,62	-0,68	-2,38	0,02	242	**0,15**	0,65	3,00	-0,62	0,03
**PA0759**	35082	5,37	784,97	17	47,22	1,14	0,19	0,59	0,55	1221	**0,98**	1,20	0,27	0,89	0,37	871	**0,92**	1,09	0,12	0,36	0,72	1476	**1,00**	1,14	3,00	0,19	0,06
**PA0761**	61920	5,85	290,39	9	15,81	1,25	0,32	1,05	0,29	736	**0,87**	1,18	0,24	0,81	0,42	959	**0,94**	1,09	0,13	0,38	0,70	1447	**1,00**	1,18	3,00	0,23	0,08
**PA0762**	23381	5,44	639,01	15	61,75	1,14	0,19	0,57	0,57	1250	**0,99**	1,14	0,18	0,59	0,56	1235	**0,97**	1,16	0,21	0,66	0,51	1099	**0,95**	1,14	3,00	0,19	0,01
**PA0763**	21752	5,67	248,22	6	28,60	1,50	0,59	1,99	0,05	279	**0,37**	1,21	0,28	0,95	0,34	833	**0,89**	1,49	0,58	1,89	0,06	322	**0,37**	1,40	3,00	0,49	0,16
**PA0764**	35842	5,81	496,04	12	33,13	1,18	0,24	0,76	0,44	1009	**0,96**	1,19	0,25	0,83	0,40	934	**0,94**	1,27	0,34	1,10	0,27	665	**0,84**	1,21	3,00	0,28	0,05

**PA0766**	54227	7,04	1341,55	27	40,03	1,39	0,47	1,58	0,11	425	**0,59**	1,42	0,51	1,80	0,07	389	**0,40**	1,29	0,37	1,19	0,23	615	**0,78**	1,37	3,00	0,45	0,07
**PA0767**	72353	5,43	1026,39	25	33,12	1,29	0,36	1,20	0,23	630	**0,80**	1,22	0,29	0,98	0,33	806	**0,88**	1,18	0,24	0,77	0,44	973	**0,93**	1,23	3,00	0,30	0,05
**PA0768**	34579	8,49	412,68	12	35,31	1,23	0,30	0,97	0,33	802	**0,90**	1,10	0,14	0,41	0,68	1528	**0,97**	1,13	0,17	0,52	0,60	1249	**0,99**	1,15	3,00	0,20	0,07
**PA0769**	15922	7,96	115,31	3	30,40	0,79	-0,34	-1,28	0,20	578	**0,76**	0,72	-0,48	-1,89	0,06	358	**0,35**	0,76	-0,39	-1,39	0,16	525	**0,64**	0,76	3,00	-0,40	0,04
**PA0770**	26749	6,20	191,80	7	28,63	1,11	0,15	0,44	0,66	1449	**0,99**	0,95	-0,08	-0,38	0,70	1563	**0,97**	1,10	0,13	0,39	0,70	1426	**1,00**	1,05	3,00	0,07	0,09
**PA0771**	37790	6,18	426,92	12	40,73	1,12	0,16	0,50	0,62	1365	**0,99**	1,16	0,22	0,72	0,47	1062	**0,96**	1,33	0,41	1,33	0,18	546	**0,69**	1,20	3,00	0,27	0,11
**PA0773**	27978	5,52	90,97	2	9,50	0,42	-1,26	-4,51	0,00	83	**0,00**	0,81	-0,30	-1,22	0,22	622	**0,77**	0,70	-0,51	-1,79	0,07	351	**0,43**	0,64	3,00	-0,64	0,20
**PA0774**	38268	6,01	239,94	7	13,10	1,16	0,21	0,67	0,50	1116	**0,98**	1,23	0,30	1,01	0,31	790	**0,85**	1,05	0,07	0,19	0,85	1748	**1,00**	1,15	3,00	0,20	0,09
**PA0775**	28496	5,40	368,44	9	36,03	1,28	0,36	1,18	0,24	646	**0,80**	1,08	0,12	0,33	0,74	1635	**0,98**	1,06	0,08	0,22	0,83	1694	**1,00**	1,14	3,00	0,19	0,12
**PA0778**	15457	4,85	127,66	2	16,80	1,44	0,52	1,75	0,08	359	**0,49**	1,53	0,61	2,19	0,03	288	**0,21**	0,97	-0,05	-0,23	0,82	1683	**1,01**	1,31	3,00	0,39	0,30
**PA0779**	96636	6,38	1574,66	41	38,94	0,93	-0,10	-0,42	0,67	1484	**0,98**	1,06	0,09	0,23	0,81	1801	**0,98**	1,04	0,06	0,15	0,88	1811	**1,00**	1,01	3,00	0,02	0,07
**PA0782**	121541	5,83	4550,04	99	62,52	3,32	1,73	5,99	0,00	52	**0,00**	3,23	1,69	6,20	0,00	59	**0,00**	3,82	1,93	6,49	0,00	51	**0,00**	3,45	3,00	1,79	0,32
**PA0783**	56148	7,66	416,38	10	13,59	3,16	1,66	5,74	0,00	58	**0,00**	2,70	1,43	5,25	0,00	76	**0,00**	3,13	1,65	5,53	0,00	71	**0,00**	3,00	3,00	1,58	0,26
**PA0789**	53378	9,44	126,96	5	6,62	1,43	0,51	1,73	0,08	365	**0,50**	1,63	0,70	2,52	0,01	226	**0,11**	2,40	1,26	4,22	0,00	104	**0,00**	1,82	3,00	0,86	0,51
**PA0794**	99090	5,41	1048,12	27	31,99	1,29	0,37	1,22	0,22	618	**0,78**	1,55	0,63	2,26	0,02	275	**0,19**	1,50	0,58	1,92	0,05	315	**0,36**	1,45	3,00	0,53	0,14
**PA0795**	44873	6,03	942,04	20	46,74	1,92	0,94	3,21	0,00	133	**0,02**	2,16	1,11	4,05	0,00	113	**0,00**	1,95	0,96	3,21	0,00	154	**0,02**	2,01	3,00	1,01	0,13
**PA0796**	34463	5,33	429,95	13	33,08	1,07	0,10	0,27	0,79	1734	**0,98**	1,22	0,29	0,98	0,33	807	**0,88**	1,43	0,52	1,69	0,09	379	**0,50**	1,24	3,00	0,31	0,18
**PA0799**	78090	5,93	64,99	2	4,00	0,88	-0,19	-0,75	0,45	1023	**0,96**	1,50	0,59	2,10	0,04	306	**0,25**	1,00	0,00	-0,07	0,94	1939	**1,00**	1,13	3,00	0,17	0,33
**PA0833**	25799	8,89	1090,73	17	57,24	1,17	0,22	0,71	0,48	1073	**0,97**	1,25	0,32	1,11	0,27	707	**0,82**	0,97	-0,04	-0,20	0,84	1725	**1,00**	1,13	3,00	0,18	0,14
**PA0834**	38242	9,53	120,29	3	12,20	1,24	0,31	0,99	0,32	781	**0,89**	1,30	0,37	1,29	0,20	576	**0,73**	1,92	0,94	3,13	0,00	160	**0,02**	1,48	3,00	0,57	0,38
**PA0835**	79234	5,20	437,31	13	17,38	1,08	0,11	0,31	0,76	1673	**0,99**	0,95	-0,07	-0,35	0,73	1614	**0,97**	0,79	-0,34	-1,20	0,23	611	**0,77**	0,94	3,00	-0,09	0,14
**PA0836**	43633	6,59	369,30	10	32,26	0,85	-0,24	-0,93	0,35	834	**0,91**	1,08	0,11	0,33	0,74	1640	**0,98**	0,93	-0,11	-0,42	0,68	1388	**1,00**	0,95	3,00	-0,07	0,12
**PA0837**	17864	4,47	233,05	6	28,60	1,20	0,26	0,84	0,40	930	**0,93**	1,12	0,16	0,49	0,63	1404	**0,96**	1,19	0,25	0,80	0,43	937	**0,94**	1,17	3,00	0,22	0,05
**PA0838**	18399	5,12	285,22	5	27,50	0,94	-0,09	-0,39	0,69	1528	**0,99**	0,99	-0,01	-0,15	0,88	1950	**0,98**	1,05	0,07	0,16	0,87	1792	**1,00**	0,99	3,00	-0,01	0,05
**PA0840**	42372	5,52	603,80	14	38,09	1,11	0,15	0,46	0,64	1421	**0,98**	1,00	0,00	-0,12	0,91	1995	**0,98**	0,96	-0,05	-0,23	0,82	1674	**1,00**	1,02	3,00	0,04	0,08
**PA0848**	23189	5,23	359,90	8	43,45	2,35	1,23	4,25	0,00	90	**0,00**	1,91	0,94	3,39	0,00	152	**0,01**	1,93	0,95	3,16	0,00	156	**0,02**	2,06	3,00	1,05	0,25
**PA0849**	35085	5,40	425,66	6	23,40	1,92	0,94	3,22	0,00	132	**0,02**	1,69	0,76	2,72	0,01	209	**0,07**	1,36	0,44	1,45	0,15	493	**0,62**	1,66	3,00	0,73	0,28
**PA0852**	44996	6,38	349,25	8	20,05	0,92	-0,12	-0,50	0,62	1364	**0,99**	0,71	-0,49	-1,93	0,05	348	**0,33**	0,82	-0,29	-1,05	0,30	714	**0,85**	0,82	3,00	-0,29	0,10
**PA0854**	51725	5,74	324,24	8	22,61	0,88	-0,18	-0,71	0,48	1077	**0,97**	0,97	-0,05	-0,27	0,78	1733	**0,98**	0,91	-0,13	-0,49	0,62	1284	**1,00**	0,92	3,00	-0,12	0,04
**PA0856**	22868	9,44	735,36	17	49,59	1,20	0,27	0,85	0,39	924	**0,93**	1,17	0,23	0,76	0,45	1025	**0,94**	1,39	0,47	1,55	0,12	438	**0,57**	1,25	3,00	0,33	0,12
**PA0857**	11938	6,69	175,30	4	34,40	1,49	0,58	1,95	0,05	290	**0,39**	1,09	0,13	0,37	0,71	1585	**0,97**	1,56	0,65	2,13	0,03	282	**0,24**	1,38	3,00	0,47	0,26
**PA0858**	38504	5,57	630,86	17	47,65	0,93	-0,11	-0,45	0,65	1433	**0,99**	1,07	0,09	0,24	0,81	1788	**0,98**	1,07	0,09	0,26	0,80	1639	**1,00**	1,02	3,00	0,03	0,08
**PA0865**	42286	5,10	2217,54	42	66,06	5,54	2,47	8,59	0,00	5	**0,00**	5,23	2,39	8,80	0,00	5	**0,00**	6,51	2,70	9,10	0,00	13	**0,00**	5,76	3,00	2,53	0,66
**PA0867**	15554	5,06	297,58	6	42,95	1,13	0,18	0,56	0,58	1279	**0,98**	1,19	0,26	0,86	0,39	909	**0,93**	1,20	0,26	0,83	0,41	912	**0,92**	1,18	3,00	0,23	0,04
**PA0869**	36057	10,61	582,77	12	35,95	1,87	0,91	3,10	0,00	144	**0,03**	1,87	0,90	3,25	0,00	158	**0,02**	2,06	1,04	3,48	0,00	138	**0,01**	1,93	3,00	0,95	0,11
**PA0870**	44963	5,86	2117,51	43	75,26	3,53	1,82	6,30	0,00	44	**0,00**	3,80	1,93	7,08	0,00	41	**0,00**	4,67	2,22	7,48	0,00	43	**0,00**	4,00	3,00	2,00	0,60
**PA0871**	14570	5,94	717,77	16	72,90	3,91	1,97	6,82	0,00	38	**0,00**	5,16	2,37	8,72	0,00	3	**0,00**	4,38	2,13	7,16	0,00	46	**0,00**	4,48	3,00	2,16	0,63
**PA0872**	31836	5,49	615,75	13	47,68	3,54	1,83	6,33	0,00	42	**0,00**	3,93	1,97	7,25	0,00	39	**0,00**	2,91	1,54	5,17	0,00	79	**0,00**	3,46	3,00	1,79	0,51
**PA0873**	59751	6,42	200,31	7	9,91	1,30	0,38	1,26	0,21	587	**0,77**	1,26	0,33	1,13	0,26	687	**0,82**	1,28	0,35	1,14	0,25	642	**0,81**	1,28	3,00	0,35	0,02
**PA0876**	35810	5,87	111,09	3	10,00	0,97	-0,05	-0,25	0,80	1765	**0,99**	1,02	0,03	0,01	0,99	2145	**1,00**	1,04	0,06	0,14	0,89	1830	**1,00**	1,01	3,00	0,01	0,04
**PA0887**	76742	5,91	852,79	19	22,45	0,35	-1,51	-5,37	0,00	69	**0,00**	0,39	-1,38	-5,22	0,00	78	**0,00**	0,33	-1,62	-5,55	0,00	70	**0,00**	0,35	3,00	-1,50	0,03
**PA0888**	31543	6,43	1941,11	36	78,66	0,99	-0,01	-0,13	0,89	1961	**0,99**	1,03	0,05	0,07	0,94	2059	**0,99**	1,05	0,07	0,19	0,85	1754	**1,00**	1,02	3,00	0,03	0,03
**PA0889**	25993	8,99	172,13	5	23,64	0,86	-0,21	-0,82	0,41	956	**0,94**	0,84	-0,26	-1,06	0,29	743	**0,84**	0,86	-0,21	-0,77	0,44	976	**0,93**	0,85	3,00	-0,23	0,02
**PA0890**	27388	9,49	52,68	3	6,00	0,81	-0,30	-1,14	0,25	672	**0,82**	0,80	-0,33	-1,32	0,19	563	**0,72**	0,79	-0,34	-1,19	0,23	617	**0,78**	0,80	3,00	-0,32	0,01
**PA0891**	41277	5,17	466,88	9	30,81	0,76	-0,41	-1,50	0,13	457	**0,63**	0,84	-0,26	-1,06	0,29	742	**0,84**	1,05	0,07	0,19	0,85	1747	**1,00**	0,88	3,00	-0,18	0,15
**PA0892**	30769	7,22	698,81	15	52,38	0,90	-0,16	-0,63	0,53	1166	**0,99**	0,95	-0,07	-0,36	0,72	1596	**0,97**	0,88	-0,18	-0,68	0,50	1084	**0,95**	0,91	3,00	-0,14	0,04
**PA0893**	38024	8,37	165,89	4	14,60	0,86	-0,22	-0,86	0,39	917	**0,93**	0,94	-0,09	-0,42	0,67	1510	**0,97**	1,03	0,04	0,09	0,92	1904	**1,00**	0,94	3,00	-0,08	0,09
**PA0895**	46694	5,63	2940,18	59	80,72	1,20	0,26	0,85	0,40	927	**0,93**	1,31	0,39	1,35	0,18	549	**0,70**	1,26	0,34	1,08	0,28	687	**0,84**	1,26	3,00	0,33	0,05

**PA0896**	38152	4,90	571,64	11	26,90	1,00	0,00	-0,09	0,93	2016	**1,00**	1,12	0,17	0,53	0,60	1334	**0,97**	1,04	0,06	0,13	0,89	1837	**1,00**	1,05	3,00	0,08	0,06
**PA0897**	39234	6,11	1080,83	25	50,30	1,10	0,14	0,42	0,67	1488	**0,98**	1,11	0,16	0,49	0,63	1408	**0,96**	1,19	0,25	0,79	0,43	944	**0,94**	1,14	3,00	0,18	0,05
**PA0898**	53582	5,86	1073,97	26	47,02	1,20	0,27	0,86	0,39	918	**0,93**	1,08	0,11	0,29	0,77	1691	**0,98**	1,00	0,01	-0,03	0,97	2001	**1,00**	1,09	3,00	0,13	0,10
**PA0899**	51518	5,85	1211,83	29	47,91	1,10	0,14	0,41	0,68	1500	**0,99**	1,14	0,19	0,61	0,54	1213	**0,97**	1,24	0,31	0,99	0,32	769	**0,87**	1,16	3,00	0,21	0,07
**PA0901**	38760	5,76	434,27	13	36,85	1,20	0,26	0,83	0,41	947	**0,94**	1,24	0,31	1,07	0,29	735	**0,84**	1,37	0,45	1,47	0,14	479	**0,60**	1,27	3,00	0,34	0,09
**PA0902**	36843	6,01	410,44	11	28,08	0,90	-0,15	-0,61	0,54	1190	**0,99**	0,99	-0,02	-0,15	0,88	1936	**0,98**	0,91	-0,13	-0,50	0,62	1280	**1,00**	0,93	3,00	-0,10	0,05
**PA0903**	101928	5,30	2764,58	58	56,30	1,14	0,18	0,57	0,57	1258	**0,98**	1,19	0,25	0,83	0,41	946	**0,93**	1,16	0,22	0,67	0,50	1090	**0,95**	1,16	3,00	0,22	0,03
**PA0904**	47145	5,21	1856,19	38	56,92	1,07	0,10	0,27	0,78	1728	**0,99**	1,04	0,06	0,14	0,89	1964	**0,98**	1,15	0,20	0,62	0,53	1143	**0,96**	1,09	3,00	0,12	0,05
**PA0913**	56237	4,71	368,42	9	23,17	0,71	-0,50	-1,84	0,07	319	**0,44**	0,65	-0,63	-2,44	0,01	247	**0,13**	0,89	-0,17	-0,62	0,54	1151	**0,96**	0,75	3,00	-0,42	0,13
**PA0916**	53090	4,87	584,08	15	33,27	1,03	0,05	0,09	0,93	2018	**1,00**	0,91	-0,13	-0,58	0,56	1250	**0,97**	0,97	-0,04	-0,21	0,83	1710	**1,01**	0,97	3,00	-0,04	0,06
**PA0919**	49835	5,17	57,24	3	9,40	1,35	0,43	1,43	0,15	499	**0,67**	1,13	0,17	0,55	0,58	1302	**0,96**	1,26	0,33	1,07	0,28	690	**0,85**	1,24	3,00	0,32	0,11
**PA0926**	17202	8,67	198,87	6	38,30	1,34	0,42	1,40	0,16	509	**0,69**	1,11	0,15	0,48	0,63	1424	**0,96**	0,89	-0,17	-0,64	0,52	1127	**0,95**	1,11	3,00	0,16	0,23
**PA0928**	105270	4,96	475,16	11	11,89	1,02	0,03	0,01	0,99	2163	**1,00**	1,07	0,10	0,26	0,79	1752	**0,98**	1,25	0,32	1,03	0,30	729	**0,86**	1,11	3,00	0,15	0,12
**PA0931**	84409	5,47	140,57	4	5,70	1,88	0,91	3,11	0,00	142	**0,03**	2,65	1,41	5,15	0,00	82	**0,00**	1,36	0,45	1,46	0,14	489	**0,61**	1,96	3,00	0,97	0,65
**PA0932**	34150	5,54	706,82	18	49,13	1,23	0,30	0,97	0,33	797	**0,90**	1,32	0,41	1,41	0,16	523	**0,65**	1,23	0,30	0,94	0,35	805	**0,88**	1,26	3,00	0,33	0,06
**PA0933**	50659	7,68	215,67	7	10,81	1,37	0,45	1,50	0,13	458	**0,63**	1,04	0,06	0,12	0,91	1994	**0,98**	1,46	0,54	1,79	0,07	353	**0,43**	1,29	3,00	0,37	0,22
**PA0934**	88887	7,14	916,84	25	28,98	1,12	0,16	0,49	0,62	1373	**0,99**	1,20	0,27	0,89	0,37	870	**0,92**	1,17	0,23	0,71	0,47	1046	**0,94**	1,16	3,00	0,22	0,04
**PA0935**	33675	4,82	220,53	7	24,34	0,89	-0,16	-0,66	0,51	1130	**0,98**	1,02	0,03	0,03	0,97	2115	**1,00**	1,22	0,29	0,91	0,36	826	**0,90**	1,05	3,00	0,06	0,16
**PA0936**	37748	9,90	165,94	5	14,26	1,52	0,61	2,05	0,04	272	**0,32**	1,44	0,53	1,87	0,06	364	**0,36**	1,09	0,13	0,38	0,70	1442	**1,00**	1,35	3,00	0,44	0,23
**PA0937**	24091	9,08	290,95	6	28,50	1,15	0,20	0,61	0,54	1186	**0,99**	1,19	0,25	0,82	0,41	950	**0,94**	1,06	0,09	0,23	0,82	1673	**1,00**	1,13	3,00	0,18	0,06
**PA0938**	52839	9,00	927,32	22	36,81	1,80	0,85	2,89	0,00	161	**0,05**	1,66	0,74	2,64	0,01	216	**0,08**	1,61	0,68	2,26	0,02	263	**0,19**	1,69	3,00	0,76	0,10
**PA0942**	21606	6,95	143,50	5	30,10	1,13	0,17	0,53	0,59	1304	**0,99**	0,94	-0,09	-0,45	0,65	1462	**0,97**	0,70	-0,52	-1,81	0,07	346	**0,42**	0,92	3,00	-0,12	0,22
**PA0943**	30192	7,77	153,13	4	24,80	1,33	0,41	1,37	0,17	522	**0,71**	1,08	0,11	0,33	0,74	1649	**0,98**	1,43	0,51	1,68	0,09	386	**0,50**	1,28	3,00	0,36	0,18
**PA0944**	25531	6,67	230,40	7	27,60	1,33	0,41	1,36	0,17	529	**0,71**	1,18	0,24	0,81	0,42	967	**0,94**	1,26	0,34	1,08	0,28	682	**0,84**	1,26	3,00	0,33	0,07
**PA0945**	39963	4,78	933,12	20	49,13	1,26	0,34	1,11	0,27	691	**0,84**	1,28	0,35	1,21	0,22	623	**0,78**	1,33	0,41	1,33	0,18	545	**0,69**	1,29	3,00	0,37	0,03
**PA0946**	37665	4,93	618,35	9	21,70	1,00	0,00	-0,07	0,94	2046	**1,00**	0,99	-0,01	-0,13	0,89	1972	**0,98**	0,91	-0,14	-0,54	0,59	1234	**0,99**	0,97	3,00	-0,05	0,05
**PA0947**	28307	5,80	225,22	5	28,10	1,28	0,35	1,16	0,25	658	**0,82**	0,98	-0,03	-0,22	0,83	1825	**0,98**	1,11	0,15	0,44	0,66	1359	**1,00**	1,12	3,00	0,16	0,15
**PA0949**	21878	6,32	102,05	4	25,65	1,37	0,46	1,53	0,13	450	**0,61**	1,09	0,13	0,38	0,71	1571	**0,97**	1,24	0,30	0,98	0,33	779	**0,87**	1,23	3,00	0,30	0,14
**PA0950**	13837	6,14	341,71	9	71,80	1,40	0,49	1,62	0,10	409	**0,55**	1,42	0,51	1,79	0,07	392	**0,41**	1,34	0,42	1,37	0,17	534	**0,65**	1,39	3,00	0,47	0,04
**PA0953**	17743	5,05	140,46	3	25,77	1,08	0,12	0,32	0,75	1636	**0,99**	1,07	0,10	0,28	0,78	1716	**0,98**	1,04	0,06	0,13	0,90	1845	**1,00**	1,06	3,00	0,09	0,02
**PA0955**	37023	5,16	326,15	8	19,50	1,39	0,47	1,58	0,11	423	**0,58**	1,31	0,38	1,34	0,18	555	**0,71**	1,27	0,35	1,12	0,26	654	**0,83**	1,32	3,00	0,40	0,06
**PA0956**	67093	5,28	2597,99	53	57,58	1,38	0,46	1,54	0,12	445	**0,61**	1,35	0,43	1,51	0,13	496	**0,57**	1,33	0,42	1,35	0,18	541	**0,67**	1,35	3,00	0,44	0,02
**PA0958**	51789	4,96	1332,33	32	48,99	0,96	-0,06	-0,28	0,78	1720	**0,99**	1,13	0,17	0,54	0,59	1313	**0,97**	1,22	0,29	0,93	0,35	816	**0,89**	1,10	3,00	0,14	0,13
**PA0959**	23867	6,10	398,68	12	32,55	1,22	0,29	0,93	0,35	836	**0,91**	1,11	0,15	0,46	0,65	1446	**0,97**	1,38	0,47	1,53	0,13	450	**0,58**	1,24	3,00	0,31	0,14
**PA0961**	7821	9,68	217,33	7	82,50	1,04	0,05	0,10	0,92	1990	**1,00**	0,98	-0,03	-0,21	0,83	1832	**0,98**	0,95	-0,08	-0,32	0,75	1554	**1,00**	0,99	3,00	-0,02	0,05
**PA0962**	18202	4,96	729,13	17	62,20	0,99	-0,02	-0,14	0,89	1955	**0,99**	1,00	0,00	-0,10	0,92	2012	**0,99**	0,97	-0,04	-0,19	0,85	1759	**1,00**	0,99	3,00	-0,02	0,01
**PA0963**	70719	5,37	2513,62	57	57,18	1,40	0,49	1,63	0,10	405	**0,55**	1,41	0,49	1,74	0,08	403	**0,44**	1,36	0,45	1,45	0,15	492	**0,61**	1,39	3,00	0,48	0,03
**PA0964**	28604	4,59	235,12	5	21,94	1,19	0,26	0,82	0,41	958	**0,94**	1,39	0,47	1,66	0,10	433	**0,49**	1,27	0,35	1,12	0,26	652	**0,83**	1,28	3,00	0,36	0,10
**PA0966**	23545	5,89	241,95	7	37,16	1,15	0,21	0,64	0,52	1146	**0,99**	0,98	-0,03	-0,20	0,84	1863	**0,98**	1,14	0,19	0,58	0,56	1201	**0,96**	1,09	3,00	0,13	0,10
**PA0967**	40206	5,33	572,84	13	36,50	1,17	0,23	0,72	0,47	1049	**0,97**	1,16	0,21	0,69	0,49	1106	**0,96**	1,15	0,20	0,62	0,53	1148	**0,96**	1,16	3,00	0,21	0,01
**PA0968**	17618	8,61	130,09	3	25,00	1,00	0,01	-0,06	0,95	2070	**1,00**	1,28	0,36	1,23	0,22	621	**0,76**	1,24	0,31	0,99	0,32	770	**0,87**	1,17	3,00	0,23	0,15
**PA0969**	26243	5,97	674,98	15	32,02	2,01	1,01	3,46	0,00	119	**0,01**	2,13	1,09	3,98	0,00	119	**0,00**	1,97	0,98	3,26	0,00	152	**0,02**	2,04	3,00	1,03	0,09
**PA0970**	17219	5,78	443,47	6	51,13	1,72	0,78	2,67	0,01	180	**0,09**	1,09	0,12	0,37	0,71	1592	**0,97**	1,80	0,85	2,82	0,00	188	**0,05**	1,54	3,00	0,62	0,39
**PA0971**	45099	9,55	178,96	7	14,90	1,18	0,24	0,77	0,44	1002	**0,95**	1,53	0,62	2,20	0,03	285	**0,21**	1,63	0,71	2,34	0,02	251	**0,16**	1,45	3,00	0,54	0,24
**PA0972**	49276	8,92	1984,62	43	65,26	1,55	0,63	2,15	0,03	257	**0,27**	1,43	0,51	1,82	0,07	387	**0,39**	1,38	0,46	1,51	0,13	457	**0,59**	1,45	3,00	0,54	0,09
**PA0973**	19545	5,95	1109,99	16	69,05	1,21	0,27	0,86	0,39	911	**0,92**	1,32	0,40	1,40	0,16	528	**0,66**	1,39	0,48	1,56	0,12	437	**0,56**	1,31	3,00	0,39	0,09
**PA0974**	31241	7,79	803,73	17	42,00	1,37	0,46	1,53	0,13	451	**0,61**	1,38	0,47	1,64	0,10	440	**0,50**	1,44	0,52	1,72	0,09	371	**0,48**	1,40	3,00	0,48	0,03
**PA0976**	25314	4,99	180,05	6	16,10	0,95	-0,07	-0,32	0,75	1644	**0,99**	1,03	0,04	0,05	0,96	2091	**0,99**	1,06	0,08	0,21	0,83	1703	**1,01**	1,01	3,00	0,02	0,05

**PA1004**	40962	5,41	388,84	10	28,25	1,09	0,13	0,38	0,71	1559	**0,98**	1,19	0,25	0,83	0,41	945	**0,94**	1,17	0,22	0,69	0,49	1069	**0,94**	1,15	3,00	0,20	0,05
**PA1005**	55457	7,81	152,43	7	18,20	1,10	0,14	0,41	0,69	1511	**0,99**	1,01	0,01	-0,07	0,95	2065	**0,99**	1,13	0,17	0,53	0,59	1240	**0,99**	1,08	3,00	0,11	0,06
**PA1008**	19784	6,73	511,01	15	63,07	1,20	0,26	0,84	0,40	935	**0,93**	1,35	0,44	1,53	0,13	489	**0,56**	1,26	0,33	1,06	0,29	705	**0,85**	1,27	3,00	0,34	0,08
**PA1009**	21091	5,45	170,24	6	13,33	1,06	0,09	0,23	0,82	1802	**0,99**	1,23	0,30	1,02	0,31	779	**0,86**	1,29	0,37	1,18	0,24	623	**0,78**	1,19	3,00	0,26	0,12
**PA1010**	33343	6,00	1300,65	26	62,74	0,89	-0,17	-0,69	0,49	1090	**0,97**	1,16	0,22	0,72	0,47	1064	**0,96**	1,14	0,19	0,58	0,56	1194	**0,96**	1,06	3,00	0,09	0,15
**PA1011**	45237	4,92	1720,72	29	71,96	1,24	0,31	1,02	0,31	760	**0,88**	1,23	0,30	1,02	0,31	781	**0,86**	1,22	0,28	0,90	0,37	833	**0,91**	1,23	3,00	0,30	0,01
**PA1013**	29978	5,31	977,23	22	65,79	1,16	0,22	0,68	0,50	1101	**0,98**	1,19	0,26	0,86	0,39	912	**0,93**	1,21	0,28	0,89	0,37	846	**0,91**	1,19	3,00	0,25	0,03
**PA1014**	38873	8,47	159,25	5	16,92	1,00	0,00	-0,07	0,95	2060	**1,00**	0,93	-0,11	-0,51	0,61	1356	**0,97**	0,84	-0,26	-0,94	0,35	811	**0,89**	0,92	3,00	-0,12	0,08
**PA1015**	29891	6,46	256,37	7	22,56	0,88	-0,19	-0,75	0,45	1024	**0,96**	0,91	-0,13	-0,58	0,56	1251	**0,97**	0,92	-0,12	-0,47	0,64	1305	**1,00**	0,90	3,00	-0,15	0,02
**PA1023**	34045	6,25	182,28	5	14,78	1,24	0,31	1,01	0,31	771	**0,89**	1,15	0,20	0,64	0,53	1185	**0,96**	1,14	0,19	0,59	0,56	1188	**0,96**	1,18	3,00	0,23	0,06
**PA1029**	11132	9,30	58,35	2	25,30	0,89	-0,17	-0,68	0,50	1106	**0,98**	0,92	-0,13	-0,57	0,57	1276	**0,96**	1,05	0,07	0,20	0,85	1743	**1,00**	0,95	3,00	-0,07	0,09
**PA1031**	54229	5,48	96,44	3	8,07	1,14	0,18	0,56	0,57	1266	**0,98**	1,07	0,10	0,28	0,78	1717	**0,98**	0,96	-0,06	-0,25	0,80	1647	**1,00**	1,06	3,00	0,08	0,09
**PA1032**	98617	7,25	442,12	12	16,23	1,27	0,34	1,12	0,26	678	**0,84**	1,22	0,29	0,98	0,33	805	**0,88**	1,25	0,32	1,04	0,30	720	**0,86**	1,25	3,00	0,32	0,02
**PA1033**	25808	6,30	41,84	2	7,00	0,97	-0,04	-0,23	0,81	1786	**0,99**	1,11	0,15	0,45	0,65	1453	**0,97**	0,82	-0,29	-1,02	0,31	736	**0,86**	0,97	3,00	-0,05	0,14
**PA1036**	20534	4,93	133,78	4	24,03	0,93	-0,11	-0,47	0,64	1407	**0,99**	0,82	-0,29	-1,17	0,24	642	**0,81**	0,58	-0,78	-2,71	0,01	204	**0,07**	0,78	3,00	-0,37	0,18
**PA1040**	19522	5,68	58,93	2	10,30	1,34	0,42	1,39	0,16	517	**0,69**	1,23	0,29	1,00	0,32	796	**0,86**	1,03	0,04	0,09	0,92	1906	**1,00**	1,20	3,00	0,26	0,15
**PA1043**	28648	7,77	355,70	9	27,81	1,21	0,28	0,90	0,37	871	**0,91**	0,94	-0,09	-0,44	0,66	1472	**0,97**	1,00	0,00	-0,05	0,96	1976	**1,00**	1,05	3,00	0,07	0,15
**PA1045**	83932	5,71	212,29	8	15,63	1,13	0,18	0,56	0,58	1275	**0,98**	1,09	0,12	0,36	0,72	1609	**0,97**	1,16	0,21	0,66	0,51	1105	**0,95**	1,13	3,00	0,17	0,04
**PA1047**	43835	5,79	282,61	7	16,61	1,02	0,03	0,03	0,98	2136	**1,00**	1,09	0,13	0,38	0,70	1558	**0,97**	1,04	0,06	0,14	0,89	1825	**1,00**	1,05	3,00	0,07	0,04
**PA1048**	35185	9,32	204,14	8	18,45	1,06	0,08	0,21	0,83	1829	**0,99**	1,19	0,25	0,83	0,41	940	**0,94**	1,15	0,20	0,62	0,53	1149	**0,96**	1,13	3,00	0,18	0,07
**PA1049**	25657	6,35	362,27	11	39,45	1,08	0,11	0,31	0,76	1659	**0,99**	1,12	0,16	0,51	0,61	1373	**0,96**	0,93	-0,11	-0,43	0,66	1365	**1,00**	1,04	3,00	0,06	0,10
**PA1052**	39744	5,14	199,51	7	15,34	1,14	0,19	0,59	0,56	1231	**0,98**	1,04	0,06	0,11	0,91	2004	**0,99**	1,59	0,67	2,22	0,03	271	**0,20**	1,26	3,00	0,33	0,29
**PA1053**	16406	9,64	626,91	12	48,93	1,08	0,11	0,32	0,75	1647	**0,99**	1,21	0,28	0,94	0,35	835	**0,90**	1,20	0,27	0,84	0,40	894	**0,92**	1,17	3,00	0,22	0,07
**PA1061**	41212	6,16	761,57	16	39,62	1,35	0,43	1,44	0,15	491	**0,66**	1,36	0,44	1,54	0,12	486	**0,55**	1,24	0,31	0,99	0,32	767	**0,87**	1,32	3,00	0,40	0,07
**PA1064**	25828	5,38	225,63	7	30,44	0,97	-0,05	-0,24	0,81	1777	**0,99**	1,00	0,00	-0,09	0,93	2024	**0,99**	0,97	-0,04	-0,21	0,83	1711	**1,00**	0,98	3,00	-0,03	0,02
**PA1068**	72045	5,57	768,65	18	29,90	0,98	-0,03	-0,18	0,86	1882	**0,99**	0,95	-0,08	-0,38	0,70	1560	**0,97**	1,05	0,06	0,16	0,87	1796	**1,00**	0,99	3,00	-0,01	0,05
**PA1069**	88971	4,62	1002,07	23	33,07	0,94	-0,09	-0,38	0,70	1550	**0,98**	0,94	-0,09	-0,41	0,68	1512	**0,97**	0,97	-0,04	-0,20	0,85	1734	**1,00**	0,95	3,00	-0,07	0,02
**PA1070**	27639	6,20	258,93	7	30,91	1,72	0,78	2,67	0,01	181	**0,09**	1,67	0,74	2,66	0,01	214	**0,08**	1,81	0,85	2,84	0,00	183	**0,05**	1,73	3,00	0,79	0,07
**PA1071**	30231	7,83	257,25	7	27,54	1,64	0,72	2,43	0,01	213	**0,15**	2,42	1,28	4,66	0,00	94	**0,00**	2,25	1,17	3,90	0,00	116	**0,00**	2,11	3,00	1,07	0,41
**PA1074**	44448	5,60	2116,44	37	62,54	2,44	1,29	4,44	0,00	85	**0,00**	2,72	1,44	5,28	0,00	74	**0,00**	3,13	1,65	5,52	0,00	72	**0,00**	2,76	3,00	1,47	0,35
**PA1076**	14599	5,62	80,70	2	55,00	1,30	0,38	1,25	0,21	592	**0,77**	0,91	-0,14	-0,60	0,55	1217	**0,97**	1,10	0,14	0,40	0,69	1407	**1,01**	1,10	3,00	0,14	0,20
**PA1080**	50324	4,63	270,02	5	10,34	0,92	-0,13	-0,53	0,60	1315	**0,99**	0,86	-0,22	-0,91	0,36	853	**0,92**	1,35	0,43	1,41	0,16	515	**0,64**	1,04	3,00	0,06	0,27
**PA1083**	26104	6,29	155,83	4	19,05	1,30	0,38	1,26	0,21	588	**0,77**	1,05	0,07	0,15	0,88	1933	**0,98**	1,06	0,08	0,21	0,83	1705	**1,01**	1,14	3,00	0,18	0,14
**PA1084**	40463	6,92	154,50	3	11,87	1,06	0,08	0,20	0,84	1854	**0,99**	1,10	0,14	0,42	0,67	1504	**0,97**	1,08	0,12	0,34	0,73	1514	**1,00**	1,08	3,00	0,11	0,02
**PA1086**	74428	4,64	32,80	2	2,55	1,21	0,28	0,90	0,37	877	**0,91**	1,04	0,05	0,10	0,92	2011	**0,99**	0,95	-0,07	-0,31	0,76	1567	**1,00**	1,07	3,00	0,09	0,13
**PA1087**	49266	4,57	204,94	4	10,53	1,08	0,12	0,33	0,74	1629	**0,99**	1,26	0,34	1,16	0,24	651	**0,81**	0,98	-0,03	-0,17	0,87	1785	**1,00**	1,11	3,00	0,15	0,14
**PA1091**	190830	5,31	359,74	10	6,12	1,11	0,15	0,46	0,65	1431	**0,98**	1,07	0,09	0,26	0,80	1759	**0,98**	1,26	0,34	1,08	0,28	683	**0,84**	1,15	3,00	0,20	0,10
**PA1092**	51663	5,40	2728,55	46	58,20	1,11	0,15	0,45	0,66	1445	**0,99**	1,02	0,03	0,03	0,98	2127	**0,99**	1,18	0,24	0,74	0,46	1008	**0,93**	1,10	3,00	0,14	0,08
**PA1093**	13890	4,60	134,91	3	31,70	1,29	0,37	1,23	0,22	612	**0,78**	1,47	0,56	1,98	0,05	332	**0,31**	1,24	0,31	1,00	0,32	751	**0,86**	1,34	3,00	0,42	0,12
**PA1094**	55184	6,52	920,84	23	44,10	1,08	0,11	0,31	0,76	1669	**0,99**	1,25	0,32	1,10	0,27	711	**0,83**	1,34	0,42	1,37	0,17	532	**0,66**	1,22	3,00	0,29	0,13
**PA1095**	14696	5,01	221,97	7	53,39	1,28	0,35	1,16	0,25	654	**0,82**	1,20	0,26	0,87	0,38	886	**0,93**	1,14	0,19	0,60	0,55	1174	**0,96**	1,21	3,00	0,27	0,07
**PA1097**	58020	5,26	771,31	20	39,58	0,94	-0,10	-0,42	0,68	1493	**0,99**	1,05	0,07	0,15	0,88	1942	**0,98**	1,05	0,07	0,17	0,87	1786	**1,00**	1,01	3,00	0,01	0,06
**PA1099**	53213	6,00	199,65	5	14,40	1,09	0,12	0,35	0,73	1597	**0,99**	0,97	-0,05	-0,29	0,77	1706	**0,98**	0,87	-0,20	-0,73	0,46	1025	**0,93**	0,98	3,00	-0,04	0,11
**PA1101**	68046	5,00	376,34	10	18,44	0,93	-0,11	-0,47	0,64	1399	**0,99**	0,94	-0,09	-0,43	0,67	1488	**0,97**	0,87	-0,21	-0,76	0,44	977	**0,94**	0,91	3,00	-0,14	0,04
**PA1102**	39722	4,92	306,05	9	25,71	0,90	-0,15	-0,62	0,54	1181	**0,99**	1,10	0,14	0,41	0,68	1518	**0,97**	0,88	-0,19	-0,70	0,49	1057	**0,95**	0,96	3,00	-0,06	0,12
**PA1104**	50536	6,30	259,74	8	14,84	0,89	-0,17	-0,67	0,51	1126	**0,98**	0,98	-0,03	-0,21	0,83	1845	**0,98**	1,09	0,13	0,37	0,71	1464	**1,00**	0,99	3,00	-0,02	0,10
**PA1116**	34361	8,98	214,11	6	16,70	1,27	0,34	1,11	0,27	687	**0,84**	1,13	0,18	0,57	0,57	1277	**0,97**	1,11	0,16	0,47	0,64	1306	**1,00**	1,17	3,00	0,23	0,08
**PA1117**	15021	9,89	93,96	2	40,30	1,05	0,08	0,18	0,85	1879	**0,99**	1,18	0,24	0,81	0,42	961	**0,94**	1,90	0,92	3,07	0,00	164	**0,03**	1,38	3,00	0,46	0,45

**PA1119**	19565	8,07	110,06	4	20,55	1,08	0,11	0,32	0,75	1648	**0,99**	1,05	0,07	0,17	0,86	1909	**0,98**	1,19	0,25	0,80	0,43	938	**0,94**	1,11	3,00	0,15	0,07
**PA1127**	37939	5,36	1007,49	21	54,66	1,15	0,20	0,61	0,54	1197	**0,99**	1,27	0,35	1,20	0,23	633	**0,79**	1,20	0,26	0,84	0,40	896	**0,92**	1,21	3,00	0,27	0,06
**PA1135**	35186	6,00	65,22	3	14,40	1,06	0,08	0,19	0,85	1866	**0,99**	1,05	0,07	0,17	0,87	1912	**0,98**	0,94	-0,09	-0,38	0,70	1449	**1,00**	1,01	3,00	0,02	0,07
**PA1140**	32982	5,88	172,39	5	15,82	1,50	0,59	1,98	0,05	280	**0,37**	1,52	0,61	2,16	0,03	293	**0,23**	1,25	0,32	1,04	0,30	721	**0,86**	1,43	3,00	0,51	0,15
**PA1155**	50756	4,76	1363,82	28	43,28	1,08	0,11	0,29	0,77	1697	**0,99**	1,05	0,07	0,15	0,88	1934	**0,98**	1,03	0,04	0,09	0,93	1912	**1,00**	1,05	3,00	0,07	0,02
**PA1156**	115137	5,61	4561,65	103	56,48	1,02	0,02	0,00	1,00	2169	**1,00**	1,03	0,04	0,05	0,96	2079	**1,00**	1,03	0,05	0,11	0,91	1875	**1,00**	1,03	3,00	0,04	0,01
**PA1159**	8585	6,73	434,11	11	81,81	1,65	0,72	2,45	0,01	211	**0,15**	1,75	0,81	2,91	0,00	189	**0,04**	1,64	0,71	2,36	0,02	246	**0,15**	1,68	3,00	0,75	0,06
**PA1161**	30622	7,67	227,84	7	35,60	1,30	0,38	1,25	0,21	594	**0,77**	1,12	0,16	0,49	0,62	1402	**0,96**	1,39	0,47	1,55	0,12	442	**0,57**	1,27	3,00	0,34	0,14
**PA1162**	42579	5,17	326,93	10	25,24	1,12	0,17	0,50	0,61	1351	**0,99**	1,29	0,37	1,28	0,20	587	**0,74**	1,35	0,43	1,39	0,16	523	**0,64**	1,25	3,00	0,33	0,12
**PA1165**	27512	6,45	129,34	4	17,40	1,04	0,06	0,14	0,89	1949	**0,99**	1,07	0,10	0,28	0,78	1715	**0,98**	1,27	0,34	1,10	0,27	667	**0,84**	1,13	3,00	0,17	0,12
**PA1171**	45486	9,00	782,11	15	38,43	1,15	0,20	0,64	0,52	1152	**0,99**	1,12	0,16	0,51	0,61	1371	**0,96**	1,13	0,17	0,52	0,60	1252	**0,99**	1,13	3,00	0,18	0,02
**PA1178**	23578	9,00	1307,00	20	55,25	0,96	-0,06	-0,28	0,78	1709	**0,99**	1,03	0,04	0,07	0,95	2062	**0,99**	1,30	0,37	1,21	0,23	604	**0,77**	1,10	3,00	0,13	0,18
**PA1179**	27023	5,34	668,93	13	61,31	1,14	0,19	0,57	0,57	1251	**0,98**	1,07	0,09	0,25	0,81	1780	**0,98**	1,10	0,13	0,39	0,69	1424	**1,00**	1,10	3,00	0,14	0,04
**PA1183**	48787	8,19	201,18	4	14,73	0,73	-0,46	-1,70	0,09	377	**0,52**	0,67	-0,59	-2,28	0,02	271	**0,18**	0,62	-0,70	-2,43	0,02	235	**0,13**	0,67	3,00	-0,58	0,06
**PA1192**	34254	6,15	295,29	11	36,19	1,13	0,17	0,52	0,60	1322	**0,99**	1,39	0,47	1,66	0,10	432	**0,49**	1,42	0,51	1,67	0,09	388	**0,50**	1,31	3,00	0,39	0,16
**PA1193**	27241	8,89	64,72	2	8,10	1,00	0,00	-0,06	0,95	2064	**1,00**	1,13	0,18	0,57	0,57	1279	**0,96**	1,55	0,64	2,10	0,04	287	**0,26**	1,23	3,00	0,30	0,29
**PA1196**	51082	6,30	101,86	3	9,80	1,65	0,72	2,46	0,01	209	**0,15**	1,05	0,07	0,17	0,87	1913	**0,98**	1,05	0,07	0,18	0,86	1772	**1,00**	1,25	3,00	0,32	0,35
**PA1202**	24135	5,03	50,65	2	7,35	1,14	0,19	0,59	0,56	1224	**0,99**	0,92	-0,11	-0,52	0,60	1346	**0,97**	1,51	0,59	1,95	0,05	312	**0,34**	1,19	3,00	0,25	0,30
**PA1203**	15197	5,12	227,81	4	27,40	0,58	-0,80	-2,87	0,00	163	**0,05**	0,72	-0,47	-1,86	0,06	369	**0,37**	0,80	-0,32	-1,15	0,25	637	**0,81**	0,70	3,00	-0,52	0,11
**PA1206**	18347	4,62	334,75	8	38,20	0,85	-0,23	-0,88	0,38	889	**0,92**	0,72	-0,48	-1,88	0,06	363	**0,36**	0,72	-0,48	-1,69	0,09	375	**0,50**	0,76	3,00	-0,39	0,08
**PA1207**	69049	6,43	208,62	5	9,68	0,83	-0,27	-1,03	0,30	754	**0,88**	0,99	-0,01	-0,15	0,88	1951	**0,98**	0,98	-0,02	-0,14	0,89	1834	**1,00**	0,93	3,00	-0,10	0,09
**PA1222**	44328	5,73	131,12	4	8,85	1,25	0,32	1,05	0,29	734	**0,87**	1,12	0,16	0,52	0,61	1354	**0,97**	1,43	0,51	1,69	0,09	382	**0,50**	1,27	3,00	0,34	0,15
**PA1225**	23160	6,44	124,89	3	13,00	0,87	-0,20	-0,78	0,44	999	**0,95**	1,00	-0,01	-0,12	0,90	1984	**0,98**	1,00	0,00	-0,06	0,95	1965	**1,00**	0,96	3,00	-0,07	0,07
**PA1226**	22963	5,40	259,37	6	32,20	1,21	0,27	0,87	0,39	910	**0,92**	0,89	-0,17	-0,73	0,46	1049	**0,96**	0,94	-0,09	-0,35	0,72	1493	**1,00**	1,01	3,00	0,02	0,17
**PA1244**	13726	8,29	151,50	5	35,92	0,81	-0,30	-1,14	0,26	674	**0,83**	1,36	0,44	1,55	0,12	481	**0,55**	1,16	0,21	0,67	0,50	1097	**0,95**	1,11	3,00	0,15	0,28
**PA1271**	69159	5,33	244,00	6	12,25	1,17	0,23	0,73	0,46	1036	**0,97**	1,09	0,12	0,36	0,72	1600	**0,97**	1,03	0,05	0,10	0,92	1896	**1,00**	1,10	3,00	0,14	0,07
**PA1272**	24584	7,08	383,99	9	33,11	1,19	0,25	0,80	0,42	975	**0,94**	1,13	0,18	0,57	0,57	1285	**0,96**	1,22	0,29	0,92	0,36	821	**0,89**	1,18	3,00	0,24	0,05
**PA1273**	47522	5,92	137,57	3	8,73	1,27	0,35	1,14	0,25	673	**0,82**	1,28	0,35	1,21	0,23	626	**0,78**	1,08	0,11	0,33	0,75	1541	**1,00**	1,21	3,00	0,27	0,11
**PA1275**	34186	8,78	108,67	4	14,88	1,06	0,09	0,23	0,81	1792	**0,99**	1,21	0,27	0,91	0,36	852	**0,92**	1,17	0,23	0,73	0,47	1032	**0,93**	1,15	3,00	0,20	0,07
**PA1276**	37296	7,75	92,04	2	4,80	1,32	0,40	1,34	0,18	541	**0,73**	1,45	0,53	1,89	0,06	357	**0,35**	1,26	0,33	1,06	0,29	702	**0,85**	1,34	3,00	0,42	0,10
**PA1278**	19512	5,49	160,40	5	27,02	1,05	0,08	0,19	0,85	1868	**0,99**	1,24	0,31	1,05	0,29	753	**0,84**	1,38	0,47	1,52	0,13	453	**0,58**	1,22	3,00	0,29	0,16
**PA1279**	37452	6,16	283,13	6	14,07	1,15	0,20	0,62	0,53	1170	**0,99**	1,14	0,19	0,59	0,55	1226	**0,97**	1,21	0,27	0,87	0,39	868	**0,92**	1,16	3,00	0,22	0,04
**PA1283**	21349	5,18	144,72	4	22,85	0,94	-0,09	-0,38	0,70	1555	**0,98**	1,12	0,17	0,53	0,60	1326	**0,97**	1,11	0,15	0,44	0,66	1363	**1,00**	1,06	3,00	0,08	0,10
**PA1287**	22118	8,32	64,29	2	11,95	1,06	0,08	0,21	0,83	1819	**0,99**	1,00	0,00	-0,08	0,94	2043	**0,99**	1,15	0,20	0,62	0,53	1144	**0,96**	1,07	3,00	0,10	0,07
**PA1288**	48992	5,74	1593,83	30	47,16	1,19	0,26	0,82	0,41	954	**0,94**	1,61	0,68	2,45	0,01	241	**0,13**	1,64	0,71	2,35	0,02	249	**0,16**	1,48	3,00	0,57	0,25
**PA1292**	31483	5,35	485,82	11	38,08	1,09	0,12	0,35	0,73	1598	**0,99**	0,91	-0,13	-0,58	0,56	1262	**0,97**	1,15	0,20	0,63	0,53	1137	**0,95**	1,05	3,00	0,07	0,12
**PA1293**	41508	5,74	428,56	9	27,51	1,02	0,03	0,04	0,97	2109	**1,00**	1,09	0,12	0,36	0,72	1599	**0,97**	0,97	-0,04	-0,20	0,85	1735	**1,00**	1,03	3,00	0,04	0,06
**PA1294**	45097	5,06	565,90	14	39,20	1,22	0,29	0,92	0,36	850	**0,91**	1,14	0,19	0,63	0,53	1197	**0,96**	1,05	0,07	0,19	0,85	1751	**1,00**	1,14	3,00	0,19	0,08
**PA1304**	78174	4,94	932,65	24	27,21	1,17	0,22	0,70	0,48	1080	**0,97**	1,30	0,37	1,29	0,20	577	**0,73**	1,32	0,40	1,30	0,19	558	**0,71**	1,26	3,00	0,33	0,08
**PA1307**	30007	5,59	107,53	2	11,30	1,24	0,31	1,00	0,32	776	**0,89**	1,11	0,16	0,49	0,63	1407	**0,96**	1,20	0,27	0,84	0,40	895	**0,92**	1,18	3,00	0,24	0,06
**PA1315**	23161	5,83	160,68	3	27,50	1,39	0,48	1,59	0,11	420	**0,58**	0,99	-0,01	-0,13	0,89	1968	**0,98**	0,92	-0,13	-0,49	0,63	1290	**1,00**	1,10	3,00	0,14	0,26
**PA1324**	19923	5,65	72,07	4	19,43	0,80	-0,32	-1,19	0,23	632	**0,80**	0,96	-0,05	-0,29	0,77	1704	**0,98**	1,02	0,02	0,02	0,98	2038	**1,00**	0,93	3,00	-0,11	0,11
**PA1330**	30178	6,03	162,71	3	18,50	0,76	-0,40	-1,50	0,13	461	**0,63**	1,05	0,06	0,14	0,89	1956	**0,98**	1,00	0,00	-0,06	0,95	1967	**1,00**	0,93	3,00	-0,10	0,16
**PA1335**	49050	6,39	130,22	5	12,72	1,03	0,04	0,06	0,95	2081	**1,00**	1,03	0,04	0,05	0,96	2095	**0,99**	0,97	-0,05	-0,22	0,82	1690	**1,01**	1,01	3,00	0,01	0,03
**PA1337**	42079	6,67	525,00	14	38,25	1,27	0,34	1,12	0,26	681	**0,84**	1,28	0,36	1,25	0,21	610	**0,75**	1,32	0,40	1,31	0,19	551	**0,71**	1,29	3,00	0,37	0,03
**PA1338**	65024	5,88	660,27	17	26,52	1,86	0,89	3,06	0,00	148	**0,03**	1,76	0,81	2,93	0,00	186	**0,04**	1,85	0,89	2,94	0,00	175	**0,04**	1,82	3,00	0,86	0,06
**PA1339**	29723	6,25	190,88	6	22,00	1,07	0,10	0,25	0,80	1757	**0,99**	0,98	-0,04	-0,23	0,82	1809	**0,98**	1,04	0,06	0,13	0,90	1846	**1,00**	1,03	3,00	0,04	0,05
**PA1341**	28676	9,14	105,40	4	13,00	1,01	0,02	-0,01	0,99	2160	**0,99**	0,96	-0,06	-0,31	0,76	1674	**0,98**	0,90	-0,15	-0,57	0,57	1211	**0,97**	0,96	3,00	-0,06	0,06

**PA1342**	37736	8,30	571,51	17	45,75	0,96	-0,06	-0,29	0,77	1700	**0,99**	1,09	0,13	0,38	0,71	1570	**0,97**	1,12	0,16	0,50	0,62	1283	**1,00**	1,06	3,00	0,08	0,09
**PA1344**	28481	5,18	124,18	3	14,63	0,89	-0,17	-0,68	0,50	1107	**0,98**	0,88	-0,19	-0,79	0,43	994	**0,94**	1,22	0,29	0,92	0,36	823	**0,90**	1,00	3,00	-0,01	0,19
**PA1357**	17926	5,40	192,81	3	18,13	1,05	0,08	0,18	0,85	1880	**0,99**	0,95	-0,08	-0,38	0,70	1565	**0,97**	1,15	0,20	0,62	0,54	1158	**0,96**	1,05	3,00	0,07	0,10
**PA1365**	91780	5,16	69,55	3	5,00	1,38	0,46	1,55	0,12	440	**0,60**	1,07	0,09	0,25	0,80	1774	**0,98**	1,46	0,54	1,78	0,07	355	**0,43**	1,30	3,00	0,38	0,21
**PA1367**	30439	4,77	171,18	4	12,95	1,11	0,15	0,46	0,65	1430	**0,98**	0,92	-0,12	-0,54	0,59	1311	**0,97**	1,37	0,46	1,49	0,14	467	**0,60**	1,14	3,00	0,18	0,23
**PA1372**	86145	5,53	760,28	20	16,81	1,05	0,07	0,17	0,87	1900	**0,99**	1,02	0,04	0,03	0,97	2112	**1,00**	0,99	-0,02	-0,11	0,91	1869	**1,00**	1,02	3,00	0,03	0,03
**PA1373**	44939	5,96	151,90	4	12,43	1,00	0,00	-0,09	0,92	2004	**1,00**	1,00	0,01	-0,07	0,94	2049	**0,99**	0,87	-0,20	-0,73	0,47	1033	**0,93**	0,96	3,00	-0,06	0,07
**PA1375**	41921	5,53	174,28	5	19,20	1,02	0,03	0,04	0,97	2110	**1,00**	1,06	0,09	0,22	0,82	1817	**0,98**	1,16	0,21	0,66	0,51	1104	**0,95**	1,08	3,00	0,11	0,07
**PA1383**	66603	5,18	103,51	2	5,00	1,13	0,18	0,53	0,59	1301	**0,99**	1,17	0,23	0,75	0,45	1031	**0,95**	1,10	0,14	0,42	0,68	1387	**1,00**	1,13	3,00	0,18	0,03
**PA1421**	36345	5,21	246,08	5	18,92	0,74	-0,44	-1,64	0,10	403	**0,55**	0,83	-0,27	-1,12	0,26	695	**0,82**	0,75	-0,41	-1,45	0,15	495	**0,62**	0,77	3,00	-0,37	0,05
**PA1430**	28371	6,09	377,97	10	47,48	1,15	0,20	0,62	0,54	1180	**0,99**	1,19	0,25	0,84	0,40	927	**0,93**	1,35	0,44	1,43	0,15	510	**0,62**	1,23	3,00	0,30	0,11
**PA1432**	24544	6,11	141,00	4	17,15	0,87	-0,20	-0,80	0,43	978	**0,94**	1,15	0,20	0,66	0,51	1147	**0,97**	0,90	-0,16	-0,58	0,56	1198	**0,96**	0,97	3,00	-0,04	0,16
**PA1433**	73740	6,09	108,37	3	8,50	1,39	0,48	1,59	0,11	421	**0,57**	1,40	0,48	1,70	0,09	417	**0,46**	1,21	0,28	0,89	0,38	852	**0,91**	1,33	3,00	0,42	0,10
**PA1440**	23272	4,14	144,28	4	16,65	1,27	0,35	1,15	0,25	669	**0,82**	1,13	0,18	0,57	0,57	1265	**0,97**	1,16	0,22	0,68	0,49	1077	**0,95**	1,19	3,00	0,25	0,07
**PA1441**	47467	4,81	148,05	4	11,10	1,08	0,12	0,32	0,75	1637	**0,99**	1,10	0,14	0,41	0,68	1513	**0,97**	1,20	0,26	0,84	0,40	898	**0,92**	1,13	3,00	0,17	0,06
**PA1442**	21054	9,62	77,71	2	15,00	0,74	-0,44	-1,64	0,10	404	**0,55**	0,97	-0,04	-0,24	0,81	1783	**0,98**	1,17	0,22	0,70	0,49	1061	**0,95**	0,96	3,00	-0,06	0,22
**PA1443**	38276	5,20	360,98	10	28,26	1,06	0,09	0,22	0,83	1815	**0,99**	1,15	0,20	0,65	0,52	1162	**0,96**	1,09	0,13	0,37	0,71	1467	**1,00**	1,10	3,00	0,14	0,04
**PA1444**	17474	4,22	212,49	4	22,90	0,94	-0,09	-0,38	0,70	1556	**0,98**	1,15	0,21	0,67	0,50	1128	**0,97**	1,08	0,11	0,33	0,74	1532	**1,00**	1,06	3,00	0,08	0,11
**PA1452**	79159	6,16	306,58	8	12,58	0,91	-0,13	-0,55	0,58	1285	**0,99**	0,90	-0,15	-0,66	0,51	1143	**0,96**	0,90	-0,15	-0,57	0,57	1213	**0,97**	0,90	3,00	-0,14	0,01
**PA1454**	31983	8,54	378,70	11	34,08	1,15	0,20	0,61	0,54	1202	**0,98**	1,04	0,05	0,09	0,93	2026	**0,99**	0,94	-0,09	-0,36	0,72	1482	**1,00**	1,04	3,00	0,06	0,10
**PA1455**	28992	5,70	192,99	5	24,20	1,10	0,14	0,42	0,67	1476	**0,99**	0,99	-0,02	-0,16	0,87	1924	**0,98**	0,92	-0,13	-0,49	0,62	1288	**1,00**	1,00	3,00	0,00	0,10
**PA1456**	15190	6,10	273,49	8	39,50	0,99	-0,01	-0,13	0,89	1962	**0,99**	1,08	0,11	0,32	0,75	1652	**0,98**	1,05	0,08	0,20	0,84	1731	**1,00**	1,04	3,00	0,06	0,05
**PA1457**	31235	4,87	486,62	13	50,42	1,10	0,13	0,39	0,70	1535	**0,99**	1,04	0,06	0,12	0,90	1986	**0,98**	1,10	0,14	0,42	0,67	1383	**1,00**	1,08	3,00	0,11	0,03
**PA1458**	85014	4,55	1249,53	24	28,37	1,11	0,15	0,45	0,65	1442	**0,98**	1,15	0,20	0,64	0,52	1182	**0,96**	1,08	0,11	0,30	0,76	1574	**1,00**	1,11	3,00	0,15	0,03
**PA1459**	41561	8,69	861,62	15	40,70	1,18	0,24	0,77	0,44	1000	**0,95**	1,13	0,18	0,57	0,57	1284	**0,96**	1,25	0,32	1,03	0,30	728	**0,86**	1,19	3,00	0,25	0,06
**PA1461**	33405	5,36	157,61	4	8,80	1,08	0,11	0,31	0,75	1653	**0,99**	1,39	0,48	1,68	0,09	427	**0,47**	1,04	0,06	0,16	0,88	1803	**1,00**	1,17	3,00	0,23	0,19
**PA1462**	29975	9,55	298,76	8	22,55	1,04	0,06	0,14	0,89	1952	**0,99**	0,91	-0,13	-0,59	0,55	1223	**0,98**	1,15	0,20	0,61	0,54	1162	**0,96**	1,03	3,00	0,05	0,12
**PA1464**	18580	4,33	1009,61	19	90,62	1,32	0,41	1,34	0,18	537	**0,72**	1,24	0,31	1,07	0,29	734	**0,84**	1,20	0,27	0,85	0,39	883	**0,92**	1,26	3,00	0,33	0,06
**PA1475**	25884	5,64	276,63	5	21,14	1,21	0,28	0,90	0,37	872	**0,91**	1,31	0,39	1,36	0,17	545	**0,69**	1,39	0,48	1,57	0,12	433	**0,56**	1,31	3,00	0,39	0,09
**PA1479**	18925	6,34	221,74	5	21,50	1,20	0,26	0,82	0,41	951	**0,94**	1,54	0,62	2,22	0,03	281	**0,20**	1,53	0,61	2,01	0,04	301	**0,31**	1,42	3,00	0,51	0,19
**PA1480**	74064	9,43	201,54	5	9,10	1,19	0,25	0,80	0,43	982	**0,94**	1,32	0,40	1,40	0,16	531	**0,66**	1,26	0,33	1,06	0,29	697	**0,85**	1,26	3,00	0,33	0,07
**PA1481**	21625	5,38	148,61	4	22,20	1,25	0,33	1,06	0,29	720	**0,87**	1,62	0,69	2,49	0,01	236	**0,12**	1,49	0,58	1,90	0,06	320	**0,37**	1,45	3,00	0,54	0,18
**PA1482**	18869	9,43	145,17	5	20,12	1,32	0,40	1,33	0,18	545	**0,73**	1,20	0,26	0,87	0,38	887	**0,93**	1,55	0,64	2,10	0,04	285	**0,26**	1,36	3,00	0,44	0,18
**PA1483**	44997	5,10	515,85	12	31,18	1,48	0,56	1,89	0,06	300	**0,42**	1,45	0,54	1,92	0,06	353	**0,34**	1,47	0,56	1,84	0,07	336	**0,41**	1,47	3,00	0,55	0,01
**PA1493**	40209	7,90	561,32	14	44,64	0,88	-0,18	-0,73	0,47	1045	**0,97**	1,07	0,09	0,25	0,81	1777	**0,98**	0,90	-0,16	-0,58	0,56	1197	**0,96**	0,95	3,00	-0,08	0,10
**PA1494**	64184	5,71	348,97	9	13,70	1,09	0,13	0,37	0,71	1565	**0,98**	1,10	0,14	0,43	0,66	1487	**0,97**	1,00	-0,01	-0,08	0,94	1938	**1,00**	1,06	3,00	0,09	0,06
**PA1504**	26032	6,97	195,96	4	19,65	1,39	0,48	1,59	0,11	422	**0,57**	1,44	0,52	1,85	0,06	375	**0,37**	1,87	0,91	3,01	0,00	170	**0,03**	1,57	3,00	0,65	0,27
**PA1516**	19939	5,01	260,25	7	44,47	1,14	0,19	0,60	0,55	1216	**0,99**	1,35	0,44	1,53	0,13	490	**0,55**	0,98	-0,03	-0,17	0,87	1780	**1,00**	1,16	3,00	0,21	0,19
**PA1517**	36900	5,55	94,37	4	13,80	1,00	0,00	-0,06	0,95	2065	**1,00**	1,20	0,26	0,89	0,37	874	**0,93**	1,40	0,48	1,58	0,11	426	**0,55**	1,20	3,00	0,26	0,20
**PA1518**	14313	6,03	116,00	3	29,37	0,71	-0,49	-1,80	0,07	336	**0,47**	0,97	-0,05	-0,28	0,78	1720	**0,98**	1,01	0,02	0,01	0,99	2044	**1,00**	0,90	3,00	-0,16	0,16
**PA1520**	30404	7,21	789,25	16	58,50	1,07	0,10	0,27	0,79	1740	**0,99**	1,12	0,16	0,51	0,61	1370	**0,96**	1,14	0,19	0,59	0,55	1184	**0,96**	1,11	3,00	0,15	0,04
**PA1521**	50293	5,31	345,22	7	19,79	1,25	0,32	1,03	0,30	747	**0,88**	1,23	0,30	1,04	0,30	760	**0,85**	1,23	0,30	0,97	0,33	782	**0,87**	1,24	3,00	0,31	0,01
**PA1526**	25821	7,04	266,97	6	34,25	1,22	0,29	0,93	0,35	833	**0,91**	1,72	0,78	2,80	0,01	201	**0,05**	1,53	0,61	2,02	0,04	299	**0,30**	1,49	3,00	0,57	0,25
**PA1527**	136775	5,08	248,74	9	8,51	1,14	0,19	0,60	0,55	1209	**0,99**	1,21	0,28	0,93	0,35	843	**0,90**	1,42	0,51	1,66	0,10	390	**0,51**	1,26	3,00	0,33	0,15
**PA1528**	35099	5,64	836,23	16	54,47	1,32	0,40	1,33	0,18	546	**0,73**	1,20	0,26	0,89	0,37	873	**0,92**	1,31	0,39	1,26	0,21	576	**0,75**	1,28	3,00	0,35	0,07
**PA1529**	91595	5,29	1415,03	34	38,31	1,26	0,34	1,10	0,27	701	**0,84**	1,24	0,31	1,06	0,29	745	**0,84**	1,32	0,40	1,29	0,20	560	**0,72**	1,27	3,00	0,35	0,04
**PA1530**	15001	7,98	70,92	2	32,00	1,25	0,32	1,05	0,29	733	**0,87**	1,36	0,44	1,56	0,12	470	**0,55**	1,49	0,57	1,88	0,06	326	**0,38**	1,37	3,00	0,45	0,12
**PA1532**	76180	4,69	681,16	20	27,88	1,14	0,19	0,57	0,57	1255	**0,98**	1,23	0,30	1,00	0,32	794	**0,86**	1,15	0,21	0,64	0,52	1129	**0,96**	1,17	3,00	0,23	0,05

**PA1533**	13310	5,11	687,66	12	45,40	1,17	0,23	0,72	0,47	1061	**0,97**	1,24	0,31	1,06	0,29	741	**0,84**	1,23	0,30	0,96	0,34	792	**0,88**	1,21	3,00	0,28	0,04
**PA1534**	21966	5,75	155,67	4	27,53	1,03	0,04	0,05	0,96	2089	**1,00**	1,05	0,07	0,18	0,86	1888	**0,98**	1,08	0,11	0,32	0,75	1552	**1,00**	1,05	3,00	0,07	0,03
**PA1543**	20703	4,81	206,11	7	31,71	1,18	0,23	0,74	0,46	1033	**0,97**	1,11	0,15	0,45	0,65	1458	**0,97**	1,14	0,19	0,58	0,56	1199	**0,96**	1,14	3,00	0,19	0,03
**PA1544**	28783	5,40	531,16	13	35,93	0,99	-0,01	-0,12	0,91	1980	**1,00**	0,84	-0,25	-1,01	0,31	786	**0,86**	1,05	0,07	0,18	0,86	1776	**1,00**	0,96	3,00	-0,06	0,11
**PA1546**	53976	5,43	454,51	13	30,50	0,96	-0,06	-0,28	0,78	1710	**0,99**	1,04	0,06	0,12	0,90	1987	**0,98**	1,09	0,12	0,35	0,72	1491	**1,00**	1,03	3,00	0,04	0,06
**PA1550**	21334	5,33	120,45	3	16,57	1,56	0,64	2,16	0,03	255	**0,26**	1,46	0,54	1,93	0,05	349	**0,33**	1,59	0,67	2,22	0,03	270	**0,20**	1,54	3,00	0,62	0,07
**PA1551**	58264	8,79	91,85	2	5,00	1,55	0,63	2,14	0,03	259	**0,27**	1,75	0,81	2,93	0,00	187	**0,04**	1,86	0,90	2,98	0,00	174	**0,03**	1,72	3,00	0,78	0,16
**PA1552**	37832	5,75	1254,61	28	52,58	1,22	0,29	0,93	0,35	844	**0,91**	1,34	0,42	1,47	0,14	507	**0,60**	1,37	0,46	1,49	0,14	470	**0,60**	1,31	3,00	0,39	0,08
**PA1553**	24853	7,79	707,40	17	40,79	1,19	0,25	0,81	0,42	971	**0,94**	1,30	0,37	1,29	0,20	574	**0,74**	1,19	0,25	0,78	0,43	957	**0,94**	1,22	3,00	0,29	0,06
**PA1554**	54849	9,42	288,98	6	9,90	1,26	0,34	1,10	0,27	699	**0,84**	1,33	0,41	1,44	0,15	511	**0,63**	1,49	0,58	1,90	0,06	319	**0,37**	1,36	3,00	0,45	0,12
**PA1555**	36713	6,08	157,32	3	18,53	0,88	-0,18	-0,71	0,48	1078	**0,97**	1,46	0,55	1,95	0,05	341	**0,33**	1,30	0,38	1,24	0,21	583	**0,76**	1,22	3,00	0,28	0,30
**PA1561**	59300	5,62	1196,20	19	37,55	1,08	0,11	0,30	0,76	1677	**0,99**	1,09	0,13	0,38	0,71	1569	**0,97**	1,03	0,04	0,09	0,93	1919	**1,00**	1,07	3,00	0,09	0,03
**PA1562**	106278	5,43	883,67	19	21,22	0,80	-0,32	-1,21	0,23	624	**0,79**	0,95	-0,07	-0,35	0,73	1613	**0,97**	1,10	0,14	0,41	0,68	1401	**1,01**	0,95	3,00	-0,07	0,15
**PA1563**	42994	8,69	90,93	2	8,10	1,10	0,14	0,41	0,68	1506	**0,99**	1,07	0,10	0,28	0,78	1725	**0,98**	1,04	0,06	0,14	0,89	1829	**1,00**	1,07	3,00	0,10	0,03
**PA1564**	9816	5,84	82,94	3	21,50	0,94	-0,09	-0,41	0,68	1497	**0,99**	1,27	0,34	1,18	0,24	641	**0,80**	1,16	0,22	0,68	0,50	1083	**0,95**	1,12	3,00	0,17	0,17
**PA1574**	11060	4,57	221,75	5	48,40	1,14	0,19	0,59	0,55	1222	**0,99**	1,53	0,61	2,18	0,03	291	**0,22**	1,11	0,15	0,45	0,65	1348	**1,00**	1,26	3,00	0,33	0,23
**PA1576**	31754	5,35	83,37	3	7,83	1,15	0,20	0,64	0,52	1147	**0,99**	1,20	0,26	0,87	0,38	888	**0,93**	0,76	-0,40	-1,41	0,16	514	**0,63**	1,04	3,00	0,05	0,24
**PA1579**	25071	7,71	243,15	6	32,28	0,98	-0,04	-0,21	0,84	1842	**0,99**	1,03	0,04	0,06	0,95	2075	**0,99**	1,07	0,09	0,26	0,80	1643	**1,00**	1,02	3,00	0,03	0,05
**PA1580**	51831	6,47	1596,09	37	45,60	0,73	-0,46	-1,70	0,09	378	**0,52**	0,83	-0,26	-1,08	0,28	724	**0,83**	0,73	-0,45	-1,60	0,11	418	**0,54**	0,76	3,00	-0,39	0,06
**PA1581**	15217	9,78	216,63	6	40,65	1,00	0,00	-0,09	0,93	2030	**1,00**	0,97	-0,04	-0,26	0,79	1753	**0,98**	1,08	0,11	0,33	0,75	1538	**1,00**	1,02	3,00	0,02	0,06
**PA1582**	14052	8,98	179,90	4	16,40	1,13	0,18	0,54	0,59	1292	**0,99**	1,02	0,03	0,00	1,00	2159	**1,00**	1,15	0,21	0,64	0,52	1124	**0,95**	1,10	3,00	0,14	0,07
**PA1583**	67699	6,04	2823,41	62	59,21	0,98	-0,03	-0,20	0,84	1850	**0,99**	1,06	0,08	0,20	0,84	1864	**0,98**	1,06	0,08	0,21	0,84	1712	**1,01**	1,03	3,00	0,04	0,05
**PA1584**	28805	6,59	741,60	18	53,67	0,94	-0,08	-0,37	0,71	1568	**0,98**	1,03	0,05	0,08	0,94	2044	**0,99**	1,07	0,09	0,26	0,79	1628	**1,00**	1,01	3,00	0,02	0,06
**PA1585**	112612	6,10	3598,92	82	47,14	1,22	0,29	0,93	0,35	845	**0,91**	1,13	0,18	0,57	0,57	1272	**0,97**	1,20	0,26	0,83	0,40	904	**0,92**	1,18	3,00	0,24	0,05
**PA1586**	47184	5,40	2648,23	46	62,40	1,22	0,29	0,92	0,36	851	**0,91**	1,23	0,30	1,01	0,31	789	**0,85**	1,27	0,35	1,11	0,27	657	**0,83**	1,24	3,00	0,31	0,03
**PA1587**	55649	6,48	3050,60	59	72,03	1,22	0,29	0,94	0,35	829	**0,91**	1,18	0,23	0,78	0,44	1006	**0,94**	1,23	0,30	0,95	0,34	799	**0,88**	1,21	3,00	0,27	0,03
**PA1588**	46502	5,83	2920,32	58	72,63	1,40	0,48	1,61	0,11	417	**0,56**	1,40	0,49	1,73	0,08	411	**0,44**	1,41	0,50	1,62	0,10	410	**0,53**	1,40	3,00	0,49	0,01
**PA1589**	33883	5,79	2354,83	49	93,23	1,33	0,41	1,37	0,17	527	**0,70**	1,35	0,44	1,52	0,13	492	**0,56**	1,38	0,46	1,50	0,13	461	**0,59**	1,35	3,00	0,44	0,02
**PA1591**	29441	6,44	126,42	3	11,00	1,17	0,23	0,73	0,47	1042	**0,97**	1,25	0,32	1,09	0,27	715	**0,83**	1,41	0,50	1,64	0,10	398	**0,53**	1,28	3,00	0,35	0,12
**PA1596**	79261	5,13	3511,17	79	71,44	0,98	-0,02	-0,16	0,87	1919	**0,99**	1,17	0,23	0,76	0,45	1024	**0,94**	1,13	0,18	0,55	0,58	1224	**0,98**	1,10	3,00	0,13	0,10
**PA1597**	26922	5,66	228,35	5	18,30	0,77	-0,38	-1,42	0,16	503	**0,67**	0,95	-0,07	-0,35	0,73	1622	**0,97**	0,98	-0,04	-0,18	0,86	1768	**1,00**	0,90	3,00	-0,15	0,11
**PA1600**	47649	7,23	58,07	2	3,95	1,22	0,29	0,92	0,36	847	**0,91**	0,79	-0,34	-1,35	0,18	550	**0,70**	0,74	-0,43	-1,50	0,13	464	**0,59**	0,92	3,00	-0,12	0,26
**PA1601**	84005	8,77	172,01	5	7,90	0,84	-0,25	-0,96	0,34	804	**0,91**	0,60	-0,74	-2,86	0,00	192	**0,05**	0,58	-0,80	-2,76	0,01	198	**0,06**	0,67	3,00	-0,58	0,15
**PA1608**	59355	4,80	445,17	12	30,50	1,56	0,64	2,18	0,03	252	**0,25**	1,40	0,48	1,69	0,09	422	**0,46**	1,29	0,37	1,21	0,23	609	**0,77**	1,42	3,00	0,50	0,13
**PA1609**	45407	5,40	2612,65	43	56,96	1,03	0,05	0,09	0,93	2026	**1,00**	1,08	0,11	0,30	0,76	1681	**0,98**	1,08	0,10	0,30	0,77	1580	**1,00**	1,06	3,00	0,09	0,02
**PA1610**	20026	6,41	838,13	16	55,25	1,18	0,24	0,76	0,45	1015	**0,96**	1,12	0,17	0,53	0,60	1333	**0,97**	1,17	0,22	0,70	0,49	1062	**0,95**	1,16	3,00	0,21	0,03
**PA1614**	38906	5,88	705,76	19	54,95	1,11	0,15	0,46	0,64	1426	**0,98**	1,11	0,15	0,48	0,63	1421	**0,96**	1,07	0,10	0,27	0,79	1622	**1,00**	1,10	3,00	0,14	0,03
**PA1616**	17137	5,96	170,48	4	15,75	1,91	0,94	3,21	0,00	134	**0,02**	1,19	0,25	0,82	0,41	952	**0,94**	1,43	0,51	1,69	0,09	380	**0,50**	1,51	3,00	0,59	0,37
**PA1618**	16155	6,49	318,65	7	35,90	1,07	0,09	0,25	0,81	1776	**0,99**	1,05	0,07	0,16	0,87	1928	**0,98**	0,85	-0,24	-0,85	0,39	882	**0,92**	0,99	3,00	-0,02	0,12
**PA1623**	26206	5,85	331,03	10	33,18	1,17	0,22	0,71	0,48	1074	**0,97**	1,21	0,27	0,91	0,36	855	**0,92**	1,19	0,25	0,78	0,44	967	**0,93**	1,19	3,00	0,25	0,02
**PA1624**	30472	6,02	460,56	8	29,30	1,23	0,30	0,96	0,34	814	**0,90**	1,25	0,32	1,11	0,27	704	**0,82**	1,13	0,17	0,52	0,60	1250	**0,99**	1,20	3,00	0,27	0,07
**PA1634**	76829	6,33	205,97	5	11,98	1,95	0,96	3,30	0,00	126	**0,02**	2,92	1,55	5,67	0,00	68	**0,00**	1,99	0,99	3,31	0,00	146	**0,01**	2,29	3,00	1,19	0,55
**PA1639**	23348	5,49	56,76	2	12,70	0,84	-0,25	-0,96	0,34	807	**0,90**	0,83	-0,27	-1,10	0,27	712	**0,83**	0,92	-0,13	-0,48	0,63	1298	**1,00**	0,86	3,00	-0,21	0,05
**PA1640**	39500	5,05	150,13	5	11,34	0,99	-0,02	-0,14	0,89	1947	**0,99**	1,26	0,34	1,16	0,25	657	**0,81**	0,97	-0,05	-0,22	0,82	1689	**1,00**	1,07	3,00	0,10	0,17
**PA1642**	38526	4,94	569,62	13	39,70	1,17	0,23	0,72	0,47	1050	**0,97**	1,21	0,28	0,94	0,35	840	**0,90**	1,22	0,28	0,90	0,37	840	**0,91**	1,20	3,00	0,26	0,02
**PA1643**	44211	6,48	387,82	9	20,39	1,26	0,33	1,08	0,28	712	**0,85**	1,04	0,06	0,11	0,91	2000	**0,99**	1,31	0,39	1,26	0,21	571	**0,74**	1,20	3,00	0,27	0,14
**PA1644**	22348	5,42	96,62	2	17,80	1,19	0,25	0,79	0,43	988	**0,94**	1,05	0,07	0,17	0,87	1915	**0,98**	0,94	-0,08	-0,34	0,74	1526	**1,00**	1,06	3,00	0,09	0,12
**PA1654**	44673	5,46	473,63	12	30,77	1,40	0,48	1,61	0,11	416	**0,56**	1,37	0,45	1,58	0,11	463	**0,53**	1,62	0,70	2,30	0,02	258	**0,17**	1,46	3,00	0,55	0,14

**PA1657**	20171	4,82	253,89	7	30,26	0,89	-0,17	-0,67	0,50	1113	**0,98**	0,69	-0,53	-2,08	0,04	312	**0,26**	0,91	-0,13	-0,50	0,61	1275	**0,99**	0,83	3,00	-0,27	0,12
**PA1658**	59941	5,30	81,62	3	7,70	0,85	-0,24	-0,92	0,36	853	**0,91**	0,71	-0,50	-1,96	0,05	336	**0,32**	0,74	-0,43	-1,50	0,13	463	**0,59**	0,77	3,00	-0,38	0,07
**PA1662**	99641	5,52	120,33	4	7,80	1,36	0,44	1,46	0,14	481	**0,65**	1,06	0,08	0,21	0,83	1840	**0,98**	0,53	-0,91	-3,13	0,00	159	**0,02**	0,98	3,00	-0,03	0,42
**PA1673**	19139	5,77	70,51	2	12,70	1,02	0,03	0,03	0,98	2134	**1,00**	1,05	0,07	0,16	0,87	1918	**0,98**	1,70	0,77	2,54	0,01	221	**0,10**	1,26	3,00	0,33	0,38
**PA1674**	21658	6,96	495,55	12	52,88	1,21	0,28	0,89	0,37	883	**0,92**	1,23	0,30	1,03	0,30	767	**0,85**	1,03	0,04	0,06	0,95	1958	**1,00**	1,16	3,00	0,21	0,11
**PA1677**	21735	6,05	1077,89	19	67,17	0,82	-0,28	-1,07	0,28	716	**0,86**	0,78	-0,36	-1,45	0,15	509	**0,62**	0,79	-0,34	-1,22	0,22	592	**0,77**	0,80	3,00	-0,33	0,02
**PA1678**	34962	4,64	272,21	6	23,67	1,10	0,14	0,39	0,69	1524	**0,99**	1,43	0,51	1,82	0,07	384	**0,39**	1,23	0,30	0,95	0,34	800	**0,88**	1,25	3,00	0,32	0,17
**PA1681**	41217	6,16	787,77	16	33,16	1,13	0,18	0,56	0,58	1280	**0,98**	1,11	0,15	0,46	0,65	1450	**0,97**	1,02	0,03	0,04	0,97	1988	**1,00**	1,09	3,00	0,12	0,06
**PA1683**	23954	6,05	210,92	4	34,10	1,08	0,11	0,32	0,75	1642	**0,99**	1,02	0,03	0,03	0,98	2124	**1,00**	1,01	0,02	0,01	0,99	2045	**1,00**	1,04	3,00	0,06	0,04
**PA1684**	21306	5,14	390,18	9	47,10	1,12	0,17	0,51	0,61	1333	**0,99**	1,16	0,21	0,69	0,49	1098	**0,96**	0,99	-0,01	-0,10	0,92	1891	**1,00**	1,09	3,00	0,13	0,09
**PA1685**	27654	4,88	202,74	5	16,10	1,05	0,07	0,17	0,87	1898	**0,99**	1,20	0,26	0,89	0,38	875	**0,93**	1,16	0,22	0,69	0,49	1071	**0,95**	1,14	3,00	0,19	0,08
**PA1687**	33076	5,12	671,38	18	46,80	1,01	0,02	-0,02	0,98	2139	**1,00**	1,00	0,00	-0,10	0,92	2010	**0,99**	0,95	-0,08	-0,33	0,74	1534	**1,00**	0,99	3,00	-0,02	0,03
**PA1689**	82154	6,06	918,33	21	27,97	1,69	0,76	2,59	0,01	190	**0,11**	1,51	0,59	2,10	0,04	305	**0,25**	1,44	0,53	1,74	0,08	364	**0,46**	1,55	3,00	0,63	0,13
**PA1714**	32532	5,14	207,50	5	20,10	0,57	-0,80	-2,90	0,00	160	**0,05**	0,52	-0,93	-3,57	0,00	138	**0,01**	0,66	-0,59	-2,07	0,04	291	**0,27**	0,59	3,00	-0,77	0,07
**PA1718**	7553	6,06	66,94	2	45,50	1,33	0,41	1,37	0,17	528	**0,71**	1,58	0,66	2,36	0,02	256	**0,15**	0,74	-0,44	-1,56	0,12	436	**0,56**	1,21	3,00	0,28	0,43
**PA1742**	28582	5,16	494,89	11	42,10	1,44	0,53	1,76	0,08	353	**0,48**	1,57	0,65	2,33	0,02	264	**0,16**	1,37	0,45	1,47	0,14	480	**0,60**	1,46	3,00	0,55	0,10
**PA1746**	19278	6,15	132,79	4	37,03	1,43	0,52	1,74	0,08	361	**0,49**	1,02	0,03	0,02	0,98	2135	**1,00**	0,97	-0,04	-0,20	0,85	1737	**1,00**	1,14	3,00	0,19	0,25
**PA1748**	26316	7,02	502,81	9	32,73	1,02	0,03	0,03	0,97	2123	**1,00**	1,05	0,08	0,19	0,85	1880	**0,98**	1,25	0,32	1,02	0,31	739	**0,85**	1,11	3,00	0,15	0,12
**PA1749**	18139	9,76	155,00	4	27,03	1,08	0,11	0,30	0,77	1681	**0,99**	1,03	0,05	0,07	0,94	2053	**0,99**	1,33	0,41	1,35	0,18	542	**0,67**	1,15	3,00	0,20	0,16
**PA1750**	42318	6,07	1042,17	21	39,00	1,03	0,04	0,07	0,95	2058	**1,00**	1,05	0,06	0,14	0,89	1954	**0,98**	1,15	0,20	0,63	0,53	1139	**0,95**	1,08	3,00	0,11	0,07
**PA1752**	35168	6,18	62,11	2	4,45	1,10	0,14	0,42	0,67	1477	**0,99**	1,04	0,05	0,10	0,92	2015	**0,99**	1,01	0,01	-0,01	0,99	2052	**1,00**	1,05	3,00	0,07	0,05
**PA1754**	39009	6,50	519,75	10	35,42	0,98	-0,02	-0,16	0,87	1920	**0,99**	0,99	-0,01	-0,14	0,89	1958	**0,98**	1,03	0,04	0,09	0,93	1920	**1,00**	1,00	3,00	0,00	0,02
**PA1756**	32444	6,01	107,95	5	12,22	1,42	0,51	1,70	0,09	376	**0,51**	1,46	0,55	1,94	0,05	344	**0,33**	1,37	0,45	1,48	0,14	476	**0,60**	1,42	3,00	0,50	0,05
**PA1757**	25307	5,17	327,11	10	51,70	1,10	0,13	0,39	0,70	1544	**0,98**	1,21	0,28	0,94	0,35	839	**0,90**	1,14	0,19	0,60	0,55	1172	**0,96**	1,15	3,00	0,20	0,06
**PA1758**	51670	5,88	269,28	7	12,44	1,04	0,06	0,14	0,89	1953	**0,99**	1,34	0,43	1,49	0,14	499	**0,59**	1,10	0,13	0,40	0,69	1419	**1,00**	1,16	3,00	0,22	0,16
**PA1766**	39345	6,50	301,96	8	34,35	1,34	0,43	1,42	0,16	504	**0,68**	1,68	0,75	2,68	0,01	212	**0,08**	1,65	0,72	2,39	0,02	239	**0,15**	1,56	3,00	0,64	0,18
**PA1767**	60235	8,77	946,76	22	25,91	1,39	0,48	1,60	0,11	418	**0,57**	1,46	0,54	1,92	0,05	352	**0,34**	1,59	0,67	2,22	0,03	269	**0,20**	1,48	3,00	0,57	0,10
**PA1768**	22583	9,18	305,54	8	37,70	0,92	-0,12	-0,50	0,62	1353	**0,99**	1,55	0,63	2,26	0,02	274	**0,19**	1,60	0,68	2,25	0,02	265	**0,19**	1,36	3,00	0,44	0,38
**PA1769**	33210	5,83	94,98	4	9,85	1,40	0,49	1,62	0,10	410	**0,55**	1,25	0,32	1,10	0,27	709	**0,82**	1,24	0,31	1,00	0,32	757	**0,86**	1,30	3,00	0,38	0,09
**PA1770**	91733	5,02	3061,44	65	50,82	1,19	0,25	0,78	0,43	993	**0,95**	1,28	0,36	1,24	0,21	614	**0,75**	1,24	0,31	0,98	0,33	772	**0,87**	1,24	3,00	0,30	0,05
**PA1771**	37708	6,01	167,02	4	13,03	1,46	0,55	1,85	0,06	317	**0,44**	1,48	0,56	2,00	0,05	325	**0,30**	1,54	0,62	2,04	0,04	294	**0,29**	1,49	3,00	0,58	0,04
**PA1772**	19116	4,83	245,19	5	32,82	0,83	-0,27	-1,02	0,31	757	**0,88**	1,13	0,18	0,57	0,57	1274	**0,96**	1,19	0,25	0,80	0,42	934	**0,93**	1,05	3,00	0,07	0,19
**PA1775**	30586	7,88	366,78	7	20,40	1,26	0,33	1,09	0,28	709	**0,85**	1,22	0,28	0,95	0,34	827	**0,89**	1,23	0,30	0,97	0,33	787	**0,87**	1,24	3,00	0,31	0,02
**PA1777**	40682	4,98	1862,99	40	68,41	1,27	0,34	1,12	0,26	680	**0,84**	1,47	0,55	1,96	0,05	339	**0,32**	1,53	0,61	2,02	0,04	300	**0,30**	1,42	3,00	0,51	0,14
**PA1787**	101392	5,22	4989,89	107	66,08	1,14	0,19	0,59	0,56	1232	**0,99**	1,15	0,20	0,64	0,52	1174	**0,96**	1,20	0,26	0,83	0,40	903	**0,92**	1,16	3,00	0,22	0,03
**PA1788**	19163	7,64	145,50	4	23,35	1,60	0,68	2,31	0,02	234	**0,20**	1,28	0,36	1,24	0,21	611	**0,76**	1,26	0,33	1,06	0,29	706	**0,85**	1,38	3,00	0,46	0,19
**PA1789**	33456	5,36	327,91	9	27,97	0,93	-0,10	-0,44	0,66	1453	**0,99**	1,04	0,06	0,11	0,91	2001	**0,98**	1,01	0,02	0,00	1,00	2060	**1,00**	0,99	3,00	-0,01	0,06
**PA1790**	25210	6,26	377,43	10	36,00	1,24	0,31	1,01	0,31	767	**0,88**	1,15	0,20	0,64	0,52	1180	**0,96**	1,52	0,61	2,00	0,05	302	**0,31**	1,30	3,00	0,38	0,20
**PA1791**	53253	5,83	597,21	16	32,50	1,43	0,52	1,73	0,08	364	**0,50**	1,29	0,36	1,25	0,21	607	**0,75**	1,45	0,54	1,77	0,08	357	**0,44**	1,39	3,00	0,47	0,09
**PA1792**	28540	7,86	52,61	2	4,80	1,27	0,35	1,15	0,25	670	**0,82**	1,13	0,18	0,58	0,56	1249	**0,97**	1,14	0,19	0,59	0,56	1189	**0,96**	1,18	3,00	0,24	0,08
**PA1793**	19988	5,79	881,81	21	49,07	1,31	0,39	1,27	0,20	582	**0,76**	1,54	0,62	2,21	0,03	283	**0,21**	1,61	0,69	2,28	0,02	260	**0,18**	1,48	3,00	0,57	0,16
**PA1794**	67126	5,56	1462,40	36	49,42	1,46	0,55	1,84	0,07	324	**0,44**	1,48	0,57	2,02	0,04	318	**0,29**	1,46	0,55	1,80	0,07	348	**0,43**	1,47	3,00	0,55	0,01
**PA1795**	54705	5,44	1669,89	40	50,45	1,41	0,49	1,64	0,10	399	**0,55**	1,39	0,48	1,68	0,09	428	**0,47**	1,36	0,44	1,44	0,15	499	**0,62**	1,38	3,00	0,47	0,02
**PA1796**	32011	5,58	687,25	15	36,55	1,42	0,51	1,70	0,09	380	**0,51**	1,48	0,56	1,99	0,05	327	**0,31**	1,73	0,79	2,61	0,01	214	**0,09**	1,54	3,00	0,62	0,16
**PA1798**	49519	5,33	44,80	2	3,40	1,10	0,14	0,42	0,67	1478	**0,99**	1,48	0,57	2,02	0,04	320	**0,30**	1,18	0,24	0,75	0,45	1000	**0,93**	1,26	3,00	0,33	0,20
**PA1799**	28212	5,88	86,73	3	11,90	0,94	-0,08	-0,38	0,71	1564	**0,98**	1,49	0,58	2,05	0,04	314	**0,28**	1,24	0,31	1,01	0,31	747	**0,86**	1,23	3,00	0,29	0,27
**PA1800**	52731	4,83	3911,17	74	72,92	1,21	0,27	0,87	0,39	906	**0,92**	1,24	0,31	1,05	0,30	754	**0,85**	1,27	0,34	1,11	0,27	662	**0,83**	1,24	3,00	0,31	0,03
**PA1801**	24922	5,96	496,65	12	33,00	1,05	0,07	0,17	0,87	1901	**0,99**	1,11	0,15	0,47	0,64	1433	**0,96**	1,07	0,09	0,25	0,80	1648	**1,00**	1,08	3,00	0,11	0,03

**PA1802**	52040	5,10	1876,95	40	53,28	1,03	0,04	0,07	0,94	2052	**1,00**	0,99	-0,01	-0,15	0,88	1946	**0,98**	1,07	0,10	0,30	0,77	1586	**1,00**	1,03	3,00	0,05	0,04
**PA1803**	97422	5,81	2662,83	68	52,65	1,02	0,03	0,02	0,99	2154	**0,99**	1,07	0,10	0,27	0,79	1737	**0,98**	1,02	0,03	0,03	0,98	2017	**1,00**	1,04	3,00	0,05	0,03
**PA1804**	10634	9,99	532,64	10	72,90	0,85	-0,24	-0,93	0,35	840	**0,91**	0,97	-0,04	-0,26	0,79	1748	**0,98**	0,95	-0,08	-0,31	0,75	1556	**1,00**	0,92	3,00	-0,12	0,07
**PA1805**	75472	4,99	2201,40	51	56,86	1,14	0,19	0,58	0,56	1238	**0,99**	1,15	0,20	0,65	0,52	1164	**0,96**	1,13	0,18	0,55	0,58	1225	**0,98**	1,14	3,00	0,19	0,01
**PA1806**	29567	5,65	263,20	7	23,00	1,21	0,27	0,88	0,38	890	**0,93**	1,15	0,20	0,65	0,51	1153	**0,96**	1,30	0,38	1,22	0,22	593	**0,77**	1,22	3,00	0,29	0,08
**PA1807**	61671	7,01	615,05	13	24,12	1,20	0,27	0,86	0,39	912	**0,93**	1,16	0,21	0,70	0,48	1091	**0,96**	1,30	0,38	1,24	0,21	584	**0,76**	1,22	3,00	0,29	0,07
**PA1810**	74989	6,09	324,45	7	15,14	1,24	0,31	1,01	0,31	770	**0,89**	1,29	0,37	1,27	0,20	596	**0,74**	1,30	0,38	1,22	0,22	596	**0,77**	1,28	3,00	0,35	0,03
**PA1812**	63779	9,81	145,46	4	16,33	1,07	0,10	0,27	0,79	1736	**0,99**	1,06	0,08	0,21	0,84	1849	**0,98**	1,06	0,08	0,21	0,84	1717	**1,00**	1,06	3,00	0,09	0,01
**PA1813**	30185	5,89	120,17	3	8,90	1,38	0,46	1,53	0,13	448	**0,61**	1,16	0,22	0,71	0,48	1076	**0,96**	1,17	0,23	0,72	0,47	1040	**0,93**	1,24	3,00	0,31	0,12
**PA1815**	18219	8,60	274,78	8	43,75	1,12	0,16	0,49	0,62	1370	**0,99**	1,26	0,33	1,14	0,25	674	**0,82**	1,33	0,41	1,33	0,18	548	**0,69**	1,24	3,00	0,30	0,11
**PA1816**	27656	5,21	293,71	6	25,47	1,08	0,12	0,33	0,74	1627	**0,99**	1,13	0,18	0,56	0,58	1299	**0,96**	1,04	0,06	0,15	0,88	1819	**1,00**	1,09	3,00	0,12	0,04
**PA1818**	86584	5,75	957,87	20	30,90	2,43	1,28	4,41	0,00	86	**0,00**	3,20	1,68	6,15	0,00	60	**0,00**	2,79	1,48	4,96	0,00	87	**0,00**	2,81	3,00	1,49	0,38
**PA1820**	55648	7,01	42,85	2	4,80	0,97	-0,04	-0,22	0,83	1813	**0,99**	1,14	0,18	0,59	0,55	1229	**0,98**	0,94	-0,09	-0,36	0,72	1480	**1,00**	1,02	3,00	0,02	0,11
**PA1821**	31805	5,51	369,95	9	38,14	1,07	0,10	0,26	0,80	1753	**0,99**	0,95	-0,07	-0,36	0,72	1598	**0,97**	1,11	0,15	0,46	0,65	1331	**1,00**	1,04	3,00	0,06	0,08
**PA1822**	62888	5,18	555,40	17	25,14	0,92	-0,13	-0,52	0,60	1325	**0,99**	0,97	-0,04	-0,26	0,79	1750	**0,98**	1,01	0,01	-0,01	0,99	2049	**1,00**	0,97	3,00	-0,05	0,05
**PA1823**	31946	5,11	115,42	4	9,15	1,10	0,14	0,41	0,68	1507	**0,99**	0,98	-0,03	-0,22	0,83	1821	**0,98**	1,17	0,22	0,70	0,48	1053	**0,94**	1,08	3,00	0,11	0,10
**PA1825**	24494	7,88	170,61	5	19,56	0,78	-0,36	-1,35	0,18	533	**0,72**	0,93	-0,11	-0,50	0,62	1392	**0,96**	0,79	-0,34	-1,21	0,23	603	**0,77**	0,83	3,00	-0,26	0,08
**PA1828**	29293	6,89	350,79	11	29,62	1,01	0,02	-0,02	0,98	2140	**1,00**	1,10	0,13	0,39	0,69	1544	**0,97**	1,03	0,04	0,07	0,94	1948	**1,00**	1,04	3,00	0,06	0,05
**PA1829**	43297	5,65	215,66	6	20,83	1,09	0,13	0,37	0,71	1570	**0,98**	1,07	0,09	0,25	0,80	1776	**0,98**	1,10	0,14	0,42	0,67	1381	**1,00**	1,09	3,00	0,12	0,02
**PA1830**	12157	4,24	547,46	12	61,87	1,21	0,28	0,90	0,37	880	**0,92**	1,11	0,15	0,45	0,65	1454	**0,97**	1,01	0,01	-0,02	0,99	2043	**1,00**	1,11	3,00	0,15	0,10
**PA1832**	41565	8,67	157,74	6	17,15	0,99	-0,02	-0,14	0,89	1950	**0,99**	1,24	0,31	1,06	0,29	740	**0,84**	1,07	0,10	0,27	0,79	1613	**1,00**	1,10	3,00	0,14	0,13
**PA1833**	37118	5,39	701,79	15	35,33	0,94	-0,09	-0,40	0,69	1519	**0,98**	1,04	0,06	0,13	0,90	1980	**0,98**	0,87	-0,21	-0,76	0,45	988	**0,94**	0,95	3,00	-0,08	0,09
**PA1837**	19376	4,88	258,04	6	38,15	1,40	0,49	1,63	0,10	408	**0,55**	1,56	0,64	2,29	0,02	269	**0,18**	1,46	0,55	1,81	0,07	345	**0,42**	1,48	3,00	0,56	0,08
**PA1838**	65586	5,68	1072,37	30	42,03	1,31	0,39	1,28	0,20	579	**0,76**	1,42	0,51	1,80	0,07	390	**0,40**	1,42	0,51	1,66	0,10	394	**0,51**	1,38	3,00	0,47	0,07
**PA1841**	19455	5,23	141,26	3	17,17	0,92	-0,12	-0,49	0,62	1374	**0,99**	1,06	0,09	0,23	0,82	1812	**0,98**	1,35	0,43	1,40	0,16	517	**0,64**	1,11	3,00	0,15	0,22
**PA1843**	144314	4,88	776,44	23	19,07	1,14	0,18	0,57	0,57	1259	**0,98**	1,07	0,09	0,25	0,80	1768	**0,98**	1,11	0,15	0,46	0,65	1335	**1,00**	1,10	3,00	0,14	0,04
**PA1846**	90290	5,78	307,74	10	20,45	1,24	0,31	1,02	0,31	762	**0,88**	0,94	-0,09	-0,45	0,66	1467	**0,97**	1,20	0,26	0,83	0,40	906	**0,92**	1,13	3,00	0,17	0,17
**PA1847**	22892	4,47	428,62	9	30,03	1,21	0,28	0,89	0,37	884	**0,92**	1,23	0,30	1,00	0,31	791	**0,86**	1,31	0,39	1,26	0,21	573	**0,75**	1,25	3,00	0,32	0,05
**PA1852**	10154	5,15	101,89	2	33,00	0,86	-0,22	-0,85	0,39	923	**0,93**	1,02	0,04	0,03	0,97	2113	**1,00**	0,78	-0,36	-1,28	0,20	565	**0,73**	0,89	3,00	-0,17	0,13
**PA1857**	34091	6,59	206,98	4	9,95	0,97	-0,04	-0,23	0,81	1787	**0,99**	1,21	0,27	0,92	0,36	847	**0,91**	1,17	0,23	0,73	0,47	1028	**0,93**	1,12	3,00	0,16	0,13
**PA1863**	28955	6,32	204,36	7	23,16	1,21	0,28	0,90	0,37	881	**0,91**	1,79	0,84	3,03	0,00	176	**0,03**	2,07	1,05	3,49	0,00	135	**0,01**	1,69	3,00	0,76	0,44
**PA1886**	92361	6,86	38,36	2	2,40	1,46	0,54	1,82	0,07	331	**0,45**	0,89	-0,16	-0,70	0,48	1087	**0,96**	1,03	0,05	0,10	0,92	1897	**1,00**	1,13	3,00	0,17	0,29
**PA1890**	23904	6,43	114,99	3	19,33	1,17	0,22	0,70	0,48	1081	**0,97**	0,97	-0,04	-0,24	0,81	1785	**0,98**	1,34	0,42	1,37	0,17	536	**0,66**	1,16	3,00	0,21	0,18
**PA1913**	26305	7,88	174,65	5	13,46	1,70	0,77	2,62	0,01	186	**0,10**	1,86	0,89	3,23	0,00	160	**0,02**	1,54	0,63	2,07	0,04	292	**0,27**	1,70	3,00	0,77	0,16
**PA1926**	71450	4,72	440,69	9	19,53	1,09	0,13	0,37	0,71	1577	**0,98**	1,09	0,13	0,37	0,71	1578	**0,97**	1,26	0,34	1,09	0,28	676	**0,84**	1,15	3,00	0,20	0,10
**PA1939**	78584	5,76	86,85	3	7,80	1,10	0,13	0,39	0,70	1543	**0,99**	1,13	0,18	0,57	0,57	1286	**0,96**	1,20	0,26	0,82	0,41	919	**0,92**	1,14	3,00	0,19	0,05
**PA1940**	45649	8,75	125,79	2	5,80	0,92	-0,12	-0,52	0,60	1329	**0,99**	1,33	0,41	1,44	0,15	513	**0,63**	1,69	0,76	2,52	0,01	224	**0,11**	1,31	3,00	0,39	0,39
**PA1941**	74306	7,67	227,28	9	14,48	1,49	0,58	1,95	0,05	289	**0,39**	1,31	0,38	1,33	0,18	556	**0,71**	1,48	0,57	1,87	0,06	328	**0,38**	1,43	3,00	0,51	0,11
**PA1949**	38094	6,68	147,74	5	16,28	1,75	0,81	2,76	0,01	174	**0,07**	2,42	1,27	4,65	0,00	95	**0,00**	1,52	0,60	1,98	0,05	305	**0,33**	1,89	3,00	0,92	0,47
**PA1950**	32731	5,15	157,50	6	15,75	1,55	0,63	2,13	0,03	260	**0,28**	1,18	0,24	0,80	0,42	972	**0,94**	1,41	0,50	1,62	0,10	409	**0,53**	1,38	3,00	0,46	0,18
**PA1964**	61766	5,04	769,52	19	33,11	1,39	0,47	1,58	0,11	426	**0,58**	1,16	0,21	0,68	0,50	1120	**0,96**	1,25	0,32	1,03	0,30	727	**0,86**	1,26	3,00	0,34	0,12
**PA1969**	14185	8,98	206,38	6	40,00	1,18	0,24	0,75	0,46	1025	**0,97**	1,11	0,15	0,48	0,63	1428	**0,96**	1,12	0,16	0,49	0,63	1292	**1,00**	1,14	3,00	0,18	0,04
**PA1998**	34790	6,10	75,41	4	13,30	1,27	0,34	1,12	0,26	682	**0,84**	1,04	0,06	0,13	0,89	1971	**0,98**	1,05	0,06	0,16	0,87	1797	**1,00**	1,12	3,00	0,16	0,13
**PA1999**	26617	5,26	499,70	9	41,03	3,63	1,86	6,45	0,00	41	**0,00**	5,88	2,56	9,42	0,00	10	**0,00**	5,98	2,58	8,69	0,00	8	**0,00**	5,17	3,00	2,37	1,33
**PA2000**	24751	4,84	661,82	13	38,93	3,19	1,67	5,79	0,00	56	**0,00**	3,24	1,70	6,23	0,00	58	**0,00**	5,52	2,46	8,29	0,00	1	**0,00**	3,98	3,00	1,99	1,33
**PA2001**	42870	6,03	2324,83	37	66,47	5,85	2,55	8,87	0,00	8	**0,00**	4,61	2,21	8,12	0,00	25	**0,00**	5,76	2,53	8,50	0,00	3	**0,00**	5,41	3,00	2,44	0,69
**PA2002**	51833	8,73	193,68	5	8,00	4,71	2,24	7,77	0,00	25	**0,00**	6,53	2,71	9,99	0,00	13	**0,00**	10,17	3,35	11,28	0,00	27	**0,00**	7,14	3,00	2,84	2,78
**PA2007**	24195	6,37	430,32	12	46,45	3,44	1,78	6,18	0,00	47	**0,00**	3,17	1,66	6,10	0,00	61	**0,00**	4,25	2,09	7,01	0,00	47	**0,00**	3,62	3,00	1,86	0,56

**PA2008**	47931	5,71	699,24	18	30,62	5,38	2,43	8,44	0,00	4	**0,00**	5,20	2,38	8,77	0,00	4	**0,00**	7,32	2,87	9,68	0,00	16	**0,00**	5,97	3,00	2,58	1,18
**PA2009**	49528	5,66	848,58	18	45,15	3,08	1,62	5,62	0,00	64	**0,00**	2,99	1,58	5,79	0,00	67	**0,00**	3,56	1,83	6,16	0,00	57	**0,00**	3,21	3,00	1,68	0,31
**PA2010**	29017	5,73	271,03	6	21,70	1,35	0,43	1,42	0,16	501	**0,67**	1,25	0,33	1,12	0,26	690	**0,82**	1,63	0,71	2,33	0,02	253	**0,16**	1,41	3,00	0,50	0,20
**PA2011**	33967	5,80	170,74	5	15,94	1,46	0,54	1,82	0,07	332	**0,45**	1,54	0,63	2,24	0,03	279	**0,19**	1,51	0,60	1,96	0,05	307	**0,33**	1,50	3,00	0,59	0,05
**PA2012**	73578	5,54	355,87	9	17,73	2,17	1,12	3,84	0,00	100	**0,00**	1,72	0,78	2,81	0,01	200	**0,05**	2,04	1,03	3,42	0,00	140	**0,01**	1,97	3,00	0,98	0,23
**PA2013**	30069	5,38	224,11	6	25,87	2,49	1,31	4,53	0,00	82	**0,00**	4,25	2,09	7,68	0,00	35	**0,00**	3,45	1,78	5,99	0,00	59	**0,00**	3,39	3,00	1,76	0,88
**PA2014**	60451	6,23	390,22	11	20,76	1,88	0,91	3,11	0,00	143	**0,03**	2,30	1,20	4,37	0,00	104	**0,00**	3,32	1,73	5,81	0,00	62	**0,00**	2,50	3,00	1,32	0,74
**PA2015**	44528	5,53	420,83	10	23,90	3,98	1,99	6,92	0,00	36	**0,00**	4,46	2,16	7,94	0,00	29	**0,00**	4,05	2,02	6,78	0,00	50	**0,00**	4,16	3,00	2,06	0,26
**PA2016**	16866	5,58	194,50	6	41,80	1,44	0,53	1,76	0,08	354	**0,48**	1,55	0,63	2,26	0,02	272	**0,19**	1,75	0,81	2,67	0,01	209	**0,07**	1,58	3,00	0,66	0,16
**PA2018**	115987	5,98	116,58	3	3,57	1,07	0,10	0,25	0,80	1758	**0,99**	1,02	0,03	0,00	1,00	2162	**1,00**	0,99	-0,01	-0,08	0,93	1923	**1,00**	1,03	3,00	0,04	0,04
**PA2019**	43837	5,97	302,27	9	22,33	0,98	-0,03	-0,18	0,86	1889	**0,99**	0,95	-0,08	-0,39	0,70	1549	**0,97**	0,87	-0,21	-0,76	0,45	990	**0,94**	0,93	3,00	-0,10	0,06
**PA2020**	25253	6,81	133,52	4	16,80	1,21	0,27	0,88	0,38	888	**0,92**	0,99	-0,01	-0,13	0,90	1979	**0,98**	1,15	0,20	0,64	0,52	1131	**0,96**	1,12	3,00	0,16	0,11
**PA2023**	34708	5,33	513,31	15	50,04	1,14	0,18	0,57	0,57	1260	**0,98**	1,19	0,25	0,84	0,40	926	**0,93**	1,12	0,17	0,51	0,61	1266	**0,99**	1,15	3,00	0,20	0,04
**PA2025**	52417	5,94	763,20	14	38,06	1,08	0,11	0,30	0,77	1682	**0,99**	1,02	0,03	0,03	0,98	2125	**1,00**	0,91	-0,13	-0,49	0,62	1286	**1,00**	1,01	3,00	0,01	0,08
**PA2040**	53291	4,77	295,42	4	36,25	1,07	0,10	0,25	0,80	1759	**0,99**	1,21	0,28	0,93	0,35	842	**0,90**	1,08	0,11	0,33	0,75	1540	**1,00**	1,12	3,00	0,16	0,08
**PA2042**	44008	7,07	213,80	6	8,80	1,14	0,18	0,56	0,57	1267	**0,98**	1,00	0,01	-0,07	0,94	2057	**0,99**	1,02	0,02	0,02	0,98	2026	**1,00**	1,05	3,00	0,07	0,07
**PA2044**	74299	5,85	1089,50	29	36,80	1,08	0,11	0,30	0,76	1678	**0,99**	1,15	0,21	0,68	0,50	1123	**0,96**	1,16	0,21	0,66	0,51	1102	**0,95**	1,13	3,00	0,18	0,05
**PA2071**	82707	5,11	209,81	5	22,26	0,98	-0,02	-0,17	0,87	1909	**0,99**	0,97	-0,04	-0,24	0,81	1784	**0,98**	1,15	0,20	0,62	0,54	1153	**0,96**	1,03	3,00	0,05	0,10
**PA2080**	47793	5,39	310,88	10	25,72	1,47	0,56	1,88	0,06	302	**0,43**	1,48	0,57	2,01	0,04	321	**0,30**	1,40	0,48	1,57	0,12	430	**0,56**	1,45	3,00	0,54	0,05
**PA2081**	23800	5,25	191,33	6	31,90	1,45	0,53	1,79	0,07	339	**0,47**	1,24	0,31	1,06	0,29	746	**0,84**	1,43	0,52	1,71	0,09	374	**0,48**	1,37	3,00	0,46	0,12
**PA2110**	33603	6,96	547,12	16	38,90	7,51	2,91	10,13	0,00	16	**0,00**	4,70	2,23	8,22	0,00	22	**0,00**	8,44	3,08	10,37	0,00	20	**0,00**	6,88	3,00	2,78	1,95
**PA2111**	26680	6,85	715,91	14	52,30	7,53	2,91	10,14	0,00	17	**0,00**	3,43	1,78	6,54	0,00	49	**0,00**	9,89	3,31	11,14	0,00	26	**0,00**	6,95	3,00	2,80	3,27
**PA2112**	26881	5,73	755,02	17	49,40	7,23	2,85	9,93	0,00	15	**0,00**	5,34	2,42	8,91	0,00	6	**0,00**	7,46	2,90	9,76	0,00	17	**0,00**	6,67	3,00	2,74	1,16
**PA2113**	46005	5,70	727,77	17	37,35	6,60	2,72	9,47	0,00	14	**0,00**	7,11	2,83	10,45	0,00	14	**0,00**	10,79	3,43	11,57	0,00	28	**0,00**	8,17	3,00	3,03	2,29
**PA2114**	47581	9,38	100,27	3	3,80	3,42	1,77	6,14	0,00	48	**0,00**	7,60	2,93	10,80	0,00	15	**0,00**	2,10	1,07	3,57	0,00	128	**0,01**	4,37	3,00	2,13	2,87
**PA2116**	29819	6,05	461,97	11	36,46	4,22	2,08	7,21	0,00	32	**0,00**	4,28	2,10	7,71	0,00	34	**0,00**	5,21	2,38	8,01	0,00	37	**0,00**	4,57	3,00	2,19	0,56
**PA2195**	46964	6,15	158,23	4	8,65	0,62	-0,69	-2,50	0,01	205	**0,13**	0,65	-0,63	-2,44	0,01	243	**0,13**	0,52	-0,95	-3,26	0,00	151	**0,02**	0,60	3,00	-0,75	0,07
**PA2197**	39071	5,93	512,06	13	35,19	0,82	-0,29	-1,09	0,28	706	**0,85**	0,92	-0,12	-0,56	0,58	1296	**0,96**	0,86	-0,22	-0,81	0,42	930	**0,93**	0,87	3,00	-0,21	0,05
**PA2198**	13172	5,38	112,22	2	34,20	0,90	-0,15	-0,60	0,55	1204	**0,98**	1,16	0,21	0,68	0,50	1121	**0,96**	0,90	-0,15	-0,55	0,58	1219	**0,98**	0,99	3,00	-0,02	0,15
**PA2199**	32543	5,47	369,42	9	31,74	0,84	-0,24	-0,94	0,35	830	**0,91**	0,84	-0,25	-1,01	0,31	787	**0,86**	0,71	-0,50	-1,74	0,08	363	**0,46**	0,80	3,00	-0,32	0,08
**PA2204**	32749	9,06	752,87	20	57,50	2,13	1,09	3,75	0,00	106	**0,00**	2,55	1,35	4,93	0,00	84	**0,00**	2,85	1,51	5,06	0,00	84	**0,00**	2,51	3,00	1,33	0,36
**PA2229**	27448	6,11	223,01	6	22,43	1,38	0,46	1,53	0,13	449	**0,61**	1,00	0,00	-0,11	0,92	2009	**0,99**	1,29	0,37	1,18	0,24	624	**0,78**	1,22	3,00	0,29	0,20
**PA2231**	57612	9,74	146,66	3	6,00	1,74	0,80	2,72	0,01	177	**0,08**	2,75	1,46	5,34	0,00	73	**0,00**	2,21	1,15	3,83	0,00	119	**0,00**	2,23	3,00	1,16	0,51
**PA2232**	55730	5,74	501,74	12	24,22	1,81	0,86	2,92	0,00	159	**0,05**	1,84	0,88	3,18	0,00	166	**0,02**	1,86	0,90	2,98	0,00	173	**0,03**	1,84	3,00	0,88	0,03
**PA2233**	34673	8,78	50,73	2	6,45	1,34	0,43	1,42	0,16	502	**0,68**	1,25	0,32	1,10	0,27	710	**0,82**	2,38	1,25	4,18	0,00	107	**0,00**	1,66	3,00	0,73	0,63
**PA2234**	29089	8,66	353,73	9	43,73	2,17	1,12	3,85	0,00	99	**0,00**	3,59	1,84	6,77	0,00	47	**0,00**	2,37	1,25	4,17	0,00	109	**0,00**	2,71	3,00	1,44	0,77
**PA2235**	78744	5,99	1381,86	34	46,68	2,33	1,22	4,21	0,00	91	**0,00**	2,16	1,11	4,05	0,00	112	**0,00**	2,40	1,26	4,22	0,00	105	**0,00**	2,30	3,00	1,20	0,12
**PA2236**	43819	8,59	122,68	4	11,83	2,00	1,00	3,42	0,00	123	**0,01**	1,69	0,76	2,72	0,01	207	**0,07**	2,67	1,42	4,74	0,00	90	**0,00**	2,12	3,00	1,08	0,50
**PA2237**	52778	5,72	98,75	4	8,25	1,85	0,89	3,04	0,00	151	**0,03**	2,19	1,13	4,11	0,00	110	**0,00**	2,38	1,25	4,19	0,00	106	**0,00**	2,14	3,00	1,10	0,27
**PA2238**	45691	6,53	79,82	3	8,70	2,00	1,00	3,43	0,00	120	**0,01**	1,72	0,78	2,81	0,00	197	**0,05**	3,29	1,72	5,77	0,00	64	**0,00**	2,34	3,00	1,23	0,84
**PA2239**	41431	8,52	136,80	4	9,55	1,62	0,70	2,37	0,02	222	**0,17**	1,85	0,89	3,20	0,00	163	**0,02**	1,61	0,68	2,26	0,02	261	**0,19**	1,69	3,00	0,76	0,13
**PA2242**	41730	9,60	44,67	2	3,90	2,60	1,38	4,75	0,00	78	**0,00**	2,11	1,08	3,92	0,00	121	**0,00**	3,31	1,73	5,79	0,00	63	**0,00**	2,67	3,00	1,42	0,60
**PA2247**	47331	5,68	833,48	21	44,84	3,18	1,67	5,77	0,00	57	**0,00**	3,34	1,74	6,39	0,00	55	**0,00**	5,16	2,37	7,96	0,00	38	**0,00**	3,89	3,00	1,96	1,10
**PA2248**	40207	5,37	377,21	10	22,30	5,59	2,48	8,63	0,00	6	**0,00**	3,42	1,77	6,52	0,00	50	**0,00**	11,76	3,56	11,99	0,00	30	**0,00**	6,92	3,00	2,79	4,33
**PA2249**	47619	5,84	1167,43	27	41,02	5,35	2,42	8,41	0,00	2	**0,00**	3,38	1,76	6,45	0,00	54	**0,00**	4,77	2,25	7,58	0,00	41	**0,00**	4,50	3,00	2,17	1,01
**PA2250**	51284	6,16	1676,45	33	61,59	4,37	2,13	7,39	0,00	29	**0,00**	5,69	2,51	9,25	0,00	8	**0,00**	5,24	2,39	8,03	0,00	36	**0,00**	5,10	3,00	2,35	0,67
**PA2252**	53156	8,75	1138,81	17	27,42	1,94	0,96	3,28	0,00	127	**0,02**	1,91	0,94	3,39	0,00	149	**0,01**	1,70	0,77	2,54	0,01	219	**0,10**	1,85	3,00	0,89	0,13
**PA2253**	35448	5,36	366,42	11	24,35	1,26	0,34	1,10	0,27	700	**0,84**	1,28	0,36	1,23	0,22	620	**0,76**	1,18	0,23	0,73	0,46	1022	**0,94**	1,24	3,00	0,31	0,06

**PA2264**	27783	5,41	392,59	9	38,27	0,88	-0,18	-0,72	0,47	1053	**0,97**	0,89	-0,17	-0,74	0,46	1044	**0,95**	0,99	-0,01	-0,10	0,92	1899	**1,00**	0,92	3,00	-0,12	0,06
**PA2265**	69084	6,76	556,39	18	27,43	1,07	0,09	0,25	0,80	1766	**0,99**	0,98	-0,02	-0,19	0,85	1879	**0,98**	1,10	0,14	0,41	0,68	1397	**1,00**	1,05	3,00	0,07	0,06
**PA2266**	48906	5,84	901,51	17	31,42	0,92	-0,12	-0,51	0,61	1344	**0,99**	1,06	0,08	0,19	0,85	1873	**0,98**	1,02	0,03	0,05	0,96	1980	**1,00**	1,00	3,00	0,00	0,07
**PA2285**	25714	7,03	164,94	4	19,30	1,10	0,14	0,41	0,68	1508	**0,99**	1,17	0,23	0,75	0,45	1036	**0,95**	1,24	0,31	1,00	0,32	752	**0,86**	1,17	3,00	0,23	0,07
**PA2289**	81197	5,52	118,88	4	4,75	1,41	0,49	1,64	0,10	398	**0,55**	1,14	0,19	0,63	0,53	1198	**0,96**	1,35	0,44	1,42	0,16	512	**0,63**	1,30	3,00	0,38	0,14
**PA2290**	90042	5,89	2404,79	46	49,14	1,65	0,72	2,44	0,01	212	**0,15**	1,64	0,72	2,57	0,01	222	**0,10**	1,60	0,67	2,23	0,03	267	**0,20**	1,63	3,00	0,70	0,03
**PA2291**	54353	5,45	1541,43	31	50,12	6,09	2,61	9,07	0,00	10	**0,00**	5,71	2,51	9,27	0,00	9	**0,00**	5,88	2,56	8,60	0,00	6	**0,00**	5,89	3,00	2,56	0,19
**PA2301**	20085	6,09	108,95	3	15,20	1,04	0,05	0,11	0,91	1985	**1,00**	1,07	0,09	0,25	0,81	1782	**0,98**	0,83	-0,27	-0,98	0,33	776	**0,87**	0,98	3,00	-0,03	0,13
**PA2302**	231559	5,07	327,50	11	8,26	1,14	0,19	0,57	0,57	1252	**0,98**	0,93	-0,10	-0,48	0,63	1429	**0,96**	0,96	-0,06	-0,24	0,81	1658	**1,00**	1,01	3,00	0,02	0,11
**PA2320**	38363	8,29	193,61	6	14,90	1,19	0,26	0,82	0,41	955	**0,94**	1,46	0,54	1,92	0,05	351	**0,34**	1,06	0,08	0,22	0,83	1693	**1,00**	1,24	3,00	0,31	0,20
**PA2323**	62845	6,00	962,49	21	40,54	1,03	0,04	0,05	0,96	2101	**1,00**	0,98	-0,03	-0,20	0,84	1860	**0,98**	1,03	0,04	0,07	0,95	1952	**1,00**	1,01	3,00	0,02	0,03
**PA2332**	33729	6,83	94,75	3	12,40	1,14	0,19	0,60	0,55	1210	**0,99**	1,10	0,14	0,41	0,68	1520	**0,97**	1,26	0,34	1,09	0,28	679	**0,84**	1,17	3,00	0,23	0,08
**PA2344**	33686	5,77	45,69	2	6,80	1,00	0,00	-0,07	0,95	2055	**1,00**	1,24	0,31	1,06	0,29	739	**0,84**	1,28	0,35	1,13	0,26	646	**0,82**	1,17	3,00	0,23	0,15
**PA2352**	43555	5,72	798,63	18	41,26	0,81	-0,31	-1,15	0,25	660	**0,82**	0,67	-0,58	-2,24	0,02	277	**0,19**	0,71	-0,49	-1,72	0,08	367	**0,48**	0,73	3,00	-0,45	0,07
**PA2378**	89699	8,44	1002,48	24	25,70	0,92	-0,11	-0,48	0,63	1387	**0,99**	0,99	-0,02	-0,17	0,86	1907	**0,98**	0,90	-0,15	-0,56	0,58	1215	**0,98**	0,94	3,00	-0,09	0,04
**PA2386**	51504	6,03	242,82	5	13,70	4,91	2,30	7,98	0,00	23	**0,00**	12,52	3,65	13,49	0,00	17	**0,00**	8,44	3,08	10,37	0,00	21	**0,00**	8,63	3,00	3,11	3,81
**PA2391**	52803	5,89	81,95	2	3,05	1,62	0,70	2,37	0,02	221	**0,17**	1,31	0,39	1,34	0,18	553	**0,70**	1,14	0,19	0,58	0,56	1202	**0,96**	1,36	3,00	0,44	0,25
**PA2396**	33037	5,15	102,42	2	9,80	2,22	1,15	3,96	0,00	96	**0,00**	3,67	1,88	6,90	0,00	43	**0,00**	6,20	2,63	8,86	0,00	11	**0,00**	4,03	3,00	2,01	2,01
**PA2398**	96587	5,38	131,18	4	7,08	1,82	0,86	2,94	0,00	158	**0,04**	1,84	0,88	3,17	0,00	168	**0,02**	2,82	1,50	5,01	0,00	86	**0,00**	2,16	3,00	1,11	0,57
**PA2400**	245182	5,24	123,55	4	3,35	2,26	1,18	4,05	0,00	95	**0,00**	2,92	1,55	5,67	0,00	69	**0,00**	1,27	0,34	1,10	0,27	666	**0,84**	2,15	3,00	1,10	0,83
**PA2402**	581138	5,24	265,62	10	2,96	2,16	1,11	3,81	0,00	103	**0,00**	2,02	1,02	3,69	0,00	134	**0,00**	3,19	1,67	5,61	0,00	69	**0,00**	2,46	3,00	1,30	0,64
**PA2410**	33390	5,19	139,22	4	14,90	1,59	0,67	2,26	0,02	242	**0,21**	1,61	0,69	2,46	0,01	239	**0,13**	2,64	1,40	4,69	0,00	92	**0,00**	1,95	3,00	0,96	0,60
**PA2424**	490269	5,29	50,01	2	1,60	2,07	1,05	3,61	0,00	113	**0,01**	2,81	1,49	5,46	0,00	71	**0,00**	2,32	1,21	4,05	0,00	112	**0,00**	2,40	3,00	1,26	0,38
**PA2442**	41528	5,95	1104,82	26	50,70	3,37	1,75	6,08	0,00	49	**0,00**	2,66	1,41	5,16	0,00	80	**0,00**	3,69	1,88	6,32	0,00	53	**0,00**	3,24	3,00	1,70	0,53
**PA2443**	51680	6,35	53,16	2	4,15	1,22	0,29	0,92	0,36	852	**0,91**	1,94	0,96	3,47	0,00	145	**0,01**	1,78	0,84	2,77	0,01	195	**0,06**	1,65	3,00	0,72	0,38
**PA2444**	48346	6,07	324,51	10	26,98	2,00	1,00	3,42	0,00	122	**0,01**	2,50	1,32	4,83	0,00	89	**0,00**	2,16	1,11	3,70	0,00	125	**0,00**	2,22	3,00	1,15	0,26
**PA2445**	108238	5,68	2464,51	56	43,90	3,05	1,61	5,57	0,00	65	**0,00**	3,32	1,73	6,36	0,00	56	**0,00**	5,45	2,44	8,23	0,00	33	**0,00**	3,94	3,00	1,98	1,31
**PA2446**	14361	4,10	167,58	5	29,10	4,22	2,08	7,20	0,00	33	**0,00**	5,51	2,46	9,07	0,00	7	**0,00**	5,83	2,54	8,56	0,00	5	**0,00**	5,18	3,00	2,37	0,85
**PA2453**	8735	7,90	76,66	3	31,50	0,73	-0,45	-1,67	0,09	387	**0,53**	0,62	-0,68	-2,65	0,01	215	**0,08**	0,73	-0,45	-1,58	0,11	427	**0,55**	0,70	3,00	-0,52	0,06
**PA2462**	598083	5,31	1169,68	38	7,33	1,13	0,17	0,53	0,60	1314	**0,99**	1,17	0,23	0,74	0,46	1041	**0,95**	1,30	0,38	1,23	0,22	586	**0,77**	1,20	3,00	0,26	0,09
**PA2464**	19200	4,86	284,11	5	23,10	0,99	-0,02	-0,16	0,88	1927	**0,99**	0,87	-0,20	-0,85	0,39	916	**0,93**	1,05	0,07	0,19	0,85	1745	**1,00**	0,97	3,00	-0,05	0,09
**PA2476**	30145	6,54	196,19	6	19,18	0,81	-0,30	-1,14	0,26	675	**0,82**	0,77	-0,37	-1,49	0,14	502	**0,59**	0,85	-0,23	-0,83	0,40	902	**0,92**	0,81	3,00	-0,30	0,04
**PA2481**	33189	8,56	125,34	5	16,90	1,05	0,07	0,16	0,87	1925	**0,99**	1,01	0,01	-0,05	0,96	2100	**0,99**	1,10	0,13	0,39	0,70	1427	**1,00**	1,05	3,00	0,07	0,04
**PA2482**	23705	6,29	108,53	3	20,30	0,95	-0,07	-0,34	0,74	1617	**0,99**	1,04	0,05	0,10	0,92	2014	**0,99**	1,12	0,17	0,51	0,61	1268	**0,99**	1,04	3,00	0,05	0,09
**PA2483**	37134	5,84	196,41	4	12,90	0,95	-0,08	-0,36	0,72	1589	**0,98**	1,04	0,05	0,10	0,92	2016	**0,99**	1,10	0,14	0,42	0,67	1384	**1,00**	1,03	3,00	0,04	0,08
**PA2491**	39056	5,37	236,29	8	22,40	0,78	-0,37	-1,37	0,17	523	**0,71**	0,79	-0,34	-1,38	0,17	539	**0,68**	0,92	-0,12	-0,45	0,65	1344	**1,00**	0,83	3,00	-0,27	0,08
**PA2503**	46864	4,86	174,00	4	14,25	1,17	0,23	0,72	0,47	1057	**0,97**	0,97	-0,04	-0,26	0,80	1761	**0,98**	0,75	-0,41	-1,44	0,15	497	**0,62**	0,97	3,00	-0,05	0,21
**PA2525**	55755	5,33	127,43	5	14,36	1,39	0,47	1,57	0,12	427	**0,59**	1,09	0,13	0,39	0,70	1553	**0,97**	0,87	-0,19	-0,72	0,47	1043	**0,93**	1,12	3,00	0,16	0,26
**PA2528**	48419	8,92	182,60	5	17,54	1,12	0,16	0,47	0,64	1406	**0,98**	1,01	0,01	-0,05	0,96	2087	**0,99**	0,97	-0,04	-0,19	0,85	1760	**1,00**	1,03	3,00	0,05	0,07
**PA2529**	54366	5,56	325,80	7	18,56	0,95	-0,07	-0,33	0,74	1626	**0,99**	0,89	-0,16	-0,70	0,48	1088	**0,96**	0,91	-0,14	-0,53	0,60	1246	**0,99**	0,92	3,00	-0,12	0,03
**PA2530**	49408	5,61	200,05	5	21,18	0,93	-0,11	-0,45	0,65	1434	**0,98**	0,84	-0,26	-1,06	0,29	738	**0,84**	1,30	0,38	1,22	0,22	597	**0,77**	1,02	3,00	0,03	0,24
**PA2532**	18658	5,16	1037,32	16	80,90	1,15	0,20	0,61	0,54	1187	**0,99**	1,13	0,18	0,56	0,58	1292	**0,96**	1,20	0,26	0,81	0,42	926	**0,92**	1,16	3,00	0,21	0,03
**PA2537**	24477	6,92	183,08	5	31,92	1,09	0,12	0,34	0,73	1610	**0,99**	1,18	0,24	0,79	0,43	990	**0,94**	1,12	0,16	0,49	0,62	1285	**1,00**	1,13	3,00	0,17	0,05
**PA2540**	67754	8,39	358,40	9	18,23	1,15	0,20	0,61	0,54	1203	**0,98**	1,06	0,08	0,20	0,84	1855	**0,98**	1,08	0,11	0,30	0,76	1573	**1,00**	1,09	3,00	0,13	0,05
**PA2545**	33093	6,01	227,52	10	31,28	0,94	-0,09	-0,39	0,69	1529	**0,99**	0,87	-0,20	-0,83	0,41	936	**0,94**	0,78	-0,35	-1,26	0,21	574	**0,75**	0,86	3,00	-0,21	0,08
**PA2551**	36944	9,48	430,07	10	28,54	1,11	0,15	0,44	0,66	1450	**0,99**	1,13	0,17	0,55	0,58	1304	**0,97**	1,02	0,03	0,04	0,97	1999	**1,00**	1,09	3,00	0,12	0,06
**PA2552**	42789	5,54	191,79	4	10,70	3,54	1,82	6,32	0,00	43	**0,00**	4,67	2,22	8,19	0,00	20	**0,00**	1,64	0,72	2,38	0,02	244	**0,15**	3,29	3,00	1,72	1,53
**PA2553**	43235	5,74	954,93	20	41,76	3,84	1,94	6,73	0,00	39	**0,00**	4,70	2,23	8,22	0,00	21	**0,00**	6,87	2,78	9,37	0,00	14	**0,00**	5,14	3,00	2,36	1,56

**PA2554**	27288	6,23	399,65	10	57,65	3,12	1,64	5,68	0,00	61	**0,00**	1,68	0,75	2,69	0,01	211	**0,07**	2,49	1,31	4,40	0,00	100	**0,00**	2,43	3,00	1,28	0,72
**PA2555**	62507	5,82	84,74	3	4,63	2,12	1,08	3,72	0,00	107	**0,00**	2,49	1,31	4,80	0,00	90	**0,00**	2,75	1,46	4,88	0,00	89	**0,00**	2,45	3,00	1,29	0,32
**PA2575**	24226	5,96	567,00	14	61,64	0,98	-0,03	-0,20	0,85	1857	**0,99**	0,99	-0,02	-0,17	0,87	1917	**0,98**	1,21	0,28	0,88	0,38	860	**0,91**	1,06	3,00	0,08	0,13
**PA2579**	34634	6,06	192,67	7	20,54	1,32	0,40	1,32	0,19	551	**0,74**	1,18	0,24	0,80	0,42	974	**0,94**	1,21	0,28	0,89	0,37	842	**0,91**	1,24	3,00	0,31	0,07
**PA2581**	41678	8,82	190,45	4	19,98	1,03	0,04	0,06	0,96	2086	**1,00**	0,91	-0,14	-0,61	0,54	1208	**0,97**	1,15	0,20	0,62	0,54	1157	**0,96**	1,03	3,00	0,04	0,12
**PA2582**	21255	9,82	284,36	7	50,19	0,95	-0,07	-0,33	0,74	1631	**0,99**	0,85	-0,23	-0,96	0,34	824	**0,89**	0,84	-0,26	-0,93	0,35	814	**0,89**	0,88	3,00	-0,18	0,06
**PA2587**	42959	6,65	196,87	5	11,60	0,75	-0,42	-1,56	0,12	438	**0,59**	0,64	-0,64	-2,49	0,01	235	**0,12**	0,82	-0,28	-1,00	0,32	758	**0,86**	0,74	3,00	-0,44	0,09
**PA2592**	43576	6,25	112,65	5	20,46	0,82	-0,29	-1,09	0,28	707	**0,85**	0,92	-0,12	-0,56	0,57	1290	**0,96**	0,87	-0,21	-0,76	0,44	979	**0,94**	0,87	3,00	-0,21	0,05
**PA2607**	11327	4,60	97,29	2	14,90	0,91	-0,13	-0,55	0,58	1283	**0,98**	1,10	0,14	0,42	0,67	1502	**0,97**	1,28	0,36	1,17	0,24	630	**0,80**	1,10	3,00	0,14	0,19
**PA2611**	51867	5,90	470,94	10	22,45	1,06	0,08	0,19	0,85	1864	**0,99**	0,92	-0,12	-0,53	0,60	1329	**0,97**	1,10	0,14	0,42	0,68	1389	**1,00**	1,03	3,00	0,04	0,09
**PA2612**	49980	5,39	959,51	22	37,56	0,98	-0,03	-0,19	0,85	1872	**0,99**	1,07	0,10	0,29	0,77	1707	**0,98**	0,98	-0,03	-0,15	0,88	1815	**1,00**	1,01	3,00	0,02	0,05
**PA2613**	51893	5,96	251,92	9	25,54	1,05	0,07	0,18	0,86	1887	**0,99**	1,15	0,20	0,65	0,51	1152	**0,96**	1,00	0,00	-0,07	0,94	1945	**1,00**	1,07	3,00	0,09	0,08
**PA2614**	24931	5,76	910,22	21	62,97	1,08	0,12	0,33	0,74	1630	**0,99**	1,15	0,20	0,64	0,53	1184	**0,96**	1,15	0,20	0,63	0,53	1136	**0,95**	1,13	3,00	0,17	0,04
**PA2615**	94662	6,41	366,30	12	15,73	0,93	-0,11	-0,47	0,64	1408	**0,99**	0,96	-0,06	-0,30	0,76	1677	**0,98**	1,10	0,14	0,42	0,68	1386	**1,00**	1,00	3,00	0,00	0,09
**PA2616**	35900	5,24	528,18	7	35,40	0,86	-0,21	-0,82	0,41	961	**0,94**	0,94	-0,09	-0,44	0,66	1482	**0,96**	0,81	-0,30	-1,07	0,29	693	**0,85**	0,87	3,00	-0,20	0,06
**PA2619**	9162	9,15	361,05	9	77,80	1,10	0,13	0,38	0,70	1548	**0,98**	0,96	-0,06	-0,33	0,74	1636	**0,98**	1,05	0,07	0,16	0,87	1790	**1,00**	1,03	3,00	0,05	0,07
**PA2620**	90293	5,69	1030,63	28	29,80	0,97	-0,04	-0,22	0,82	1804	**0,99**	0,97	-0,04	-0,24	0,81	1789	**0,98**	1,06	0,08	0,21	0,83	1706	**1,00**	1,00	3,00	0,00	0,05
**PA2623**	50298	5,10	2589,39	60	71,40	0,66	-0,61	-2,22	0,03	250	**0,23**	0,72	-0,47	-1,86	0,06	370	**0,37**	0,71	-0,49	-1,72	0,09	370	**0,48**	0,70	3,00	-0,52	0,04
**PA2624**	89404	5,81	4330,57	95	76,11	0,76	-0,40	-1,49	0,14	467	**0,64**	0,79	-0,35	-1,40	0,16	530	**0,66**	0,79	-0,34	-1,22	0,22	599	**0,77**	0,78	3,00	-0,36	0,02
**PA2626**	44874	5,73	292,24	8	26,10	0,97	-0,04	-0,21	0,83	1817	**0,99**	1,03	0,04	0,06	0,95	2070	**0,99**	1,06	0,09	0,23	0,82	1672	**1,00**	1,02	3,00	0,03	0,04
**PA2627**	23783	10,35	388,35	8	38,10	0,98	-0,03	-0,18	0,86	1890	**0,99**	1,15	0,20	0,66	0,51	1139	**0,96**	0,99	-0,01	-0,10	0,92	1890	**1,00**	1,04	3,00	0,06	0,10
**PA2629**	53876	5,59	1487,74	36	59,67	0,95	-0,07	-0,34	0,73	1612	**0,99**	0,92	-0,12	-0,54	0,59	1316	**0,97**	0,91	-0,13	-0,50	0,61	1272	**1,00**	0,93	3,00	-0,11	0,02
**PA2630**	45321	4,42	408,65	9	19,09	0,86	-0,21	-0,82	0,41	962	**0,94**	0,83	-0,27	-1,09	0,28	717	**0,83**	0,84	-0,25	-0,89	0,37	843	**0,91**	0,85	3,00	-0,24	0,02
**PA2631**	17039	5,04	217,62	4	29,45	0,90	-0,16	-0,64	0,52	1142	**0,99**	0,95	-0,07	-0,36	0,72	1593	**0,97**	1,05	0,07	0,18	0,86	1770	**1,00**	0,97	3,00	-0,05	0,08
**PA2634**	64693	5,68	1236,94	34	38,80	0,79	-0,34	-1,28	0,20	575	**0,75**	0,91	-0,13	-0,58	0,56	1253	**0,97**	0,82	-0,29	-1,04	0,30	716	**0,85**	0,84	3,00	-0,25	0,07
**PA2637**	16009	9,98	58,23	2	20,40	1,03	0,04	0,07	0,94	2054	**1,00**	1,09	0,12	0,36	0,72	1602	**0,97**	0,73	-0,46	-1,61	0,11	414	**0,54**	0,95	3,00	-0,08	0,19
**PA2638**	27080	5,31	501,35	14	38,97	0,98	-0,03	-0,20	0,85	1858	**0,99**	0,98	-0,04	-0,23	0,82	1806	**0,98**	0,92	-0,12	-0,45	0,65	1339	**1,00**	0,96	3,00	-0,06	0,03
**PA2639**	71634	6,22	1323,91	38	53,93	0,98	-0,03	-0,19	0,85	1871	**0,99**	1,02	0,02	-0,01	0,99	2143	**1,00**	0,97	-0,04	-0,20	0,84	1727	**1,00**	0,99	3,00	-0,02	0,02
**PA2640**	19172	5,01	394,58	9	46,80	0,97	-0,04	-0,23	0,81	1788	**0,99**	0,97	-0,04	-0,23	0,82	1804	**0,98**	0,99	-0,01	-0,10	0,92	1902	**1,00**	0,98	3,00	-0,03	0,01
**PA2641**	52186	6,57	1165,22	32	59,09	1,02	0,03	0,04	0,97	2120	**0,99**	0,98	-0,02	-0,19	0,85	1874	**0,98**	1,09	0,12	0,35	0,73	1497	**1,00**	1,03	3,00	0,04	0,05
**PA2642**	104619	5,71	3062,73	56	50,28	0,98	-0,03	-0,19	0,85	1873	**0,99**	1,01	0,01	-0,05	0,96	2099	**0,99**	0,97	-0,04	-0,19	0,85	1758	**1,00**	0,99	3,00	-0,02	0,02
**PA2643**	38154	6,41	126,76	4	11,95	0,89	-0,16	-0,65	0,52	1139	**0,99**	0,98	-0,03	-0,20	0,84	1857	**0,98**	1,17	0,22	0,70	0,48	1052	**0,94**	1,01	3,00	0,02	0,14
**PA2644**	22419	6,36	465,67	13	52,42	0,98	-0,03	-0,20	0,84	1853	**0,99**	1,12	0,16	0,51	0,61	1372	**0,96**	1,05	0,08	0,20	0,84	1724	**1,00**	1,05	3,00	0,07	0,07
**PA2647**	68531	8,24	90,51	2	3,70	1,01	0,01	-0,03	0,97	2124	**1,00**	0,87	-0,20	-0,85	0,40	920	**0,93**	1,03	0,04	0,08	0,93	1928	**1,00**	0,97	3,00	-0,05	0,09
**PA2652**	62613	5,14	750,35	14	27,41	0,74	-0,43	-1,58	0,11	424	**0,58**	0,75	-0,41	-1,64	0,10	441	**0,50**	0,65	-0,62	-2,16	0,03	277	**0,23**	0,71	3,00	-0,48	0,06
**PA2654**	79954	4,94	758,84	14	22,55	0,93	-0,10	-0,43	0,66	1461	**0,99**	0,91	-0,14	-0,63	0,53	1187	**0,96**	0,88	-0,18	-0,67	0,51	1098	**0,95**	0,91	3,00	-0,14	0,02
**PA2660**	41551	9,62	365,05	11	24,30	0,88	-0,19	-0,74	0,46	1028	**0,97**	0,87	-0,21	-0,87	0,39	899	**0,93**	0,79	-0,34	-1,22	0,22	600	**0,77**	0,84	3,00	-0,24	0,05
**PA2665**	58840	6,31	68,05	3	4,40	0,72	-0,48	-1,75	0,08	358	**0,49**	1,06	0,09	0,22	0,82	1818	**0,98**	1,04	0,06	0,15	0,88	1812	**1,00**	0,94	3,00	-0,09	0,19
**PA2667**	15677	9,45	512,27	12	42,33	1,01	0,01	-0,04	0,97	2106	**1,00**	1,10	0,14	0,42	0,68	1511	**0,97**	1,14	0,19	0,58	0,56	1204	**0,97**	1,08	3,00	0,11	0,07
**PA2684**	149454	6,17	101,00	3	2,27	1,11	0,15	0,46	0,64	1424	**0,98**	0,95	-0,07	-0,37	0,71	1589	**0,97**	0,90	-0,16	-0,60	0,55	1178	**0,96**	0,99	3,00	-0,02	0,11
**PA2691**	44160	5,10	231,05	6	15,00	1,07	0,10	0,28	0,78	1715	**0,98**	0,80	-0,31	-1,27	0,20	597	**0,74**	1,45	0,53	1,76	0,08	360	**0,45**	1,11	3,00	0,15	0,32
**PA2692**	20179	6,19	160,14	5	29,12	1,04	0,05	0,11	0,92	1988	**1,00**	0,80	-0,32	-1,27	0,20	591	**0,74**	0,82	-0,29	-1,03	0,30	726	**0,85**	0,89	3,00	-0,17	0,13
**PA2705**	50547	6,59	118,32	4	7,48	0,94	-0,09	-0,39	0,70	1537	**0,99**	1,12	0,17	0,52	0,60	1342	**0,97**	0,81	-0,30	-1,06	0,29	700	**0,85**	0,96	3,00	-0,06	0,15
**PA2707**	35978	5,86	412,76	11	34,94	0,77	-0,38	-1,40	0,16	510	**0,69**	0,88	-0,19	-0,79	0,43	992	**0,94**	0,86	-0,21	-0,78	0,43	955	**0,94**	0,84	3,00	-0,26	0,06
**PA2709**	37262	6,24	696,67	13	42,93	0,78	-0,36	-1,34	0,18	538	**0,73**	0,82	-0,29	-1,17	0,24	646	**0,81**	0,75	-0,41	-1,44	0,15	502	**0,62**	0,78	3,00	-0,35	0,03
**PA2725**	71657	5,93	282,47	9	16,34	0,82	-0,28	-1,08	0,28	713	**0,86**	0,82	-0,29	-1,16	0,24	649	**0,81**	0,71	-0,50	-1,76	0,08	359	**0,45**	0,78	3,00	-0,35	0,07
**PA2727**	138348	6,50	170,87	5	4,42	1,12	0,16	0,48	0,63	1395	**0,99**	1,19	0,25	0,84	0,40	932	**0,93**	0,84	-0,25	-0,90	0,37	837	**0,91**	1,05	3,00	0,07	0,18
**PA2732**	139486	5,86	386,65	16	13,14	0,78	-0,36	-1,34	0,18	539	**0,73**	0,97	-0,04	-0,24	0,81	1790	**0,98**	0,72	-0,47	-1,64	0,10	396	**0,52**	0,83	3,00	-0,28	0,13

**PA2734**	52460	5,87	610,42	17	28,59	0,81	-0,30	-1,12	0,26	683	**0,84**	0,84	-0,25	-1,04	0,30	757	**0,85**	0,86	-0,21	-0,77	0,44	975	**0,93**	0,84	3,00	-0,25	0,02
**PA2735**	98588	5,46	1591,11	46	42,66	0,85	-0,23	-0,87	0,38	895	**0,93**	0,83	-0,27	-1,09	0,28	718	**0,83**	0,86	-0,21	-0,77	0,44	971	**0,93**	0,85	3,00	-0,23	0,02
**PA2738**	12905	9,57	76,87	3	25,33	0,64	-0,64	-2,32	0,02	228	**0,19**	0,92	-0,11	-0,52	0,60	1345	**0,97**	0,81	-0,31	-1,11	0,27	660	**0,83**	0,79	3,00	-0,34	0,14
**PA2739**	90625	5,26	2258,82	47	51,83	0,95	-0,07	-0,34	0,74	1618	**0,99**	0,90	-0,15	-0,65	0,51	1148	**0,97**	0,89	-0,18	-0,65	0,52	1113	**0,95**	0,91	3,00	-0,13	0,03
**PA2740**	40136	5,62	921,24	24	52,34	0,92	-0,13	-0,52	0,60	1327	**0,99**	0,91	-0,14	-0,62	0,54	1204	**0,96**	0,88	-0,19	-0,69	0,49	1072	**0,95**	0,90	3,00	-0,15	0,02
**PA2741**	15518	11,74	326,74	11	44,77	0,94	-0,08	-0,37	0,71	1569	**0,98**	0,94	-0,08	-0,41	0,68	1522	**0,97**	1,00	0,00	-0,04	0,97	1990	**1,00**	0,96	3,00	-0,05	0,03
**PA2743**	24212	9,60	1248,30	28	63,40	1,03	0,04	0,06	0,95	2080	**1,00**	1,04	0,05	0,09	0,93	2023	**0,99**	1,02	0,03	0,03	0,97	2005	**1,00**	1,03	3,00	0,04	0,01
**PA2744**	78635	5,97	1206,60	32	49,00	0,90	-0,16	-0,64	0,52	1153	**0,99**	0,88	-0,18	-0,76	0,45	1030	**0,94**	0,86	-0,21	-0,78	0,44	960	**0,93**	0,88	3,00	-0,18	0,02
**PA2745**	31573	5,41	169,23	5	16,40	0,81	-0,31	-1,18	0,24	643	**0,81**	0,88	-0,19	-0,80	0,42	976	**0,94**	0,84	-0,25	-0,90	0,37	834	**0,91**	0,84	3,00	-0,25	0,04
**PA2755**	19516	6,17	186,32	6	28,55	0,69	-0,54	-1,96	0,05	286	**0,38**	0,71	-0,50	-1,96	0,05	338	**0,32**	0,76	-0,39	-1,39	0,16	524	**0,64**	0,72	3,00	-0,48	0,04
**PA2760**	50182	5,54	1091,33	22	36,95	1,59	0,67	2,27	0,02	238	**0,21**	1,77	0,82	2,98	0,00	180	**0,04**	2,21	1,14	3,82	0,00	120	**0,00**	1,86	3,00	0,89	0,32
**PA2765**	33477	4,70	554,25	13	28,37	0,76	-0,39	-1,46	0,14	475	**0,66**	0,76	-0,39	-1,56	0,12	469	**0,55**	0,79	-0,35	-1,23	0,22	588	**0,77**	0,77	3,00	-0,38	0,01
**PA2770**	29767	4,96	180,77	5	19,70	0,67	-0,58	-2,11	0,03	262	**0,29**	0,72	-0,47	-1,84	0,07	376	**0,37**	0,59	-0,76	-2,63	0,01	213	**0,08**	0,66	3,00	-0,60	0,07
**PA2774**	19969	9,57	92,96	3	11,10	0,76	-0,40	-1,49	0,14	468	**0,64**	1,06	0,09	0,23	0,81	1803	**0,98**	0,96	-0,05	-0,24	0,81	1663	**1,00**	0,93	3,00	-0,11	0,16
**PA2776**	49822	6,80	473,22	12	22,27	1,20	0,26	0,85	0,40	929	**0,93**	1,15	0,20	0,64	0,52	1169	**0,96**	1,11	0,14	0,43	0,67	1369	**1,00**	1,15	3,00	0,20	0,05
**PA2793**	41704	8,39	118,41	4	15,50	0,79	-0,34	-1,28	0,20	576	**0,75**	0,88	-0,18	-0,78	0,43	996	**0,94**	0,89	-0,17	-0,63	0,53	1140	**0,96**	0,85	3,00	-0,23	0,06
**PA2795**	38473	6,57	56,03	2	7,05	0,98	-0,02	-0,16	0,87	1926	**0,99**	0,83	-0,26	-1,07	0,29	733	**0,84**	0,79	-0,34	-1,20	0,23	612	**0,78**	0,87	3,00	-0,20	0,10
**PA2796**	35937	5,15	605,13	16	45,71	0,73	-0,45	-1,67	0,09	388	**0,53**	0,84	-0,25	-1,02	0,31	773	**0,85**	0,82	-0,29	-1,05	0,29	711	**0,85**	0,80	3,00	-0,33	0,06
**PA2797**	18846	4,87	126,16	2	10,00	0,91	-0,13	-0,54	0,59	1288	**0,99**	1,13	0,17	0,55	0,58	1306	**0,97**	0,86	-0,22	-0,81	0,42	927	**0,93**	0,97	3,00	-0,05	0,14
**PA2798**	45234	4,86	684,16	11	26,01	0,90	-0,15	-0,60	0,55	1208	**0,98**	0,92	-0,13	-0,57	0,57	1269	**0,97**	1,06	0,08	0,22	0,83	1700	**1,00**	0,96	3,00	-0,06	0,09
**PA2800**	27763	5,41	298,63	7	26,61	0,87	-0,20	-0,77	0,44	1001	**0,95**	0,79	-0,35	-1,39	0,16	532	**0,66**	0,91	-0,13	-0,51	0,61	1261	**0,99**	0,86	3,00	-0,22	0,06
**PA2805**	10130	4,50	84,38	2	27,60	0,99	-0,01	-0,12	0,90	1974	**0,99**	0,82	-0,28	-1,15	0,25	663	**0,81**	1,15	0,20	0,61	0,54	1169	**0,96**	0,99	3,00	-0,02	0,16
**PA2806**	32212	5,48	370,05	10	35,03	0,97	-0,04	-0,21	0,83	1818	**0,99**	0,83	-0,27	-1,11	0,27	706	**0,82**	0,96	-0,06	-0,25	0,80	1656	**1,00**	0,92	3,00	-0,12	0,08
**PA2812**	36917	5,93	219,13	6	19,00	0,77	-0,37	-1,39	0,16	515	**0,69**	0,83	-0,27	-1,12	0,26	692	**0,82**	0,88	-0,18	-0,67	0,50	1095	**0,95**	0,83	3,00	-0,27	0,06
**PA2817**	16460	4,64	229,40	5	29,72	0,78	-0,37	-1,36	0,17	530	**0,71**	0,78	-0,36	-1,43	0,15	521	**0,64**	0,79	-0,35	-1,24	0,22	585	**0,76**	0,78	3,00	-0,36	0,00
**PA2823**	35128	5,59	244,52	8	32,80	0,81	-0,31	-1,15	0,25	661	**0,82**	0,90	-0,16	-0,68	0,50	1124	**0,96**	0,87	-0,21	-0,75	0,45	995	**0,93**	0,86	3,00	-0,22	0,05
**PA2826**	19850	8,33	174,89	4	24,70	0,83	-0,27	-1,04	0,30	743	**0,87**	1,01	0,01	-0,05	0,96	2098	**0,99**	0,84	-0,25	-0,89	0,37	845	**0,91**	0,89	3,00	-0,16	0,10
**PA2828**	47929	5,81	770,20	15	25,91	0,97	-0,04	-0,23	0,81	1789	**0,99**	1,11	0,15	0,47	0,64	1430	**0,96**	0,95	-0,07	-0,29	0,77	1591	**1,00**	1,01	3,00	0,02	0,09
**PA2830**	33060	7,03	412,74	8	22,35	1,06	0,09	0,23	0,82	1799	**0,99**	1,11	0,15	0,44	0,66	1469	**0,97**	1,03	0,04	0,08	0,93	1927	**1,00**	1,07	3,00	0,09	0,04
**PA2831**	44099	5,34	371,44	12	29,85	0,71	-0,50	-1,82	0,07	330	**0,45**	0,76	-0,39	-1,54	0,12	485	**0,55**	0,69	-0,53	-1,84	0,07	334	**0,41**	0,72	3,00	-0,47	0,04
**PA2840**	65273	9,00	1205,86	28	44,35	0,88	-0,18	-0,70	0,48	1085	**0,97**	0,70	-0,51	-1,99	0,05	329	**0,31**	0,75	-0,41	-1,45	0,15	491	**0,61**	0,78	3,00	-0,36	0,09
**PA2843**	51825	5,76	216,19	5	12,90	0,99	-0,02	-0,15	0,88	1942	**0,99**	0,82	-0,28	-1,14	0,25	671	**0,81**	0,81	-0,31	-1,12	0,26	655	**0,83**	0,87	3,00	-0,20	0,10
**PA2849**	17932	5,56	154,99	4	17,55	1,22	0,29	0,93	0,35	846	**0,91**	1,22	0,29	0,97	0,33	809	**0,88**	1,36	0,44	1,45	0,15	494	**0,62**	1,27	3,00	0,34	0,08
**PA2850**	15470	6,71	629,34	10	61,30	3,13	1,65	5,70	0,00	60	**0,00**	3,07	1,62	5,93	0,00	66	**0,00**	2,96	1,57	5,25	0,00	77	**0,00**	3,05	3,00	1,61	0,08
**PA2851**	23369	4,82	627,21	14	34,60	0,86	-0,23	-0,87	0,38	899	**0,93**	0,83	-0,27	-1,12	0,26	697	**0,82**	0,86	-0,22	-0,79	0,43	950	**0,94**	0,85	3,00	-0,24	0,02
**PA2853**	9884	7,90	259,86	6	25,30	0,90	-0,15	-0,61	0,54	1191	**0,99**	0,95	-0,07	-0,36	0,72	1608	**0,97**	0,99	-0,01	-0,09	0,93	1909	**1,00**	0,95	3,00	-0,08	0,05
**PA2854**	36841	5,49	604,43	13	49,43	0,98	-0,02	-0,17	0,87	1911	**0,99**	0,96	-0,06	-0,30	0,76	1678	**0,98**	0,87	-0,19	-0,71	0,48	1048	**0,94**	0,94	3,00	-0,09	0,06
**PA2856**	22079	5,83	136,82	4	31,45	0,84	-0,25	-0,97	0,33	799	**0,90**	1,05	0,07	0,15	0,88	1940	**0,98**	0,89	-0,18	-0,65	0,51	1111	**0,95**	0,92	3,00	-0,11	0,11
**PA2857**	25192	5,56	164,35	3	21,60	0,99	-0,01	-0,13	0,89	1963	**0,99**	1,08	0,11	0,31	0,76	1667	**0,98**	1,01	0,02	0,00	1,00	2061	**1,00**	1,03	3,00	0,04	0,05
**PA2859**	20141	6,19	183,07	6	29,57	1,05	0,08	0,19	0,85	1870	**0,99**	1,02	0,03	0,03	0,98	2121	**1,00**	1,01	0,01	-0,03	0,98	2023	**1,00**	1,03	3,00	0,04	0,02
**PA2866**	30287	5,78	45,52	2	15,70	0,93	-0,10	-0,43	0,67	1470	**0,99**	0,91	-0,14	-0,61	0,54	1210	**0,97**	0,94	-0,09	-0,37	0,71	1463	**1,00**	0,93	3,00	-0,11	0,02
**PA2867**	52912	4,93	647,53	12	28,95	1,24	0,31	0,99	0,32	783	**0,89**	1,19	0,25	0,82	0,41	951	**0,94**	1,02	0,03	0,03	0,98	2015	**1,00**	1,15	3,00	0,20	0,11
**PA2871**	29299	5,51	302,81	8	28,15	0,91	-0,14	-0,57	0,57	1262	**0,98**	0,92	-0,11	-0,53	0,60	1336	**0,97**	0,82	-0,29	-1,02	0,31	738	**0,85**	0,88	3,00	-0,18	0,06
**PA2875**	33931	5,58	163,09	3	17,00	0,76	-0,39	-1,46	0,14	479	**0,65**	0,49	-1,03	-3,94	0,00	120	**0,00**	0,76	-0,39	-1,39	0,17	528	**0,65**	0,67	3,00	-0,58	0,16
**PA2876**	26096	5,44	324,13	10	44,76	0,98	-0,03	-0,18	0,85	1878	**0,99**	0,91	-0,13	-0,58	0,56	1244	**0,97**	0,92	-0,12	-0,47	0,64	1304	**1,00**	0,94	3,00	-0,09	0,04
**PA2885**	23000	5,16	168,54	4	17,20	0,98	-0,04	-0,21	0,84	1839	**0,99**	1,01	0,01	-0,05	0,96	2078	**1,00**	0,98	-0,03	-0,17	0,87	1788	**1,00**	0,99	3,00	-0,02	0,02
**PA2891**	74233	5,83	51,20	2	2,30	1,08	0,11	0,31	0,76	1660	**0,99**	0,94	-0,09	-0,42	0,67	1506	**0,97**	0,88	-0,19	-0,69	0,49	1074	**0,94**	0,97	3,00	-0,05	0,10
**PA2897**	55603	9,31	137,08	3	5,37	1,00	0,00	-0,09	0,93	2020	**1,00**	1,29	0,37	1,28	0,20	580	**0,75**	1,19	0,25	0,80	0,43	936	**0,94**	1,16	3,00	0,21	0,15

**PA2900**	31368	6,23	450,54	10	39,02	0,88	-0,18	-0,72	0,47	1059	**0,97**	0,93	-0,11	-0,50	0,62	1393	**0,96**	0,72	-0,48	-1,67	0,09	387	**0,50**	0,84	3,00	-0,25	0,11
**PA2901**	14671	5,58	177,09	4	29,65	0,72	-0,47	-1,72	0,09	368	**0,50**	1,01	0,02	-0,02	0,98	2138	**1,00**	0,93	-0,10	-0,40	0,69	1417	**1,00**	0,89	3,00	-0,17	0,15
**PA2902**	32342	5,00	98,37	4	17,98	0,83	-0,27	-1,01	0,31	766	**0,88**	1,00	-0,01	-0,12	0,91	1989	**0,98**	1,07	0,09	0,26	0,79	1631	**1,00**	0,97	3,00	-0,05	0,12
**PA2903**	59767	5,78	426,17	10	19,75	1,00	0,01	-0,06	0,95	2083	**1,00**	1,13	0,18	0,57	0,57	1267	**0,97**	1,04	0,06	0,14	0,89	1821	**1,00**	1,06	3,00	0,08	0,07
**PA2904**	28836	6,02	231,75	6	38,67	1,06	0,08	0,21	0,83	1830	**0,99**	1,10	0,14	0,44	0,66	1484	**0,96**	1,13	0,17	0,53	0,60	1247	**0,99**	1,10	3,00	0,13	0,03
**PA2905**	22351	5,68	268,34	7	26,99	1,06	0,09	0,22	0,82	1807	**0,99**	1,12	0,17	0,52	0,60	1349	**0,97**	1,00	0,01	-0,03	0,97	2003	**1,00**	1,06	3,00	0,09	0,06
**PA2907**	45157	8,20	130,93	4	7,25	1,01	0,01	-0,05	0,96	2094	**0,99**	0,87	-0,20	-0,85	0,40	921	**0,93**	0,95	-0,07	-0,30	0,77	1585	**1,00**	0,94	3,00	-0,09	0,07
**PA2944**	141502	5,35	186,80	6	6,50	1,01	0,01	-0,05	0,96	2097	**1,00**	0,97	-0,05	-0,28	0,78	1721	**0,98**	0,99	-0,01	-0,09	0,93	1914	**1,00**	0,99	3,00	-0,02	0,02
**PA2945**	43200	5,10	531,20	11	28,44	1,00	0,00	-0,09	0,93	2017	**1,00**	0,88	-0,19	-0,81	0,42	962	**0,94**	0,95	-0,07	-0,29	0,77	1594	**1,00**	0,94	3,00	-0,09	0,06
**PA2949**	36302	6,09	135,03	4	13,20	1,05	0,07	0,16	0,88	1929	**0,99**	0,84	-0,25	-1,05	0,30	755	**0,85**	0,96	-0,05	-0,23	0,82	1677	**1,00**	0,95	3,00	-0,07	0,11
**PA2950**	47818	5,01	1282,05	37	52,29	0,85	-0,24	-0,92	0,36	849	**0,91**	0,91	-0,13	-0,58	0,56	1261	**0,97**	0,85	-0,24	-0,87	0,38	865	**0,91**	0,87	3,00	-0,20	0,04
**PA2951**	33997	4,98	2113,91	30	75,88	0,84	-0,24	-0,94	0,35	831	**0,91**	0,90	-0,15	-0,66	0,51	1138	**0,97**	0,89	-0,16	-0,61	0,54	1164	**0,96**	0,88	3,00	-0,19	0,03
**PA2952**	30009	9,01	1765,07	32	71,90	0,81	-0,31	-1,15	0,25	662	**0,82**	0,89	-0,16	-0,71	0,48	1082	**0,96**	0,89	-0,17	-0,62	0,54	1150	**0,96**	0,86	3,00	-0,21	0,05
**PA2953**	65985	5,51	1694,59	40	50,08	0,76	-0,39	-1,45	0,15	488	**0,66**	0,78	-0,36	-1,44	0,15	517	**0,63**	0,75	-0,42	-1,46	0,14	485	**0,61**	0,76	3,00	-0,39	0,01
**PA2957**	27364	6,68	198,88	6	22,00	0,80	-0,32	-1,19	0,23	636	**0,80**	0,90	-0,15	-0,67	0,50	1126	**0,96**	0,88	-0,18	-0,67	0,50	1089	**0,95**	0,86	3,00	-0,22	0,05
**PA2959**	28857	5,32	162,49	5	19,22	0,98	-0,03	-0,19	0,85	1867	**0,99**	0,82	-0,28	-1,14	0,25	672	**0,81**	0,78	-0,36	-1,26	0,21	572	**0,75**	0,86	3,00	-0,22	0,10
**PA2960**	14337	9,47	144,57	2	19,50	0,78	-0,36	-1,34	0,18	535	**0,73**	0,63	-0,67	-2,59	0,01	221	**0,09**	0,78	-0,36	-1,28	0,20	564	**0,73**	0,73	3,00	-0,46	0,09
**PA2961**	38207	6,19	388,35	9	33,52	0,89	-0,17	-0,69	0,49	1097	**0,98**	1,02	0,03	0,03	0,98	2129	**0,99**	1,05	0,07	0,18	0,86	1763	**1,00**	0,99	3,00	-0,02	0,09
**PA2963**	41313	8,50	151,90	5	19,20	0,99	-0,01	-0,13	0,90	1972	**0,99**	0,89	-0,17	-0,73	0,46	1050	**0,95**	1,04	0,06	0,16	0,88	1802	**1,00**	0,97	3,00	-0,04	0,08
**PA2965**	44965	5,62	1035,13	18	35,57	0,80	-0,32	-1,19	0,23	633	**0,80**	0,96	-0,05	-0,30	0,77	1690	**0,98**	0,85	-0,23	-0,84	0,40	891	**0,93**	0,87	3,00	-0,20	0,08
**PA2966**	9600	4,07	273,09	6	37,15	1,19	0,25	0,81	0,42	968	**0,93**	0,96	-0,06	-0,33	0,74	1642	**0,98**	0,96	-0,06	-0,28	0,78	1611	**1,00**	1,04	3,00	0,05	0,14
**PA2967**	26866	6,17	1217,55	21	57,23	0,88	-0,19	-0,74	0,46	1029	**0,97**	0,82	-0,28	-1,14	0,25	669	**0,82**	0,83	-0,27	-0,98	0,32	771	**0,87**	0,84	3,00	-0,25	0,03
**PA2968**	33951	5,05	662,93	14	38,56	0,87	-0,20	-0,79	0,43	984	**0,94**	0,83	-0,27	-1,11	0,27	703	**0,82**	0,82	-0,29	-1,05	0,30	712	**0,86**	0,84	3,00	-0,26	0,03
**PA2970**	7652	9,69	335,83	7	47,40	1,16	0,21	0,67	0,50	1120	**0,98**	0,80	-0,33	-1,32	0,19	562	**0,72**	0,97	-0,04	-0,19	0,85	1757	**1,00**	0,98	3,00	-0,03	0,18
**PA2971**	20749	4,52	253,87	5	32,00	0,97	-0,04	-0,21	0,83	1826	**0,99**	1,01	0,02	-0,03	0,98	2119	**1,00**	1,05	0,07	0,17	0,87	1782	**1,00**	1,01	3,00	0,02	0,04
**PA2972**	21854	5,77	277,26	5	20,44	0,87	-0,20	-0,78	0,43	994	**0,95**	0,90	-0,15	-0,64	0,52	1172	**0,96**	0,94	-0,08	-0,34	0,74	1525	**1,00**	0,91	3,00	-0,14	0,04
**PA2973**	39643	7,82	392,08	9	21,23	0,98	-0,03	-0,20	0,85	1859	**0,99**	0,95	-0,08	-0,38	0,70	1562	**0,97**	1,03	0,04	0,07	0,95	1953	**1,00**	0,98	3,00	-0,02	0,04
**PA2974**	26164	5,41	130,25	3	18,43	0,93	-0,11	-0,46	0,65	1427	**0,98**	0,96	-0,06	-0,33	0,74	1639	**0,98**	1,03	0,04	0,08	0,94	1934	**1,00**	0,97	3,00	-0,04	0,05
**PA2975**	38501	9,91	334,61	10	30,50	1,03	0,05	0,09	0,93	2025	**1,00**	1,09	0,13	0,39	0,70	1551	**0,97**	0,99	-0,01	-0,11	0,91	1880	**1,00**	1,04	3,00	0,06	0,05
**PA2976**	121810	4,91	2343,68	54	33,05	0,92	-0,12	-0,52	0,61	1332	**0,99**	0,87	-0,21	-0,87	0,38	893	**0,93**	0,87	-0,20	-0,72	0,47	1034	**0,94**	0,89	3,00	-0,18	0,03
**PA2977**	39008	6,31	307,09	8	18,86	0,98	-0,03	-0,17	0,87	1905	**0,99**	0,90	-0,16	-0,68	0,49	1116	**0,96**	0,96	-0,07	-0,28	0,78	1610	**1,00**	0,95	3,00	-0,08	0,04
**PA2978**	17856	5,83	31,22	2	13,00	0,93	-0,11	-0,45	0,65	1435	**0,98**	1,07	0,10	0,26	0,79	1755	**0,98**	0,89	-0,17	-0,65	0,52	1118	**0,95**	0,96	3,00	-0,06	0,10
**PA2979**	28298	5,41	285,73	7	20,43	0,92	-0,12	-0,50	0,62	1355	**0,99**	0,89	-0,17	-0,71	0,47	1070	**0,96**	0,76	-0,40	-1,40	0,16	521	**0,64**	0,86	3,00	-0,22	0,09
**PA2980**	7627	4,69	244,50	7	66,50	0,94	-0,09	-0,40	0,69	1521	**0,99**	0,87	-0,20	-0,86	0,39	910	**0,93**	1,01	0,01	-0,01	0,99	2051	**1,00**	0,94	3,00	-0,09	0,07
**PA2982**	16683	5,59	99,68	3	13,27	0,83	-0,28	-1,05	0,29	738	**0,87**	0,83	-0,27	-1,11	0,27	708	**0,82**	0,77	-0,37	-1,31	0,19	556	**0,71**	0,81	3,00	-0,30	0,03
**PA2983**	24618	6,37	337,02	6	20,90	0,60	-0,75	-2,70	0,01	179	**0,08**	0,58	-0,79	-3,04	0,00	175	**0,03**	0,62	-0,68	-2,36	0,02	247	**0,15**	0,60	3,00	-0,74	0,02
**PA2986**	49579	9,69	117,73	3	6,50	1,26	0,34	1,10	0,27	702	**0,84**	1,18	0,23	0,77	0,44	1015	**0,94**	0,77	-0,38	-1,36	0,18	539	**0,67**	1,07	3,00	0,10	0,26
**PA2987**	25447	6,17	79,27	3	17,17	0,79	-0,33	-1,25	0,21	595	**0,77**	0,89	-0,17	-0,74	0,46	1045	**0,95**	0,94	-0,09	-0,35	0,73	1495	**1,00**	0,87	3,00	-0,19	0,08
**PA2988**	47688	9,07	130,23	4	11,63	0,96	-0,06	-0,27	0,78	1729	**0,99**	1,03	0,04	0,07	0,95	2067	**0,99**	1,14	0,19	0,58	0,56	1195	**0,96**	1,04	3,00	0,06	0,09
**PA2990**	28654	9,04	77,25	3	9,43	0,77	-0,37	-1,39	0,17	518	**0,69**	0,80	-0,32	-1,27	0,20	592	**0,74**	0,80	-0,32	-1,13	0,26	649	**0,82**	0,79	3,00	-0,33	0,02
**PA2991**	54051	6,23	1850,08	35	52,73	0,71	-0,49	-1,82	0,07	333	**0,45**	0,74	-0,43	-1,72	0,09	412	**0,45**	0,65	-0,63	-2,18	0,03	272	**0,22**	0,70	3,00	-0,52	0,05
**PA2993**	38780	5,41	87,65	3	8,70	1,12	0,16	0,48	0,63	1388	**0,99**	1,10	0,14	0,42	0,67	1505	**0,97**	1,10	0,13	0,39	0,69	1423	**1,00**	1,11	3,00	0,14	0,01
**PA2994**	48850	5,03	298,28	8	18,13	0,94	-0,09	-0,40	0,69	1523	**0,99**	0,98	-0,02	-0,19	0,85	1881	**0,98**	0,86	-0,22	-0,81	0,42	931	**0,93**	0,93	3,00	-0,11	0,06
**PA2997**	30547	5,67	678,09	13	53,89	0,90	-0,15	-0,61	0,54	1195	**0,99**	1,07	0,09	0,26	0,80	1760	**0,98**	0,94	-0,09	-0,38	0,71	1453	**1,00**	0,97	3,00	-0,05	0,09
**PA2999**	50973	7,13	875,77	19	42,39	0,88	-0,18	-0,71	0,48	1071	**0,97**	0,96	-0,05	-0,30	0,77	1686	**0,98**	0,89	-0,17	-0,64	0,52	1125	**0,96**	0,91	3,00	-0,13	0,05
**PA3001**	53866	7,74	2579,92	52	65,02	0,81	-0,30	-1,15	0,25	665	**0,82**	0,86	-0,21	-0,88	0,38	882	**0,93**	0,89	-0,17	-0,65	0,52	1116	**0,95**	0,85	3,00	-0,23	0,04
**PA3002**	134868	5,64	939,79	25	21,32	1,05	0,07	0,18	0,86	1894	**0,99**	1,07	0,09	0,25	0,81	1779	**0,98**	0,99	-0,01	-0,10	0,92	1898	**1,00**	1,04	3,00	0,05	0,04
**PA3003**	23371	4,74	308,81	6	26,83	1,00	0,00	-0,07	0,95	2061	**1,00**	1,04	0,06	0,11	0,91	2002	**0,98**	1,17	0,23	0,71	0,48	1051	**0,94**	1,07	3,00	0,10	0,09

**PA3004**	26964	6,14	383,07	8	32,63	0,94	-0,09	-0,39	0,69	1530	**0,99**	0,98	-0,03	-0,21	0,83	1829	**0,98**	0,88	-0,19	-0,70	0,48	1056	**0,94**	0,93	3,00	-0,10	0,05
**PA3005**	36983	5,83	309,51	8	32,20	0,80	-0,33	-1,24	0,22	604	**0,77**	0,83	-0,27	-1,12	0,26	696	**0,82**	0,75	-0,42	-1,49	0,14	471	**0,60**	0,79	3,00	-0,34	0,04
**PA3006**	27831	9,38	112,69	3	12,90	0,76	-0,40	-1,47	0,14	471	**0,66**	0,93	-0,11	-0,49	0,62	1400	**0,96**	0,73	-0,45	-1,58	0,11	425	**0,55**	0,81	3,00	-0,31	0,11
**PA3007**	24169	6,15	199,92	5	27,38	0,96	-0,07	-0,31	0,75	1657	**0,99**	1,03	0,04	0,05	0,96	2081	**1,00**	0,96	-0,05	-0,24	0,81	1667	**1,00**	0,98	3,00	-0,03	0,04
**PA3010**	19970	4,94	60,66	2	11,00	0,75	-0,41	-1,53	0,13	453	**0,61**	0,71	-0,50	-1,94	0,05	343	**0,33**	1,29	0,37	1,19	0,23	619	**0,78**	0,92	3,00	-0,12	0,32
**PA3011**	108431	8,70	1369,76	35	32,75	0,89	-0,17	-0,67	0,50	1114	**0,98**	0,92	-0,11	-0,52	0,60	1343	**0,97**	0,92	-0,12	-0,46	0,65	1330	**1,00**	0,91	3,00	-0,13	0,02
**PA3012**	14730	5,38	44,85	2	12,10	0,78	-0,35	-1,32	0,19	549	**0,73**	1,38	0,47	1,64	0,10	439	**0,50**	0,84	-0,26	-0,93	0,35	813	**0,89**	1,00	3,00	0,00	0,33
**PA3013**	44728	6,31	459,68	10	26,02	0,70	-0,51	-1,87	0,06	306	**0,44**	0,72	-0,48	-1,87	0,06	367	**0,36**	0,68	-0,56	-1,96	0,05	308	**0,33**	0,70	3,00	-0,52	0,02
**PA3014**	85971	5,67	1606,18	37	40,85	0,64	-0,64	-2,31	0,02	229	**0,20**	0,65	-0,63	-2,44	0,01	246	**0,13**	0,67	-0,58	-2,03	0,04	297	**0,30**	0,65	3,00	-0,62	0,01
**PA3019**	75482	5,24	803,45	20	27,78	0,93	-0,11	-0,47	0,64	1414	**0,98**	0,91	-0,13	-0,58	0,56	1257	**0,97**	0,79	-0,34	-1,20	0,23	610	**0,77**	0,88	3,00	-0,19	0,08
**PA3020**	76941	9,50	345,19	7	15,40	0,90	-0,15	-0,59	0,55	1218	**0,99**	0,88	-0,18	-0,78	0,44	1003	**0,94**	0,78	-0,36	-1,29	0,20	562	**0,72**	0,85	3,00	-0,23	0,07
**PA3021**	15914	7,56	115,80	3	20,30	0,86	-0,21	-0,82	0,41	948	**0,94**	0,90	-0,15	-0,65	0,52	1159	**0,96**	0,75	-0,41	-1,46	0,15	490	**0,61**	0,84	3,00	-0,25	0,08
**PA3022**	30661	5,87	283,45	5	22,32	0,96	-0,06	-0,29	0,77	1701	**0,99**	0,88	-0,18	-0,78	0,43	999	**0,94**	0,76	-0,40	-1,42	0,16	513	**0,63**	0,87	3,00	-0,21	0,10
**PA3028**	44859	5,25	184,56	4	10,50	0,80	-0,33	-1,22	0,22	617	**0,78**	0,60	-0,74	-2,87	0,00	191	**0,05**	0,70	-0,51	-1,80	0,07	349	**0,43**	0,70	3,00	-0,52	0,10
**PA3029**	20208	5,52	668,41	12	58,22	0,71	-0,50	-1,84	0,07	320	**0,44**	0,80	-0,32	-1,28	0,20	588	**0,74**	0,68	-0,55	-1,91	0,06	316	**0,36**	0,73	3,00	-0,45	0,06
**PA3040**	13062	5,67	165,61	3	16,80	0,76	-0,39	-1,46	0,14	476	**0,66**	1,01	0,02	-0,02	0,98	2136	**1,00**	0,88	-0,19	-0,70	0,49	1060	**0,94**	0,88	3,00	-0,18	0,13
**PA3046**	14019	4,79	215,49	5	19,30	0,85	-0,23	-0,90	0,37	873	**0,91**	0,84	-0,26	-1,06	0,29	744	**0,84**	0,93	-0,11	-0,43	0,67	1374	**1,00**	0,87	3,00	-0,20	0,05
**PA3047**	55234	9,69	224,05	7	11,40	0,95	-0,07	-0,34	0,74	1619	**0,99**	0,89	-0,17	-0,72	0,47	1060	**0,96**	0,86	-0,22	-0,79	0,43	942	**0,94**	0,90	3,00	-0,15	0,05
**PA3048**	85986	7,98	464,08	14	17,59	1,08	0,11	0,29	0,77	1698	**0,99**	0,85	-0,23	-0,97	0,33	815	**0,89**	0,84	-0,25	-0,89	0,37	847	**0,91**	0,92	3,00	-0,12	0,13
**PA3050**	38026	8,61	522,20	17	45,75	0,96	-0,06	-0,30	0,77	1685	**0,99**	1,05	0,07	0,16	0,87	1919	**0,98**	1,00	0,00	-0,07	0,94	1943	**1,00**	1,00	3,00	0,00	0,05
**PA3053**	39374	7,68	137,45	5	10,04	0,77	-0,37	-1,39	0,17	519	**0,69**	0,93	-0,10	-0,48	0,63	1427	**0,96**	0,96	-0,06	-0,27	0,79	1618	**1,00**	0,89	3,00	-0,17	0,10
**PA3056**	19464	6,02	227,96	3	23,03	0,80	-0,32	-1,20	0,23	628	**0,80**	0,76	-0,39	-1,54	0,12	483	**0,55**	1,07	0,10	0,27	0,79	1614	**1,00**	0,88	3,00	-0,19	0,17
**PA3068**	193406	5,84	5797,14	124	56,76	1,05	0,07	0,16	0,88	1930	**0,99**	1,16	0,21	0,70	0,48	1086	**0,96**	1,16	0,21	0,66	0,51	1101	**0,95**	1,12	3,00	0,17	0,06
**PA3070**	37613	5,14	116,96	4	9,73	0,83	-0,26	-1,01	0,31	768	**0,89**	0,93	-0,11	-0,51	0,61	1375	**0,96**	0,76	-0,40	-1,43	0,15	508	**0,62**	0,84	3,00	-0,25	0,09
**PA3075**	60886	5,16	208,82	3	9,53	1,11	0,15	0,46	0,64	1422	**0,98**	0,76	-0,39	-1,55	0,12	476	**0,55**	1,24	0,31	0,98	0,33	777	**0,87**	1,04	3,00	0,05	0,25
**PA3081**	53963	8,92	561,51	14	27,19	0,69	-0,54	-1,97	0,05	281	**0,38**	0,71	-0,50	-1,95	0,05	340	**0,32**	0,68	-0,56	-1,96	0,05	310	**0,33**	0,69	3,00	-0,53	0,02
**PA3082**	73810	4,74	316,80	10	14,28	0,47	-1,08	-3,87	0,00	98	**0,00**	0,54	-0,89	-3,42	0,00	147	**0,01**	0,46	-1,13	-3,88	0,00	117	**0,00**	0,49	3,00	-1,03	0,04
**PA3083**	104721	5,11	1250,81	30	28,39	0,94	-0,09	-0,40	0,69	1513	**0,99**	0,97	-0,04	-0,25	0,81	1778	**0,98**	0,90	-0,15	-0,57	0,57	1207	**0,97**	0,94	3,00	-0,09	0,04
**PA3088**	34413	6,79	147,43	5	19,74	1,07	0,10	0,29	0,78	1707	**0,99**	0,89	-0,16	-0,71	0,48	1080	**0,96**	0,87	-0,21	-0,76	0,45	989	**0,94**	0,94	3,00	-0,08	0,11
**PA3092**	78191	6,70	130,94	4	9,25	0,63	-0,66	-2,40	0,02	218	**0,16**	0,96	-0,05	-0,29	0,77	1695	**0,98**	0,79	-0,34	-1,22	0,22	601	**0,77**	0,79	3,00	-0,33	0,17
**PA3093**	41998	6,01	163,70	5	13,80	1,00	0,01	-0,06	0,95	2084	**1,00**	0,88	-0,19	-0,80	0,43	982	**0,94**	0,84	-0,25	-0,89	0,37	844	**0,91**	0,91	3,00	-0,14	0,09
**PA3101**	17976	8,04	125,69	3	24,10	0,72	-0,48	-1,76	0,08	356	**0,48**	0,58	-0,77	-2,98	0,00	178	**0,03**	0,58	-0,80	-2,76	0,01	199	**0,06**	0,63	3,00	-0,68	0,08
**PA3105**	73369	5,78	429,99	12	17,55	0,89	-0,17	-0,68	0,50	1108	**0,98**	0,86	-0,22	-0,91	0,37	858	**0,92**	0,98	-0,03	-0,14	0,89	1823	**1,00**	0,91	3,00	-0,14	0,06
**PA3106**	27793	4,91	322,94	7	26,76	1,05	0,07	0,16	0,87	1918	**0,99**	1,10	0,14	0,43	0,67	1492	**0,97**	0,97	-0,04	-0,20	0,84	1722	**1,00**	1,04	3,00	0,06	0,07
**PA3107**	45215	5,89	768,89	17	39,76	1,05	0,06	0,15	0,88	1943	**0,99**	1,04	0,05	0,10	0,92	2021	**0,99**	1,02	0,03	0,05	0,96	1973	**1,00**	1,04	3,00	0,05	0,01
**PA3108**	59352	6,33	1150,47	27	46,83	1,00	0,00	-0,08	0,94	2035	**1,00**	1,02	0,02	-0,01	0,99	2147	**1,00**	1,00	0,00	-0,05	0,96	1972	**1,00**	1,01	3,00	0,01	0,01
**PA3110**	24409	5,69	122,94	3	10,50	1,02	0,03	0,01	0,99	2164	**1,00**	0,88	-0,19	-0,79	0,43	995	**0,94**	1,05	0,07	0,18	0,86	1764	**1,00**	0,98	3,00	-0,03	0,09
**PA3111**	47813	5,20	280,98	6	18,50	0,95	-0,08	-0,35	0,72	1596	**0,99**	0,87	-0,19	-0,82	0,41	954	**0,94**	1,16	0,21	0,65	0,51	1112	**0,95**	0,99	3,00	-0,01	0,15
**PA3112**	34594	6,62	940,49	23	45,99	0,79	-0,34	-1,27	0,20	583	**0,76**	0,89	-0,18	-0,75	0,45	1034	**0,95**	0,83	-0,27	-0,96	0,34	796	**0,88**	0,84	3,00	-0,26	0,05
**PA3114**	32564	9,14	113,45	4	22,35	0,85	-0,23	-0,90	0,37	875	**0,91**	0,77	-0,37	-1,48	0,14	504	**0,60**	0,74	-0,44	-1,55	0,12	441	**0,57**	0,79	3,00	-0,35	0,06
**PA3115**	101914	4,18	1668,92	30	27,24	0,63	-0,67	-2,43	0,02	217	**0,15**	0,59	-0,77	-2,96	0,00	181	**0,04**	0,56	-0,85	-2,92	0,00	178	**0,04**	0,59	3,00	-0,76	0,04
**PA3116**	36416	4,77	179,20	6	14,30	0,92	-0,11	-0,48	0,63	1389	**0,99**	0,88	-0,19	-0,81	0,42	964	**0,94**	0,64	-0,64	-2,24	0,03	266	**0,20**	0,81	3,00	-0,30	0,15
**PA3117**	43103	5,54	894,57	19	42,71	0,92	-0,12	-0,51	0,61	1345	**0,99**	0,83	-0,26	-1,08	0,28	725	**0,83**	0,82	-0,29	-1,03	0,30	723	**0,86**	0,86	3,00	-0,22	0,05
**PA3118**	41252	5,02	428,60	10	25,38	0,82	-0,29	-1,09	0,27	705	**0,85**	0,81	-0,30	-1,20	0,23	629	**0,79**	0,83	-0,26	-0,95	0,34	801	**0,88**	0,82	3,00	-0,28	0,01
**PA3120**	25385	4,90	154,01	5	16,20	0,80	-0,32	-1,20	0,23	631	**0,80**	0,79	-0,34	-1,36	0,17	544	**0,69**	1,03	0,04	0,09	0,92	1905	**1,00**	0,87	3,00	-0,19	0,14
**PA3121**	54450	5,89	68,68	2	5,30	0,63	-0,66	-2,40	0,02	219	**0,16**	0,73	-0,45	-1,79	0,07	391	**0,41**	0,81	-0,30	-1,08	0,28	681	**0,84**	0,72	3,00	-0,47	0,09
**PA3126**	17725	5,80	473,82	11	55,70	0,82	-0,28	-1,07	0,29	719	**0,86**	0,89	-0,18	-0,75	0,45	1035	**0,95**	0,73	-0,46	-1,61	0,11	412	**0,54**	0,81	3,00	-0,30	0,08
**PA3131**	23697	5,39	315,68	5	21,34	0,73	-0,46	-1,69	0,09	381	**0,51**	0,71	-0,50	-1,95	0,05	342	**0,32**	0,78	-0,36	-1,27	0,20	569	**0,74**	0,74	3,00	-0,44	0,04

**PA3134**	60926	6,24	1099,86	26	40,90	0,82	-0,28	-1,06	0,29	725	**0,87**	0,80	-0,32	-1,28	0,20	585	**0,74**	0,85	-0,24	-0,86	0,39	875	**0,92**	0,82	3,00	-0,28	0,02
**PA3138**	80101	5,30	304,74	8	14,90	0,88	-0,18	-0,72	0,47	1058	**0,97**	0,80	-0,31	-1,27	0,21	598	**0,74**	0,94	-0,10	-0,38	0,70	1439	**1,00**	0,87	3,00	-0,19	0,07
**PA3139**	45592	6,21	2450,15	47	79,46	1,44	0,53	1,77	0,08	349	**0,48**	1,64	0,72	2,57	0,01	223	**0,10**	1,47	0,55	1,81	0,07	342	**0,42**	1,52	3,00	0,60	0,11
**PA3141**	77533	9,33	946,56	21	27,00	0,82	-0,29	-1,10	0,27	696	**0,85**	0,95	-0,07	-0,35	0,73	1612	**0,97**	0,81	-0,30	-1,09	0,28	673	**0,84**	0,86	3,00	-0,22	0,08
**PA3146**	35685	8,94	279,90	7	20,70	1,23	0,30	0,96	0,34	810	**0,90**	1,19	0,25	0,84	0,40	931	**0,93**	1,09	0,13	0,39	0,70	1438	**1,00**	1,17	3,00	0,23	0,07
**PA3147**	48168	6,51	404,27	8	26,35	1,12	0,17	0,51	0,61	1334	**0,99**	1,12	0,16	0,49	0,62	1395	**0,96**	1,46	0,55	1,80	0,07	347	**0,43**	1,23	3,00	0,30	0,20
**PA3148**	41326	5,64	1251,78	23	51,23	1,02	0,03	0,02	0,98	2147	**1,00**	1,07	0,10	0,28	0,78	1710	**0,98**	1,04	0,06	0,15	0,88	1813	**1,00**	1,05	3,00	0,06	0,03
**PA3149**	46027	8,86	195,28	5	15,48	1,02	0,03	0,02	0,98	2143	**1,00**	1,00	-0,01	-0,12	0,91	1993	**0,98**	1,11	0,16	0,47	0,64	1308	**1,00**	1,04	3,00	0,06	0,06
**PA3150**	48233	8,30	397,05	8	26,30	0,89	-0,17	-0,68	0,50	1110	**0,98**	0,89	-0,17	-0,71	0,48	1074	**0,96**	0,93	-0,10	-0,40	0,69	1411	**1,01**	0,90	3,00	-0,15	0,02
**PA3151**	30013	5,62	228,81	7	22,99	0,96	-0,06	-0,30	0,76	1679	**0,99**	1,10	0,14	0,43	0,67	1493	**0,97**	0,96	-0,06	-0,26	0,80	1640	**1,00**	1,01	3,00	0,01	0,08
**PA3152**	24758	6,58	417,85	9	39,77	0,87	-0,20	-0,78	0,44	997	**0,95**	1,14	0,19	0,61	0,54	1214	**0,97**	1,25	0,32	1,04	0,30	718	**0,86**	1,09	3,00	0,12	0,20
**PA3155**	42104	5,75	1215,95	25	44,20	0,98	-0,03	-0,20	0,85	1860	**0,99**	0,99	-0,01	-0,13	0,89	1967	**0,98**	1,04	0,06	0,15	0,88	1818	**1,00**	1,00	3,00	0,01	0,03
**PA3156**	22122	7,60	411,55	8	26,70	0,84	-0,25	-0,95	0,34	818	**0,91**	1,09	0,12	0,35	0,73	1620	**0,97**	1,06	0,09	0,24	0,81	1669	**1,00**	1,00	3,00	0,00	0,13
**PA3158**	38519	6,11	797,65	18	43,20	0,81	-0,31	-1,16	0,25	656	**0,82**	0,90	-0,16	-0,68	0,49	1119	**0,96**	0,80	-0,32	-1,13	0,26	647	**0,82**	0,84	3,00	-0,26	0,05
**PA3159**	52101	5,36	1600,42	38	54,71	0,93	-0,11	-0,47	0,64	1415	**0,98**	0,83	-0,27	-1,09	0,28	719	**0,83**	0,86	-0,22	-0,81	0,42	923	**0,93**	0,87	3,00	-0,20	0,05
**PA3160**	41301	6,09	883,05	19	37,46	0,89	-0,16	-0,66	0,51	1131	**0,98**	0,91	-0,13	-0,59	0,56	1240	**0,97**	0,83	-0,27	-0,96	0,34	794	**0,87**	0,88	3,00	-0,19	0,04
**PA3162**	68120	4,83	3704,11	68	58,65	0,96	-0,06	-0,29	0,77	1703	**0,99**	0,95	-0,08	-0,40	0,69	1537	**0,97**	0,93	-0,11	-0,43	0,67	1377	**1,00**	0,94	3,00	-0,08	0,02
**PA3163**	25677	5,02	664,56	13	51,50	0,98	-0,03	-0,20	0,84	1844	**0,99**	0,98	-0,03	-0,21	0,83	1843	**0,98**	1,06	0,08	0,21	0,84	1714	**1,00**	1,00	3,00	0,01	0,05
**PA3165**	41280	5,05	613,93	17	34,98	1,05	0,08	0,19	0,85	1875	**0,99**	0,89	-0,16	-0,71	0,48	1078	**0,96**	0,97	-0,04	-0,21	0,83	1708	**1,01**	0,97	3,00	-0,04	0,08
**PA3166**	43961	5,89	675,88	15	33,57	0,90	-0,15	-0,60	0,55	1212	**0,98**	0,94	-0,10	-0,46	0,65	1447	**0,97**	0,99	-0,01	-0,10	0,92	1901	**1,00**	0,94	3,00	-0,08	0,05
**PA3167**	42578	4,96	936,39	22	47,93	0,91	-0,14	-0,58	0,56	1245	**0,98**	0,90	-0,15	-0,65	0,52	1161	**0,96**	0,91	-0,13	-0,50	0,62	1276	**0,99**	0,91	3,00	-0,14	0,01
**PA3168**	106928	4,91	2432,36	54	43,24	0,84	-0,25	-0,97	0,33	800	**0,90**	0,81	-0,30	-1,20	0,23	631	**0,79**	0,80	-0,32	-1,15	0,25	639	**0,81**	0,82	3,00	-0,29	0,02
**PA3169**	40599	5,28	312,78	9	39,50	0,92	-0,11	-0,48	0,63	1390	**0,99**	0,88	-0,19	-0,79	0,43	993	**0,94**	0,85	-0,23	-0,83	0,41	908	**0,92**	0,89	3,00	-0,17	0,04
**PA3170**	49990	5,42	269,41	8	18,98	0,87	-0,20	-0,79	0,43	987	**0,94**	0,92	-0,11	-0,52	0,60	1341	**0,97**	1,02	0,02	0,02	0,98	2036	**1,00**	0,94	3,00	-0,10	0,07
**PA3171**	27710	5,91	293,59	7	26,09	1,00	0,00	-0,09	0,92	2005	**1,00**	0,99	-0,01	-0,14	0,89	1962	**0,98**	0,85	-0,24	-0,86	0,39	880	**0,92**	0,95	3,00	-0,08	0,08
**PA3172**	26167	5,15	257,95	10	49,10	0,92	-0,12	-0,51	0,61	1336	**0,99**	0,91	-0,14	-0,63	0,53	1194	**0,96**	0,90	-0,16	-0,59	0,56	1186	**0,97**	0,91	3,00	-0,14	0,01
**PA3173**	27324	5,09	327,45	6	25,00	1,03	0,05	0,09	0,93	2023	**1,00**	0,91	-0,13	-0,58	0,56	1255	**0,97**	0,88	-0,18	-0,66	0,51	1109	**0,95**	0,94	3,00	-0,08	0,08
**PA3179**	46384	10,32	550,76	16	38,86	0,89	-0,16	-0,64	0,52	1140	**0,99**	0,84	-0,25	-1,04	0,30	758	**0,85**	0,83	-0,27	-0,96	0,34	790	**0,88**	0,85	3,00	-0,23	0,03
**PA3181**	24987	7,01	424,24	8	44,65	3,30	1,72	5,96	0,00	53	**0,00**	2,68	1,42	5,20	0,00	79	**0,00**	3,11	1,64	5,49	0,00	75	**0,00**	3,03	3,00	1,60	0,32
**PA3182**	26322	6,06	630,11	12	56,17	3,32	1,73	5,99	0,00	51	**0,00**	3,26	1,71	6,26	0,00	57	**0,00**	3,27	1,71	5,73	0,00	65	**0,00**	3,28	3,00	1,71	0,03
**PA3183**	58219	5,60	1039,19	28	43,80	3,47	1,79	6,22	0,00	46	**0,00**	2,76	1,46	5,36	0,00	72	**0,00**	3,08	1,62	5,44	0,00	76	**0,00**	3,10	3,00	1,63	0,36
**PA3184**	32539	6,32	155,18	4	17,28	1,10	0,14	0,42	0,67	1479	**0,99**	0,82	-0,28	-1,13	0,26	681	**0,82**	0,83	-0,27	-0,97	0,33	785	**0,87**	0,92	3,00	-0,12	0,16
**PA3187**	44848	6,99	1190,55	26	51,80	4,38	2,13	7,40	0,00	28	**0,00**	4,50	2,17	7,99	0,00	28	**0,00**	5,80	2,54	8,54	0,00	4	**0,00**	4,90	3,00	2,29	0,79
**PA3190**	49898	5,68	3139,84	68	66,00	6,32	2,66	9,26	0,00	11	**0,00**	7,91	2,98	11,02	0,00	16	**0,00**	8,40	3,07	10,35	0,00	19	**0,00**	7,54	3,00	2,92	1,09
**PA3193**	35247	5,18	440,97	8	23,45	2,14	1,10	3,77	0,00	105	**0,00**	2,41	1,27	4,64	0,00	96	**0,00**	1,94	0,96	3,18	0,00	155	**0,02**	2,16	3,00	1,11	0,24
**PA3194**	67671	6,24	1070,19	24	35,75	2,01	1,01	3,46	0,00	118	**0,01**	2,16	1,11	4,04	0,00	115	**0,00**	2,29	1,19	3,99	0,00	113	**0,00**	2,15	3,00	1,11	0,14
**PA3195**	37582	6,15	586,54	15	38,30	3,08	1,62	5,62	0,00	63	**0,00**	3,66	1,87	6,88	0,00	44	**0,00**	5,94	2,57	8,65	0,00	7	**0,00**	4,23	3,00	2,08	1,51
**PA3208**	20460	6,06	473,04	10	51,08	0,85	-0,23	-0,90	0,37	876	**0,91**	0,80	-0,32	-1,27	0,20	590	**0,74**	0,82	-0,28	-1,00	0,32	755	**0,86**	0,83	3,00	-0,28	0,02
**PA3211**	42193	5,40	100,20	2	7,10	0,89	-0,17	-0,68	0,50	1103	**0,98**	1,08	0,11	0,32	0,75	1659	**0,98**	0,57	-0,81	-2,82	0,00	190	**0,05**	0,85	3,00	-0,24	0,26
**PA3213**	35373	6,46	133,04	3	10,37	0,93	-0,11	-0,47	0,64	1410	**0,98**	0,93	-0,11	-0,51	0,61	1358	**0,97**	0,89	-0,17	-0,64	0,52	1122	**0,96**	0,91	3,00	-0,13	0,02
**PA3221**	13040	5,77	77,32	2	10,80	1,19	0,25	0,81	0,42	969	**0,93**	2,15	1,11	4,02	0,00	117	**0,00**	1,79	0,84	2,78	0,01	194	**0,06**	1,71	3,00	0,77	0,48
**PA3225**	35276	6,85	405,91	9	35,31	0,82	-0,28	-1,06	0,29	732	**0,87**	0,88	-0,18	-0,77	0,44	1011	**0,94**	0,91	-0,13	-0,50	0,62	1279	**1,00**	0,87	3,00	-0,19	0,05
**PA3226**	32361	7,63	86,26	3	11,97	1,02	0,03	0,03	0,98	2137	**1,00**	0,95	-0,08	-0,38	0,70	1566	**0,97**	0,71	-0,49	-1,72	0,08	369	**0,47**	0,89	3,00	-0,16	0,16
**PA3227**	21723	7,85	550,92	13	57,97	1,09	0,13	0,38	0,71	1560	**0,98**	1,13	0,18	0,57	0,57	1273	**0,96**	1,20	0,26	0,84	0,40	897	**0,92**	1,14	3,00	0,19	0,05
**PA3228**	70057	6,98	130,80	3	6,97	1,11	0,16	0,47	0,64	1413	**0,98**	1,82	0,87	3,13	0,00	172	**0,02**	1,47	0,56	1,83	0,07	338	**0,41**	1,47	3,00	0,56	0,35
**PA3238**	53731	6,16	226,09	6	22,50	0,94	-0,09	-0,41	0,68	1503	**0,99**	0,92	-0,12	-0,53	0,60	1332	**0,97**	0,74	-0,43	-1,51	0,13	458	**0,59**	0,87	3,00	-0,20	0,11
**PA3242**	37764	9,84	180,83	4	12,65	0,86	-0,22	-0,86	0,39	916	**0,93**	1,06	0,09	0,24	0,81	1798	**0,98**	0,96	-0,07	-0,28	0,78	1608	**1,00**	0,96	3,00	-0,06	0,10
**PA3243**	30072	6,14	390,89	10	31,82	0,79	-0,33	-1,25	0,21	598	**0,77**	0,84	-0,25	-1,01	0,31	788	**0,86**	0,81	-0,31	-1,09	0,27	671	**0,84**	0,82	3,00	-0,29	0,03

**PA3244**	32573	5,58	1424,30	27	68,58	0,79	-0,34	-1,29	0,20	573	**0,75**	0,77	-0,37	-1,47	0,14	505	**0,60**	0,81	-0,31	-1,11	0,27	659	**0,83**	0,79	3,00	-0,34	0,02
**PA3245**	10551	5,76	270,16	9	42,90	0,75	-0,42	-1,55	0,12	439	**0,60**	0,80	-0,32	-1,28	0,20	582	**0,74**	0,80	-0,32	-1,15	0,25	640	**0,81**	0,78	3,00	-0,35	0,03
**PA3255**	22579	4,63	99,25	3	18,80	0,85	-0,24	-0,91	0,36	866	**0,91**	0,91	-0,14	-0,61	0,54	1211	**0,97**	0,92	-0,12	-0,47	0,64	1315	**1,00**	0,89	3,00	-0,16	0,04
**PA3256**	35130	5,43	392,34	10	26,40	0,91	-0,13	-0,54	0,59	1289	**0,99**	0,80	-0,33	-1,33	0,18	559	**0,71**	0,78	-0,35	-1,26	0,21	575	**0,75**	0,83	3,00	-0,27	0,07
**PA3257**	86637	6,22	844,28	20	28,52	0,80	-0,32	-1,20	0,23	629	**0,79**	0,90	-0,15	-0,66	0,51	1135	**0,96**	0,83	-0,27	-0,96	0,34	795	**0,87**	0,84	3,00	-0,24	0,05
**PA3262**	30477	5,09	1605,64	30	52,68	0,84	-0,25	-0,95	0,34	815	**0,91**	0,80	-0,33	-1,32	0,19	561	**0,72**	0,82	-0,29	-1,02	0,31	735	**0,86**	0,82	3,00	-0,29	0,02
**PA3263**	37158	4,99	978,97	21	60,64	0,66	-0,59	-2,16	0,03	256	**0,26**	0,73	-0,46	-1,80	0,07	388	**0,40**	0,68	-0,56	-1,96	0,05	309	**0,33**	0,69	3,00	-0,54	0,03
**PA3266**	8465	8,15	351,57	6	75,40	0,92	-0,12	-0,50	0,62	1369	**0,98**	0,69	-0,54	-2,12	0,03	298	**0,25**	0,75	-0,41	-1,44	0,15	498	**0,62**	0,79	3,00	-0,35	0,12
**PA3268**	82926	5,06	386,28	10	18,40	1,39	0,47	1,57	0,12	428	**0,59**	1,37	0,46	1,61	0,11	451	**0,52**	1,36	0,44	1,44	0,15	500	**0,62**	1,37	3,00	0,46	0,01
**PA3270**	22798	9,51	365,25	8	41,28	0,80	-0,31	-1,18	0,24	641	**0,80**	0,83	-0,27	-1,11	0,26	700	**0,82**	0,79	-0,35	-1,23	0,22	589	**0,77**	0,81	3,00	-0,31	0,02
**PA3271**	131631	8,26	291,02	7	5,57	0,92	-0,12	-0,49	0,62	1376	**0,99**	0,70	-0,51	-2,00	0,05	324	**0,30**	0,78	-0,35	-1,24	0,21	582	**0,76**	0,80	3,00	-0,32	0,11
**PA3286**	40409	5,95	874,08	19	39,00	0,77	-0,38	-1,43	0,15	497	**0,67**	0,70	-0,51	-2,01	0,04	322	**0,30**	0,69	-0,54	-1,89	0,06	323	**0,37**	0,72	3,00	-0,48	0,04
**PA3295**	17066	5,75	223,70	7	39,69	0,60	-0,73	-2,65	0,01	182	**0,10**	0,87	-0,20	-0,86	0,39	911	**0,93**	0,70	-0,52	-1,82	0,07	340	**0,42**	0,72	3,00	-0,47	0,13
**PA3297**	158830	8,43	803,37	22	16,45	0,88	-0,18	-0,71	0,48	1067	**0,97**	0,88	-0,18	-0,78	0,44	1009	**0,94**	0,91	-0,13	-0,50	0,61	1273	**0,99**	0,89	3,00	-0,16	0,02
**PA3299**	67885	7,11	1195,95	27	41,89	0,83	-0,27	-1,01	0,31	764	**0,88**	0,82	-0,28	-1,15	0,25	664	**0,82**	0,82	-0,28	-1,00	0,32	753	**0,86**	0,83	3,00	-0,28	0,00
**PA3302**	18188	5,24	537,86	10	42,90	0,85	-0,24	-0,93	0,35	841	**0,91**	0,76	-0,39	-1,56	0,12	472	**0,55**	0,73	-0,46	-1,62	0,10	407	**0,53**	0,78	3,00	-0,36	0,06
**PA3308**	109984	5,06	1853,08	39	41,35	0,92	-0,12	-0,50	0,62	1366	**0,99**	0,90	-0,15	-0,65	0,51	1149	**0,96**	0,92	-0,12	-0,45	0,65	1342	**1,00**	0,92	3,00	-0,13	0,01
**PA3309**	17540	5,50	951,00	21	78,80	0,71	-0,49	-1,80	0,07	337	**0,47**	0,82	-0,28	-1,15	0,25	666	**0,82**	0,82	-0,29	-1,05	0,29	707	**0,85**	0,78	3,00	-0,35	0,06
**PA3310**	65681	6,88	50,63	2	5,30	1,18	0,24	0,75	0,46	1026	**0,96**	1,09	0,12	0,37	0,71	1591	**0,97**	0,87	-0,20	-0,74	0,46	1013	**0,93**	1,05	3,00	0,06	0,16
**PA3312**	32271	5,65	230,06	6	17,13	0,97	-0,04	-0,23	0,81	1790	**0,99**	0,84	-0,24	-1,00	0,32	793	**0,86**	0,95	-0,07	-0,30	0,77	1584	**1,00**	0,92	3,00	-0,12	0,07
**PA3313**	40263	9,61	1163,19	29	61,49	0,76	-0,39	-1,46	0,14	477	**0,66**	0,87	-0,21	-0,88	0,38	884	**0,93**	0,84	-0,24	-0,88	0,38	859	**0,91**	0,82	3,00	-0,28	0,06
**PA3322**	27272	5,50	116,05	3	14,77	0,97	-0,04	-0,24	0,81	1783	**0,99**	0,73	-0,45	-1,77	0,08	397	**0,42**	1,31	0,39	1,28	0,20	566	**0,73**	1,01	3,00	0,01	0,29
**PA3326**	24002	5,44	174,92	4	19,15	0,70	-0,51	-1,88	0,06	304	**0,43**	0,89	-0,16	-0,70	0,48	1085	**0,96**	0,69	-0,54	-1,88	0,06	324	**0,38**	0,76	3,00	-0,39	0,12
**PA3337**	38024	6,11	242,88	5	15,88	0,90	-0,14	-0,59	0,56	1230	**0,98**	0,82	-0,28	-1,16	0,25	660	**0,81**	0,79	-0,33	-1,19	0,23	620	**0,78**	0,84	3,00	-0,25	0,06
**PA3340**	75016	4,83	63,86	2	6,30	1,41	0,50	1,66	0,10	393	**0,54**	1,20	0,27	0,90	0,37	862	**0,92**	1,25	0,33	1,04	0,30	715	**0,85**	1,29	3,00	0,37	0,11
**PA3341**	16684	8,01	180,65	4	24,30	1,06	0,08	0,20	0,84	1855	**0,99**	1,20	0,26	0,87	0,38	890	**0,93**	0,98	-0,02	-0,13	0,89	1839	**1,00**	1,08	3,00	0,11	0,11
**PA3344**	82978	5,68	488,62	12	21,07	0,85	-0,23	-0,88	0,38	893	**0,93**	0,95	-0,08	-0,38	0,70	1567	**0,97**	0,90	-0,15	-0,57	0,57	1212	**0,97**	0,90	3,00	-0,15	0,05
**PA3348**	33086	9,31	235,93	5	16,62	0,92	-0,11	-0,48	0,63	1391	**0,99**	0,94	-0,10	-0,45	0,65	1456	**0,97**	1,07	0,10	0,28	0,78	1603	**1,00**	0,98	3,00	-0,03	0,08
**PA3349**	36635	5,85	560,42	15	42,71	0,84	-0,26	-0,99	0,32	784	**0,89**	0,87	-0,20	-0,85	0,40	924	**0,93**	0,83	-0,28	-0,99	0,32	765	**0,87**	0,84	3,00	-0,24	0,02
**PA3351**	12252	5,29	155,79	4	39,73	0,99	-0,01	-0,11	0,91	1983	**1,00**	1,08	0,12	0,34	0,73	1628	**0,98**	1,23	0,30	0,95	0,34	797	**0,88**	1,10	3,00	0,14	0,12
**PA3352**	17579	5,31	254,33	7	41,66	0,99	-0,02	-0,15	0,88	1934	**0,99**	0,97	-0,05	-0,27	0,79	1741	**0,98**	0,85	-0,23	-0,84	0,40	900	**0,92**	0,94	3,00	-0,10	0,07
**PA3353**	31268	6,37	137,42	5	21,54	1,23	0,29	0,95	0,34	819	**0,91**	1,22	0,29	0,99	0,32	801	**0,87**	1,44	0,53	1,73	0,08	366	**0,47**	1,30	3,00	0,38	0,13
**PA3356**	46797	5,47	320,23	6	13,05	1,07	0,10	0,28	0,78	1714	**0,99**	1,18	0,24	0,80	0,43	980	**0,94**	1,02	0,03	0,04	0,97	1998	**1,00**	1,09	3,00	0,13	0,08
**PA3357**	49507	5,70	265,12	9	22,78	0,97	-0,05	-0,25	0,80	1770	**0,99**	0,74	-0,44	-1,75	0,08	401	**0,43**	0,73	-0,46	-1,61	0,11	411	**0,54**	0,81	3,00	-0,30	0,14
**PA3366**	41189	5,31	216,11	7	16,26	3,00	1,58	5,48	0,00	67	**0,00**	3,77	1,91	7,04	0,00	42	**0,00**	3,12	1,64	5,50	0,00	74	**0,00**	3,30	3,00	1,72	0,41
**PA3397**	31562	5,65	639,67	17	42,72	0,99	-0,01	-0,11	0,91	1987	**1,00**	0,99	-0,02	-0,16	0,87	1930	**0,98**	0,97	-0,05	-0,22	0,82	1685	**1,01**	0,98	3,00	-0,02	0,01
**PA3402**	37769	5,95	98,74	4	11,45	1,20	0,26	0,83	0,41	938	**0,94**	0,99	-0,02	-0,18	0,86	1896	**0,98**	1,47	0,56	1,83	0,07	339	**0,41**	1,22	3,00	0,28	0,24
**PA3413**	11160	4,51	182,25	3	27,67	0,80	-0,33	-1,22	0,22	616	**0,78**	0,61	-0,70	-2,72	0,01	208	**0,07**	0,56	-0,85	-2,93	0,00	177	**0,04**	0,66	3,00	-0,61	0,13
**PA3435**	16628	4,54	433,36	9	62,20	0,74	-0,44	-1,62	0,11	411	**0,56**	0,85	-0,24	-0,99	0,32	799	**0,87**	0,74	-0,44	-1,54	0,12	444	**0,57**	0,77	3,00	-0,37	0,06
**PA3438**	22110	7,01	72,72	3	27,77	1,02	0,03	0,02	0,99	2155	**0,99**	0,78	-0,36	-1,44	0,15	516	**0,63**	1,07	0,09	0,26	0,80	1636	**1,00**	0,96	3,00	-0,07	0,15
**PA3440**	12874	5,65	452,73	10	68,16	0,78	-0,37	-1,37	0,17	524	**0,71**	1,04	0,06	0,13	0,90	1974	**0,98**	0,93	-0,10	-0,40	0,69	1416	**1,01**	0,92	3,00	-0,12	0,13
**PA3450**	26069	5,29	127,25	4	17,93	0,85	-0,24	-0,92	0,36	857	**0,91**	1,10	0,14	0,41	0,68	1514	**0,97**	0,93	-0,11	-0,43	0,67	1368	**1,00**	0,96	3,00	-0,06	0,13
**PA3452**	62356	5,66	1387,42	34	43,00	0,85	-0,23	-0,87	0,38	896	**0,93**	0,76	-0,40	-1,57	0,12	467	**0,54**	0,76	-0,39	-1,39	0,17	527	**0,65**	0,79	3,00	-0,34	0,05
**PA3453**	24768	5,44	431,88	10	52,22	0,78	-0,35	-1,31	0,19	562	**0,74**	0,81	-0,30	-1,23	0,22	617	**0,76**	0,78	-0,36	-1,26	0,21	570	**0,74**	0,79	3,00	-0,34	0,02
**PA3455**	63431	8,20	113,32	3	7,47	0,82	-0,28	-1,06	0,29	729	**0,86**	0,62	-0,69	-2,66	0,01	213	**0,08**	0,87	-0,20	-0,74	0,46	1020	**0,93**	0,77	3,00	-0,37	0,13
**PA3456**	72368	6,75	275,51	7	8,60	0,88	-0,19	-0,73	0,46	1034	**0,97**	0,91	-0,13	-0,59	0,56	1242	**0,97**	1,01	0,01	-0,03	0,97	2010	**1,00**	0,93	3,00	-0,10	0,07
**PA3466**	54429	9,79	360,38	12	26,53	0,97	-0,05	-0,25	0,80	1771	**0,99**	0,92	-0,12	-0,56	0,58	1298	**0,96**	0,91	-0,14	-0,54	0,59	1229	**0,98**	0,93	3,00	-0,10	0,03
**PA3468**	51185	8,41	164,29	6	12,90	0,80	-0,32	-1,21	0,23	623	**0,79**	0,80	-0,32	-1,30	0,19	571	**0,73**	0,62	-0,70	-2,42	0,02	236	**0,13**	0,74	3,00	-0,44	0,11

**PA3471**	65434	5,34	1031,61	17	29,67	0,69	-0,53	-1,95	0,05	287	**0,39**	0,66	-0,60	-2,33	0,02	265	**0,16**	0,71	-0,50	-1,76	0,08	358	**0,45**	0,69	3,00	-0,54	0,02
**PA3480**	22956	5,95	272,73	8	26,20	0,79	-0,35	-1,30	0,19	567	**0,75**	0,91	-0,14	-0,62	0,54	1202	**0,96**	0,76	-0,40	-1,43	0,15	507	**0,62**	0,82	3,00	-0,29	0,08
**PA3481**	40777	5,27	701,13	14	45,10	0,81	-0,30	-1,15	0,25	667	**0,82**	0,84	-0,25	-1,03	0,30	769	**0,85**	0,88	-0,18	-0,66	0,51	1108	**0,95**	0,85	3,00	-0,24	0,04
**PA3482**	81034	5,42	1727,01	41	42,54	0,86	-0,21	-0,83	0,41	945	**0,93**	0,96	-0,06	-0,31	0,75	1664	**0,98**	0,93	-0,10	-0,40	0,69	1415	**1,00**	0,92	3,00	-0,12	0,05
**PA3483**	29893	6,34	198,65	6	24,05	0,85	-0,24	-0,91	0,36	867	**0,91**	0,98	-0,03	-0,19	0,85	1871	**0,98**	0,94	-0,09	-0,38	0,71	1457	**1,00**	0,92	3,00	-0,12	0,07
**PA3484**	46574	8,79	85,97	3	10,80	1,11	0,15	0,46	0,64	1423	**0,98**	0,72	-0,48	-1,88	0,06	362	**0,36**	0,69	-0,53	-1,84	0,07	333	**0,41**	0,84	3,00	-0,25	0,24
**PA3524**	15008	4,87	51,84	2	14,45	0,68	-0,55	-2,01	0,04	276	**0,35**	0,69	-0,54	-2,12	0,03	300	**0,25**	0,62	-0,69	-2,39	0,02	241	**0,15**	0,66	3,00	-0,59	0,04
**PA3525**	49252	5,40	1250,89	27	56,00	0,89	-0,16	-0,65	0,52	1138	**0,99**	0,82	-0,29	-1,19	0,23	634	**0,79**	0,83	-0,28	-0,99	0,32	764	**0,87**	0,85	3,00	-0,24	0,04
**PA3526**	36868	9,79	80,60	2	15,30	0,99	-0,01	-0,13	0,90	1965	**0,99**	0,83	-0,28	-1,13	0,26	682	**0,82**	0,87	-0,20	-0,72	0,47	1037	**0,93**	0,90	3,00	-0,16	0,08
**PA3527**	40008	6,17	119,33	4	13,80	0,64	-0,63	-2,31	0,02	232	**0,20**	1,00	0,01	-0,07	0,94	2054	**0,99**	1,19	0,26	0,81	0,42	928	**0,93**	0,95	3,00	-0,08	0,28
**PA3528**	26286	5,02	520,58	8	39,15	1,23	0,30	0,98	0,33	792	**0,90**	0,93	-0,11	-0,50	0,62	1386	**0,96**	1,00	0,00	-0,04	0,97	1984	**1,00**	1,05	3,00	0,08	0,16
**PA3529**	24062	5,37	1228,82	30	85,50	0,55	-0,86	-3,09	0,00	145	**0,03**	0,72	-0,47	-1,86	0,06	368	**0,37**	0,59	-0,77	-2,66	0,01	210	**0,08**	0,62	3,00	-0,69	0,09
**PA3531**	20507	5,05	251,39	5	32,76	0,62	-0,70	-2,53	0,01	199	**0,12**	0,52	-0,95	-3,64	0,00	137	**0,00**	0,63	-0,67	-2,34	0,02	252	**0,16**	0,59	3,00	-0,77	0,06
**PA3533**	13080	4,82	536,08	14	61,10	0,81	-0,30	-1,14	0,25	671	**0,82**	0,94	-0,09	-0,43	0,67	1495	**0,97**	1,07	0,09	0,26	0,79	1632	**1,00**	0,94	3,00	-0,09	0,13
**PA3537**	35384	5,55	212,20	7	23,26	0,77	-0,37	-1,39	0,16	516	**0,69**	0,95	-0,08	-0,40	0,69	1541	**0,97**	0,67	-0,57	-1,99	0,05	303	**0,31**	0,80	3,00	-0,33	0,14
**PA3538**	40935	5,96	136,57	4	14,48	0,83	-0,27	-1,04	0,30	745	**0,87**	0,91	-0,14	-0,63	0,53	1189	**0,96**	0,77	-0,37	-1,31	0,19	555	**0,71**	0,84	3,00	-0,26	0,07
**PA3539**	31704	6,23	310,88	8	26,83	0,77	-0,38	-1,41	0,16	505	**0,68**	0,70	-0,52	-2,03	0,04	317	**0,29**	0,72	-0,47	-1,65	0,10	395	**0,52**	0,73	3,00	-0,45	0,04
**PA3552**	43512	7,79	142,14	4	12,15	0,73	-0,45	-1,67	0,10	390	**0,53**	0,80	-0,32	-1,28	0,20	586	**0,74**	0,72	-0,47	-1,64	0,10	399	**0,53**	0,75	3,00	-0,41	0,04
**PA3553**	37935	8,31	79,97	2	8,30	1,00	0,00	-0,09	0,93	2021	**1,00**	0,99	-0,01	-0,13	0,90	1978	**0,98**	0,85	-0,24	-0,86	0,39	874	**0,92**	0,95	3,00	-0,08	0,09
**PA3554**	77028	6,23	1000,20	25	41,50	0,80	-0,32	-1,22	0,22	619	**0,78**	0,84	-0,25	-1,02	0,31	772	**0,86**	0,76	-0,39	-1,38	0,17	531	**0,65**	0,80	3,00	-0,32	0,04
**PA3561**	34271	5,56	177,11	4	16,90	1,24	0,31	1,02	0,31	763	**0,88**	1,21	0,28	0,94	0,35	834	**0,90**	1,26	0,34	1,09	0,28	678	**0,84**	1,24	3,00	0,31	0,03
**PA3562**	104138	5,05	1012,21	24	27,05	1,30	0,38	1,24	0,21	602	**0,77**	1,19	0,25	0,84	0,40	928	**0,93**	1,15	0,20	0,62	0,54	1152	**0,96**	1,21	3,00	0,28	0,08
**PA3563**	37540	6,04	509,25	11	33,70	1,12	0,17	0,51	0,61	1348	**0,99**	1,01	0,02	-0,04	0,97	2108	**0,99**	1,06	0,08	0,21	0,84	1715	**1,00**	1,06	3,00	0,09	0,06
**PA3566**	11495	5,80	160,10	3	29,57	0,78	-0,36	-1,33	0,18	547	**0,73**	0,75	-0,42	-1,67	0,10	429	**0,48**	0,99	-0,01	-0,10	0,92	1892	**1,00**	0,84	3,00	-0,25	0,13
**PA3567**	38673	6,12	234,91	6	26,40	0,85	-0,24	-0,91	0,36	868	**0,91**	0,86	-0,21	-0,89	0,37	869	**0,92**	0,81	-0,31	-1,10	0,27	669	**0,84**	0,84	3,00	-0,25	0,03
**PA3574**	25743	5,91	102,10	3	15,27	0,76	-0,39	-1,44	0,15	495	**0,66**	0,87	-0,20	-0,85	0,40	919	**0,93**	0,63	-0,67	-2,32	0,02	254	**0,16**	0,75	3,00	-0,41	0,12
**PA3579**	56415	5,55	377,88	11	17,67	0,97	-0,04	-0,22	0,83	1810	**0,99**	0,74	-0,44	-1,74	0,08	407	**0,44**	0,84	-0,24	-0,88	0,38	854	**0,91**	0,85	3,00	-0,23	0,12
**PA3582**	59279	5,70	967,11	24	38,05	2,40	1,26	4,35	0,00	88	**0,00**	2,52	1,34	4,88	0,00	87	**0,00**	3,20	1,68	5,63	0,00	68	**0,00**	2,71	3,00	1,44	0,43
**PA3583**	28416	5,41	263,79	7	26,01	0,86	-0,22	-0,85	0,39	922	**0,93**	0,97	-0,05	-0,28	0,78	1719	**0,98**	0,90	-0,16	-0,59	0,55	1181	**0,96**	0,91	3,00	-0,14	0,06
**PA3584**	59923	8,46	761,39	21	38,15	1,20	0,26	0,82	0,41	952	**0,94**	1,03	0,05	0,07	0,94	2060	**0,99**	1,11	0,16	0,47	0,64	1309	**1,00**	1,11	3,00	0,16	0,08
**PA3602**	62477	7,64	375,52	8	17,35	0,72	-0,48	-1,76	0,08	351	**0,48**	0,82	-0,28	-1,16	0,25	659	**0,81**	0,54	-0,88	-3,04	0,00	167	**0,03**	0,69	3,00	-0,53	0,14
**PA3604**	24571	4,99	597,02	13	50,89	0,77	-0,38	-1,43	0,15	498	**0,67**	0,82	-0,29	-1,19	0,23	635	**0,79**	0,86	-0,21	-0,78	0,44	963	**0,93**	0,81	3,00	-0,30	0,05
**PA3607**	41855	5,35	408,86	9	19,30	0,77	-0,38	-1,40	0,16	511	**0,69**	0,75	-0,41	-1,64	0,10	438	**0,50**	0,74	-0,44	-1,55	0,12	440	**0,57**	0,75	3,00	-0,41	0,02
**PA3610**	43075	5,57	797,10	20	54,36	0,86	-0,22	-0,85	0,40	926	**0,93**	0,83	-0,26	-1,08	0,28	726	**0,83**	1,03	0,04	0,09	0,93	1911	**1,00**	0,91	3,00	-0,14	0,11
**PA3611**	16267	8,86	229,29	4	31,05	1,02	0,03	0,01	0,99	2165	**1,00**	1,14	0,19	0,60	0,55	1219	**0,97**	0,79	-0,34	-1,22	0,22	594	**0,77**	0,98	3,00	-0,03	0,18
**PA3613**	90749	5,99	345,45	11	17,35	0,80	-0,32	-1,21	0,23	625	**0,79**	0,82	-0,28	-1,15	0,25	667	**0,81**	0,62	-0,69	-2,39	0,02	240	**0,15**	0,75	3,00	-0,42	0,11
**PA3614**	53751	6,47	268,86	6	11,97	0,62	-0,70	-2,53	0,01	200	**0,13**	1,00	0,00	-0,11	0,91	1999	**0,98**	1,04	0,06	0,15	0,88	1814	**1,00**	0,89	3,00	-0,17	0,23
**PA3615**	42117	5,68	212,80	4	11,98	1,13	0,18	0,56	0,58	1271	**0,98**	0,75	-0,42	-1,66	0,10	430	**0,48**	0,81	-0,30	-1,09	0,28	677	**0,84**	0,90	3,00	-0,16	0,21
**PA3617**	40551	5,39	1287,28	25	62,04	0,65	-0,62	-2,25	0,02	244	**0,22**	0,63	-0,67	-2,59	0,01	220	**0,09**	0,66	-0,59	-2,06	0,04	293	**0,28**	0,65	3,00	-0,63	0,02
**PA3618**	18228	5,26	208,04	4	26,80	0,73	-0,45	-1,67	0,10	392	**0,53**	0,92	-0,13	-0,56	0,57	1288	**0,96**	0,70	-0,52	-1,83	0,07	337	**0,41**	0,78	3,00	-0,36	0,12
**PA3620**	99465	5,82	1362,86	36	33,24	0,85	-0,24	-0,93	0,35	839	**0,91**	0,84	-0,25	-1,03	0,30	762	**0,86**	0,81	-0,30	-1,07	0,29	691	**0,85**	0,83	3,00	-0,26	0,02
**PA3621**	13429	3,99	116,02	3	24,00	0,96	-0,06	-0,28	0,78	1711	**0,99**	0,77	-0,37	-1,47	0,14	506	**0,61**	0,85	-0,24	-0,86	0,39	872	**0,92**	0,86	3,00	-0,22	0,09
**PA3624**	23969	5,84	463,17	9	47,77	0,79	-0,34	-1,28	0,20	577	**0,75**	0,78	-0,36	-1,44	0,15	512	**0,63**	0,84	-0,26	-0,94	0,35	808	**0,89**	0,80	3,00	-0,32	0,03
**PA3625**	27164	5,25	187,58	4	16,50	0,78	-0,35	-1,32	0,19	550	**0,73**	0,88	-0,18	-0,77	0,44	1016	**0,94**	0,89	-0,16	-0,61	0,54	1167	**0,96**	0,85	3,00	-0,23	0,06
**PA3627**	17805	6,54	195,34	5	26,22	1,07	0,10	0,28	0,78	1716	**0,98**	0,86	-0,22	-0,91	0,36	854	**0,92**	0,93	-0,11	-0,42	0,68	1393	**1,00**	0,95	3,00	-0,07	0,11
**PA3629**	42905	5,47	409,18	12	24,83	0,76	-0,40	-1,49	0,14	462	**0,64**	0,76	-0,40	-1,59	0,11	456	**0,53**	0,70	-0,52	-1,81	0,07	344	**0,42**	0,74	3,00	-0,44	0,03
**PA3633**	26456	5,28	212,74	5	26,12	1,14	0,19	0,57	0,57	1253	**0,98**	0,71	-0,50	-1,96	0,05	337	**0,32**	0,80	-0,32	-1,14	0,25	643	**0,81**	0,88	3,00	-0,18	0,23
**PA3635**	49772	5,04	2030,27	35	62,17	0,87	-0,19	-0,76	0,45	1014	**0,96**	0,95	-0,07	-0,37	0,71	1581	**0,97**	0,96	-0,06	-0,25	0,80	1654	**1,00**	0,93	3,00	-0,11	0,05

**PA3636**	34281	6,11	1164,10	29	55,78	1,03	0,05	0,09	0,93	2024	**1,00**	1,11	0,14	0,44	0,66	1475	**0,96**	1,06	0,08	0,21	0,84	1713	**1,00**	1,07	3,00	0,09	0,04
**PA3637**	63171	5,42	2296,37	49	64,40	1,00	0,01	-0,06	0,95	2071	**1,00**	1,02	0,03	0,00	1,00	2156	**1,00**	0,94	-0,09	-0,35	0,73	1500	**1,00**	0,99	3,00	-0,02	0,04
**PA3639**	37224	5,34	1865,39	40	62,39	0,79	-0,34	-1,29	0,20	571	**0,75**	0,79	-0,34	-1,37	0,17	541	**0,69**	0,79	-0,34	-1,19	0,23	616	**0,78**	0,79	3,00	-0,34	0,00
**PA3640**	141219	5,27	1701,50	47	34,21	1,01	0,02	-0,02	0,98	2149	**1,00**	0,97	-0,05	-0,27	0,79	1740	**0,98**	0,96	-0,06	-0,27	0,79	1616	**1,00**	0,98	3,00	-0,03	0,03
**PA3641**	52352	8,29	305,61	9	16,23	0,86	-0,22	-0,85	0,40	928	**0,93**	0,77	-0,38	-1,52	0,13	493	**0,56**	0,97	-0,04	-0,20	0,84	1719	**1,00**	0,87	3,00	-0,21	0,10
**PA3642**	23094	6,11	214,58	5	24,30	0,96	-0,06	-0,28	0,78	1727	**0,98**	0,91	-0,14	-0,62	0,53	1201	**0,96**	0,94	-0,09	-0,38	0,71	1456	**1,00**	0,94	3,00	-0,10	0,03
**PA3643**	42703	9,21	470,28	12	32,55	1,10	0,14	0,42	0,68	1496	**0,98**	0,96	-0,06	-0,31	0,75	1663	**0,98**	1,06	0,08	0,23	0,82	1679	**1,00**	1,04	3,00	0,06	0,07
**PA3644**	29237	6,53	303,66	7	21,99	0,97	-0,05	-0,25	0,80	1772	**0,99**	0,95	-0,07	-0,37	0,71	1583	**0,97**	0,85	-0,24	-0,87	0,39	871	**0,92**	0,92	3,00	-0,12	0,06
**PA3645**	18486	6,96	177,33	7	37,26	1,78	0,83	2,83	0,00	165	**0,06**	0,82	-0,28	-1,14	0,26	679	**0,82**	0,73	-0,46	-1,61	0,11	413	**0,53**	1,11	3,00	0,15	0,58
**PA3646**	37938	5,83	216,49	4	16,40	1,16	0,21	0,67	0,50	1117	**0,98**	1,05	0,07	0,16	0,87	1925	**0,98**	1,09	0,12	0,34	0,73	1512	**1,00**	1,10	3,00	0,14	0,06
**PA3647**	22360	9,38	753,69	19	54,01	0,96	-0,06	-0,28	0,78	1721	**0,99**	0,97	-0,05	-0,28	0,78	1712	**0,98**	0,88	-0,19	-0,69	0,49	1070	**0,95**	0,94	3,00	-0,10	0,05
**PA3648**	93945	5,05	1537,97	31	30,39	0,78	-0,36	-1,34	0,18	540	**0,73**	0,81	-0,31	-1,25	0,21	608	**0,75**	0,75	-0,42	-1,46	0,14	486	**0,61**	0,78	3,00	-0,36	0,03
**PA3649**	50563	6,42	109,22	4	7,55	1,01	0,02	-0,02	0,98	2150	**1,00**	1,13	0,18	0,58	0,56	1256	**0,97**	1,03	0,04	0,06	0,95	1959	**1,00**	1,06	3,00	0,08	0,07
**PA3650**	43576	5,57	442,69	13	32,61	0,90	-0,15	-0,62	0,54	1175	**0,99**	1,01	0,01	-0,07	0,95	2064	**0,99**	0,99	-0,02	-0,13	0,90	1847	**1,00**	0,96	3,00	-0,05	0,06
**PA3652**	29693	7,01	128,83	5	13,24	0,79	-0,35	-1,29	0,20	569	**0,75**	1,00	0,00	-0,11	0,91	2005	**0,99**	0,84	-0,24	-0,89	0,38	851	**0,91**	0,88	3,00	-0,19	0,11
**PA3653**	22778	5,85	1345,10	30	67,48	0,79	-0,33	-1,25	0,21	589	**0,77**	0,78	-0,35	-1,41	0,16	526	**0,65**	0,77	-0,37	-1,32	0,19	549	**0,70**	0,78	3,00	-0,35	0,01
**PA3654**	27979	5,48	473,88	12	43,30	0,71	-0,50	-1,83	0,07	329	**0,45**	0,83	-0,27	-1,12	0,26	691	**0,82**	0,66	-0,60	-2,09	0,04	288	**0,26**	0,73	3,00	-0,45	0,09
**PA3655**	34571	5,22	2982,12	62	85,14	0,86	-0,21	-0,82	0,41	949	**0,94**	0,86	-0,22	-0,91	0,36	857	**0,92**	0,91	-0,14	-0,54	0,59	1232	**0,98**	0,88	3,00	-0,19	0,03
**PA3656**	30103	8,54	1991,68	40	78,06	0,89	-0,17	-0,67	0,50	1115	**0,98**	0,97	-0,05	-0,27	0,79	1738	**0,98**	0,96	-0,06	-0,25	0,80	1653	**1,00**	0,94	3,00	-0,09	0,04
**PA3657**	32082	5,89	340,67	12	36,53	0,84	-0,26	-0,98	0,33	790	**0,90**	0,79	-0,34	-1,38	0,17	538	**0,68**	0,84	-0,25	-0,89	0,37	848	**0,91**	0,82	3,00	-0,28	0,03
**PA3659**	46372	5,53	409,24	11	17,61	0,91	-0,14	-0,58	0,56	1243	**0,98**	0,87	-0,21	-0,86	0,39	904	**0,93**	0,71	-0,49	-1,72	0,09	372	**0,48**	0,83	3,00	-0,27	0,10
**PA3664**	14780	8,54	162,93	5	40,00	0,78	-0,37	-1,37	0,17	525	**0,71**	0,96	-0,06	-0,34	0,74	1632	**0,98**	1,19	0,25	0,78	0,43	954	**0,94**	0,97	3,00	-0,04	0,21
**PA3666**	38394	5,74	846,92	17	39,88	0,75	-0,42	-1,54	0,12	443	**0,60**	0,72	-0,48	-1,89	0,06	360	**0,35**	0,81	-0,30	-1,06	0,29	699	**0,85**	0,76	3,00	-0,40	0,05
**PA3667**	43818	5,87	141,50	3	9,20	1,06	0,09	0,22	0,82	1808	**0,99**	1,02	0,03	0,02	0,98	2134	**1,00**	1,11	0,15	0,44	0,66	1355	**1,00**	1,06	3,00	0,09	0,04
**PA3673**	98976	9,35	245,91	6	12,07	0,92	-0,13	-0,53	0,60	1316	**0,99**	0,81	-0,31	-1,26	0,21	605	**0,74**	0,78	-0,35	-1,25	0,21	580	**0,75**	0,83	3,00	-0,26	0,07
**PA3674**	15295	6,82	292,04	8	45,06	0,70	-0,52	-1,91	0,06	299	**0,41**	0,73	-0,46	-1,82	0,07	386	**0,39**	0,63	-0,67	-2,34	0,02	250	**0,16**	0,68	3,00	-0,55	0,05
**PA3675**	26270	8,92	622,22	13	55,79	0,95	-0,07	-0,34	0,74	1622	**0,99**	0,80	-0,33	-1,31	0,19	566	**0,73**	0,88	-0,19	-0,70	0,49	1058	**0,95**	0,88	3,00	-0,19	0,08
**PA3678**	25193	5,91	79,78	4	11,80	0,92	-0,13	-0,53	0,60	1310	**0,99**	1,01	0,01	-0,04	0,96	2102	**0,99**	0,87	-0,21	-0,76	0,44	978	**0,94**	0,93	3,00	-0,10	0,07
**PA3685**	24690	5,02	114,92	4	16,35	0,83	-0,26	-1,00	0,32	778	**0,89**	0,90	-0,15	-0,64	0,52	1170	**0,96**	0,91	-0,13	-0,50	0,62	1282	**1,00**	0,88	3,00	-0,18	0,04
**PA3686**	25301	5,98	1067,02	23	67,48	0,69	-0,53	-1,95	0,05	288	**0,38**	0,66	-0,61	-2,37	0,02	255	**0,15**	0,69	-0,54	-1,90	0,06	318	**0,37**	0,68	3,00	-0,56	0,02
**PA3687**	100741	5,87	71,33	3	3,70	0,87	-0,20	-0,80	0,43	979	**0,94**	0,80	-0,32	-1,27	0,20	593	**0,74**	0,60	-0,74	-2,55	0,01	216	**0,10**	0,76	3,00	-0,40	0,14
**PA3690**	82028	5,68	510,53	12	19,63	2,16	1,11	3,82	0,00	102	**0,00**	2,02	1,02	3,69	0,00	133	**0,00**	1,90	0,93	3,08	0,00	163	**0,03**	2,03	3,00	1,02	0,13
**PA3692**	30416	9,45	400,25	7	32,11	0,76	-0,39	-1,45	0,15	489	**0,65**	0,74	-0,43	-1,71	0,09	416	**0,46**	0,58	-0,79	-2,72	0,01	203	**0,07**	0,69	3,00	-0,53	0,10
**PA3694**	13015	5,56	133,38	4	10,30	0,90	-0,16	-0,63	0,53	1159	**0,99**	0,96	-0,06	-0,32	0,75	1651	**0,98**	0,99	-0,01	-0,09	0,93	1915	**1,00**	0,95	3,00	-0,07	0,05
**PA3700**	61040	5,23	2293,01	53	60,19	0,93	-0,11	-0,46	0,65	1429	**0,98**	0,95	-0,07	-0,38	0,71	1572	**0,97**	0,88	-0,18	-0,66	0,51	1107	**0,95**	0,92	3,00	-0,12	0,03
**PA3702**	39252	5,55	232,14	5	17,60	1,09	0,12	0,34	0,73	1615	**0,99**	0,75	-0,41	-1,63	0,10	443	**0,50**	0,87	-0,20	-0,75	0,45	999	**0,93**	0,90	3,00	-0,15	0,17
**PA3708**	62063	4,93	441,71	9	17,19	0,84	-0,25	-0,98	0,33	795	**0,90**	0,86	-0,22	-0,92	0,36	850	**0,92**	0,88	-0,18	-0,68	0,50	1085	**0,95**	0,86	3,00	-0,22	0,02
**PA3713**	72225	6,25	851,33	26	40,74	0,64	-0,65	-2,37	0,02	223	**0,18**	0,59	-0,77	-2,96	0,00	184	**0,04**	0,60	-0,73	-2,54	0,01	222	**0,10**	0,61	3,00	-0,72	0,03
**PA3715**	30754	5,46	124,93	4	15,40	1,07	0,10	0,25	0,80	1760	**0,99**	0,89	-0,17	-0,72	0,47	1061	**0,96**	1,01	0,02	0,01	0,99	2048	**1,00**	0,99	3,00	-0,01	0,09
**PA3716**	64823	4,38	1031,42	19	35,84	0,73	-0,46	-1,70	0,09	373	**0,51**	0,71	-0,50	-1,96	0,05	335	**0,32**	0,75	-0,42	-1,48	0,14	474	**0,60**	0,73	3,00	-0,46	0,02
**PA3725**	64799	5,64	204,85	5	7,70	0,82	-0,29	-1,09	0,28	711	**0,85**	0,96	-0,06	-0,31	0,76	1671	**0,98**	0,96	-0,05	-0,24	0,81	1662	**1,00**	0,91	3,00	-0,13	0,08
**PA3726**	21396	5,05	222,64	5	34,18	0,90	-0,14	-0,59	0,56	1229	**0,98**	0,92	-0,12	-0,53	0,59	1321	**0,97**	0,80	-0,33	-1,16	0,24	631	**0,80**	0,87	3,00	-0,19	0,07
**PA3728**	204868	5,13	863,37	26	12,25	0,88	-0,18	-0,73	0,47	1047	**0,97**	0,80	-0,32	-1,29	0,20	578	**0,73**	0,86	-0,22	-0,79	0,43	939	**0,94**	0,85	3,00	-0,24	0,04
**PA3729**	83520	5,18	1891,46	46	44,39	0,80	-0,33	-1,24	0,22	606	**0,78**	0,76	-0,39	-1,55	0,12	478	**0,55**	0,76	-0,39	-1,38	0,17	530	**0,65**	0,77	3,00	-0,37	0,02
**PA3731**	27472	6,67	884,91	18	62,66	0,76	-0,40	-1,47	0,14	472	**0,65**	0,73	-0,46	-1,83	0,07	383	**0,38**	0,72	-0,46	-1,63	0,10	402	**0,53**	0,74	3,00	-0,44	0,02
**PA3732**	16283	4,28	107,01	2	19,00	0,74	-0,42	-1,57	0,12	429	**0,59**	0,75	-0,42	-1,65	0,10	434	**0,49**	0,69	-0,54	-1,87	0,06	329	**0,38**	0,73	3,00	-0,46	0,03
**PA3735**	54344	6,16	1317,70	28	50,32	0,76	-0,40	-1,49	0,14	463	**0,64**	0,70	-0,52	-2,03	0,04	316	**0,29**	0,70	-0,51	-1,79	0,07	350	**0,43**	0,72	3,00	-0,48	0,03
**PA3736**	48797	5,26	1001,11	21	45,70	0,76	-0,39	-1,46	0,15	483	**0,65**	0,74	-0,43	-1,70	0,09	418	**0,46**	0,65	-0,61	-2,13	0,03	283	**0,24**	0,72	3,00	-0,47	0,06

**PA3737**	29144	8,78	313,37	8	31,75	0,88	-0,19	-0,75	0,45	1022	**0,96**	0,88	-0,18	-0,76	0,45	1029	**0,95**	0,89	-0,17	-0,62	0,53	1142	**0,96**	0,88	3,00	-0,18	0,01
**PA3738**	34996	7,92	124,01	4	18,95	0,70	-0,51	-1,88	0,06	305	**0,43**	1,02	0,02	-0,01	0,99	2141	**1,00**	0,97	-0,04	-0,20	0,85	1739	**1,00**	0,90	3,00	-0,16	0,17
**PA3742**	14898	10,40	1032,77	23	68,77	1,00	0,00	-0,08	0,94	2036	**1,00**	0,96	-0,06	-0,34	0,74	1629	**0,98**	0,97	-0,04	-0,19	0,85	1744	**1,00**	0,98	3,00	-0,03	0,02
**PA3743**	29730	5,11	184,91	5	18,96	0,97	-0,05	-0,26	0,80	1748	**0,99**	1,05	0,07	0,18	0,86	1899	**0,98**	1,02	0,03	0,03	0,98	2018	**1,00**	1,01	3,00	0,02	0,04
**PA3744**	21068	4,86	46,99	2	13,70	0,93	-0,10	-0,44	0,66	1458	**0,99**	0,89	-0,16	-0,70	0,48	1084	**0,96**	0,90	-0,16	-0,59	0,55	1183	**0,96**	0,91	3,00	-0,14	0,02
**PA3745**	9919	10,88	256,20	5	69,90	0,85	-0,23	-0,89	0,38	887	**0,92**	0,93	-0,11	-0,49	0,62	1398	**0,96**	0,82	-0,29	-1,03	0,30	734	**0,86**	0,87	3,00	-0,21	0,06
**PA3746**	55813	9,56	887,90	20	38,42	0,77	-0,38	-1,40	0,16	508	**0,69**	0,84	-0,25	-1,02	0,31	778	**0,86**	0,87	-0,21	-0,75	0,45	994	**0,93**	0,83	3,00	-0,27	0,05
**PA3751**	44229	5,80	426,80	13	33,22	0,81	-0,30	-1,12	0,26	684	**0,84**	0,85	-0,24	-0,98	0,33	808	**0,88**	0,76	-0,40	-1,40	0,16	520	**0,64**	0,81	3,00	-0,31	0,04
**PA3763**	147203	5,04	3236,71	72	50,23	1,01	0,01	-0,05	0,96	2099	**1,00**	1,00	0,00	-0,10	0,92	2013	**0,99**	0,92	-0,12	-0,45	0,65	1350	**1,00**	0,98	3,00	-0,03	0,05
**PA3765**	21513	5,97	106,13	3	18,90	0,84	-0,25	-0,98	0,33	796	**0,90**	1,00	0,00	-0,11	0,91	2006	**0,99**	0,72	-0,48	-1,68	0,09	384	**0,50**	0,85	3,00	-0,23	0,14
**PA3768**	53906	6,16	352,51	11	22,03	1,10	0,14	0,42	0,67	1475	**0,99**	0,97	-0,04	-0,24	0,81	1794	**0,98**	0,94	-0,09	-0,36	0,72	1483	**1,00**	1,01	3,00	0,01	0,09
**PA3769**	61801	5,49	1751,60	43	57,81	0,88	-0,19	-0,74	0,46	1030	**0,97**	0,89	-0,17	-0,72	0,47	1058	**0,96**	0,87	-0,20	-0,73	0,46	1024	**0,94**	0,88	3,00	-0,19	0,01
**PA3770**	55514	6,24	3001,45	52	68,37	0,90	-0,15	-0,61	0,54	1198	**0,99**	0,92	-0,12	-0,54	0,59	1315	**0,97**	0,92	-0,12	-0,46	0,64	1319	**1,00**	0,91	3,00	-0,13	0,01
**PA3779**	39691	5,42	212,36	4	12,40	1,99	0,99	3,39	0,00	125	**0,01**	1,97	0,98	3,54	0,00	141	**0,01**	1,62	0,69	2,30	0,02	259	**0,17**	1,86	3,00	0,89	0,21
**PA3785**	18142	6,28	214,81	6	33,97	1,28	0,36	1,18	0,24	640	**0,80**	1,58	0,66	2,36	0,02	260	**0,15**	1,79	0,84	2,79	0,01	193	**0,06**	1,55	3,00	0,63	0,25
**PA3787**	33152	9,87	237,48	6	21,40	0,94	-0,09	-0,41	0,68	1499	**0,98**	1,00	0,01	-0,07	0,94	2048	**0,99**	0,91	-0,14	-0,53	0,60	1248	**0,99**	0,95	3,00	-0,07	0,05
**PA3790**	83859	6,02	274,10	10	14,52	0,82	-0,29	-1,11	0,27	692	**0,84**	0,91	-0,13	-0,59	0,56	1230	**0,98**	0,87	-0,20	-0,74	0,46	1016	**0,93**	0,87	3,00	-0,21	0,05
**PA3792**	69711	5,47	503,17	17	24,70	0,91	-0,14	-0,56	0,58	1277	**0,98**	0,90	-0,15	-0,65	0,52	1157	**0,96**	0,78	-0,37	-1,30	0,19	557	**0,71**	0,86	3,00	-0,21	0,08
**PA3798**	44475	5,81	330,74	8	24,89	0,79	-0,33	-1,25	0,21	599	**0,77**	0,80	-0,32	-1,28	0,20	581	**0,74**	0,66	-0,61	-2,12	0,03	284	**0,24**	0,75	3,00	-0,41	0,08
**PA3799**	59871	9,07	1080,28	25	49,98	0,93	-0,11	-0,46	0,64	1419	**0,98**	0,89	-0,17	-0,73	0,46	1048	**0,96**	0,92	-0,12	-0,46	0,64	1325	**1,00**	0,91	3,00	-0,13	0,02
**PA3800**	42580	5,33	1009,60	19	41,50	0,92	-0,12	-0,50	0,62	1357	**0,99**	1,01	0,01	-0,05	0,96	2086	**0,99**	0,93	-0,11	-0,42	0,67	1385	**1,00**	0,95	3,00	-0,07	0,05
**PA3801**	25832	5,01	950,29	22	66,55	0,98	-0,03	-0,18	0,86	1891	**0,99**	0,92	-0,12	-0,54	0,59	1317	**0,97**	0,98	-0,03	-0,15	0,88	1816	**1,00**	0,96	3,00	-0,06	0,03
**PA3802**	50130	5,27	981,88	26	56,42	1,04	0,05	0,09	0,92	2007	**1,00**	1,02	0,03	0,00	1,00	2161	**1,00**	0,97	-0,04	-0,20	0,85	1733	**1,01**	1,01	3,00	0,01	0,03
**PA3803**	43287	6,04	1330,84	27	46,00	0,90	-0,15	-0,61	0,54	1183	**0,99**	0,98	-0,03	-0,19	0,85	1869	**0,98**	0,94	-0,08	-0,34	0,74	1516	**1,00**	0,94	3,00	-0,09	0,04
**PA3804**	37856	5,11	223,18	6	19,30	1,02	0,03	0,03	0,97	2127	**1,00**	0,87	-0,20	-0,83	0,41	939	**0,93**	0,87	-0,20	-0,74	0,46	1017	**0,93**	0,92	3,00	-0,12	0,09
**PA3805**	30871	6,67	808,04	16	53,80	0,81	-0,31	-1,17	0,24	652	**0,81**	0,91	-0,13	-0,58	0,56	1254	**0,97**	0,84	-0,25	-0,91	0,36	828	**0,90**	0,85	3,00	-0,23	0,05
**PA3806**	45211	8,55	296,63	9	29,03	0,98	-0,03	-0,18	0,86	1884	**0,99**	1,01	0,01	-0,06	0,96	2076	**0,99**	1,04	0,05	0,12	0,90	1855	**1,00**	1,01	3,00	0,01	0,03
**PA3807**	17069	5,48	1219,89	30	91,60	0,91	-0,14	-0,57	0,57	1263	**0,98**	0,94	-0,09	-0,44	0,66	1481	**0,96**	0,87	-0,20	-0,74	0,46	1005	**0,94**	0,91	3,00	-0,14	0,03
**PA3808**	8373	4,35	152,31	4	34,05	1,02	0,03	0,04	0,97	2115	**1,00**	0,93	-0,11	-0,49	0,62	1401	**0,96**	1,02	0,03	0,04	0,97	1986	**1,00**	0,99	3,00	-0,01	0,05
**PA3810**	69327	4,89	1458,51	29	38,37	1,06	0,09	0,24	0,81	1784	**0,99**	1,04	0,06	0,13	0,89	1969	**0,98**	1,05	0,08	0,20	0,84	1726	**1,00**	1,05	3,00	0,08	0,01
**PA3812**	12564	4,85	288,64	7	29,90	1,03	0,04	0,07	0,94	2044	**1,00**	1,12	0,16	0,49	0,62	1403	**0,96**	1,00	-0,01	-0,08	0,94	1930	**1,00**	1,05	3,00	0,07	0,06
**PA3813**	15899	5,69	698,15	14	62,10	0,91	-0,14	-0,56	0,58	1273	**0,98**	0,89	-0,17	-0,71	0,48	1073	**0,96**	0,99	-0,01	-0,10	0,92	1894	**1,00**	0,93	3,00	-0,10	0,05
**PA3814**	47803	5,69	1508,50	37	53,32	0,90	-0,16	-0,63	0,53	1167	**0,99**	0,93	-0,11	-0,51	0,61	1355	**0,97**	0,87	-0,21	-0,75	0,45	993	**0,94**	0,90	3,00	-0,16	0,03
**PA3815**	18792	6,36	289,71	7	37,79	1,04	0,06	0,13	0,90	1971	**0,99**	1,10	0,14	0,41	0,68	1519	**0,97**	0,97	-0,05	-0,22	0,82	1687	**1,01**	1,04	3,00	0,05	0,07
**PA3816**	29596	6,07	346,78	6	22,90	0,87	-0,21	-0,81	0,42	964	**0,94**	0,89	-0,16	-0,71	0,48	1075	**0,96**	0,87	-0,20	-0,75	0,45	1001	**0,93**	0,88	3,00	-0,19	0,01
**PA3817**	29957	5,86	462,76	10	31,52	0,95	-0,07	-0,33	0,74	1634	**0,99**	0,83	-0,27	-1,11	0,27	705	**0,82**	0,85	-0,24	-0,87	0,38	867	**0,91**	0,88	3,00	-0,19	0,07
**PA3818**	31180	6,18	896,09	24	51,01	0,88	-0,19	-0,74	0,46	1032	**0,97**	0,77	-0,38	-1,52	0,13	494	**0,56**	0,74	-0,43	-1,51	0,13	459	**0,59**	0,80	3,00	-0,33	0,07
**PA3819**	20894	9,08	116,24	2	33,00	0,70	-0,51	-1,86	0,06	309	**0,44**	0,78	-0,36	-1,44	0,15	510	**0,63**	0,95	-0,07	-0,28	0,78	1599	**1,00**	0,81	3,00	-0,30	0,13
**PA3820**	34462	4,81	460,60	7	24,61	0,94	-0,08	-0,37	0,71	1581	**0,98**	0,93	-0,10	-0,49	0,63	1406	**0,96**	0,90	-0,15	-0,58	0,56	1203	**0,96**	0,92	3,00	-0,11	0,02
**PA3821**	72099	8,88	1809,02	39	41,44	0,87	-0,20	-0,80	0,43	980	**0,94**	0,83	-0,27	-1,11	0,27	701	**0,82**	0,87	-0,20	-0,74	0,46	1011	**0,93**	0,86	3,00	-0,23	0,02
**PA3822**	13440	8,96	478,26	10	42,00	0,80	-0,32	-1,21	0,23	622	**0,79**	0,79	-0,35	-1,39	0,16	533	**0,66**	0,89	-0,16	-0,61	0,54	1165	**0,96**	0,83	3,00	-0,28	0,06
**PA3823**	44048	6,40	781,08	17	49,55	0,99	-0,01	-0,12	0,90	1975	**0,99**	0,95	-0,07	-0,36	0,72	1604	**0,97**	0,94	-0,09	-0,38	0,71	1452	**1,00**	0,96	3,00	-0,06	0,03
**PA3824**	39191	6,02	322,47	8	27,10	0,86	-0,21	-0,82	0,41	957	**0,94**	1,08	0,11	0,30	0,76	1680	**0,98**	0,86	-0,21	-0,78	0,44	964	**0,93**	0,93	3,00	-0,10	0,12
**PA3827**	40420	9,09	161,00	7	15,91	0,91	-0,13	-0,54	0,59	1295	**0,99**	1,08	0,11	0,30	0,76	1683	**0,98**	0,94	-0,09	-0,36	0,72	1474	**1,00**	0,98	3,00	-0,03	0,09
**PA3828**	43016	9,45	280,54	7	15,17	0,92	-0,12	-0,50	0,62	1358	**0,99**	0,92	-0,11	-0,52	0,60	1344	**0,97**	1,00	-0,01	-0,08	0,94	1936	**1,00**	0,95	3,00	-0,08	0,04
**PA3831**	58195	8,68	1547,19	31	47,91	0,79	-0,34	-1,26	0,21	585	**0,77**	0,78	-0,35	-1,41	0,16	527	**0,66**	0,75	-0,41	-1,46	0,14	488	**0,61**	0,78	3,00	-0,37	0,02
**PA3832**	16624	5,58	158,26	2	29,60	0,90	-0,16	-0,63	0,53	1168	**0,99**	1,07	0,10	0,26	0,80	1756	**0,98**	0,86	-0,22	-0,81	0,42	925	**0,93**	0,94	3,00	-0,09	0,11
**PA3834**	115219	5,36	2140,06	56	50,16	0,84	-0,26	-0,98	0,33	791	**0,90**	0,85	-0,23	-0,94	0,35	838	**0,90**	0,82	-0,30	-1,06	0,29	703	**0,85**	0,84	3,00	-0,26	0,02

**PA3836**	38378	7,75	889,60	16	43,65	1,50	0,58	1,96	0,05	285	**0,38**	1,76	0,81	2,93	0,00	185	**0,04**	1,70	0,77	2,55	0,01	217	**0,10**	1,65	3,00	0,73	0,14
**PA3838**	29913	6,45	473,25	12	34,13	1,30	0,38	1,25	0,21	597	**0,77**	1,55	0,63	2,25	0,02	276	**0,19**	1,37	0,46	1,50	0,13	465	**0,59**	1,41	3,00	0,49	0,13
**PA3842**	13529	5,04	91,16	2	15,50	0,54	-0,89	-3,19	0,00	136	**0,02**	0,69	-0,54	-2,10	0,04	304	**0,25**	0,42	-1,25	-4,31	0,00	103	**0,00**	0,55	3,00	-0,86	0,14
**PA3848**	52966	7,90	354,16	8	17,65	0,72	-0,48	-1,76	0,08	352	**0,48**	0,81	-0,30	-1,21	0,23	625	**0,78**	0,91	-0,14	-0,53	0,60	1245	**0,99**	0,81	3,00	-0,30	0,10
**PA3849**	40835	5,24	567,16	14	32,26	0,84	-0,25	-0,95	0,34	820	**0,91**	0,76	-0,40	-1,59	0,11	460	**0,53**	0,87	-0,20	-0,74	0,46	1007	**0,94**	0,82	3,00	-0,28	0,06
**PA3859**	24173	5,02	206,49	4	18,15	0,71	-0,50	-1,83	0,07	326	**0,45**	0,80	-0,32	-1,28	0,20	589	**0,74**	0,84	-0,25	-0,89	0,37	850	**0,91**	0,78	3,00	-0,35	0,07
**PA3861**	47104	5,93	749,29	19	47,33	1,00	0,00	-0,08	0,94	2037	**1,00**	1,02	0,02	-0,01	0,99	2140	**1,00**	1,02	0,03	0,04	0,97	1996	**1,00**	1,01	3,00	0,02	0,01
**PA3862**	34730	5,58	389,39	7	34,09	0,99	-0,02	-0,14	0,89	1956	**0,99**	0,91	-0,14	-0,63	0,53	1188	**0,96**	1,02	0,03	0,06	0,96	1971	**1,00**	0,97	3,00	-0,04	0,06
**PA3865**	31246	6,02	164,02	4	17,90	0,91	-0,13	-0,54	0,59	1296	**0,99**	1,00	0,01	-0,08	0,94	2047	**0,99**	1,38	0,46	1,50	0,13	462	**0,59**	1,10	3,00	0,13	0,25
**PA3866**	86735	8,26	86,27	3	5,50	0,75	-0,41	-1,53	0,13	446	**0,61**	0,59	-0,76	-2,92	0,00	188	**0,04**	0,93	-0,11	-0,43	0,66	1366	**1,00**	0,76	3,00	-0,40	0,17
**PA3886**	32651	5,12	78,78	2	6,70	0,87	-0,20	-0,79	0,43	985	**0,95**	0,78	-0,35	-1,41	0,16	524	**0,65**	0,83	-0,28	-1,00	0,32	761	**0,86**	0,83	3,00	-0,28	0,04
**PA3887**	46368	6,08	63,40	2	2,80	0,62	-0,70	-2,53	0,01	201	**0,13**	0,55	-0,85	-3,28	0,00	156	**0,01**	0,47	-1,07	-3,70	0,00	126	**0,00**	0,55	3,00	-0,87	0,07
**PA3892**	35405	5,92	230,56	5	19,90	2,17	1,12	3,83	0,00	101	**0,00**	1,44	0,53	1,87	0,06	365	**0,36**	2,27	1,18	3,94	0,00	115	**0,00**	1,96	3,00	0,97	0,45
**PA3894**	55574	5,78	262,10	6	13,43	1,86	0,90	3,06	0,00	147	**0,03**	1,63	0,70	2,53	0,01	225	**0,11**	1,30	0,38	1,23	0,22	587	**0,77**	1,60	3,00	0,68	0,28
**PA3895**	36179	5,47	191,85	5	10,38	0,90	-0,16	-0,64	0,52	1143	**0,99**	0,77	-0,38	-1,51	0,13	497	**0,58**	0,95	-0,07	-0,28	0,78	1601	**1,00**	0,87	3,00	-0,20	0,09
**PA3896**	37575	7,81	209,52	6	18,12	0,96	-0,06	-0,29	0,77	1704	**0,99**	0,94	-0,08	-0,41	0,68	1516	**0,97**	0,89	-0,16	-0,60	0,55	1176	**0,96**	0,93	3,00	-0,10	0,03
**PA3901**	88534	5,48	1491,75	33	35,19	2,63	1,39	4,81	0,00	75	**0,00**	2,70	1,43	5,24	0,00	77	**0,00**	2,38	1,25	4,18	0,00	108	**0,00**	2,57	3,00	1,36	0,17
**PA3903**	65337	5,60	1162,06	26	43,75	0,90	-0,15	-0,59	0,55	1219	**0,99**	0,93	-0,10	-0,46	0,64	1439	**0,96**	0,95	-0,08	-0,31	0,75	1555	**1,00**	0,93	3,00	-0,11	0,02
**PA3908**	28089	5,20	265,21	6	25,20	0,80	-0,32	-1,20	0,23	627	**0,79**	0,75	-0,41	-1,64	0,10	442	**0,50**	0,88	-0,19	-0,70	0,48	1055	**0,94**	0,81	3,00	-0,30	0,06
**PA3918**	18730	6,58	148,14	7	36,34	0,89	-0,17	-0,68	0,49	1098	**0,98**	1,02	0,03	0,03	0,98	2130	**0,99**	0,85	-0,24	-0,87	0,38	864	**0,91**	0,92	3,00	-0,12	0,09
**PA3925**	43352	6,03	370,11	7	23,50	1,59	0,67	2,27	0,02	240	**0,21**	1,86	0,89	3,23	0,00	159	**0,02**	1,98	0,98	3,28	0,00	148	**0,01**	1,81	3,00	0,85	0,20
**PA3931**	31975	7,74	148,91	4	24,35	1,17	0,22	0,70	0,49	1088	**0,97**	1,22	0,29	0,99	0,32	803	**0,87**	2,16	1,11	3,70	0,00	124	**0,00**	1,52	3,00	0,60	0,56
**PA3934**	72157	5,34	148,75	4	5,73	0,96	-0,06	-0,29	0,77	1694	**0,99**	0,91	-0,14	-0,63	0,53	1193	**0,96**	1,00	0,00	-0,06	0,95	1968	**1,00**	0,95	3,00	-0,07	0,05
**PA3940**	11327	8,01	654,51	13	72,85	0,80	-0,32	-1,19	0,23	634	**0,80**	0,90	-0,15	-0,65	0,52	1165	**0,96**	0,92	-0,12	-0,47	0,64	1314	**1,00**	0,88	3,00	-0,19	0,06
**PA3948**	24803	6,30	136,41	4	18,20	0,98	-0,02	-0,16	0,87	1917	**0,99**	0,65	-0,63	-2,44	0,01	245	**0,13**	0,73	-0,45	-1,60	0,11	416	**0,54**	0,79	3,00	-0,35	0,18
**PA3950**	53130	9,94	444,70	12	21,45	1,04	0,06	0,11	0,91	1981	**1,00**	0,96	-0,06	-0,33	0,75	1650	**0,98**	0,84	-0,25	-0,90	0,37	836	**0,91**	0,95	3,00	-0,08	0,10
**PA3961**	94412	6,97	127,04	5	7,36	1,01	0,01	-0,04	0,96	2103	**1,00**	0,91	-0,14	-0,62	0,54	1203	**0,96**	0,98	-0,03	-0,15	0,88	1806	**1,00**	0,97	3,00	-0,05	0,05
**PA3965**	20712	5,63	213,32	7	39,66	0,94	-0,09	-0,39	0,69	1531	**0,99**	0,96	-0,06	-0,34	0,74	1630	**0,98**	0,80	-0,32	-1,13	0,26	648	**0,82**	0,90	3,00	-0,15	0,08
**PA3967**	15554	7,77	203,53	5	30,34	0,75	-0,42	-1,57	0,12	431	**0,59**	0,74	-0,44	-1,73	0,08	409	**0,44**	0,85	-0,24	-0,86	0,39	877	**0,92**	0,78	3,00	-0,36	0,06
**PA3970**	57960	6,36	500,19	12	19,50	0,94	-0,10	-0,42	0,68	1494	**0,99**	0,78	-0,36	-1,43	0,15	520	**0,64**	0,81	-0,31	-1,10	0,27	663	**0,84**	0,84	3,00	-0,25	0,08
**PA3972**	63164	6,01	312,30	8	15,08	0,88	-0,18	-0,72	0,47	1054	**0,97**	0,93	-0,11	-0,50	0,61	1378	**0,96**	0,98	-0,04	-0,18	0,86	1774	**1,00**	0,93	3,00	-0,11	0,05
**PA3975**	28905	5,81	168,50	5	15,46	0,99	-0,02	-0,14	0,89	1946	**0,99**	1,06	0,08	0,20	0,84	1856	**0,98**	0,96	-0,07	-0,28	0,78	1607	**1,00**	1,00	3,00	0,00	0,05
**PA3976**	22801	6,52	217,95	5	24,70	1,06	0,08	0,20	0,84	1845	**0,99**	1,00	0,00	-0,11	0,91	1997	**0,99**	0,94	-0,08	-0,34	0,73	1505	**1,00**	1,00	3,00	0,00	0,06
**PA3977**	47904	5,47	1615,14	30	54,16	0,85	-0,23	-0,90	0,37	879	**0,91**	0,91	-0,13	-0,58	0,56	1245	**0,97**	0,87	-0,20	-0,73	0,46	1021	**0,94**	0,88	3,00	-0,19	0,03
**PA3979**	13913	5,22	284,55	10	55,20	0,80	-0,32	-1,19	0,24	639	**0,80**	0,90	-0,16	-0,69	0,49	1105	**0,96**	0,83	-0,27	-0,97	0,33	784	**0,87**	0,84	3,00	-0,25	0,05
**PA3980**	53341	5,62	394,69	10	28,38	0,79	-0,34	-1,27	0,20	580	**0,76**	0,76	-0,39	-1,55	0,12	479	**0,55**	0,80	-0,33	-1,17	0,24	628	**0,79**	0,78	3,00	-0,35	0,02
**PA3981**	40637	6,69	384,84	10	32,10	1,01	0,01	-0,05	0,96	2093	**1,00**	1,09	0,12	0,35	0,73	1617	**0,97**	1,05	0,06	0,16	0,87	1795	**1,00**	1,05	3,00	0,07	0,04
**PA3983**	33114	4,81	315,81	8	35,25	1,18	0,24	0,75	0,45	1019	**0,96**	1,28	0,36	1,24	0,21	613	**0,76**	1,21	0,28	0,87	0,38	863	**0,91**	1,22	3,00	0,29	0,05
**PA3984**	57514	9,17	482,27	13	18,85	0,84	-0,25	-0,97	0,33	801	**0,90**	0,98	-0,03	-0,21	0,83	1844	**0,98**	0,82	-0,29	-1,03	0,30	730	**0,86**	0,88	3,00	-0,19	0,09
**PA3987**	104478	5,46	1929,10	43	40,43	0,86	-0,22	-0,87	0,39	908	**0,92**	0,85	-0,23	-0,96	0,34	818	**0,89**	0,87	-0,21	-0,75	0,45	992	**0,94**	0,86	3,00	-0,22	0,01
**PA3988**	24069	5,23	731,00	15	51,02	0,82	-0,28	-1,07	0,28	717	**0,86**	0,80	-0,32	-1,31	0,19	567	**0,73**	0,72	-0,47	-1,66	0,10	389	**0,51**	0,78	3,00	-0,36	0,05
**PA3989**	39655	6,47	251,71	4	20,00	1,21	0,27	0,88	0,38	891	**0,92**	0,86	-0,22	-0,90	0,37	863	**0,92**	0,94	-0,09	-0,36	0,72	1471	**1,00**	1,00	3,00	0,00	0,18
**PA3992**	49697	7,81	364,18	9	20,10	0,94	-0,09	-0,41	0,68	1504	**0,99**	0,70	-0,51	-1,98	0,05	330	**0,31**	0,72	-0,48	-1,69	0,09	377	**0,49**	0,79	3,00	-0,35	0,13
**PA3996**	40058	6,43	590,12	15	35,82	0,96	-0,05	-0,26	0,79	1745	**0,99**	0,86	-0,22	-0,92	0,36	849	**0,92**	0,92	-0,12	-0,45	0,65	1343	**1,00**	0,92	3,00	-0,13	0,05
**PA3997**	24844	5,74	84,43	2	20,30	0,94	-0,09	-0,41	0,68	1509	**0,99**	1,03	0,04	0,05	0,96	2093	**0,99**	0,83	-0,27	-0,98	0,33	778	**0,87**	0,93	3,00	-0,10	0,10
**PA3998**	10954	4,79	184,35	5	52,70	1,22	0,28	0,91	0,36	861	**0,91**	0,94	-0,08	-0,41	0,69	1532	**0,97**	0,92	-0,12	-0,48	0,63	1300	**1,00**	1,03	3,00	0,04	0,17
**PA3999**	45503	6,27	1228,59	27	57,50	0,90	-0,16	-0,63	0,53	1169	**0,99**	0,89	-0,17	-0,72	0,47	1059	**0,96**	0,92	-0,12	-0,45	0,65	1337	**1,00**	0,90	3,00	-0,15	0,02
**PA4000**	38840	9,69	228,32	4	15,05	0,89	-0,17	-0,67	0,50	1122	**0,98**	1,04	0,05	0,10	0,92	2019	**0,99**	0,98	-0,02	-0,14	0,89	1827	**1,00**	0,97	3,00	-0,04	0,07

**PA4001**	39397	5,67	268,24	9	24,34	0,87	-0,20	-0,78	0,43	995	**0,95**	1,03	0,04	0,06	0,95	2068	**0,99**	1,04	0,05	0,11	0,91	1873	**1,00**	0,98	3,00	-0,03	0,09
**PA4003**	76203	7,88	136,29	4	4,95	0,92	-0,12	-0,51	0,61	1346	**0,99**	0,90	-0,15	-0,64	0,52	1177	**0,96**	0,79	-0,34	-1,22	0,22	595	**0,77**	0,87	3,00	-0,20	0,07
**PA4004**	18402	8,97	321,19	9	46,02	0,90	-0,15	-0,62	0,54	1176	**0,99**	0,95	-0,07	-0,35	0,73	1621	**0,97**	0,89	-0,16	-0,60	0,55	1175	**0,96**	0,92	3,00	-0,13	0,03
**PA4005**	14100	4,59	213,36	6	32,80	0,92	-0,13	-0,53	0,60	1311	**0,99**	1,03	0,05	0,07	0,94	2058	**0,99**	1,02	0,03	0,03	0,97	2008	**1,00**	0,99	3,00	-0,02	0,06
**PA4007**	46930	5,33	832,70	19	40,79	0,85	-0,24	-0,92	0,36	854	**0,91**	0,87	-0,19	-0,82	0,41	948	**0,94**	0,83	-0,27	-0,96	0,34	789	**0,88**	0,85	3,00	-0,23	0,02
**PA4010**	28368	8,77	112,01	4	22,60	0,93	-0,11	-0,47	0,64	1411	**0,98**	1,00	0,01	-0,07	0,94	2050	**0,99**	0,98	-0,03	-0,14	0,89	1824	**1,00**	0,97	3,00	-0,04	0,04
**PA4011**	47630	10,15	113,25	3	5,30	1,05	0,06	0,15	0,88	1945	**0,99**	1,00	0,01	-0,08	0,94	2045	**0,99**	1,23	0,30	0,97	0,33	786	**0,87**	1,09	3,00	0,13	0,12
**PA4015**	18223	5,54	143,18	4	33,80	0,96	-0,06	-0,30	0,77	1686	**0,99**	0,86	-0,22	-0,92	0,36	848	**0,91**	0,95	-0,07	-0,28	0,78	1600	**1,00**	0,92	3,00	-0,11	0,06
**PA4017**	25017	6,54	383,35	9	24,60	0,74	-0,44	-1,64	0,10	401	**0,55**	0,88	-0,19	-0,81	0,42	958	**0,94**	0,92	-0,12	-0,46	0,65	1333	**1,00**	0,84	3,00	-0,24	0,10
**PA4019**	23303	5,08	57,95	3	9,40	0,82	-0,29	-1,10	0,27	698	**0,84**	0,89	-0,16	-0,71	0,48	1083	**0,96**	1,08	0,11	0,32	0,75	1543	**1,00**	0,93	3,00	-0,10	0,14
**PA4020**	50352	5,97	662,39	13	29,86	0,96	-0,06	-0,28	0,78	1722	**0,98**	0,86	-0,21	-0,88	0,38	881	**0,93**	0,89	-0,17	-0,63	0,53	1141	**0,96**	0,91	3,00	-0,14	0,05
**PA4022**	58869	5,50	1260,94	24	41,60	3,49	1,80	6,25	0,00	45	**0,00**	3,92	1,97	7,25	0,00	40	**0,00**	5,62	2,49	8,38	0,00	2	**0,00**	4,34	3,00	2,12	1,12
**PA4029**	25814	9,45	121,34	3	11,47	0,97	-0,05	-0,24	0,81	1778	**0,99**	1,12	0,16	0,50	0,62	1383	**0,96**	0,99	-0,01	-0,09	0,93	1917	**1,00**	1,03	3,00	0,04	0,08
**PA4031**	21736	5,04	589,29	16	50,97	0,64	-0,65	-2,37	0,02	224	**0,17**	0,81	-0,30	-1,23	0,22	618	**0,76**	0,86	-0,22	-0,81	0,42	924	**0,93**	0,77	3,00	-0,38	0,12
**PA4035**	38127	5,93	224,99	5	13,56	0,94	-0,09	-0,39	0,69	1532	**0,98**	0,83	-0,27	-1,11	0,27	702	**0,82**	0,67	-0,58	-2,03	0,04	296	**0,29**	0,81	3,00	-0,30	0,14
**PA4042**	9590	4,24	191,06	5	41,80	0,82	-0,29	-1,09	0,28	708	**0,85**	0,67	-0,58	-2,24	0,02	278	**0,19**	0,83	-0,27	-0,97	0,33	781	**0,87**	0,77	3,00	-0,37	0,09
**PA4043**	32638	5,22	339,28	8	26,39	0,78	-0,37	-1,36	0,17	531	**0,71**	0,76	-0,40	-1,59	0,11	458	**0,53**	0,82	-0,29	-1,03	0,30	724	**0,86**	0,78	3,00	-0,35	0,03
**PA4044**	71937	5,47	828,38	22	27,35	0,80	-0,32	-1,19	0,23	637	**0,80**	0,93	-0,10	-0,47	0,64	1438	**0,97**	0,81	-0,30	-1,07	0,28	688	**0,85**	0,85	3,00	-0,23	0,07
**PA4047**	23678	6,18	278,76	8	25,78	0,86	-0,21	-0,83	0,41	940	**0,94**	0,95	-0,07	-0,35	0,73	1618	**0,97**	0,97	-0,04	-0,18	0,86	1767	**1,00**	0,93	3,00	-0,10	0,06
**PA4052**	19295	8,03	173,46	4	29,60	1,19	0,25	0,81	0,42	972	**0,94**	0,90	-0,14	-0,64	0,52	1181	**0,96**	0,98	-0,02	-0,14	0,89	1836	**1,00**	1,03	3,00	0,04	0,15
**PA4053**	17602	5,69	737,93	16	65,36	0,89	-0,16	-0,65	0,51	1135	**0,98**	0,94	-0,08	-0,41	0,68	1526	**0,97**	0,95	-0,08	-0,32	0,75	1546	**1,00**	0,93	3,00	-0,11	0,03
**PA4054**	41130	5,23	1171,47	23	60,12	0,98	-0,02	-0,16	0,87	1921	**0,99**	0,97	-0,04	-0,25	0,80	1764	**0,98**	0,98	-0,02	-0,14	0,89	1832	**1,00**	0,98	3,00	-0,03	0,01
**PA4055**	24941	5,98	336,52	8	33,80	1,09	0,13	0,37	0,71	1572	**0,98**	1,12	0,16	0,51	0,61	1366	**0,96**	1,12	0,16	0,48	0,63	1294	**1,00**	1,11	3,00	0,15	0,01
**PA4056**	40788	6,77	229,75	7	20,34	0,85	-0,24	-0,91	0,36	860	**0,91**	0,90	-0,16	-0,69	0,49	1111	**0,96**	1,00	0,00	-0,07	0,95	1950	**1,00**	0,91	3,00	-0,13	0,08
**PA4057**	19395	7,71	167,38	5	28,34	0,81	-0,31	-1,17	0,24	651	**0,81**	0,82	-0,28	-1,16	0,25	661	**0,81**	0,81	-0,30	-1,09	0,28	675	**0,84**	0,81	3,00	-0,30	0,01
**PA4058**	17412	9,97	401,32	10	47,98	0,88	-0,18	-0,70	0,48	1086	**0,97**	1,11	0,15	0,47	0,64	1434	**0,96**	1,03	0,05	0,11	0,91	1876	**1,00**	1,01	3,00	0,01	0,12
**PA4061**	33916	4,63	950,79	20	62,76	0,73	-0,46	-1,68	0,09	385	**0,53**	0,83	-0,27	-1,12	0,26	699	**0,82**	0,85	-0,23	-0,84	0,40	889	**0,92**	0,80	3,00	-0,32	0,06
**PA4067**	27483	4,85	483,40	14	35,30	0,54	-0,88	-3,16	0,00	138	**0,02**	0,74	-0,44	-1,74	0,08	405	**0,44**	0,73	-0,45	-1,57	0,12	431	**0,56**	0,67	3,00	-0,57	0,11
**PA4068**	34535	5,57	379,54	8	29,65	0,82	-0,29	-1,12	0,26	686	**0,84**	0,77	-0,38	-1,52	0,13	495	**0,57**	0,75	-0,42	-1,49	0,14	468	**0,60**	0,78	3,00	-0,37	0,04
**PA4069**	33968	5,93	103,04	3	13,03	0,90	-0,15	-0,62	0,53	1171	**0,99**	0,74	-0,44	-1,74	0,08	406	**0,44**	1,07	0,09	0,26	0,79	1629	**1,00**	0,90	3,00	-0,15	0,17
**PA4079**	24684	5,27	204,22	5	17,72	1,05	0,06	0,15	0,88	1944	**0,99**	0,74	-0,43	-1,69	0,09	421	**0,46**	0,99	-0,02	-0,13	0,90	1842	**1,00**	0,92	3,00	-0,11	0,16
**PA4115**	54066	6,63	472,29	11	21,95	0,71	-0,50	-1,84	0,07	322	**0,45**	0,63	-0,68	-2,62	0,01	219	**0,09**	0,63	-0,67	-2,31	0,02	255	**0,17**	0,65	3,00	-0,61	0,05
**PA4138**	46408	6,29	423,13	10	34,32	0,91	-0,13	-0,55	0,58	1284	**0,98**	1,01	0,01	-0,05	0,96	2082	**0,99**	0,92	-0,11	-0,44	0,66	1356	**1,00**	0,95	3,00	-0,08	0,05
**PA4154**	25595	9,51	54,56	2	11,30	1,07	0,10	0,27	0,79	1738	**0,99**	1,06	0,09	0,23	0,82	1807	**0,98**	1,20	0,26	0,83	0,41	907	**0,92**	1,11	3,00	0,15	0,08
**PA4163**	62324	4,93	632,35	14	24,00	0,84	-0,25	-0,95	0,34	821	**0,91**	0,97	-0,04	-0,25	0,80	1767	**0,98**	0,88	-0,19	-0,69	0,49	1066	**0,95**	0,90	3,00	-0,16	0,07
**PA4176**	10625	5,87	676,82	11	71,77	0,73	-0,45	-1,67	0,09	389	**0,53**	0,79	-0,34	-1,35	0,18	551	**0,70**	0,69	-0,54	-1,88	0,06	325	**0,38**	0,74	3,00	-0,44	0,05
**PA4179**	49911	8,92	70,54	2	6,25	0,35	-1,51	-5,37	0,00	70	**0,00**	0,30	-1,71	-6,49	0,00	52	**0,00**	0,25	-2,03	-6,93	0,00	49	**0,00**	0,30	3,00	-1,73	0,05
**PA4198**	62374	5,77	84,86	2	6,70	1,67	0,74	2,52	0,01	203	**0,13**	1,38	0,47	1,65	0,10	435	**0,49**	2,46	1,30	4,34	0,00	101	**0,00**	1,84	3,00	0,88	0,55
**PA4199**	66612	5,22	298,03	8	20,80	0,91	-0,14	-0,58	0,56	1240	**0,98**	0,97	-0,04	-0,25	0,80	1766	**0,98**	0,97	-0,05	-0,21	0,83	1701	**1,01**	0,95	3,00	-0,08	0,04
**PA4200**	32901	5,46	97,08	3	9,17	0,73	-0,46	-1,69	0,09	383	**0,52**	0,83	-0,26	-1,08	0,28	727	**0,83**	0,82	-0,29	-1,05	0,29	709	**0,85**	0,79	3,00	-0,34	0,06
**PA4202**	37906	7,77	291,41	9	27,80	0,88	-0,19	-0,73	0,46	1039	**0,97**	0,87	-0,20	-0,83	0,41	937	**0,94**	1,00	0,00	-0,07	0,95	1957	**1,00**	0,92	3,00	-0,13	0,07
**PA4232**	19411	5,46	398,56	9	51,81	0,69	-0,54	-1,97	0,05	282	**0,38**	0,70	-0,51	-1,99	0,05	328	**0,31**	0,73	-0,45	-1,60	0,11	421	**0,54**	0,71	3,00	-0,50	0,02
**PA4233**	50365	9,19	128,68	2	7,80	0,91	-0,14	-0,58	0,56	1248	**0,98**	0,92	-0,12	-0,53	0,60	1322	**0,97**	1,04	0,05	0,12	0,91	1864	**1,00**	0,96	3,00	-0,07	0,07
**PA4234**	111763	6,20	1151,29	30	24,20	0,80	-0,31	-1,18	0,24	642	**0,80**	0,82	-0,29	-1,16	0,25	653	**0,81**	0,81	-0,31	-1,10	0,27	668	**0,84**	0,81	3,00	-0,30	0,01
**PA4235**	18840	5,01	477,54	9	55,82	0,90	-0,15	-0,60	0,55	1211	**0,99**	0,94	-0,10	-0,46	0,65	1448	**0,97**	1,18	0,24	0,76	0,44	981	**0,93**	1,01	3,00	0,01	0,15
**PA4236**	58771	6,21	354,61	7	19,30	0,70	-0,51	-1,86	0,06	308	**0,44**	0,97	-0,04	-0,23	0,82	1805	**0,98**	0,94	-0,09	-0,37	0,71	1466	**1,00**	0,87	3,00	-0,20	0,15
**PA4237**	16126	10,45	954,79	23	66,70	0,85	-0,24	-0,91	0,36	862	**0,91**	0,93	-0,10	-0,48	0,63	1425	**0,96**	1,00	0,00	-0,07	0,95	1949	**1,00**	0,93	3,00	-0,11	0,07
**PA4238**	39313	4,88	1536,35	39	58,15	0,96	-0,06	-0,28	0,78	1712	**0,99**	0,90	-0,15	-0,66	0,51	1133	**0,97**	0,81	-0,30	-1,06	0,29	698	**0,85**	0,89	3,00	-0,17	0,07

**PA4239**	25897	9,94	1260,29	30	67,32	0,93	-0,10	-0,44	0,66	1454	**0,99**	0,96	-0,05	-0,30	0,77	1687	**0,98**	0,95	-0,08	-0,33	0,74	1528	**1,00**	0,95	3,00	-0,08	0,02
**PA4240**	15442	10,83	780,23	16	78,30	0,99	-0,01	-0,11	0,91	1984	**1,00**	0,90	-0,15	-0,65	0,51	1155	**0,96**	1,00	0,01	-0,03	0,97	2002	**1,00**	0,97	3,00	-0,05	0,06
**PA4241**	14790	10,94	1073,63	28	71,20	0,98	-0,02	-0,16	0,87	1916	**0,99**	0,93	-0,10	-0,47	0,64	1437	**0,96**	1,00	0,00	-0,07	0,94	1940	**1,00**	0,97	3,00	-0,04	0,03
**PA4243**	49911	9,83	290,73	10	19,35	0,94	-0,09	-0,38	0,70	1551	**0,98**	0,96	-0,06	-0,33	0,74	1637	**0,98**	1,03	0,04	0,08	0,93	1924	**1,00**	0,98	3,00	-0,04	0,05
**PA4244**	17039	10,96	1114,18	27	70,30	0,92	-0,12	-0,51	0,61	1337	**0,99**	0,95	-0,08	-0,40	0,69	1539	**0,97**	0,94	-0,09	-0,35	0,73	1501	**1,00**	0,94	3,00	-0,10	0,01
**PA4245**	7384	10,12	180,50	4	49,10	0,85	-0,23	-0,87	0,38	897	**0,93**	0,93	-0,10	-0,48	0,63	1417	**0,96**	0,95	-0,08	-0,31	0,75	1559	**1,00**	0,91	3,00	-0,13	0,05
**PA4246**	19822	9,95	1824,64	34	75,90	0,91	-0,14	-0,58	0,56	1236	**0,98**	0,91	-0,13	-0,59	0,55	1225	**0,97**	0,98	-0,03	-0,14	0,89	1822	**1,00**	0,93	3,00	-0,10	0,04
**PA4247**	14429	10,23	900,72	20	68,00	0,90	-0,16	-0,64	0,52	1144	**0,99**	0,97	-0,05	-0,27	0,78	1728	**0,98**	0,91	-0,13	-0,50	0,62	1281	**1,00**	0,93	3,00	-0,11	0,04
**PA4248**	21681	9,87	1069,91	20	65,74	0,96	-0,06	-0,31	0,76	1671	**0,99**	0,98	-0,02	-0,18	0,86	1887	**0,98**	1,00	0,00	-0,07	0,95	1955	**1,00**	0,98	3,00	-0,03	0,02
**PA4249**	16081	9,59	722,76	17	52,72	0,92	-0,12	-0,51	0,61	1347	**0,99**	0,96	-0,07	-0,34	0,73	1625	**0,97**	0,94	-0,08	-0,34	0,74	1518	**1,00**	0,94	3,00	-0,09	0,02
**PA4250**	13333	11,35	298,79	6	35,60	1,12	0,16	0,49	0,63	1380	**0,98**	0,93	-0,11	-0,51	0,61	1374	**0,96**	0,82	-0,29	-1,03	0,30	725	**0,86**	0,95	3,00	-0,07	0,15
**PA4251**	22829	9,69	1594,83	38	67,60	0,92	-0,12	-0,50	0,62	1359	**0,99**	0,96	-0,06	-0,33	0,74	1638	**0,98**	0,95	-0,07	-0,28	0,78	1597	**1,00**	0,94	3,00	-0,08	0,02
**PA4252**	13769	10,17	1209,35	24	65,40	0,90	-0,15	-0,60	0,55	1214	**0,98**	0,94	-0,10	-0,46	0,65	1452	**0,97**	0,95	-0,07	-0,30	0,77	1583	**1,00**	0,93	3,00	-0,10	0,03
**PA4253**	15034	10,76	1353,40	29	70,50	0,94	-0,09	-0,39	0,70	1546	**0,98**	0,95	-0,07	-0,36	0,72	1595	**0,97**	0,96	-0,05	-0,23	0,82	1675	**1,00**	0,95	3,00	-0,07	0,01
**PA4254**	11567	9,93	391,74	13	66,65	0,81	-0,31	-1,17	0,24	653	**0,81**	0,92	-0,12	-0,55	0,58	1307	**0,97**	0,86	-0,22	-0,79	0,43	949	**0,94**	0,86	3,00	-0,21	0,06
**PA4255**	8206	10,27	545,94	13	77,80	0,72	-0,48	-1,77	0,08	347	**0,48**	1,10	0,13	0,39	0,69	1543	**0,97**	1,27	0,34	1,11	0,27	661	**0,83**	1,03	3,00	0,04	0,28
**PA4256**	17697	11,20	633,23	15	48,90	1,10	0,13	0,38	0,70	1552	**0,98**	0,93	-0,11	-0,50	0,62	1384	**0,96**	1,00	0,00	-0,06	0,95	1964	**1,00**	1,01	3,00	0,01	0,08
**PA4257**	28704	10,14	1721,74	38	71,90	0,93	-0,10	-0,43	0,67	1466	**0,99**	0,93	-0,11	-0,50	0,62	1389	**0,96**	0,95	-0,07	-0,31	0,76	1560	**1,00**	0,94	3,00	-0,09	0,01
**PA4258**	14111	10,12	947,31	15	53,60	0,95	-0,07	-0,32	0,75	1645	**0,99**	0,92	-0,13	-0,57	0,57	1280	**0,96**	0,95	-0,07	-0,30	0,76	1571	**1,00**	0,94	3,00	-0,09	0,02
**PA4259**	12048	10,62	720,73	19	68,74	0,97	-0,04	-0,23	0,82	1795	**0,99**	0,94	-0,09	-0,44	0,66	1471	**0,97**	0,94	-0,09	-0,35	0,72	1494	**1,00**	0,95	3,00	-0,07	0,02
**PA4260**	32823	11,23	1621,08	36	64,39	0,94	-0,09	-0,40	0,69	1522	**0,99**	0,96	-0,06	-0,32	0,75	1658	**0,98**	0,93	-0,10	-0,40	0,69	1408	**1,01**	0,94	3,00	-0,08	0,01
**PA4261**	13105	10,05	579,09	14	71,70	0,88	-0,18	-0,71	0,48	1072	**0,97**	1,02	0,02	-0,01	0,99	2146	**1,00**	1,00	0,01	-0,04	0,97	1994	**1,00**	0,97	3,00	-0,05	0,07
**PA4262**	24076	9,90	1528,90	24	67,83	0,93	-0,11	-0,45	0,65	1443	**0,98**	1,00	0,00	-0,10	0,92	2017	**0,99**	0,99	-0,01	-0,10	0,92	1888	**1,00**	0,97	3,00	-0,04	0,04
**PA4263**	24543	9,89	1151,44	19	63,07	1,03	0,04	0,06	0,96	2088	**0,99**	0,92	-0,11	-0,52	0,60	1337	**0,97**	1,00	0,01	-0,04	0,97	1993	**1,00**	0,98	3,00	-0,02	0,05
**PA4264**	12912	9,77	1172,43	28	65,50	0,94	-0,09	-0,38	0,70	1549	**0,98**	0,94	-0,08	-0,41	0,69	1531	**0,97**	0,96	-0,06	-0,27	0,79	1617	**1,00**	0,95	3,00	-0,08	0,01
**PA4265**	46900	5,23	4499,68	82	89,21	0,89	-0,17	-0,69	0,49	1094	**0,97**	0,83	-0,26	-1,07	0,28	731	**0,84**	0,84	-0,24	-0,88	0,38	857	**0,91**	0,86	3,00	-0,23	0,03
**PA4266**	84752	5,06	7206,29	141	84,33	0,91	-0,13	-0,53	0,60	1307	**0,99**	0,88	-0,18	-0,76	0,45	1028	**0,95**	0,92	-0,12	-0,46	0,64	1324	**1,00**	0,91	3,00	-0,14	0,02
**PA4267**	19943	10,24	1112,53	31	62,29	0,94	-0,09	-0,40	0,69	1514	**0,99**	0,96	-0,06	-0,32	0,75	1656	**0,98**	1,00	-0,01	-0,08	0,94	1929	**1,00**	0,96	3,00	-0,05	0,03
**PA4268**	15802	11,01	586,64	16	49,41	0,99	-0,02	-0,15	0,88	1935	**0,99**	0,90	-0,15	-0,64	0,52	1168	**0,96**	0,97	-0,04	-0,20	0,84	1732	**1,00**	0,95	3,00	-0,07	0,04
**PA4269**	167646	6,67	7298,42	151	60,65	0,88	-0,18	-0,72	0,47	1055	**0,97**	0,86	-0,22	-0,91	0,36	856	**0,92**	0,84	-0,25	-0,91	0,36	825	**0,90**	0,86	3,00	-0,22	0,02
**PA4270**	162732	5,61	7843,91	168	65,26	0,90	-0,15	-0,61	0,54	1192	**0,99**	0,87	-0,19	-0,82	0,41	949	**0,94**	0,86	-0,21	-0,77	0,44	974	**0,93**	0,88	3,00	-0,19	0,02
**PA4271**	14489	4,71	902,53	15	76,83	1,17	0,23	0,71	0,48	1066	**0,97**	1,04	0,05	0,11	0,92	2007	**0,99**	1,46	0,54	1,79	0,07	352	**0,43**	1,22	3,00	0,29	0,21
**PA4272**	19609	8,87	1478,69	24	53,20	0,93	-0,10	-0,43	0,67	1471	**0,99**	0,95	-0,08	-0,38	0,70	1561	**0,97**	1,02	0,03	0,05	0,96	1977	**1,00**	0,97	3,00	-0,05	0,05
**PA4273**	27533	9,59	2626,88	50	81,80	0,95	-0,07	-0,32	0,75	1639	**0,99**	0,92	-0,12	-0,56	0,58	1295	**0,96**	0,98	-0,02	-0,14	0,89	1826	**1,00**	0,95	3,00	-0,07	0,03
**PA4274**	16817	9,76	1115,26	21	61,00	0,93	-0,10	-0,43	0,67	1472	**0,99**	0,98	-0,02	-0,18	0,85	1885	**0,98**	0,94	-0,09	-0,35	0,72	1492	**1,00**	0,95	3,00	-0,07	0,03
**PA4275**	22135	5,76	436,83	13	70,77	0,87	-0,21	-0,81	0,42	970	**0,94**	0,84	-0,26	-1,05	0,29	751	**0,85**	0,94	-0,08	-0,34	0,74	1523	**1,00**	0,88	3,00	-0,18	0,06
**PA4276**	14381	9,77	71,89	2	13,10	0,83	-0,27	-1,02	0,31	761	**0,88**	0,97	-0,04	-0,26	0,79	1754	**0,98**	0,85	-0,23	-0,85	0,40	886	**0,92**	0,88	3,00	-0,18	0,08
**PA4278**	27449	5,86	198,46	4	11,10	0,71	-0,50	-1,83	0,07	327	**0,45**	0,79	-0,34	-1,35	0,18	548	**0,70**	0,81	-0,30	-1,08	0,28	684	**0,84**	0,77	3,00	-0,38	0,05
**PA4279**	27829	5,49	498,07	7	24,31	0,81	-0,31	-1,15	0,25	663	**0,81**	0,82	-0,28	-1,14	0,25	670	**0,82**	0,74	-0,44	-1,54	0,12	448	**0,57**	0,79	3,00	-0,34	0,05
**PA4280**	35417	7,04	97,56	2	11,05	0,71	-0,50	-1,84	0,07	323	**0,45**	1,02	0,03	0,03	0,98	2122	**1,00**	0,95	-0,07	-0,30	0,76	1579	**1,00**	0,89	3,00	-0,16	0,17
**PA4283**	80563	6,21	249,26	6	9,05	1,16	0,21	0,66	0,51	1132	**0,98**	1,20	0,26	0,88	0,38	883	**0,93**	1,37	0,45	1,46	0,14	484	**0,61**	1,24	3,00	0,31	0,11
**PA4285**	135049	5,38	127,93	4	4,38	1,05	0,07	0,18	0,86	1885	**0,99**	1,03	0,04	0,04	0,97	2104	**0,99**	1,18	0,24	0,77	0,44	972	**0,93**	1,09	3,00	0,12	0,08
**PA4292**	56144	8,85	285,95	7	11,96	1,10	0,13	0,38	0,70	1553	**0,98**	1,02	0,03	0,01	0,99	2144	**1,00**	1,04	0,05	0,12	0,90	1854	**1,00**	1,05	3,00	0,07	0,04
**PA4296**	31928	6,26	84,66	2	10,20	1,28	0,36	1,19	0,23	638	**0,80**	1,01	0,02	-0,03	0,98	2120	**1,00**	1,19	0,25	0,79	0,43	941	**0,94**	1,16	3,00	0,22	0,14
**PA4307**	70854	4,90	2116,52	43	51,03	0,94	-0,09	-0,40	0,69	1515	**0,98**	0,91	-0,13	-0,59	0,55	1227	**0,97**	0,96	-0,06	-0,27	0,79	1624	**1,00**	0,94	3,00	-0,10	0,02
**PA4308**	56430	6,14	230,11	5	10,82	1,02	0,03	0,03	0,97	2128	**0,99**	0,96	-0,06	-0,31	0,75	1665	**0,98**	1,14	0,19	0,58	0,56	1200	**0,96**	1,04	3,00	0,06	0,09
**PA4309**	71237	5,19	128,57	3	29,93	1,25	0,32	1,04	0,30	740	**0,87**	1,36	0,44	1,55	0,12	475	**0,55**	1,38	0,47	1,52	0,13	454	**0,58**	1,33	3,00	0,41	0,07
**PA4310**	71009	5,00	511,98	12	43,07	1,10	0,13	0,39	0,70	1542	**0,98**	1,16	0,22	0,71	0,48	1077	**0,96**	1,07	0,09	0,26	0,80	1641	**1,00**	1,11	3,00	0,15	0,05

**PA4314**	34539	6,04	860,04	19	58,87	1,04	0,05	0,10	0,92	1999	**1,00**	1,13	0,17	0,55	0,58	1301	**0,96**	1,09	0,12	0,35	0,72	1490	**1,00**	1,08	3,00	0,12	0,05
**PA4315**	17054	9,47	717,82	19	62,90	1,01	0,02	-0,02	0,99	2158	**0,99**	0,99	-0,01	-0,13	0,89	1966	**0,98**	1,01	0,02	0,01	1,00	2053	**1,00**	1,01	3,00	0,01	0,01
**PA4316**	57028	5,48	180,68	9	17,52	0,84	-0,26	-0,99	0,32	785	**0,89**	0,98	-0,03	-0,20	0,84	1862	**0,98**	0,92	-0,12	-0,45	0,65	1346	**1,00**	0,91	3,00	-0,13	0,07
**PA4317**	27556	8,89	113,03	3	12,13	1,18	0,24	0,76	0,45	1012	**0,96**	0,93	-0,11	-0,51	0,61	1359	**0,96**	1,32	0,40	1,31	0,19	552	**0,70**	1,14	3,00	0,19	0,20
**PA4320**	61743	9,09	112,44	4	10,30	1,10	0,14	0,39	0,69	1526	**0,99**	1,00	0,00	-0,11	0,91	1996	**0,99**	1,13	0,18	0,55	0,58	1226	**0,98**	1,08	3,00	0,11	0,07
**PA4321**	48539	5,50	64,12	3	6,60	0,95	-0,08	-0,35	0,73	1602	**0,99**	1,06	0,09	0,24	0,81	1792	**0,98**	0,96	-0,06	-0,27	0,79	1627	**1,00**	0,99	3,00	-0,01	0,06
**PA4322**	38218	5,91	873,38	21	45,20	1,11	0,15	0,44	0,66	1460	**0,99**	1,12	0,17	0,53	0,60	1323	**0,97**	1,09	0,12	0,35	0,73	1498	**1,00**	1,11	3,00	0,15	0,02
**PA4328**	35446	5,64	103,95	4	10,00	1,00	0,00	-0,10	0,92	2000	**1,00**	1,16	0,21	0,69	0,49	1108	**0,96**	0,97	-0,05	-0,23	0,82	1678	**1,00**	1,04	3,00	0,06	0,10
**PA4329**	56341	6,21	2227,45	45	66,12	0,92	-0,12	-0,50	0,62	1367	**0,99**	0,86	-0,21	-0,88	0,38	877	**0,93**	0,89	-0,17	-0,64	0,53	1134	**0,95**	0,89	3,00	-0,17	0,03
**PA4333**	59425	5,31	2340,45	46	55,12	0,79	-0,33	-1,25	0,21	590	**0,77**	0,86	-0,22	-0,91	0,36	851	**0,92**	0,81	-0,30	-1,09	0,28	672	**0,85**	0,82	3,00	-0,29	0,03
**PA4336**	22028	5,66	202,93	6	26,27	0,94	-0,10	-0,42	0,68	1492	**0,98**	1,07	0,09	0,24	0,81	1786	**0,98**	0,96	-0,06	-0,27	0,79	1619	**1,00**	0,99	3,00	-0,02	0,07
**PA4340**	22318	5,82	125,97	3	20,33	0,88	-0,18	-0,72	0,47	1064	**0,97**	0,98	-0,03	-0,21	0,83	1834	**0,98**	1,03	0,05	0,11	0,92	1882	**1,00**	0,96	3,00	-0,05	0,08
**PA4352**	32440	5,92	625,27	12	40,40	0,82	-0,28	-1,08	0,28	714	**0,86**	0,76	-0,39	-1,55	0,12	480	**0,55**	0,97	-0,04	-0,20	0,84	1728	**1,00**	0,85	3,00	-0,23	0,11
**PA4356**	39162	5,11	825,07	17	45,55	0,55	-0,87	-3,15	0,00	140	**0,03**	0,64	-0,65	-2,51	0,01	231	**0,11**	0,74	-0,44	-1,55	0,12	439	**0,57**	0,64	3,00	-0,64	0,10
**PA4360**	19133	9,30	510,17	12	65,95	1,05	0,07	0,18	0,86	1895	**0,99**	1,20	0,26	0,88	0,38	880	**0,93**	1,14	0,19	0,58	0,56	1193	**0,97**	1,13	3,00	0,18	0,07
**PA4363**	33336	6,26	67,29	2	8,00	0,86	-0,22	-0,87	0,39	909	**0,92**	1,83	0,87	3,14	0,00	170	**0,02**	0,74	-0,43	-1,53	0,13	451	**0,58**	1,14	3,00	0,19	0,60
**PA4366**	23159	5,27	544,51	11	33,67	0,72	-0,48	-1,77	0,08	350	**0,48**	0,88	-0,19	-0,79	0,43	987	**0,94**	0,81	-0,30	-1,07	0,29	692	**0,85**	0,80	3,00	-0,32	0,08
**PA4369**	22665	7,85	216,43	6	32,60	0,78	-0,35	-1,32	0,19	555	**0,73**	0,83	-0,27	-1,12	0,26	694	**0,82**	0,75	-0,42	-1,47	0,14	481	**0,60**	0,79	3,00	-0,35	0,04
**PA4370**	52141	4,68	1852,63	43	55,17	1,79	0,84	2,88	0,00	162	**0,05**	1,92	0,94	3,41	0,00	148	**0,01**	1,82	0,86	2,86	0,00	181	**0,05**	1,84	3,00	0,88	0,07
**PA4371**	52486	5,64	210,42	6	15,55	1,32	0,40	1,32	0,19	553	**0,74**	1,48	0,57	2,02	0,04	319	**0,29**	1,41	0,50	1,63	0,10	404	**0,53**	1,40	3,00	0,49	0,08
**PA4372**	41608	5,76	1173,20	28	57,95	1,20	0,26	0,84	0,40	931	**0,93**	1,37	0,46	1,61	0,11	452	**0,52**	1,29	0,37	1,20	0,23	613	**0,78**	1,29	3,00	0,37	0,09
**PA4373**	41075	6,14	250,32	7	19,17	1,22	0,29	0,93	0,35	837	**0,91**	1,22	0,28	0,95	0,34	828	**0,89**	1,23	0,30	0,96	0,34	793	**0,88**	1,22	3,00	0,29	0,01
**PA4374**	43725	5,55	96,35	3	10,73	1,01	0,02	-0,02	0,98	2151	**0,99**	0,91	-0,13	-0,58	0,56	1252	**0,97**	0,84	-0,26	-0,94	0,35	809	**0,89**	0,92	3,00	-0,12	0,09
**PA4376**	48433	6,20	277,03	6	14,80	0,94	-0,08	-0,38	0,71	1558	**0,98**	0,87	-0,19	-0,82	0,41	953	**0,94**	0,86	-0,22	-0,79	0,43	940	**0,94**	0,89	3,00	-0,16	0,04
**PA4378**	28281	9,54	54,63	2	14,10	1,16	0,22	0,68	0,50	1109	**0,98**	1,14	0,19	0,59	0,55	1224	**0,98**	0,87	-0,20	-0,72	0,47	1035	**0,94**	1,06	3,00	0,08	0,16
**PA4381**	26654	5,65	97,72	2	28,20	0,98	-0,02	-0,16	0,87	1922	**0,99**	1,40	0,48	1,71	0,09	415	**0,46**	1,00	0,00	-0,05	0,96	1982	**1,00**	1,13	3,00	0,17	0,23
**PA4385**	63292	5,04	6379,44	111	81,34	0,81	-0,31	-1,18	0,24	644	**0,81**	0,98	-0,03	-0,21	0,83	1841	**0,98**	0,91	-0,14	-0,54	0,59	1233	**0,99**	0,90	3,00	-0,16	0,09
**PA4386**	11269	5,20	445,51	10	61,84	0,72	-0,47	-1,74	0,08	360	**0,49**	0,96	-0,05	-0,29	0,77	1701	**0,98**	0,87	-0,20	-0,74	0,46	1012	**0,93**	0,85	3,00	-0,23	0,12
**PA4387**	17516	10,56	66,91	2	16,80	1,40	0,48	1,61	0,11	412	**0,56**	1,05	0,08	0,18	0,85	1883	**0,98**	1,03	0,05	0,11	0,91	1878	**1,00**	1,16	3,00	0,22	0,20
**PA4388**	27824	5,94	219,16	6	33,17	0,85	-0,24	-0,92	0,36	855	**0,91**	0,88	-0,19	-0,80	0,43	979	**0,94**	0,85	-0,24	-0,85	0,39	881	**0,92**	0,86	3,00	-0,22	0,02
**PA4389**	29192	6,75	570,33	15	44,94	1,00	0,01	-0,06	0,95	2072	**1,00**	1,02	0,02	0,00	1,00	2155	**1,00**	1,06	0,08	0,22	0,83	1691	**1,01**	1,03	3,00	0,04	0,03
**PA4390**	41886	5,40	100,60	4	11,05	0,81	-0,30	-1,15	0,25	666	**0,82**	1,01	0,02	-0,03	0,97	2118	**0,99**	0,94	-0,09	-0,36	0,72	1481	**1,00**	0,92	3,00	-0,12	0,10
**PA4394**	31528	6,62	363,26	7	21,13	1,00	0,00	-0,09	0,93	2010	**1,00**	0,99	-0,02	-0,17	0,86	1904	**0,98**	0,93	-0,10	-0,40	0,69	1412	**1,01**	0,97	3,00	-0,04	0,03
**PA4395**	21007	7,71	446,09	11	48,07	1,08	0,11	0,31	0,76	1661	**0,99**	1,11	0,16	0,49	0,63	1409	**0,96**	1,09	0,12	0,36	0,72	1484	**1,00**	1,09	3,00	0,13	0,02
**PA4400**	35535	5,27	504,29	10	33,50	0,81	-0,30	-1,15	0,25	668	**0,82**	0,80	-0,32	-1,30	0,19	573	**0,74**	0,92	-0,12	-0,47	0,64	1316	**1,00**	0,84	3,00	-0,25	0,07
**PA4402**	44410	5,39	1354,72	20	49,25	1,00	0,01	-0,06	0,95	2085	**1,00**	0,94	-0,09	-0,44	0,66	1473	**0,97**	1,01	0,01	-0,03	0,98	2016	**1,00**	0,98	3,00	-0,03	0,04
**PA4403**	111467	5,43	2864,49	63	51,61	0,96	-0,06	-0,27	0,78	1732	**0,98**	0,94	-0,09	-0,45	0,65	1460	**0,97**	0,99	-0,01	-0,10	0,92	1889	**1,00**	0,96	3,00	-0,05	0,03
**PA4406**	35910	5,21	968,42	21	52,66	1,05	0,07	0,18	0,86	1888	**0,99**	1,09	0,13	0,38	0,70	1557	**0,97**	0,99	-0,02	-0,12	0,90	1857	**1,00**	1,04	3,00	0,06	0,05
**PA4407**	43590	4,93	1602,66	37	57,67	1,23	0,30	0,97	0,33	798	**0,90**	1,30	0,38	1,30	0,19	570	**0,73**	1,30	0,38	1,23	0,22	590	**0,77**	1,28	3,00	0,35	0,04
**PA4408**	47200	5,20	1751,27	33	61,56	1,24	0,31	1,00	0,32	774	**0,89**	1,20	0,26	0,86	0,39	905	**0,93**	1,19	0,25	0,79	0,43	943	**0,94**	1,21	3,00	0,27	0,03
**PA4409**	33671	9,90	101,16	2	9,80	1,35	0,43	1,44	0,15	494	**0,66**	1,44	0,53	1,87	0,06	366	**0,36**	1,35	0,44	1,42	0,16	511	**0,63**	1,38	3,00	0,47	0,05
**PA4410**	36301	4,90	431,86	14	38,16	1,35	0,44	1,46	0,15	482	**0,65**	1,31	0,39	1,37	0,17	540	**0,69**	1,34	0,42	1,37	0,17	533	**0,66**	1,34	3,00	0,42	0,02
**PA4411**	53974	5,80	622,60	17	25,41	1,35	0,44	1,45	0,15	487	**0,66**	1,15	0,20	0,65	0,51	1151	**0,97**	1,33	0,41	1,33	0,18	544	**0,69**	1,28	3,00	0,35	0,11
**PA4412**	39590	9,59	561,65	15	35,67	1,35	0,43	1,44	0,15	492	**0,66**	1,27	0,34	1,18	0,24	640	**0,80**	1,32	0,41	1,32	0,19	550	**0,71**	1,31	3,00	0,39	0,04
**PA4414**	50966	5,62	934,88	21	40,07	1,20	0,27	0,85	0,39	921	**0,93**	1,23	0,30	1,02	0,31	775	**0,85**	1,24	0,32	1,01	0,31	746	**0,86**	1,23	3,00	0,29	0,02
**PA4416**	49348	6,09	733,35	16	33,85	1,21	0,27	0,87	0,38	898	**0,93**	1,18	0,24	0,82	0,41	955	**0,94**	1,25	0,32	1,03	0,30	732	**0,86**	1,21	3,00	0,28	0,03
**PA4417**	53335	5,24	750,90	20	33,62	1,23	0,30	0,96	0,34	811	**0,90**	1,12	0,16	0,50	0,62	1379	**0,96**	1,20	0,26	0,82	0,41	921	**0,92**	1,18	3,00	0,24	0,06
**PA4418**	66898	9,58	292,00	8	14,35	1,05	0,07	0,18	0,86	1893	**0,99**	1,07	0,10	0,28	0,78	1727	**0,98**	1,12	0,17	0,52	0,61	1258	**0,99**	1,08	3,00	0,11	0,04

**PA4420**	36971	7,01	716,26	12	40,58	1,03	0,04	0,06	0,96	2087	**1,00**	0,99	-0,01	-0,14	0,89	1965	**0,98**	1,08	0,11	0,30	0,76	1572	**1,00**	1,03	3,00	0,05	0,04
**PA4422**	31713	5,87	430,70	14	45,55	1,05	0,07	0,16	0,87	1914	**0,99**	1,05	0,07	0,18	0,86	1897	**0,98**	1,14	0,19	0,58	0,56	1205	**0,96**	1,08	3,00	0,11	0,05
**PA4423**	68321	6,69	1711,26	34	49,08	1,12	0,16	0,48	0,63	1396	**0,99**	1,10	0,13	0,39	0,69	1542	**0,97**	1,11	0,14	0,43	0,66	1367	**1,00**	1,11	3,00	0,15	0,01
**PA4425**	22258	5,00	674,03	11	52,80	1,02	0,03	0,03	0,97	2129	**0,99**	1,20	0,27	0,90	0,37	864	**0,92**	1,35	0,43	1,40	0,16	519	**0,64**	1,19	3,00	0,25	0,16
**PA4426**	22814	9,21	475,42	11	42,52	0,99	-0,01	-0,12	0,90	1976	**0,99**	1,24	0,32	1,08	0,28	728	**0,83**	1,08	0,11	0,31	0,76	1568	**1,00**	1,10	3,00	0,14	0,13
**PA4428**	25029	5,87	179,90	4	18,00	0,96	-0,06	-0,29	0,77	1695	**0,99**	1,00	0,00	-0,10	0,92	2022	**0,99**	0,91	-0,13	-0,50	0,62	1277	**1,00**	0,96	3,00	-0,06	0,04
**PA4429**	31897	7,00	1117,02	24	57,33	0,95	-0,08	-0,36	0,72	1591	**0,99**	0,98	-0,03	-0,21	0,83	1835	**0,98**	1,00	0,00	-0,07	0,95	1956	**1,00**	0,97	3,00	-0,04	0,03
**PA4430**	48728	8,59	234,93	7	15,53	0,86	-0,21	-0,83	0,41	941	**0,94**	0,96	-0,06	-0,34	0,74	1631	**0,98**	0,93	-0,11	-0,43	0,67	1375	**1,00**	0,92	3,00	-0,13	0,05
**PA4431**	22728	6,07	436,68	11	35,69	0,82	-0,28	-1,06	0,29	727	**0,86**	0,88	-0,19	-0,81	0,42	963	**0,94**	0,87	-0,21	-0,76	0,45	986	**0,94**	0,86	3,00	-0,23	0,03
**PA4432**	16047	11,22	425,88	10	49,20	0,92	-0,13	-0,52	0,60	1323	**0,99**	0,89	-0,16	-0,71	0,48	1081	**0,96**	0,89	-0,16	-0,61	0,54	1166	**0,96**	0,90	3,00	-0,15	0,01
**PA4433**	18324	9,84	679,92	20	70,40	0,93	-0,11	-0,46	0,64	1425	**0,98**	0,94	-0,09	-0,42	0,67	1509	**0,97**	1,01	0,01	-0,03	0,98	2022	**1,00**	0,96	3,00	-0,06	0,04
**PA4434**	39767	5,62	413,58	10	29,40	0,83	-0,27	-1,01	0,31	765	**0,88**	1,01	0,01	-0,05	0,96	2092	**0,99**	0,98	-0,03	-0,16	0,87	1800	**1,00**	0,94	3,00	-0,09	0,10
**PA4438**	45321	5,84	879,03	25	50,32	0,94	-0,09	-0,41	0,68	1501	**0,99**	0,95	-0,07	-0,37	0,71	1582	**0,97**	0,97	-0,05	-0,22	0,82	1684	**1,01**	0,95	3,00	-0,07	0,01
**PA4439**	51761	5,70	857,59	21	42,74	0,90	-0,15	-0,61	0,54	1196	**0,98**	0,92	-0,13	-0,57	0,57	1283	**0,96**	0,94	-0,09	-0,38	0,71	1451	**1,00**	0,92	3,00	-0,12	0,02
**PA4440**	23062	5,79	503,43	9	43,69	0,97	-0,05	-0,26	0,80	1750	**0,99**	0,94	-0,09	-0,45	0,65	1461	**0,97**	0,99	-0,02	-0,13	0,90	1840	**1,00**	0,96	3,00	-0,06	0,02
**PA4441**	17529	5,91	597,88	13	63,75	0,97	-0,04	-0,21	0,83	1822	**0,99**	0,92	-0,12	-0,53	0,59	1320	**0,97**	1,12	0,17	0,51	0,61	1270	**0,99**	1,01	3,00	0,01	0,10
**PA4442**	73733	5,55	754,20	18	25,48	1,18	0,24	0,77	0,44	1008	**0,95**	1,35	0,43	1,50	0,13	498	**0,58**	1,31	0,39	1,25	0,21	579	**0,75**	1,28	3,00	0,35	0,09
**PA4443**	38816	6,58	391,41	11	22,36	1,18	0,23	0,74	0,46	1031	**0,96**	1,49	0,57	2,03	0,04	315	**0,29**	1,16	0,21	0,67	0,50	1092	**0,95**	1,27	3,00	0,35	0,18
**PA4444**	43009	9,83	50,92	2	10,10	1,44	0,52	1,76	0,08	355	**0,48**	1,52	0,61	2,16	0,03	295	**0,23**	1,41	0,50	1,63	0,10	401	**0,52**	1,46	3,00	0,54	0,06
**PA4445**	28247	5,33	409,09	8	30,15	0,91	-0,13	-0,53	0,60	1308	**0,99**	0,96	-0,06	-0,33	0,74	1647	**0,98**	0,94	-0,10	-0,38	0,70	1440	**1,00**	0,94	3,00	-0,10	0,02
**PA4446**	43038	6,25	414,33	10	20,28	1,05	0,07	0,17	0,87	1899	**0,99**	1,10	0,14	0,43	0,67	1496	**0,97**	1,04	0,06	0,15	0,88	1805	**1,00**	1,07	3,00	0,09	0,03
**PA4447**	40498	5,69	384,20	10	31,04	0,81	-0,31	-1,17	0,24	649	**0,81**	0,88	-0,18	-0,78	0,44	1001	**0,94**	0,86	-0,23	-0,82	0,41	916	**0,92**	0,85	3,00	-0,24	0,04
**PA4448**	48677	5,07	1234,99	28	49,46	0,88	-0,18	-0,71	0,48	1068	**0,97**	0,87	-0,20	-0,86	0,39	908	**0,93**	0,79	-0,34	-1,21	0,23	608	**0,77**	0,85	3,00	-0,24	0,05
**PA4449**	24750	8,97	909,57	18	78,20	1,10	0,13	0,39	0,70	1534	**0,99**	0,94	-0,09	-0,43	0,67	1491	**0,97**	0,94	-0,08	-0,34	0,73	1510	**1,00**	0,99	3,00	-0,01	0,09
**PA4450**	47395	5,52	1116,25	26	42,40	0,89	-0,17	-0,67	0,50	1123	**0,98**	0,94	-0,09	-0,44	0,66	1480	**0,96**	0,96	-0,07	-0,28	0,78	1604	**1,00**	0,93	3,00	-0,11	0,03
**PA4452**	11393	5,48	189,72	5	49,00	1,06	0,08	0,21	0,84	1835	**0,99**	1,06	0,08	0,21	0,84	1847	**0,98**	0,95	-0,07	-0,30	0,76	1578	**1,00**	1,02	3,00	0,03	0,06
**PA4453**	26483	9,13	951,93	22	46,62	1,10	0,14	0,42	0,67	1480	**0,99**	1,19	0,25	0,83	0,41	942	**0,93**	1,27	0,35	1,13	0,26	650	**0,82**	1,19	3,00	0,25	0,08
**PA4454**	17988	4,90	395,71	7	47,24	1,01	0,02	-0,02	0,98	2152	**0,99**	1,09	0,13	0,37	0,71	1579	**0,97**	1,18	0,23	0,73	0,46	1023	**0,94**	1,09	3,00	0,13	0,08
**PA4455**	29130	6,74	331,53	7	25,93	0,97	-0,05	-0,26	0,80	1749	**0,99**	1,11	0,16	0,49	0,63	1410	**0,96**	1,24	0,31	0,99	0,32	762	**0,87**	1,11	3,00	0,15	0,14
**PA4456**	31234	6,45	217,36	8	38,58	1,11	0,16	0,47	0,64	1412	**0,98**	1,05	0,06	0,14	0,89	1953	**0,98**	1,04	0,05	0,11	0,91	1872	**1,00**	1,07	3,00	0,09	0,04
**PA4457**	36412	5,48	529,32	12	30,77	0,96	-0,06	-0,28	0,78	1723	**0,98**	0,96	-0,06	-0,32	0,75	1657	**0,98**	0,95	-0,07	-0,31	0,76	1565	**1,00**	0,96	3,00	-0,06	0,01
**PA4458**	20089	5,52	334,86	8	44,40	1,03	0,04	0,07	0,94	2045	**1,00**	1,08	0,11	0,33	0,74	1648	**0,98**	1,06	0,09	0,25	0,81	1657	**1,00**	1,06	3,00	0,08	0,03
**PA4459**	23069	6,21	162,84	5	29,36	1,15	0,20	0,64	0,52	1148	**0,99**	0,89	-0,17	-0,73	0,47	1054	**0,96**	0,89	-0,16	-0,61	0,54	1159	**0,96**	0,98	3,00	-0,03	0,15
**PA4460**	21132	9,07	512,90	13	50,75	1,06	0,08	0,20	0,84	1843	**0,99**	1,00	-0,01	-0,12	0,91	1992	**0,98**	1,02	0,03	0,06	0,96	1969	**1,00**	1,03	3,00	0,04	0,03
**PA4461**	28255	6,20	635,11	16	53,65	1,15	0,20	0,63	0,53	1157	**0,99**	0,94	-0,09	-0,44	0,66	1477	**0,97**	1,01	0,01	-0,01	0,99	2050	**1,00**	1,03	3,00	0,05	0,11
**PA4462**	58751	4,70	653,12	16	27,63	1,02	0,02	0,01	0,99	2166	**1,00**	0,97	-0,04	-0,26	0,80	1757	**0,98**	1,02	0,02	0,02	0,98	2025	**1,00**	1,00	3,00	0,00	0,03
**PA4464**	17388	4,89	434,50	9	52,68	1,16	0,21	0,67	0,50	1125	**0,97**	1,22	0,29	0,97	0,33	813	**0,89**	1,36	0,45	1,46	0,14	487	**0,61**	1,25	3,00	0,32	0,11
**PA4465**	33824	5,55	295,11	9	22,53	1,01	0,02	-0,02	0,98	2146	**1,00**	1,07	0,10	0,28	0,78	1711	**0,98**	1,05	0,07	0,20	0,85	1741	**1,00**	1,05	3,00	0,06	0,03
**PA4466**	10508	5,56	96,27	3	28,50	1,13	0,18	0,53	0,59	1302	**0,99**	1,06	0,08	0,20	0,84	1865	**0,98**	1,15	0,20	0,64	0,52	1132	**0,96**	1,11	3,00	0,15	0,05
**PA4470**	50775	5,65	235,42	6	21,87	3,00	1,58	5,48	0,00	68	**0,00**	2,39	1,25	4,58	0,00	98	**0,00**	2,01	1,01	3,36	0,00	142	**0,01**	2,47	3,00	1,30	0,50
**PA4472**	49262	5,49	412,98	9	23,04	1,08	0,11	0,30	0,76	1676	**0,99**	0,97	-0,05	-0,27	0,78	1732	**0,98**	0,97	-0,05	-0,22	0,82	1686	**1,01**	1,00	3,00	0,01	0,06
**PA4473**	25088	8,99	198,25	6	23,50	1,18	0,24	0,77	0,44	1005	**0,95**	1,15	0,20	0,64	0,52	1179	**0,96**	0,99	-0,02	-0,11	0,91	1870	**1,00**	1,11	3,00	0,15	0,10
**PA4474**	52725	5,22	555,27	11	21,16	0,96	-0,07	-0,31	0,75	1658	**0,99**	1,07	0,09	0,25	0,80	1770	**0,98**	0,93	-0,11	-0,43	0,67	1378	**1,00**	0,98	3,00	-0,02	0,07
**PA4475**	31419	6,34	100,63	4	16,13	0,87	-0,19	-0,76	0,44	1011	**0,96**	1,14	0,20	0,63	0,53	1191	**0,96**	0,91	-0,13	-0,50	0,62	1278	**1,00**	0,98	3,00	-0,03	0,15
**PA4477**	57501	5,26	294,39	8	18,75	1,07	0,10	0,26	0,80	1754	**0,99**	1,00	-0,01	-0,12	0,90	1985	**0,98**	1,14	0,19	0,60	0,55	1173	**0,96**	1,07	3,00	0,10	0,07
**PA4478**	21721	5,02	112,22	3	13,27	1,16	0,21	0,66	0,51	1133	**0,98**	0,85	-0,24	-1,00	0,32	797	**0,86**	1,34	0,43	1,39	0,16	526	**0,65**	1,12	3,00	0,16	0,25
**PA4480**	36763	5,64	223,78	7	15,89	1,14	0,18	0,57	0,57	1261	**0,98**	1,05	0,06	0,14	0,89	1955	**0,98**	0,83	-0,27	-0,96	0,34	791	**0,87**	1,00	3,00	0,01	0,16
**PA4481**	38866	5,33	1664,45	37	59,74	1,04	0,05	0,10	0,92	1992	**1,00**	1,01	0,01	-0,05	0,96	2085	**0,99**	0,95	-0,08	-0,33	0,74	1527	**1,00**	1,00	3,00	0,00	0,05

**PA4482**	10956	4,56	292,56	6	88,50	0,95	-0,08	-0,35	0,73	1606	**0,99**	1,21	0,28	0,95	0,34	832	**0,89**	1,42	0,51	1,66	0,10	391	**0,51**	1,20	3,00	0,26	0,24
**PA4483**	54752	5,52	1782,61	40	56,80	1,00	0,00	-0,07	0,95	2056	**1,00**	1,10	0,14	0,43	0,66	1485	**0,97**	1,04	0,05	0,13	0,90	1850	**1,00**	1,05	3,00	0,07	0,05
**PA4484**	58223	5,28	1996,72	42	53,67	1,03	0,04	0,05	0,96	2095	**1,00**	1,00	0,01	-0,07	0,94	2056	**0,99**	1,04	0,06	0,13	0,90	1844	**1,00**	1,02	3,00	0,03	0,02
**PA4489**	175684	5,48	2439,71	53	34,26	1,12	0,16	0,48	0,63	1386	**0,98**	1,09	0,13	0,37	0,71	1588	**0,97**	1,07	0,09	0,26	0,80	1638	**1,00**	1,09	3,00	0,13	0,03
**PA4491**	66861	5,11	913,51	21	32,89	1,23	0,29	0,95	0,34	817	**0,91**	1,08	0,11	0,30	0,76	1682	**0,98**	1,03	0,04	0,09	0,93	1913	**1,00**	1,11	3,00	0,15	0,10
**PA4493**	21704	5,38	200,14	5	24,20	1,03	0,04	0,05	0,96	2102	**1,00**	0,99	-0,01	-0,13	0,90	1976	**0,98**	1,08	0,11	0,33	0,75	1539	**1,00**	1,03	3,00	0,05	0,04
**PA4495**	27071	5,79	577,39	10	49,19	0,98	-0,03	-0,20	0,85	1861	**0,99**	0,88	-0,19	-0,79	0,43	989	**0,94**	0,81	-0,30	-1,08	0,28	680	**0,84**	0,89	3,00	-0,17	0,08
**PA4496**	66852	7,12	245,23	5	19,92	2,61	1,38	4,77	0,00	77	**0,00**	3,64	1,86	6,85	0,00	45	**0,00**	3,61	1,85	6,22	0,00	55	**0,00**	3,29	3,00	1,72	0,59
**PA4498**	45527	5,79	1263,61	30	61,00	3,20	1,68	5,81	0,00	55	**0,00**	3,12	1,64	6,02	0,00	64	**0,00**	3,43	1,78	5,97	0,00	60	**0,00**	3,25	3,00	1,70	0,16
**PA4500**	65729	8,09	1969,71	44	45,79	5,33	2,41	8,39	0,00	1	**0,00**	5,01	2,32	8,57	0,00	2	**0,00**	4,76	2,25	7,57	0,00	42	**0,00**	5,03	3,00	2,33	0,28
**PA4501**	55705	5,62	620,28	12	22,03	4,34	2,12	7,35	0,00	31	**0,00**	4,39	2,14	7,86	0,00	32	**0,00**	8,52	3,09	10,42	0,00	23	**0,00**	5,75	3,00	2,52	2,40
**PA4502**	62552	6,16	1807,17	38	57,68	6,34	2,66	9,27	0,00	12	**0,00**	4,45	2,15	7,93	0,00	31	**0,00**	7,16	2,84	9,57	0,00	15	**0,00**	5,98	3,00	2,58	1,39
**PA4503**	37952	8,04	563,15	15	27,40	4,37	2,13	7,38	0,00	30	**0,00**	4,23	2,08	7,66	0,00	37	**0,00**	6,19	2,63	8,86	0,00	10	**0,00**	4,93	3,00	2,30	1,10
**PA4504**	33205	7,72	406,61	11	26,39	3,32	1,73	6,00	0,00	50	**0,00**	3,40	1,77	6,49	0,00	51	**0,00**	8,73	3,13	10,53	0,00	24	**0,00**	5,15	3,00	2,37	3,10
**PA4505**	36979	5,75	685,36	16	43,85	4,64	2,21	7,69	0,00	27	**0,00**	6,41	2,68	9,89	0,00	12	**0,00**	4,22	2,08	6,99	0,00	48	**0,00**	5,09	3,00	2,35	1,16
**PA4506**	38564	9,30	1131,29	31	65,21	5,22	2,39	8,29	0,00	21	**0,00**	4,76	2,25	8,29	0,00	23	**0,00**	3,38	1,76	5,89	0,00	61	**0,00**	4,45	3,00	2,16	0,96
**PA4512**	37153	9,65	91,82	2	10,20	0,92	-0,12	-0,50	0,62	1360	**0,99**	0,91	-0,13	-0,59	0,55	1228	**0,97**	1,12	0,16	0,49	0,63	1293	**1,00**	0,98	3,00	-0,02	0,12
**PA4513**	97130	6,75	387,97	11	11,71	1,99	0,99	3,40	0,00	124	**0,01**	1,72	0,78	2,82	0,00	196	**0,05**	1,67	0,74	2,44	0,01	233	**0,13**	1,79	3,00	0,84	0,17
**PA4514**	87710	5,72	1578,49	36	36,73	3,92	1,97	6,84	0,00	37	**0,00**	4,38	2,13	7,85	0,00	33	**0,00**	3,62	1,86	6,23	0,00	54	**0,00**	3,98	3,00	1,99	0,38
**PA4515**	26655	6,13	232,22	9	34,00	2,10	1,07	3,69	0,00	109	**0,00**	1,71	0,78	2,80	0,01	202	**0,06**	1,89	0,92	3,06	0,00	165	**0,03**	1,90	3,00	0,93	0,20
**PA4519**	47705	4,95	864,47	21	47,15	1,26	0,33	1,09	0,28	710	**0,85**	1,10	0,14	0,43	0,66	1486	**0,97**	1,15	0,20	0,61	0,54	1168	**0,96**	1,17	3,00	0,23	0,08
**PA4524**	31968	5,26	740,45	16	50,44	1,09	0,12	0,35	0,73	1604	**0,99**	0,96	-0,05	-0,29	0,77	1700	**0,98**	0,94	-0,09	-0,37	0,71	1462	**1,00**	1,00	3,00	0,00	0,08
**PA4525**	17179	6,23	600,82	12	46,30	0,64	-0,63	-2,31	0,02	233	**0,20**	0,66	-0,60	-2,32	0,02	267	**0,16**	0,65	-0,63	-2,18	0,03	273	**0,22**	0,65	3,00	-0,62	0,01
**PA4526**	67563	5,63	299,89	10	14,03	0,76	-0,40	-1,49	0,14	465	**0,64**	0,75	-0,41	-1,62	0,10	446	**0,51**	0,73	-0,46	-1,63	0,10	405	**0,53**	0,75	3,00	-0,42	0,02
**PA4529**	23550	6,31	248,66	6	27,35	0,94	-0,09	-0,38	0,70	1557	**0,98**	0,90	-0,15	-0,66	0,51	1136	**0,96**	0,77	-0,37	-1,31	0,19	553	**0,70**	0,87	3,00	-0,20	0,09
**PA4533**	18755	9,01	134,13	4	25,30	1,21	0,27	0,87	0,38	901	**0,93**	0,90	-0,15	-0,67	0,50	1130	**0,97**	0,99	-0,02	-0,12	0,90	1852	**1,00**	1,03	3,00	0,04	0,16
**PA4542**	102532	5,34	2766,07	67	59,69	0,83	-0,27	-1,03	0,30	755	**0,88**	0,95	-0,07	-0,35	0,73	1616	**0,97**	0,95	-0,08	-0,32	0,75	1545	**1,00**	0,91	3,00	-0,14	0,07
**PA4544**	37044	6,79	363,90	10	31,94	1,17	0,22	0,70	0,48	1082	**0,97**	1,12	0,16	0,50	0,62	1381	**0,96**	1,12	0,17	0,51	0,61	1267	**0,99**	1,14	3,00	0,18	0,03
**PA4545**	41231	4,98	858,60	16	37,33	1,00	0,01	-0,06	0,95	2073	**1,00**	1,06	0,08	0,20	0,84	1858	**0,98**	1,04	0,06	0,15	0,88	1817	**1,00**	1,03	3,00	0,05	0,03
**PA4547**	52772	5,82	453,60	10	20,80	0,90	-0,15	-0,60	0,55	1207	**0,99**	0,89	-0,17	-0,74	0,46	1046	**0,95**	0,88	-0,18	-0,68	0,50	1082	**0,94**	0,89	3,00	-0,17	0,01
**PA4548**	40778	6,11	316,96	8	26,35	0,73	-0,46	-1,69	0,09	382	**0,51**	0,72	-0,48	-1,89	0,06	359	**0,35**	0,80	-0,33	-1,18	0,24	626	**0,79**	0,75	3,00	-0,42	0,04
**PA4553**	21635	5,87	101,17	3	12,63	0,40	-1,32	-4,72	0,00	79	**0,00**	0,51	-0,97	-3,71	0,00	132	**0,00**	0,47	-1,08	-3,72	0,00	123	**0,00**	0,46	3,00	-1,12	0,06
**PA4554**	133070	6,00	512,51	10	8,25	0,36	-1,48	-5,29	0,00	71	**0,00**	0,41	-1,29	-4,89	0,00	86	**0,00**	0,39	-1,37	-4,70	0,00	91	**0,00**	0,38	3,00	-1,38	0,03
**PA4556**	16371	9,69	125,03	3	13,47	0,55	-0,87	-3,12	0,00	141	**0,03**	0,61	-0,70	-2,72	0,01	210	**0,07**	0,39	-1,35	-4,63	0,00	93	**0,00**	0,52	3,00	-0,95	0,11
**PA4557**	36844	5,31	374,50	10	25,15	1,07	0,10	0,29	0,78	1708	**0,99**	1,18	0,23	0,78	0,44	1005	**0,94**	1,10	0,14	0,41	0,68	1398	**1,00**	1,12	3,00	0,16	0,05
**PA4558**	17271	4,51	559,32	11	60,90	1,25	0,33	1,06	0,29	721	**0,87**	1,07	0,09	0,25	0,80	1771	**0,98**	1,08	0,11	0,31	0,76	1569	**1,00**	1,13	3,00	0,18	0,11
**PA4560**	112573	5,76	2031,54	46	42,18	1,01	0,02	-0,01	0,99	2162	**1,00**	1,07	0,09	0,25	0,80	1763	**0,98**	1,07	0,09	0,26	0,80	1637	**1,00**	1,05	3,00	0,07	0,03
**PA4561**	35558	9,14	343,77	9	29,79	1,13	0,18	0,56	0,58	1272	**0,98**	0,93	-0,10	-0,47	0,64	1435	**0,96**	1,03	0,04	0,08	0,94	1933	**1,00**	1,03	3,00	0,05	0,10
**PA4563**	11897	11,14	225,02	5	41,80	0,94	-0,09	-0,39	0,70	1538	**0,99**	0,92	-0,11	-0,52	0,60	1338	**0,97**	0,87	-0,21	-0,75	0,45	996	**0,93**	0,91	3,00	-0,14	0,04
**PA4564**	18391	8,95	143,21	4	25,80	1,12	0,17	0,50	0,61	1352	**0,99**	1,02	0,03	0,00	1,00	2157	**1,00**	1,11	0,15	0,45	0,65	1340	**1,00**	1,08	3,00	0,12	0,06
**PA4565**	41498	6,73	454,01	9	25,03	1,12	0,16	0,49	0,62	1372	**0,99**	1,05	0,07	0,16	0,87	1920	**0,98**	1,08	0,11	0,33	0,74	1535	**1,00**	1,08	3,00	0,12	0,04
**PA4566**	47096	4,80	733,02	14	44,43	0,99	-0,02	-0,15	0,88	1932	**0,99**	0,94	-0,09	-0,43	0,67	1497	**0,97**	0,87	-0,20	-0,73	0,47	1030	**0,93**	0,93	3,00	-0,10	0,06
**PA4567**	10714	10,67	324,49	9	60,00	0,84	-0,25	-0,97	0,33	803	**0,90**	0,91	-0,14	-0,60	0,55	1215	**0,97**	0,97	-0,05	-0,22	0,83	1695	**1,01**	0,91	3,00	-0,14	0,06
**PA4568**	13376	9,85	910,65	20	71,80	0,92	-0,13	-0,53	0,60	1312	**0,99**	0,97	-0,05	-0,28	0,78	1724	**0,98**	0,90	-0,16	-0,59	0,55	1185	**0,96**	0,93	3,00	-0,11	0,04
**PA4569**	36455	4,86	663,77	14	44,61	1,07	0,10	0,26	0,80	1755	**0,99**	1,00	0,00	-0,08	0,93	2030	**0,99**	0,98	-0,03	-0,17	0,87	1784	**1,00**	1,02	3,00	0,02	0,05
**PA4572**	22503	4,78	470,67	10	40,12	0,94	-0,09	-0,39	0,70	1547	**0,98**	0,71	-0,50	-1,97	0,05	333	**0,32**	0,72	-0,48	-1,68	0,09	385	**0,50**	0,79	3,00	-0,34	0,13
**PA4576**	95441	4,95	1884,34	46	43,49	1,02	0,03	0,02	0,99	2156	**0,99**	1,00	0,00	-0,08	0,93	2039	**0,99**	0,95	-0,07	-0,30	0,76	1570	**1,00**	0,99	3,00	-0,01	0,04
**PA4579**	72939	5,37	61,40	2	7,80	0,73	-0,45	-1,66	0,10	395	**0,53**	1,08	0,11	0,32	0,75	1654	**0,98**	0,84	-0,25	-0,90	0,37	839	**0,91**	0,89	3,00	-0,18	0,18

**PA4582**	45368	6,12	129,53	4	11,70	1,26	0,34	1,11	0,27	693	**0,84**	1,00	0,00	-0,11	0,91	1998	**0,99**	0,86	-0,22	-0,81	0,42	932	**0,93**	1,04	3,00	0,06	0,21
**PA4583**	48295	8,40	121,02	3	8,60	0,82	-0,28	-1,06	0,29	728	**0,87**	0,94	-0,08	-0,41	0,68	1524	**0,97**	0,82	-0,29	-1,04	0,30	717	**0,85**	0,86	3,00	-0,21	0,07
**PA4595**	66354	5,48	2238,57	48	58,92	0,88	-0,18	-0,73	0,47	1048	**0,97**	0,82	-0,28	-1,15	0,25	662	**0,81**	0,84	-0,24	-0,88	0,38	858	**0,91**	0,85	3,00	-0,24	0,03
**PA4601**	163596	5,74	65,60	2	4,20	1,22	0,29	0,94	0,35	825	**0,91**	1,25	0,32	1,09	0,28	720	**0,83**	1,36	0,44	1,44	0,15	496	**0,62**	1,28	3,00	0,35	0,07
**PA4602**	48529	5,70	1232,12	28	46,20	0,94	-0,09	-0,40	0,69	1516	**0,98**	0,96	-0,06	-0,31	0,75	1666	**0,98**	0,87	-0,20	-0,75	0,46	1004	**0,93**	0,92	3,00	-0,12	0,05
**PA4604**	38616	4,72	225,46	6	20,85	0,76	-0,40	-1,49	0,14	469	**0,64**	0,76	-0,40	-1,58	0,11	461	**0,53**	0,60	-0,73	-2,53	0,01	223	**0,11**	0,71	3,00	-0,50	0,09
**PA4606**	76152	7,15	230,53	7	8,06	0,78	-0,37	-1,37	0,17	526	**0,71**	0,87	-0,21	-0,87	0,38	895	**0,93**	0,99	-0,01	-0,09	0,93	1910	**1,00**	0,88	3,00	-0,19	0,11
**PA4609**	51204	6,43	520,59	12	30,70	0,86	-0,22	-0,84	0,40	933	**0,93**	0,88	-0,18	-0,76	0,45	1022	**0,95**	1,18	0,24	0,74	0,46	1009	**0,94**	0,97	3,00	-0,04	0,18
**PA4613**	60987	7,85	426,71	13	24,44	1,54	0,62	2,10	0,04	265	**0,29**	1,96	0,97	3,52	0,00	142	**0,01**	1,52	0,60	1,99	0,05	304	**0,32**	1,67	3,00	0,74	0,25
**PA4614**	15864	4,90	456,31	10	46,44	1,06	0,08	0,20	0,84	1851	**0,99**	1,05	0,08	0,19	0,85	1878	**0,98**	0,96	-0,07	-0,28	0,78	1609	**1,00**	1,02	3,00	0,03	0,06
**PA4615**	30460	5,40	451,55	13	35,68	1,26	0,34	1,10	0,27	695	**0,85**	1,15	0,20	0,66	0,51	1144	**0,96**	1,29	0,37	1,19	0,23	618	**0,78**	1,23	3,00	0,30	0,07
**PA4616**	40827	8,79	362,83	8	22,15	0,25	-2,02	-7,16	0,00	34	**0,00**	0,22	-2,17	-8,18	0,00	24	**0,00**	0,14	-2,80	-9,54	0,00	32	**0,00**	0,20	3,00	-2,29	0,05
**PA4617**	42364	8,66	167,87	5	16,64	1,16	0,22	0,68	0,49	1100	**0,98**	1,01	0,01	-0,07	0,95	2066	**0,99**	1,05	0,07	0,17	0,87	1781	**1,00**	1,07	3,00	0,10	0,08
**PA4619**	47408	5,85	371,11	8	20,00	0,68	-0,56	-2,06	0,04	270	**0,32**	0,67	-0,59	-2,28	0,02	270	**0,18**	0,82	-0,30	-1,06	0,29	704	**0,85**	0,72	3,00	-0,48	0,08
**PA4620**	21426	5,34	84,47	3	9,70	0,49	-1,02	-3,66	0,00	111	**0,00**	0,51	-0,98	-3,75	0,00	130	**0,00**	0,52	-0,95	-3,27	0,00	149	**0,01**	0,51	3,00	-0,98	0,01
**PA4621**	109939	6,81	300,14	9	10,83	0,71	-0,50	-1,85	0,06	316	**0,44**	0,77	-0,39	-1,53	0,13	488	**0,56**	0,61	-0,71	-2,46	0,01	230	**0,12**	0,69	3,00	-0,53	0,08
**PA4626**	35215	6,20	249,24	7	29,84	0,93	-0,10	-0,44	0,66	1455	**0,99**	1,07	0,10	0,27	0,79	1746	**0,98**	0,97	-0,04	-0,20	0,85	1738	**1,00**	0,99	3,00	-0,01	0,07
**PA4627**	37206	7,82	322,28	9	27,17	0,95	-0,07	-0,34	0,73	1609	**0,99**	0,80	-0,33	-1,31	0,19	565	**0,73**	0,83	-0,26	-0,95	0,34	798	**0,88**	0,86	3,00	-0,22	0,08
**PA4628**	54688	9,16	266,47	6	8,93	1,06	0,08	0,21	0,84	1836	**0,99**	0,98	-0,03	-0,22	0,83	1828	**0,98**	1,28	0,35	1,13	0,26	645	**0,82**	1,10	3,00	0,14	0,15
**PA4631**	38067	7,29	222,79	8	23,85	1,08	0,11	0,29	0,77	1687	**0,99**	1,03	0,05	0,08	0,93	2031	**0,99**	0,99	-0,01	-0,11	0,91	1881	**1,00**	1,03	3,00	0,05	0,04
**PA4632**	31106	6,97	886,86	18	46,45	0,91	-0,13	-0,54	0,59	1299	**0,99**	0,99	-0,01	-0,15	0,88	1939	**0,98**	0,97	-0,04	-0,20	0,84	1720	**1,00**	0,96	3,00	-0,06	0,04
**PA4633**	79694	4,78	688,40	13	25,64	1,15	0,20	0,61	0,54	1193	**0,98**	1,21	0,27	0,92	0,36	846	**0,91**	1,38	0,47	1,53	0,13	449	**0,58**	1,25	3,00	0,32	0,12
**PA4636**	46484	6,85	756,10	21	50,90	1,08	0,12	0,32	0,75	1638	**0,99**	1,09	0,13	0,39	0,70	1552	**0,97**	1,07	0,10	0,29	0,77	1588	**1,00**	1,08	3,00	0,12	0,01
**PA4639**	21922	9,47	645,49	17	63,89	1,15	0,20	0,61	0,54	1188	**0,99**	1,19	0,25	0,83	0,41	935	**0,94**	1,20	0,26	0,83	0,40	905	**0,92**	1,18	3,00	0,24	0,03
**PA4640**	59826	6,49	1282,30	27	49,09	0,85	-0,24	-0,91	0,36	869	**0,91**	0,90	-0,15	-0,66	0,51	1140	**0,96**	0,84	-0,25	-0,90	0,37	835	**0,91**	0,86	3,00	-0,21	0,03
**PA4642**	12091	5,12	272,12	5	59,18	1,13	0,17	0,53	0,60	1309	**0,99**	1,31	0,39	1,35	0,18	547	**0,69**	1,02	0,03	0,05	0,96	1979	**1,00**	1,15	3,00	0,21	0,15
**PA4644**	17957	5,59	263,31	6	33,73	0,66	-0,61	-2,22	0,03	251	**0,23**	0,72	-0,47	-1,84	0,07	378	**0,38**	0,73	-0,45	-1,60	0,11	420	**0,54**	0,70	3,00	-0,51	0,04
**PA4645**	22251	5,50	359,36	9	37,29	0,60	-0,73	-2,64	0,01	183	**0,10**	0,59	-0,77	-2,98	0,00	179	**0,04**	0,62	-0,69	-2,39	0,02	238	**0,14**	0,60	3,00	-0,73	0,02
**PA4646**	25373	6,45	833,03	16	48,05	0,97	-0,05	-0,25	0,80	1773	**0,98**	1,01	0,02	-0,03	0,97	2111	**1,00**	1,01	0,01	-0,02	0,98	2034	**1,00**	1,00	3,00	-0,01	0,02
**PA4655**	40074	6,38	157,62	5	10,06	0,84	-0,25	-0,96	0,34	808	**0,90**	0,90	-0,15	-0,67	0,50	1129	**0,96**	1,00	0,00	-0,07	0,95	1951	**1,00**	0,91	3,00	-0,13	0,08
**PA4661**	19679	5,87	363,49	7	38,89	0,79	-0,34	-1,26	0,21	586	**0,77**	0,75	-0,42	-1,65	0,10	437	**0,49**	0,95	-0,08	-0,33	0,74	1533	**1,00**	0,83	3,00	-0,27	0,10
**PA4662**	29496	5,42	343,42	8	31,98	1,10	0,13	0,39	0,70	1536	**0,99**	1,30	0,38	1,32	0,19	560	**0,72**	1,14	0,19	0,59	0,56	1192	**0,96**	1,18	3,00	0,24	0,11
**PA4663**	27712	5,54	197,77	6	17,87	1,00	0,00	-0,08	0,94	2042	**1,00**	1,08	0,11	0,30	0,77	1689	**0,98**	1,08	0,10	0,30	0,77	1581	**1,00**	1,05	3,00	0,07	0,04
**PA4664**	31136	5,33	154,31	4	16,93	1,00	0,00	-0,07	0,94	2048	**1,00**	1,24	0,31	1,07	0,29	737	**0,84**	1,16	0,21	0,67	0,50	1093	**0,95**	1,13	3,00	0,18	0,12
**PA4665**	42270	5,10	436,21	8	23,30	1,18	0,24	0,75	0,45	1021	**0,96**	1,07	0,10	0,26	0,79	1751	**0,98**	1,23	0,30	0,94	0,35	806	**0,88**	1,16	3,00	0,21	0,08
**PA4666**	48395	5,55	602,78	14	36,30	1,12	0,16	0,49	0,63	1381	**0,98**	1,16	0,21	0,69	0,49	1103	**0,96**	1,06	0,09	0,23	0,82	1671	**1,01**	1,11	3,00	0,15	0,05
**PA4667**	70377	5,07	798,55	20	32,55	1,04	0,06	0,13	0,90	1967	**0,99**	1,09	0,12	0,35	0,73	1619	**0,97**	1,06	0,09	0,23	0,82	1670	**1,01**	1,06	3,00	0,09	0,02
**PA4668**	24069	6,92	345,10	9	42,47	1,03	0,04	0,06	0,95	2074	**1,00**	1,15	0,20	0,64	0,52	1175	**0,96**	1,22	0,29	0,93	0,35	812	**0,89**	1,13	3,00	0,18	0,10
**PA4669**	32357	6,14	49,39	2	7,10	1,21	0,28	0,89	0,37	882	**0,92**	1,03	0,05	0,07	0,94	2055	**0,99**	1,05	0,06	0,16	0,87	1799	**1,00**	1,10	3,00	0,13	0,10
**PA4670**	35900	6,12	2063,86	38	67,02	1,19	0,25	0,78	0,43	991	**0,95**	1,30	0,38	1,30	0,19	569	**0,73**	1,18	0,24	0,74	0,46	1014	**0,93**	1,22	3,00	0,29	0,07
**PA4671**	24012	5,81	1821,73	34	83,09	0,92	-0,12	-0,49	0,62	1377	**0,98**	0,95	-0,07	-0,37	0,71	1574	**0,97**	0,96	-0,06	-0,25	0,80	1645	**1,00**	0,94	3,00	-0,08	0,02
**PA4672**	21912	9,34	297,21	8	43,80	1,20	0,26	0,84	0,40	934	**0,93**	1,04	0,05	0,09	0,93	2027	**0,99**	1,05	0,07	0,20	0,85	1740	**1,00**	1,10	3,00	0,13	0,09
**PA4673**	44173	5,02	1531,02	30	55,79	1,09	0,12	0,36	0,72	1592	**0,99**	0,99	-0,02	-0,16	0,87	1926	**0,98**	1,10	0,14	0,43	0,67	1376	**1,00**	1,06	3,00	0,09	0,06
**PA4675**	85371	5,33	1350,97	34	34,34	2,08	1,06	3,62	0,00	112	**0,01**	2,14	1,10	3,99	0,00	118	**0,00**	1,90	0,93	3,09	0,00	161	**0,03**	2,04	3,00	1,03	0,12
**PA4676**	25539	5,67	275,54	7	34,71	0,91	-0,14	-0,58	0,56	1241	**0,98**	0,98	-0,03	-0,19	0,85	1870	**0,98**	0,88	-0,19	-0,70	0,49	1059	**0,95**	0,92	3,00	-0,12	0,05
**PA4679**	25639	5,30	66,17	2	15,90	1,09	0,12	0,34	0,73	1611	**0,99**	0,85	-0,24	-0,99	0,32	800	**0,87**	0,89	-0,17	-0,64	0,52	1126	**0,96**	0,94	3,00	-0,09	0,13
**PA4684**	50979	6,82	197,64	5	10,20	1,46	0,54	1,83	0,07	328	**0,45**	1,56	0,64	2,30	0,02	268	**0,17**	1,76	0,81	2,70	0,01	205	**0,07**	1,59	3,00	0,67	0,15
**PA4685**	27151	4,52	200,78	4	20,30	1,84	0,88	3,00	0,00	154	**0,04**	4,45	2,15	7,92	0,00	30	**0,00**	3,23	1,69	5,68	0,00	67	**0,00**	3,17	3,00	1,67	1,31

**PA4686**	112763	5,37	1007,03	30	27,35	1,36	0,44	1,47	0,14	473	**0,65**	1,41	0,50	1,76	0,08	399	**0,43**	1,38	0,46	1,52	0,13	455	**0,59**	1,38	3,00	0,47	0,03
**PA4687**	39487	5,54	1790,44	32	63,45	1,01	0,01	-0,04	0,96	2104	**1,00**	1,09	0,12	0,36	0,72	1601	**0,97**	1,15	0,20	0,64	0,52	1130	**0,96**	1,08	3,00	0,11	0,07
**PA4688**	57158	10,04	103,57	2	4,30	1,20	0,27	0,86	0,39	914	**0,93**	1,08	0,11	0,30	0,76	1684	**0,98**	1,06	0,09	0,24	0,81	1664	**1,00**	1,11	3,00	0,16	0,08
**PA4689**	90109	8,96	522,88	13	16,94	1,02	0,02	0,00	1,00	2170	**1,00**	1,07	0,10	0,27	0,79	1744	**0,98**	1,08	0,11	0,33	0,74	1530	**1,00**	1,06	3,00	0,08	0,04
**PA4694**	39992	5,57	1448,77	30	53,22	0,83	-0,27	-1,03	0,30	748	**0,88**	0,91	-0,13	-0,60	0,55	1222	**0,97**	0,83	-0,27	-0,98	0,33	773	**0,87**	0,86	3,00	-0,22	0,05
**PA4695**	19071	5,93	476,52	8	48,76	0,93	-0,11	-0,45	0,65	1441	**0,98**	0,95	-0,08	-0,39	0,70	1550	**0,97**	0,93	-0,10	-0,39	0,70	1435	**1,00**	0,94	3,00	-0,09	0,01
**PA4696**	67695	6,30	717,15	20	26,06	0,93	-0,10	-0,42	0,67	1486	**0,98**	0,95	-0,08	-0,39	0,69	1547	**0,97**	0,91	-0,14	-0,52	0,60	1255	**0,99**	0,93	3,00	-0,10	0,02
**PA4699**	26865	9,72	146,00	3	11,43	1,15	0,20	0,63	0,53	1158	**0,99**	0,99	-0,02	-0,15	0,88	1937	**0,98**	1,00	0,00	-0,07	0,94	1947	**1,00**	1,05	3,00	0,07	0,09
**PA4700**	89561	9,24	500,96	12	15,63	1,08	0,11	0,31	0,76	1663	**0,99**	1,15	0,21	0,68	0,50	1122	**0,96**	1,12	0,16	0,48	0,63	1302	**1,00**	1,12	3,00	0,16	0,04
**PA4701**	61558	5,71	1522,94	33	57,00	0,79	-0,33	-1,25	0,21	596	**0,77**	0,82	-0,29	-1,19	0,23	638	**0,79**	0,78	-0,36	-1,29	0,20	561	**0,72**	0,80	3,00	-0,33	0,02
**PA4708**	32604	6,86	430,62	10	39,13	4,68	2,23	7,74	0,00	26	**0,00**	3,14	1,65	6,05	0,00	62	**0,00**	3,46	1,79	6,01	0,00	58	**0,00**	3,76	3,00	1,91	0,81
**PA4709**	40900	5,50	343,46	9	28,51	4,79	2,26	7,86	0,00	24	**0,00**	2,52	1,33	4,86	0,00	88	**0,00**	4,48	2,16	7,28	0,00	44	**0,00**	3,93	3,00	1,98	1,24
**PA4710**	90195	5,72	978,12	21	24,85	4,95	2,31	8,02	0,00	22	**0,00**	4,57	2,19	8,08	0,00	27	**0,00**	5,32	2,41	8,11	0,00	35	**0,00**	4,95	3,00	2,31	0,37
**PA4715**	48767	7,74	488,84	12	21,02	0,93	-0,10	-0,44	0,66	1459	**0,99**	1,11	0,15	0,44	0,66	1470	**0,97**	1,00	0,00	-0,04	0,97	1991	**1,00**	1,01	3,00	0,02	0,09
**PA4718**	17711	9,90	104,29	3	28,87	1,02	0,03	0,03	0,98	2135	**1,00**	1,42	0,50	1,78	0,08	394	**0,41**	1,11	0,15	0,44	0,66	1354	**1,00**	1,18	3,00	0,24	0,21
**PA4720**	43284	6,48	425,09	9	33,33	0,99	-0,01	-0,12	0,91	1979	**0,99**	1,00	0,00	-0,08	0,93	2037	**0,99**	1,05	0,07	0,16	0,87	1789	**1,00**	1,01	3,00	0,02	0,03
**PA4722**	43687	5,53	370,49	9	21,97	0,93	-0,11	-0,47	0,64	1400	**0,99**	0,92	-0,12	-0,53	0,60	1330	**0,97**	0,93	-0,10	-0,40	0,69	1410	**1,01**	0,93	3,00	-0,11	0,00
**PA4723**	19103	5,04	504,74	13	49,30	0,87	-0,21	-0,81	0,42	965	**0,94**	0,90	-0,15	-0,65	0,52	1158	**0,96**	0,80	-0,33	-1,17	0,24	627	**0,79**	0,85	3,00	-0,23	0,05
**PA4724**	33247	6,11	88,03	3	12,60	1,13	0,17	0,52	0,60	1320	**0,99**	0,96	-0,07	-0,34	0,73	1626	**0,97**	0,88	-0,19	-0,71	0,48	1050	**0,94**	0,99	3,00	-0,02	0,13
**PA4726**	55176	5,51	574,27	13	32,20	1,01	0,02	-0,02	0,98	2153	**0,99**	1,07	0,09	0,25	0,80	1769	**0,98**	0,97	-0,04	-0,18	0,86	1766	**1,00**	1,02	3,00	0,03	0,05
**PA4727**	55810	9,91	699,93	19	33,58	1,41	0,49	1,65	0,10	397	**0,55**	1,46	0,55	1,93	0,05	347	**0,33**	1,30	0,37	1,21	0,23	605	**0,77**	1,39	3,00	0,47	0,08
**PA4728**	18337	4,79	447,30	8	35,25	1,25	0,33	1,07	0,29	718	**0,86**	1,15	0,20	0,64	0,52	1178	**0,96**	1,74	0,80	2,64	0,01	211	**0,08**	1,38	3,00	0,46	0,31
**PA4729**	29625	5,57	669,15	13	40,91	0,76	-0,39	-1,46	0,14	478	**0,65**	0,98	-0,04	-0,23	0,82	1813	**0,98**	1,06	0,09	0,24	0,81	1660	**1,00**	0,93	3,00	-0,10	0,16
**PA4730**	32062	6,11	553,76	14	33,33	0,86	-0,21	-0,82	0,41	963	**0,93**	0,89	-0,16	-0,70	0,49	1094	**0,96**	0,99	-0,02	-0,13	0,90	1848	**1,00**	0,91	3,00	-0,13	0,06
**PA4732**	64827	6,10	596,34	14	28,64	0,98	-0,02	-0,17	0,87	1910	**0,99**	0,96	-0,06	-0,31	0,75	1662	**0,98**	0,90	-0,15	-0,56	0,58	1216	**0,98**	0,95	3,00	-0,08	0,04
**PA4735**	129372	5,32	714,46	20	17,56	1,00	0,00	-0,08	0,94	2040	**1,00**	0,91	-0,13	-0,58	0,56	1263	**0,97**	0,96	-0,06	-0,26	0,79	1633	**1,00**	0,96	3,00	-0,06	0,04
**PA4739**	13771	8,93	235,04	6	30,40	0,90	-0,16	-0,64	0,52	1154	**0,99**	0,86	-0,21	-0,89	0,38	876	**0,93**	0,94	-0,08	-0,34	0,74	1521	**1,00**	0,90	3,00	-0,15	0,04
**PA4740**	81322	5,07	3512,26	77	61,36	1,01	0,01	-0,04	0,97	2117	**1,00**	0,95	-0,08	-0,40	0,69	1536	**0,97**	0,94	-0,08	-0,34	0,73	1507	**1,00**	0,97	3,00	-0,05	0,04
**PA4741**	11406	10,06	480,67	11	62,17	0,86	-0,22	-0,84	0,40	937	**0,94**	1,17	0,22	0,73	0,47	1051	**0,96**	1,28	0,36	1,15	0,25	638	**0,81**	1,10	3,00	0,14	0,22
**PA4742**	35271	6,43	689,15	13	48,05	1,03	0,04	0,05	0,96	2096	**0,99**	0,92	-0,12	-0,56	0,57	1289	**0,96**	0,97	-0,05	-0,21	0,83	1707	**1,00**	0,97	3,00	-0,04	0,05
**PA4743**	15547	6,08	437,79	11	62,00	1,07	0,09	0,24	0,81	1782	**0,99**	0,96	-0,06	-0,31	0,76	1670	**0,98**	0,86	-0,22	-0,81	0,42	929	**0,93**	0,96	3,00	-0,06	0,10
**PA4744**	99739	5,83	4087,46	80	62,39	0,97	-0,04	-0,21	0,83	1827	**0,99**	0,89	-0,17	-0,73	0,46	1047	**0,96**	0,94	-0,09	-0,38	0,71	1450	**1,00**	0,93	3,00	-0,10	0,04
**PA4745**	57986	4,55	2071,12	44	50,02	0,89	-0,17	-0,67	0,50	1124	**0,97**	0,95	-0,08	-0,40	0,69	1538	**0,97**	0,90	-0,15	-0,55	0,58	1218	**0,98**	0,91	3,00	-0,13	0,03
**PA4746**	17941	4,61	145,06	2	25,00	1,10	0,13	0,39	0,70	1545	**0,98**	1,07	0,10	0,27	0,79	1739	**0,98**	0,95	-0,07	-0,30	0,76	1576	**1,00**	1,04	3,00	0,06	0,08
**PA4747**	14496	5,21	255,90	5	40,46	1,15	0,20	0,61	0,54	1194	**0,99**	1,02	0,03	0,03	0,97	2117	**1,00**	1,34	0,42	1,36	0,17	537	**0,67**	1,17	3,00	0,22	0,16
**PA4748**	26988	5,44	567,83	10	39,52	0,96	-0,06	-0,28	0,78	1717	**0,99**	1,10	0,13	0,39	0,69	1545	**0,97**	1,06	0,08	0,21	0,84	1716	**1,00**	1,04	3,00	0,05	0,07
**PA4749**	50728	5,69	579,06	16	33,93	1,13	0,17	0,52	0,61	1331	**0,99**	1,14	0,18	0,59	0,56	1238	**0,97**	1,07	0,09	0,26	0,80	1642	**1,00**	1,11	3,00	0,15	0,04
**PA4750**	31329	5,43	440,03	11	38,81	1,08	0,11	0,31	0,76	1670	**0,99**	1,19	0,26	0,86	0,39	913	**0,93**	1,10	0,13	0,39	0,70	1429	**1,00**	1,12	3,00	0,17	0,06
**PA4751**	74850	5,62	1556,65	37	47,94	1,03	0,04	0,07	0,94	2053	**1,00**	1,04	0,06	0,13	0,89	1970	**0,98**	1,10	0,13	0,40	0,69	1418	**1,00**	1,06	3,00	0,08	0,04
**PA4752**	24939	7,03	82,60	2	23,70	0,93	-0,10	-0,42	0,67	1487	**0,98**	0,68	-0,56	-2,20	0,03	287	**0,21**	0,89	-0,16	-0,61	0,54	1160	**0,96**	0,83	3,00	-0,26	0,14
**PA4753**	13281	9,41	88,69	3	37,50	0,64	-0,64	-2,34	0,02	226	**0,18**	0,80	-0,33	-1,33	0,18	557	**0,71**	0,87	-0,20	-0,73	0,46	1026	**0,93**	0,77	3,00	-0,38	0,12
**PA4755**	18589	4,94	653,57	18	62,35	0,95	-0,07	-0,34	0,74	1620	**0,99**	0,89	-0,17	-0,72	0,47	1065	**0,96**	0,91	-0,14	-0,52	0,60	1254	**0,99**	0,92	3,00	-0,12	0,03
**PA4756**	126672	5,05	2573,70	62	43,52	0,81	-0,30	-1,12	0,26	679	**0,83**	0,82	-0,29	-1,16	0,25	654	**0,81**	0,80	-0,33	-1,18	0,24	625	**0,79**	0,81	3,00	-0,30	0,01
**PA4758**	42698	5,30	611,25	14	32,34	0,92	-0,11	-0,48	0,63	1392	**0,99**	0,82	-0,29	-1,17	0,24	644	**0,81**	0,82	-0,28	-1,01	0,31	750	**0,86**	0,86	3,00	-0,23	0,06
**PA4759**	30030	5,75	925,48	20	69,16	0,93	-0,11	-0,47	0,64	1416	**0,98**	0,92	-0,12	-0,56	0,58	1291	**0,96**	0,90	-0,16	-0,60	0,55	1177	**0,96**	0,91	3,00	-0,13	0,02
**PA4760**	44153	7,03	557,79	15	30,20	0,86	-0,21	-0,82	0,41	950	**0,94**	0,91	-0,13	-0,59	0,56	1241	**0,97**	0,92	-0,12	-0,46	0,64	1320	**1,00**	0,90	3,00	-0,15	0,03
**PA4761**	76091	4,79	6130,66	119	73,03	0,85	-0,24	-0,92	0,36	856	**0,91**	0,91	-0,14	-0,61	0,54	1212	**0,97**	0,91	-0,14	-0,52	0,60	1251	**0,99**	0,89	3,00	-0,17	0,04
**PA4762**	21986	4,49	894,70	20	62,28	0,93	-0,11	-0,47	0,64	1401	**0,98**	0,99	-0,01	-0,15	0,88	1947	**0,98**	0,95	-0,07	-0,29	0,77	1590	**1,00**	0,96	3,00	-0,06	0,03

**PA4763**	63056	5,01	116,56	3	6,47	0,91	-0,13	-0,54	0,59	1300	**0,99**	0,87	-0,20	-0,83	0,41	944	**0,93**	0,97	-0,04	-0,20	0,84	1729	**1,00**	0,92	3,00	-0,12	0,05
**PA4764**	16712	5,62	735,49	20	59,98	1,08	0,12	0,33	0,74	1633	**0,99**	1,23	0,30	1,03	0,30	761	**0,86**	1,13	0,17	0,53	0,60	1243	**0,99**	1,15	3,00	0,20	0,08
**PA4765**	20483	4,79	403,39	9	37,46	0,84	-0,25	-0,95	0,34	823	**0,91**	0,79	-0,34	-1,36	0,17	543	**0,69**	1,15	0,20	0,62	0,54	1156	**0,96**	0,93	3,00	-0,11	0,19
**PA4767**	17281	6,73	76,85	2	13,20	0,99	-0,02	-0,15	0,88	1936	**0,99**	1,07	0,10	0,26	0,79	1749	**0,98**	0,90	-0,15	-0,56	0,58	1217	**0,98**	0,99	3,00	-0,02	0,08
**PA4768**	21283	9,77	135,07	4	29,90	0,98	-0,03	-0,17	0,87	1903	**0,99**	1,08	0,11	0,32	0,75	1661	**0,98**	1,05	0,07	0,19	0,85	1755	**1,00**	1,04	3,00	0,05	0,05
**PA4771**	43016	6,04	516,39	13	31,34	0,46	-1,13	-4,06	0,00	94	**0,00**	0,36	-1,48	-5,60	0,00	70	**0,00**	0,35	-1,50	-5,15	0,00	80	**0,00**	0,39	3,00	-1,36	0,06
**PA4774**	40204	4,67	173,29	4	22,28	0,68	-0,55	-2,01	0,04	277	**0,35**	0,60	-0,73	-2,83	0,00	195	**0,05**	0,58	-0,80	-2,76	0,01	197	**0,06**	0,62	3,00	-0,69	0,06
**PA4776**	25100	5,53	319,65	6	32,55	1,03	0,05	0,09	0,93	2027	**1,00**	0,86	-0,21	-0,89	0,37	872	**0,92**	0,99	-0,02	-0,12	0,90	1856	**1,00**	0,96	3,00	-0,06	0,09
**PA4778**	16342	7,74	84,28	3	15,90	0,81	-0,31	-1,18	0,24	645	**0,81**	0,81	-0,30	-1,21	0,23	624	**0,78**	0,82	-0,29	-1,05	0,29	708	**0,85**	0,81	3,00	-0,30	0,01
**PA4808**	51748	9,06	304,12	7	17,13	0,69	-0,53	-1,92	0,05	296	**0,40**	0,73	-0,44	-1,76	0,08	400	**0,43**	0,84	-0,26	-0,93	0,35	817	**0,89**	0,76	3,00	-0,40	0,07
**PA4809**	35422	5,61	241,48	6	21,50	0,77	-0,39	-1,43	0,15	496	**0,66**	0,96	-0,05	-0,29	0,77	1698	**0,98**	0,73	-0,45	-1,59	0,11	422	**0,54**	0,82	3,00	-0,29	0,13
**PA4839**	74503	5,27	944,71	23	35,38	1,28	0,36	1,17	0,24	650	**0,81**	1,23	0,30	1,03	0,30	768	**0,85**	1,23	0,30	0,94	0,35	804	**0,89**	1,25	3,00	0,32	0,03
**PA4840**	14727	9,37	157,34	4	31,88	1,08	0,11	0,30	0,76	1680	**0,99**	1,22	0,28	0,96	0,34	817	**0,89**	0,94	-0,08	-0,34	0,74	1520	**1,00**	1,08	3,00	0,11	0,14
**PA4841**	20147	4,70	62,68	2	17,40	1,46	0,55	1,85	0,06	315	**0,44**	0,88	-0,18	-0,78	0,44	1008	**0,94**	0,94	-0,10	-0,38	0,70	1446	**1,00**	1,09	3,00	0,13	0,32
**PA4842**	41838	5,24	270,21	5	16,10	0,91	-0,13	-0,55	0,58	1286	**0,99**	0,86	-0,22	-0,92	0,36	845	**0,91**	0,72	-0,46	-1,63	0,10	403	**0,53**	0,83	3,00	-0,27	0,10
**PA4843**	64195	5,95	151,30	5	5,44	1,58	0,66	2,24	0,02	246	**0,22**	1,40	0,48	1,69	0,09	424	**0,46**	1,21	0,28	0,88	0,38	861	**0,91**	1,40	3,00	0,48	0,19
**PA4845**	65062	8,40	141,05	3	7,77	0,91	-0,13	-0,54	0,59	1297	**0,99**	0,99	-0,02	-0,16	0,87	1931	**0,98**	0,95	-0,07	-0,29	0,77	1592	**1,00**	0,95	3,00	-0,07	0,04
**PA4847**	17787	4,97	693,46	13	53,35	0,90	-0,15	-0,61	0,54	1184	**0,99**	0,92	-0,12	-0,54	0,59	1318	**0,97**	0,96	-0,06	-0,25	0,80	1655	**1,00**	0,93	3,00	-0,11	0,03
**PA4848**	52782	5,92	2196,41	44	55,16	0,96	-0,05	-0,26	0,79	1742	**0,99**	0,94	-0,09	-0,43	0,67	1490	**0,97**	0,92	-0,12	-0,46	0,65	1329	**1,00**	0,94	3,00	-0,09	0,02
**PA4850**	32216	4,41	206,80	5	13,80	0,98	-0,03	-0,18	0,86	1883	**0,99**	1,14	0,19	0,62	0,54	1205	**0,96**	0,91	-0,13	-0,50	0,61	1274	**0,99**	1,01	3,00	0,02	0,12
**PA4851**	48275	4,73	195,09	7	14,50	1,00	0,00	-0,09	0,93	2011	**1,00**	0,88	-0,18	-0,76	0,44	1019	**0,94**	0,95	-0,07	-0,31	0,76	1562	**1,00**	0,94	3,00	-0,08	0,06
**PA4853**	13103	6,04	141,60	3	42,10	0,80	-0,33	-1,24	0,22	607	**0,77**	1,05	0,07	0,18	0,86	1901	**0,98**	0,98	-0,03	-0,16	0,88	1804	**1,00**	0,94	3,00	-0,09	0,13
**PA4854**	62183	6,06	1706,33	43	63,37	1,17	0,23	0,72	0,47	1051	**0,97**	1,26	0,34	1,16	0,24	652	**0,81**	1,28	0,35	1,15	0,25	641	**0,81**	1,24	3,00	0,31	0,06
**PA4855**	48106	4,94	1447,38	25	50,85	1,14	0,18	0,57	0,57	1264	**0,98**	1,09	0,13	0,37	0,71	1576	**0,97**	1,09	0,13	0,37	0,71	1459	**1,00**	1,11	3,00	0,15	0,03
**PA4856**	108502	6,08	154,69	5	5,26	1,04	0,05	0,11	0,91	1986	**1,00**	1,07	0,10	0,27	0,78	1735	**0,98**	1,11	0,15	0,44	0,66	1364	**1,00**	1,07	3,00	0,10	0,03
**PA4872**	32540	5,42	406,07	7	32,10	0,85	-0,24	-0,91	0,36	864	**0,92**	1,02	0,03	0,03	0,97	2116	**1,00**	0,88	-0,18	-0,67	0,50	1094	**0,95**	0,92	3,00	-0,12	0,09
**PA4873**	48623	5,25	177,20	4	8,30	0,80	-0,33	-1,23	0,22	610	**0,78**	0,54	-0,89	-3,43	0,00	146	**0,01**	0,51	-0,98	-3,38	0,00	141	**0,01**	0,61	3,00	-0,70	0,16
**PA4887**	48943	6,30	112,04	3	5,50	1,38	0,46	1,55	0,12	441	**0,60**	0,88	-0,18	-0,77	0,44	1013	**0,94**	1,30	0,38	1,22	0,22	598	**0,77**	1,19	3,00	0,25	0,27
**PA4889**	40723	8,71	177,05	5	11,30	0,71	-0,49	-1,80	0,07	338	**0,47**	0,64	-0,65	-2,52	0,01	227	**0,11**	0,61	-0,72	-2,48	0,01	227	**0,12**	0,65	3,00	-0,62	0,05
**PA4890**	23750	8,20	192,63	5	26,36	0,90	-0,15	-0,60	0,55	1205	**0,98**	0,80	-0,32	-1,28	0,20	583	**0,74**	0,93	-0,10	-0,39	0,70	1436	**1,00**	0,88	3,00	-0,18	0,07
**PA4907**	28415	5,26	1026,82	18	57,30	0,93	-0,11	-0,45	0,65	1436	**0,98**	1,01	0,01	-0,04	0,97	2103	**0,99**	0,88	-0,19	-0,69	0,49	1067	**0,94**	0,94	3,00	-0,09	0,07
**PA4916**	26398	6,08	180,29	4	17,60	1,05	0,07	0,16	0,88	1931	**0,99**	0,83	-0,27	-1,09	0,28	721	**0,83**	0,93	-0,10	-0,41	0,69	1406	**1,00**	0,94	3,00	-0,09	0,11
**PA4918**	25425	4,94	93,71	4	12,35	0,86	-0,22	-0,85	0,39	919	**0,93**	0,92	-0,12	-0,55	0,58	1309	**0,96**	0,88	-0,18	-0,68	0,50	1087	**0,95**	0,89	3,00	-0,17	0,03
**PA4919**	48359	5,93	684,16	18	37,95	1,00	0,00	-0,07	0,94	2049	**1,00**	1,04	0,05	0,11	0,92	2008	**0,99**	0,94	-0,08	-0,34	0,73	1511	**1,00**	0,99	3,00	-0,01	0,05
**PA4920**	31500	5,42	1371,95	29	63,09	0,98	-0,04	-0,21	0,84	1840	**0,99**	0,95	-0,07	-0,36	0,72	1603	**0,97**	1,09	0,12	0,35	0,72	1489	**1,00**	1,01	3,00	0,01	0,07
**PA4922**	18009	6,39	121,44	4	26,00	1,24	0,32	1,03	0,30	753	**0,88**	0,92	-0,12	-0,55	0,58	1305	**0,97**	1,07	0,09	0,26	0,80	1644	**1,00**	1,08	3,00	0,11	0,16
**PA4923**	21524	5,32	133,33	3	15,90	1,05	0,07	0,15	0,88	1939	**0,99**	0,84	-0,26	-1,05	0,29	749	**0,84**	0,81	-0,30	-1,08	0,28	685	**0,84**	0,90	3,00	-0,15	0,13
**PA4928**	91876	9,09	183,66	7	9,11	1,02	0,03	0,03	0,97	2132	**0,99**	1,12	0,17	0,53	0,60	1324	**0,97**	1,09	0,13	0,39	0,70	1437	**1,00**	1,08	3,00	0,11	0,05
**PA4930**	39427	5,78	430,51	10	34,05	0,92	-0,13	-0,52	0,60	1321	**0,99**	0,90	-0,15	-0,67	0,51	1131	**0,97**	0,88	-0,18	-0,68	0,50	1086	**0,95**	0,90	3,00	-0,15	0,02
**PA4931**	54774	4,98	784,71	18	36,95	0,93	-0,10	-0,43	0,66	1463	**0,99**	1,00	0,00	-0,11	0,91	2003	**0,98**	0,96	-0,05	-0,24	0,81	1665	**1,00**	0,96	3,00	-0,05	0,03
**PA4932**	17107	5,47	1071,79	19	77,00	0,95	-0,07	-0,34	0,74	1621	**0,99**	0,97	-0,05	-0,29	0,77	1705	**0,98**	0,92	-0,12	-0,47	0,64	1312	**1,00**	0,94	3,00	-0,08	0,02
**PA4934**	10211	10,48	335,29	8	61,80	0,89	-0,17	-0,66	0,51	1127	**0,98**	0,91	-0,14	-0,62	0,53	1199	**0,96**	0,91	-0,13	-0,51	0,61	1265	**1,00**	0,90	3,00	-0,15	0,01
**PA4935**	17065	4,87	1059,98	24	66,68	0,99	-0,01	-0,11	0,91	1982	**1,00**	0,94	-0,09	-0,44	0,66	1483	**0,96**	0,93	-0,10	-0,39	0,70	1434	**1,01**	0,96	3,00	-0,07	0,03
**PA4936**	28833	6,67	421,57	7	28,20	1,08	0,12	0,33	0,74	1628	**0,99**	1,07	0,09	0,25	0,80	1772	**0,98**	1,06	0,08	0,22	0,83	1698	**1,01**	1,07	3,00	0,10	0,01
**PA4937**	108434	9,04	1657,75	40	33,60	1,07	0,10	0,27	0,79	1735	**0,98**	0,99	-0,01	-0,14	0,89	1957	**0,98**	1,07	0,10	0,29	0,77	1593	**1,00**	1,05	3,00	0,06	0,05
**PA4938**	50185	5,70	1203,32	27	48,72	1,07	0,10	0,26	0,79	1747	**0,99**	0,99	-0,02	-0,16	0,87	1922	**0,98**	0,96	-0,05	-0,24	0,81	1666	**1,00**	1,01	3,00	0,01	0,06
**PA4939**	43234	5,03	1000,42	22	42,15	1,16	0,22	0,68	0,50	1104	**0,98**	1,08	0,11	0,29	0,77	1692	**0,98**	1,15	0,21	0,64	0,52	1123	**0,96**	1,13	3,00	0,18	0,05
**PA4941**	35977	9,48	736,74	22	44,35	1,08	0,11	0,31	0,75	1654	**0,99**	1,03	0,05	0,08	0,93	2034	**0,99**	1,09	0,12	0,35	0,72	1487	**1,00**	1,07	3,00	0,09	0,03

**PA4942**	46180	5,15	1689,93	26	48,37	1,09	0,12	0,35	0,72	1594	**0,99**	1,10	0,14	0,42	0,67	1508	**0,97**	1,11	0,15	0,47	0,64	1318	**1,00**	1,10	3,00	0,14	0,01
**PA4943**	50525	5,97	210,77	7	14,27	1,02	0,03	0,02	0,98	2144	**1,00**	0,94	-0,10	-0,46	0,65	1449	**0,97**	0,97	-0,05	-0,23	0,82	1681	**1,00**	0,97	3,00	-0,04	0,04
**PA4946**	72050	6,10	599,40	16	27,16	1,06	0,09	0,24	0,81	1785	**0,99**	1,16	0,21	0,69	0,49	1115	**0,96**	1,05	0,07	0,18	0,86	1777	**1,00**	1,09	3,00	0,12	0,06
**PA4947**	53149	9,80	575,59	14	27,74	1,05	0,08	0,18	0,85	1881	**0,99**	1,14	0,19	0,62	0,54	1207	**0,96**	1,22	0,29	0,92	0,36	822	**0,90**	1,14	3,00	0,19	0,08
**PA4948**	16977	4,92	99,83	2	9,35	1,14	0,19	0,57	0,57	1254	**0,98**	1,04	0,06	0,13	0,90	1973	**0,98**	0,97	-0,04	-0,19	0,85	1753	**1,00**	1,05	3,00	0,07	0,08
**PA4949**	52561	7,21	449,79	11	21,10	1,19	0,26	0,82	0,41	959	**0,94**	0,91	-0,13	-0,60	0,55	1220	**0,97**	0,95	-0,08	-0,32	0,75	1547	**1,00**	1,02	3,00	0,02	0,15
**PA4951**	21770	5,48	119,78	4	17,23	1,04	0,06	0,14	0,89	1951	**0,99**	0,92	-0,13	-0,57	0,57	1266	**0,97**	0,90	-0,15	-0,55	0,58	1221	**0,98**	0,95	3,00	-0,07	0,08
**PA4952**	38438	6,07	350,31	9	21,03	1,08	0,11	0,31	0,75	1652	**0,99**	0,91	-0,14	-0,63	0,53	1196	**0,96**	0,95	-0,07	-0,31	0,76	1566	**1,00**	0,98	3,00	-0,03	0,09
**PA4953**	40589	5,08	121,81	4	16,40	1,19	0,25	0,78	0,43	992	**0,95**	0,85	-0,23	-0,97	0,33	814	**0,89**	1,01	0,01	-0,03	0,98	2020	**1,00**	1,01	3,00	0,02	0,17
**PA4956**	30572	5,30	412,53	10	39,56	0,84	-0,26	-0,99	0,32	786	**0,89**	0,98	-0,02	-0,19	0,85	1875	**0,98**	0,87	-0,21	-0,76	0,45	987	**0,94**	0,90	3,00	-0,16	0,08
**PA4957**	33651	7,70	442,60	10	30,10	1,11	0,15	0,44	0,66	1451	**0,99**	1,07	0,10	0,27	0,79	1743	**0,98**	1,22	0,28	0,90	0,37	831	**0,91**	1,13	3,00	0,18	0,08
**PA4958**	55606	6,65	180,75	5	7,16	0,92	-0,12	-0,51	0,61	1339	**0,99**	0,97	-0,05	-0,28	0,78	1713	**0,98**	0,75	-0,42	-1,48	0,14	475	**0,60**	0,88	3,00	-0,19	0,12
**PA4959**	80637	5,11	529,83	13	17,26	1,02	0,03	0,04	0,97	2116	**0,99**	0,87	-0,20	-0,83	0,41	943	**0,94**	0,87	-0,20	-0,73	0,47	1029	**0,93**	0,92	3,00	-0,12	0,09
**PA4960**	49240	5,14	541,90	14	26,09	1,09	0,13	0,36	0,72	1584	**0,98**	0,91	-0,14	-0,63	0,53	1192	**0,96**	0,98	-0,03	-0,16	0,87	1793	**1,00**	0,99	3,00	-0,01	0,09
**PA4961**	58082	4,84	255,46	7	10,50	0,95	-0,08	-0,35	0,73	1600	**0,99**	0,87	-0,21	-0,87	0,39	900	**0,93**	0,86	-0,22	-0,80	0,42	935	**0,93**	0,89	3,00	-0,17	0,05
**PA4963**	28053	9,07	275,18	8	33,45	1,00	0,01	-0,06	0,95	2078	**1,00**	1,22	0,28	0,96	0,34	823	**0,88**	0,94	-0,08	-0,34	0,74	1519	**1,00**	1,06	3,00	0,08	0,14
**PA4964**	89019	5,76	1157,97	27	34,64	0,90	-0,16	-0,64	0,52	1150	**0,99**	0,85	-0,23	-0,96	0,34	825	**0,89**	0,82	-0,28	-1,01	0,31	749	**0,86**	0,86	3,00	-0,22	0,04
**PA4966**	38033	5,53	272,99	6	20,37	0,87	-0,20	-0,77	0,44	1006	**0,95**	0,83	-0,26	-1,07	0,29	732	**0,84**	0,98	-0,02	-0,14	0,89	1833	**1,00**	0,90	3,00	-0,16	0,08
**PA4967**	75032	5,72	864,03	23	27,43	0,86	-0,21	-0,83	0,41	946	**0,94**	0,88	-0,19	-0,80	0,42	970	**0,94**	0,84	-0,25	-0,91	0,37	829	**0,91**	0,86	3,00	-0,22	0,02
**PA4968**	22966	6,25	93,61	2	15,50	0,74	-0,44	-1,64	0,10	402	**0,55**	1,02	0,02	-0,02	0,99	2139	**1,00**	0,73	-0,45	-1,60	0,11	417	**0,54**	0,83	3,00	-0,27	0,16
**PA4969**	30860	4,94	72,16	2	9,60	0,89	-0,17	-0,69	0,49	1095	**0,97**	0,45	-1,16	-4,41	0,00	102	**0,00**	0,65	-0,61	-2,13	0,03	280	**0,24**	0,66	3,00	-0,59	0,22
**PA4971**	23879	4,95	173,62	6	30,37	0,86	-0,21	-0,83	0,41	943	**0,94**	0,93	-0,11	-0,51	0,61	1360	**0,96**	0,84	-0,26	-0,93	0,35	815	**0,89**	0,87	3,00	-0,19	0,05
**PA4972**	29992	5,98	123,96	3	19,90	1,17	0,23	0,73	0,47	1043	**0,97**	0,91	-0,14	-0,60	0,55	1216	**0,97**	0,99	-0,01	-0,10	0,92	1893	**1,00**	1,02	3,00	0,04	0,13
**PA4973**	75054	5,64	203,33	6	11,93	0,99	-0,02	-0,15	0,88	1940	**0,99**	1,15	0,20	0,66	0,51	1141	**0,96**	1,23	0,30	0,95	0,34	803	**0,88**	1,12	3,00	0,17	0,12
**PA4974**	55967	5,80	1460,42	29	53,08	0,73	-0,46	-1,70	0,09	375	**0,51**	0,77	-0,39	-1,54	0,12	487	**0,55**	0,75	-0,41	-1,44	0,15	501	**0,62**	0,75	3,00	-0,42	0,02
**PA4976**	43245	5,36	40,69	2	6,10	1,38	0,47	1,56	0,12	434	**0,59**	1,63	0,70	2,51	0,01	229	**0,11**	1,64	0,72	2,37	0,02	245	**0,15**	1,55	3,00	0,63	0,15
**PA4991**	44141	7,72	249,23	6	14,30	1,06	0,08	0,20	0,84	1856	**0,99**	1,06	0,08	0,21	0,83	1839	**0,98**	0,90	-0,16	-0,59	0,55	1180	**0,96**	1,00	3,00	0,01	0,09
**PA4992**	30833	6,00	229,22	5	14,16	1,00	0,00	-0,09	0,93	2031	**1,00**	0,99	-0,02	-0,17	0,86	1905	**0,98**	1,10	0,14	0,43	0,67	1373	**1,00**	1,03	3,00	0,04	0,07
**PA4996**	52860	5,88	1219,05	27	45,07	0,88	-0,18	-0,71	0,48	1079	**0,97**	0,89	-0,17	-0,73	0,47	1055	**0,96**	0,84	-0,26	-0,94	0,35	810	**0,89**	0,87	3,00	-0,20	0,03
**PA4997**	69993	6,49	815,18	18	27,73	0,88	-0,18	-0,71	0,48	1069	**0,97**	0,98	-0,02	-0,19	0,85	1882	**0,98**	0,97	-0,04	-0,19	0,85	1750	**1,00**	0,95	3,00	-0,08	0,06
**PA4998**	26359	9,60	123,11	3	19,40	0,71	-0,49	-1,79	0,07	344	**0,47**	0,69	-0,53	-2,09	0,04	309	**0,26**	0,69	-0,53	-1,84	0,07	335	**0,41**	0,70	3,00	-0,51	0,01
**PA5000**	36098	6,92	157,41	5	18,40	1,19	0,26	0,82	0,41	960	**0,94**	0,92	-0,13	-0,57	0,57	1270	**0,97**	1,07	0,10	0,29	0,77	1589	**1,00**	1,06	3,00	0,09	0,14
**PA5001**	38993	8,71	501,63	12	34,60	0,94	-0,09	-0,39	0,70	1539	**0,99**	1,00	0,00	-0,08	0,94	2042	**0,99**	1,10	0,14	0,41	0,68	1402	**1,01**	1,01	3,00	0,02	0,08
**PA5002**	54865	8,67	326,54	8	20,65	1,09	0,13	0,38	0,71	1562	**0,98**	1,05	0,07	0,18	0,86	1894	**0,98**	0,83	-0,28	-0,99	0,32	766	**0,87**	0,99	3,00	-0,01	0,14
**PA5003**	35172	9,56	269,96	4	19,83	1,21	0,27	0,88	0,38	892	**0,92**	0,87	-0,20	-0,85	0,40	917	**0,93**	0,76	-0,40	-1,40	0,16	518	**0,64**	0,95	3,00	-0,08	0,23
**PA5004**	44170	8,73	499,06	13	30,22	0,96	-0,06	-0,30	0,76	1674	**0,99**	0,90	-0,16	-0,69	0,49	1109	**0,96**	0,92	-0,12	-0,47	0,64	1313	**1,00**	0,92	3,00	-0,11	0,03
**PA5005**	71849	6,48	1155,27	23	36,25	0,88	-0,18	-0,72	0,47	1056	**0,97**	0,86	-0,21	-0,89	0,37	867	**0,93**	0,93	-0,10	-0,40	0,69	1409	**1,01**	0,89	3,00	-0,17	0,04
**PA5006**	57255	9,69	307,85	6	12,27	1,28	0,36	1,18	0,24	647	**0,80**	1,08	0,11	0,31	0,76	1669	**0,98**	1,28	0,36	1,16	0,24	633	**0,80**	1,21	3,00	0,28	0,12
**PA5007**	30353	6,46	193,78	4	20,33	1,07	0,10	0,27	0,79	1739	**0,99**	0,96	-0,06	-0,33	0,74	1641	**0,98**	1,18	0,24	0,74	0,46	1010	**0,94**	1,07	3,00	0,10	0,11
**PA5009**	33401	9,87	84,79	3	19,00	1,08	0,11	0,29	0,77	1689	**0,99**	1,14	0,18	0,59	0,56	1236	**0,97**	0,81	-0,30	-1,07	0,29	694	**0,85**	1,01	3,00	0,01	0,17
**PA5010**	44141	7,10	240,11	5	9,40	0,96	-0,06	-0,28	0,78	1724	**0,98**	1,07	0,09	0,25	0,80	1773	**0,98**	1,09	0,12	0,36	0,72	1477	**1,00**	1,04	3,00	0,06	0,07
**PA5011**	41871	9,12	189,17	4	9,98	1,37	0,46	1,53	0,13	452	**0,61**	1,01	0,01	-0,07	0,95	2063	**0,99**	0,93	-0,11	-0,44	0,66	1361	**1,00**	1,10	3,00	0,14	0,24
**PA5012**	40194	8,11	438,04	12	26,05	0,90	-0,15	-0,61	0,54	1185	**0,99**	1,03	0,04	0,06	0,95	2074	**0,99**	1,00	0,00	-0,07	0,94	1944	**1,00**	0,98	3,00	-0,04	0,07
**PA5013**	35838	5,83	1067,12	24	56,70	1,00	-0,01	-0,10	0,92	1993	**1,00**	0,97	-0,05	-0,28	0,78	1714	**0,98**	0,93	-0,11	-0,42	0,68	1391	**1,00**	0,96	3,00	-0,05	0,03
**PA5014**	113377	5,39	444,52	10	11,55	1,11	0,15	0,45	0,65	1440	**0,98**	0,92	-0,12	-0,54	0,59	1310	**0,97**	0,97	-0,04	-0,20	0,84	1721	**1,00**	1,00	3,00	0,00	0,10
**PA5015**	106550	5,56	3357,34	91	49,69	1,53	0,61	2,08	0,04	268	**0,31**	1,47	0,56	1,98	0,05	331	**0,31**	1,42	0,51	1,66	0,10	392	**0,51**	1,47	3,00	0,56	0,05
**PA5016**	62930	5,23	2755,08	58	47,57	1,43	0,51	1,71	0,09	370	**0,51**	1,40	0,49	1,71	0,09	414	**0,45**	1,38	0,46	1,50	0,13	460	**0,59**	1,40	3,00	0,49	0,02
**PA5018**	24695	5,20	154,68	5	24,54	0,74	-0,44	-1,63	0,10	406	**0,56**	0,72	-0,48	-1,88	0,06	361	**0,36**	0,77	-0,38	-1,33	0,18	547	**0,69**	0,74	3,00	-0,43	0,03

**PA5019**	33061	5,72	287,07	7	23,26	0,83	-0,26	-1,01	0,31	769	**0,88**	0,75	-0,41	-1,61	0,11	450	**0,52**	0,87	-0,20	-0,72	0,47	1041	**0,93**	0,82	3,00	-0,29	0,06
**PA5022**	131087	5,96	981,31	24	16,96	1,19	0,25	0,80	0,43	983	**0,94**	1,28	0,36	1,23	0,22	619	**0,77**	1,13	0,17	0,53	0,59	1239	**0,99**	1,20	3,00	0,26	0,08
**PA5025**	46964	6,04	255,48	6	17,97	0,92	-0,12	-0,48	0,63	1384	**0,99**	0,77	-0,38	-1,52	0,13	491	**0,56**	0,87	-0,20	-0,73	0,47	1031	**0,93**	0,85	3,00	-0,23	0,08
**PA5027**	30777	6,30	288,38	7	25,83	0,84	-0,25	-0,96	0,34	813	**0,90**	0,79	-0,35	-1,39	0,17	535	**0,67**	0,98	-0,03	-0,15	0,88	1808	**1,00**	0,87	3,00	-0,20	0,10
**PA5034**	40779	5,93	286,14	9	23,63	1,03	0,05	0,09	0,93	2014	**1,00**	1,05	0,07	0,18	0,86	1889	**0,98**	1,06	0,08	0,22	0,83	1692	**1,01**	1,05	3,00	0,07	0,01
**PA5036**	171891	5,76	95,50	4	5,70	0,64	-0,64	-2,31	0,02	230	**0,20**	0,85	-0,23	-0,97	0,33	812	**0,88**	0,57	-0,81	-2,82	0,00	189	**0,05**	0,69	3,00	-0,54	0,15
**PA5037**	60726	6,62	800,94	17	24,15	0,89	-0,16	-0,65	0,51	1136	**0,98**	1,00	-0,01	-0,12	0,91	1991	**0,98**	0,99	-0,01	-0,11	0,91	1879	**1,00**	0,96	3,00	-0,06	0,06
**PA5038**	41951	5,52	816,38	17	46,12	0,95	-0,07	-0,34	0,73	1613	**0,99**	1,06	0,08	0,21	0,84	1846	**0,98**	0,93	-0,11	-0,42	0,68	1392	**1,00**	0,98	3,00	-0,03	0,07
**PA5040**	83764	5,48	2266,30	55	54,14	0,73	-0,46	-1,69	0,09	384	**0,52**	0,74	-0,44	-1,73	0,08	408	**0,44**	0,72	-0,48	-1,69	0,09	378	**0,50**	0,73	3,00	-0,46	0,01
**PA5041**	20817	9,04	148,41	5	19,46	1,63	0,70	2,38	0,02	220	**0,17**	0,65	-0,63	-2,45	0,01	242	**0,13**	0,65	-0,61	-2,13	0,03	281	**0,24**	0,98	3,00	-0,04	0,56
**PA5042**	24868	5,04	468,61	9	37,59	0,56	-0,84	-3,04	0,00	152	**0,03**	0,50	-1,01	-3,86	0,00	125	**0,00**	0,48	-1,06	-3,65	0,00	127	**0,00**	0,51	3,00	-0,97	0,04
**PA5043**	24073	9,43	508,49	7	40,40	0,54	-0,89	-3,22	0,00	131	**0,02**	0,46	-1,11	-4,25	0,00	109	**0,00**	0,47	-1,09	-3,75	0,00	122	**0,00**	0,49	3,00	-1,03	0,04
**PA5044**	40758	4,83	761,72	17	39,48	0,56	-0,83	-2,98	0,00	156	**0,04**	0,55	-0,86	-3,31	0,00	153	**0,01**	0,52	-0,95	-3,28	0,00	147	**0,01**	0,54	3,00	-0,88	0,02
**PA5045**	96278	5,87	557,46	18	22,15	0,96	-0,06	-0,28	0,78	1713	**0,99**	1,00	0,00	-0,08	0,93	2038	**0,99**	1,02	0,03	0,06	0,95	1962	**1,00**	1,00	3,00	-0,01	0,03
**PA5046**	49710	5,05	2074,16	49	63,63	1,28	0,35	1,15	0,25	659	**0,82**	1,38	0,46	1,62	0,10	447	**0,51**	1,36	0,44	1,43	0,15	503	**0,62**	1,34	3,00	0,42	0,05
**PA5047**	54414	8,45	101,11	4	8,93	1,04	0,05	0,09	0,92	2008	**1,00**	1,14	0,19	0,63	0,53	1195	**0,96**	1,00	0,00	-0,07	0,94	1946	**1,00**	1,06	3,00	0,08	0,08
**PA5049**	9298	8,93	71,17	2	21,10	1,00	0,00	-0,09	0,92	2006	**1,00**	1,12	0,16	0,50	0,62	1390	**0,96**	0,99	-0,02	-0,11	0,91	1874	**1,00**	1,03	3,00	0,05	0,07
**PA5050**	85261	9,02	74,95	2	2,75	1,86	0,89	3,06	0,00	149	**0,03**	1,02	0,02	-0,01	1,00	2153	**1,00**	1,20	0,26	0,82	0,41	922	**0,93**	1,36	3,00	0,44	0,44
**PA5051**	69181	5,35	1058,61	25	42,04	0,92	-0,12	-0,48	0,63	1385	**0,98**	0,98	-0,03	-0,22	0,82	1819	**0,98**	0,94	-0,08	-0,34	0,73	1509	**1,00**	0,95	3,00	-0,08	0,03
**PA5052**	27658	10,19	207,14	7	28,61	1,38	0,47	1,56	0,12	435	**0,59**	1,13	0,18	0,56	0,58	1293	**0,96**	0,94	-0,09	-0,35	0,73	1496	**1,00**	1,15	3,00	0,20	0,22
**PA5053**	19955	6,23	194,14	4	26,58	0,74	-0,44	-1,61	0,11	415	**0,56**	0,94	-0,09	-0,45	0,65	1459	**0,97**	0,66	-0,60	-2,09	0,04	289	**0,26**	0,78	3,00	-0,36	0,14
**PA5054**	54276	5,49	1189,15	28	48,45	0,85	-0,24	-0,93	0,35	843	**0,91**	0,91	-0,13	-0,58	0,56	1258	**0,97**	0,91	-0,14	-0,53	0,60	1244	**0,99**	0,89	3,00	-0,17	0,04
**PA5055**	14798	5,23	103,78	3	32,50	1,17	0,23	0,73	0,47	1044	**0,97**	1,16	0,21	0,70	0,48	1092	**0,96**	1,02	0,02	0,02	0,98	2028	**1,00**	1,12	3,00	0,16	0,09
**PA5060**	38773	10,31	309,91	9	21,97	0,73	-0,46	-1,70	0,09	379	**0,51**	0,72	-0,46	-1,83	0,07	381	**0,38**	0,74	-0,44	-1,54	0,12	445	**0,57**	0,73	3,00	-0,45	0,01
**PA5063**	30876	9,06	850,98	20	58,44	0,88	-0,18	-0,72	0,47	1065	**0,97**	0,84	-0,25	-1,03	0,30	766	**0,85**	0,87	-0,21	-0,76	0,45	985	**0,94**	0,86	3,00	-0,21	0,02
**PA5064**	23710	5,69	195,71	5	27,14	1,00	0,00	-0,10	0,92	2001	**1,00**	1,02	0,02	-0,01	0,99	2149	**1,00**	0,86	-0,23	-0,82	0,41	917	**0,92**	0,96	3,00	-0,06	0,09
**PA5065**	64703	9,12	353,42	10	19,56	0,98	-0,03	-0,19	0,85	1874	**0,99**	1,03	0,04	0,05	0,96	2089	**0,99**	1,00	-0,01	-0,08	0,94	1937	**1,00**	1,00	3,00	0,00	0,02
**PA5067**	13086	5,09	92,36	3	20,40	0,92	-0,12	-0,49	0,62	1378	**0,98**	1,14	0,18	0,59	0,56	1237	**0,97**	1,05	0,07	0,20	0,85	1742	**1,00**	1,04	3,00	0,05	0,11
**PA5071**	27680	8,77	58,36	2	4,70	0,98	-0,04	-0,21	0,84	1831	**0,99**	0,94	-0,08	-0,41	0,68	1517	**0,97**	1,23	0,30	0,97	0,33	783	**0,87**	1,05	3,00	0,07	0,16
**PA5072**	71002	5,07	598,27	13	29,78	1,11	0,15	0,43	0,67	1468	**0,99**	0,89	-0,17	-0,74	0,46	1043	**0,96**	0,85	-0,23	-0,83	0,41	911	**0,92**	0,95	3,00	-0,08	0,14
**PA5074**	28795	5,68	140,93	3	15,20	1,17	0,23	0,72	0,47	1060	**0,97**	1,01	0,01	-0,05	0,96	2088	**0,99**	0,98	-0,02	-0,14	0,89	1835	**1,00**	1,05	3,00	0,08	0,10
**PA5075**	36703	7,93	157,04	5	15,64	0,85	-0,23	-0,87	0,38	894	**0,93**	1,11	0,15	0,46	0,65	1451	**0,97**	0,93	-0,11	-0,43	0,67	1372	**1,00**	0,96	3,00	-0,05	0,13
**PA5076**	33366	6,85	814,47	16	47,23	0,91	-0,13	-0,54	0,59	1298	**0,99**	1,06	0,09	0,24	0,81	1796	**0,98**	0,92	-0,11	-0,44	0,66	1352	**1,00**	0,97	3,00	-0,05	0,08
**PA5077**	101192	8,91	504,99	12	16,80	1,00	0,00	-0,07	0,95	2062	**1,00**	0,95	-0,08	-0,39	0,70	1555	**0,97**	0,96	-0,06	-0,27	0,79	1621	**1,00**	0,97	3,00	-0,04	0,03
**PA5078**	64883	6,54	1175,44	28	50,98	0,90	-0,15	-0,61	0,54	1199	**0,99**	0,89	-0,17	-0,73	0,47	1052	**0,96**	0,86	-0,22	-0,79	0,43	947	**0,94**	0,88	3,00	-0,18	0,02
**PA5080**	37541	5,80	276,53	9	30,30	0,97	-0,05	-0,24	0,81	1780	**0,99**	1,12	0,17	0,52	0,60	1348	**0,97**	0,92	-0,12	-0,45	0,65	1345	**1,00**	1,00	3,00	0,01	0,10
**PA5105**	28621	6,87	208,14	6	24,27	0,93	-0,10	-0,43	0,67	1473	**0,99**	1,11	0,14	0,44	0,66	1476	**0,97**	0,91	-0,14	-0,53	0,59	1238	**0,99**	0,98	3,00	-0,03	0,11
**PA5110**	39302	5,71	742,51	17	36,85	1,02	0,02	0,00	1,00	2171	**1,00**	1,00	0,00	-0,08	0,93	2035	**0,99**	0,96	-0,07	-0,28	0,78	1605	**1,00**	0,99	3,00	-0,01	0,03
**PA5112**	71866	4,68	493,09	10	17,89	1,64	0,72	2,43	0,02	216	**0,15**	1,72	0,78	2,81	0,00	199	**0,05**	1,79	0,84	2,80	0,01	191	**0,05**	1,72	3,00	0,78	0,08
**PA5113**	51304	6,30	229,20	6	11,75	1,01	0,01	-0,04	0,97	2118	**0,99**	0,90	-0,15	-0,64	0,52	1173	**0,96**	0,83	-0,27	-0,98	0,33	775	**0,87**	0,91	3,00	-0,13	0,09
**PA5117**	72141	5,22	1733,56	37	36,77	1,03	0,04	0,07	0,95	2059	**1,00**	0,98	-0,02	-0,18	0,85	1884	**0,98**	1,00	0,00	-0,06	0,95	1966	**1,00**	1,00	3,00	0,01	0,02
**PA5118**	58261	6,22	348,68	7	16,41	1,08	0,11	0,29	0,77	1690	**0,99**	1,07	0,10	0,27	0,78	1734	**0,98**	0,91	-0,13	-0,51	0,61	1260	**0,99**	1,02	3,00	0,03	0,09
**PA5119**	56995	5,14	1679,86	39	57,90	1,10	0,14	0,39	0,69	1527	**0,99**	1,12	0,16	0,51	0,61	1367	**0,96**	1,09	0,12	0,36	0,72	1475	**1,00**	1,10	3,00	0,14	0,02
**PA5125**	54929	5,59	260,96	7	16,53	0,80	-0,33	-1,24	0,22	608	**0,77**	0,89	-0,17	-0,71	0,47	1071	**0,96**	1,01	0,01	-0,03	0,97	2011	**1,00**	0,90	3,00	-0,16	0,10
**PA5128**	18902	4,46	933,54	18	78,97	0,95	-0,08	-0,37	0,71	1583	**0,98**	0,97	-0,05	-0,28	0,78	1722	**0,98**	0,93	-0,10	-0,41	0,68	1399	**1,00**	0,95	3,00	-0,08	0,02
**PA5130**	16779	9,65	459,05	12	60,73	0,94	-0,09	-0,39	0,70	1540	**0,98**	0,97	-0,04	-0,24	0,81	1797	**0,98**	1,04	0,05	0,12	0,91	1862	**1,00**	0,98	3,00	-0,02	0,05
**PA5131**	58632	5,07	1183,46	25	47,18	0,88	-0,19	-0,76	0,45	1017	**0,96**	0,86	-0,22	-0,90	0,37	861	**0,92**	0,89	-0,16	-0,61	0,54	1161	**0,96**	0,88	3,00	-0,19	0,02
**PA5133**	51390	9,40	77,35	2	5,60	0,93	-0,10	-0,44	0,66	1456	**0,99**	1,18	0,23	0,77	0,44	1010	**0,94**	1,19	0,25	0,78	0,43	958	**0,93**	1,10	3,00	0,14	0,14

**PA5134**	49783	5,52	1081,30	25	47,68	0,92	-0,12	-0,49	0,62	1379	**0,98**	0,93	-0,11	-0,51	0,61	1376	**0,96**	0,97	-0,04	-0,19	0,85	1749	**1,00**	0,94	3,00	-0,09	0,03
**PA5135**	28820	9,63	78,30	2	17,10	1,14	0,19	0,59	0,56	1226	**0,99**	1,23	0,30	1,03	0,30	765	**0,85**	1,11	0,15	0,44	0,66	1360	**1,00**	1,16	3,00	0,21	0,07
**PA5136**	55822	4,88	264,05	6	14,57	1,10	0,13	0,38	0,70	1554	**0,98**	0,96	-0,05	-0,30	0,77	1688	**0,98**	1,11	0,15	0,46	0,64	1323	**1,00**	1,06	3,00	0,08	0,08
**PA5138**	30636	5,17	224,36	5	24,72	1,03	0,05	0,09	0,93	2015	**1,00**	1,06	0,08	0,21	0,83	1837	**0,98**	0,96	-0,05	-0,23	0,82	1676	**1,00**	1,02	3,00	0,03	0,05
**PA5139**	29317	5,72	438,94	10	41,08	0,55	-0,85	-3,08	0,00	146	**0,03**	0,52	-0,95	-3,64	0,00	136	**0,00**	0,44	-1,18	-4,07	0,00	111	**0,00**	0,50	3,00	-0,99	0,06
**PA5140**	29079	5,10	471,80	12	44,98	0,96	-0,06	-0,28	0,78	1725	**0,98**	1,02	0,02	-0,01	0,99	2142	**1,00**	1,03	0,05	0,10	0,92	1884	**1,00**	1,00	3,00	0,01	0,04
**PA5141**	27736	4,95	1040,32	23	68,23	0,93	-0,11	-0,47	0,64	1403	**0,99**	1,02	0,02	-0,01	0,99	2148	**1,00**	1,00	0,00	-0,07	0,94	1942	**1,00**	0,98	3,00	-0,03	0,05
**PA5142**	24509	5,87	245,19	8	37,88	1,12	0,16	0,49	0,63	1382	**0,98**	1,12	0,16	0,50	0,62	1388	**0,96**	0,99	-0,01	-0,08	0,93	1922	**1,00**	1,08	3,00	0,11	0,07
**PA5143**	23456	6,03	332,67	11	46,69	0,97	-0,04	-0,22	0,83	1814	**0,99**	1,00	-0,01	-0,12	0,90	1983	**0,98**	1,06	0,08	0,21	0,83	1702	**1,01**	1,01	3,00	0,01	0,04
**PA5146**	88622	8,07	1099,35	28	41,33	0,93	-0,11	-0,47	0,64	1402	**0,98**	1,01	0,01	-0,05	0,96	2083	**0,99**	0,95	-0,07	-0,29	0,77	1587	**1,00**	0,96	3,00	-0,06	0,04
**PA5147**	41175	8,61	72,97	2	3,00	1,00	0,00	-0,09	0,93	2019	**1,00**	0,98	-0,04	-0,23	0,82	1808	**0,98**	1,04	0,05	0,12	0,91	1863	**1,00**	1,00	3,00	0,00	0,03
**PA5152**	30351	6,67	307,96	9	23,21	2,31	1,21	4,15	0,00	93	**0,00**	2,03	1,02	3,71	0,00	131	**0,00**	2,57	1,36	4,56	0,00	95	**0,00**	2,30	3,00	1,20	0,27
**PA5153**	31815	5,13	466,69	13	42,28	2,32	1,21	4,17	0,00	92	**0,00**	2,27	1,18	4,31	0,00	108	**0,00**	2,88	1,53	5,12	0,00	82	**0,00**	2,49	3,00	1,32	0,34
**PA5161**	41385	5,63	933,26	21	45,97	0,88	-0,18	-0,70	0,48	1087	**0,97**	0,88	-0,19	-0,80	0,42	971	**0,94**	0,86	-0,21	-0,78	0,44	962	**0,93**	0,87	3,00	-0,19	0,01
**PA5162**	34279	5,82	747,77	17	41,11	0,96	-0,06	-0,29	0,77	1693	**0,99**	0,89	-0,16	-0,71	0,48	1079	**0,96**	0,91	-0,14	-0,52	0,60	1256	**0,99**	0,92	3,00	-0,12	0,03
**PA5163**	34500	5,22	648,89	18	46,35	0,87	-0,20	-0,79	0,43	990	**0,95**	0,87	-0,20	-0,84	0,40	929	**0,93**	0,82	-0,29	-1,04	0,30	719	**0,86**	0,85	3,00	-0,23	0,03
**PA5164**	22044	5,74	437,91	12	57,50	0,90	-0,15	-0,62	0,54	1177	**0,99**	0,89	-0,17	-0,75	0,45	1037	**0,95**	0,93	-0,10	-0,41	0,68	1395	**1,00**	0,90	3,00	-0,14	0,02
**PA5166**	52753	5,49	312,04	11	21,17	0,87	-0,20	-0,80	0,43	981	**0,94**	0,92	-0,12	-0,53	0,60	1328	**0,97**	0,90	-0,16	-0,59	0,56	1190	**0,97**	0,90	3,00	-0,16	0,03
**PA5167**	41829	8,37	1201,33	31	62,27	5,37	2,43	8,43	0,00	3	**0,00**	4,62	2,21	8,13	0,00	26	**0,00**	6,27	2,65	8,92	0,00	12	**0,00**	5,42	3,00	2,44	0,83
**PA5168**	23951	5,49	240,27	4	17,15	3,00	1,59	5,49	0,00	66	**0,00**	4,00	2,00	7,36	0,00	38	**0,00**	5,01	2,33	7,82	0,00	40	**0,00**	4,01	3,00	2,00	1,01
**PA5170**	55059	6,53	86,42	2	7,70	1,00	0,00	-0,08	0,93	2032	**1,00**	0,97	-0,05	-0,28	0,78	1726	**0,98**	1,16	0,21	0,66	0,51	1106	**0,95**	1,04	3,00	0,06	0,10
**PA5171**	49611	5,52	2249,52	47	61,55	1,12	0,17	0,51	0,61	1335	**0,99**	1,01	0,01	-0,05	0,96	2084	**1,00**	1,11	0,15	0,46	0,65	1334	**1,00**	1,08	3,00	0,11	0,06
**PA5172**	41681	6,13	846,38	26	50,63	1,02	0,03	0,02	0,99	2159	**0,99**	1,10	0,14	0,41	0,68	1527	**0,97**	1,14	0,19	0,59	0,56	1191	**0,96**	1,09	3,00	0,12	0,06
**PA5173**	35889	5,25	618,21	12	35,30	1,10	0,14	0,42	0,67	1481	**0,98**	1,02	0,03	0,03	0,98	2123	**1,00**	1,12	0,16	0,48	0,63	1303	**1,00**	1,08	3,00	0,11	0,05
**PA5174**	72698	5,75	2348,17	44	54,62	1,04	0,06	0,13	0,90	1968	**0,99**	0,96	-0,06	-0,32	0,75	1655	**0,98**	0,99	-0,02	-0,11	0,91	1868	**1,00**	1,00	3,00	0,00	0,04
**PA5175**	30666	5,04	286,45	7	25,31	1,02	0,03	0,02	0,98	2145	**0,99**	0,90	-0,15	-0,67	0,50	1125	**0,96**	0,94	-0,09	-0,38	0,71	1455	**1,00**	0,95	3,00	-0,07	0,06
**PA5176**	21880	4,81	348,27	9	44,80	0,91	-0,14	-0,55	0,58	1282	**0,98**	0,89	-0,17	-0,73	0,47	1053	**0,96**	1,02	0,03	0,06	0,96	1970	**1,00**	0,94	3,00	-0,09	0,07
**PA5177**	26380	5,63	153,78	4	12,90	1,02	0,03	0,02	0,99	2157	**0,99**	1,17	0,23	0,75	0,45	1032	**0,95**	1,11	0,15	0,45	0,65	1349	**1,00**	1,10	3,00	0,14	0,08
**PA5178**	17613	5,45	646,58	13	58,80	0,72	-0,47	-1,72	0,08	366	**0,50**	0,74	-0,44	-1,74	0,08	404	**0,44**	0,75	-0,42	-1,47	0,14	482	**0,61**	0,74	3,00	-0,44	0,01
**PA5184**	21073	8,76	242,24	7	43,87	0,97	-0,04	-0,22	0,83	1812	**0,99**	0,95	-0,08	-0,38	0,70	1564	**0,97**	0,88	-0,19	-0,69	0,49	1063	**0,95**	0,93	3,00	-0,10	0,05
**PA5190**	23582	5,74	194,74	5	38,80	0,90	-0,15	-0,62	0,53	1172	**0,99**	1,19	0,26	0,86	0,39	914	**0,93**	0,77	-0,38	-1,36	0,18	538	**0,67**	0,95	3,00	-0,07	0,22
**PA5192**	60100	5,27	2240,09	47	54,54	0,83	-0,28	-1,05	0,29	739	**0,86**	0,80	-0,31	-1,26	0,21	600	**0,74**	0,80	-0,31	-1,12	0,26	651	**0,83**	0,81	3,00	-0,30	0,01
**PA5193**	33902	4,73	326,32	10	27,10	0,82	-0,29	-1,11	0,27	688	**0,84**	0,88	-0,19	-0,79	0,43	991	**0,94**	0,82	-0,29	-1,05	0,30	713	**0,85**	0,84	3,00	-0,26	0,04
**PA5198**	34074	6,30	155,23	4	17,55	0,91	-0,14	-0,58	0,56	1237	**0,98**	0,90	-0,15	-0,65	0,51	1150	**0,96**	1,01	0,02	0,01	1,00	2054	**1,00**	0,94	3,00	-0,09	0,06
**PA5199**	50143	5,92	138,67	4	11,30	1,10	0,14	0,40	0,69	1518	**0,98**	1,20	0,26	0,86	0,39	906	**0,93**	1,29	0,37	1,18	0,24	622	**0,78**	1,19	3,00	0,26	0,09
**PA5200**	29538	6,23	468,93	13	36,98	0,97	-0,05	-0,25	0,80	1763	**0,99**	1,02	0,03	0,03	0,97	2114	**1,00**	1,03	0,04	0,08	0,93	1925	**1,00**	1,01	3,00	0,01	0,03
**PA5201**	92831	6,01	2356,31	55	55,31	0,84	-0,24	-0,94	0,35	828	**0,91**	0,82	-0,28	-1,14	0,25	668	**0,82**	0,84	-0,25	-0,91	0,36	827	**0,90**	0,84	3,00	-0,26	0,01
**PA5202**	14519	6,90	34,46	2	13,20	1,08	0,11	0,31	0,75	1655	**0,99**	0,85	-0,24	-0,99	0,32	802	**0,87**	0,98	-0,04	-0,18	0,86	1769	**1,00**	0,97	3,00	-0,05	0,12
**PA5203**	61410	5,11	393,23	12	22,30	0,98	-0,03	-0,20	0,85	1862	**0,99**	0,89	-0,17	-0,74	0,46	1040	**0,95**	0,95	-0,07	-0,31	0,76	1561	**1,00**	0,94	3,00	-0,09	0,05
**PA5204**	49068	6,05	335,45	9	21,66	0,65	-0,62	-2,24	0,03	247	**0,22**	0,63	-0,66	-2,56	0,01	224	**0,10**	0,57	-0,80	-2,76	0,01	196	**0,06**	0,62	3,00	-0,69	0,04
**PA5209**	55986	8,90	605,75	16	32,60	0,93	-0,10	-0,44	0,66	1448	**0,99**	0,90	-0,15	-0,65	0,52	1163	**0,96**	1,01	0,01	-0,03	0,97	2012	**1,00**	0,95	3,00	-0,08	0,05
**PA5210**	69242	6,11	208,32	6	8,43	0,59	-0,75	-2,72	0,01	176	**0,08**	0,56	-0,83	-3,18	0,00	167	**0,02**	0,49	-1,02	-3,50	0,00	133	**0,01**	0,55	3,00	-0,86	0,05
**PA5214**	14498	4,09	181,24	4	21,30	1,08	0,11	0,32	0,75	1643	**0,99**	1,18	0,24	0,80	0,42	973	**0,94**	0,99	-0,02	-0,12	0,91	1860	**1,00**	1,08	3,00	0,12	0,10
**PA5215**	40514	5,43	401,91	9	25,77	0,90	-0,15	-0,62	0,54	1178	**0,99**	0,93	-0,11	-0,50	0,62	1391	**0,96**	0,93	-0,11	-0,43	0,67	1379	**1,00**	0,92	3,00	-0,12	0,02
**PA5217**	39100	6,02	444,22	14	33,64	1,76	0,82	2,78	0,01	169	**0,07**	1,77	0,82	2,96	0,00	182	**0,04**	1,81	0,85	2,83	0,00	185	**0,05**	1,78	3,00	0,83	0,02
**PA5221**	45373	6,23	274,65	7	17,94	1,13	0,18	0,56	0,58	1276	**0,98**	1,09	0,13	0,37	0,71	1580	**0,97**	0,98	-0,03	-0,17	0,86	1778	**1,00**	1,07	3,00	0,09	0,08
**PA5223**	44033	6,38	271,58	7	13,29	0,90	-0,16	-0,64	0,52	1151	**0,99**	0,95	-0,07	-0,35	0,73	1615	**0,97**	1,08	0,11	0,33	0,74	1531	**1,00**	0,98	3,00	-0,03	0,10
**PA5224**	52116	5,25	711,76	17	38,74	0,90	-0,15	-0,59	0,55	1220	**0,98**	1,03	0,04	0,05	0,96	2094	**0,99**	0,90	-0,15	-0,57	0,57	1208	**0,97**	0,94	3,00	-0,08	0,07

**PA5225**	20060	4,30	202,24	4	27,58	0,92	-0,12	-0,50	0,62	1361	**0,98**	0,95	-0,08	-0,39	0,70	1556	**0,97**	1,08	0,11	0,33	0,74	1536	**1,00**	0,98	3,00	-0,02	0,09
**PA5227**	12460	5,37	116,48	3	31,70	0,90	-0,15	-0,62	0,54	1174	**0,99**	1,37	0,46	1,60	0,11	454	**0,52**	0,81	-0,30	-1,06	0,29	701	**0,85**	1,03	3,00	0,04	0,30
**PA5229**	17915	6,73	313,71	7	29,70	0,79	-0,33	-1,24	0,21	603	**0,77**	0,91	-0,13	-0,59	0,56	1231	**0,97**	0,92	-0,13	-0,48	0,63	1299	**1,00**	0,87	3,00	-0,19	0,07
**PA5231**	103632	6,18	154,31	5	6,08	1,00	0,00	-0,08	0,94	2034	**1,00**	0,90	-0,15	-0,65	0,52	1166	**0,96**	1,16	0,22	0,68	0,49	1079	**0,94**	1,02	3,00	0,03	0,13
**PA5232**	41468	9,11	877,90	19	46,35	0,83	-0,27	-1,02	0,31	758	**0,88**	0,82	-0,29	-1,16	0,24	647	**0,82**	0,84	-0,25	-0,90	0,37	830	**0,91**	0,83	3,00	-0,27	0,01
**PA5234**	36485	6,46	137,71	3	15,70	0,90	-0,16	-0,63	0,53	1161	**0,99**	0,85	-0,23	-0,97	0,33	811	**0,88**	0,90	-0,16	-0,58	0,56	1196	**0,96**	0,88	3,00	-0,18	0,03
**PA5235**	51901	8,95	112,04	2	5,60	0,92	-0,13	-0,53	0,60	1313	**0,99**	0,71	-0,49	-1,93	0,05	350	**0,33**	0,74	-0,43	-1,51	0,13	456	**0,59**	0,79	3,00	-0,34	0,11
**PA5236**	36526	5,55	138,79	3	14,07	0,87	-0,20	-0,78	0,43	996	**0,95**	0,98	-0,03	-0,20	0,84	1850	**0,98**	0,96	-0,06	-0,25	0,80	1651	**1,00**	0,94	3,00	-0,09	0,06
**PA5237**	58753	6,15	353,84	10	15,90	1,04	0,05	0,10	0,92	2003	**1,00**	0,81	-0,31	-1,24	0,21	612	**0,76**	0,94	-0,10	-0,38	0,70	1445	**1,00**	0,93	3,00	-0,11	0,11
**PA5239**	51887	6,40	2195,43	46	72,80	0,91	-0,13	-0,54	0,59	1290	**0,99**	0,86	-0,22	-0,93	0,35	844	**0,91**	0,86	-0,21	-0,78	0,44	961	**0,93**	0,88	3,00	-0,19	0,03
**PA5240**	13441	4,70	765,46	14	62,00	0,83	-0,27	-1,03	0,30	751	**0,88**	0,86	-0,21	-0,88	0,38	879	**0,93**	0,92	-0,12	-0,46	0,64	1326	**1,00**	0,87	3,00	-0,20	0,05
**PA5241**	59306	6,25	526,78	16	30,13	0,87	-0,21	-0,81	0,42	973	**0,94**	0,92	-0,12	-0,55	0,58	1300	**0,96**	0,85	-0,23	-0,84	0,40	890	**0,92**	0,88	3,00	-0,19	0,04
**PA5242**	88563	7,32	1405,09	32	36,16	1,14	0,19	0,59	0,56	1233	**0,98**	1,17	0,23	0,74	0,46	1038	**0,95**	1,23	0,30	0,96	0,34	788	**0,88**	1,18	3,00	0,24	0,05
**PA5243**	38423	5,06	634,61	12	36,50	0,79	-0,35	-1,30	0,19	568	**0,75**	0,94	-0,08	-0,41	0,69	1530	**0,97**	0,99	-0,01	-0,09	0,93	1916	**1,00**	0,91	3,00	-0,14	0,11
**PA5245**	25370	4,95	847,81	18	70,95	0,90	-0,16	-0,63	0,53	1160	**0,99**	1,06	0,08	0,21	0,83	1836	**0,98**	1,04	0,05	0,12	0,91	1861	**1,00**	1,00	3,00	0,00	0,09
**PA5252**	75099	5,70	939,54	25	34,11	0,98	-0,02	-0,16	0,87	1923	**0,98**	1,00	-0,01	-0,12	0,91	1990	**0,98**	0,98	-0,04	-0,18	0,86	1773	**1,00**	0,99	3,00	-0,02	0,01
**PA5253**	44026	10,60	323,52	7	10,63	0,69	-0,53	-1,93	0,05	294	**0,40**	0,82	-0,29	-1,19	0,23	639	**0,79**	0,83	-0,26	-0,95	0,34	802	**0,88**	0,78	3,00	-0,36	0,08
**PA5255**	18852	4,83	69,37	2	15,00	0,98	-0,02	-0,17	0,87	1912	**0,99**	0,76	-0,39	-1,54	0,12	484	**0,55**	1,00	0,00	-0,05	0,96	1983	**1,00**	0,92	3,00	-0,13	0,13
**PA5257**	48050	8,94	1005,17	23	54,09	1,01	0,01	-0,05	0,96	2100	**1,00**	1,01	0,02	-0,04	0,97	2107	**0,99**	1,00	-0,01	-0,08	0,94	1935	**1,00**	1,00	3,00	0,01	0,01
**PA5258**	42320	4,99	816,19	17	47,04	1,02	0,02	0,00	1,00	2172	**1,00**	0,99	-0,01	-0,14	0,89	1961	**0,98**	0,89	-0,17	-0,62	0,54	1154	**0,96**	0,97	3,00	-0,05	0,07
**PA5260**	35101	5,39	522,06	14	39,73	1,14	0,19	0,59	0,56	1227	**0,98**	1,01	0,02	-0,02	0,98	2132	**1,00**	0,96	-0,06	-0,26	0,79	1630	**1,00**	1,04	3,00	0,05	0,09
**PA5261**	29124	6,73	212,09	6	26,40	0,79	-0,34	-1,27	0,20	581	**0,76**	0,75	-0,41	-1,61	0,11	449	**0,52**	0,84	-0,25	-0,90	0,37	838	**0,91**	0,80	3,00	-0,33	0,04
**PA5263**	54288	5,50	731,85	20	31,77	0,92	-0,12	-0,50	0,62	1363	**0,99**	0,94	-0,09	-0,42	0,67	1507	**0,97**	0,88	-0,18	-0,66	0,51	1100	**0,95**	0,92	3,00	-0,13	0,03
**PA5274**	15163	4,46	553,12	8	69,40	0,94	-0,09	-0,40	0,69	1517	**0,98**	0,87	-0,21	-0,86	0,39	902	**0,93**	1,05	0,07	0,18	0,86	1762	**1,00**	0,95	3,00	-0,07	0,09
**PA5275**	12542	4,19	203,10	4	53,18	1,03	0,04	0,06	0,95	2075	**1,00**	1,10	0,14	0,43	0,67	1494	**0,97**	1,25	0,32	1,02	0,31	740	**0,85**	1,13	3,00	0,17	0,11
**PA5277**	47232	5,41	1160,79	24	52,04	1,02	0,02	0,00	1,00	2173	**1,00**	1,05	0,07	0,17	0,87	1916	**0,98**	1,00	0,00	-0,05	0,96	1981	**1,00**	1,02	3,00	0,03	0,02
**PA5278**	31926	7,16	255,97	7	21,44	1,09	0,12	0,36	0,72	1593	**0,99**	1,11	0,15	0,45	0,66	1466	**0,97**	1,00	-0,01	-0,08	0,94	1931	**1,00**	1,06	3,00	0,09	0,06
**PA5279**	26518	5,36	179,35	4	15,98	0,91	-0,14	-0,58	0,56	1244	**0,98**	0,91	-0,14	-0,60	0,55	1218	**0,97**	0,93	-0,10	-0,41	0,68	1396	**1,00**	0,92	3,00	-0,13	0,01
**PA5288**	13864	5,41	582,03	13	75,42	0,61	-0,71	-2,56	0,01	193	**0,12**	0,67	-0,57	-2,24	0,03	280	**0,20**	0,61	-0,71	-2,48	0,01	228	**0,12**	0,63	3,00	-0,66	0,03
**PA5289**	10392	5,17	223,10	7	69,97	0,90	-0,15	-0,61	0,54	1200	**0,98**	0,92	-0,12	-0,55	0,58	1308	**0,96**	0,89	-0,16	-0,60	0,55	1171	**0,96**	0,90	3,00	-0,14	0,01
**PA5291**	75131	6,25	137,83	5	3,90	0,69	-0,53	-1,94	0,05	293	**0,39**	0,66	-0,61	-2,36	0,02	257	**0,15**	0,57	-0,82	-2,82	0,00	186	**0,05**	0,64	3,00	-0,65	0,06
**PA5296**	82745	5,78	405,25	10	13,60	1,03	0,05	0,08	0,94	2039	**1,00**	1,20	0,26	0,87	0,38	891	**0,93**	1,03	0,05	0,10	0,92	1885	**1,00**	1,09	3,00	0,12	0,10
**PA5298**	22435	6,17	596,09	11	58,96	1,15	0,20	0,61	0,54	1189	**0,99**	1,24	0,31	1,06	0,29	747	**0,84**	1,16	0,21	0,65	0,52	1115	**0,95**	1,18	3,00	0,24	0,05
**PA5300**	15599	6,70	473,03	10	41,20	1,01	0,02	-0,02	0,98	2141	**1,00**	1,03	0,04	0,06	0,95	2069	**0,99**	1,23	0,29	0,94	0,35	807	**0,89**	1,09	3,00	0,12	0,12
**PA5301**	21373	5,42	220,68	6	31,80	0,97	-0,05	-0,26	0,80	1751	**0,99**	1,02	0,02	-0,01	0,99	2151	**1,00**	1,09	0,13	0,37	0,71	1468	**1,00**	1,02	3,00	0,03	0,06
**PA5302**	40463	6,38	529,82	15	38,63	2,66	1,41	4,87	0,00	72	**0,00**	2,46	1,30	4,75	0,00	91	**0,00**	2,85	1,51	5,06	0,00	83	**0,00**	2,66	3,00	1,41	0,19
**PA5304**	49744	6,97	1371,81	36	66,13	2,35	1,23	4,25	0,00	89	**0,00**	2,54	1,35	4,92	0,00	85	**0,00**	2,59	1,37	4,59	0,00	94	**0,00**	2,49	3,00	1,32	0,12
**PA5305**	12817	9,57	153,55	4	55,30	0,80	-0,33	-1,23	0,22	613	**0,78**	0,90	-0,15	-0,66	0,51	1137	**0,96**	1,02	0,03	0,03	0,98	2019	**1,00**	0,90	3,00	-0,14	0,11
**PA5308**	20533	7,83	80,72	3	10,07	0,92	-0,11	-0,48	0,63	1393	**0,98**	0,90	-0,16	-0,68	0,49	1118	**0,96**	0,77	-0,38	-1,34	0,18	543	**0,68**	0,86	3,00	-0,21	0,08
**PA5309**	49867	6,90	303,67	8	15,81	1,11	0,15	0,43	0,67	1469	**0,99**	1,11	0,15	0,47	0,64	1432	**0,96**	1,10	0,13	0,39	0,70	1430	**1,00**	1,10	3,00	0,14	0,01
**PA5312**	58050	5,40	2071,16	38	59,62	1,16	0,22	0,69	0,49	1096	**0,97**	1,19	0,25	0,84	0,40	930	**0,93**	1,10	0,14	0,42	0,68	1390	**1,00**	1,15	3,00	0,20	0,05
**PA5313**	50276	6,21	625,61	15	37,75	0,88	-0,18	-0,71	0,48	1070	**0,97**	0,82	-0,29	-1,16	0,24	648	**0,81**	0,77	-0,37	-1,31	0,19	554	**0,71**	0,83	3,00	-0,28	0,05
**PA5315**	7482	9,99	130,01	2	29,40	0,48	-1,05	-3,77	0,00	104	**0,00**	0,98	-0,03	-0,21	0,83	1842	**0,98**	0,95	-0,07	-0,30	0,76	1577	**1,00**	0,80	3,00	-0,31	0,28
**PA5316**	10115	11,72	217,48	6	64,53	0,86	-0,22	-0,87	0,38	905	**0,92**	0,94	-0,08	-0,41	0,69	1533	**0,97**	0,95	-0,08	-0,31	0,75	1558	**1,00**	0,92	3,00	-0,13	0,05
**PA5317**	61631	6,47	261,75	7	11,97	0,80	-0,32	-1,22	0,22	620	**0,79**	0,76	-0,39	-1,54	0,12	482	**0,55**	0,86	-0,22	-0,80	0,42	933	**0,93**	0,81	3,00	-0,31	0,05
**PA5320**	45020	5,88	691,24	13	39,77	0,96	-0,06	-0,29	0,77	1702	**0,99**	0,95	-0,07	-0,36	0,72	1597	**0,97**	0,95	-0,07	-0,30	0,76	1575	**1,00**	0,95	3,00	-0,07	0,00
**PA5321**	16533	5,35	329,25	5	35,10	1,54	0,62	2,10	0,04	266	**0,29**	0,96	-0,06	-0,34	0,74	1633	**0,97**	0,97	-0,04	-0,20	0,84	1723	**1,00**	1,15	3,00	0,21	0,33
**PA5322**	54188	5,17	1331,10	30	47,65	0,95	-0,08	-0,35	0,73	1607	**0,99**	0,95	-0,07	-0,37	0,71	1584	**0,97**	0,93	-0,11	-0,43	0,67	1370	**1,00**	0,94	3,00	-0,09	0,01

**PA5323**	34613	6,32	967,34	19	51,78	0,99	-0,01	-0,12	0,90	1978	**0,99**	0,98	-0,03	-0,22	0,83	1823	**0,98**	0,96	-0,07	-0,28	0,78	1606	**1,00**	0,98	3,00	-0,04	0,02
**PA5330**	23620	9,09	429,40	9	43,11	0,75	-0,42	-1,56	0,12	433	**0,59**	0,74	-0,43	-1,69	0,09	420	**0,46**	0,72	-0,48	-1,68	0,09	383	**0,50**	0,74	3,00	-0,44	0,02
**PA5331**	24505	5,31	647,71	11	53,56	0,39	-1,36	-4,84	0,00	73	**0,00**	0,42	-1,23	-4,70	0,00	93	**0,00**	0,49	-1,03	-3,55	0,00	129	**0,01**	0,44	3,00	-1,20	0,05
**PA5332**	31451	5,33	328,33	8	22,30	0,18	-2,51	-8,90	0,00	20	**0,00**	0,13	-2,90	-10,91	0,00	19	**0,00**	0,14	-2,88	-9,82	0,00	31	**0,00**	0,15	3,00	-2,75	0,02
**PA5334**	27171	5,84	455,55	9	39,08	0,90	-0,15	-0,61	0,54	1201	**0,98**	0,90	-0,15	-0,64	0,52	1171	**0,96**	0,95	-0,08	-0,32	0,75	1542	**1,00**	0,92	3,00	-0,12	0,03
**PA5335**	33320	5,72	491,63	15	46,51	0,97	-0,04	-0,22	0,82	1803	**0,99**	0,99	-0,02	-0,17	0,87	1914	**0,98**	0,97	-0,05	-0,23	0,82	1680	**1,01**	0,97	3,00	-0,04	0,01
**PA5336**	23808	5,40	465,94	12	30,50	1,00	0,00	-0,06	0,95	2066	**1,00**	0,98	-0,03	-0,20	0,84	1851	**0,98**	0,89	-0,17	-0,62	0,53	1145	**0,96**	0,96	3,00	-0,06	0,06
**PA5337**	10535	4,20	121,54	3	37,50	0,83	-0,27	-1,03	0,30	752	**0,88**	0,85	-0,23	-0,94	0,35	836	**0,90**	0,82	-0,28	-1,00	0,32	756	**0,86**	0,84	3,00	-0,26	0,02
**PA5338**	84800	9,11	464,56	12	15,95	1,14	0,19	0,59	0,56	1228	**0,98**	1,15	0,20	0,66	0,51	1132	**0,97**	1,18	0,24	0,76	0,44	980	**0,93**	1,16	3,00	0,21	0,02
**PA5339**	14754	5,10	1068,44	22	84,97	0,90	-0,16	-0,63	0,53	1164	**0,99**	1,22	0,28	0,95	0,34	826	**0,89**	1,02	0,03	0,03	0,97	2007	**1,00**	1,04	3,00	0,06	0,16
**PA5340**	27991	4,69	286,41	7	28,20	1,18	0,24	0,76	0,44	1010	**0,96**	0,97	-0,04	-0,24	0,81	1795	**0,98**	0,75	-0,42	-1,48	0,14	478	**0,60**	0,97	3,00	-0,05	0,22
**PA5343**	31232	5,81	356,74	8	26,21	0,92	-0,13	-0,53	0,60	1317	**0,99**	0,79	-0,34	-1,36	0,17	542	**0,69**	0,82	-0,29	-1,02	0,31	737	**0,86**	0,84	3,00	-0,25	0,07
**PA5344**	36331	6,27	441,44	12	39,88	0,90	-0,15	-0,62	0,54	1182	**0,99**	0,82	-0,28	-1,14	0,25	676	**0,81**	0,94	-0,09	-0,36	0,72	1473	**1,00**	0,89	3,00	-0,17	0,06
**PA5345**	79145	6,83	143,61	4	7,75	0,99	-0,01	-0,13	0,90	1969	**0,99**	1,07	0,09	0,24	0,81	1787	**0,98**	0,92	-0,12	-0,46	0,64	1321	**1,00**	0,99	3,00	-0,01	0,07
**PA5346**	53643	5,76	297,49	7	20,30	1,02	0,03	0,04	0,97	2122	**0,99**	1,08	0,11	0,32	0,75	1660	**0,98**	1,00	0,00	-0,04	0,97	1985	**1,00**	1,04	3,00	0,05	0,04
**PA5348**	11130	10,49	448,02	13	71,30	4,05	2,02	7,00	0,00	35	**0,00**	3,39	1,76	6,46	0,00	53	**0,00**	5,31	2,41	8,11	0,00	34	**0,00**	4,25	3,00	2,09	0,98
**PA5349**	42787	5,50	454,45	11	26,53	0,75	-0,42	-1,56	0,12	437	**0,60**	0,76	-0,39	-1,56	0,12	471	**0,55**	0,68	-0,56	-1,94	0,05	313	**0,34**	0,73	3,00	-0,45	0,04
**PA5357**	19736	4,96	133,13	3	24,50	0,88	-0,19	-0,75	0,46	1027	**0,96**	1,04	0,06	0,13	0,90	1982	**0,98**	0,93	-0,10	-0,40	0,69	1413	**1,00**	0,95	3,00	-0,07	0,08
**PA5360**	27327	5,27	325,29	6	24,43	1,07	0,10	0,28	0,78	1726	**0,98**	1,11	0,15	0,47	0,64	1436	**0,96**	1,25	0,32	1,04	0,30	722	**0,86**	1,14	3,00	0,20	0,09
**PA5362**	53314	5,58	60,64	3	5,73	0,98	-0,03	-0,20	0,85	1863	**0,99**	0,96	-0,06	-0,33	0,74	1645	**0,98**	0,88	-0,18	-0,67	0,50	1096	**0,95**	0,94	3,00	-0,09	0,05
**PA5364**	35545	9,05	137,60	2	11,85	1,31	0,39	1,29	0,20	570	**0,75**	0,68	-0,56	-2,19	0,03	289	**0,21**	1,02	0,03	0,05	0,96	1978	**1,00**	1,00	3,00	0,01	0,32
**PA5365**	28859	5,21	390,95	10	49,31	1,06	0,08	0,21	0,84	1837	**0,99**	1,11	0,15	0,46	0,65	1444	**0,97**	1,11	0,15	0,46	0,65	1328	**1,00**	1,09	3,00	0,13	0,03
**PA5366**	33659	5,91	291,37	9	30,29	1,21	0,27	0,87	0,38	902	**0,93**	1,20	0,27	0,90	0,37	865	**0,92**	1,14	0,18	0,57	0,57	1210	**0,97**	1,18	3,00	0,24	0,04
**PA5367**	65033	7,90	306,04	8	10,93	1,55	0,63	2,15	0,03	258	**0,27**	1,27	0,35	1,21	0,23	627	**0,79**	1,41	0,50	1,63	0,10	400	**0,53**	1,41	3,00	0,50	0,14
**PA5368**	76971	6,01	344,08	7	7,41	1,20	0,26	0,82	0,41	953	**0,94**	1,18	0,24	0,81	0,42	960	**0,94**	1,09	0,12	0,35	0,72	1488	**1,00**	1,16	3,00	0,21	0,06
**PA5369**	38723	9,13	232,54	8	25,40	1,30	0,38	1,24	0,22	605	**0,77**	1,24	0,32	1,08	0,28	729	**0,83**	1,10	0,13	0,39	0,70	1431	**1,00**	1,21	3,00	0,28	0,10
**PA5373**	56221	5,25	360,94	10	19,68	0,61	-0,70	-2,55	0,01	196	**0,12**	0,75	-0,42	-1,65	0,10	436	**0,49**	0,65	-0,62	-2,17	0,03	276	**0,23**	0,67	3,00	-0,58	0,07
**PA5376**	47232	7,11	54,63	2	8,70	0,72	-0,47	-1,72	0,08	367	**0,50**	0,74	-0,43	-1,72	0,09	413	**0,45**	0,80	-0,33	-1,17	0,24	629	**0,79**	0,75	3,00	-0,41	0,04
**PA5378**	38048	6,77	115,87	4	12,65	0,83	-0,27	-1,03	0,30	756	**0,88**	0,65	-0,63	-2,44	0,01	244	**0,13**	0,90	-0,15	-0,55	0,58	1223	**0,98**	0,79	3,00	-0,33	0,13
**PA5406**	12838	4,31	200,03	5	33,66	0,76	-0,39	-1,45	0,15	485	**0,66**	0,64	-0,64	-2,49	0,01	237	**0,12**	1,02	0,02	0,02	0,99	2042	**1,00**	0,81	3,00	-0,31	0,19
**PA5413**	40326	5,18	750,04	16	39,41	1,12	0,17	0,51	0,61	1341	**0,99**	1,22	0,28	0,96	0,34	819	**0,89**	1,09	0,12	0,35	0,73	1499	**1,00**	1,14	3,00	0,19	0,07
**PA5414**	24400	5,76	331,64	8	50,70	0,97	-0,04	-0,23	0,82	1801	**0,99**	1,06	0,09	0,22	0,82	1815	**0,98**	1,16	0,21	0,66	0,51	1103	**0,95**	1,06	3,00	0,09	0,09
**PA5422**	37421	5,28	181,93	5	18,04	0,89	-0,17	-0,68	0,49	1099	**0,98**	0,93	-0,11	-0,51	0,61	1357	**0,97**	0,95	-0,07	-0,29	0,77	1596	**1,00**	0,92	3,00	-0,12	0,03
**PA5423**	21765	5,64	122,71	4	22,93	1,59	0,67	2,27	0,02	241	**0,21**	1,23	0,30	1,02	0,31	780	**0,86**	1,20	0,26	0,82	0,41	920	**0,92**	1,34	3,00	0,42	0,22
**PA5425**	40575	5,37	297,65	8	26,90	0,98	-0,04	-0,21	0,84	1841	**0,99**	0,90	-0,15	-0,64	0,52	1167	**0,96**	0,89	-0,17	-0,65	0,52	1120	**0,95**	0,92	3,00	-0,12	0,05
**PA5426**	18078	5,80	697,76	14	59,86	0,92	-0,13	-0,53	0,60	1318	**0,99**	0,94	-0,09	-0,44	0,66	1474	**0,96**	0,97	-0,05	-0,22	0,83	1697	**1,00**	0,94	3,00	-0,09	0,03
**PA5427**	38228	5,57	429,54	9	35,10	0,72	-0,46	-1,71	0,09	372	**0,51**	0,92	-0,12	-0,56	0,58	1297	**0,96**	1,12	0,16	0,48	0,63	1296	**1,00**	0,92	3,00	-0,12	0,20
**PA5428**	34473	6,68	133,33	4	18,45	0,90	-0,15	-0,60	0,55	1206	**0,98**	1,27	0,35	1,19	0,23	636	**0,79**	0,81	-0,30	-1,07	0,28	689	**0,85**	1,00	3,00	-0,01	0,24
**PA5429**	53815	5,55	2023,50	39	61,84	1,54	0,62	2,10	0,04	264	**0,29**	1,51	0,59	2,12	0,03	303	**0,25**	1,50	0,59	1,94	0,05	314	**0,34**	1,52	3,00	0,60	0,02
**PA5435**	71282	5,59	1460,45	30	35,97	1,84	0,88	3,00	0,00	155	**0,04**	2,15	1,11	4,02	0,00	116	**0,00**	2,22	1,15	3,85	0,00	118	**0,00**	2,07	3,00	1,05	0,21
**PA5436**	54745	6,19	832,71	21	33,57	1,76	0,81	2,78	0,01	170	**0,07**	1,85	0,88	3,20	0,00	164	**0,02**	1,90	0,93	3,08	0,00	162	**0,03**	1,84	3,00	0,88	0,07
**PA5438**	33686	6,73	158,37	6	25,30	0,96	-0,07	-0,31	0,76	1665	**0,99**	0,87	-0,21	-0,87	0,38	896	**0,93**	0,92	-0,11	-0,44	0,66	1353	**1,00**	0,92	3,00	-0,13	0,05
**PA5441**	85399	4,90	1533,69	34	46,76	1,02	0,02	0,00	1,00	2168	**1,00**	0,95	-0,07	-0,36	0,72	1594	**0,97**	0,99	-0,01	-0,10	0,92	1887	**1,00**	0,99	3,00	-0,02	0,03
**PA5443**	84804	5,60	1212,84	27	34,12	0,91	-0,13	-0,53	0,59	1303	**0,99**	0,96	-0,05	-0,30	0,77	1685	**0,98**	0,90	-0,15	-0,58	0,56	1206	**0,96**	0,93	3,00	-0,11	0,03
**PA5445**	56519	5,87	1129,45	25	35,94	0,51	-0,98	-3,51	0,00	115	**0,01**	0,34	-1,57	-5,96	0,00	65	**0,00**	0,41	-1,30	-4,47	0,00	97	**0,00**	0,42	3,00	-1,26	0,09
**PA5446**	8308	11,71	63,99	2	26,10	1,60	0,68	2,29	0,02	235	**0,20**	1,97	0,98	3,54	0,00	140	**0,01**	2,89	1,53	5,12	0,00	81	**0,00**	2,15	3,00	1,10	0,66
**PA5456**	51149	5,91	73,51	2	4,00	1,37	0,46	1,53	0,13	455	**0,61**	1,85	0,89	3,21	0,00	161	**0,02**	1,16	0,22	0,68	0,50	1088	**0,95**	1,46	3,00	0,55	0,35
**PA5459**	32307	4,90	170,91	5	19,98	1,66	0,73	2,48	0,01	208	**0,14**	1,84	0,88	3,19	0,00	165	**0,02**	1,72	0,78	2,59	0,01	215	**0,09**	1,74	3,00	0,80	0,09

**PA5472**	30677	5,46	383,10	8	41,45	0,71	-0,49	-1,82	0,07	334	**0,45**	0,64	-0,64	-2,50	0,01	233	**0,12**	0,63	-0,67	-2,31	0,02	257	**0,17**	0,66	3,00	-0,60	0,04
**PA5479**	50616	9,17	283,51	8	14,06	0,82	-0,28	-1,06	0,29	722	**0,87**	0,97	-0,05	-0,29	0,78	1709	**0,98**	0,85	-0,23	-0,85	0,40	887	**0,92**	0,88	3,00	-0,18	0,08
**PA5483**	51684	5,35	387,32	11	23,63	1,00	0,00	-0,08	0,94	2043	**1,00**	0,99	-0,01	-0,15	0,88	1949	**0,98**	0,95	-0,08	-0,33	0,74	1529	**1,00**	0,98	3,00	-0,03	0,03
**PA5485**	30062	6,71	110,26	3	15,20	0,80	-0,33	-1,23	0,22	614	**0,78**	0,95	-0,07	-0,35	0,73	1623	**0,97**	0,97	-0,05	-0,22	0,82	1688	**1,01**	0,91	3,00	-0,14	0,09
**PA5487**	76830	4,89	41,88	2	7,50	0,92	-0,12	-0,51	0,61	1343	**0,99**	1,15	0,20	0,66	0,51	1145	**0,97**	1,06	0,08	0,21	0,84	1718	**1,00**	1,04	3,00	0,06	0,12
**PA5489**	26190	5,98	889,99	19	41,24	1,02	0,03	0,03	0,97	2131	**0,99**	0,98	-0,03	-0,22	0,83	1824	**0,98**	1,00	0,01	-0,04	0,97	1995	**1,00**	1,00	3,00	0,00	0,02
**PA5490**	22779	7,04	529,27	11	24,90	0,92	-0,11	-0,48	0,63	1394	**0,98**	1,13	0,18	0,58	0,56	1246	**0,97**	1,36	0,44	1,43	0,15	505	**0,62**	1,14	3,00	0,19	0,22
**PA5492**	25592	6,52	478,18	12	38,93	0,94	-0,08	-0,37	0,71	1575	**0,98**	1,06	0,08	0,20	0,85	1866	**0,98**	0,99	-0,02	-0,13	0,90	1849	**1,00**	1,00	3,00	-0,01	0,06
**PA5493**	106307	5,00	2127,84	51	41,60	0,91	-0,14	-0,57	0,57	1249	**0,99**	0,88	-0,19	-0,80	0,43	981	**0,94**	0,81	-0,30	-1,08	0,28	686	**0,84**	0,87	3,00	-0,21	0,05
**PA5495**	35824	5,46	377,27	10	32,30	1,06	0,08	0,19	0,85	1865	**0,99**	0,84	-0,25	-1,01	0,31	783	**0,86**	0,89	-0,18	-0,65	0,52	1114	**0,95**	0,93	3,00	-0,11	0,11
**PA5497**	87760	5,76	160,91	5	9,44	0,80	-0,32	-1,22	0,22	621	**0,78**	0,96	-0,06	-0,31	0,76	1672	**0,98**	0,91	-0,13	-0,51	0,61	1262	**0,99**	0,89	3,00	-0,17	0,08
**PA5503**	37995	6,15	561,67	17	29,80	1,09	0,12	0,35	0,73	1605	**0,99**	1,10	0,14	0,42	0,67	1503	**0,97**	1,17	0,23	0,71	0,48	1049	**0,94**	1,12	3,00	0,16	0,04
**PA5504**	25105	5,78	67,42	2	11,10	1,07	0,10	0,26	0,80	1756	**0,98**	1,34	0,43	1,49	0,14	501	**0,59**	1,26	0,33	1,06	0,29	696	**0,85**	1,22	3,00	0,29	0,14
**PA5505**	32083	7,79	1064,97	22	53,42	0,97	-0,05	-0,25	0,80	1774	**0,98**	1,00	0,01	-0,07	0,94	2051	**0,99**	0,96	-0,06	-0,27	0,79	1625	**1,00**	0,98	3,00	-0,03	0,02
**PA5506**	33080	7,08	411,81	10	30,76	1,79	0,84	2,86	0,00	164	**0,06**	1,74	0,80	2,87	0,00	190	**0,05**	1,57	0,65	2,15	0,03	279	**0,23**	1,70	3,00	0,76	0,11
**PA5507**	25198	5,97	894,18	19	63,38	2,10	1,07	3,67	0,00	110	**0,00**	1,73	0,79	2,85	0,00	193	**0,05**	1,67	0,74	2,44	0,01	234	**0,13**	1,83	3,00	0,87	0,23
**PA5508**	49209	5,71	248,65	6	13,17	2,05	1,03	3,55	0,00	114	**0,01**	1,43	0,52	1,84	0,07	377	**0,38**	1,75	0,81	2,69	0,01	206	**0,07**	1,74	3,00	0,80	0,31
**PA5509**	25030	6,06	206,20	7	22,07	1,75	0,81	2,75	0,01	175	**0,07**	1,91	0,94	3,39	0,00	151	**0,01**	1,61	0,68	2,26	0,02	262	**0,19**	1,76	3,00	0,81	0,15
**PA5510**	49847	9,17	95,67	2	3,10	1,50	0,58	1,96	0,05	284	**0,38**	1,10	0,14	0,41	0,68	1525	**0,97**	1,47	0,56	1,84	0,07	332	**0,41**	1,36	3,00	0,44	0,22
**PA5511**	52035	5,65	101,88	4	10,78	0,99	-0,02	-0,16	0,88	1928	**0,99**	0,83	-0,26	-1,08	0,28	723	**0,83**	1,02	0,02	0,02	0,98	2029	**1,00**	0,94	3,00	-0,08	0,10
**PA5516**	32308	5,69	109,85	3	9,27	0,87	-0,21	-0,81	0,42	966	**0,94**	1,01	0,02	-0,02	0,98	2137	**1,00**	0,79	-0,34	-1,20	0,23	614	**0,78**	0,89	3,00	-0,17	0,11
**PA5521**	28647	5,69	315,70	6	35,50	1,14	0,18	0,56	0,57	1268	**0,98**	1,16	0,22	0,72	0,47	1067	**0,96**	1,01	0,02	0,01	0,99	2046	**1,00**	1,10	3,00	0,14	0,08
**PA5523**	50294	6,25	388,61	8	16,59	1,40	0,48	1,61	0,11	413	**0,56**	1,16	0,22	0,72	0,47	1068	**0,96**	1,20	0,27	0,85	0,40	888	**0,92**	1,25	3,00	0,33	0,13
**PA5528**	31450	6,10	570,92	11	37,00	1,38	0,46	1,54	0,12	444	**0,61**	1,13	0,17	0,54	0,59	1319	**0,97**	1,06	0,08	0,22	0,83	1699	**1,00**	1,19	3,00	0,25	0,17
**PA5530**	50652	8,95	619,62	12	13,11	3,12	1,64	5,68	0,00	62	**0,00**	3,13	1,65	6,04	0,00	63	**0,00**	4,42	2,14	7,21	0,00	45	**0,00**	3,56	3,00	1,83	0,75
**PA5531**	40852	8,70	142,60	4	15,60	2,47	1,31	4,50	0,00	84	**0,00**	2,07	1,05	3,80	0,00	127	**0,00**	2,27	1,18	3,95	0,00	114	**0,00**	2,27	3,00	1,18	0,20
**PA5549**	69844	5,65	1471,90	29	43,77	0,92	-0,12	-0,51	0,61	1340	**0,99**	0,99	-0,02	-0,16	0,87	1929	**0,98**	0,94	-0,08	-0,34	0,73	1508	**1,00**	0,95	3,00	-0,07	0,04
**PA5552**	51311	5,82	1133,99	25	38,45	1,07	0,10	0,28	0,78	1719	**0,98**	1,11	0,15	0,45	0,66	1465	**0,97**	1,03	0,04	0,09	0,93	1918	**1,00**	1,07	3,00	0,10	0,04
**PA5553**	16060	5,14	509,13	10	48,48	1,08	0,11	0,31	0,75	1656	**0,99**	1,09	0,12	0,36	0,72	1610	**0,97**	1,19	0,25	0,79	0,43	952	**0,94**	1,12	3,00	0,16	0,06
**PA5554**	52829	4,98	4865,32	82	85,20	1,11	0,15	0,43	0,67	1467	**0,99**	1,12	0,16	0,52	0,61	1352	**0,97**	1,11	0,15	0,44	0,66	1358	**1,00**	1,11	3,00	0,15	0,01
**PA5555**	34409	7,70	1516,30	30	79,37	1,14	0,18	0,56	0,57	1270	**0,98**	1,15	0,20	0,63	0,53	1186	**0,96**	1,10	0,14	0,41	0,68	1400	**1,01**	1,13	3,00	0,17	0,02
**PA5556**	59676	5,33	3179,72	70	61,01	1,09	0,13	0,37	0,71	1573	**0,98**	1,15	0,20	0,66	0,51	1146	**0,97**	1,17	0,23	0,72	0,47	1038	**0,93**	1,14	3,00	0,19	0,04
**PA5557**	21312	5,78	881,33	22	79,01	1,11	0,15	0,45	0,65	1444	**0,98**	1,12	0,16	0,50	0,62	1382	**0,96**	1,17	0,22	0,69	0,49	1065	**0,94**	1,13	3,00	0,18	0,03
**PA5558**	18689	5,78	1126,86	23	69,20	1,17	0,23	0,73	0,46	1038	**0,97**	1,16	0,21	0,69	0,49	1097	**0,96**	1,19	0,25	0,78	0,43	953	**0,94**	1,17	3,00	0,23	0,01
**PA5560**	33341	6,03	366,20	7	21,80	1,13	0,18	0,54	0,59	1291	**0,99**	1,08	0,11	0,29	0,77	1694	**0,98**	1,07	0,10	0,27	0,79	1623	**1,00**	1,09	3,00	0,13	0,03
**PA5562**	33823	9,39	628,12	16	58,17	0,87	-0,21	-0,81	0,42	967	**0,93**	0,88	-0,19	-0,79	0,43	985	**0,94**	0,89	-0,17	-0,62	0,53	1146	**0,96**	0,88	3,00	-0,19	0,01
**PA5563**	30216	5,84	289,77	7	25,74	0,94	-0,08	-0,37	0,71	1580	**0,98**	1,08	0,11	0,31	0,76	1675	**0,98**	0,96	-0,06	-0,27	0,79	1626	**1,00**	0,99	3,00	-0,01	0,07
**PA5564**	25207	5,90	269,97	8	37,85	0,97	-0,05	-0,25	0,81	1775	**0,99**	0,93	-0,11	-0,49	0,62	1396	**0,96**	0,85	-0,23	-0,83	0,41	914	**0,92**	0,92	3,00	-0,12	0,06
**PA5565**	72819	6,08	613,72	17	31,98	0,84	-0,25	-0,96	0,34	812	**0,90**	0,93	-0,10	-0,48	0,63	1414	**0,96**	1,01	0,01	-0,01	0,99	2047	**1,00**	0,93	3,00	-0,11	0,08
**PA5567**	50437	5,13	291,67	7	15,60	0,98	-0,02	-0,16	0,87	1924	**0,98**	1,06	0,09	0,22	0,82	1816	**0,98**	1,11	0,16	0,47	0,64	1311	**1,00**	1,05	3,00	0,07	0,07
**PA5568**	69406	7,63	940,56	17	26,26	1,12	0,17	0,51	0,61	1350	**0,99**	1,13	0,18	0,58	0,56	1260	**0,97**	1,22	0,29	0,93	0,35	819	**0,89**	1,16	3,00	0,21	0,05
**PA5569**	16858	10,89	121,35	2	20,40	0,88	-0,18	-0,70	0,48	1084	**0,97**	1,11	0,15	0,48	0,63	1423	**0,96**	1,01	0,02	0,00	1,00	2057	**1,00**	1,00	3,00	0,00	0,11
**PA5570**	6071	13,00	38,38	2	20,50	1,26	0,34	1,11	0,27	694	**0,84**	1,16	0,21	0,69	0,49	1114	**0,96**	0,85	-0,24	-0,87	0,39	870	**0,92**	1,09	3,00	0,12	0,22
**PA0010**	21959	4,87	46,25	2	19,10	1,02	0,03	0,03	0,98	2133	**1,00**							1,07	0,10	0,27	0,79	1615	**1,00**	1,05	2,00	0,06	0,03
**PA0015**	12952	8,79	58,93	2	17,65	0,87	-0,20	-0,80	0,43	976	**0,95**							0,85	-0,23	-0,83	0,40	901	**0,93**	0,86	2,00	-0,22	0,01
**PA0046**	14200	5,66	80,02	2	22,65	0,83	-0,27	-1,04	0,30	741	**0,87**	1,15	0,20	0,63	0,53	1190	**0,96**							0,99	2,00	-0,02	0,22
**PA0091**	74884	5,98	42,17	2	7,55	0,90	-0,16	-0,63	0,53	1163	**0,99**							0,82	-0,28	-1,01	0,31	745	**0,86**	0,86	2,00	-0,22	0,05
**PA0092**	11392	6,56	158,49	6	64,20							0,92	-0,12	-0,54	0,59	1312	**0,97**	1,13	0,18	0,54	0,59	1236	**0,99**	1,02	2,00	0,04	0,15

**PA0095**	87109	5,37	60,69	2	5,40							1,17	0,23	0,75	0,45	1033	**0,95**	1,03	0,04	0,07	0,94	1941	**1,00**	1,10	2,00	0,14	0,10
**PA0115**	17742	5,85	85,61	4	25,30	0,94	-0,08	-0,37	0,71	1574	**0,98**							0,81	-0,31	-1,10	0,27	670	**0,84**	0,88	2,00	-0,19	0,10
**PA0118**	22757	6,30	135,81	4	21,65	0,94	-0,09	-0,39	0,70	1541	**0,98**	1,14	0,19	0,61	0,54	1209	**0,96**							1,04	2,00	0,06	0,14
**PA0127**	18701	8,68	65,07	2	15,10	0,84	-0,25	-0,96	0,34	806	**0,90**	0,66	-0,61	-2,36	0,02	258	**0,15**							0,75	2,00	-0,42	0,13
**PA0262**	118784	5,80	54,93	2	2,10	0,70	-0,51	-1,86	0,06	311	**0,44**	2,17	1,12	4,07	0,00	111	**0,00**							1,44	2,00	0,52	1,04
**PA0305**	90945	5,53	44,49	2	4,00							0,93	-0,10	-0,46	0,65	1445	**0,97**	0,85	-0,24	-0,88	0,38	862	**0,91**	0,89	2,00	-0,17	0,06
**PA0333**	46186	9,67	114,34	2	7,10							0,81	-0,30	-1,20	0,23	632	**0,79**	1,24	0,31	0,99	0,32	763	**0,87**	1,03	2,00	0,04	0,30
**PA0345**	50294	6,92	66,53	2	4,00	0,85	-0,24	-0,91	0,36	859	**0,91**							0,99	-0,01	-0,11	0,92	1883	**1,00**	0,92	2,00	-0,12	0,10
**PA0358**	23482	9,94	51,92	2	6,30							1,25	0,33	1,12	0,26	698	**0,82**	1,77	0,83	2,74	0,01	201	**0,06**	1,51	2,00	0,60	0,37
**PA0366**	56393	8,91	87,39	2	4,20							1,10	0,14	0,42	0,67	1500	**0,97**	0,76	-0,39	-1,38	0,17	529	**0,65**	0,93	2,00	-0,10	0,24
**PA0386**	43982	5,30	113,49	3	4,50	1,03	0,04	0,07	0,94	2051	**1,00**	1,08	0,12	0,34	0,73	1627	**0,98**							1,06	2,00	0,08	0,04
**PA0406**	38780	9,99	45,27	3	6,90	0,83	-0,27	-1,04	0,30	742	**0,87**	0,66	-0,61	-2,35	0,02	261	**0,15**							0,74	2,00	-0,43	0,12
**PA0414**	38544	5,09	72,05	2	9,90							1,10	0,13	0,39	0,70	1548	**0,97**	0,73	-0,45	-1,57	0,12	432	**0,56**	0,91	2,00	-0,13	0,26
**PA0424**	17668	5,64	91,29	3	25,87	1,32	0,40	1,33	0,18	544	**0,73**	0,90	-0,16	-0,69	0,49	1112	**0,96**							1,11	2,00	0,15	0,30
**PA0461**	37643	9,24	85,53	2	6,10							0,95	-0,07	-0,37	0,71	1587	**0,97**	1,02	0,02	0,02	0,98	2037	**1,00**	0,98	2,00	-0,02	0,05
**PA0464**	54046	5,85	51,72	2	6,10	1,02	0,03	0,03	0,97	2126	**1,00**							0,89	-0,16	-0,61	0,54	1163	**0,96**	0,96	2,00	-0,06	0,09
**PA0467**	24146	6,92	101,13	2	16,00	1,04	0,05	0,10	0,92	1991	**1,00**							0,89	-0,17	-0,63	0,53	1138	**0,95**	0,96	2,00	-0,05	0,10
**PA0468**	34340	5,05	63,46	3	13,30	1,01	0,01	-0,05	0,96	2091	**1,00**	1,03	0,04	0,05	0,96	2096	**0,99**							1,02	2,00	0,02	0,02
**PA0507**	68417	5,99	84,33	3	6,50	0,91	-0,14	-0,55	0,58	1281	**0,98**	0,69	-0,53	-2,08	0,04	311	**0,26**							0,80	2,00	-0,32	0,16
**PA0563**	14484	9,33	84,24	2	12,00							0,75	-0,41	-1,63	0,10	444	**0,50**	0,88	-0,19	-0,69	0,49	1068	**0,94**	0,82	2,00	-0,29	0,09
**PA0568**	18098	4,50	61,34	2	11,85							1,01	0,01	-0,06	0,95	2073	**0,99**	1,11	0,15	0,46	0,65	1332	**1,00**	1,06	2,00	0,08	0,07
**PA0584**	47524	6,42	50,82	2	13,40	1,28	0,35	1,16	0,25	657	**0,82**							0,88	-0,19	-0,68	0,49	1075	**0,95**	1,08	2,00	0,11	0,28
**PA0597**	24901	5,70	133,64	2	13,40	1,07	0,10	0,26	0,80	1752	**0,99**	0,80	-0,32	-1,30	0,19	568	**0,73**							0,93	2,00	-0,10	0,19
**PA0601**	24053	6,09	63,88	2	14,80	0,79	-0,34	-1,28	0,20	574	**0,75**	1,41	0,50	1,76	0,08	398	**0,42**							1,10	2,00	0,14	0,44
**PA0618**	32575	4,60	67,62	2	5,90							0,89	-0,17	-0,72	0,47	1066	**0,96**	0,78	-0,36	-1,29	0,20	563	**0,72**	0,83	2,00	-0,26	0,08
**PA0633**	18871	4,93	48,84	2	8,50							0,67	-0,58	-2,26	0,02	273	**0,19**	0,56	-0,83	-2,87	0,00	180	**0,05**	0,62	2,00	-0,70	0,08
**PA0653**	16806	5,95	137,28	4	23,55	0,88	-0,19	-0,76	0,45	1016	**0,96**							1,13	0,17	0,53	0,59	1237	**0,99**	1,00	2,00	0,00	0,18
**PA0658**	30158	10,00	107,79	3	9,23	0,80	-0,33	-1,22	0,22	615	**0,78**							0,74	-0,42	-1,50	0,13	466	**0,59**	0,77	2,00	-0,37	0,04
**PA0741**	23727	6,16	66,64	3	13,60	1,13	0,17	0,52	0,60	1330	**0,99**							0,87	-0,20	-0,75	0,45	1003	**0,93**	1,00	2,00	0,00	0,18
**PA0832**	24562	5,76	89,54	2	9,85	0,86	-0,21	-0,83	0,41	944	**0,94**	0,94	-0,09	-0,45	0,66	1464	**0,97**							0,90	2,00	-0,15	0,05
**PA0851**	35241	6,27	89,70	3	12,80	1,66	0,73	2,48	0,01	207	**0,14**	0,98	-0,03	-0,21	0,83	1833	**0,98**							1,32	2,00	0,40	0,48
**PA0860**	68005	8,59	93,72	2	5,90							1,24	0,31	1,05	0,29	750	**0,85**	1,09	0,13	0,37	0,71	1461	**1,00**	1,16	2,00	0,22	0,10
**PA0863**	34927	5,18	50,69	2	7,50	1,17	0,22	0,69	0,49	1092	**0,97**	1,01	0,01	-0,04	0,97	2105	**0,99**							1,09	2,00	0,12	0,11
**PA0905**	7625	6,73	75,74	3	63,90							1,07	0,09	0,25	0,81	1781	**0,98**	0,82	-0,28	-1,02	0,31	743	**0,85**	0,94	2,00	-0,08	0,17
**PA0915**	18938	5,95	116,94	2	10,50	1,06	0,08	0,21	0,84	1834	**0,99**	0,72	-0,46	-1,83	0,07	380	**0,38**							0,89	2,00	-0,17	0,24
**PA0917**	70270	6,96	97,54	2	1,95	1,04	0,06	0,13	0,90	1970	**0,99**							1,28	0,35	1,14	0,26	644	**0,82**	1,16	2,00	0,21	0,17
**PA0920**	98475	9,55	120,44	4	6,50							1,19	0,25	0,85	0,40	923	**0,93**	1,48	0,57	1,86	0,06	330	**0,39**	1,34	2,00	0,42	0,20
**PA0941**	9236	5,83	58,91	2	15,60							0,65	-0,61	-2,39	0,02	253	**0,15**	0,70	-0,51	-1,77	0,08	356	**0,44**	0,68	2,00	-0,56	0,04
**PA0975**	30869	6,60	143,26	3	12,10	1,11	0,15	0,43	0,66	1464	**0,99**	0,96	-0,06	-0,33	0,74	1643	**0,98**							1,03	2,00	0,05	0,11
**PA1051**	48927	8,32	159,48	3	3,47	1,22	0,29	0,93	0,35	838	**0,91**	1,29	0,37	1,28	0,20	579	**0,75**							1,26	2,00	0,33	0,05
**PA1075**	12195	4,46	77,22	2	21,00	1,24	0,31	0,99	0,32	780	**0,89**							1,64	0,72	2,38	0,02	243	**0,15**	1,44	2,00	0,53	0,29
**PA1089**	23171	5,17	39,86	2	10,50							1,05	0,07	0,18	0,86	1891	**0,98**	1,08	0,12	0,34	0,73	1515	**1,00**	1,07	2,00	0,10	0,02
**PA1096**	11575	4,47	69,71	2	12,20	0,88	-0,19	-0,73	0,46	1041	**0,97**	1,03	0,04	0,05	0,96	2097	**0,99**							0,95	2,00	-0,07	0,10
**PA1103**	31487	4,78	70,24	2	9,30	1,27	0,34	1,13	0,26	677	**0,83**	1,25	0,32	1,09	0,28	716	**0,83**							1,26	2,00	0,33	0,02
**PA1122**	16802	5,20	173,07	3	20,20	1,07	0,10	0,26	0,79	1746	**0,99**	1,05	0,07	0,15	0,88	1945	**0,98**							1,06	2,00	0,08	0,02
**PA1137**	36151	5,72	70,73	3	11,60	1,44	0,52	1,75	0,08	357	**0,48**	1,17	0,22	0,72	0,47	1057	**0,96**							1,30	2,00	0,38	0,19
**PA1151**	12051	6,74	175,72	5	41,40	0,96	-0,05	-0,26	0,79	1741	**0,99**	1,26	0,33	1,13	0,26	680	**0,82**							1,11	2,00	0,15	0,21
**PA1157**	27458	4,96	98,74	5	13,14							1,29	0,37	1,27	0,21	599	**0,74**	1,53	0,62	2,03	0,04	298	**0,30**	1,41	2,00	0,50	0,17

**PA1160**	14938	4,81	71,86	3	23,90	1,23	0,30	0,98	0,33	793	**0,90**	1,19	0,25	0,84	0,40	933	**0,93**							1,21	2,00	0,28	0,03
**PA1180**	51940	5,85	108,25	4	6,15	1,20	0,26	0,84	0,40	936	**0,94**							1,39	0,47	1,54	0,12	447	**0,57**	1,29	2,00	0,37	0,13
**PA1181**	128173	5,50	125,83	3	3,40	1,00	0,00	-0,06	0,95	2063	**1,00**	1,45	0,54	1,91	0,06	354	**0,34**							1,23	2,00	0,30	0,32
**PA1200**	25630	5,38	52,45	2	7,50							0,92	-0,12	-0,56	0,58	1294	**0,96**	1,20	0,26	0,83	0,41	913	**0,92**	1,06	2,00	0,08	0,20
**PA1228**	13357	10,81	117,01	2	25,45							0,93	-0,11	-0,52	0,61	1353	**0,97**	0,61	-0,72	-2,50	0,01	225	**0,11**	0,77	2,00	-0,38	0,23
**PA1243**	95605	5,17	116,49	4	5,85	1,18	0,24	0,77	0,44	1003	**0,95**	0,98	-0,02	-0,18	0,86	1890	**0,98**							1,08	2,00	0,12	0,14
**PA1250**	14587	5,32	44,62	2	18,30	1,35	0,44	1,45	0,15	484	**0,66**	1,00	0,00	-0,08	0,94	2041	**0,99**							1,18	2,00	0,24	0,25
**PA1277**	54200	5,99	137,57	4	11,40							1,12	0,16	0,50	0,62	1380	**0,96**	1,12	0,17	0,51	0,61	1264	**0,99**	1,12	2,00	0,16	0,00
**PA1285**	17100	6,96	50,56	2	9,40	0,91	-0,13	-0,53	0,60	1305	**0,99**	0,89	-0,16	-0,70	0,49	1095	**0,96**							0,90	2,00	-0,14	0,01
**PA1295**	12409	5,37	59,12	2	32,00	1,36	0,44	1,46	0,14	480	**0,65**	1,26	0,33	1,14	0,25	673	**0,82**							1,31	2,00	0,39	0,07
**PA1417**	58828	5,79	169,10	4	9,28							1,29	0,36	1,25	0,21	609	**0,75**	1,01	0,02	0,00	1,00	2058	**1,00**	1,15	2,00	0,20	0,19
**PA1463**	33272	4,56	165,50	3	9,10							0,94	-0,09	-0,42	0,67	1499	**0,97**	1,19	0,25	0,78	0,43	959	**0,93**	1,06	2,00	0,09	0,17
**PA1473**	12620	4,68	109,27	3	20,10							1,18	0,24	0,80	0,43	984	**0,94**	1,19	0,25	0,78	0,44	965	**0,93**	1,18	2,00	0,24	0,00
**PA1474**	55008	6,11	159,92	3	7,77	1,76	0,81	2,78	0,01	172	**0,07**	1,09	0,13	0,38	0,70	1559	**0,97**							1,43	2,00	0,51	0,47
**PA1490**	29085	6,68	78,74	3	14,20	0,96	-0,06	-0,29	0,77	1691	**0,99**	1,06	0,09	0,23	0,81	1802	**0,98**							1,01	2,00	0,02	0,07
**PA1513**	47864	8,60	94,23	2	13,90							1,15	0,20	0,65	0,51	1154	**0,96**	1,70	0,77	2,55	0,01	218	**0,10**	1,43	2,00	0,51	0,39
**PA1552.1**	7759	8,09	192,32	3	32,23	1,35	0,44	1,45	0,15	490	**0,66**							2,00	1,00	3,32	0,00	145	**0,01**	1,67	2,00	0,74	0,45
**PA1556**	25207	7,72	161,54	4	31,20	0,97	-0,05	-0,25	0,80	1769	**0,99**	1,16	0,21	0,69	0,49	1096	**0,96**							1,06	2,00	0,09	0,14
**PA1558**	23804	9,46	63,21	2	6,65	1,43	0,52	1,74	0,08	363	**0,49**	1,05	0,07	0,18	0,86	1898	**0,98**							1,24	2,00	0,31	0,27
**PA1565**	50531	7,30	32,12	2	3,45							1,10	0,13	0,39	0,69	1546	**0,97**	1,16	0,22	0,68	0,49	1076	**0,95**	1,13	2,00	0,18	0,05
**PA1572**	42019	5,15	88,20	2	5,75	1,33	0,41	1,35	0,18	534	**0,72**	2,09	1,06	3,85	0,00	126	**0,00**							1,71	2,00	0,77	0,54
**PA1611**	73119	5,01	104,30	3	4,60	1,17	0,23	0,72	0,47	1052	**0,97**	1,11	0,14	0,44	0,66	1479	**0,97**							1,14	2,00	0,19	0,05
**PA1636**	98873	5,84	112,76	3	3,77	1,68	0,75	2,55	0,01	194	**0,12**							1,49	0,58	1,89	0,06	321	**0,37**	1,59	2,00	0,67	0,14
**PA1655**	24689	5,49	57,87	2	10,75	1,15	0,20	0,63	0,53	1155	**0,99**	1,22	0,28	0,96	0,33	816	**0,89**							1,19	2,00	0,24	0,05
**PA1675**	22775	9,26	40,67	2	7,30	1,15	0,21	0,64	0,52	1145	**0,99**	1,06	0,08	0,19	0,85	1872	**0,98**							1,10	2,00	0,14	0,07
**PA1686**	32957	6,36	57,94	2	7,25	1,20	0,27	0,86	0,39	913	**0,93**							1,20	0,27	0,85	0,39	884	**0,92**	1,20	2,00	0,27	0,00
**PA1688**	32877	5,24	90,89	3	10,70	1,40	0,49	1,63	0,10	407	**0,56**							1,21	0,28	0,88	0,38	853	**0,91**	1,31	2,00	0,39	0,13
**PA1738**	34252	7,18	88,43	3	13,80							0,93	-0,11	-0,51	0,61	1368	**0,96**	0,98	-0,03	-0,15	0,88	1809	**1,00**	0,95	2,00	-0,07	0,04
**PA1760**	105864	6,35	89,46	3	3,90	1,67	0,74	2,51	0,01	204	**0,13**							1,88	0,91	3,03	0,00	168	**0,03**	1,77	2,00	0,83	0,15
**PA1776**	21710	8,41	99,02	3	20,60	1,25	0,32	1,03	0,30	750	**0,88**	1,11	0,15	0,46	0,64	1442	**0,97**							1,18	2,00	0,24	0,10
**PA1808**	39479	9,19	34,59	2	8,00	1,25	0,33	1,06	0,29	723	**0,87**	1,88	0,91	3,30	0,00	155	**0,01**							1,57	2,00	0,65	0,44
**PA1809**	41414	6,13	62,74	2	7,50	1,06	0,09	0,22	0,82	1806	**0,99**							1,06	0,09	0,24	0,81	1659	**1,00**	1,06	2,00	0,09	0,00
**PA1831**	27227	5,88	76,11	2	10,80	1,11	0,15	0,45	0,66	1446	**0,99**	1,31	0,39	1,36	0,17	546	**0,69**							1,21	2,00	0,28	0,14
**PA2076**	33631	5,73	87,25	3	17,30	1,09	0,13	0,37	0,71	1571	**0,98**							0,88	-0,19	-0,69	0,49	1064	**0,94**	0,99	2,00	-0,02	0,15
**PA2100**	54057	6,69	86,64	3	7,57							0,78	-0,36	-1,44	0,15	515	**0,63**	1,14	0,19	0,60	0,55	1179	**0,96**	0,96	2,00	-0,06	0,26
**PA2115**	36693	7,82	126,74	3	13,77	1,49	0,58	1,94	0,05	291	**0,39**							1,75	0,81	2,68	0,01	208	**0,07**	1,62	2,00	0,70	0,18
**PA2119**	40778	5,43	145,76	6	23,47	1,11	0,15	0,45	0,65	1438	**0,98**	1,05	0,08	0,19	0,85	1877	**0,98**							1,08	2,00	0,11	0,04
**PA2241**	50122	10,06	87,38	2	7,70	1,28	0,35	1,15	0,25	664	**0,82**	1,37	0,45	1,58	0,11	462	**0,53**							1,32	2,00	0,40	0,06
**PA2379**	18272	6,49	94,51	2	20,50	1,01	0,01	-0,04	0,97	2113	**1,00**	1,03	0,05	0,08	0,94	2040	**0,99**							1,02	2,00	0,03	0,02
**PA2399**	278779	5,22	137,20	7	5,30	1,74	0,79	2,71	0,01	178	**0,08**							2,09	1,06	3,54	0,00	130	**0,01**	1,91	2,00	0,93	0,25
**PA2413**	52098	6,03	146,11	4	9,55	2,42	1,28	4,40	0,00	87	**0,00**	2,38	1,25	4,57	0,00	100	**0,00**							2,40	2,00	1,26	0,03
**PA2449**	57426	5,84	85,95	2	5,80	1,11	0,15	0,44	0,66	1457	**0,99**	0,76	-0,39	-1,56	0,12	468	**0,54**							0,93	2,00	-0,10	0,25
**PA2450**	35595	6,03	39,21	2	5,75							1,20	0,26	0,87	0,38	897	**0,93**	0,99	-0,01	-0,10	0,92	1895	**1,00**	1,09	2,00	0,13	0,15
**PA2535**	38349	6,42	74,21	2	8,65							0,56	-0,83	-3,20	0,00	162	**0,02**	0,94	-0,09	-0,36	0,72	1478	**1,00**	0,75	2,00	-0,41	0,27
**PA2541**	23293	9,34	40,56	2	4,70	1,13	0,17	0,52	0,60	1319	**0,99**							0,92	-0,12	-0,47	0,64	1317	**1,00**	1,02	2,00	0,03	0,15
**PA2550**	47988	6,16	33,75	2	3,15	1,15	0,20	0,63	0,53	1156	**0,99**	1,16	0,21	0,69	0,49	1107	**0,96**							1,15	2,00	0,21	0,00
**PA2583**	112753	5,63	55,18	2	3,10	1,56	0,64	2,17	0,03	254	**0,26**							0,95	-0,08	-0,32	0,75	1549	**1,00**	1,25	2,00	0,33	0,43
**PA2586**	25665	5,84	70,09	2	5,60							0,92	-0,11	-0,52	0,60	1351	**0,97**	1,10	0,13	0,39	0,70	1433	**1,00**	1,01	2,00	0,01	0,12

**PA2601**	33260	5,56	39,87	2	10,10	0,94	-0,08	-0,37	0,71	1579	**0,98**	0,88	-0,18	-0,76	0,45	1027	**0,95**							0,91	2,00	-0,13	0,04
**PA2605**	14457	5,51	121,78	4	19,85	0,94	-0,08	-0,38	0,71	1563	**0,98**							0,86	-0,22	-0,79	0,43	951	**0,94**	0,90	2,00	-0,15	0,06
**PA2609**	38371	6,25	92,97	3	9,80	0,95	-0,07	-0,32	0,75	1650	**0,99**	0,77	-0,37	-1,48	0,14	503	**0,60**							0,86	2,00	-0,21	0,13
**PA2621**	9936	4,80	72,44	3	24,40	0,82	-0,29	-1,12	0,26	685	**0,84**	1,07	0,10	0,27	0,79	1747	**0,98**							0,94	2,00	-0,09	0,18
**PA2622**	10603	5,54	104,65	3	40,00	0,79	-0,35	-1,30	0,19	566	**0,75**	0,64	-0,65	-2,52	0,01	228	**0,11**							0,71	2,00	-0,49	0,11
**PA2648**	57534	7,21	110,62	2	4,30	0,98	-0,03	-0,20	0,84	1848	**0,99**	1,23	0,30	1,00	0,32	792	**0,86**							1,10	2,00	0,14	0,18
**PA2657**	25526	5,62	60,54	2	6,30	0,92	-0,13	-0,52	0,60	1326	**0,99**							1,02	0,02	0,02	0,98	2027	**1,00**	0,97	2,00	-0,05	0,07
**PA2679**	28483	5,29	81,15	2	12,80	1,09	0,12	0,34	0,74	1623	**0,99**	0,97	-0,05	-0,27	0,79	1742	**0,98**							1,03	2,00	0,04	0,08
**PA2720**	24469	4,97	177,02	3	18,23	1,37	0,46	1,52	0,13	456	**0,61**	0,96	-0,06	-0,33	0,74	1644	**0,98**							1,17	2,00	0,22	0,29
**PA2730**	39451	5,97	59,72	2	5,50	1,04	0,06	0,14	0,89	1948	**0,99**	1,18	0,24	0,81	0,42	969	**0,94**							1,11	2,00	0,16	0,10
**PA2737**	11112	9,81	68,49	2	32,90							1,15	0,20	0,64	0,52	1183	**0,96**	1,67	0,74	2,46	0,01	229	**0,12**	1,41	2,00	0,50	0,37
**PA2742**	9377	12,24	52,71	2	10,90	0,98	-0,03	-0,18	0,85	1877	**0,99**	0,98	-0,03	-0,21	0,84	1848	**0,98**							0,98	2,00	-0,03	0,00
**PA2813**	24248	5,06	51,52	2	16,00	0,97	-0,04	-0,23	0,81	1791	**0,99**	0,85	-0,24	-1,00	0,32	798	**0,86**							0,91	2,00	-0,14	0,09
**PA2815**	93876	5,69	85,71	3	5,07	0,97	-0,05	-0,25	0,80	1762	**0,99**	0,65	-0,62	-2,39	0,02	252	**0,14**							0,81	2,00	-0,31	0,22
**PA2820**	31471	8,61	134,85	3	15,47	0,79	-0,34	-1,29	0,20	572	**0,75**							1,01	0,01	-0,02	0,98	2035	**1,00**	0,90	2,00	-0,16	0,16
**PA2821**	25946	5,88	63,68	2	9,30							0,76	-0,39	-1,56	0,12	473	**0,55**	0,78	-0,35	-1,25	0,21	578	**0,75**	0,77	2,00	-0,37	0,01
**PA2822**	17879	4,63	84,71	3	16,90	0,91	-0,14	-0,57	0,57	1257	**0,98**	1,02	0,03	0,02	0,98	2133	**1,00**							0,97	2,00	-0,05	0,08
**PA2829**	17188	5,37	76,41	2	11,35	0,92	-0,12	-0,50	0,62	1362	**0,99**							0,99	-0,02	-0,13	0,90	1841	**1,00**	0,95	2,00	-0,07	0,05
**PA2906**	52936	8,30	85,88	3	7,97	1,30	0,38	1,27	0,21	584	**0,76**	0,88	-0,18	-0,78	0,44	1004	**0,94**							1,09	2,00	0,13	0,30
**PA2911**	82115	6,24	125,23	3	5,63	0,85	-0,24	-0,93	0,35	842	**0,91**	0,80	-0,32	-1,28	0,20	584	**0,74**							0,82	2,00	-0,28	0,03
**PA2942**	37526	5,63	204,81	4	15,40							0,82	-0,28	-1,14	0,25	675	**0,81**	0,94	-0,08	-0,34	0,74	1522	**1,00**	0,88	2,00	-0,18	0,09
**PA2962**	23397	5,14	238,80	6	19,53	0,93	-0,11	-0,47	0,64	1409	**0,99**	0,86	-0,22	-0,90	0,37	860	**0,92**							0,89	2,00	-0,16	0,05
**PA3031**	8774	4,92	201,40	3	35,60	0,65	-0,62	-2,26	0,02	243	**0,21**							0,91	-0,14	-0,52	0,61	1259	**0,99**	0,78	2,00	-0,36	0,18
**PA3035**	22957	8,07	66,55	2	9,60	1,06	0,08	0,21	0,83	1824	**0,99**							1,01	0,01	-0,03	0,97	2013	**1,00**	1,03	2,00	0,05	0,04
**PA3084**	32016	7,52	69,09	2	8,60	0,76	-0,40	-1,48	0,14	470	**0,64**							0,98	-0,03	-0,15	0,88	1807	**1,00**	0,87	2,00	-0,20	0,16
**PA3185**	33324	7,72	65,43	2	8,50	0,70	-0,52	-1,92	0,05	297	**0,40**	1,05	0,07	0,15	0,88	1935	**0,98**							0,87	2,00	-0,20	0,25
**PA3186**	54895	5,48	669,71	15	53,70	8,40	3,07	10,70	0,00	18	**0,00**							5,12	2,36	7,92	0,00	39	**0,00**	6,76	2,00	2,76	2,32
**PA3188**	31961	9,24	87,57	2	6,40							12,59	3,65	13,52	0,00	18	**0,00**	11,02	3,46	11,67	0,00	29	**0,00**	11,80	2,00	3,56	1,11
**PA3189**	35774	9,76	342,22	7	12,56	9,51	3,25	11,32	0,00	19	**0,00**	4,25	2,09	7,68	0,00	36	**0,00**							6,88	2,00	2,78	3,72
**PA3199**	24322	6,21	125,32	5	21,04							0,84	-0,26	-1,05	0,29	752	**0,84**	0,78	-0,35	-1,25	0,21	581	**0,75**	0,81	2,00	-0,30	0,04
**PA3214**	23967	9,97	82,62	2	11,20	0,88	-0,18	-0,73	0,47	1046	**0,97**	0,95	-0,07	-0,37	0,71	1575	**0,97**							0,92	2,00	-0,13	0,05
**PA3246**	25228	6,53	52,79	2	7,35	0,98	-0,03	-0,19	0,85	1869	**0,99**	1,11	0,15	0,44	0,66	1468	**0,97**							1,04	2,00	0,06	0,09
**PA3247**	48006	5,65	107,34	4	5,75	0,90	-0,15	-0,60	0,55	1213	**0,98**							1,22	0,28	0,90	0,37	832	**0,91**	1,06	2,00	0,08	0,22
**PA3260**	11208	4,83	116,10	4	25,20	0,91	-0,13	-0,53	0,60	1306	**0,99**	0,89	-0,17	-0,71	0,47	1072	**0,96**							0,90	2,00	-0,15	0,02
**PA3267**	71064	8,84	84,07	3	4,23							0,95	-0,07	-0,36	0,72	1606	**0,97**	0,94	-0,09	-0,35	0,72	1486	**1,00**	0,95	2,00	-0,08	0,01
**PA3314**	29283	9,75	90,44	3	11,77	0,97	-0,04	-0,23	0,82	1794	**0,99**	1,11	0,16	0,49	0,63	1412	**0,96**							1,04	2,00	0,06	0,10
**PA3345**	13798	5,56	53,40	2	26,70	0,97	-0,04	-0,23	0,82	1798	**0,99**	0,87	-0,21	-0,86	0,39	907	**0,93**							0,92	2,00	-0,12	0,07
**PA3350**	26779	8,52	54,76	2	7,80	0,86	-0,22	-0,85	0,40	925	**0,93**							0,87	-0,20	-0,73	0,46	1027	**0,93**	0,87	2,00	-0,21	0,01
**PA3364**	43443	5,63	38,36	2	10,40	2,00	1,00	3,43	0,00	121	**0,01**	1,95	0,96	3,49	0,00	143	**0,01**							1,97	2,00	0,98	0,04
**PA3385**	12758	6,97	119,82	2	15,75	0,63	-0,67	-2,43	0,01	214	**0,15**	0,69	-0,54	-2,09	0,04	307	**0,26**							0,66	2,00	-0,60	0,04
**PA3439**	15056	5,82	95,15	3	25,50	0,93	-0,10	-0,43	0,66	1462	**0,99**	0,81	-0,30	-1,21	0,23	628	**0,78**							0,87	2,00	-0,20	0,08
**PA3475**	32296	6,40	84,80	3	7,13	1,47	0,56	1,89	0,06	301	**0,43**							0,84	-0,25	-0,89	0,37	849	**0,91**	1,16	2,00	0,21	0,45
**PA3477**	29139	6,66	186,08	5	23,20							0,88	-0,18	-0,77	0,44	1012	**0,94**	0,63	-0,67	-2,31	0,02	256	**0,17**	0,76	2,00	-0,40	0,18
**PA3560**	61719	8,39	272,39	4	6,23							1,02	0,03	0,00	1,00	2158	**1,00**	0,69	-0,54	-1,90	0,06	317	**0,37**	0,85	2,00	-0,23	0,23
**PA3623**	32684	10,43	42,13	2	4,55	0,70	-0,51	-1,86	0,06	312	**0,44**	1,06	0,08	0,19	0,85	1867	**0,98**							0,88	2,00	-0,18	0,25
**PA3626**	40233	6,34	230,54	5	11,36							0,96	-0,05	-0,29	0,77	1697	**0,98**	0,85	-0,23	-0,83	0,41	909	**0,92**	0,91	2,00	-0,14	0,08
**PA3631**	45368	10,32	176,60	4	7,18							0,86	-0,22	-0,90	0,37	859	**0,92**	1,12	0,17	0,50	0,61	1271	**1,00**	0,99	2,00	-0,01	0,18
**PA3672**	34625	6,18	38,77	2	10,70	0,96	-0,06	-0,31	0,76	1672	**0,99**	1,03	0,04	0,04	0,97	2109	**0,99**							0,99	2,00	-0,01	0,05

**PA3691**	16234	7,85	93,97	2	14,90	0,85	-0,23	-0,89	0,37	885	**0,92**	1,00	0,00	-0,08	0,93	2033	**0,99**							0,93	2,00	-0,11	0,11
**PA3696**	28113	5,43	184,53	6	17,78	0,91	-0,13	-0,54	0,59	1294	**0,99**	0,79	-0,35	-1,38	0,17	537	**0,67**							0,85	2,00	-0,23	0,09
**PA3704**	86376	5,05	71,70	3	4,50							1,05	0,07	0,18	0,86	1900	**0,98**	1,02	0,02	0,02	0,98	2030	**1,00**	1,03	2,00	0,05	0,02
**PA3705**	25410	5,35	143,55	3	20,40	0,65	-0,63	-2,28	0,02	237	**0,21**							0,66	-0,60	-2,08	0,04	290	**0,27**	0,65	2,00	-0,61	0,01
**PA3706**	47666	7,72	100,50	3	10,17							0,62	-0,68	-2,63	0,01	218	**0,09**	0,92	-0,11	-0,44	0,66	1357	**1,00**	0,77	2,00	-0,37	0,21
**PA3717**	13835	6,29	95,05	2	22,60							0,85	-0,23	-0,96	0,34	822	**0,88**	0,90	-0,15	-0,57	0,57	1214	**0,97**	0,88	2,00	-0,19	0,04
**PA3730**	24403	5,48	150,62	4	24,40	0,89	-0,16	-0,65	0,51	1134	**0,98**							1,04	0,06	0,14	0,89	1831	**1,00**	0,97	2,00	-0,05	0,10
**PA3733**	46801	5,73	74,09	2	4,30	0,84	-0,24	-0,94	0,35	832	**0,91**	0,72	-0,47	-1,83	0,07	379	**0,38**							0,78	2,00	-0,35	0,09
**PA3748**	49559	5,04	93,65	2	6,00	1,04	0,05	0,10	0,92	1998	**1,00**							1,05	0,07	0,19	0,85	1752	**1,00**	1,04	2,00	0,06	0,01
**PA3759**	36959	5,56	97,00	3	12,17	0,84	-0,25	-0,94	0,35	827	**0,91**							1,31	0,39	1,26	0,21	577	**0,75**	1,08	2,00	0,11	0,33
**PA3760**	91324	5,43	86,88	3	5,63							1,33	0,41	1,43	0,15	519	**0,64**	1,39	0,47	1,55	0,12	443	**0,57**	1,36	2,00	0,44	0,04
**PA3761**	62496	8,24	46,82	2	2,65	1,16	0,22	0,68	0,50	1102	**0,98**							1,20	0,26	0,82	0,41	915	**0,92**	1,18	2,00	0,24	0,02
**PA3789**	53542	9,64	99,36	4	9,05	0,63	-0,68	-2,45	0,01	210	**0,15**	0,93	-0,10	-0,46	0,65	1443	**0,97**							0,78	2,00	-0,36	0,22
**PA3795**	36845	5,37	123,10	3	10,80	1,21	0,28	0,90	0,37	878	**0,91**	0,71	-0,49	-1,93	0,05	346	**0,33**							0,96	2,00	-0,06	0,36
**PA3811**	21147	4,93	56,06	2	13,90	0,86	-0,22	-0,87	0,39	907	**0,93**							1,07	0,09	0,25	0,80	1650	**1,00**	0,96	2,00	-0,06	0,15
**PA3840**	39519	9,01	121,88	3	9,93							0,70	-0,51	-2,01	0,04	323	**0,30**	0,88	-0,18	-0,66	0,51	1110	**0,95**	0,79	2,00	-0,33	0,13
**PA3852**	35346	5,42	141,46	4	8,70	1,07	0,09	0,25	0,80	1767	**0,99**	0,84	-0,25	-1,03	0,30	770	**0,85**							0,95	2,00	-0,07	0,16
**PA3919**	54780	5,85	137,48	4	8,65	1,12	0,16	0,47	0,64	1404	**0,99**	0,84	-0,25	-1,03	0,31	771	**0,86**							0,98	2,00	-0,03	0,19
**PA3941**	22582	5,57	55,31	2	21,20							0,81	-0,31	-1,26	0,21	602	**0,74**	0,69	-0,53	-1,85	0,06	331	**0,40**	0,75	2,00	-0,42	0,08
**PA3942**	34211	5,57	274,72	4	17,83	0,58	-0,78	-2,82	0,00	167	**0,06**	0,93	-0,11	-0,51	0,61	1364	**0,96**							0,75	2,00	-0,41	0,24
**PA3949**	43734	9,53	67,89	2	9,50	1,09	0,13	0,37	0,71	1582	**0,98**							0,91	-0,14	-0,53	0,60	1241	**0,99**	1,00	2,00	0,00	0,13
**PA3951**	19275	6,93	71,36	2	7,20	0,91	-0,14	-0,58	0,56	1235	**0,98**	1,12	0,16	0,49	0,62	1397	**0,96**							1,01	2,00	0,02	0,15
**PA3958**	43524	5,67	67,08	2	5,80	1,00	0,01	-0,06	0,95	2068	**1,00**							0,84	-0,25	-0,92	0,36	824	**0,90**	0,92	2,00	-0,12	0,12
**PA3968**	22683	9,68	33,21	2	9,80	0,65	-0,62	-2,25	0,02	245	**0,22**	0,96	-0,06	-0,31	0,76	1673	**0,98**							0,81	2,00	-0,31	0,22
**PA3978**	20194	4,57	43,50	2	9,90	0,97	-0,04	-0,23	0,82	1800	**0,99**	1,05	0,07	0,17	0,86	1908	**0,98**							1,01	2,00	0,02	0,06
**PA3982**	18874	4,37	129,16	3	18,53	1,07	0,10	0,27	0,79	1737	**0,99**	1,28	0,36	1,24	0,21	615	**0,75**							1,18	2,00	0,24	0,15
**PA4026**	19380	6,44	107,92	3	20,27	0,68	-0,55	-2,03	0,04	275	**0,34**							0,89	-0,17	-0,65	0,52	1117	**0,95**	0,78	2,00	-0,35	0,15
**PA4032**	28316	5,60	47,15	2	5,25	1,17	0,22	0,71	0,48	1075	**0,97**	1,11	0,15	0,45	0,65	1463	**0,97**							1,14	2,00	0,19	0,04
**PA4051**	33645	4,68	85,65	2	10,20							1,18	0,24	0,81	0,42	966	**0,94**	0,76	-0,39	-1,37	0,17	535	**0,66**	0,97	2,00	-0,04	0,30
**PA4060**	10884	4,72	42,20	2	13,00	1,30	0,37	1,24	0,22	609	**0,77**							0,68	-0,56	-1,96	0,05	311	**0,33**	0,99	2,00	-0,02	0,44
**PA4135**	17769	9,93	154,55	5	45,00	0,82	-0,28	-1,06	0,29	726	**0,86**	1,06	0,09	0,23	0,82	1811	**0,98**							0,94	2,00	-0,08	0,17
**PA4180**	63169	5,91	129,79	3	7,53	1,10	0,14	0,39	0,69	1525	**0,99**							0,75	-0,42	-1,47	0,14	483	**0,61**	0,92	2,00	-0,11	0,25
**PA4190**	44708	5,98	52,21	2	4,50	0,72	-0,48	-1,78	0,08	346	**0,48**	1,02	0,03	0,02	0,98	2131	**1,00**							0,87	2,00	-0,20	0,22
**PA4201**	38712	5,16	93,11	2	12,70	0,78	-0,35	-1,32	0,19	556	**0,73**	0,95	-0,08	-0,38	0,71	1568	**0,97**							0,87	2,00	-0,21	0,12
**PA4282**	142893	5,56	43,40	2	3,30							0,86	-0,21	-0,89	0,37	866	**0,93**	1,09	0,12	0,36	0,72	1485	**1,00**	0,98	2,00	-0,04	0,16
**PA4284**	142781	5,47	73,93	3	2,53	1,34	0,42	1,39	0,17	520	**0,69**	1,23	0,30	1,02	0,31	776	**0,85**							1,28	2,00	0,36	0,07
**PA4290**	59515	5,35	121,26	2	5,90	0,71	-0,50	-1,84	0,07	321	**0,44**	0,45	-1,14	-4,33	0,00	106	**0,00**							0,58	2,00	-0,78	0,18
**PA4323**	51718	9,79	68,15	3	5,67	1,14	0,19	0,59	0,56	1225	**0,99**	0,83	-0,28	-1,12	0,26	689	**0,82**							0,98	2,00	-0,02	0,22
**PA4361**	37484	8,22	57,92	3	9,10	1,10	0,14	0,42	0,67	1490	**0,98**							1,30	0,37	1,21	0,23	607	**0,77**	1,20	2,00	0,26	0,14
**PA4367**	80853	5,69	55,93	2	4,80	0,89	-0,17	-0,69	0,49	1091	**0,97**	1,00	0,00	-0,08	0,93	2032	**0,99**							0,94	2,00	-0,08	0,08
**PA4399**	21911	5,88	58,78	2	14,35							1,39	0,48	1,68	0,09	425	**0,47**	1,01	0,02	0,00	1,00	2055	**1,00**	1,20	2,00	0,27	0,27
**PA4427**	15162	4,57	56,07	2	25,90	1,04	0,06	0,12	0,90	1973	**0,99**	0,93	-0,11	-0,51	0,61	1369	**0,97**							0,98	2,00	-0,02	0,08
**PA4476**	146791	6,61	61,62	2	3,25	0,98	-0,03	-0,17	0,87	1906	**0,99**							0,85	-0,23	-0,84	0,40	892	**0,92**	0,92	2,00	-0,12	0,09
**PA4487**	29471	5,12	176,03	3	22,30							1,42	0,50	1,77	0,08	396	**0,42**	1,05	0,07	0,18	0,86	1771	**1,00**	1,23	2,00	0,30	0,26
**PA4494**	48137	6,26	75,42	2	5,10	1,12	0,16	0,49	0,62	1371	**0,99**	1,37	0,46	1,61	0,11	453	**0,52**							1,25	2,00	0,32	0,18
**PA4499**	21333	5,84	78,74	2	7,20	1,53	0,61	2,07	0,04	269	**0,31**	1,25	0,32	1,10	0,27	714	**0,83**							1,39	2,00	0,47	0,20
**PA4520**	75170	5,06	63,89	2	8,60	1,30	0,38	1,25	0,21	601	**0,77**							0,92	-0,13	-0,49	0,63	1291	**1,00**	1,11	2,00	0,15	0,27
**PA4521**	31252	8,89	111,09	2	7,75	0,92	-0,12	-0,50	0,62	1368	**0,99**	1,06	0,08	0,19	0,85	1868	**0,98**							0,99	2,00	-0,02	0,09

**PA4538**	49409	8,38	131,48	4	10,55	0,96	-0,06	-0,27	0,78	1733	**0,98**	0,82	-0,29	-1,17	0,24	645	**0,81**							0,89	2,00	-0,17	0,10
**PA4571**	77495	6,20	324,13	8	11,98	0,84	-0,25	-0,95	0,34	822	**0,91**	0,96	-0,05	-0,29	0,77	1703	**0,98**							0,90	2,00	-0,15	0,09
**PA4577**	12077	5,17	51,24	2	11,55	0,73	-0,46	-1,70	0,09	374	**0,51**	0,88	-0,19	-0,80	0,43	977	**0,94**							0,80	2,00	-0,32	0,11
**PA4585**	37178	7,74	66,63	2	17,90	1,30	0,38	1,25	0,21	593	**0,77**	1,20	0,26	0,87	0,39	901	**0,93**							1,25	2,00	0,32	0,07
**PA4618**	36092	5,51	104,84	4	8,95	0,93	-0,10	-0,42	0,67	1485	**0,98**	0,88	-0,18	-0,76	0,45	1021	**0,94**							0,91	2,00	-0,14	0,04
**PA4698**	12320	4,34	109,18	3	26,60	1,08	0,11	0,31	0,76	1662	**0,99**							1,60	0,68	2,25	0,02	264	**0,19**	1,34	2,00	0,42	0,37
**PA4717**	33033	5,87	166,03	3	14,50	0,87	-0,20	-0,78	0,44	998	**0,95**	0,99	-0,02	-0,15	0,88	1938	**0,98**							0,93	2,00	-0,10	0,08
**PA4769**	29874	6,02	48,91	2	9,55	0,63	-0,67	-2,43	0,01	215	**0,15**	0,68	-0,56	-2,20	0,03	286	**0,21**							0,65	2,00	-0,62	0,03
**PA4772**	108275	6,21	154,29	5	4,40							0,60	-0,73	-2,81	0,00	198	**0,05**	0,35	-1,52	-5,20	0,00	78	**0,00**	0,48	2,00	-1,07	0,18
**PA4773**	17834	5,24	85,06	3	17,30							1,29	0,36	1,26	0,21	604	**0,74**	0,94	-0,09	-0,36	0,72	1479	**1,00**	1,11	2,00	0,16	0,25
**PA4785**	48742	6,69	38,38	2	4,90							1,06	0,08	0,22	0,83	1822	**0,98**	0,90	-0,14	-0,55	0,58	1228	**0,98**	0,98	2,00	-0,03	0,11
**PA4846**	16559	6,17	89,85	3	20,40							1,11	0,16	0,49	0,63	1413	**0,96**	1,02	0,04	0,06	0,95	1960	**1,00**	1,07	2,00	0,10	0,06
**PA4875**	10938	5,02	55,58	2	25,50							0,83	-0,26	-1,07	0,28	730	**0,84**	0,85	-0,24	-0,86	0,39	878	**0,92**	0,84	2,00	-0,25	0,01
**PA4876**	13950	8,84	96,61	3	21,33	0,92	-0,13	-0,52	0,60	1328	**0,99**							0,72	-0,48	-1,69	0,09	376	**0,50**	0,82	2,00	-0,29	0,14
**PA4878**	32204	6,42	94,81	3	14,07	1,71	0,77	2,63	0,01	184	**0,10**	0,94	-0,09	-0,43	0,67	1489	**0,97**							1,32	2,00	0,41	0,54
**PA4945**	37234	7,12	122,83	4	11,98	1,25	0,32	1,06	0,29	730	**0,86**							1,04	0,05	0,12	0,91	1865	**1,00**	1,14	2,00	0,19	0,15
**PA4955**	57406	9,74	98,70	2	8,90	1,20	0,27	0,86	0,39	915	**0,93**	1,32	0,41	1,41	0,16	525	**0,65**							1,26	2,00	0,34	0,09
**PA4975**	27445	5,69	133,65	4	9,73							1,94	0,96	3,47	0,00	144	**0,01**	2,49	1,32	4,40	0,00	99	**0,00**	2,21	2,00	1,15	0,39
**PA4988**	47454	9,01	86,41	3	12,70							0,97	-0,04	-0,25	0,80	1765	**0,98**	1,04	0,06	0,13	0,89	1838	**1,00**	1,01	2,00	0,01	0,05
**PA5008**	31341	10,06	52,98	3	18,00	1,03	0,05	0,09	0,93	2013	**1,00**	1,12	0,17	0,53	0,60	1325	**0,97**							1,08	2,00	0,11	0,06
**PA5023**	56230	5,48	52,23	2	7,20	1,18	0,24	0,75	0,45	1018	**0,96**	1,18	0,24	0,78	0,43	998	**0,94**							1,18	2,00	0,24	0,00
**PA5035**	56988	6,10	35,21	2	8,20							0,66	-0,60	-2,33	0,02	266	**0,16**	0,53	-0,92	-3,16	0,00	157	**0,02**	0,60	2,00	-0,75	0,09
**PA5079**	16431	5,90	127,45	5	40,68	0,93	-0,11	-0,46	0,64	1420	**0,98**							1,02	0,03	0,04	0,97	1987	**1,00**	0,97	2,00	-0,04	0,07
**PA5108**	10417	8,39	91,50	3	41,20	0,86	-0,22	-0,87	0,38	904	**0,93**							0,98	-0,03	-0,16	0,87	1801	**1,00**	0,92	2,00	-0,12	0,09
**PA5111**	21060	5,00	139,34	3	11,00							0,99	-0,01	-0,13	0,90	1977	**0,98**	0,98	-0,03	-0,17	0,86	1779	**1,00**	0,99	2,00	-0,02	0,01
**PA5121**	84097	9,90	149,60	4	6,90	0,84	-0,25	-0,95	0,34	816	**0,91**							0,94	-0,08	-0,34	0,73	1506	**1,00**	0,89	2,00	-0,16	0,07
**PA5123**	21144	6,42	39,09	2	10,75							0,68	-0,55	-2,16	0,03	296	**0,23**	0,92	-0,12	-0,45	0,65	1341	**1,00**	0,80	2,00	-0,32	0,17
**PA5127**	18063	8,42	53,81	2	5,90	0,97	-0,04	-0,22	0,83	1811	**0,99**	0,99	-0,02	-0,16	0,87	1921	**0,98**							0,98	2,00	-0,03	0,01
**PA5129**	10235	6,70	49,90	2	10,10							1,09	0,12	0,34	0,73	1624	**0,97**	1,18	0,24	0,74	0,46	1015	**0,93**	1,13	2,00	0,18	0,06
**PA5148**	11817	6,10	158,84	4	30,00							0,84	-0,25	-1,02	0,31	774	**0,85**	0,90	-0,16	-0,59	0,56	1187	**0,96**	0,87	2,00	-0,20	0,04
**PA5154**	26157	9,65	127,21	3	19,90	1,58	0,66	2,24	0,03	248	**0,22**	1,87	0,90	3,27	0,00	157	**0,01**							1,73	2,00	0,79	0,21
**PA5158**	54388	6,41	60,57	2	5,30							1,30	0,38	1,33	0,18	558	**0,71**	1,00	0,00	-0,07	0,95	1954	**1,00**	1,15	2,00	0,20	0,22
**PA5197**	35027	9,35	97,92	3	7,43	1,32	0,40	1,31	0,19	558	**0,74**							0,90	-0,14	-0,55	0,58	1227	**0,98**	1,11	2,00	0,15	0,29
**PA5208**	26682	5,37	59,39	2	12,20	1,00	0,00	-0,10	0,92	2002	**1,00**	0,87	-0,20	-0,83	0,41	947	**0,93**							0,94	2,00	-0,10	0,09
**PA5248**	69950	5,14	63,19	2	2,70	1,34	0,42	1,40	0,16	512	**0,69**	0,95	-0,08	-0,39	0,70	1554	**0,97**							1,14	2,00	0,19	0,28
**PA5259**	27846	5,02	86,00	2	11,20	1,37	0,46	1,53	0,13	454	**0,61**							1,01	0,01	-0,02	0,98	2033	**1,00**	1,19	2,00	0,25	0,26
**PA5269**	10523	4,18	99,32	2	26,40	1,26	0,33	1,09	0,27	704	**0,84**	0,76	-0,40	-1,58	0,11	464	**0,53**							1,01	2,00	0,01	0,35
**PA5285**	12746	4,13	39,71	2	21,00	1,17	0,22	0,69	0,49	1093	**0,97**	1,03	0,04	0,05	0,96	2090	**0,99**							1,10	2,00	0,13	0,10
**PA5303**	13633	4,74	112,22	4	17,50							1,60	0,67	2,42	0,02	251	**0,13**	1,93	0,95	3,15	0,00	158	**0,02**	1,76	2,00	0,82	0,23
**PA5361**	52009	6,99	61,09	2	2,70	0,95	-0,07	-0,32	0,75	1641	**0,99**							1,01	0,01	-0,02	0,99	2041	**0,99**	0,98	2,00	-0,03	0,04
**PA5374**	22758	8,79	81,79	3	15,23							0,92	-0,11	-0,52	0,60	1339	**0,97**	0,72	-0,47	-1,64	0,10	397	**0,52**	0,82	2,00	-0,28	0,14
**PA5412**	94827	4,99	34,77	2	1,15	1,15	0,21	0,64	0,52	1141	**0,99**	0,88	-0,19	-0,81	0,42	965	**0,94**							1,01	2,00	0,02	0,20
**PA5430**	43219	10,63	48,83	2	4,70	1,02	0,03	0,03	0,97	2130	**0,99**							1,36	0,44	1,43	0,15	506	**0,62**	1,19	2,00	0,25	0,24
**PA5437**	36678	7,72	48,06	2	4,80	0,91	-0,13	-0,54	0,59	1293	**0,99**							0,91	-0,14	-0,53	0,60	1242	**0,99**	0,91	2,00	-0,13	0,00
**PA5440**	53871	6,12	100,51	4	7,93	0,95	-0,08	-0,36	0,72	1588	**0,99**	0,99	-0,02	-0,15	0,88	1932	**0,98**							0,97	2,00	-0,05	0,03
**PA5457**	32773	5,84	135,20	4	11,15	1,44	0,53	1,77	0,08	348	**0,48**	1,05	0,07	0,18	0,86	1895	**0,98**							1,25	2,00	0,32	0,28
**PA5475**	21551	5,02	36,33	2	11,55	0,72	-0,47	-1,72	0,09	369	**0,51**	0,87	-0,20	-0,83	0,41	941	**0,93**							0,80	2,00	-0,33	0,11
**PA5484**	67492	5,47	75,78	2	3,50							0,94	-0,08	-0,41	0,68	1521	**0,97**	1,03	0,05	0,10	0,92	1886	**1,00**	0,99	2,00	-0,02	0,06

**PA5498**	35134	5,78	222,95	6	15,73	1,00	0,00	-0,09	0,93	2012	**1,00**	0,95	-0,07	-0,37	0,71	1586	**0,97**							0,97	2,00	-0,04	0,03
**PA5502**	29208	6,52	145,23	5	16,58	1,04	0,06	0,14	0,89	1954	**0,99**							0,99	-0,02	-0,13	0,90	1843	**1,00**	1,01	2,00	0,02	0,04
**PA5518**	61388	5,75	136,60	4	5,73	1,00	0,00	-0,08	0,93	2033	**1,00**	1,12	0,17	0,53	0,60	1331	**0,97**							1,06	2,00	0,09	0,09
**PA5522**	51276	5,50	104,16	3	11,60	1,08	0,11	0,31	0,76	1664	**0,99**	1,12	0,17	0,53	0,60	1335	**0,97**							1,10	2,00	0,14	0,03
**PA5545**	36489	6,98	85,20	2	8,20	1,68	0,75	2,55	0,01	195	**0,12**							1,95	0,96	3,21	0,00	153	**0,02**	1,82	2,00	0,86	0,19
**PA5550**	29156	5,70	79,91	3	14,50	0,84	-0,25	-0,95	0,34	824	**0,91**	1,07	0,10	0,28	0,78	1718	**0,98**							0,96	2,00	-0,06	0,16
**PA0012**	10302	5,52	57,28	2	47,70	1,29	0,37	1,23	0,22	611	**0,78**													1,29	1,00	0,37	ND
**PA0022**	20929	6,05	123,02	3	11,13	0,92	-0,13	-0,52	0,60	1324	**0,99**													0,92	1,00	-0,13	ND
**PA0065**	25696	5,71	72,05	3	13,40	0,94	-0,08	-0,37	0,71	1567	**0,98**													0,94	1,00	-0,08	ND
**PA0072**	43031	9,42	32,38	2	4,65	1,03	0,04	0,06	0,95	2067	**1,00**													1,03	1,00	0,04	ND
**PA0114**	24682	7,79	104,76	3	20,53	0,99	-0,01	-0,13	0,89	1960	**0,99**													0,99	1,00	-0,01	ND
**PA0133**	34407	6,42	54,44	2	6,90	1,07	0,10	0,29	0,78	1705	**0,99**													1,07	1,00	0,10	ND
**PA0159**	35324	5,70	147,77	4	11,05	0,98	-0,04	-0,21	0,84	1838	**0,99**													0,98	1,00	-0,04	ND
**PA0163**	30520	9,02	30,61	2	6,20							1,38	0,46	1,62	0,11	448	**0,51**							1,38	1,00	0,46	ND
**PA0164**	59937	6,09	32,03	2	4,30	0,71	-0,49	-1,81	0,07	335	**0,45**													0,71	1,00	-0,49	ND
**PA0223**	33067	5,33	58,67	2	8,20							0,82	-0,29	-1,16	0,25	656	**0,81**							0,82	1,00	-0,29	ND
**PA0230**	50041	5,87	108,78	3	10,00							0,93	-0,11	-0,49	0,62	1399	**0,96**							0,93	1,00	-0,11	ND
**PA0231**	28538	5,20	55,05	2	7,75							0,97	-0,04	-0,25	0,80	1775	**0,98**							0,97	1,00	-0,04	ND
**PA0259**	55052	5,21	71,04	2	6,30	1,00	0,00	-0,08	0,94	2041	**1,00**													1,00	1,00	0,00	ND
**PA0261**	19376	8,28	35,06	2	32,10							0,66	-0,60	-2,35	0,02	262	**0,15**							0,66	1,00	-0,60	ND
**PA0294**	24933	5,98	44,55	2	6,35							1,07	0,10	0,27	0,79	1736	**0,98**							1,07	1,00	0,10	ND
**PA0303**	33872	5,15	119,71	2	5,10	1,30	0,38	1,25	0,21	591	**0,77**													1,30	1,00	0,38	ND
**PA0304**	33056	9,33	67,46	2	11,10	0,98	-0,02	-0,16	0,87	1915	**0,99**													0,98	1,00	-0,02	ND
**PA0365**	20646	6,84	31,34	2	6,85							1,01	0,02	-0,03	0,98	2126	**0,99**							1,01	1,00	0,02	ND
**PA0369**	10831	4,00	60,01	2	16,70							1,13	0,18	0,58	0,56	1243	**0,97**							1,13	1,00	0,18	ND
**PA0404**	16856	5,74	50,35	2	26,40							1,20	0,26	0,86	0,39	903	**0,93**							1,20	1,00	0,26	ND
**PA0405**	21012	4,86	94,29	3	11,60	1,02	0,03	0,04	0,97	2107	**1,00**													1,02	1,00	0,03	ND
**PA0535**	20887	5,15	50,53	2	8,20	0,98	-0,03	-0,20	0,84	1849	**0,99**													0,98	1,00	-0,03	ND
**PA0547**	37175	5,60	124,63	3	6,80	1,01	0,01	-0,04	0,97	2105	**1,00**													1,01	1,00	0,01	ND
**PA0572**	105087	6,13	34,72	2	2,75	0,91	-0,14	-0,58	0,56	1234	**0,99**													0,91	1,00	-0,14	ND
**PA0575**	144500	5,46	32,52	2	2,20	1,25	0,32	1,05	0,29	735	**0,87**													1,25	1,00	0,32	ND
**PA0599**	40550	5,09	48,58	2	6,20							1,07	0,10	0,27	0,78	1730	**0,98**							1,07	1,00	0,10	ND
**PA0602**	41261	6,92	64,96	2	12,20	1,37	0,45	1,50	0,13	459	**0,63**													1,37	1,00	0,45	ND
**PA0606**	30259	9,72	111,01	2	5,40	0,73	-0,45	-1,66	0,10	394	**0,54**													0,73	1,00	-0,45	ND
**PA0608**	31615	5,05	193,32	4	19,58	0,96	-0,06	-0,28	0,78	1718	**0,98**													0,96	1,00	-0,06	ND
**PA0624**	12994	4,62	66,53	2	25,40							0,50	-0,99	-3,78	0,00	128	**0,00**							0,50	1,00	-0,99	ND
**PA0657**	57440	5,81	106,52	4	6,50							0,92	-0,12	-0,53	0,60	1327	**0,97**							0,92	1,00	-0,12	ND
**PA0716**	53523	5,15	65,43	2	11,30							1,60	0,68	2,43	0,01	249	**0,13**							1,60	1,00	0,68	ND
**PA0735**	31955	9,72	52,18	2	10,00							0,91	-0,13	-0,58	0,56	1259	**0,97**							0,91	1,00	-0,13	ND
**PA0793**	43596	5,44	85,57	3	8,40	1,18	0,24	0,75	0,45	1020	**0,96**													1,18	1,00	0,24	ND
**PA0801**	57356	9,48	114,66	3	5,40							1,70	0,77	2,75	0,01	205	**0,06**							1,70	1,00	0,77	ND
**PA0830**	34677	9,37	103,11	2	15,20							1,39	0,47	1,66	0,10	431	**0,49**							1,39	1,00	0,47	ND
**PA0859**	21735	4,87	179,59	3	13,50	1,02	0,03	0,04	0,97	2119	**1,00**													1,02	1,00	0,03	ND
**PA0864**	29237	8,93	33,39	2	4,60							1,52	0,61	2,16	0,03	294	**0,23**							1,52	1,00	0,61	ND
**PA0927**	36449	5,74	154,44	3	15,80							1,44	0,53	1,86	0,06	371	**0,37**							1,44	1,00	0,53	ND
**PA0948**	16225	10,01	69,75	3	9,40	1,52	0,60	2,04	0,04	273	**0,33**													1,52	1,00	0,60	ND
**PA0960**	8150	4,16	62,86	2	60,90	0,97	-0,04	-0,22	0,83	1816	**0,99**													0,97	1,00	-0,04	ND
**PA0965**	19547	9,93	56,54	2	6,90							0,98	-0,03	-0,21	0,83	1838	**0,98**							0,98	1,00	-0,03	ND

**PA0989**	23098	9,19	108,09	3	13,97	0,90	-0,15	-0,62	0,54	1173	**0,99**							0,90	1,00	-0,15	ND
**PA0998**	40121	5,09	72,34	2	10,30							0,45	-1,14	-4,35	0,00	105	**0,00**	0,45	1,00	-1,14	ND
**PA1006**	9949	5,74	30,82	2	17,45							1,00	0,00	-0,08	0,93	2029	**0,99**	1,00	1,00	0,00	ND
**PA1024**	35892	6,20	63,15	3	7,20							1,03	0,05	0,08	0,93	2028	**0,99**	1,03	1,00	0,05	ND
**PA1026**	18373	5,05	75,19	2	13,90	1,14	0,19	0,58	0,56	1247	**0,98**							1,14	1,00	0,19	ND
**PA1039**	18313	6,21	38,79	2	8,90							1,07	0,10	0,27	0,78	1731	**0,98**	1,07	1,00	0,10	ND
**PA1056**	53467	9,51	66,88	2	3,20							0,95	-0,07	-0,35	0,72	1611	**0,97**	0,95	1,00	-0,07	ND
**PA1062**	19524	6,06	108,52	2	13,90							1,44	0,52	1,86	0,06	372	**0,37**	1,44	1,00	0,52	ND
**PA1079**	27141	5,48	75,40	2	12,70	1,01	0,01	-0,05	0,96	2092	**1,00**							1,01	1,00	0,01	ND
**PA1110**	28176	8,48	36,99	2	11,00							1,06	0,08	0,20	0,84	1853	**0,98**	1,06	1,00	0,08	ND
**PA1123**	12638	4,90	58,80	2	18,90							1,01	0,01	-0,07	0,95	2061	**0,99**	1,01	1,00	0,01	ND
**PA1128**	34190	5,90	38,50	2	7,60	1,05	0,07	0,17	0,87	1907	**0,99**							1,05	1,00	0,07	ND
**PA1142**	27770	8,61	42,78	2	8,40	1,39	0,47	1,57	0,12	430	**0,59**							1,39	1,00	0,47	ND
**PA1164**	30787	8,86	63,73	2	8,90	1,05	0,07	0,17	0,87	1908	**0,99**							1,05	1,00	0,07	ND
**PA1247**	49608	7,96	34,62	2	4,30	0,69	-0,54	-1,97	0,05	283	**0,38**							0,69	1,00	-0,54	ND
**PA1269**	26113	5,69	65,54	2	11,70	1,18	0,24	0,77	0,44	1007	**0,95**							1,18	1,00	0,24	ND
**PA1318**	77383	8,86	54,58	2	4,75	0,31	-1,67	-5,94	0,00	54	**0,00**							0,31	1,00	-1,67	ND
**PA1326**	58261	6,08	73,93	2	13,40							0,98	-0,03	-0,21	0,83	1830	**0,98**	0,98	1,00	-0,03	ND
**PA1343**	17408	7,98	84,02	2	9,20	0,96	-0,06	-0,27	0,78	1731	**0,98**							0,96	1,00	-0,06	ND
**PA1397**	23706	5,36	115,92	3	9,33	0,83	-0,28	-1,05	0,29	737	**0,87**							0,83	1,00	-0,28	ND
**PA1453**	49011	9,53	71,59	2	5,15							1,02	0,03	0,01	1,00	2154	**1,00**	1,02	1,00	0,03	ND
**PA1460**	27096	5,03	47,97	2	8,15	1,32	0,41	1,34	0,18	536	**0,73**							1,32	1,00	0,41	ND
**PA1470**	26070	5,14	67,10	2	6,10							1,10	0,13	0,40	0,69	1535	**0,97**	1,10	1,00	0,13	ND
**PA1495**	23855	5,64	67,86	2	15,90							1,18	0,24	0,78	0,43	1000	**0,94**	1,18	1,00	0,24	ND
**PA1549**	89395	6,14	80,18	3	3,37							1,62	0,69	2,49	0,01	234	**0,12**	1,62	1,00	0,69	ND
**PA1557**	55021	9,07	141,42	3	8,93							1,15	0,21	0,67	0,50	1127	**0,96**	1,15	1,00	0,21	ND
**PA1570**	33121	5,64	37,12	2	5,65	1,17	0,23	0,73	0,46	1037	**0,97**							1,17	1,00	0,23	ND
**PA1594**	17451	7,01	43,92	2	14,55	1,38	0,47	1,56	0,12	436	**0,59**							1,38	1,00	0,47	ND
**PA1630**	34252	7,26	29,55	2	4,70							0,69	-0,54	-2,12	0,03	302	**0,24**	0,69	1,00	-0,54	ND
**PA1633**	61757	7,66	87,66	2	2,10							2,58	1,37	5,01	0,00	83	**0,00**	2,58	1,00	1,37	ND
**PA1665**	45047	5,27	28,43	2	6,30							0,76	-0,39	-1,55	0,12	477	**0,55**	0,76	1,00	-0,39	ND
**PA1707**	19053	4,59	64,45	2	10,50							0,48	-1,06	-4,05	0,00	114	**0,00**	0,48	1,00	-1,06	ND
**PA1709**	33308	5,83	54,78	2	3,90							0,71	-0,50	-1,96	0,05	334	**0,32**	0,71	1,00	-0,50	ND
**PA1753**	18937	4,92	110,55	2	10,90	0,98	-0,03	-0,20	0,84	1852	**0,99**							0,98	1,00	-0,03	ND
**PA1759**	104201	5,97	42,14	2	2,45							2,30	1,20	4,38	0,00	103	**0,00**	2,30	1,00	1,20	ND
**PA1773**	38557	5,28	31,62	2	8,10	1,11	0,15	0,45	0,65	1437	**0,98**							1,11	1,00	0,15	ND
**PA1859**	33567	6,42	42,50	2	3,55	1,36	0,45	1,49	0,14	464	**0,64**							1,36	1,00	0,45	ND
**PA1880**	83078	6,86	37,81	2	3,35							0,99	-0,01	-0,14	0,89	1959	**0,98**	0,99	1,00	-0,01	ND
**PA1961**	36085	7,07	83,12	2	7,25							1,08	0,11	0,31	0,76	1668	**0,98**	1,08	1,00	0,11	ND
**PA1986**	33988	5,42	29,39	2	8,20	1,24	0,31	0,99	0,32	782	**0,89**							1,24	1,00	0,31	ND
**PA1988**	10881	4,72	77,01	2	11,95							0,88	-0,18	-0,78	0,44	1007	**0,94**	0,88	1,00	-0,18	ND
**PA2034**	25172	5,99	79,57	3	13,50	1,67	0,74	2,52	0,01	202	**0,13**							1,67	1,00	0,74	ND
**PA2049**	55488	7,07	64,96	2	2,80	1,40	0,49	1,64	0,10	400	**0,55**							1,40	1,00	0,49	ND
**PA2063**	48506	9,56	87,97	4	11,28	1,17	0,23	0,72	0,47	1062	**0,97**							1,17	1,00	0,23	ND
**PA2126**	24429	7,12	129,05	3	7,60							1,50	0,59	2,08	0,04	310	**0,26**	1,50	1,00	0,59	ND
**PA2196**	23260	6,08	120,11	3	14,43							1,13	0,18	0,57	0,57	1287	**0,96**	1,13	1,00	0,18	ND
**PA2240**	54237	10,81	101,52	2	3,10	1,68	0,74	2,53	0,01	198	**0,12**							1,68	1,00	0,74	ND
**PA2280**	27419	6,85	50,92	2	12,60							0,87	-0,21	-0,87	0,38	889	**0,93**	0,87	1,00	-0,21	ND

**PA2282**	17028	5,20	59,24	2	15,50	0,79	-0,35	-1,31	0,19	563	**0,74**							0,79	1,00	-0,35	ND
**PA2318**	13890	4,72	127,42	3	34,73							0,86	-0,22	-0,93	0,35	841	**0,90**	0,86	1,00	-0,22	ND
**PA2321**	19349	6,58	101,82	2	30,10							1,29	0,37	1,27	0,20	595	**0,74**	1,29	1,00	0,37	ND
**PA2342**	55935	5,88	35,94	2	2,45							0,97	-0,04	-0,24	0,81	1799	**0,98**	0,97	1,00	-0,04	ND
**PA2394**	50350	6,80	74,07	2	14,10	1,51	0,59	2,00	0,05	278	**0,35**							1,51	1,00	0,59	ND
**PA2397**	63891	6,88	79,44	2	3,40	1,46	0,55	1,85	0,06	314	**0,44**							1,46	1,00	0,55	ND
**PA2407**	36738	9,20	106,22	3	15,80	2,11	1,08	3,71	0,00	108	**0,00**							2,11	1,00	1,08	ND
**PA2463**	64953	8,39	65,77	3	11,63							1,37	0,45	1,59	0,11	455	**0,53**	1,37	1,00	0,45	ND
**PA2502**	55177	6,05	54,88	2	5,20	1,32	0,40	1,32	0,19	552	**0,73**							1,32	1,00	0,40	ND
**PA2526**	115304	5,87	49,02	2	2,55							1,42	0,50	1,78	0,08	395	**0,41**	1,42	1,00	0,50	ND
**PA2527**	115749	5,27	110,78	2	3,70							1,49	0,58	2,05	0,04	313	**0,28**	1,49	1,00	0,58	ND
**PA2536**	36315	9,11	43,51	2	8,00	1,23	0,30	0,98	0,33	789	**0,90**							1,23	1,00	0,30	ND
**PA2557**	64877	5,80	34,91	2	7,80	1,85	0,88	3,02	0,00	153	**0,04**							1,85	1,00	0,88	ND
**PA2559**	20242	5,89	58,45	2	8,35	0,66	-0,60	-2,18	0,03	253	**0,25**							0,66	1,00	-0,60	ND
**PA2617**	26570	7,05	45,51	2	10,65	1,11	0,15	0,45	0,65	1439	**0,98**							1,11	1,00	0,15	ND
**PA2625**	17750	5,29	115,19	3	16,27							0,85	-0,23	-0,95	0,34	831	**0,89**	0,85	1,00	-0,23	ND
**PA2649**	53219	7,79	84,46	3	3,30	0,99	-0,02	-0,15	0,88	1941	**0,99**							0,99	1,00	-0,02	ND
**PA2650**	30734	5,56	64,74	2	9,30							0,86	-0,21	-0,88	0,38	878	**0,93**	0,86	1,00	-0,21	ND
**PA2656**	51714	6,36	63,75	2	6,85	1,09	0,13	0,37	0,71	1576	**0,98**							1,09	1,00	0,13	ND
**PA2685**	85010	5,90	119,55	5	7,98	0,82	-0,28	-1,07	0,28	715	**0,86**							0,82	1,00	-0,28	ND
**PA2802**	27939	6,02	58,12	2	9,45							0,72	-0,48	-1,89	0,06	356	**0,35**	0,72	1,00	-0,48	ND
**PA2852**	43894	5,36	44,99	2	3,75							1,44	0,52	1,85	0,06	373	**0,37**	1,44	1,00	0,52	ND
**PA2877**	34344	6,81	64,55	2	6,90							1,01	0,01	-0,06	0,95	2072	**0,99**	1,01	1,00	0,01	ND
**PA2932**	39941	5,58	90,42	2	7,90							0,42	-1,24	-4,72	0,00	92	**0,00**	0,42	1,00	-1,24	ND
**PA2964**	30674	5,62	34,40	2	11,25							1,00	0,00	-0,10	0,92	2020	**0,99**	1,00	1,00	0,00	ND
**PA2969**	36261	7,71	61,63	3	13,80	1,49	0,57	1,93	0,05	295	**0,40**							1,49	1,00	0,57	ND
**PA2981**	37628	6,77	57,54	2	7,50	1,05	0,07	0,15	0,88	1933	**0,99**							1,05	1,00	0,07	ND
**PA2996**	25650	9,48	95,04	3	6,70	0,94	-0,09	-0,41	0,68	1502	**0,99**							0,94	1,00	-0,09	ND
**PA2998**	45428	6,75	66,73	2	2,50							1,17	0,22	0,73	0,47	1056	**0,96**	1,17	1,00	0,22	ND
**PA3030**	22473	7,05	70,04	3	8,43							0,93	-0,11	-0,50	0,62	1385	**0,96**	0,93	1,00	-0,11	ND
**PA3052**	37913	9,25	114,26	4	9,35	0,92	-0,12	-0,49	0,62	1375	**0,99**							0,92	1,00	-0,12	ND
**PA3062**	19333	5,33	33,86	2	7,55	1,38	0,47	1,56	0,12	432	**0,59**							1,38	1,00	0,47	ND
**PA3073**	38579	7,98	44,57	2	12,40	1,30	0,38	1,25	0,21	600	**0,77**							1,30	1,00	0,38	ND
**PA3076**	41662	8,33	61,97	2	12,30	1,02	0,03	0,04	0,97	2111	**1,00**							1,02	1,00	0,03	ND
**PA3079**	92897	6,66	42,78	2	3,20							1,12	0,16	0,51	0,61	1363	**0,96**	1,12	1,00	0,16	ND
**PA3085**	10916	4,86	107,41	2	29,90							0,75	-0,42	-1,68	0,09	426	**0,47**	0,75	1,00	-0,42	ND
**PA3087**	38426	5,92	55,88	2	8,00							0,88	-0,18	-0,76	0,44	1018	**0,94**	0,88	1,00	-0,18	ND
**PA3123**	13842	4,85	35,63	2	24,00	1,00	0,00	-0,09	0,93	2028	**1,00**							1,00	1,00	0,00	ND
**PA3127**	29765	5,19	64,67	2	12,00							1,04	0,05	0,10	0,92	2018	**0,99**	1,04	1,00	0,05	ND
**PA3129**	36749	7,74	39,25	2	6,00							0,84	-0,26	-1,07	0,29	736	**0,84**	0,84	1,00	-0,26	ND
**PA3130**	17069	5,35	51,57	2	12,40	0,94	-0,09	-0,41	0,68	1510	**0,99**							0,94	1,00	-0,09	ND
**PA3133**	20362	5,97	92,47	3	15,00	1,02	0,03	0,04	0,97	2121	**0,99**							1,02	1,00	0,03	ND
**PA3157**	72300	9,24	37,71	2	4,30	1,25	0,32	1,06	0,29	731	**0,87**							1,25	1,00	0,32	ND
**PA3161**	11778	9,52	75,82	3	29,10							0,96	-0,06	-0,30	0,76	1679	**0,98**	0,96	1,00	-0,06	ND
**PA3192**	28223	5,63	35,90	2	11,60	1,45	0,53	1,79	0,07	341	**0,47**							1,45	1,00	0,53	ND
**PA3196**	20381	4,19	53,92	2	9,10							1,00	0,01	-0,08	0,94	2046	**0,99**	1,00	1,00	0,01	ND
**PA3200**	33630	6,31	33,74	2	3,40	1,04	0,05	0,10	0,92	1996	**1,00**							1,04	1,00	0,05	ND
**PA3202**	11323	6,07	31,02	2	8,10	0,83	-0,26	-1,00	0,32	773	**0,89**							0,83	1,00	-0,26	ND

**PA3217**	54863	9,20	81,22	3	10,80	0,82	-0,29	-1,10	0,27	703	**0,84**							0,82	1,00	-0,29	ND
**PA3259**	18282	10,43	56,03	3	19,50	0,78	-0,35	-1,31	0,19	561	**0,74**							0,78	1,00	-0,35	ND
**PA3301**	36578	6,76	86,37	3	10,40	0,92	-0,12	-0,50	0,62	1356	**0,99**							0,92	1,00	-0,12	ND
**PA3304**	32525	8,89	46,92	2	8,60							1,33	0,41	1,43	0,15	522	**0,64**	1,33	1,00	0,41	ND
**PA3317**	26975	6,64	57,20	2	7,25	1,05	0,07	0,17	0,86	1896	**0,99**							1,05	1,00	0,07	ND
**PA3330**	33303	8,84	88,05	2	5,10							0,83	-0,27	-1,12	0,26	693	**0,82**	0,83	1,00	-0,27	ND
**PA3339**	84740	5,32	108,63	2	6,20							1,14	0,18	0,59	0,56	1234	**0,97**	1,14	1,00	0,18	ND
**PA3347**	11697	5,56	68,00	3	12,90	0,54	-0,88	-3,15	0,00	139	**0,03**							0,54	1,00	-0,88	ND
**PA3354**	22810	5,54	46,69	2	9,90	1,08	0,11	0,29	0,77	1699	**0,99**							1,08	1,00	0,11	ND
**PA3365**	43631	8,63	52,81	2	8,25	3,64	1,87	6,47	0,00	40	**0,00**							3,64	1,00	1,87	ND
**PA3394**	34090	8,98	41,05	2	5,10							1,62	0,69	2,48	0,01	238	**0,12**	1,62	1,00	0,69	ND
**PA3398**	36094	6,52	63,29	2	8,90	0,74	-0,44	-1,61	0,11	414	**0,56**							0,74	1,00	-0,44	ND
**PA3408**	101066	6,08	32,58	2	2,95	1,21	0,27	0,87	0,38	900	**0,93**							1,21	1,00	0,27	ND
**PA3433**	33567	5,73	83,35	2	12,50							0,83	-0,28	-1,13	0,26	685	**0,82**	0,83	1,00	-0,28	ND
**PA3454**	44455	5,30	35,32	2	6,90	1,04	0,05	0,10	0,92	1997	**1,00**							1,04	1,00	0,05	ND
**PA3458**	18309	5,59	85,65	3	17,80							0,90	-0,15	-0,65	0,52	1160	**0,96**	0,90	1,00	-0,15	ND
**PA3465**	63533	9,20	77,34	3	5,57							0,88	-0,18	-0,77	0,44	1017	**0,94**	0,88	1,00	-0,18	ND
**PA3491**	88036	6,44	38,90	2	5,00							0,91	-0,13	-0,59	0,56	1239	**0,97**	0,91	1,00	-0,13	ND
**PA3495**	26258	8,76	49,30	2	18,90							0,93	-0,10	-0,48	0,63	1418	**0,96**	0,93	1,00	-0,10	ND
**PA3575**	20130	10,15	57,19	2	9,10	0,78	-0,35	-1,31	0,19	560	**0,74**							0,78	1,00	-0,35	ND
**PA3580**	18356	9,52	45,01	2	12,20	0,68	-0,56	-2,05	0,04	271	**0,32**							0,68	1,00	-0,56	ND
**PA3628**	32508	5,84	112,22	3	13,40							0,74	-0,43	-1,70	0,09	419	**0,46**	0,74	1,00	-0,43	ND
**PA3658**	107455	5,43	43,49	2	2,45							1,03	0,04	0,04	0,97	2106	**0,99**	1,03	1,00	0,04	ND
**PA3677**	41299	9,46	63,83	2	5,70	1,41	0,50	1,67	0,10	391	**0,53**							1,41	1,00	0,50	ND
**PA3680**	29852	8,88	100,29	3	18,80	1,22	0,28	0,91	0,36	870	**0,91**							1,22	1,00	0,28	ND
**PA3721**	24521	5,56	87,64	3	16,13							1,14	0,19	0,62	0,54	1206	**0,96**	1,14	1,00	0,19	ND
**PA3758**	39757	6,01	48,37	2	8,30	0,90	-0,16	-0,64	0,52	1149	**0,99**							0,90	1,00	-0,16	ND
**PA3767**	21184	9,12	51,48	2	9,60	1,35	0,44	1,45	0,15	486	**0,66**							1,35	1,00	0,44	ND
**PA3777**	52780	10,86	53,27	2	12,00	0,96	-0,06	-0,29	0,77	1692	**0,99**							0,96	1,00	-0,06	ND
**PA3793**	12642	4,09	101,72	2	27,30							1,23	0,30	1,01	0,31	784	**0,86**	1,23	1,00	0,30	ND
**PA3846**	20548	5,35	60,18	2	18,90							1,18	0,24	0,80	0,42	975	**0,94**	1,18	1,00	0,24	ND
**PA3863**	41538	5,29	100,84	2	7,05							0,89	-0,17	-0,74	0,46	1039	**0,95**	0,89	1,00	-0,17	ND
**PA3864**	25327	5,71	131,15	3	15,57							0,81	-0,31	-1,24	0,22	616	**0,76**	0,81	1,00	-0,31	ND
**PA3902**	18283	6,29	71,59	3	22,20	0,82	-0,29	-1,10	0,27	697	**0,84**							0,82	1,00	-0,29	ND
**PA3924**	65774	5,75	58,30	2	5,40							1,18	0,24	0,80	0,43	978	**0,94**	1,18	1,00	0,24	ND
**PA4006**	24455	5,97	89,95	3	18,67	1,07	0,10	0,26	0,79	1743	**0,99**							1,07	1,00	0,10	ND
**PA4059**	14413	10,02	116,01	3	26,13							0,99	-0,01	-0,14	0,89	1963	**0,98**	0,99	1,00	-0,01	ND
**PA4110**	45772	8,65	48,82	2	4,15	0,83	-0,27	-1,04	0,30	744	**0,87**							0,83	1,00	-0,27	ND
**PA4112**	157306	5,48	30,11	2	3,05	1,52	0,60	2,04	0,04	274	**0,33**							1,52	1,00	0,60	ND
**PA4181**	26609	5,13	132,76	2	9,20							0,74	-0,44	-1,73	0,08	410	**0,44**	0,74	1,00	-0,44	ND
**PA4197**	83704	6,43	48,72	2	2,80	0,89	-0,17	-0,67	0,50	1118	**0,98**							0,89	1,00	-0,17	ND
**PA4226**	158247	5,27	57,21	2	3,20							1,43	0,52	1,83	0,07	382	**0,38**	1,43	1,00	0,52	ND
**PA4242**	5434	11,33	41,36	2	21,10	1,09	0,13	0,38	0,71	1561	**0,98**							1,09	1,00	0,13	ND
**PA4281**	46651	5,34	118,46	2	6,60							1,52	0,61	2,17	0,03	292	**0,22**	1,52	1,00	0,61	ND
**PA4286**	25410	5,37	101,48	2	10,40	1,07	0,09	0,24	0,81	1781	**0,99**							1,07	1,00	0,09	ND
**PA4318**	29498	6,53	134,15	2	15,50							0,73	-0,45	-1,78	0,07	393	**0,41**	0,73	1,00	-0,45	ND
**PA4347**	44005	7,10	35,27	2	3,10							0,93	-0,10	-0,48	0,63	1415	**0,96**	0,93	1,00	-0,10	ND
**PA4375**	114923	5,20	49,14	2	2,80	1,07	0,10	0,25	0,80	1761	**0,99**							1,07	1,00	0,10	ND

**PA4421**	18169	5,13	40,20	2	12,25	1,14	0,19	0,58	0,56	1242	**0,98**							1,14	1,00	0,19	ND
**PA4486**	14796	5,60	116,00	2	9,40							1,11	0,15	0,46	0,64	1440	**0,97**	1,11	1,00	0,15	ND
**PA4535**	25131	9,24	31,92	2	11,25							1,05	0,07	0,16	0,87	1927	**0,98**	1,05	1,00	0,07	ND
**PA4536**	17312	9,58	53,12	2	9,75							0,99	-0,02	-0,17	0,86	1906	**0,98**	0,99	1,00	-0,02	ND
**PA4551**	22341	8,50	29,21	2	9,20	0,56	-0,85	-3,06	0,00	150	**0,03**							0,56	1,00	-0,85	ND
**PA4587**	40734	6,26	69,34	2	11,00							1,54	0,62	2,22	0,03	282	**0,20**	1,54	1,00	0,62	ND
**PA4624**	66212	5,57	43,40	2	5,10	1,34	0,42	1,38	0,17	521	**0,69**							1,34	1,00	0,42	ND
**PA4647**	46739	8,46	70,91	2	4,90							1,11	0,16	0,48	0,63	1416	**0,96**	1,11	1,00	0,16	ND
**PA4656**	35168	8,38	49,47	2	3,80	0,89	-0,17	-0,68	0,50	1111	**0,98**							0,89	1,00	-0,17	ND
**PA4657**	37366	6,35	127,02	3	11,30	0,94	-0,09	-0,39	0,69	1533	**0,98**							0,94	1,00	-0,09	ND
**PA4659**	34699	7,78	50,17	2	4,15	1,06	0,08	0,21	0,83	1820	**0,99**							1,06	1,00	0,08	ND
**PA4683**	23852	5,91	68,13	2	9,50	0,75	-0,41	-1,53	0,13	447	**0,61**							0,75	1,00	-0,41	ND
**PA4693**	30693	6,92	47,96	2	2,60	0,95	-0,07	-0,33	0,74	1635	**0,99**							0,95	1,00	-0,07	ND
**PA4704**	30609	5,31	67,53	3	15,07							0,49	-1,03	-3,92	0,00	122	**0,00**	0,49	1,00	-1,03	ND
**PA4725**	111182	5,63	56,46	2	3,70	0,86	-0,21	-0,83	0,41	942	**0,94**							0,86	1,00	-0,21	ND
**PA4770**	61244	8,94	36,43	2	4,10							0,68	-0,57	-2,21	0,03	284	**0,21**	0,68	1,00	-0,57	ND
**PA4786**	49597	9,40	89,92	2	4,10	0,61	-0,71	-2,56	0,01	192	**0,12**							0,61	1,00	-0,71	ND
**PA4793**	22178	7,79	45,53	2	10,70	0,87	-0,19	-0,76	0,45	1013	**0,96**							0,87	1,00	-0,19	ND
**PA4868**	63632	5,81	36,29	2	6,70							1,25	0,33	1,12	0,26	688	**0,82**	1,25	1,00	0,33	ND
**PA4880**	21116	4,64	80,59	2	17,80	0,71	-0,50	-1,83	0,07	325	**0,45**							0,71	1,00	-0,50	ND
**PA4944**	9962	9,52	45,16	2	34,75	0,60	-0,73	-2,63	0,01	185	**0,10**							0,60	1,00	-0,73	ND
**PA4954**	32184	5,53	31,54	2	9,90	0,88	-0,18	-0,70	0,48	1083	**0,97**							0,88	1,00	-0,18	ND
**PA4977**	60650	6,85	68,62	2	4,70							1,81	0,85	3,09	0,00	173	**0,03**	1,81	1,00	0,85	ND
**PA5020**	69253	5,84	60,54	2	7,40	0,89	-0,17	-0,68	0,50	1112	**0,98**							0,89	1,00	-0,17	ND
**PA5021**	58727	6,01	94,04	3	4,13	0,98	-0,03	-0,18	0,86	1892	**0,99**							0,98	1,00	-0,03	ND
**PA5028**	30683	4,90	59,42	2	16,10	0,77	-0,38	-1,41	0,16	506	**0,68**							0,77	1,00	-0,38	ND
**PA5048**	28653	10,34	88,62	3	18,40							0,98	-0,02	-0,19	0,85	1876	**0,98**	0,98	1,00	-0,02	ND
**PA5062**	11287	7,90	113,80	3	31,90	0,78	-0,35	-1,31	0,19	557	**0,74**							0,78	1,00	-0,35	ND
**PA5082**	35964	7,70	63,28	2	11,40							1,58	0,66	2,35	0,02	263	**0,15**	1,58	1,00	0,66	ND
**PA5091**	30631	5,63	64,60	2	11,30							0,97	-0,05	-0,27	0,78	1729	**0,98**	0,97	1,00	-0,05	ND
**PA5100**	64039	5,95	69,91	3	6,80							0,75	-0,41	-1,62	0,10	445	**0,51**	0,75	1,00	-0,41	ND
**PA5122**	19326	5,91	136,80	3	26,00	0,82	-0,29	-1,11	0,27	690	**0,84**							0,82	1,00	-0,29	ND
**PA5124**	41134	5,77	90,31	2	10,90	1,29	0,36	1,19	0,23	635	**0,80**							1,29	1,00	0,36	ND
**PA5165**	70410	8,97	37,99	2	5,20	1,07	0,10	0,26	0,79	1744	**0,99**							1,07	1,00	0,10	ND
**PA5188**	45975	5,36	41,93	2	5,60	0,99	-0,02	-0,14	0,89	1957	**0,99**							0,99	1,00	-0,02	ND
**PA5226**	13280	9,25	31,67	2	19,40							0,99	-0,01	-0,13	0,90	1975	**0,98**	0,99	1,00	-0,01	ND
**PA5233**	16376	4,74	84,80	2	15,10							1,32	0,40	1,40	0,16	529	**0,66**	1,32	1,00	0,40	ND
**PA5281**	26459	5,68	61,72	2	10,30	1,32	0,40	1,34	0,18	543	**0,73**							1,32	1,00	0,40	ND
**PA5310**	61659	9,40	53,96	2	6,40	0,89	-0,17	-0,67	0,50	1119	**0,98**							0,89	1,00	-0,17	ND
**PA5347**	17167	10,43	58,72	2	19,20							0,61	-0,72	-2,77	0,01	204	**0,06**	0,61	1,00	-0,72	ND
**PA5372**	64137	6,44	52,74	2	6,40	1,12	0,17	0,51	0,61	1349	**0,99**							1,12	1,00	0,17	ND
**PA5439**	57127	6,70	35,25	2	3,70	0,70	-0,52	-1,91	0,06	298	**0,41**							0,70	1,00	-0,52	ND
**PA5449**	54544	6,42	104,07	4	7,05	1,61	0,69	2,34	0,02	227	**0,19**							1,61	1,00	0,69	ND
**PA5453**	38342	6,13	44,24	2	13,00							1,77	0,82	2,96	0,00	183	**0,04**	1,77	1,00	0,82	ND
**PA5455**	62927	5,13	41,03	2	3,30							1,60	0,68	2,42	0,02	250	**0,13**	1,60	1,00	0,68	ND
**PA5464**	77588	6,57	72,93	2	3,15	0,96	-0,07	-0,31	0,76	1666	**0,99**							0,96	1,00	-0,07	ND
**PA5471**	42954	6,19	84,96	3	9,50							1,10	0,14	0,41	0,68	1529	**0,97**	1,10	1,00	0,14	ND
**PA5478**	44445	7,71	56,05	2	7,10	0,83	-0,27	-1,02	0,31	759	**0,88**							0,83	1,00	-0,27	ND

**PA5500**	30664	7,82	61,49	2	10,60							1,23	0,30	1,03	0,30	764	**0,86**	1,23	1,00	0,30	ND
**PA5524**	27954	5,25	34,10	2	8,80							1,13	0,17	0,55	0,58	1303	**0,97**	1,13	1,00	0,17	ND
**PA5525**	28449	5,42	63,67	2	4,25	0,95	-0,07	-0,34	0,73	1614	**0,99**							0,95	1,00	-0,07	ND

Table S3. Complete list of transcriptomic, proteomic and post-transcriptional regulation data for all *Pseudomonas aeruginosa* genes.

ID – Gene [a]	TC [b]	PC [c]	PTV[d]	ID – Gene [a]	TC [b]	PC [c]	PTV[d]	ID – Gene [a]	TC [b]	PC [c]	PTV[d]
PA0001 dnaA	0,08	0,17	0,42	PA1791 -	0,72	0,47	0,63	PA3831 pepA	-0,46	-0,37	-0,59
PA0002 dnaN	-0,14	-0,09	-0,11	PA1792 -	0,48	0,24	0,21	PA3832 holC	-0,20	-0,09	-0,05
PA0003 recF	0,08	-0,22	-0,71	PA1793 ppiB	0,41	0,57	**1,21**	PA3833 -	-0,07	ND	ND
PA0004 gyrB	-0,20	-0,27	-0,56	PA1794 glnS	0,59	0,55	0,99	PA3834 valS	-0,34	-0,26	-0,40
PA0005 lptA	0,32	-0,13	-0,69	PA1795 cysS	0,65	0,47	0,69	PA3835 -	-0,23	ND	ND
PA0006 -	-0,38	0,10	0,65	PA1796 folD	0,74	0,62	**1,04**	PA3836 -	0,12	0,73	**1,94**
PA0007 -	0,40	ND	ND	PA1796.1 -	**1,84**	ND	ND	PA3837 -	0,02	ND	ND
PA0008 glyS	-0,24	-0,16	-0,21	PA1796.2 -	**1,68**	ND	ND	PA3838 -	-0,44	0,49	**1,84**
PA0009 glyQ	-0,16	-0,18	-0,35	PA1796.3 -	**1,26**	ND	ND	PA3839 -	-0,47	ND	ND
PA0010 tag	-0,29	0,06	0,47	PA1796.4 -	**1,35**	ND	ND	PA3840 -	-0,39	-0,33	-0,56
PA0011 -	-0,06	-0,02	0,00	PA1797 -	-0,22	ND	ND	PA3841 exoS	**-2,56**	ND	ND
PA0012 -	-0,15	ND	ND	PA1798 parS	0,57	0,33	0,36	PA3842 -	**-1,81**	-0,86	-0,65
PA0013 -	0,40	ND	ND	PA1799 parR	**1,18**	0,29	-0,34	PA3843 -	**-2,43**	ND	ND
PA0014 -	0,39	ND	ND	PA1800 tig	0,47	0,31	0,41	PA3844 -	-0,87	ND	ND
PA0015 -	-0,06	-0,22	-0,56	PA1801 clpP	0,18	0,11	0,12	PA3845 -	-0,50	ND	ND
PA0016 trkA	-0,02	0,10	0,30	PA1802 clpX	0,26	0,05	-0,13	PA3846 -	-0,96	ND	ND
PA0017 -	-0,06	0,05	0,19	PA1803 lon	0,10	0,05	0,05	PA3847 -	-0,17	ND	ND
PA0018 fmt	0,12	0,07	0,08	PA1804 hupB	-0,17	-0,12	-0,17	PA3848 -	-0,61	-0,30	-0,25
PA0019 def	0,11	0,04	0,00	PA1804.1 -	0,05	ND	ND	PA3849 -	-0,40	-0,28	-0,40
PA0020 -	-0,85	-0,86	**-1,60**	PA1805 ppiD	0,25	0,19	0,28	PA3850 -	-0,54	ND	ND
PA0022 -	-0,07	ND	ND	PA1806 fabI	0,63	0,29	0,19	PA3851 -	**-1,08**	ND	ND
PA0023 qor	-0,37	-0,36	-0,66	PA1807 -	0,58	0,29	0,24	PA3852 -	-0,67	-0,07	0,48
PA0024 hemF	0,17	0,17	0,30	PA1808 -	0,66	0,65	**1,18**	PA3853 -	-0,14	ND	ND
PA0025 aroE	0,27	-0,02	-0,31	PA1809 -	0,75	0,09	-0,50	PA3854 -	-0,07	ND	ND
PA0026 plcB	**-2,00**	ND	ND	PA1810 -	0,41	0,35	0,59	PA3855 -	-0,31	ND	ND
PA0027 -	**-1,67**	ND	ND	PA1811 -	0,26	ND	ND	PA3856 -	**-1,29**	ND	ND
PA0028 -	**-1,79**	ND	ND	PA1812 mltD	0,35	0,09	-0,10	PA3857 pcs	**-1,00**	ND	ND
PA0031 betC	-0,45	ND	ND	PA1813 -	-0,13	0,31	**1,00**	PA3858 -	-0,81	ND	ND
PA0032 -	-0,42	ND	ND	PA1814 -	0,42	ND	ND	PA3859 -	-0,60	-0,35	-0,40
PA0033 -	-0,76	ND	ND	PA1815 rnhA	0,82	0,30	0,05	PA3860 -	-0,74	ND	ND
PA0034 -	-0,07	ND	ND	PA1816 dnaQ	0,56	0,12	-0,23	PA3861 rhl	-0,26	0,02	0,30
PA0035 trpA	-0,87	-0,19	0,33	PA1817 -	0,61	ND	ND	PA3862 dauB	-0,32	-0,04	0,21
PA0036 trpB	**-1,06**	-0,22	0,43	PA1818 ldcA	**1,28**	**1,49**	**2,96**	PA3863 dauA	-0,56	ND	ND
PA0037 trpI	-0,60	ND	ND	PA1819 -	0,95	ND	ND	PA3864 dauR	-0,33	ND	ND
PA0038 -	**-1,03**	ND	ND	PA1820 nhaB	0,07	0,02	-0,01	PA3865 -	-0,39	0,13	0,77
PA0039 -	0,67	ND	ND	PA1821 -	-0,24	0,06	0,42	PA3866 -	**-1,42**	-0,40	0,27
PA0040 -	0,04	0,18	0,49	PA1822 fimL	0,09	-0,05	-0,24	PA3867 -	-0,53	ND	ND
PA0041 -	-0,27	ND	ND	PA1823 -	0,08	0,11	0,24	PA3869 -	-0,70	ND	ND
PA0042 -	0,19	ND	ND	PA1824 -	0,67	ND	ND	PA3878 narX	0,56	ND	ND
PA0043 -	-0,46	ND	ND	PA1825 -	0,69	-0,26	**-1,45**	PA3879 narL	0,18	ND	ND
PA0044 exoT	**-2,02**	ND	ND	PA1826 -	0,55	ND	ND	PA3880 -	-0,95	ND	ND
PA0045 -	-0,62	0,04	0,74	PA1828 -	-0,32	0,06	0,49	PA3881 -	**-1,09**	ND	ND
PA0046 -	-0,63	-0,02	0,57	PA1829 -	-0,33	0,12	0,68	PA3882 -	-0,64	ND	ND
PA0047 -	-0,58	-0,07	0,37	PA1830 -	0,12	0,15	0,31	PA3883 -	-0,23	ND	ND
PA0048 -	0,39	ND	ND	PA1831 -	0,04	0,28	0,74	PA3884 -	0,22	ND	ND
PA0049 -	**2,90**	ND	ND	PA1832 -	0,28	0,14	0,11	PA3886 -	-0,97	-0,28	0,18
PA0050 -	0,46	ND	ND	PA1833 -	0,44	-0,08	-0,65	PA3887 nhaP	**-1,10**	-0,87	**-1,36**
PA0053 -	-0,14	ND	ND	PA1834 -	0,47	ND	ND	PA3888 -	-0,87	ND	ND
PA0054 -	-0,52	ND	ND	PA1835 -	-0,85	ND	ND	PA3890 -	-0,24	ND	ND
PA0055 -	-0,08	-0,20	-0,50	PA1836 -	0,19	ND	ND	PA3891 -	0,54	ND	ND
PA0056 -	-0,13	ND	ND	PA1837 -	0,94	0,56	0,66	PA3892 -	**2,13**	0,97	0,63
PA0060 -	-0,38	ND	ND	PA1838 cysI	**1,17**	0,47	0,16	PA3893 -	**2,04**	ND	ND
PA0061 -	-0,45	ND	ND	PA1839 -	0,94	ND	ND	PA3894 -	**2,28**	0,68	-0,35
PA0062 -	0,00	ND	ND	PA1840 -	0,77	ND	ND	PA3895 -	0,00	-0,20	-0,56
PA0063 -	-0,32	ND	ND	PA1841 -	0,31	0,15	0,13	PA3896 -	-0,36	-0,10	0,07
PA0064 -	-0,21	ND	ND	PA1842 -	0,39	ND	ND	PA3897 -	-0,87	ND	ND
PA0065 -	0,06	ND	ND	PA1843 metH	0,19	0,14	0,22	PA3898 -	-0,30	ND	ND
PA0066 -	-0,09	ND	ND	PA1844 -	0,34	ND	ND	PA3899 -	-0,53	ND	ND
PA0067 prlC	-0,45	-0,15	0,02	PA1845 -	0,42	ND	ND	PA3901 fecA	-0,44	**1,36**	**4,31**
PA0068 -	-0,32	ND	ND	PA1846 cti	0,37	0,17	0,12	PA3902 -	-0,47	ND	ND
PA0069 -	-0,49	ND	ND	PA1847 -	-0,06	0,32	0,98	PA3903 prfC	-0,23	-0,11	-0,07
PA0070 -	0,06	0,02	0,00	PA1850 -	0,00	ND	ND	PA3904 -	-0,32	ND	ND
PA0071 -	0,00	-0,10	-0,29	PA1851 -	0,83	ND	ND	PA3905 -	-0,17	ND	ND
PA0072 -	0,30	ND	ND	PA1852 -	**1,41**	-0,17	**-1,90**	PA3906 -	-0,31	ND	ND
PA0073 -	0,03	ND	ND	PA1853 -	0,37	ND	ND	PA3907 -	-0,30	ND	ND
PA0074 ppkA	-0,14	-0,12	-0,20	PA1854 -	0,36	ND	ND	PA3908 -	-0,36	-0,30	-0,51

ID – Gene [a]	TC [b]	PC [c]	PTV[d]	ID – Gene [a]	TC [b]	PC [c]	PTV[d]	ID – Gene [a]	TC [b]	PC [c]	PTV[d]
PA0075 pppA	0,27	-0,09	-0,54	PA1856 -	-0,11	ND	ND	PA3911 -	1,27	ND	ND
PA0076 -	0,24	-0,23	-0,90	PA1857 -	0,53	0,16	-0,08	PA3912 -	0,58	ND	ND
PA0077 icmF1	-0,05	-0,16	-0,40	PA1858 str	0,22	ND	ND	PA3913 -	0,08	ND	ND
PA0078 -	-0,05	-0,08	-0,17	PA1859 -	0,14	ND	ND	PA3916 moaE	-0,26	ND	ND
PA0079 -	-0,03	-0,24	-0,65	PA1861 modC	0,37	ND	ND	PA3917 moaD	-0,71	ND	ND
PA0080 -	0,07	0,03	0,02	PA1862 modB	0,69	ND	ND	PA3918 moaC	-0,66	-0,12	0,32
PA0081 fha1	0,04	-0,56	-1,63	PA1863 modA	0,21	0,76	1,94	PA3919 -	-1,24	-0,03	1,15
PA0082 -	0,00	-0,34	-0,97	PA1865 -	0,00	ND	ND	PA3921 -	-0,26	ND	ND
PA0083 -	-0,29	-0,47	-1,06	PA1866 -	-0,39	ND	ND	PA3923 -	-1,10	ND	ND
PA0084 -	-0,16	-0,46	-1,14	PA1869 -	-0,32	ND	ND	PA3925 -	0,33	0,85	2,10
PA0085 hcp1	-0,20	-0,49	-1,19	PA1871 lasA	-0,22	ND	ND	PA3927 -	-1,93	ND	ND
PA0086 -	0,23	-0,25	-0,95	PA1872 -	-0,31	ND	ND	PA3928 -	-0,70	ND	ND
PA0087 -	0,00	ND	ND	PA1878 -	0,24	ND	ND	PA3929 cioB	-1,18	ND	ND
PA0088 -	-0,03	-0,20	-0,55	PA1879 -	0,32	ND	ND	PA3930 cioA	-2,17	ND	ND
PA0089 -	-0,11	ND	ND	PA1881 -	0,29	ND	ND	PA3931 -	ND	0,60	ND
PA0090 clpV1	-0,08	-0,14	-0,32	PA1882 -	-0,42	ND	ND	PA3933 -	-0,63	ND	ND
PA0091 vgrG1	0,03	-0,22	-0,64	PA1883 -	1,87	ND	ND	PA3934 -	-0,37	-0,07	0,18
PA0092 -	0,03	0,04	0,07	PA1884 -	-0,39	ND	ND	PA3939 -	-1,46	ND	ND
PA0093 -	0,00	ND	ND	PA1885 -	-0,46	ND	ND	PA3940 -	-0,32	-0,19	-0,23
PA0094 -	-0,14	-0,15	-0,28	PA1886 polB	0,69	0,17	-0,20	PA3941 -	-0,32	-0,42	-0,87
PA0095 -	-1,20	0,14	1,58	PA1887 -	0,29	ND	ND	PA3942 tesB	-0,54	-0,41	-0,62
PA0096 -	-0,93	ND	ND	PA1889 -	0,74	ND	ND	PA3943 -	-0,30	ND	ND
PA0097 -	-1,17	ND	ND	PA1890 -	0,17	0,21	0,44	PA3944 -	-0,24	ND	ND
PA0098 -	-1,09	ND	ND	PA1893 -	1,26	ND	ND	PA3945 -	-0,95	ND	ND
PA0099 -	-0,97	ND	ND	PA1894 -	1,49	ND	ND	PA3947 rocR	-0,58	ND	ND
PA0100 -	-0,85	-0,35	-0,14	PA1895 -	1,17	ND	ND	PA3948 rocA1	-0,69	-0,35	-0,30
PA0101 -	-1,00	ND	ND	PA1896 -	1,10	ND	ND	PA3949 -	-0,26	0,00	0,26
PA0102 -	-0,22	-0,50	-1,20	PA1897 -	1,13	ND	ND	PA3950 -	-0,28	-0,08	0,05
PA0103 -	-0,75	ND	ND	PA1901 phzC2	-0,13	ND	ND	PA3951 -	-0,30	0,02	0,34
PA0104 -	-0,54	ND	ND	PA1906 -	0,49	ND	ND	PA3952 -	-0,33	ND	ND
PA0105 coxB	-0,66	ND	ND	PA1912 femI	1,58	ND	ND	PA3955 -	-0,15	ND	ND
PA0109 -	-0,21	ND	ND	PA1913 -	1,69	0,77	0,50	PA3956 -	-1,27	ND	ND
PA0113 -	-0,81	ND	ND	PA1915 -	0,19	ND	ND	PA3957 -	-1,00	ND	ND
PA0114 senC	-0,58	ND	ND	PA1919 nrdG	0,29	ND	ND	PA3958 -	-0,60	-0,12	0,26
PA0115 -	-0,67	-0,19	0,12	PA1926 -	0,33	0,20	0,24	PA3959 -	1,05	ND	ND
PA0116 -	-0,27	ND	ND	PA1927 metE	0,46	ND	ND	PA3960 -	1,19	ND	ND
PA0117 -	-0,40	ND	ND	PA1928 rimJ	0,21	ND	ND	PA3961 -	-0,21	-0,05	0,07
PA0118 -	-0,33	0,06	0,50	PA1929 -	0,58	ND	ND	PA3962 -	-0,37	ND	ND
PA0119 -	-1,17	ND	ND	PA1932 -	-0,46	ND	ND	PA3963 -	-0,39	ND	ND
PA0120 -	0,05	ND	ND	PA1933 -	-0,26	ND	ND	PA3965 -	-0,37	-0,15	-0,06
PA0121 -	-0,14	ND	ND	PA1934 -	0,11	ND	ND	PA3966 -	-0,19	ND	ND
PA0122 -	-0,85	ND	ND	PA1935 -	0,64	ND	ND	PA3967 -	-0,18	-0,36	-0,86
PA0123 -	-0,04	ND	ND	PA1936 -	0,47	ND	ND	PA3968 -	-0,30	-0,31	-0,58
PA0124 -	-0,15	ND	ND	PA1937 -	0,31	ND	ND	PA3969 -	-0,71	ND	ND
PA0125 -	0,00	0,04	0,11	PA1938 -	0,76	ND	ND	PA3970 amn	-0,92	-0,25	0,21
PA0126 -	0,02	-0,11	-0,34	PA1939 -	0,24	0,19	0,30	PA3971 -	-0,24	ND	ND
PA0127 -	-0,75	-0,42	-0,44	PA1940 -	0,97	0,39	0,15	PA3972 -	-0,50	-0,11	0,19
PA0128 -	0,35	-0,26	-1,10	PA1941 -	0,75	0,51	0,71	PA3973 -	-1,66	ND	ND
PA0129 gabP	-0,26	ND	ND	PA1942 -	0,00	ND	ND	PA3974 ladS	-0,26	ND	ND
PA0130 -	-1,02	1,03	3,94	PA1943 -	0,42	ND	ND	PA3975 thiD	-0,20	0,00	0,20
PA0131 -	-1,28	ND	ND	PA1944 -	0,39	ND	ND	PA3976 thiE	-0,44	0,00	0,43
PA0132 -	-1,36	1,41	5,37	PA1946 rbsB	-0,67	ND	ND	PA3977 hemL	-0,20	-0,19	-0,33
PA0133 -	-0,61	ND	ND	PA1947 rbsA	1,15	ND	ND	PA3978 -	-0,32	0,02	0,37
PA0134 -	-0,09	ND	ND	PA1948 rbsC	1,48	ND	ND	PA3979 -	0,20	-0,25	-0,90
PA0139 ahpC	-0,50	0,22	1,11	PA1949 rbsR	1,46	0,92	1,17	PA3980 -	-0,14	-0,35	-0,87
PA0140 ahpF	0,10	0,27	0,67	PA1950 rbsK	1,26	0,46	0,06	PA3981 -	-0,46	0,07	0,64
PA0141 -	0,38	-0,25	-1,08	PA1951 -	1,27	ND	ND	PA3982 -	0,01	0,24	0,66
PA0142 -	-0,45	ND	ND	PA1957 -	0,55	ND	ND	PA3983 -	0,19	0,29	0,64
PA0143 nuh	-0,34	-0,35	-0,66	PA1958 -	0,75	ND	ND	PA3984 lnt	-0,04	-0,19	-0,48
PA0144 -	-0,34	ND	ND	PA1959 bacA	0,59	ND	ND	PA3985 -	-0,58	ND	ND
PA0145 -	-0,58	ND	ND	PA1960 -	0,29	ND	ND	PA3986 -	-0,53	ND	ND
PA0147 -	-1,00	ND	ND	PA1961 -	0,34	ND	ND	PA3987 leuS	-0,41	-0,22	-0,21
PA0148 -	-0,73	-0,32	-0,19	PA1962 azoR2	0,65	ND	ND	PA3988 -	-0,25	-0,36	-0,77
PA0149 -	0,16	ND	ND	PA1963 -	-0,29	ND	ND	PA3989 holA	-0,05	0,00	0,06
PA0150 -	0,05	ND	ND	PA1964 -	0,28	0,34	0,68	PA3990 -	0,91	ND	ND
PA0152 pcaQ	-0,86	ND	ND	PA1965 -	0,47	ND	ND	PA3991 -	-0,45	ND	ND
PA0154 pcaG	-1,19	ND	ND	PA1966 -	0,82	ND	ND	PA3992 -	-0,73	-0,35	-0,27
PA0155 pcaR	-0,13	0,07	0,34	PA1967 -	0,25	ND	ND	PA3993 -	-0,19	ND	ND
PA0156 -	-0,03	0,00	0,02	PA1968 -	0,28	ND	ND	PA3995 -	-0,29	ND	ND
PA0157 -	-0,25	-0,21	-0,35	PA1969 -	0,22	0,18	0,31	PA3996 lis	0,06	-0,13	-0,42
PA0158 -	-0,34	0,02	0,40	PA1970 -	0,29	ND	ND	PA3997 lipB	0,16	-0,10	-0,45
PA0159 -	-0,21	ND	ND	PA1971 braZ	-0,68	ND	ND	PA3998 -	-0,12	0,04	0,23
PA0160 -	1,39	ND	ND	PA1973 pqqF	-0,32	ND	ND	PA3999 dacC	-0,08	-0,15	-0,34
PA0161 -	1,59	ND	ND	PA1978 erbR	-2,46	ND	ND	PA4000 -	-0,02	-0,04	-0,11
PA0162 opdC	0,53	0,24	0,14	PA1984 exaC	1,92	ND	ND	PA4001 sltB1	0,11	-0,03	-0,19
PA0163 -	-0,21	ND	ND	PA1985 pqqA	-2,35	ND	ND	PA4002 rodA	-0,05	ND	ND

ID – Gene [a]	TC [b]	PC [c]	PTV[d]
PA0164 -	**-1,98**	ND	ND
PA0165 -	-0,48	-0,27	-0,29
PA0167 -	**-1,05**	-0,05	0,91
PA0168 -	-0,40	ND	ND
PA0169 -	**1,27**	**1,05**	**1,72**
PA0170 -	**1,39**	0,96	**1,33**
PA0171 -	**1,50**	**1,44**	**2,60**
PA0172 -	**1,06**	ND	ND
PA0179 -	**-1,05**	ND	ND
PA0180 cttP	-0,34	ND	ND
PA0181 -	-0,86	ND	ND
PA0182 -	-0,35	ND	ND
PA0183 atsA	-0,46	ND	ND
PA0195 pntAA	**1,53**	0,53	-0,03
PA0195.1 pntAB	0,46	ND	ND
PA0196 pntB	**1,27**	ND	ND
PA0199 exbD1	-0,35	ND	ND
PA0200 -	**-2,34**	ND	ND
PA0201 -	-0,52	ND	ND
PA0202 -	0,65	ND	ND
PA0203 -	**1,87**	ND	ND
PA0205 -	-0,29	ND	ND
PA0206 -	0,64	ND	ND
PA0207 -	-0,79	ND	ND
PA0208 mdcA	-0,58	ND	ND
PA0209 -	**2,46**	ND	ND
PA0210 mdcC	**2,81**	ND	ND
PA0211 mdcD	**1,68**	ND	ND
PA0212 mdcE	**1,49**	ND	ND
PA0213 -	**1,22**	ND	ND
PA0214 -	0,24	ND	ND
PA0215 -	-0,66	ND	ND
PA0216 -	0,89	ND	ND
PA0217 -	-0,72	ND	ND
PA0218 -	-0,88	ND	ND
PA0223 -	-0,42	ND	ND
PA0224 -	-0,13	ND	ND
PA0225 -	-0,57	ND	ND
PA0226 -	0,00	ND	ND
PA0230 pcaB	-0,63	ND	ND
PA0231 pcaD	-0,66	ND	ND
PA0232 pcaC	-0,37	ND	ND
PA0233 -	-0,87	ND	ND
PA0234 -	0,53	ND	ND
PA0236 -	0,21	-0,07	-0,42
PA0239 -	-0,21	ND	ND
PA0243 -	-0,28	ND	ND
PA0245 aroQ2	-0,46	ND	ND
PA0246 -	-0,43	ND	ND
PA0248 -	-0,79	ND	ND
PA0249 -	0,10	ND	ND
PA0250 -	-0,77	-0,09	0,52
PA0252 -	0,08	ND	ND
PA0253 -	-0,20	ND	ND
PA0254 -	-0,24	ND	ND
PA0255 -	-0,31	ND	ND
PA0256 -	**-1,00**	ND	ND
PA0257 -	-0,50	ND	ND
PA0258 -	-0,84	ND	ND
PA0259 -	-0,60	ND	ND
PA0260 -	-0,39	ND	ND
PA0261 -	-0,33	ND	ND
PA0262 -	-0,58	0,52	**2,08**
PA0263 hcpC	-0,87	ND	ND
PA0263.1 -	0,59	ND	ND
PA0264 -	**-1,33**	ND	ND
PA0265 gabD	**-1,44**	-0,18	0,92
PA0266 gabT	**-1,69**	-0,21	**1,10**
PA0267 -	-0,33	ND	ND
PA0268 -	-0,36	ND	ND
PA0269 -	-0,92	0,14	**1,31**
PA0270 -	-0,74	ND	ND
PA0271 -	0,06	ND	ND
PA0272 -	-0,61	ND	ND
PA0275 -	**-1,55**	ND	ND
PA0276 -	-0,24	ND	ND

ID – Gene [a]	TC [b]	PC [c]	PTV[d]
PA1986 pqqB	**-2,00**	ND	ND
PA1987 pqqC	**-1,70**	ND	ND
PA1988 pqqD	**-1,39**	ND	ND
PA1989 pqqE	**-1,00**	ND	ND
PA1991 -	0,44	ND	ND
PA1992 ercS	**1,24**	ND	ND
PA1993 -	**1,13**	ND	ND
PA1994 -	0,22	ND	ND
PA1995 -	0,49	ND	ND
PA1996 ppiC1	0,73	ND	ND
PA1997 -	0,07	ND	ND
PA1998 dhcR	-0,93	0,16	**1,39**
PA1999 dhcA	**4,29**	**2,37**	**2,46**
PA2000 dhcB	**4,07**	**1,99**	**1,61**
PA2001 atoB	**3,42**	**2,44**	**3,51**
PA2002 -	**3,70**	**2,84**	**4,37**
PA2003 bdhA	**2,44**	ND	ND
PA2004 -	**1,14**	ND	ND
PA2005 -	**1,16**	ND	ND
PA2006 -	**3,33**	ND	ND
PA2007 maiA	**3,66**	**1,86**	**1,62**
PA2008 fahA	**3,24**	**2,58**	**4,09**
PA2009 hmgA	**3,36**	**1,68**	**1,43**
PA2010 -	**1,20**	0,50	0,21
PA2011 liuE	**1,87**	0,59	-0,19
PA2012 liuD	**2,21**	0,98	0,58
PA2013 liuC	**2,27**	**1,76**	**2,74**
PA2014 liuB	**2,37**	**1,32**	**1,39**
PA2015 liuA	**2,16**	**2,06**	**3,69**
PA2016 liuR	0,52	0,66	**1,35**
PA2017 -	0,46	ND	ND
PA2018 -	-0,60	0,04	0,71
PA2019 -	-0,66	-0,10	0,37
PA2020 -	0,18	0,16	0,29
PA2021 -	-0,53	ND	ND
PA2022 -	0,87	ND	ND
PA2023 galU	0,72	0,20	-0,15
PA2024 -	0,22	ND	ND
PA2025 gor	-0,14	0,01	0,16
PA2026 -	**-2,64**	ND	ND
PA2028 -	0,17	ND	ND
PA2029 -	-0,21	ND	ND
PA2030 -	0,30	ND	ND
PA2031 -	0,28	ND	ND
PA2032 -	0,57	ND	ND
PA2037 -	0,52	ND	ND
PA2038 -	0,77	ND	ND
PA2039 -	0,50	ND	ND
PA2040 -	**-1,00**	0,16	**1,47**
PA2041 -	-0,78	ND	ND
PA2042 -	0,30	0,07	-0,09
PA2043 -	-0,09	ND	ND
PA2044 -	-0,01	0,18	0,52
PA2045 -	0,52	ND	ND
PA2047 -	0,00	ND	ND
PA2048 -	**2,32**	ND	ND
PA2049 -	0,31	ND	ND
PA2052 cynS	0,29	ND	ND
PA2054 cynR	-0,35	ND	ND
PA2056 -	-0,29	ND	ND
PA2063 -	0,74	ND	ND
PA2064 pcoB	0,69	ND	ND
PA2071 fusA2	0,00	0,05	0,14
PA2076 -	0,32	-0,02	-0,38
PA2077 -	0,87	ND	ND
PA2079 -	0,82	ND	ND
PA2080 kynU	0,48	0,54	**1,05**
PA2081 kynB	0,31	0,46	1,00
PA2082 -	0,03	ND	ND
PA2083 -	0,13	ND	ND
PA2097 -	**1,46**	ND	ND
PA2100 -	-0,05	-0,06	-0,12
PA2101 -	-0,09	ND	ND
PA2102 -	0,26	ND	ND
PA2103 -	0,00	ND	ND
PA2104 -	0,22	ND	ND

ID – Gene [a]	TC [b]	PC [c]	PTV[d]
PA4003 pbpA	-0,23	-0,20	-0,34
PA4004 -	-0,23	-0,13	-0,13
PA4005 -	-0,25	-0,02	0,20
PA4006 nadD	-0,20	ND	ND
PA4007 proA	-0,20	-0,23	-0,46
PA4009 -	-0,46	ND	ND
PA4010 -	0,08	-0,04	-0,20
PA4011 -	-0,19	0,13	0,56
PA4012 -	-0,33	ND	ND
PA4013 -	-0,46	ND	ND
PA4014 -	-0,38	ND	ND
PA4015 -	-0,87	-0,11	0,54
PA4016 -	-0,56	ND	ND
PA4017 -	-0,37	-0,24	-0,33
PA4018 -	-0,38	ND	ND
PA4019 -	-0,43	-0,10	0,13
PA4020 mpl	-0,39	-0,14	-0,02
PA4021 -	-0,19	ND	ND
PA4022 -	**2,04**	**2,12**	**3,99**
PA4023 -	**1,19**	ND	ND
PA4024 eutB	**1,22**	ND	ND
PA4025 -	0,92	ND	ND
PA4026 -	**-1,02**	-0,35	0,02
PA4027 -	-0,35	ND	ND
PA4029 -	-0,06	0,04	0,17
PA4030 -	-0,28	ND	ND
PA4031 ppa	-0,31	-0,38	-0,77
PA4032 -	0,27	0,19	0,26
PA4033 -	**-1,38**	ND	ND
PA4034 aqpZ	**-1,26**	ND	ND
PA4035 -	-0,85	-0,30	-0,01
PA4036 -	-0,24	ND	ND
PA4042 xseB	-0,45	-0,37	-0,61
PA4043 ispA	-0,48	-0,35	-0,52
PA4044 dxs	-0,26	-0,23	-0,41
PA4045 -	-0,35	ND	ND
PA4046 -	-0,20	ND	ND
PA4047 ribA	-0,24	-0,10	-0,06
PA4048 -	-0,60	ND	ND
PA4049 -	0,00	ND	ND
PA4050 pgpA	0,20	ND	ND
PA4051 thiL	-0,02	-0,04	-0,09
PA4052 nusB	-0,13	0,04	0,24
PA4053 ribE	0,00	-0,11	-0,30
PA4054 ribB	0,52	-0,03	-0,60
PA4055 ribC	0,54	0,15	-0,11
PA4056 ribD	-0,48	-0,13	0,11
PA4057 nrdR	-0,44	-0,30	-0,41
PA4058 -	0,03	0,01	0,01
PA4059 -	-0,19	ND	ND
PA4060 -	0,09	-0,02	-0,14
PA4061 -	-0,35	-0,32	-0,56
PA4062 -	-0,83	ND	ND
PA4063 -	0,00	ND	ND
PA4064 -	**-1,19**	ND	ND
PA4065 -	-0,63	ND	ND
PA4066 -	-0,40	ND	ND
PA4067 oprG	0,60	-0,57	**-2,24**
PA4068 -	-0,31	-0,37	-0,73
PA4069 -	-0,68	-0,15	0,25
PA4070 -	**-1,14**	ND	ND
PA4074 -	-0,74	ND	ND
PA4075 -	-0,72	ND	ND
PA4076 -	-0,09	ND	ND
PA4077 -	-0,34	ND	ND
PA4079 -	-0,65	-0,11	0,33
PA4080 -	-0,63	ND	ND
PA4086 cupB1	0,13	ND	ND
PA4090 -	**-1,08**	ND	ND
PA4094 -	-0,71	ND	ND
PA4101 bfmR	**-1,00**	ND	ND
PA4108 -	**-2,38**	ND	ND
PA4109 ampR	-0,20	ND	ND
PA4111 -	**-1,32**	ND	ND
PA4112 -	0,00	ND	ND
PA4113 -	-0,11	ND	ND

ID – Gene [a]	TC [b]	PC [c]	PTV[d]
PA0277 -	0,45	-0,04	-0,58
PA0279 -	-0,52	ND	ND
PA0280 cysA	0,68	0,08	-0,45
PA0281 cysW	0,31	ND	ND
PA0282 cysT	0,10	ND	ND
PA0283 sbp	0,31	ND	ND
PA0284 -	0,42	ND	ND
PA0285 -	-0,57	0,00	0,55
PA0286 desA	-0,10	ND	ND
PA0289 gpuR	-0,82	ND	ND
PA0290 -	-0,79	ND	ND
PA0291 oprE	-0,13	0,28	0,95
PA0292 aguA	-0,17	-0,25	-0,54
PA0293 aguB	0,17	0,25	0,54
PA0294 aguR	-0,19	ND	ND
PA0295 -	**-1,09**	-0,35	0,09
PA0296 spuI	-0,61	-0,04	0,49
PA0297 spuA	-0,39	0,60	**2,11**
PA0298 spuB	-0,51	0,53	**2,01**
PA0299 spuC	-0,57	0,31	**1,46**
PA0300 spuD	-0,02	0,49	**1,40**
PA0301 spuE	-0,40	-0,63	**-1,38**
PA0302 spuF	-0,50	0,12	0,84
PA0303 spuG	-0,42	ND	ND
PA0304 spuH	-0,30	ND	ND
PA0305 -	-0,58	-0,17	0,11
PA0306 -	-0,62	ND	ND
PA0307 -	-0,17	ND	ND
PA0308 -	-0,28	0,14	0,67
PA0309 -	0,13	-0,12	-0,47
PA0310 -	-0,80	ND	ND
PA0311 -	-0,54	ND	ND
PA0312 -	-0,59	ND	ND
PA0313 -	-0,22	ND	ND
PA0314 -	-0,04	0,24	0,71
PA0315 -	**-1,06**	-0,11	0,76
PA0316 serA	0,11	0,09	0,13
PA0317 -	-0,20	-0,02	0,13
PA0318 -	-0,68	-0,44	-0,59
PA0319 -	-0,19	0,10	0,47
PA0320 -	-0,12	ND	ND
PA0327 -	**-1,44**	ND	ND
PA0328 -	**-2,15**	-0,40	**1,01**
PA0329 -	-0,08	-0,21	-0,51
PA0330 rpiA	-0,09	0,11	0,41
PA0331 ilvA1	-0,19	-0,04	0,07
PA0332 -	-0,31	ND	ND
PA0333 -	-0,13	0,04	0,24
PA0334 -	-0,55	ND	ND
PA0335 -	**-1,19**	-0,14	0,78
PA0336 ygdP	-0,27	ND	ND
PA0337 ptsP	-0,10	0,01	0,14
PA0338 -	-0,07	ND	ND
PA0339 -	-0,91	ND	ND
PA0340 -	0,00	ND	ND
PA0341 lgt	-0,01	-0,07	-0,18
PA0342 thyA	-0,02	-0,09	-0,23
PA0343 -	-0,06	ND	ND
PA0344 -	-0,69	ND	ND
PA0345 -	-0,81	-0,12	0,46
PA0346 -	0,07	ND	ND
PA0347 glpQ	0,28	ND	ND
PA0350 folA	0,06	-0,23	-0,72
PA0351 -	-0,63	ND	ND
PA0352 -	0,15	ND	ND
PA0353 ilvD	-0,51	-0,44	-0,75
PA0354 -	-0,22	-0,07	0,02
PA0356 -	0,06	-0,04	-0,17
PA0357 mutM	-0,05	-0,18	-0,45
PA0358 -	**1,20**	0,60	0,50
PA0359 -	**-1,53**	ND	ND
PA0360 -	-0,58	ND	ND
PA0361 -	-0,25	ND	ND
PA0362 fdx1	-0,01	ND	ND
PA0363 coaD	-0,17	-0,19	-0,36
PA0364 -	0,00	ND	ND

ID – Gene [a]	TC [b]	PC [c]	PTV[d]
PA2105 -	0,31	ND	ND
PA2106 -	0,22	ND	ND
PA2108 -	**3,62**	ND	ND
PA2109 -	**7,76**	ND	ND
PA2110 -	**8,59**	**2,78**	-0,67
PA2111 -	**8,65**	**2,80**	-0,69
PA2112 -	**8,45**	**2,74**	-0,66
PA2113 opdO	**7,97**	**3,03**	0,65
PA2114 -	**5,92**	**2,13**	0,13
PA2115 -	**2,78**	0,70	-0,80
PA2116 -	**5,32**	**2,19**	0,92
PA2117 -	**3,94**	ND	ND
PA2118 ada	**1,55**	ND	ND
PA2119 -	0,10	0,11	0,23
PA2121 -	-0,87	ND	ND
PA2123 -	0,13	ND	ND
PA2125 -	**2,46**	ND	ND
PA2126 -	**3,60**	ND	ND
PA2127 -	**1,54**	ND	ND
PA2128 cupA1	**4,01**	ND	ND
PA2129 cupA2	**5,04**	ND	ND
PA2130 cupA3	**3,17**	ND	ND
PA2131 cupA4	**3,58**	ND	ND
PA2166 -	-0,87	ND	ND
PA2182 -	**1,87**	ND	ND
PA2191 exoY	**-1,17**	ND	ND
PA2193 hcnA	0,83	ND	ND
PA2194 hcnB	**1,11**	ND	ND
PA2195 hcnC	**1,39**	-0,75	**-3,52**
PA2196 -	0,34	ND	ND
PA2197 -	-0,26	-0,21	-0,34
PA2198 -	-0,24	-0,02	0,19
PA2199 -	-0,21	-0,32	-0,71
PA2200 -	0,00	ND	ND
PA2201 -	-0,11	ND	ND
PA2202 -	**1,37**	ND	ND
PA2203 -	**2,81**	ND	ND
PA2204 -	**2,01**	**1,33**	**1,77**
PA2205 -	0,39	ND	ND
PA2206 -	0,00	ND	ND
PA2218 -	0,42	ND	ND
PA2220 -	-0,08	ND	ND
PA2222 -	0,08	ND	ND
PA2223 -	0,67	ND	ND
PA2224 -	0,89	ND	ND
PA2225 -	**1,38**	ND	ND
PA2226 -	0,37	ND	ND
PA2227 vqsM	0,70	ND	ND
PA2228 -	**1,14**	ND	ND
PA2229 -	0,05	0,29	0,76
PA2230 -	0,72	ND	ND
PA2231 pslA	**1,56**	**1,16**	**1,74**
PA2232 pslB	**1,82**	0,88	0,68
PA2233 pslC	**1,81**	0,73	0,27
PA2234 pslD	**2,03**	**1,44**	**2,06**
PA2235 pslE	**1,93**	**1,20**	**1,49**
PA2236 pslF	**2,03**	**1,08**	**1,05**
PA2237 pslG	**2,14**	**1,10**	0,98
PA2238 pslH	**2,19**	**1,23**	**1,30**
PA2239 pslI	**2,38**	0,76	-0,22
PA2240 pslJ	**2,29**	ND	ND
PA2241 pslK	**2,22**	0,40	**-1,08**
PA2242 pslL	**2,09**	**1,42**	**1,94**
PA2244 pslN	-0,24	ND	ND
PA2245 pslO	0,52	ND	ND
PA2246 bkdR	-0,97	ND	ND
PA2247 bkdA1	**3,61**	**1,96**	**1,97**
PA2248 bkdA2	**3,98**	**2,79**	**3,97**
PA2249 bkdB	**3,99**	**2,17**	**2,18**
PA2250 lpdV	**4,12**	**2,35**	**2,57**
PA2251 -	0,62	ND	ND
PA2252 -	**-3,32**	0,89	**5,85**
PA2253 ansA	**-2,19**	0,31	**3,07**
PA2258 ptxR	**-1,00**	ND	ND
PA2259 ptxS	-0,31	ND	ND
PA2264 -	0,24	-0,12	-0,58

ID – Gene [a]	TC [b]	PC [c]	PTV[d]
PA4114 -	-0,73	ND	ND
PA4115 -	**-1,51**	-0,61	-0,23
PA4116 bphO	-0,73	ND	ND
PA4117 bphP	-0,60	ND	ND
PA4118 -	-0,43	ND	ND
PA4119 aph	-0,64	ND	ND
PA4124 hpcB	**-1,14**	ND	ND
PA4129 -	-0,13	ND	ND
PA4130 -	-0,65	ND	ND
PA4132 -	-0,94	ND	ND
PA4133 -	0,76	ND	ND
PA4134 -	-0,07	ND	ND
PA4135 -	-0,05	-0,08	-0,19
PA4136 -	-0,24	ND	ND
PA4137 -	0,46	ND	ND
PA4138 tyrS	0,24	-0,08	-0,46
PA4139 -	-0,36	ND	ND
PA4141 -	**-1,04**	ND	ND
PA4145 -	-0,91	ND	ND
PA4146 -	-0,87	ND	ND
PA4147 acoR	0,45	ND	ND
PA4149 -	-0,78	ND	ND
PA4150 -	**-1,14**	ND	ND
PA4154 -	-0,34	0,15	0,77
PA4157 -	0,14	ND	ND
PA4158 fepC	-0,24	ND	ND
PA4162 -	-0,87	ND	ND
PA4163 -	-0,25	-0,16	-0,19
PA4164 -	-0,36	ND	ND
PA4165 -	-0,68	ND	ND
PA4166 -	**-1,46**	ND	ND
PA4168 fpvB	**-1,00**	ND	ND
PA4169 -	-0,37	ND	ND
PA4170 -	-0,78	ND	ND
PA4174 -	-0,71	ND	ND
PA4175 piv	**-1,53**	ND	ND
PA4176 ppiC2	**-1,05**	-0,44	-0,20
PA4178 -	-0,21	ND	ND
PA4179 -	**-3,97**	**-1,73**	-0,96
PA4180 -	**-1,18**	-0,11	0,85
PA4181 -	-0,74	ND	ND
PA4182 -	-0,34	ND	ND
PA4183 -	-0,36	ND	ND
PA4184 -	0,21	ND	ND
PA4185 -	-0,34	ND	ND
PA4186 -	-0,58	ND	ND
PA4190 pqsL	-0,66	-0,20	0,08
PA4191 -	-0,64	ND	ND
PA4195 -	-0,42	ND	ND
PA4196 bfiR	0,16	ND	ND
PA4197 bfiS	-0,35	ND	ND
PA4198 -	-0,83	0,88	**3,32**
PA4199 -	-0,69	-0,08	0,47
PA4200 -	-0,20	-0,34	-0,76
PA4201 ddlA	-0,86	-0,21	0,27
PA4202 -	-0,53	-0,13	0,17
PA4203 -	-0,36	ND	ND
PA4204 ppgL	-0,45	ND	ND
PA4205 mexG	0,84	ND	ND
PA4206 mexH	**1,12**	ND	ND
PA4207 mexI	0,46	ND	ND
PA4212 phzC1	0,13	ND	ND
PA4217 phzS	**-1,46**	ND	ND
PA4227 pchR	0,11	ND	ND
PA4232 ssb	-0,58	-0,50	-0,84
PA4233 -	-0,06	-0,07	-0,12
PA4234 uvrA	-0,34	-0,30	-0,52
PA4235 bfrA	-0,56	0,01	0,59
PA4236 katA	-0,19	-0,20	-0,37
PA4237 rplQ	-0,26	-0,11	-0,05
PA4238 rpoA	-0,23	-0,17	-0,24
PA4239 rpsD	-0,28	-0,08	0,06
PA4240 rpsK	-0,25	-0,05	0,11
PA4241 rpsM	-0,15	-0,04	0,03
PA4242 rpmJ	-0,19	ND	ND
PA4243 secY	-0,19	-0,04	0,09

ID – Gene [a]	TC [b]	PC [c]	PTV[d]	ID – Gene [a]	TC [b]	PC [c]	PTV[d]	ID – Gene [a]	TC [b]	PC [c]	PTV[d]
PA0365 -	0,18	ND	ND	PA2265 -	0,21	0,07	-0,01	PA4244 rplO	-0,44	-0,10	0,17
PA0366 -	**-1,28**	-0,10	0,99	PA2266 -	0,37	0,00	-0,37	PA4245 rpmD	-0,31	-0,13	-0,07
PA0367 -	-0,57	ND	ND	PA2267 -	0,04	ND	ND	PA4246 rpsE	-0,32	-0,10	0,04
PA0368 -	-0,30	ND	ND	PA2268 -	-0,13	ND	ND	PA4247 rplR	-0,31	-0,11	-0,01
PA0369 -	0,08	ND	ND	PA2269 -	-0,22	ND	ND	PA4248 rplF	-0,34	-0,03	0,25
PA0370 -	-0,05	-0,23	-0,62	PA2270 -	0,21	ND	ND	PA4249 rpsH	-0,09	-0,09	-0,16
PA0371 -	0,02	-0,04	-0,13	PA2271 -	-0,19	ND	ND	PA4250 rpsN	-0,20	-0,07	0,01
PA0372 -	-0,06	-0,05	-0,09	PA2272 pbpC	0,43	ND	ND	PA4251 rplE	-0,27	-0,08	0,04
PA0373 ftsY	0,04	0,14	0,36	PA2273 soxR	-0,70	ND	ND	PA4252 rplX	-0,45	-0,10	0,15
PA0374 ftsE	0,04	-0,07	-0,23	PA2277 arsR	0,36	ND	ND	PA4253 rplN	-0,46	-0,07	0,26
PA0375 ftsX	0,19	0,02	-0,12	PA2278 arsB	0,28	ND	ND	PA4254 rpsQ	-0,43	-0,21	-0,18
PA0376 rpoH	0,09	ND	ND	PA2279 arsC	0,00	ND	ND	PA4255 rpmC	-0,28	0,04	0,39
PA0377 -	0,23	ND	ND	PA2280 -	0,47	ND	ND	PA4256 rplP	-0,33	0,01	0,36
PA0378 -	0,20	ND	ND	PA2281 -	0,42	ND	ND	PA4257 rpsC	-0,44	-0,09	0,17
PA0379 -	-0,27	ND	ND	PA2282 -	0,81	ND	ND	PA4258 rplV	-0,45	-0,09	0,20
PA0380 -	0,36	ND	ND	PA2285 -	0,32	0,23	0,33	PA4259 rpsS	-0,34	-0,07	0,13
PA0381 thiG	0,08	-0,13	-0,45	PA2286 -	0,24	ND	ND	PA4260 rplB	-0,28	-0,08	0,04
PA0382 micA	0,11	-0,01	-0,15	PA2287 -	-0,15	ND	ND	PA4261 rplW	-0,26	-0,05	0,12
PA0383 -	0,13	ND	ND	PA2288 -	-0,19	ND	ND	PA4262 rplD	-0,25	-0,04	0,14
PA0384 -	0,00	ND	ND	PA2289 -	0,93	0,38	0,14	PA4263 rplC	-0,01	-0,02	-0,06
PA0385 -	-0,05	ND	ND	PA2290 gcd	0,80	0,70	**1,20**	PA4264 rpsJ	-0,23	-0,08	0,01
PA0386 -	0,19	0,08	0,04	PA2291 -	**1,37**	**2,56**	**5,91**	PA4265 tufA	-0,39	-0,23	-0,25
PA0387 -	0,07	0,06	0,10	PA2292 -	0,00	ND	ND	PA4266 fusA1	-0,43	-0,14	0,03
PA0388 -	0,12	-0,02	-0,16	PA2298 -	-0,29	ND	ND	PA4267 rpsG	-0,38	-0,05	0,23
PA0389 -	-0,06	-0,07	-0,14	PA2299 -	0,00	ND	ND	PA4268 rpsL	-0,37	-0,07	0,18
PA0390 metX	0,08	-0,07	-0,28	PA2301 -	-0,13	-0,03	0,03	PA4269 rpoC	-0,13	-0,22	-0,49
PA0391 -	**-1,37**	ND	ND	PA2302 ambE	0,58	0,02	-0,54	PA4270 rpoB	-0,09	-0,19	-0,44
PA0392 -	0,04	ND	ND	PA2303 ambD	0,54	ND	ND	PA4270.1 -	-0,07	ND	ND
PA0393 proC	-0,06	0,07	0,27	PA2304 ambC	0,26	ND	ND	PA4271 rplL	-0,19	0,29	**1,02**
PA0394 -	0,18	0,11	0,14	PA2305 ambB	0,28	ND	ND	PA4272 rplJ	-0,15	-0,05	0,01
PA0395 pilT	-0,99	-0,89	**-1,55**	PA2306 ambA	0,62	ND	ND	PA4272.1 -	-0,06	ND	ND
PA0396 pilU	**-2,01**	**-1,11**	**-1,16**	PA2313 -	0,19	ND	ND	PA4273 rplA	-0,22	-0,07	0,01
PA0397 -	-0,92	ND	ND	PA2316 -	-0,19	ND	ND	PA4274 rplK	-0,35	-0,07	0,16
PA0398 -	-0,43	ND	ND	PA2318 -	-0,08	ND	ND	PA4275 nusG	0,01	-0,18	-0,53
PA0399 -	-0,25	-0,14	-0,13	PA2319 -	-0,04	ND	ND	PA4276 secE	0,14	-0,18	-0,65
PA0400 -	-0,35	-0,10	0,07	PA2320 gntR	**-1,27**	0,31	**2,14**	PA4276.1 -	0,07	ND	ND
PA0401 -	-0,10	0,00	0,10	PA2321 -	**-1,78**	ND	ND	PA4277 tufB	-0,48	ND	ND
PA0402 pyrB	-0,02	-0,04	-0,08	PA2322 -	**-1,00**	ND	ND	PA4277.1 -	0,75	ND	ND
PA0403 pyrR	-0,54	-0,20	-0,03	PA2323 -	-0,47	0,02	0,52	PA4277.2 -	**1,11**	ND	ND
PA0404 -	-0,17	ND	ND	PA2328 -	0,87	ND	ND	PA4277.3 -	**1,26**	ND	ND
PA0405 -	-0,35	ND	ND	PA2329 -	0,87	ND	ND	PA4278 -	-0,27	-0,38	-0,80
PA0406 tonB3	-0,58	-0,43	-0,64	PA2330 -	0,92	ND	ND	PA4279 -	-0,12	-0,34	-0,84
PA0407 gshB	**-1,17**	-0,03	**1,09**	PA2331 -	0,07	ND	ND	PA4280 birA	-0,11	-0,16	-0,35
PA0408 pilG	-0,97	-0,79	**-1,28**	PA2332 -	-0,40	0,23	**1,04**	PA4280.3 -	-0,15	ND	ND
PA0409 pilH	-0,79	-0,86	**-1,65**	PA2337 mtlR	-0,13	ND	ND	PA4280.4 -	0,13	ND	ND
PA0410 pilI	-0,52	-0,53	-0,98	PA2344 mtlZ	0,28	0,23	0,38	PA4281 sbcD	-0,46	ND	ND
PA0411 pilJ	-0,81	-0,92	**-1,81**	PA2345 -	-0,29	ND	ND	PA4282 -	-0,04	-0,04	-0,06
PA0412 pilK	-0,69	ND	ND	PA2352 -	-0,47	-0,45	-0,82	PA4283 recD	0,00	0,31	0,88
PA0413 chpA	-0,57	-0,68	**-1,36**	PA2353 -	0,00	ND	ND	PA4284 recB	0,08	0,36	0,95
PA0414 chpB	-0,66	-0,13	0,29	PA2358 -	**-1,64**	ND	ND	PA4285 recC	0,34	0,12	0,01
PA0415 chpC	-0,64	-0,79	**-1,59**	PA2364 -	-0,74	ND	ND	PA4286 -	0,27	ND	ND
PA0416 chpD	-0,81	ND	ND	PA2365 -	**-1,46**	ND	ND	PA4288 -	-0,65	ND	ND
PA0417 chpE	-0,65	ND	ND	PA2372 -	0,22	ND	ND	PA4289 -	**-2,00**	ND	ND
PA0418 -	-0,22	-0,10	-0,07	PA2373 -	0,78	ND	ND	PA4290 -	**-4,58**	-0,78	**2,35**
PA0419 -	0,46	0,28	0,35	PA2376 -	-0,46	ND	ND	PA4291 -	0,86	ND	ND
PA0420 bioA	0,15	-0,34	**-1,13**	PA2378 -	-0,81	-0,09	0,55	PA4292 -	0,30	0,07	-0,10
PA0421 -	0,23	0,03	-0,15	PA2379 -	-0,64	0,03	0,72	PA4296 pprB	-0,09	0,22	0,70
PA0422 -	-0,26	ND	ND	PA2380 -	0,24	ND	ND	PA4297 tadG	-0,87	ND	ND
PA0423 pasP	-0,26	0,45	**1,55**	PA2381 -	-0,86	ND	ND	PA4306 flp	**-1,52**	ND	ND
PA0424 mexR	-0,18	0,15	0,60	PA2383 -	-0,42	ND	ND	PA4307 pctC	-0,55	-0,10	0,28
PA0425 mexA	0,70	0,62	**1,05**	PA2384 -	**1,81**	ND	ND	PA4308 -	-0,36	0,06	0,52
PA0426 mexB	0,66	0,61	**1,08**	PA2386 pvdA	**1,39**	**3,11**	**7,46**	PA4309 pctA	0,18	0,41	0,99
PA0427 oprM	0,64	0,67	**1,26**	PA2387 fpvI	0,28	ND	ND	PA4310 pctB	0,05	0,15	0,37
PA0428 -	-0,29	-0,04	0,19	PA2388 fpvR	0,22	ND	ND	PA4311 -	-0,65	ND	ND
PA0429 -	-0,14	0,17	0,61	PA2389 pvdR	0,69	ND	ND	PA4312 -	0,34	ND	ND
PA0430 metF	-0,16	0,15	0,59	PA2390 pvdT	0,69	ND	ND	PA4313 -	0,30	ND	ND
PA0431 -	-0,10	ND	ND	PA2391 opmQ	0,47	0,44	0,78	PA4314 purU1	0,18	0,12	0,16
PA0432 sahH	-0,21	-0,05	0,08	PA2396 pvdF	0,58	**2,01**	**5,14**	PA4315 mvaT	-0,15	0,01	0,17
PA0433 -	0,63	ND	ND	PA2397 pvdE	0,65	ND	ND	PA4316 sbcB	-0,44	-0,13	0,07
PA0434 -	**1,38**	ND	ND	PA2398 fpvA	**1,20**	**1,11**	**1,96**	PA4317 -	0,18	0,19	0,37
PA0436 -	-0,81	ND	ND	PA2399 pvdD	**1,14**	0,93	**1,52**	PA4318 -	0,38	ND	ND
PA0437 codA	-0,26	0,04	0,38	PA2400 pvdJ	ND	**1,10**	ND	PA4319 -	0,20	ND	ND
PA0438 codB	0,10	ND	ND	PA2402 -	ND	**1,30**	ND	PA4320 -	0,14	0,11	0,16
PA0445 -	-0,16	ND	ND	PA2403 -	**1,29**	ND	ND	PA4321 -	0,25	-0,01	-0,28
PA0446 -	**1,55**	**1,43**	**2,52**	PA2404 -	**1,24**	ND	ND	PA4322 -	0,22	0,15	0,20
PA0447 gcdH	**2,64**	**1,09**	0,48	PA2405 -	**1,58**	ND	ND	PA4323 -	0,10	-0,02	-0,17

ID – Gene [a]	TC [b]	PC [c]	PTV [d]
PA0448 -	-0,28	ND	ND
PA0449 -	-0,24	-0,06	0,08
PA0450 -	0,28	ND	ND
PA0453 -	**-1,13**	ND	ND
PA0454 -	-0,07	ND	ND
PA0455 dbpA	-0,36	-0,01	0,35
PA0456 -	-0,61	-0,15	0,19
PA0457 -	-0,47	ND	ND
PA0457.1 -	0,06	ND	ND
PA0458 -	-0,47	ND	ND
PA0459 -	ND	-0,25	ND
PA0460 -	0,29	0,01	-0,27
PA0461 -	0,01	-0,02	-0,08
PA0462 -	-0,65	-0,06	0,48
PA0463 creB	-0,78	-0,13	0,41
PA0464 creC	-0,15	-0,06	-0,03
PA0467 -	-0,24	-0,05	0,08
PA0468 -	-0,76	0,02	0,83
PA0469 -	**-1,00**	-0,56	-0,60
PA0470 fiuA	-0,24	**1,05**	**3,22**
PA0471 -	0,09	ND	ND
PA0472 -	0,12	ND	ND
PA0473 -	0,03	0,83	**2,35**
PA0475 -	-0,09	ND	ND
PA0476 -	-0,46	ND	ND
PA0477 -	**-1,25**	ND	ND
PA0478 -	-0,47	ND	ND
PA0479 -	-0,54	ND	ND
PA0481 -	0,33	ND	ND
PA0482 glcB	-0,55	-0,43	-0,68
PA0483 -	**-1,47**	ND	ND
PA0484 -	-0,64	ND	ND
PA0485 -	0,71	ND	ND
PA0486 -	-0,55	-0,24	-0,12
PA0487 -	**-1,14**	-0,01	**1,10**
PA0488 -	-0,11	ND	ND
PA0490 -	-0,18	ND	ND
PA0491 -	-0,72	ND	ND
PA0492 -	**-1,25**	ND	ND
PA0493 -	-0,77	ND	ND
PA0494 -	-0,32	ND	ND
PA0495 -	0,05	ND	ND
PA0496 -	-0,18	ND	ND
PA0497 -	0,32	ND	ND
PA0498 -	-0,81	ND	ND
PA0499 -	-0,78	ND	ND
PA0500 bioB	0,48	0,06	-0,30
PA0501 bioF	0,68	0,08	-0,46
PA0502 -	0,60	0,06	-0,43
PA0503 -	0,72	0,21	-0,13
PA0504 bioD	0,98	0,12	-0,63
PA0505 -	-0,67	ND	ND
PA0506 -	-0,19	-0,16	-0,26
PA0507 -	ND	-0,32	ND
PA0508 -	-0,83	-0,23	0,17
PA0509 nirN	**2,00**	ND	ND
PA0510 -	**2,39**	ND	ND
PA0511 nirJ	**1,77**	ND	ND
PA0512 -	**2,64**	ND	ND
PA0513 -	**2,12**	ND	ND
PA0514 nirL	**2,58**	ND	ND
PA0515 -	**1,58**	ND	ND
PA0516 nirF	**2,36**	ND	ND
PA0517 nirC	**2,43**	ND	ND
PA0518 nirM	**1,69**	ND	ND
PA0519 nirS	0,05	ND	ND
PA0520 nirQ	-0,66	ND	ND
PA0525 -	0,87	ND	ND
PA0526 -	0,24	ND	ND
PA0527 dnr	-0,42	0,08	0,65
PA0527.1 rsmY	0,47	ND	ND
PA0528 -	-0,35	ND	ND
PA0529 -	**-2,00**	ND	ND
PA0530 -	**-1,00**	ND	ND
PA0532 -	-0,92	ND	ND
PA0533 -	-0,13	ND	ND

ID – Gene [a]	TC [b]	PC [c]	PTV [d]
PA2406 -	**1,32**	ND	ND
PA2407 -	**1,84**	ND	ND
PA2408 -	**2,26**	ND	ND
PA2409 -	**1,93**	ND	ND
PA2410 -	**1,79**	0,96	0,94
PA2411 -	**2,50**	ND	ND
PA2412 -	**2,63**	ND	ND
PA2413 pvdH	ND	**1,26**	ND
PA2417 -	0,09	ND	ND
PA2423 -	-0,08	ND	ND
PA2424 pvdL	ND	**1,26**	ND
PA2426 pvdS	**1,44**	ND	ND
PA2432 bexR	-0,32	ND	ND
PA2433 -	**-1,49**	ND	ND
PA2435 -	**-1,10**	ND	ND
PA2436 -	**-1,73**	ND	ND
PA2440 -	**1,95**	ND	ND
PA2441 -	**2,62**	ND	ND
PA2442 gcvT2	**2,63**	**1,70**	**2,20**
PA2443 sdaA	**2,70**	0,72	-0,65
PA2444 glyA2	**2,37**	**1,15**	0,90
PA2445 gcvP2	**2,51**	**1,98**	**3,13**
PA2446 gcvH2	**2,68**	**2,37**	**4,08**
PA2449 -	0,37	-0,10	-0,65
PA2450 -	0,35	0,13	0,02
PA2453 -	-0,72	-0,52	-0,78
PA2454 -	-0,45	ND	ND
PA2455 -	-0,39	ND	ND
PA2456 -	-0,49	ND	ND
PA2457 -	-0,08	ND	ND
PA2458 -	0,54	ND	ND
PA2459 -	0,70	ND	ND
PA2460 -	0,39	ND	ND
PA2461 -	0,84	ND	ND
PA2462 -	0,75	0,26	0,00
PA2463 -	0,50	ND	ND
PA2464 -	-0,22	-0,05	0,09
PA2467 foxR	-0,32	ND	ND
PA2468 foxI	0,08	ND	ND
PA2469 -	-0,54	ND	ND
PA2476 dsbG	-0,31	-0,30	-0,54
PA2477 -	-0,65	ND	ND
PA2478 -	0,00	ND	ND
PA2479 -	-0,55	ND	ND
PA2480 -	-0,64	ND	ND
PA2481 -	-0,92	0,07	**1,12**
PA2482 -	**-1,16**	0,05	**1,31**
PA2483 -	-0,37	0,04	0,48
PA2484 -	-0,05	ND	ND
PA2485 -	0,09	ND	ND
PA2486 -	-0,11	ND	ND
PA2488 -	0,00	ND	ND
PA2489 -	-0,54	ND	ND
PA2490 -	-0,70	ND	ND
PA2491 -	-0,20	-0,27	-0,58
PA2492 mexT	-0,04	ND	ND
PA2496 -	0,00	ND	ND
PA2497 -	-0,13	ND	ND
PA2500 -	0,06	ND	ND
PA2501 -	-0,51	ND	ND
PA2502 -	0,51	ND	ND
PA2503 -	-0,19	-0,05	0,04
PA2504 -	0,13	ND	ND
PA2510 catR	-0,78	ND	ND
PA2523 -	-0,87	ND	ND
PA2524 -	-0,53	ND	ND
PA2525 -	0,69	0,16	-0,23
PA2526 -	0,71	ND	ND
PA2527 -	0,07	ND	ND
PA2528 -	0,18	0,05	-0,04
PA2529 -	-0,11	-0,12	-0,24
PA2530 -	-0,36	0,03	0,44
PA2532 tpx	-0,28	0,21	0,88
PA2533 -	**1,08**	ND	ND
PA2534 -	-0,55	ND	ND
PA2535 -	0,17	-0,41	**-1,35**

ID – Gene [a]	TC [b]	PC [c]	PTV [d]
PA4324 -	-0,19	ND	ND
PA4325 -	-0,17	ND	ND
PA4326 -	-0,33	ND	ND
PA4327 -	-0,30	ND	ND
PA4328 -	-0,38	0,06	0,53
PA4329 pykA	-0,36	-0,17	-0,11
PA4330 -	-0,50	ND	ND
PA4331 -	-0,25	ND	ND
PA4332 -	-0,02	ND	ND
PA4333 -	-0,28	-0,29	-0,54
PA4334 -	0,14	ND	ND
PA4335 -	-0,26	ND	ND
PA4336 -	-0,16	-0,02	0,11
PA4338 -	-0,22	ND	ND
PA4339 -	-0,42	ND	ND
PA4340 -	-0,28	-0,05	0,13
PA4345 -	-0,31	ND	ND
PA4346 -	0,64	ND	ND
PA4347 -	0,71	ND	ND
PA4348 -	-0,04	ND	ND
PA4349 -	-0,09	ND	ND
PA4350 -	**1,29**	ND	ND
PA4351 -	**1,24**	ND	ND
PA4352 -	0,01	-0,23	-0,66
PA4353 -	-0,87	ND	ND
PA4354 -	-0,23	ND	ND
PA4355 -	-0,35	ND	ND
PA4356 xenB	-0,77	-0,64	**-1,06**
PA4357 -	**1,75**	ND	ND
PA4358 -	**1,10**	ND	ND
PA4359 -	0,73	ND	ND
PA4360 -	0,18	0,18	0,32
PA4361 -	-0,17	0,26	0,92
PA4362 -	-0,46	ND	ND
PA4363 iciA	-0,32	0,19	0,86
PA4366 sodB	-0,85	-0,32	-0,05
PA4367 bifA	-0,42	-0,08	0,18
PA4368 -	-0,42	ND	ND
PA4369 -	-0,89	-0,35	-0,10
PA4370 icmP	0,07	0,88	**2,44**
PA4371 -	0,53	0,49	0,86
PA4372 -	0,14	0,37	0,91
PA4373 -	0,27	0,29	0,56
PA4374 -	-0,29	-0,12	-0,05
PA4375 -	-0,06	ND	ND
PA4376 pncB2	-0,08	-0,16	-0,38
PA4377 -	-0,42	ND	ND
PA4378 inaA	-0,05	0,08	0,28
PA4379 -	0,06	ND	ND
PA4380 -	0,06	ND	ND
PA4381 -	-0,16	0,17	0,66
PA4382 -	-0,65	ND	ND
PA4383 -	-0,56	ND	ND
PA4384 -	-0,37	ND	ND
PA4385 groEL	-0,17	-0,16	-0,28
PA4386 groES	-0,08	-0,23	-0,58
PA4387 -	-0,02	0,22	0,63
PA4388 -	-0,35	-0,22	-0,28
PA4389 speA	-0,25	0,04	0,36
PA4390 -	0,17	-0,12	-0,51
PA4391 -	-0,22	ND	ND
PA4392 -	-0,43	ND	ND
PA4393 ampG	-0,23	ND	ND
PA4394 -	-0,17	-0,04	0,05
PA4395 -	0,05	0,13	0,32
PA4396 -	-0,91	ND	ND
PA4397 panE	-0,35	ND	ND
PA4398 -	0,00	ND	ND
PA4399 -	-0,29	0,27	**1,05**
PA4400 -	-0,81	-0,25	0,11
PA4401 -	0,08	ND	ND
PA4402 argJ	0,15	-0,03	-0,22
PA4403 secA	0,01	-0,05	-0,16
PA4404 -	-0,32	ND	ND
PA4405 -	0,32	ND	ND
PA4406 lpxC	0,39	0,06	-0,21

ID – Gene [a]	TC [b]	PC [c]	PTV [d]	ID – Gene [a]	TC [b]	PC [c]	PTV [d]	ID – Gene [a]	TC [b]	PC [c]	PTV [d]
PA0534 -	0,56	ND	ND	PA2536 -	0,53	ND	ND	PA4406.1 -	0,27	ND	ND
PA0535 -	0,35	ND	ND	PA2537 -	0,78	0,17	-0,28	PA4407 ftsZ	0,30	0,35	0,70
PA0536 -	-0,04	0,30	0,90	PA2538 -	0,86	ND	ND	PA4408 ftsA	0,25	0,27	0,53
PA0537 -	-0,21	0,08	0,45	PA2539 -	0,60	ND	ND	PA4409 ftsQ	0,38	0,47	0,95
PA0538 dsbB	-0,66	ND	ND	PA2540 -	0,48	0,13	-0,11	PA4410 ddlB	0,21	0,42	0,98
PA0540 -	-0,37	ND	ND	PA2541 -	0,52	0,03	-0,42	PA4411 murC	0,53	0,35	0,47
PA0541 -	0,39	0,13	-0,02	PA2542 -	0,19	ND	ND	PA4412 murG	0,32	0,39	0,81
PA0542 -	-0,13	-0,14	-0,26	PA2543 -	0,16	ND	ND	PA4413 ftsW	0,24	ND	ND
PA0544 -	-0,58	ND	ND	PA2544 -	-0,36	ND	ND	PA4414 murD	0,46	0,29	0,38
PA0545 -	0,98	ND	ND	PA2545 xthA	-0,47	-0,21	-0,12	PA4415 mraY	0,42	ND	ND
PA0546 metK	-0,31	-0,26	-0,44	PA2546 -	0,00	ND	ND	PA4416 murF	0,36	0,28	0,44
PA0547 -	0,03	ND	ND	PA2547 -	-0,87	ND	ND	PA4417 murE	0,43	0,24	0,25
PA0548 tktA	-0,16	-0,13	-0,20	PA2549 -	-0,72	ND	ND	PA4418 ftsI	0,42	0,11	-0,09
PA0549 -	-0,10	ND	ND	PA2550 -	-0,22	0,21	0,81	PA4419 ftsL	0,31	ND	ND
PA0550 -	-0,25	ND	ND	PA2551 -	0,25	0,12	0,09	PA4420 -	0,45	0,05	-0,32
PA0551 epd	0,10	0,14	0,29	PA2552 -	**3,27**	**1,72**	**1,61**	PA4421 -	0,36	ND	ND
PA0552 pgk	-0,14	-0,14	-0,26	PA2553 -	**4,09**	**2,36**	**2,63**	PA4421.1 rnpB	0,37	ND	ND
PA0553 -	-0,10	ND	ND	PA2554 -	**3,87**	**1,28**	-0,23	PA4422 -	0,04	0,11	0,28
PA0554 -	0,11	ND	ND	PA2555 -	**3,87**	**1,29**	-0,19	PA4423 -	-0,07	0,15	0,48
PA0555 fda	-0,33	-0,22	-0,31	PA2556 -	0,45	ND	ND	PA4424 -	0,49	ND	ND
PA0556 -	-0,12	ND	ND	PA2557 -	**1,78**	ND	ND	PA4425 -	0,20	0,25	0,52
PA0557 -	-0,18	ND	ND	PA2558 -	0,00	ND	ND	PA4426 -	0,04	0,14	0,37
PA0558 -	-0,61	ND	ND	PA2559 -	-0,19	ND	ND	PA4427 sspB	-0,10	-0,02	0,03
PA0559 -	0,12	0,08	0,11	PA2560 -	**1,33**	ND	ND	PA4428 sspA	0,09	-0,06	-0,27
PA0560 -	-0,42	ND	ND	PA2561 -	0,19	ND	ND	PA4429 -	-0,05	-0,04	-0,05
PA0561 -	-0,16	ND	ND	PA2562 -	-0,44	ND	ND	PA4430 -	-0,13	-0,13	-0,23
PA0562 -	-0,10	-0,14	-0,30	PA2563 -	0,29	ND	ND	PA4431 -	0,03	-0,23	-0,67
PA0563 -	-0,26	-0,29	-0,58	PA2567 -	-0,22	ND	ND	PA4432 rpsI	-0,02	-0,15	-0,41
PA0564 -	-0,94	ND	ND	PA2568 -	-0,40	ND	ND	PA4433 rplM	0,07	-0,06	-0,24
PA0565 -	0,00	ND	ND	PA2569 -	-0,67	ND	ND	PA4434 -	-0,05	-0,09	-0,21
PA0566 -	-0,32	ND	ND	PA2570.1 -	-0,12	ND	ND	PA4435 -	-0,35	ND	ND
PA0567 -	-0,14	ND	ND	PA2572 -	**-1,46**	ND	ND	PA4436 -	0,36	ND	ND
PA0568 -	0,00	0,08	0,24	PA2573 -	-0,83	ND	ND	PA4437 -	0,32	ND	ND
PA0569 -	-0,08	ND	ND	PA2575 -	-0,44	0,08	0,68	PA4438 -	0,15	-0,07	-0,35
PA0570 -	-0,53	ND	ND	PA2576 -	-0,17	ND	ND	PA4439 trpS	-0,07	-0,12	-0,28
PA0571 -	-0,20	-0,15	-0,23	PA2577 -	**-1,00**	ND	ND	PA4440 -	-0,64	-0,06	0,48
PA0572 -	**-2,35**	ND	ND	PA2578 -	0,00	ND	ND	PA4441 -	0,70	0,01	-0,67
PA0573 -	**-1,47**	ND	ND	PA2579 kynA	0,51	0,31	0,37	PA4442 cysN	0,85	0,35	0,16
PA0574 -	-0,60	ND	ND	PA2580 -	0,30	ND	ND	PA4443 cysD	0,74	0,35	0,26
PA0574.1 -	0,83	ND	ND	PA2581 -	-0,12	0,04	0,23	PA4444 mltB1	-0,23	0,54	**1,77**
PA0576 rpoD	0,01	-0,12	-0,35	PA2581.1 -	**1,27**	ND	ND	PA4445 -	-0,05	-0,10	-0,22
PA0577 dnaG	0,27	0,12	0,06	PA2582 -	-0,27	-0,18	-0,26	PA4446 algW	0,80	0,09	-0,54
PA0578 -	0,58	0,16	-0,13	PA2583 -	0,00	0,33	0,93	PA4447 hisC1	0,00	-0,24	-0,68
PA0579 rpsU	0,70	-0,07	-0,90	PA2583.1 -	0,99	ND	ND	PA4448 hisD	-0,23	-0,24	-0,45
PA0580 gcp	0,38	-0,11	-0,70	PA2584 pgsA	0,53	ND	ND	PA4449 hisG	0,00	-0,01	-0,03
PA0581 -	-0,15	ND	ND	PA2585 uvrC	0,25	ND	ND	PA4450 murA	-0,07	-0,11	-0,24
PA0582 folB	0,39	0,02	-0,33	PA2586 gacA	0,09	0,01	-0,05	PA4451 -	0,28	ND	ND
PA0583 -	0,30	0,28	0,49	PA2587 pqsH	-0,49	-0,44	-0,76	PA4451.1 -	0,15	ND	ND
PA0584 cca	-0,29	0,11	0,60	PA2588 -	-0,72	ND	ND	PA4452 -	0,31	0,03	-0,21
PA0586 -	-0,24	ND	ND	PA2591 -	-0,13	ND	ND	PA4453 -	0,33	0,25	0,38
PA0587 -	-0,69	ND	ND	PA2592 -	-0,16	-0,21	-0,42	PA4454 -	0,29	0,13	0,07
PA0588 -	-0,50	-0,27	-0,27	PA2593 qteE	-0,24	ND	ND	PA4455 -	0,39	0,15	0,02
PA0589 -	-0,08	ND	ND	PA2594 -	-0,32	ND	ND	PA4456 -	0,52	0,09	-0,26
PA0590 apaH	-0,16	-0,08	-0,06	PA2601 -	-0,79	-0,13	0,42	PA4457 -	-0,02	-0,06	-0,16
PA0591 -	-0,18	-0,02	0,14	PA2602 -	**-1,00**	ND	ND	PA4458 -	0,08	0,08	0,16
PA0592 ksgA	0,32	0,07	-0,13	PA2603 -	-0,22	ND	ND	PA4459 -	0,09	-0,03	-0,18
PA0593 pdxA	0,00	-0,01	-0,03	PA2603.1 -	-0,14	ND	ND	PA4460 -	0,13	0,04	-0,02
PA0594 surA	0,02	0,14	0,37	PA2604 -	-0,39	ND	ND	PA4461 -	0,12	0,05	0,01
PA0595 ostA	-0,04	-0,15	-0,38	PA2605 -	-0,52	-0,15	0,10	PA4462 rpoN	0,03	0,00	-0,02
PA0596 -	0,04	0,07	0,16	PA2606 -	-0,52	ND	ND	PA4463 -	-0,43	ND	ND
PA0597 -	-0,05	-0,10	-0,23	PA2607 -	-0,05	0,14	0,44	PA4464 ptsN	0,07	0,32	0,84
PA0598 -	0,14	ND	ND	PA2608 -	-0,26	ND	ND	PA4465 -	0,31	0,06	-0,13
PA0599 -	-0,23	ND	ND	PA2609 -	-0,03	-0,21	-0,57	PA4466 -	0,40	0,15	0,03
PA0600 -	0,05	ND	ND	PA2610 -	-0,18	ND	ND	PA4468 sodM	-0,53	ND	ND
PA0601 -	-0,22	0,14	0,62	PA2611 cysG	0,11	0,04	0,00	PA4470 fumC1	-0,24	**1,30**	**3,95**
PA0602 -	-0,89	ND	ND	PA2612 serS	-0,02	0,02	0,07	PA4472 pmbA	-0,23	0,01	0,25
PA0603 -	**-6,40**	-0,12	**6,07**	PA2613 -	0,24	0,09	0,02	PA4473 -	0,19	0,15	0,23
PA0604 -	**-5,35**	0,05	**5,50**	PA2614 lolA	0,13	0,17	0,36	PA4474 -	-0,05	-0,02	-0,02
PA0605 -	**-5,14**	0,08	**5,36**	PA2615 ftsK	0,00	0,00	-0,01	PA4475 -	0,09	-0,03	-0,18
PA0606 -	**-4,67**	ND	ND	PA2616 trxB1	-0,40	-0,20	-0,16	PA4476 -	0,04	-0,12	-0,40
PA0607 rpe	0,21	0,12	0,13	PA2617 aat	0,24	ND	ND	PA4477 cafA	-0,34	0,10	0,62
PA0608 -	0,42	ND	ND	PA2618 -	0,08	ND	ND	PA4478 -	-0,11	0,16	0,55
PA0609 trpE	0,21	0,17	0,28	PA2619 infA	0,77	0,05	-0,63	PA4479 mreD	0,24	ND	ND
PA0610 prtN	-0,18	ND	ND	PA2620 clpA	0,02	0,00	-0,01	PA4480 mreC	0,19	0,01	-0,17
PA0611 prtR	-0,60	ND	ND	PA2621 -	-0,13	-0,09	-0,11	PA4481 mreB	0,21	0,00	-0,23
PA0612 ptrB	-0,06	ND	ND	PA2622 cspD	-0,85	-0,49	-0,54	PA4482 gatC	-0,11	0,26	0,84

ID – Gene [a]	TC [b]	PC [c]	PTV [d]
PA0613 -	-0,23	ND	ND
PA0614 -	-0,52	ND	ND
PA0615 -	-0,63	ND	ND
PA0616 -	-0,81	ND	ND
PA0617 -	-0,55	ND	ND
PA0618 -	-0,53	-0,26	-0,21
PA0619 -	-0,50	ND	ND
PA0620 -	-0,53	ND	ND
PA0621 -	-0,46	ND	ND
PA0622 -	-0,59	-0,68	**-1,36**
PA0623 -	-0,64	-0,49	-0,77
PA0624 -	-0,66	ND	ND
PA0625 -	-0,80	ND	ND
PA0626 -	-0,50	ND	ND
PA0627 -	-0,50	ND	ND
PA0628 -	-0,42	ND	ND
PA0629 -	-0,63	ND	ND
PA0630 -	-0,49	ND	ND
PA0631 -	-0,37	ND	ND
PA0632 -	-0,58	ND	ND
PA0633 -	-0,86	-0,70	**-1,13**
PA0634 -	-0,98	-0,51	-0,46
PA0635 -	-0,97	ND	ND
PA0636 -	-0,69	ND	ND
PA0637 -	-0,78	ND	ND
PA0638 -	-0,97	ND	ND
PA0639 -	-0,77	ND	ND
PA0640 -	-0,58	ND	ND
PA0641 -	-0,64	ND	ND
PA0642 -	-0,62	ND	ND
PA0643 -	-0,60	ND	ND
PA0644 -	-0,82	ND	ND
PA0645 -	-0,65	ND	ND
PA0646 -	-0,90	ND	ND
PA0647 -	-0,79	ND	ND
PA0648 -	-0,73	ND	ND
PA0649 trpG	-0,14	0,17	0,61
PA0650 trpD	-0,23	-0,04	0,11
PA0651 trpC	-0,28	-0,04	0,18
PA0652 vfr	**-1,60**	-0,69	-0,35
PA0653 -	-0,20	0,00	0,21
PA0654 speD	0,26	-0,21	-0,87
PA0655 -	-0,74	-0,05	0,59
PA0656 -	-0,80	ND	ND
PA0657 -	-0,54	ND	ND
PA0658 -	-0,42	-0,37	-0,65
PA0659 -	-0,49	-0,53	**-1,00**
PA0660 -	-0,56	0,03	0,64
PA0661 -	-0,69	ND	ND
PA0662 argC	-0,12	-0,21	-0,47
PA0663 -	-0,41	-0,27	-0,35
PA0664 -	-0,44	-0,49	-0,96
PA0665 -	-0,27	0,17	0,76
PA0666 -	-0,18	0,24	0,87
PA0667 -	-0,23	ND	ND
PA0668 tyrZ	-0,27	-0,18	-0,25
PA0668.2 -	0,09	ND	ND
PA0668.3 -	-0,12	ND	ND
PA0668.5 -	0,64	ND	ND
PA0670 -	0,00	ND	ND
PA0672 hemO	0,51	**2,55**	**6,75**
PA0673 -	-0,35	ND	ND
PA0688 -	**1,12**	ND	ND
PA0689 -	0,31	ND	ND
PA0703 -	0,29	ND	ND
PA0704 -	0,09	ND	ND
PA0705 migA	0,18	0,12	0,16
PA0706 cat	0,09	ND	ND
PA0708 -	0,05	ND	ND
PA0709 -	0,28	ND	ND
PA0710 gloA2	-0,29	ND	ND
PA0712 -	0,19	ND	ND
PA0713 -	**-2,97**	ND	ND
PA0714.1 phrD	0,00	ND	ND
PA0715 -	0,22	0,14	0,16
PA0716 -	0,30	ND	ND

ID – Gene [a]	TC [b]	PC [c]	PTV [d]
PA2623 icd	-0,62	-0,52	-0,87
PA2624 idh	-0,88	-0,36	-0,16
PA2625 -	-0,33	ND	ND
PA2626 trmU	0,06	0,03	0,03
PA2627 -	0,06	0,06	0,10
PA2628 -	-0,24	ND	ND
PA2629 purB	0,03	-0,11	-0,34
PA2630 -	-0,07	-0,24	-0,62
PA2631 -	-0,17	-0,05	0,02
PA2632 -	-0,46	ND	ND
PA2633 -	-0,63	ND	ND
PA2634 aceA	-0,21	-0,25	-0,50
PA2637 nuoA	0,13	-0,08	-0,35
PA2638 nuoB	-0,10	-0,06	-0,07
PA2639 nuoD	-0,14	-0,02	0,10
PA2640 nuoE	-0,14	-0,03	0,05
PA2641 nuoF	0,01	0,04	0,11
PA2642 nuoG	-0,05	-0,02	0,00
PA2643 nuoH	0,01	0,02	0,05
PA2644 nuoI	0,12	0,07	0,08
PA2645 nuoJ	0,22	ND	ND
PA2646 nuoK	0,08	ND	ND
PA2647 nuoL	0,22	-0,05	-0,35
PA2648 nuoM	0,14	0,14	0,26
PA2649 nuoN	0,31	ND	ND
PA2650 -	0,08	ND	ND
PA2651 -	-0,81	ND	ND
PA2652 -	-0,65	-0,48	-0,73
PA2653 -	-0,29	ND	ND
PA2654 -	-0,57	-0,14	0,17
PA2655 -	-0,35	ND	ND
PA2656 -	0,17	ND	ND
PA2657 -	-0,42	-0,05	0,28
PA2658 -	-0,57	ND	ND
PA2659 -	-0,58	ND	ND
PA2660 -	-0,26	-0,24	-0,44
PA2661 -	-0,08	ND	ND
PA2662 -	-0,42	ND	ND
PA2663 ppyR	-0,81	ND	ND
PA2665 -	-0,16	-0,09	-0,09
PA2666 -	0,15	ND	ND
PA2667 -	-0,31	0,11	0,63
PA2668 -	-0,31	ND	ND
PA2678 -	**-1,70**	ND	ND
PA2679 -	**-3,12**	0,04	**3,23**
PA2681 -	-0,78	ND	ND
PA2682 -	-0,46	ND	ND
PA2683 -	-0,18	ND	ND
PA2684 -	-0,21	-0,02	0,16
PA2685 -	-0,30	ND	ND
PA2686 pfeR	-0,13	ND	ND
PA2687 pfeS	-0,13	ND	ND
PA2690 -	0,22	ND	ND
PA2691 -	**-1,14**	0,15	**1,56**
PA2692 -	-0,11	-0,17	-0,38
PA2693 -	0,00	ND	ND
PA2694 -	0,14	ND	ND
PA2695 -	-0,06	ND	ND
PA2698 -	-0,32	ND	ND
PA2702 -	-0,14	ND	ND
PA2703 -	-0,64	ND	ND
PA2704 -	-0,58	ND	ND
PA2705 -	-0,87	-0,06	0,69
PA2706 -	-0,47	ND	ND
PA2707 -	-0,87	-0,26	0,15
PA2709 cysK	**-1,08**	-0,35	0,08
PA2710 -	-0,43	ND	ND
PA2712 -	**1,49**	ND	ND
PA2713 -	-0,28	ND	ND
PA2715 -	-0,87	ND	ND
PA2718 -	-0,94	ND	ND
PA2720 -	-0,09	0,22	0,72
PA2723 -	-0,82	ND	ND
PA2725 -	-0,38	-0,35	-0,63
PA2726 -	-0,40	ND	ND
PA2727 -	-0,29	0,07	0,49

ID – Gene [a]	TC [b]	PC [c]	PTV [d]
PA4483 gatA	-0,17	0,07	0,36
PA4484 gatB	-0,11	0,03	0,21
PA4485 -	0,37	ND	ND
PA4486 -	0,24	ND	ND
PA4487 -	0,20	0,30	0,66
PA4488 -	0,25	ND	ND
PA4489 -	0,12	0,13	0,24
PA4490 -	0,29	ND	ND
PA4491 -	0,20	0,15	0,24
PA4492 -	0,20	ND	ND
PA4493 roxR	0,01	0,05	0,13
PA4494 roxS	-0,07	0,32	0,97
PA4495 -	-0,17	-0,17	-0,31
PA4496 -	**1,45**	**1,72**	**3,44**
PA4497 -	**1,29**	ND	ND
PA4498 -	**1,23**	**1,70**	**3,62**
PA4499 -	**1,19**	0,47	0,16
PA4500 -	**3,37**	**2,33**	**3,27**
PA4501 opdP	**4,52**	**2,52**	**2,66**
PA4502 -	**4,52**	**2,58**	**2,83**
PA4503 -	**4,43**	**2,30**	**2,12**
PA4504 -	**4,85**	**2,37**	**1,88**
PA4505 -	**4,61**	**2,35**	**2,08**
PA4506 -	**4,55**	**2,16**	**1,59**
PA4507 -	**1,22**	ND	ND
PA4508 -	0,33	ND	ND
PA4509 -	-0,09	ND	ND
PA4510 -	-0,18	ND	ND
PA4511 -	-0,25	ND	ND
PA4512 lpxO1	-0,71	-0,02	0,65
PA4513 -	0,66	0,84	**1,73**
PA4514 -	0,87	**1,99**	**4,80**
PA4515 -	0,73	0,93	**1,91**
PA4516 -	0,28	ND	ND
PA4517 -	-0,07	ND	ND
PA4518 -	0,00	ND	ND
PA4519 speC	0,47	0,23	0,17
PA4520 -	0,30	0,15	0,12
PA4521 -	-0,37	-0,02	0,32
PA4522 ampD	-0,66	ND	ND
PA4523 -	**-1,61**	ND	ND
PA4524 nadC	-0,10	0,00	0,09
PA4524.1 -	-0,24	ND	ND
PA4525 pilA	-0,26	-0,62	**-1,50**
PA4526 pilB	-0,66	-0,42	-0,55
PA4528 pilD	**-1,84**	ND	ND
PA4529 coaE	-0,87	-0,20	0,30
PA4530 -	-0,89	ND	ND
PA4531 -	-0,17	ND	ND
PA4532 -	0,40	ND	ND
PA4533 -	0,22	0,04	-0,09
PA4534 -	0,41	ND	ND
PA4535 -	-0,98	ND	ND
PA4536 -	-0,64	ND	ND
PA4537 -	-0,08	ND	ND
PA4538 ndh	-0,66	-0,17	0,19
PA4539 -	0,23	ND	ND
PA4541 -	-0,24	ND	ND
PA4541.1 -	**1,87**	ND	ND
PA4541.2 -	**2,03**	ND	ND
PA4541.3 -	**2,23**	ND	ND
PA4542 clpB	0,24	-0,14	-0,62
PA4543 -	0,23	ND	ND
PA4544 rluD	0,32	0,18	0,20
PA4545 comL	0,27	0,05	-0,13
PA4546 pilS	0,22	ND	ND
PA4547 pilR	-0,09	-0,17	-0,39
PA4548 -	-0,97	-0,42	-0,23
PA4550 fimU	**-2,27**	ND	ND
PA4551 pilV	**-2,00**	ND	ND
PA4552 pilW	**-2,06**	ND	ND
PA4553 pilX	**-1,85**	**-1,12**	**-1,32**
PA4554 pilY1	**-1,70**	**-1,38**	**-2,22**
PA4555 pilY2	**-1,52**	ND	ND
PA4556 pilE	**-1,37**	-0,95	**-1,32**
PA4557 lytB	0,42	0,16	0,03

ID – Gene [a]	TC [b]	PC [c]	PTV [d]	ID – Gene [a]	TC [b]	PC [c]	PTV [d]	ID – Gene [a]	TC [b]	PC [c]	PTV [d]
PA0717 -	0,24	ND	ND	PA2728 -	-0,44	ND	ND	PA4558 -	0,25	0,18	0,26
PA0718 -	-0,58	ND	ND	PA2729 -	-0,35	ND	ND	PA4559 lspA	0,25	ND	ND
PA0719 -	-0,29	ND	ND	PA2730 -	-0,10	0,16	0,54	PA4560 ileS	0,13	0,07	0,07
PA0720 -	-0,57	ND	ND	PA2731 -	0,01	ND	ND	PA4561 ribF	0,27	0,05	-0,14
PA0721 -	-0,14	ND	ND	PA2732 -	-0,04	-0,28	-0,75	PA4562 -	0,29	ND	ND
PA0722 -	-0,23	ND	ND	PA2733 -	-0,10	ND	ND	PA4563 rpsT	0,47	-0,14	-0,85
PA0723 coaB	0,19	ND	ND	PA2734 -	-0,10	-0,25	-0,62	PA4564 -	0,18	0,12	0,15
PA0727 -	-0,24	ND	ND	PA2735 -	-0,20	-0,23	-0,46	PA4565 proB	0,07	0,12	0,26
PA0728 -	0,00	ND	ND	PA2736 -	0,26	ND	ND	PA4566 obg	-0,19	-0,10	-0,09
PA0729 -	0,12	0,17	0,36	PA2736.1 -	**1,20**	ND	ND	PA4567 rpmA	-0,16	-0,14	-0,25
PA0729.1 -	0,75	ND	ND	PA2737 -	-0,40	0,50	**1,82**	PA4568 rplU	-0,06	-0,11	-0,26
PA0730 -	0,09	ND	ND	PA2738 himA	-0,54	-0,34	-0,42	PA4569 ispB	0,26	0,02	-0,20
PA0731 -	0,56	ND	ND	PA2739 pheT	-0,20	-0,13	-0,18	PA4570 -	-0,29	ND	ND
PA0732 -	-0,49	ND	ND	PA2740 pheS	-0,16	-0,15	-0,26	PA4571 -	0,49	-0,15	-0,91
PA0733 -	-0,30	ND	ND	PA2741 rplT	-0,02	-0,05	-0,13	PA4572 fklB	-0,38	-0,34	-0,60
PA0734 -	-0,08	ND	ND	PA2742 rpmI	-0,14	-0,03	0,06	PA4573 -	0,53	ND	ND
PA0735 -	0,00	ND	ND	PA2743 infC	-0,01	0,04	0,13	PA4574 -	0,70	ND	ND
PA0736 -	-0,04	ND	ND	PA2744 thrS	-0,10	-0,18	-0,42	PA4575 -	-0,43	ND	ND
PA0739 -	-0,32	ND	ND	PA2744.1 -	-0,46	ND	ND	PA4576 -	-0,08	-0,01	0,04
PA0741 -	**-1,22**	0,00	**1,21**	PA2745 -	-0,17	-0,25	-0,54	PA4577 -	-0,99	-0,32	0,09
PA0742 -	**-3,39**	ND	ND	PA2746 -	-0,70	ND	ND	PA4578 -	-0,08	ND	ND
PA0743 -	0,65	ND	ND	PA2747 -	0,19	ND	ND	PA4579 -	-0,34	-0,18	-0,16
PA0744 -	**1,86**	**1,28**	**1,78**	PA2748 -	-0,49	ND	ND	PA4580 -	-0,17	ND	ND
PA0745 -	**1,65**	0,79	0,60	PA2749 endA	0,10	ND	ND	PA4581 rtcR	-0,25	ND	ND
PA0746 -	**1,65**	0,92	0,97	PA2750 -	-0,16	ND	ND	PA4581.1 -	-0,01	ND	ND
PA0747 -	0,47	ND	ND	PA2752 -	0,22	ND	ND	PA4582 -	-0,25	0,06	0,40
PA0749 -	0,58	ND	ND	PA2753 -	**-1,65**	ND	ND	PA4583 -	-0,50	-0,21	-0,11
PA0750 ung	0,23	-0,03	-0,31	PA2754 -	-0,40	ND	ND	PA4584 -	-0,27	ND	ND
PA0751 -	0,22	ND	ND	PA2755 eco	-0,87	-0,48	-0,48	PA4585 rtcA	-0,15	0,32	**1,06**
PA0752 -	0,28	ND	ND	PA2756 -	-0,49	ND	ND	PA4586 -	0,18	ND	ND
PA0753 -	0,78	ND	ND	PA2757 -	-0,38	ND	ND	PA4587 ccpR	0,37	ND	ND
PA0754 -	0,06	**2,80**	**7,90**	PA2758 -	**-1,08**	ND	ND	PA4588 gdhA	-0,42	ND	ND
PA0755 opdH	-0,50	**2,54**	**7,72**	PA2759 -	**-2,65**	ND	ND	PA4589 -	0,65	ND	ND
PA0756 -	-0,04	0,36	**1,07**	PA2760 -	0,33	0,89	**2,21**	PA4590 pra	-0,06	ND	ND
PA0757 -	-0,13	ND	ND	PA2761 -	-0,96	ND	ND	PA4592 -	0,00	ND	ND
PA0758 -	-0,61	-0,62	**-1,15**	PA2762 -	-0,95	ND	ND	PA4593 -	-0,29	ND	ND
PA0759 -	-0,05	0,19	0,60	PA2763 -	-0,78	ND	ND	PA4594 -	-0,55	ND	ND
PA0760 -	0,58	ND	ND	PA2764 -	-0,44	ND	ND	PA4595 -	-0,30	-0,24	-0,37
PA0761 nadB	0,16	0,23	0,51	PA2765 -	-0,20	-0,38	-0,87	PA4596 -	-0,49	ND	ND
PA0762 algU	0,38	0,19	0,18	PA2766 -	-0,50	ND	ND	PA4597 oprJ	0,13	ND	ND
PA0763 mucA	0,40	0,49	0,99	PA2767 -	-0,46	ND	ND	PA4598 mexD	-0,46	ND	ND
PA0764 mucB	0,49	0,28	0,30	PA2768 -	-0,25	ND	ND	PA4600 nfxB	-0,28	ND	ND
PA0765 mucC	0,73	ND	ND	PA2769 -	-0,44	ND	ND	PA4601 morA	0,00	0,35	**1,00**
PA0766 mucD	0,72	0,45	0,57	PA2770 -	-0,70	-0,60	-1,00	PA4602 glyA3	0,01	-0,12	-0,34
PA0767 lepA	0,29	0,30	0,56	PA2771 -	0,10	ND	ND	PA4603 -	**-1,16**	ND	ND
PA0768 lepB	0,20	0,20	0,38	PA2772 -	0,41	ND	ND	PA4604 -	**-1,24**	-0,50	-0,19
PA0769 -	0,14	-0,40	**-1,29**	PA2773 -	0,07	ND	ND	PA4605 -	**-1,21**	ND	ND
PA0770 rnc	0,04	0,07	0,17	PA2774 -	-0,13	-0,11	-0,17	PA4606 -	**-1,52**	-0,19	0,98
PA0771 era	0,08	0,27	0,69	PA2775 -	0,07	ND	ND	PA4607 -	-0,79	ND	ND
PA0772 recO	0,11	ND	ND	PA2776 -	**-1,55**	0,20	**2,13**	PA4608 -	-0,47	ND	ND
PA0773 pdxJ	0,36	-0,64	**-2,17**	PA2777 -	-0,11	ND	ND	PA4609 radA	0,18	-0,04	-0,29
PA0774 -	0,35	0,20	0,21	PA2778 -	0,43	ND	ND	PA4610 -	-0,75	ND	ND
PA0775 -	0,72	0,19	-0,18	PA2779 -	0,48	ND	ND	PA4611 -	-1,00	ND	ND
PA0777 -	0,07	ND	ND	PA2780 -	**-1,42**	ND	ND	PA4612 -	-0,46	ND	ND
PA0778 icp	0,16	0,39	0,95	PA2781 -	**-1,00**	ND	ND	PA4613 katB	0,68	0,74	**1,43**
PA0779 -	0,28	0,02	-0,22	PA2788 -	**-1,14**	ND	ND	PA4614 mscL	-0,62	0,03	0,71
PA0780 pruR	0,00	ND	ND	PA2789 -	-0,11	ND	ND	PA4615 -	-0,70	0,30	**1,57**
PA0782 putA	**-3,29**	**1,79**	**8,38**	PA2790 -	-0,75	ND	ND	PA4616 -	**-2,84**	**-2,29**	**-3,68**
PA0783 putP	-0,79	**1,58**	**5,30**	PA2791 -	-0,22	ND	ND	PA4617 -	-0,03	0,10	0,32
PA0784 -	-0,63	ND	ND	PA2792 -	-0,07	ND	ND	PA4618 -	-0,84	-0,14	0,45
PA0785 azoR1	0,00	ND	ND	PA2793 -	0,02	-0,23	-0,67	PA4619 -	**-1,38**	-0,48	0,03
PA0787 -	0,22	ND	ND	PA2794 -	-0,54	ND	ND	PA4620 -	**-1,74**	-0,98	**-1,05**
PA0789 -	0,38	0,86	**2,08**	PA2795 -	0,00	-0,20	-0,57	PA4621 -	**-1,66**	-0,53	0,16
PA0790 -	0,24	ND	ND	PA2796 tal	-0,77	-0,33	-0,16	PA4622 -	0,00	ND	ND
PA0791 -	0,43	ND	ND	PA2797 -	0,27	-0,05	-0,41	PA4623 -	0,29	ND	ND
PA0792 prpD	0,86	ND	ND	PA2798 -	0,39	-0,06	-0,56	PA4624 -	0,50	ND	ND
PA0793 -	**1,08**	ND	ND	PA2799 -	-0,31	ND	ND	PA4625 -	0,93	ND	ND
PA0794 -	0,90	0,53	0,62	PA2800 -	-0,11	-0,22	-0,53	PA4626 hprA	-0,37	-0,01	0,33
PA0795 prpC	0,98	**1,01**	**1,89**	PA2801 -	-0,44	ND	ND	PA4627 -	-0,19	-0,22	-0,43
PA0796 prpB	0,51	0,31	0,38	PA2802 -	-0,28	ND	ND	PA4628 lysP	-0,37	0,14	0,78
PA0797 -	0,75	ND	ND	PA2805 -	**-1,08**	-0,02	**1,03**	PA4629 -	0,30	ND	ND
PA0798 pmtA	0,00	ND	ND	PA2806 -	0,24	-0,12	-0,58	PA4630 -	0,32	ND	ND
PA0799 -	-0,36	0,17	0,85	PA2808 ptrA	0,63	ND	ND	PA4631 -	0,35	0,05	-0,21
PA0800 -	**1,24**	ND	ND	PA2809 copR	-0,42	ND	ND	PA4632 -	0,12	-0,06	-0,29
PA0801 -	0,40	ND	ND	PA2810 copS	0,19	ND	ND	PA4633 -	-0,05	0,32	0,95
PA0802 -	0,45	ND	ND	PA2811 -	-0,09	ND	ND	PA4634 -	-0,10	ND	ND

ID – Gene [a]	TC [b]	PC [c]	PTV [d]	ID – Gene [a]	TC [b]	PC [c]	PTV [d]	ID – Gene [a]	TC [b]	PC [c]	PTV [d]
PA0803 -	0,44	ND	ND	PA2812 -	0,00	-0,27	-0,78	PA4635 -	-0,65	ND	ND
PA0804 -	-0,12	ND	ND	PA2813 -	-0,43	-0,14	0,03	PA4636 -	0,40	0,12	-0,07
PA0805 -	-0,54	ND	ND	PA2815 -	-0,91	-0,31	0,04	PA4637 -	0,14	ND	ND
PA0807 ampDh3	-0,53	ND	ND	PA2816 -	-0,94	ND	ND	PA4638 -	0,12	ND	ND
PA0808 -	0,16	ND	ND	PA2817 -	-0,20	-0,36	-0,82	PA4639 -	0,43	0,24	0,25
PA0809 -	-0,45	ND	ND	PA2818 arr	-0,06	ND	ND	PA4640 mqoB	-0,56	-0,21	-0,04
PA0810 -	-0,22	ND	ND	PA2819 -	0,99	ND	ND	PA4642 -	0,47	0,21	0,12
PA0811 -	-0,53	ND	ND	PA2819.1 -	**1,18**	ND	ND	PA4643 -	0,00	ND	ND
PA0812 -	-0,78	ND	ND	PA2819.2 -	**1,27**	ND	ND	PA4644 -	-0,43	-0,51	**-1,02**
PA0813 -	-0,13	ND	ND	PA2819.3 -	**1,31**	ND	ND	PA4645 -	-0,32	-0,73	**-1,76**
PA0815 -	**-1,03**	ND	ND	PA2820 -	-0,50	-0,16	0,06	PA4646 upp	-0,05	-0,01	0,03
PA0816 -	0,20	ND	ND	PA2821 -	-0,37	-0,37	-0,69	PA4647 uraA	-0,14	ND	ND
PA0817 -	-0,55	ND	ND	PA2822 -	-0,35	-0,05	0,20	PA4655 hemH	-0,08	-0,13	-0,30
PA0818 -	-0,93	ND	ND	PA2823 -	-0,49	-0,22	-0,14	PA4656 -	-0,07	ND	ND
PA0819 -	-0,58	ND	ND	PA2824 -	-0,58	ND	ND	PA4657 -	-0,33	ND	ND
PA0820 -	-0,14	ND	ND	PA2825 ospR	-0,31	ND	ND	PA4658 -	-0,14	ND	ND
PA0821 -	0,20	ND	ND	PA2826 -	-0,72	-0,16	0,26	PA4659 -	0,00	ND	ND
PA0822 -	0,12	ND	ND	PA2827 -	-0,76	ND	ND	PA4660 phr	-0,38	ND	ND
PA0823 -	0,30	ND	ND	PA2828 -	0,14	0,02	-0,10	PA4661 pagL	-0,33	-0,27	-0,44
PA0824 -	0,09	ND	ND	PA2829 -	0,31	-0,07	-0,51	PA4662 murI	0,42	0,24	0,27
PA0826 -	-0,06	ND	ND	PA2830 htpX	-0,01	0,09	0,27	PA4663 moeB	0,27	0,07	-0,07
PA0826.1 -	0,48	ND	ND	PA2831 -	-0,87	-0,47	-0,46	PA4664 hemK	0,23	0,18	0,29
PA0826.2 ssrA	0,40	ND	ND	PA2832 tpm	-0,88	ND	ND	PA4665 prfA	0,10	0,21	0,50
PA0827 -	-0,55	ND	ND	PA2834 -	-0,50	ND	ND	PA4666 hemA	0,42	0,15	0,02
PA0830 -	-0,25	ND	ND	PA2840 -	-0,90	-0,36	-0,12	PA4667 -	0,03	0,09	0,22
PA0831 oruR	-0,11	ND	ND	PA2841 -	-0,81	ND	ND	PA4668 -	-0,06	0,18	0,58
PA0832 -	-0,13	-0,15	-0,31	PA2842 -	-0,54	ND	ND	PA4669 ipk	**1,13**	0,13	-0,76
PA0833 -	0,65	0,18	-0,15	PA2843 -	-0,37	-0,20	-0,20	PA4669.1 -	**1,05**	ND	ND
PA0834 -	0,63	0,57	0,99	PA2844 -	**-1,17**	ND	ND	PA4670 prs	0,77	0,29	0,04
PA0835 pta	0,87	-0,09	**-1,12**	PA2846 -	-0,92	ND	ND	PA4671 -	-0,02	-0,08	-0,22
PA0836 ackA	0,05	-0,07	-0,25	PA2847 -	**-1,36**	ND	ND	PA4672 -	0,42	0,13	-0,04
PA0836.1 -	-0,43	ND	ND	PA2849 ohrR	-0,49	0,34	**1,46**	PA4673 -	0,42	0,09	-0,17
PA0837 slyD	0,09	0,22	0,55	PA2850 ohr	0,00	**1,61**	**4,58**	PA4673.1 -	**3,70**	ND	ND
PA0838 -	0,25	-0,01	-0,28	PA2851 efp	-0,26	-0,24	-0,42	PA4674 -	0,48	ND	ND
PA0839 -	-0,54	ND	ND	PA2852 -	0,00	ND	ND	PA4675 -	-0,11	**1,03**	**3,03**
PA0840 -	-0,78	0,04	0,89	PA2852.1 -	**1,03**	ND	ND	PA4676 -	-0,25	-0,12	-0,09
PA0841 -	0,70	ND	ND	PA2853 oprI	-0,08	-0,08	-0,14	PA4677 -	-0,07	ND	ND
PA0842 -	0,89	ND	ND	PA2854 -	-0,07	-0,09	-0,18	PA4678 rimI	0,30	ND	ND
PA0846 -	0,39	ND	ND	PA2855 -	-0,04	ND	ND	PA4679 -	0,00	-0,09	-0,25
PA0847 -	-0,29	ND	ND	PA2856 tesA	-0,61	-0,11	0,29	PA4683 -	0,00	ND	ND
PA0848 -	0,89	**1,05**	**2,08**	PA2857 -	-0,31	0,04	0,42	PA4684 -	0,32	0,67	**1,59**
PA0849 trxB2	0,90	0,73	**1,17**	PA2858 -	-0,44	ND	ND	PA4685 -	**3,62**	**1,67**	**1,11**
PA0850 -	0,58	ND	ND	PA2859 greB	0,05	0,04	0,06	PA4686 -	0,03	0,47	**1,31**
PA0851 -	0,33	0,40	0,80	PA2860 -	-0,12	ND	ND	PA4687 hitA	0,05	0,11	0,27
PA0852 cbpD	-0,48	-0,29	-0,35	PA2862 lipA	**-1,87**	ND	ND	PA4688 hitB	0,23	0,16	0,22
PA0853 -	0,03	ND	ND	PA2864 -	-0,69	ND	ND	PA4689 -	0,00	0,08	0,23
PA0854 fumC2	0,09	-0,12	-0,42	PA2865 -	-0,40	ND	ND	PA4690.3 -	0,00	ND	ND
PA0855 -	-0,05	ND	ND	PA2866 mttC	0,05	-0,11	-0,37	PA4690.4 -	0,05	ND	ND
PA0856 -	0,22	0,33	0,72	PA2867 -	-0,03	0,20	0,59	PA4691 -	-0,08	ND	ND
PA0857 bolA	0,43	0,47	0,90	PA2868 -	0,29	ND	ND	PA4692 -	-0,19	ND	ND
PA0858 -	0,38	0,03	-0,30	PA2869 -	-0,35	ND	ND	PA4693 pssA	0,16	ND	ND
PA0859 -	0,51	ND	ND	PA2870 -	-0,54	ND	ND	PA4694 ilvC	-0,45	-0,22	-0,19
PA0860 -	0,37	0,22	0,26	PA2871 -	-0,47	-0,18	-0,04	PA4695 ilvH	-0,14	-0,09	-0,13
PA0861 -	-0,37	ND	ND	PA2872 -	-0,72	ND	ND	PA4696 ilvI	-0,19	-0,10	-0,11
PA0862 -	-0,46	ND	ND	PA2873 -	**-1,22**	ND	ND	PA4697 -	**-1,21**	ND	ND
PA0863 -	0,29	0,12	0,06	PA2874 -	**-1,13**	ND	ND	PA4698 -	0,20	0,42	**1,00**
PA0865 hpd	**2,46**	**2,53**	**4,73**	PA2875 -	-0,68	-0,58	-0,96	PA4699 -	0,04	0,07	0,14
PA0866 aroP2	**2,75**	ND	ND	PA2876 pyrF	-0,04	-0,09	-0,23	PA4700 mrcB	0,35	0,16	0,10
PA0867 mliC	0,19	0,23	0,48	PA2877 -	-0,39	ND	ND	PA4701 -	-0,58	-0,33	-0,36
PA0868 -	0,59	ND	ND	PA2878 -	0,00	ND	ND	PA4702 -	0,32	ND	ND
PA0869 pbpG	0,68	0,95	**2,03**	PA2879 -	-0,81	ND	ND	PA4704 cbpA	**-1,65**	ND	ND
PA0870 phhC	**2,41**	**2,00**	**3,28**	PA2880 -	-0,58	ND	ND	PA4704.1 prrF1	-0,10	ND	ND
PA0871 phhB	**2,14**	**2,16**	**4,02**	PA2883 -	**-1,01**	ND	ND	PA4704.2 prrF2	0,16	ND	ND
PA0872 phhA	**1,68**	**1,79**	**3,42**	PA2884 -	0,05	ND	ND	PA4705 -	0,00	ND	ND
PA0873 phhR	-0,49	0,35	**1,50**	PA2885 atuR	-0,68	-0,02	0,62	PA4706 -	0,12	ND	ND
PA0874 -	**1,46**	ND	ND	PA2886 atuA	0,00	ND	ND	PA4707 -	0,34	ND	ND
PA0876 -	0,58	0,01	-0,54	PA2887 atuB	0,81	ND	ND	PA4708 phuT	0,22	**1,91**	**5,22**
PA0877 -	0,19	ND	ND	PA2888 atuC	0,87	ND	ND	PA4709 -	0,29	**1,98**	**5,33**
PA0887 acsA	**-3,29**	**-1,50**	-0,97	PA2889 atuD	0,35	ND	ND	PA4710 phuR	-0,46	**2,31**	**7,03**
PA0887.1 -	-0,35	ND	ND	PA2891 atuF	ND	-0,05	ND	PA4711 -	0,25	ND	ND
PA0888 aotJ	-0,08	0,03	0,18	PA2893 atuH	0,00	ND	ND	PA4712 -	0,17	ND	ND
PA0889 aotQ	-0,13	-0,23	-0,51	PA2894 -	-0,27	ND	ND	PA4713 -	-0,24	ND	ND
PA0890 aotM	-0,19	-0,32	-0,73	PA2895 -	0,20	ND	ND	PA4714 -	-0,38	ND	ND
PA0891 -	-0,19	-0,18	-0,33	PA2896 -	-0,26	ND	ND	PA4715 -	0,45	0,02	-0,40
PA0892 aotP	-0,42	-0,14	0,04	PA2897 -	-0,08	0,21	0,69	PA4716 -	-0,56	ND	ND

ID – Gene [a]	TC [b]	PC [c]	PTV [d]
PA0893 argR	-0,18	-0,08	-0,06
PA0894 -	-0,54	ND	ND
PA0895 aruC	0,15	0,33	0,79
PA0896 aruF	0,47	0,08	-0,26
PA0897 aruG	0,20	0,18	0,32
PA0898 aruD	0,29	0,13	0,08
PA0899 aruB	0,42	0,21	0,19
PA0900 -	0,12	ND	ND
PA0901 aruE	0,46	0,34	0,51
PA0902 -	-0,48	-0,10	0,20
PA0903 alaS	0,29	0,22	0,33
PA0904 lysC	0,27	0,12	0,08
PA0905 rsmA	0,18	-0,08	-0,42
PA0905.1 -	**1,77**	ND	ND
PA0905.2 -	**1,76**	ND	ND
PA0905.3 -	**1,69**	ND	ND
PA0906 -	-0,17	ND	ND
PA0907 -	-0,47	ND	ND
PA0908 -	0,24	ND	ND
PA0909 -	-0,06	ND	ND
PA0910 -	0,24	ND	ND
PA0911 -	-0,05	ND	ND
PA0912 -	-0,24	ND	ND
PA0913 mgtE	-0,05	-0,42	**-1,14**
PA0914 -	0,03	ND	ND
PA0915 -	0,45	-0,17	-0,92
PA0916 -	0,25	-0,04	-0,37
PA0917 kup	0,62	0,21	-0,01
PA0918 -	0,17	ND	ND
PA0919 -	0,53	0,32	0,37
PA0920 -	0,62	0,42	0,57
PA0921 -	0,37	ND	ND
PA0922 -	0,03	ND	ND
PA0922.1 -	**1,70**	ND	ND
PA0923 dinB	0,35	ND	ND
PA0924 -	0,54	ND	ND
PA0925 -	0,28	ND	ND
PA0926 -	0,09	0,16	0,35
PA0927 ldhA	0,35	ND	ND
PA0928 gacS	0,21	0,15	0,23
PA0929 -	0,25	ND	ND
PA0930 -	0,27	ND	ND
PA0931 pirA	0,29	0,97	**2,48**
PA0932 cysM	0,55	0,33	0,40
PA0933 ygcA	0,45	0,37	0,59
PA0934 relA	0,39	0,22	0,23
PA0935 -	0,49	0,06	-0,31
PA0936 lpxO2	0,57	0,44	0,67
PA0937 -	0,55	0,18	-0,04
PA0938 -	0,95	0,76	**1,20**
PA0939 -	0,78	ND	ND
PA0940 -	-0,64	ND	ND
PA0941 -	**-1,30**	-0,56	-0,29
PA0942 -	-0,24	-0,12	-0,09
PA0943 -	0,36	0,36	0,65
PA0944 purN	0,52	0,33	0,43
PA0945 purM	0,63	0,37	0,42
PA0946 -	0,01	-0,05	-0,15
PA0947 -	0,83	0,16	-0,36
PA0948 -	0,54	ND	ND
PA0949 wrbA	0,53	0,30	0,33
PA0950 -	0,69	0,47	0,66
PA0951 -	0,30	ND	ND
PA0952 -	-0,38	ND	ND
PA0953 -	0,00	0,09	0,26
PA0954 -	0,21	ND	ND
PA0955 -	0,42	0,40	0,73
PA0956 proS	0,43	0,44	0,81
PA0957 -	-0,03	ND	ND
PA0958 oprD	-0,31	0,14	0,71
PA0959 -	0,72	0,31	0,16
PA0960 -	0,60	ND	ND
PA0961 -	0,61	-0,02	-0,66
PA0962 -	0,17	-0,02	-0,22
PA0963 aspS	0,51	0,48	0,84
PA0964 pmpR	0,21	0,36	0,81

ID – Gene [a]	TC [b]	PC [c]	PTV [d]
PA2899 -	-0,50	ND	ND
PA2900 -	-0,08	-0,25	-0,62
PA2901 -	-0,17	-0,17	-0,30
PA2902 -	0,00	-0,05	-0,15
PA2903 cobJ	0,13	0,08	0,10
PA2904 cobI	-0,13	0,13	0,51
PA2905 cobH	0,19	0,09	0,06
PA2906 -	0,28	0,13	0,09
PA2907 cobL	-0,30	-0,09	0,05
PA2908 cbiD	-0,19	ND	ND
PA2909 -	-0,42	ND	ND
PA2911 -	0,09	-0,28	-0,88
PA2912 -	0,29	ND	ND
PA2915 -	-0,81	ND	ND
PA2917 -	0,58	ND	ND
PA2918 -	-0,78	ND	ND
PA2920 -	-0,70	ND	ND
PA2921 -	-0,52	ND	ND
PA2927 -	0,00	ND	ND
PA2928 -	-0,26	ND	ND
PA2930 -	-0,50	ND	ND
PA2931 cifR	0,33	ND	ND
PA2941 -	0,06	ND	ND
PA2942 -	-0,40	-0,18	-0,11
PA2942.1 -	-0,21	ND	ND
PA2943 -	0,11	ND	ND
PA2944 cobN	-0,28	-0,02	0,23
PA2945 -	-0,29	-0,09	0,05
PA2946 -	-0,64	ND	ND
PA2947 -	-0,20	ND	ND
PA2948 cobM	-0,65	ND	ND
PA2949 -	-0,22	-0,07	0,01
PA2950 -	0,23	-0,20	-0,81
PA2951 etfA	-0,46	-0,19	-0,07
PA2952 etfB	-0,28	-0,21	-0,31
PA2953 -	-0,30	-0,39	-0,80
PA2954 -	-0,19	ND	ND
PA2955 -	0,06	ND	ND
PA2956 -	-0,11	ND	ND
PA2957 -	0,28	-0,22	-0,90
PA2958 -	-0,28	ND	ND
PA2958.1 rgsA	-0,27	ND	ND
PA2959 -	-0,28	-0,22	-0,34
PA2960 pilZ	-0,12	-0,46	**-1,18**
PA2961 holB	-0,36	-0,02	0,30
PA2962 tmk	-0,12	-0,16	-0,34
PA2963 -	0,04	-0,04	-0,15
PA2964 pabC	-0,19	ND	ND
PA2965 fabF1	-0,09	-0,20	-0,47
PA2966 acpP	0,00	0,05	0,15
PA2967 fabG	0,12	-0,25	-0,83
PA2968 fabD	0,47	-0,26	**-1,19**
PA2969 plsX	0,16	ND	ND
PA2970 rpmF	-0,09	-0,03	-0,01
PA2971 -	0,55	0,02	-0,51
PA2972 -	-0,03	-0,14	-0,37
PA2973 -	0,19	-0,02	-0,25
PA2974 -	0,07	-0,04	-0,19
PA2975 rluC	0,17	0,06	-0,01
PA2976 rne	-0,48	-0,18	-0,02
PA2977 murB	0,00	-0,08	-0,23
PA2978 ptpA	0,03	-0,06	-0,19
PA2979 kdsB	0,18	-0,22	-0,82
PA2980 -	-0,02	-0,09	-0,24
PA2981 lpxK	-0,28	ND	ND
PA2982 -	-0,58	-0,30	-0,29
PA2983 -	-0,17	-0,74	**-1,93**
PA2985 -	-0,75	ND	ND
PA2986 -	0,07	0,10	0,20
PA2987 -	0,03	-0,19	-0,58
PA2988 -	0,33	0,06	-0,15
PA2989 -	0,13	ND	ND
PA2990 -	-0,89	-0,33	-0,06
PA2991 sth	-0,83	-0,52	-0,64
PA2992 -	-0,19	ND	ND
PA2993 -	-0,18	0,14	0,59

ID – Gene [a]	TC [b]	PC [c]	PTV [d]
PA4717 -	0,07	-0,10	-0,37
PA4718 -	0,18	0,24	0,51
PA4719 -	0,05	ND	ND
PA4720 trmA	-0,18	0,02	0,23
PA4721 -	0,20	ND	ND
PA4722 -	-0,18	-0,11	-0,13
PA4723 dksA	-0,04	-0,23	-0,61
PA4724 -	-0,19	-0,02	0,13
PA4724.1 -	0,22	ND	ND
PA4725 cbrA	-0,16	ND	ND
PA4726 cbrB	-0,34	0,03	0,41
PA4726.1 -	-0,31	ND	ND
PA4726.11 crcZ	**-8,22**	ND	ND
PA4727 pcnB	0,78	0,47	0,56
PA4728 folK	0,64	0,46	0,68
PA4729 panB	0,17	-0,10	-0,46
PA4730 panC	0,20	-0,13	-0,57
PA4731 panD	0,10	ND	ND
PA4732 pgi	-0,46	-0,08	0,24
PA4733 acsB	-0,05	ND	ND
PA4734 -	-0,43	ND	ND
PA4735 -	-0,02	-0,06	-0,15
PA4736 -	-0,15	ND	ND
PA4737 -	0,38	ND	ND
PA4738 -	-0,76	ND	ND
PA4739 -	-0,71	-0,15	0,28
PA4740 pnp	-0,51	-0,05	0,37
PA4741 rpsO	-0,24	0,14	0,64
PA4742 truB	0,01	-0,04	-0,13
PA4743 rbfA	-0,04	-0,06	-0,12
PA4744 infB	-0,14	-0,10	-0,14
PA4745 nusA	-0,30	-0,13	-0,08
PA4746 -	0,59	0,06	-0,43
PA4746.1 -	**1,13**	ND	ND
PA4746.2 -	0,50	ND	ND
PA4747 secG	0,37	0,22	0,27
PA4748 tpiA	0,60	0,05	-0,45
PA4749 glmM	0,07	0,15	0,36
PA4750 folP	0,37	0,17	0,11
PA4751 ftsH	-0,09	0,08	0,32
PA4752 ftsJ	0,14	-0,26	-0,88
PA4753 -	-0,20	-0,38	-0,88
PA4754 -	-0,26	ND	ND
PA4755 greA	-0,23	-0,12	-0,12
PA4756 carB	-0,28	-0,30	-0,58
PA4757 -	0,05	ND	ND
PA4758 carA	0,00	-0,23	-0,64
PA4758.1 -	-0,09	ND	ND
PA4759 dapB	0,22	-0,13	-0,59
PA4760 dnaJ	0,03	-0,15	-0,47
PA4761 dnaK	-0,10	-0,17	-0,38
PA4762 grpE	0,02	-0,06	-0,21
PA4763 recN	-0,82	-0,12	0,47
PA4764 fur	-0,36	0,20	0,93
PA4765 omlA	0,21	-0,11	-0,52
PA4766 -	0,30	ND	ND
PA4767 -	0,08	-0,02	-0,14
PA4768 smpB	0,17	0,05	-0,02
PA4769 -	-0,51	-0,62	**-1,25**
PA4770 lldP	**-1,76**	ND	ND
PA4771 lldD	**-1,13**	**-1,36**	**-2,74**
PA4772 -	-0,97	**-1,07**	**-2,08**
PA4773 -	-0,12	0,16	0,57
PA4774 -	-0,16	-0,69	**-1,80**
PA4775 -	0,00	ND	ND
PA4776 pmrA	-0,13	-0,06	-0,03
PA4777 pmrB	0,00	ND	ND
PA4778 cueR	-0,08	-0,30	-0,78
PA4779 -	-0,09	ND	ND
PA4780 -	-0,52	ND	ND
PA4781 -	-0,13	ND	ND
PA4782 -	0,33	ND	ND
PA4783 -	0,68	ND	ND
PA4784 -	-0,19	ND	ND
PA4785 -	ND	-0,03	ND
PA4786 -	-0,54	ND	ND

ID – Gene [a]	TC [b]	PC [c]	PTV [d]
PA0965 ruvC	0,51	ND	ND
PA0966 ruvA	0,37	0,13	-0,01
PA0967 ruvB	0,46	0,21	0,15
PA0968 -	0,98	0,23	-0,33
PA0969 tolQ	0,41	**1,03**	**2,52**
PA0970 tolR	0,33	0,62	**1,43**
PA0971 tolA	0,63	0,54	0,90
PA0972 tolB	0,41	0,54	**1,13**
PA0973 oprL	0,28	0,39	0,82
PA0974 -	0,62	0,48	0,76
PA0975 -	**1,07**	0,05	-0,94
PA0976 -	0,21	0,02	-0,16
PA0976.1 -	**1,41**	ND	ND
PA0977 -	**1,21**	ND	ND
PA0978 -	-0,11	ND	ND
PA0979 -	0,22	ND	ND
PA0980 -	-0,24	ND	ND
PA0981 -	-0,47	ND	ND
PA0982 -	-0,83	ND	ND
PA0983 -	0,10	ND	ND
PA0984 -	0,47	ND	ND
PA0985 pyoS5	-0,63	ND	ND
PA0988 -	-0,31	ND	ND
PA0989 -	0,25	ND	ND
PA0990 -	0,00	ND	ND
PA0991 -	0,10	ND	ND
PA0992 cupC1	-0,09	ND	ND
PA0994 cupC3	0,00	ND	ND
PA0995 ogt	**-1,09**	ND	ND
PA0996 pqsA	**-4,29**	ND	ND
PA0997 pqsB	**-4,46**	ND	ND
PA0998 pqsC	**-3,37**	ND	ND
PA0999 pqsD	**-2,06**	ND	ND
PA1000 pqsE	-0,96	ND	ND
PA1001 phnA	**-1,00**	ND	ND
PA1002 phnB	-0,18	ND	ND
PA1003 mvfR	0,03	ND	ND
PA1004 nadA	0,56	0,20	0,01
PA1005 -	-0,05	0,11	0,36
PA1006 -	0,72	ND	ND
PA1007 -	0,91	ND	ND
PA1008 bcp	0,25	0,34	0,73
PA1009 -	0,45	0,26	0,28
PA1010 dapA	0,41	0,09	-0,15
PA1011 -	0,40	0,30	0,45
PA1012 -	0,57	ND	ND
PA1013 purC	0,23	0,25	0,49
PA1013.1 -	0,84	ND	ND
PA1014 -	0,58	-0,12	-0,91
PA1015 -	0,07	-0,15	-0,49
PA1016 -	0,07	ND	ND
PA1020 -	0,12	ND	ND
PA1021 -	0,00	ND	ND
PA1022 -	0,13	ND	ND
PA1023 -	0,00	0,23	0,66
PA1024 -	0,00	ND	ND
PA1026 -	0,48	ND	ND
PA1027 -	-0,42	ND	ND
PA1029 -	-0,37	-0,07	0,17
PA1030 -	0,18	ND	ND
PA1030.1 -	**1,09**	ND	ND
PA1031 -	0,49	0,08	-0,27
PA1032 quiP	0,62	0,32	0,28
PA1033 -	-0,25	-0,05	0,11
PA1034 -	0,53	ND	ND
PA1035 -	-0,39	ND	ND
PA1036 -	-0,88	-0,37	-0,17
PA1037 -	0,45	ND	ND
PA1038 -	0,19	ND	ND
PA1039 -	0,20	ND	ND
PA1040 -	-0,10	0,26	0,84
PA1041 -	-0,32	ND	ND
PA1042 -	0,28	ND	ND
PA1043 -	0,29	0,07	-0,09
PA1044 -	0,16	ND	ND
PA1045 -	0,29	0,17	0,20

ID – Gene [a]	TC [b]	PC [c]	PTV [d]
PA2994 nqrF	-0,07	-0,11	-0,25
PA2995 nqrE	-0,14	ND	ND
PA2996 nqrD	-0,20	ND	ND
PA2997 nqrC	-0,28	-0,05	0,15
PA2998 nqrB	-0,27	ND	ND
PA2999 nqrA	-0,13	-0,13	-0,25
PA3000 aroP1	-0,13	ND	ND
PA3001 -	-0,11	-0,23	-0,54
PA3002 mfd	-0,04	0,05	0,19
PA3003 -	0,08	0,10	0,20
PA3004 -	-0,15	-0,10	-0,14
PA3005 nagZ	-0,29	-0,34	-0,68
PA3006 psrA	-0,54	-0,31	-0,34
PA3007 lexA	**-1,01**	-0,03	0,93
PA3008 -	-0,65	ND	ND
PA3009 -	0,42	ND	ND
PA3010 -	-0,28	-0,12	-0,08
PA3011 topA	-0,30	-0,13	-0,08
PA3012 -	-0,80	0,00	0,80
PA3013 foaB	-0,57	-0,52	-0,90
PA3014 faoA	-0,71	-0,62	**-1,04**
PA3015 -	-0,95	ND	ND
PA3016 -	0,07	ND	ND
PA3017 -	-0,39	ND	ND
PA3018 -	0,17	ND	ND
PA3019 -	0,01	-0,19	-0,55
PA3020 -	-0,16	-0,23	-0,49
PA3021 -	-0,16	-0,25	-0,56
PA3022 -	-0,13	-0,21	-0,46
PA3023 -	-0,53	ND	ND
PA3024 -	-0,58	ND	ND
PA3025 -	-0,46	ND	ND
PA3026 -	-0,13	ND	ND
PA3027 -	0,07	ND	ND
PA3028 moeA2	-0,91	-0,52	-0,56
PA3029 moaB2	-0,91	-0,45	-0,38
PA3030 mobA	-0,78	ND	ND
PA3031 -	-0,65	-0,36	-0,37
PA3033 -	-0,26	ND	ND
PA3034 -	-0,54	ND	ND
PA3035 -	-0,13	0,05	0,26
PA3036 -	0,00	ND	ND
PA3037 -	**-1,14**	ND	ND
PA3038 -	**-1,17**	ND	ND
PA3040 -	-0,74	-0,18	0,23
PA3041 -	-0,23	ND	ND
PA3042 -	-0,49	ND	ND
PA3043 -	**-1,00**	ND	ND
PA3046 -	-0,40	-0,20	-0,16
PA3047 -	-0,41	-0,15	-0,02
PA3048 -	-0,21	-0,12	-0,12
PA3049 rmf	-0,35	ND	ND
PA3050 pyrD	0,03	0,00	-0,02
PA3051 -	-0,37	ND	ND
PA3052 -	-0,73	ND	ND
PA3053 -	**-1,00**	-0,17	0,51
PA3054 -	0,00	ND	ND
PA3055 -	-0,48	ND	ND
PA3056 -	-0,52	-0,19	-0,01
PA3057 -	0,46	ND	ND
PA3066 -	-0,88	ND	ND
PA3067 -	0,25	ND	ND
PA3068 gdhB	0,01	0,17	0,46
PA3069 -	-0,87	ND	ND
PA3070 -	-0,29	-0,25	-0,43
PA3071 -	-0,23	ND	ND
PA3072 -	0,00	ND	ND
PA3073 -	0,00	ND	ND
PA3074 -	0,14	ND	ND
PA3075 -	-0,21	0,05	0,36
PA3076 -	0,20	ND	ND
PA3077 -	-0,06	ND	ND
PA3078 -	-0,06	ND	ND
PA3079 -	0,05	ND	ND
PA3080 -	0,00	ND	ND
PA3081 -	**-1,24**	-0,53	-0,27

ID – Gene [a]	TC [b]	PC [c]	PTV [d]
PA4787 -	0,03	ND	ND
PA4789 -	-0,18	ND	ND
PA4790 -	0,19	ND	ND
PA4791 -	0,00	ND	ND
PA4792 -	0,11	ND	ND
PA4793 -	**-1,29**	ND	ND
PA4794 -	-0,07	ND	ND
PA4795 -	0,69	ND	ND
PA4796 -	0,58	ND	ND
PA4797 -	-0,17	ND	ND
PA4798 -	-0,05	ND	ND
PA4799 -	-0,24	ND	ND
PA4800 -	0,36	ND	ND
PA4801 -	0,15	ND	ND
PA4802 -	-0,13	ND	ND
PA4802.1 -	-0,13	ND	ND
PA4803 -	**-1,00**	ND	ND
PA4804 -	-0,91	ND	ND
PA4806 -	-0,08	ND	ND
PA4807 selB	0,08	ND	ND
PA4808 selA	-0,43	-0,40	-0,72
PA4809 fdhE	0,25	-0,29	**-1,07**
PA4810 fdnI	-0,08	ND	ND
PA4811 fdnH	0,17	ND	ND
PA4812 fdnG	-0,11	ND	ND
PA4815 -	-0,21	ND	ND
PA4816 -	-0,26	ND	ND
PA4821 -	-0,36	ND	ND
PA4826 -	0,11	ND	ND
PA4827 -	-0,58	ND	ND
PA4829 lpd3	-0,53	ND	ND
PA4830 -	0,19	ND	ND
PA4831 -	-0,17	ND	ND
PA4832 -	-0,28	ND	ND
PA4833 -	-0,50	ND	ND
PA4838 -	-0,26	ND	ND
PA4839 speA	0,76	0,32	0,14
PA4840 -	**1,11**	0,11	-0,79
PA4841 -	-0,13	0,13	0,50
PA4842 -	-0,27	-0,27	-0,49
PA4843 -	0,19	0,48	**1,18**
PA4845 dipZ	-0,13	-0,07	-0,07
PA4846 aroQ1	0,67	0,10	-0,39
PA4847 accB	0,12	-0,11	-0,43
PA4848 accC	0,19	-0,09	-0,43
PA4849 -	0,61	ND	ND
PA4850 prmA	0,19	0,02	-0,15
PA4851 -	0,09	-0,08	-0,33
PA4852 -	0,35	ND	ND
PA4853 fis	0,08	-0,09	-0,32
PA4854 purH	0,04	0,31	0,84
PA4855 purD	0,21	0,15	0,20
PA4856 retS	0,19	0,10	0,09
PA4857 -	-0,39	ND	ND
PA4863 -	-0,24	ND	ND
PA4864 ureD	-0,39	ND	ND
PA4865 ureA	-0,78	ND	ND
PA4866 -	-0,25	ND	ND
PA4867 ureB	0,58	ND	ND
PA4868 ureC	-0,06	ND	ND
PA4869 -	-0,33	ND	ND
PA4870 -	-0,47	ND	ND
PA4871 -	-0,47	ND	ND
PA4872 -	-0,68	-0,12	0,33
PA4873 -	-0,39	-0,70	**-1,61**
PA4874 -	-0,60	ND	ND
PA4875 -	-0,09	-0,25	-0,62
PA4876 osmE	-0,49	-0,29	-0,34
PA4878 -	-0,53	0,41	**1,69**
PA4879 -	-0,21	ND	ND
PA4880 -	-0,43	ND	ND
PA4881 -	0,00	ND	ND
PA4885 irlR	-0,84	ND	ND
PA4886 -	0,13	ND	ND
PA4887 -	0,81	0,25	-0,11
PA4888 desB	**-1,47**	ND	ND

ID – Gene [a]	TC [b]	PC [c]	PTV [d]	ID – Gene [a]	TC [b]	PC [c]	PTV [d]	ID – Gene [a]	TC [b]	PC [c]	PTV [d]
PA1046 -	0,37	ND	ND	PA3082 gbt	**-1,42**	**-1,03**	**-1,52**	PA4889 -	**-1,57**	-0,62	-0,18
PA1047 -	0,13	0,07	0,08	PA3083 pepN	-0,31	-0,09	0,04	PA4890 desT	-0,47	-0,18	-0,05
PA1048 -	-0,02	0,18	0,53	PA3084 -	-0,01	-0,20	-0,56	PA4891 ureE	**-1,09**	ND	ND
PA1049 pdxH	0,50	0,06	-0,33	PA3085 -	-0,03	ND	ND	PA4893 ureG	-0,65	ND	ND
PA1050 -	**1,00**	ND	ND	PA3086 -	-0,14	ND	ND	PA4902 -	-0,24	ND	ND
PA1051 -	**-2,46**	0,33	**3,40**	PA3087 -	0,05	ND	ND	PA4906 -	-0,26	ND	ND
PA1052 -	-0,95	0,33	**1,89**	PA3088 -	-0,33	-0,08	0,09	PA4907 -	0,13	-0,09	-0,39
PA1053 -	0,12	0,22	0,51	PA3090 -	-0,71	ND	ND	PA4914 -	**-1,10**	ND	ND
PA1054 -	0,34	ND	ND	PA3091 -	**-1,85**	ND	ND	PA4915 -	-0,55	ND	ND
PA1055 -	0,00	ND	ND	PA3092 fadH1	**-1,00**	-0,33	0,06	PA4916 -	**-1,10**	-0,09	0,83
PA1056 -	0,20	ND	ND	PA3093 -	-0,13	-0,14	-0,26	PA4917 -	**-2,18**	ND	ND
PA1057 -	0,34	ND	ND	PA3094 -	-0,46	ND	ND	PA4918 -	**-3,41**	-0,17	**2,91**
PA1058 -	0,47	ND	ND	PA3094.1 -	**1,50**	ND	ND	PA4919 pncB1	**-1,57**	-0,01	**1,55**
PA1059 -	0,10	ND	ND	PA3094.2 -	**1,12**	ND	ND	PA4920 nadE	-0,79	0,01	0,81
PA1060 -	0,57	ND	ND	PA3094.3 -	**1,40**	ND	ND	PA4921 -	-0,47	ND	ND
PA1061 -	0,97	0,40	0,15	PA3095 xcpZ	-0,34	ND	ND	PA4922 azu	0,23	0,11	0,07
PA1062 -	0,91	ND	ND	PA3096 xcpY	-0,64	ND	ND	PA4923 -	-0,14	-0,15	-0,30
PA1063 -	0,48	ND	ND	PA3097 xcpX	-0,43	ND	ND	PA4924 -	-0,33	ND	ND
PA1064 -	-0,08	-0,03	-0,01	PA3098 xcpW	-0,67	ND	ND	PA4926 -	-0,35	ND	ND
PA1065 -	0,45	ND	ND	PA3099 xcpV	-0,68	ND	ND	PA4928 -	-0,04	0,11	0,36
PA1066 -	0,45	ND	ND	PA3100 xcpU	-0,70	ND	ND	PA4929 -	-0,09	ND	ND
PA1067 -	-0,81	ND	ND	PA3101 xcpT	-0,62	-0,68	**-1,30**	PA4930 alr	-0,03	-0,15	-0,40
PA1068 -	-0,05	-0,01	0,01	PA3102 xcpS	-0,67	ND	ND	PA4931 dnaB	-0,02	-0,05	-0,13
PA1069 -	0,08	-0,07	-0,28	PA3103 xcpR	-0,68	ND	ND	PA4932 rplI	-0,27	-0,08	0,04
PA1070 braG	0,94	0,79	**1,32**	PA3104 xcpP	-0,37	ND	ND	PA4933 -	-0,29	ND	ND
PA1071 braF	**1,06**	**1,07**	**2,00**	PA3105 xcpQ	0,04	-0,14	-0,42	PA4934 rpsR	-0,37	-0,15	-0,05
PA1072 braE	**1,03**	ND	ND	PA3106 -	0,21	0,06	-0,04	PA4935 rpsF	-0,31	-0,07	0,12
PA1073 braD	0,93	ND	ND	PA3107 metZ	0,32	0,05	-0,18	PA4936 -	-0,14	0,10	0,42
PA1074 braC	0,96	**1,47**	**3,21**	PA3108 purF	0,15	0,01	-0,12	PA4937 rnr	-0,46	0,06	0,64
PA1075 -	0,23	0,53	**1,27**	PA3109 -	0,17	ND	ND	PA4937.1 -	**1,50**	ND	ND
PA1076 -	0,13	0,14	0,27	PA3110 -	-0,01	-0,03	-0,06	PA4937.2 -	**1,37**	ND	ND
PA1077 flgB	0,38	ND	ND	PA3111 folC	0,22	-0,01	-0,25	PA4938 purA	-0,04	0,01	0,07
PA1078 flgC	0,40	ND	ND	PA3112 accD	0,46	-0,26	**-1,20**	PA4939 -	0,18	0,18	0,32
PA1079 flgD	0,01	ND	ND	PA3113 trpF	-0,50	ND	ND	PA4940 -	0,42	ND	ND
PA1080 flgE	-0,09	0,06	0,26	PA3114 truA	-0,44	-0,35	-0,55	PA4941 hflC	-0,01	0,09	0,28
PA1081 flgF	0,51	ND	ND	PA3115 fimV	-0,78	-0,76	**-1,38**	PA4942 hflK	0,05	0,14	0,35
PA1082 flgG	-0,05	ND	ND	PA3116 -	0,03	-0,30	-0,88	PA4943 -	-0,01	-0,04	-0,10
PA1083 flgH	0,04	0,18	0,49	PA3117 asd	-0,44	-0,22	-0,20	PA4944 hfq	-0,23	ND	ND
PA1084 flgI	0,24	0,11	0,08	PA3118 leuB	-0,56	-0,28	-0,24	PA4945 miaA	-0,10	0,19	0,65
PA1085 flgJ	0,03	ND	ND	PA3119 -	0,42	ND	ND	PA4946 mutL	0,22	0,12	0,14
PA1086 flgK	-0,10	0,09	0,37	PA3120 leuD	-0,44	-0,19	-0,11	PA4947 amiB	0,27	0,19	0,27
PA1087 flgL	0,12	0,15	0,30	PA3121 leuC	-0,60	-0,47	-0,72	PA4948 -	0,10	0,07	0,11
PA1088 -	0,16	ND	ND	PA3122 -	-0,22	ND	ND	PA4949 -	0,11	0,02	-0,04
PA1089 -	0,33	0,10	-0,06	PA3123 -	-0,41	ND	ND	PA4950 -	-0,17	ND	ND
PA1090 -	0,48	ND	ND	PA3124 -	-0,28	ND	ND	PA4951 orn	-0,30	-0,07	0,11
PA1091 fgtA	0,48	0,20	0,09	PA3125 -	-0,42	ND	ND	PA4952 -	0,01	-0,03	-0,10
PA1092 fliC	0,30	0,14	0,11	PA3126 ibpA	0,46	-0,30	**-1,31**	PA4953 motB	-0,18	0,02	0,24
PA1093 -	0,41	0,42	0,78	PA3127 -	-0,75	ND	ND	PA4954 motA	-0,25	ND	ND
PA1094 fliD	0,41	0,29	0,42	PA3128 -	-0,37	ND	ND	PA4955 -	-0,44	0,34	**1,40**
PA1095 -	0,26	0,27	0,51	PA3129 -	0,04	ND	ND	PA4956 rhdA	0,26	-0,16	-0,71
PA1096 -	0,26	-0,07	-0,46	PA3130 -	-0,86	ND	ND	PA4957 psd	-0,01	0,18	0,52
PA1097 fleQ	-0,13	0,01	0,17	PA3131 -	-0,43	-0,44	-0,81	PA4958 -	-0,89	-0,19	0,35
PA1098 fleS	0,12	ND	ND	PA3132 -	0,46	ND	ND	PA4959 fimX	-0,16	-0,12	-0,17
PA1099 fleR	-0,36	-0,04	0,26	PA3133 -	-0,24	ND	ND	PA4960 -	0,00	-0,01	-0,03
PA1100 fliE	0,14	ND	ND	PA3133.1 -	**1,00**	ND	ND	PA4961 -	-0,23	-0,17	-0,25
PA1101 fliF	-0,05	-0,14	-0,34	PA3133.2 -	**1,06**	ND	ND	PA4962 -	-0,09	ND	ND
PA1102 fliG	0,03	-0,06	-0,21	PA3133.3 -	0,94	ND	ND	PA4963 -	0,32	0,08	-0,09
PA1103 -	0,24	0,33	0,70	PA3133.4 -	**1,01**	ND	ND	PA4964 parC	-0,44	-0,22	-0,20
PA1104 fliI	0,36	-0,02	-0,41	PA3134 gltX	-0,26	-0,28	-0,53	PA4965 -	-0,42	ND	ND
PA1105 fliJ	-0,34	ND	ND	PA3135 -	-0,49	ND	ND	PA4966 -	-0,49	-0,16	0,05
PA1106 -	-0,03	ND	ND	PA3137 -	-0,22	ND	ND	PA4967 parE	-0,32	-0,22	-0,30
PA1109 -	0,00	ND	ND	PA3138 uvrB	-0,50	-0,19	-0,06	PA4968 -	-0,53	-0,27	-0,25
PA1110 -	0,11	ND	ND	PA3139 -	0,79	0,60	0,92	PA4969 cpdA	-0,98	-0,59	-0,71
PA1112 -	-0,13	ND	ND	PA3139.1 -	0,95	ND	ND	PA4970 -	-0,09	ND	ND
PA1112.1 -	-0,49	ND	ND	PA3140 -	0,09	ND	ND	PA4971 aspP	-0,17	-0,19	-0,38
PA1113 -	0,13	ND	ND	PA3141 wbpM	-0,18	-0,22	-0,44	PA4972 -	0,29	0,04	-0,19
PA1114 -	0,14	ND	ND	PA3142 -	-0,49	ND	ND	PA4973 thiC	-0,12	0,17	0,60
PA1115 -	-0,34	ND	ND	PA3143 -	0,18	ND	ND	PA4974 -	-0,24	-0,42	-0,95
PA1116 -	0,74	0,23	-0,10	PA3144 -	0,29	ND	ND	PA4975 -	0,84	**1,15**	**2,42**
PA1117 -	0,66	0,46	0,66	PA3145 wbpL	0,41	ND	ND	PA4976 aruH	**1,09**	0,63	0,71
PA1118 -	-0,25	ND	ND	PA3146 wbpK	0,29	0,23	0,36	PA4977 aruI	**1,81**	ND	ND
PA1119 yfiB	0,74	0,15	-0,32	PA3147 wbpJ	0,23	0,30	0,64	PA4978 -	**2,17**	ND	ND
PA1120 tpbB	0,42	ND	ND	PA3148 wbpI	0,07	0,06	0,11	PA4979 -	**2,36**	ND	ND
PA1121 yfiR	0,40	ND	ND	PA3149 wbpH	0,28	0,06	-0,11	PA4980 -	**1,06**	ND	ND
PA1122 -	0,16	0,08	0,07	PA3150 wbpG	0,20	-0,15	-0,61	PA4982 -	**1,14**	ND	ND
PA1123 -	0,98	ND	ND	PA3151 hisF2	0,12	0,01	-0,09	PA4983 -	0,21	ND	ND

ID – Gene [a]	TC [b]	PC [c]	PTV [d]	ID – Gene [a]	TC [b]	PC [c]	PTV [d]	ID – Gene [a]	TC [b]	PC [c]	PTV [d]
PA1124 dgt	-0,05	ND	ND	PA3152 hisH2	0,32	0,12	0,02	PA4984 -	-0,78	ND	ND
PA1125 -	0,37	ND	ND	PA3153 wzx	0,20	ND	ND	PA4985 -	-0,83	ND	ND
PA1126 -	0,40	ND	ND	PA3154 wzy	0,38	ND	ND	PA4986 -	-0,64	ND	ND
PA1127 -	-0,45	0,27	**1,22**	PA3155 wbpE	-0,04	0,01	0,05	PA4987 -	0,00	ND	ND
PA1128 -	-0,22	ND	ND	PA3156 wbpD	0,05	0,00	-0,06	PA4988 waaA	-0,08	0,01	0,10
PA1129 -	0,39	ND	ND	PA3157 -	0,07	ND	ND	PA4989 -	-0,64	ND	ND
PA1130 rhlC	0,00	ND	ND	PA3158 wbpB	-0,19	-0,26	-0,55	PA4990 -	**-1,25**	ND	ND
PA1132 -	0,79	ND	ND	PA3159 wbpA	-0,26	-0,20	-0,30	PA4991 -	-0,45	0,01	0,47
PA1133 -	0,22	ND	ND	PA3160 wzz	0,05	-0,19	-0,58	PA4992 -	-0,19	0,04	0,31
PA1135 -	-0,10	0,02	0,16	PA3161 himD	-0,71	ND	ND	PA4993 -	-0,31	ND	ND
PA1136 -	-0,78	ND	ND	PA3162 rpsA	-0,35	-0,08	0,11	PA4995 -	-0,50	ND	ND
PA1137 -	ND	0,38	ND	PA3163 cmk	-0,05	0,01	0,07	PA4996 rfaE	-0,36	-0,20	-0,21
PA1138 -	-0,74	ND	ND	PA3165 hisC2	-0,13	-0,04	0,01	PA4997 msbA	-0,19	-0,08	-0,03
PA1139 -	-0,11	ND	ND	PA3166 pheA	-0,22	-0,08	-0,02	PA4998 -	-0,31	-0,51	**-1,15**
PA1140 -	0,93	0,51	0,53	PA3167 serC	-0,12	-0,14	-0,28	PA4999 waaL	-0,01	ND	ND
PA1141 -	-0,14	ND	ND	PA3168 gyrA	-0,33	-0,29	-0,49	PA5000 wapR	0,04	0,09	0,21
PA1142 -	-0,04	ND	ND	PA3169 -	-0,14	-0,17	-0,36	PA5001 -	0,14	0,02	-0,08
PA1145 -	0,42	ND	ND	PA3170 -	-0,32	-0,10	0,05	PA5002 -	0,18	-0,01	-0,21
PA1147 -	0,54	ND	ND	PA3171 ubiG	-0,36	-0,08	0,13	PA5003 -	0,00	-0,08	-0,23
PA1150 pys2	-0,35	ND	ND	PA3172 -	-0,22	-0,14	-0,18	PA5004 -	-0,06	-0,11	-0,26
PA1151 imm2	-0,16	0,15	0,59	PA3173 -	-0,32	-0,08	0,08	PA5005 -	-0,15	-0,17	-0,32
PA1152 -	0,54	ND	ND	PA3177 -	-0,66	ND	ND	PA5006 -	0,20	0,28	0,60
PA1153 -	0,12	ND	ND	PA3178 -	-0,25	ND	ND	PA5007 -	0,07	0,10	0,20
PA1154 -	0,24	ND	ND	PA3179 -	0,03	-0,23	-0,68	PA5008 -	0,06	0,11	0,25
PA1155 nrdB	0,05	0,07	0,15	PA3180 -	0,26	ND	ND	PA5009 waaP	0,15	0,01	-0,12
PA1156 nrdA	-0,28	0,04	0,38	PA3181 -	-0,29	**1,60**	**4,84**	PA5010 waaG	0,10	0,06	0,06
PA1157 -	0,78	0,50	0,63	PA3182 pgl	-0,64	**1,71**	**5,53**	PA5011 waaC	0,11	0,14	0,29
PA1158 -	0,70	ND	ND	PA3183 zwf	**-1,18**	**1,63**	**5,83**	PA5012 waaF	-0,11	-0,04	0,00
PA1159 -	0,49	0,75	**1,63**	PA3184 -	-0,22	-0,12	-0,12	PA5013 ilvE	-0,12	-0,05	-0,03
PA1160 -	0,44	0,28	0,35	PA3185 -	-0,04	-0,20	-0,53	PA5014 glnE	0,22	0,00	-0,22
PA1161 rrmA	0,35	0,34	0,62	PA3186 oprB	**2,35**	**2,76**	**5,50**	PA5015 aceE	0,50	0,56	**1,09**
PA1162 dapE	0,30	0,33	0,62	PA3187 -	-0,22	**2,29**	**6,75**	PA5016 aceF	0,64	0,49	0,75
PA1163 ndvB	0,82	ND	ND	PA3188 -	-0,46	**3,56**	**10,60**	PA5017 -	-0,28	ND	ND
PA1164 -	0,54	ND	ND	PA3189 -	ND	**2,78**	ND	PA5018 msrA	-0,67	-0,43	-0,56
PA1165 pcpS	0,41	0,17	0,09	PA3190 -	0,20	**2,92**	**8,10**	PA5019 -	-0,05	-0,29	-0,77
PA1167 -	-0,22	ND	ND	PA3191 -	0,56	ND	ND	PA5020 -	**-1,85**	ND	ND
PA1170 -	**1,18**	ND	ND	PA3192 gltR	0,06	ND	ND	PA5021 -	-0,06	ND	ND
PA1171 -	0,43	0,18	0,08	PA3193 glk	0,02	**1,11**	**3,15**	PA5022 -	0,05	0,26	0,69
PA1172 napC	0,31	ND	ND	PA3194 edd	**-1,07**	**1,11**	**4,22**	PA5023 -	-0,79	0,24	**1,46**
PA1173 napB	0,46	ND	ND	PA3195 gapA	-0,10	**2,08**	**6,02**	PA5024 -	**1,64**	ND	ND
PA1177 napE	-0,22	ND	ND	PA3196 -	0,20	ND	ND	PA5025 metY	-0,31	-0,23	-0,34
PA1178 oprH	0,10	0,13	0,28	PA3197 -	-0,13	ND	ND	PA5026 -	0,00	ND	ND
PA1179 phoP	0,07	0,14	0,32	PA3198 -	0,00	ND	ND	PA5027 -	**-1,28**	-0,20	0,71
PA1180 phoQ	0,29	0,37	0,76	PA3199 -	-0,43	-0,30	-0,43	PA5028 -	-0,55	ND	ND
PA1181 -	**1,19**	0,30	-0,34	PA3200 -	-0,27	ND	ND	PA5029 -	-0,49	ND	ND
PA1182 -	0,81	ND	ND	PA3201 -	-0,31	ND	ND	PA5030 -	**2,86**	ND	ND
PA1183 dctA	ND	-0,58	ND	PA3202 -	-0,09	ND	ND	PA5031 -	**1,14**	ND	ND
PA1184 -	-0,26	ND	ND	PA3203 -	-0,14	ND	ND	PA5033 -	**-1,29**	ND	ND
PA1188 -	0,29	ND	ND	PA3204 -	-0,14	ND	ND	PA5034 hemE	0,24	0,07	-0,04
PA1189 -	0,58	ND	ND	PA3205 -	**1,22**	ND	ND	PA5035 gltD	-0,25	-0,75	**-1,88**
PA1190 -	0,00	ND	ND	PA3206 -	-0,25	ND	ND	PA5036 gltB	-0,47	-0,54	**-1,07**
PA1191 -	-0,89	ND	ND	PA3207 -	-0,34	ND	ND	PA5037 -	0,01	-0,06	-0,18
PA1192 -	0,60	0,39	0,52	PA3208 -	-0,26	-0,28	-0,53	PA5038 aroB	-0,04	-0,03	-0,05
PA1193 -	0,36	0,30	0,49	PA3209 -	0,67	ND	ND	PA5039 aroK	-0,22	ND	ND
PA1194 -	0,50	ND	ND	PA3210 trkH	-0,21	ND	ND	PA5040 pilQ	-0,77	-0,46	-0,54
PA1196 -	-0,38	0,32	**1,30**	PA3211 -	-0,42	-0,24	-0,27	PA5041 pilP	**-1,07**	-0,04	0,97
PA1197 -	0,54	ND	ND	PA3212 -	0,06	ND	ND	PA5042 pilO	**-1,11**	-0,97	**-1,64**
PA1198 -	0,45	ND	ND	PA3213 -	-0,18	-0,13	-0,19	PA5043 pilN	**-1,28**	**-1,03**	**-1,65**
PA1199 -	-0,73	ND	ND	PA3214 -	-0,17	-0,13	-0,19	PA5044 pilM	**-1,48**	-0,88	**-1,02**
PA1200 -	-0,63	0,08	0,86	PA3215 -	-0,09	ND	ND	PA5045 ponA	-0,16	-0,01	0,14
PA1201 -	-0,10	ND	ND	PA3216 -	-0,58	ND	ND	PA5046 -	0,44	0,42	0,75
PA1202 -	-0,55	0,25	**1,27**	PA3217 cyaB	0,13	ND	ND	PA5047 -	0,45	0,08	-0,22
PA1203 -	-0,69	-0,52	-0,79	PA3220 -	-0,53	ND	ND	PA5048 -	0,18	ND	ND
PA1204 -	-0,57	ND	ND	PA3221 csaA	**2,93**	0,77	-0,73	PA5049 rpmE	0,13	0,05	0,01
PA1205 -	-0,40	ND	ND	PA3222 -	**3,50**	ND	ND	PA5050 priA	-0,09	0,44	**1,34**
PA1206 -	0,28	-0,39	**-1,39**	PA3223 azoR3	**2,21**	ND	ND	PA5051 argS	-0,13	-0,08	-0,09
PA1207 kefB	0,19	-0,10	-0,47	PA3224 -	-0,14	ND	ND	PA5052 -	0,01	0,20	0,57
PA1208 -	0,10	ND	ND	PA3225 -	0,00	-0,19	-0,55	PA5053 hslV	0,15	-0,36	**-1,18**
PA1209 -	-0,35	ND	ND	PA3226 -	0,09	-0,16	-0,55	PA5054 hslU	0,00	-0,17	-0,48
PA1222 -	0,50	0,34	0,47	PA3227 ppiA	-0,10	0,19	0,64	PA5055 -	-0,11	0,16	0,56
PA1223 -	-0,34	ND	ND	PA3228 -	0,41	0,56	**1,17**	PA5056 phaC1	-0,21	ND	ND
PA1224 -	-0,25	ND	ND	PA3229 -	-0,29	ND	ND	PA5057 phaD	-0,35	ND	ND
PA1225 -	-0,33	-0,07	0,15	PA3230 -	-0,21	ND	ND	PA5058 phaC2	-0,58	ND	ND
PA1226 -	-0,05	0,02	0,10	PA3232 -	-0,87	ND	ND	PA5059 -	-0,18	ND	ND
PA1227 -	-0,42	ND	ND	PA3233 -	**-1,26**	ND	ND	PA5060 phaF	-0,62	-0,45	-0,68
PA1228 -	-0,28	-0,38	-0,81	PA3234 -	**-2,04**	ND	ND	PA5061 -	-0,39	ND	ND

ID – Gene [a]	TC [b]	PC [c]	PTV[d]
PA1229 -	-0,42	ND	ND
PA1233 -	0,22	ND	ND
PA1234 -	0,10	ND	ND
PA1235 -	-0,49	ND	ND
PA1241 -	0,00	ND	ND
PA1243 -	0,54	0,12	-0,21
PA1244 -	0,55	0,15	-0,12
PA1245 -	**-1,27**	ND	ND
PA1247 aprE	**-1,46**	ND	ND
PA1249 aprA	-0,45	ND	ND
PA1250 aprI	0,36	0,24	0,32
PA1251 -	0,19	ND	ND
PA1263 -	0,21	ND	ND
PA1264 -	0,65	ND	ND
PA1269 -	0,03	ND	ND
PA1271 -	0,74	0,14	-0,36
PA1272 cobO	0,60	0,24	0,08
PA1273 cobB	0,43	0,27	0,35
PA1274 -	0,70	ND	ND
PA1275 cobD	0,57	0,20	-0,01
PA1276 cobC	0,66	0,42	0,55
PA1277 cobQ	0,60	0,16	-0,13
PA1278 cobP	0,70	0,29	0,13
PA1279 cobU	0,51	0,22	0,12
PA1280 -	0,65	ND	ND
PA1281 cobV	0,51	ND	ND
PA1282 -	0,00	ND	ND
PA1283 -	-0,21	0,08	0,44
PA1285 -	0,27	-0,14	-0,68
PA1286 -	0,29	ND	ND
PA1287 -	-0,26	0,10	0,54
PA1288 -	-0,13	0,57	**1,74**
PA1289 -	-0,83	ND	ND
PA1290 -	0,26	ND	ND
PA1291 -	-0,11	ND	ND
PA1292 -	0,00	0,07	0,21
PA1293 -	-0,19	0,04	0,31
PA1294 rnd	0,40	0,19	0,14
PA1295 -	0,43	0,39	0,67
PA1296 -	0,44	ND	ND
PA1297 -	0,36	ND	ND
PA1298 -	-0,17	ND	ND
PA1299 -	0,84	ND	ND
PA1300 -	**1,17**	ND	ND
PA1301 -	0,28	ND	ND
PA1303 -	0,39	ND	ND
PA1304 -	0,44	0,33	0,51
PA1305 -	0,50	ND	ND
PA1306 -	0,34	ND	ND
PA1307 -	0,14	0,24	0,56
PA1308 -	0,39	ND	ND
PA1309 -	-0,37	ND	ND
PA1312 -	-0,24	ND	ND
PA1314 -	0,09	ND	ND
PA1315 -	-0,47	0,14	0,86
PA1316 -	0,00	ND	ND
PA1317 cyoA	**-2,90**	ND	ND
PA1318 cyoB	**-3,15**	ND	ND
PA1319 cyoC	**-3,54**	ND	ND
PA1320 cyoD	**-2,56**	ND	ND
PA1321 cyoE	**-2,63**	ND	ND
PA1323 -	0,00	ND	ND
PA1324 -	-0,13	-0,11	-0,18
PA1324.1 -	-0,37	ND	ND
PA1325 -	-0,78	ND	ND
PA1326 ilvA2	**-1,00**	ND	ND
PA1328 -	-0,93	ND	ND
PA1330 -	-0,21	-0,10	-0,07
PA1331 -	0,48	ND	ND
PA1332 -	-0,76	ND	ND
PA1333 -	**-2,00**	ND	ND
PA1335 -	0,51	0,01	-0,48
PA1336 -	0,27	ND	ND
PA1337 ansB	**1,36**	0,37	-0,31
PA1338 ggt	0,46	0,86	**2,00**
PA1339 -	0,04	0,04	0,07

ID – Gene [a]	TC [b]	PC [c]	PTV[d]
PA3235 -	**-2,78**	ND	ND
PA3238 -	-0,87	-0,20	0,28
PA3239 -	-0,62	ND	ND
PA3240 -	-0,95	ND	ND
PA3241 -	-0,22	ND	ND
PA3242 -	0,18	-0,06	-0,35
PA3243 minC	-0,75	-0,29	-0,09
PA3244 minD	-0,20	-0,34	-0,77
PA3245 minE	-0,32	-0,35	-0,69
PA3246 rluA	0,09	0,06	0,08
PA3247 -	-0,39	0,08	0,62
PA3248 -	-0,72	ND	ND
PA3254 -	-0,28	ND	ND
PA3255 -	-0,88	-0,16	0,42
PA3256 -	-0,76	-0,27	-0,01
PA3257 prc	-0,71	-0,24	0,02
PA3258 -	-0,24	ND	ND
PA3259 -	-0,58	ND	ND
PA3260 -	-0,55	-0,15	0,13
PA3261 -	-0,31	ND	ND
PA3262 -	-0,31	-0,29	-0,50
PA3262.1 -	**1,28**	ND	ND
PA3262.2 -	**1,49**	ND	ND
PA3263 -	-0,64	-0,54	-0,89
PA3264 -	-0,40	ND	ND
PA3265 -	0,23	ND	ND
PA3266 capB	-0,73	-0,35	-0,26
PA3267 -	-0,09	-0,08	-0,14
PA3268 -	-0,06	0,46	**1,36**
PA3269 -	-0,78	ND	ND
PA3270 -	-0,78	-0,31	-0,10
PA3271 -	**-2,75**	-0,32	**1,85**
PA3272 -	-0,42	ND	ND
PA3274 -	**-1,28**	ND	ND
PA3276 -	-0,21	ND	ND
PA3277 -	-0,87	ND	ND
PA3278 -	**-1,19**	ND	ND
PA3281 -	-0,46	ND	ND
PA3283 -	-0,58	ND	ND
PA3284 -	**-1,03**	ND	ND
PA3285 -	-0,22	ND	ND
PA3286 -	-0,08	-0,48	**-1,28**
PA3287 -	-0,34	ND	ND
PA3288 -	0,07	ND	ND
PA3289 -	-0,22	ND	ND
PA3290 -	-0,13	ND	ND
PA3291 -	-0,42	ND	ND
PA3293 -	0,00	ND	ND
PA3294 -	-0,08	ND	ND
PA3295 -	-0,94	-0,47	-0,39
PA3297 -	-0,17	-0,16	-0,30
PA3299 fadD1	-0,56	-0,28	-0,23
PA3300 fadD2	0,20	ND	ND
PA3301 -	-0,39	ND	ND
PA3302 -	-0,59	-0,36	-0,44
PA3304 -	0,45	ND	ND
PA3304.1 -	-0,49	ND	ND
PA3305 -	-0,49	ND	ND
PA3305.1 phrS	-0,56	ND	ND
PA3306 -	-0,40	ND	ND
PA3307 -	**-1,28**	ND	ND
PA3308 hepA	-0,20	-0,13	-0,17
PA3309 -	0,20	-0,35	**-1,20**
PA3310 -	0,24	0,06	-0,06
PA3312 -	-0,29	-0,12	-0,04
PA3313 -	-0,17	-0,28	-0,63
PA3314 -	-0,33	0,06	0,50
PA3315 -	0,19	ND	ND
PA3316 -	0,00	ND	ND
PA3317 -	-0,47	ND	ND
PA3321 -	0,00	ND	ND
PA3322 -	-0,30	0,01	0,32
PA3325 -	-0,91	ND	ND
PA3326 -	**-1,90**	-0,39	0,78
PA3331 -	**-2,58**	ND	ND
PA3332 -	**-2,81**	ND	ND

ID – Gene [a]	TC [b]	PC [c]	PTV[d]
PA5062 -	-0,84	ND	ND
PA5063 ubiE	-0,28	-0,21	-0,32
PA5064 -	-0,35	-0,06	0,17
PA5065 ubiB	0,00	0,00	0,00
PA5066 hisI	-0,42	ND	ND
PA5067 hisE	-0,01	0,05	0,16
PA5068 tatA	-0,12	ND	ND
PA5069 tatB	0,07	ND	ND
PA5070 tatC	0,39	ND	ND
PA5071 -	0,23	0,07	-0,02
PA5072 -	-0,06	-0,08	-0,15
PA5073 -	-0,13	ND	ND
PA5074 -	-0,15	0,08	0,37
PA5075 -	0,01	-0,05	-0,16
PA5076 -	0,14	-0,05	-0,27
PA5077 mdoH	-0,12	-0,04	-0,01
PA5078 -	-0,20	-0,18	-0,31
PA5079 -	-0,03	-0,04	-0,08
PA5080 -	0,09	0,01	-0,07
PA5081 -	-0,18	ND	ND
PA5082 -	0,07	ND	ND
PA5083 -	0,00	ND	ND
PA5085 -	-0,70	ND	ND
PA5086 -	-0,19	ND	ND
PA5087 -	-0,16	ND	ND
PA5088 -	-0,28	ND	ND
PA5091 hutG	-0,42	ND	ND
PA5092 hutI	-0,35	ND	ND
PA5094 -	-0,65	ND	ND
PA5095 -	-0,65	ND	ND
PA5103 -	-0,46	ND	ND
PA5104 -	0,06	ND	ND
PA5105 hutC	-0,36	-0,03	0,29
PA5106 -	**-1,14**	ND	ND
PA5107 blc	0,03	ND	ND
PA5108 -	-0,02	-0,12	-0,33
PA5109 -	-0,31	ND	ND
PA5110 fbp	0,00	-0,01	-0,04
PA5111 gloA3	0,08	-0,02	-0,14
PA5112 estA	0,37	0,78	**1,86**
PA5113 -	0,00	-0,13	-0,37
PA5114 -	0,02	ND	ND
PA5115 -	**-1,14**	ND	ND
PA5116 -	**-1,58**	ND	ND
PA5117 typA	-0,44	0,01	0,46
PA5118 thiI	0,20	0,03	-0,12
PA5119 glnA	0,24	0,14	0,16
PA5120 -	0,28	ND	ND
PA5121 -	0,38	-0,16	-0,85
PA5122 -	**-1,35**	ND	ND
PA5123 -	**-1,03**	-0,32	0,13
PA5124 ntrB	0,33	ND	ND
PA5125 ntrC	-0,05	-0,16	-0,40
PA5126 -	-0,29	ND	ND
PA5127 -	0,42	-0,03	-0,50
PA5128 secB	-0,35	-0,08	0,12
PA5129 grx	-0,30	0,18	0,81
PA5130 -	0,22	-0,02	-0,29
PA5131 pgm	-0,09	-0,19	-0,45
PA5132 -	0,55	ND	ND
PA5133 -	0,01	0,14	0,37
PA5134 -	-0,21	-0,09	-0,04
PA5135 -	0,02	0,21	0,59
PA5136 -	0,02	0,08	0,21
PA5137 -	-0,29	ND	ND
PA5138 -	-0,67	0,03	0,75
PA5139 -	**-1,39**	-0,99	**-1,42**
PA5140 hisF1	-0,14	0,01	0,16
PA5141 hisA	-0,01	-0,03	-0,07
PA5142 hisH1	-0,12	0,11	0,43
PA5143 hisB	-0,07	0,01	0,11
PA5144 -	0,58	ND	ND
PA5145 -	-0,37	ND	ND
PA5146 -	0,00	-0,06	-0,16
PA5147 mutY	0,03	0,00	-0,02
PA5148 -	-0,17	-0,20	-0,40

ID – Gene [a]	TC [b]	PC [c]	PTV[d]
PA1340 -	0,03	ND	ND
PA1341 -	0,11	-0,06	-0,28
PA1342 -	0,08	0,08	0,15
PA1343 -	0,29	ND	ND
PA1344 -	0,11	-0,01	-0,12
PA1357 -	-0,24	0,07	0,44
PA1358 -	0,04	ND	ND
PA1359 -	0,53	ND	ND
PA1360 -	0,17	ND	ND
PA1361 -	0,30	ND	ND
PA1362 -	-0,29	ND	ND
PA1363 -	0,95	ND	ND
PA1364 -	0,39	ND	ND
PA1365 -	0,39	0,38	0,69
PA1366 -	-0,27	ND	ND
PA1367 -	0,22	0,18	0,30
PA1368 -	-0,26	ND	ND
PA1369 -	-0,25	ND	ND
PA1370 -	0,10	ND	ND
PA1371 -	0,17	ND	ND
PA1372 -	0,08	0,03	0,01
PA1373 fabF2	0,21	-0,06	-0,39
PA1374 -	-0,39	ND	ND
PA1375 pdxB	0,19	0,11	0,13
PA1376 aceK	-0,22	ND	ND
PA1377 -	-0,03	ND	ND
PA1378 -	0,25	ND	ND
PA1380 -	0,11	ND	ND
PA1381 -	0,00	ND	ND
PA1382 -	0,29	ND	ND
PA1383 -	0,00	0,18	0,52
PA1384 galE	0,29	ND	ND
PA1385 -	0,87	ND	ND
PA1386 -	0,24	ND	ND
PA1387 -	-0,08	ND	ND
PA1388 -	0,53	ND	ND
PA1389 -	0,24	ND	ND
PA1390 -	0,65	ND	ND
PA1392 -	**1,00**	ND	ND
PA1393 cysC	0,36	ND	ND
PA1394 -	0,90	ND	ND
PA1395 -	0,22	ND	ND
PA1396 -	0,06	ND	ND
PA1397 -	0,43	ND	ND
PA1398 -	0,06	ND	ND
PA1399 -	-0,30	ND	ND
PA1401 -	-0,32	ND	ND
PA1403 -	0,16	ND	ND
PA1404 -	0,24	ND	ND
PA1405 -	0,00	ND	ND
PA1406 -	0,00	ND	ND
PA1407 -	0,18	ND	ND
PA1411 -	0,13	ND	ND
PA1413 -	-0,92	ND	ND
PA1414 -	0,54	ND	ND
PA1415 -	0,35	ND	ND
PA1416 -	-0,82	ND	ND
PA1417 -	**-2,07**	0,20	**2,64**
PA1418 -	**-2,17**	ND	ND
PA1419 -	**-1,93**	ND	ND
PA1420 -	**-3,81**	ND	ND
PA1421 gbuA	**-3,52**	-0,37	**2,45**
PA1422 gbuR	0,58	ND	ND
PA1423 bdlA	0,44	ND	ND
PA1424 -	-0,07	ND	ND
PA1425 -	0,39	ND	ND
PA1426 -	0,40	ND	ND
PA1427 -	0,31	ND	ND
PA1428 -	0,71	ND	ND
PA1429 -	0,45	ND	ND
PA1430 lasR	-0,07	0,30	0,92
PA1431 rsaL	-0,34	ND	ND
PA1432 lasI	0,16	-0,04	-0,28
PA1433 -	0,04	0,42	**1,14**
PA1434 -	0,64	ND	ND
PA1437 -	-0,30	ND	ND

ID – Gene [a]	TC [b]	PC [c]	PTV[d]
PA3333 fabH2	**-2,46**	ND	ND
PA3334 -	**-4,39**	ND	ND
PA3336 -	-0,65	ND	ND
PA3337 rfaD	-0,89	-0,25	0,17
PA3338 -	-0,87	ND	ND
PA3339 plpD	0,00	ND	ND
PA3340 -	-0,15	0,37	**1,19**
PA3341 -	**-1,06**	0,11	**1,38**
PA3342 -	-0,81	ND	ND
PA3343 -	-0,96	ND	ND
PA3344 recQ	-0,03	-0,15	-0,40
PA3345 -	0,26	-0,12	-0,61
PA3346 -	-0,79	ND	ND
PA3347 -	**-1,03**	ND	ND
PA3348 -	-0,20	-0,03	0,11
PA3349 -	-0,20	-0,24	-0,50
PA3350 -	-0,64	-0,21	0,04
PA3351 flgM	-0,53	0,14	0,93
PA3352 -	-0,30	-0,10	0,03
PA3353 -	-0,24	0,38	**1,31**
PA3354 -	-0,15	ND	ND
PA3355 -	**-1,18**	ND	ND
PA3356 -	-0,85	0,13	**1,21**
PA3357 dsdA	-0,47	-0,30	-0,39
PA3358 -	0,00	ND	ND
PA3359 -	0,28	ND	ND
PA3360 -	**1,49**	ND	ND
PA3362 -	**1,00**	ND	ND
PA3363 amiR	**1,72**	ND	ND
PA3364 amiC	**1,97**	0,98	0,82
PA3365 -	**2,88**	ND	ND
PA3366 amiE	**1,95**	**1,72**	**2,95**
PA3366.1 amiL	**1,69**	ND	ND
PA3367 -	0,08	ND	ND
PA3368.1 -	0,83	ND	ND
PA3369 -	-0,42	ND	ND
PA3370 -	-0,18	ND	ND
PA3371 -	0,93	ND	ND
PA3372 -	-0,39	ND	ND
PA3373 -	-0,08	ND	ND
PA3385 amrZ	**-1,30**	-0,60	-0,41
PA3386 -	**-1,03**	ND	ND
PA3388 -	-0,47	ND	ND
PA3390 -	**-1,19**	ND	ND
PA3396 nosL	0,65	ND	ND
PA3397 fpr	0,02	-0,02	-0,09
PA3398 -	-0,21	ND	ND
PA3399 -	-0,27	ND	ND
PA3400 -	-0,09	ND	ND
PA3401 -	-0,30	ND	ND
PA3402 -	-0,29	0,28	**1,10**
PA3403 -	0,57	ND	ND
PA3407 hasAp	-0,22	ND	ND
PA3410 -	0,00	ND	ND
PA3411 -	0,65	ND	ND
PA3413 -	**-1,62**	-0,61	-0,11
PA3414 -	-0,97	ND	ND
PA3419 -	**-1,39**	ND	ND
PA3423 -	-0,67	ND	ND
PA3425 -	0,46	ND	ND
PA3426 -	0,00	ND	ND
PA3427 -	**-1,00**	ND	ND
PA3431 -	-0,13	ND	ND
PA3432 -	-0,09	ND	ND
PA3433 -	-0,58	ND	ND
PA3434 -	0,00	ND	ND
PA3435 -	-0,51	-0,37	-0,54
PA3436 -	**1,58**	ND	ND
PA3437 folM	-0,83	ND	ND
PA3438 folE1	-0,67	-0,07	0,48
PA3439 folX	-0,86	-0,20	0,30
PA3440 -	-0,18	-0,12	-0,17
PA3441 -	0,02	ND	ND
PA3442 -	0,09	ND	ND
PA3446 -	**-1,14**	ND	ND
PA3450 -	-0,31	-0,06	0,13

ID – Gene [a]	TC [b]	PC [c]	PTV[d]
PA5149 -	-0,25	ND	ND
PA5149.1 -	0,74	ND	ND
PA5150 -	-0,26	ND	ND
PA5151 -	-0,21	ND	ND
PA5152 -	-0,09	**1,20**	**3,51**
PA5153 -	0,80	**1,32**	**2,95**
PA5154 -	**1,37**	0,79	0,87
PA5155 -	**1,22**	ND	ND
PA5156 -	-0,13	ND	ND
PA5157 -	0,22	ND	ND
PA5158 -	0,22	0,20	0,35
PA5159 -	0,23	ND	ND
PA5160 -	0,63	ND	ND
PA5160.1 -	**2,04**	ND	ND
PA5161 rmlB	-0,30	-0,19	-0,25
PA5162 rmlD	-0,29	-0,12	-0,06
PA5163 rmlA	-0,31	-0,23	-0,34
PA5164 rmlC	-0,24	-0,14	-0,17
PA5165 -	-0,37	ND	ND
PA5166 -	-0,03	-0,16	-0,42
PA5167 -	**2,07**	**2,44**	**4,87**
PA5168 -	**1,98**	**2,00**	**3,72**
PA5169 -	**2,83**	ND	ND
PA5170 arcD	-0,11	0,06	0,28
PA5171 arcA	**2,67**	0,11	**-2,35**
PA5172 arcB	**3,36**	0,12	**-3,02**
PA5173 arcC	**3,49**	0,11	**-3,16**
PA5174 -	0,55	0,00	-0,56
PA5175 cysQ	-0,15	-0,07	-0,06
PA5176 -	-0,20	-0,09	-0,05
PA5177 -	-0,22	0,14	0,61
PA5178 -	-0,32	-0,44	-0,94
PA5179 -	-0,36	ND	ND
PA5180 -	-0,64	ND	ND
PA5181 -	-0,81	ND	ND
PA5181.1 -	-0,49	ND	ND
PA5182 -	-0,23	ND	ND
PA5183 -	-0,34	ND	ND
PA5184 -	-0,45	-0,10	0,16
PA5185 -	-0,97	ND	ND
PA5186 -	-0,58	ND	ND
PA5187 -	-0,87	ND	ND
PA5188 -	-0,42	ND	ND
PA5189 -	-0,20	ND	ND
PA5190 -	-0,17	-0,07	-0,03
PA5191 -	-0,30	ND	ND
PA5192 pckA	-0,24	-0,30	-0,61
PA5193 yrfI	-0,18	-0,26	-0,55
PA5194 -	-0,02	ND	ND
PA5195 -	-0,30	ND	ND
PA5196 -	-0,14	ND	ND
PA5197 rimK	-0,34	0,15	0,77
PA5198 -	-0,35	-0,09	0,10
PA5199 amgS	-0,06	0,26	0,79
PA5200 amgR	-0,33	0,01	0,35
PA5201 -	-0,15	-0,26	-0,59
PA5202 -	-0,06	-0,05	-0,08
PA5203 gshA	-0,09	-0,09	-0,18
PA5204 argA	**-1,26**	-0,69	-0,70
PA5205 -	-0,25	ND	ND
PA5206 argE	-0,61	ND	ND
PA5207 -	0,88	ND	ND
PA5208 -	-0,38	-0,10	0,11
PA5209 -	-0,44	-0,08	0,21
PA5210 -	-0,88	-0,86	**-1,57**
PA5211 -	0,00	ND	ND
PA5212 -	-0,11	ND	ND
PA5213 gcvP1	0,18	ND	ND
PA5214 gcvH1	-0,03	0,12	0,36
PA5215 gcvT1	0,26	-0,12	-0,61
PA5216 -	-0,04	ND	ND
PA5217 -	-0,01	0,83	**2,37**
PA5218 -	-0,44	ND	ND
PA5219 -	-0,28	ND	ND
PA5220 -	-0,27	ND	ND
PA5221 -	-0,04	0,09	0,30

ID – Gene [a]	TC [b]	PC [c]	PTV [d]
PA1438 -	0,92	ND	ND
PA1439 -	0,14	ND	ND
PA1440 -	0,31	0,25	0,40
PA1441 -	0,32	0,17	0,18
PA1442 -	0,47	-0,06	-0,64
PA1443 fliM	0,21	0,14	0,18
PA1444 fliN	0,19	0,08	0,04
PA1445 fliO	0,39	ND	ND
PA1446 fliP	0,32	ND	ND
PA1447 fliQ	0,25	ND	ND
PA1448 fliR	0,50	ND	ND
PA1449 flhB	0,49	ND	ND
PA1450 -	0,44	ND	ND
PA1451 -	0,56	ND	ND
PA1452 flhA	0,02	-0,14	-0,43
PA1453 flhF	-0,18	ND	ND
PA1454 fleN	0,17	0,06	-0,01
PA1455 fliA	0,24	0,00	-0,23
PA1456 cheY	0,26	0,06	-0,09
PA1457 cheZ	0,26	0,11	0,06
PA1458 -	0,23	0,15	0,20
PA1459 -	0,29	0,25	0,42
PA1460 motC	0,61	ND	ND
PA1461 motD	0,64	0,23	0,01
PA1462 -	0,39	0,05	-0,26
PA1463 -	0,36	0,09	-0,10
PA1464 -	0,34	0,33	0,60
PA1465 -	0,43	ND	ND
PA1466 -	0,76	ND	ND
PA1467 -	-0,17	ND	ND
PA1468 -	0,47	ND	ND
PA1469 -	-0,09	ND	ND
PA1470 -	0,00	ND	ND
PA1471 -	0,52	ND	ND
PA1472 -	0,69	ND	ND
PA1473 -	0,53	0,24	0,16
PA1474 -	0,69	0,51	0,77
PA1475 ccmA	1,11	0,39	-0,01
PA1476 ccmB	1,24	ND	ND
PA1477 ccmC	0,95	ND	ND
PA1478 -	0,59	ND	ND
PA1479 ccmE	0,57	0,51	0,87
PA1480 ccmF	0,63	0,33	0,30
PA1481 ccmG	0,72	0,54	0,81
PA1482 ccmH	0,64	0,44	0,62
PA1483 cycH	0,76	0,55	0,82
PA1484 -	0,25	ND	ND
PA1487 -	-0,35	ND	ND
PA1488 -	-0,71	ND	ND
PA1489 -	-0,79	ND	ND
PA1490 -	0,22	0,02	-0,18
PA1491 -	-0,13	ND	ND
PA1492 -	-0,35	ND	ND
PA1493 cysP	-0,54	-0,08	0,32
PA1494 -	0,00	0,09	0,26
PA1495 -	0,58	ND	ND
PA1496 -	0,39	ND	ND
PA1501 -	-0,63	ND	ND
PA1504 -	0,30	0,65	1,55
PA1505 moaA2	-1,06	ND	ND
PA1506 -	-1,27	ND	ND
PA1507 -	-0,29	ND	ND
PA1508 -	0,31	ND	ND
PA1509 -	0,42	ND	ND
PA1510 -	0,14	ND	ND
PA1511 -	-0,49	ND	ND
PA1513 -	0,21	0,51	1,25
PA1514 -	0,29	ND	ND
PA1515 alc	0,31	ND	ND
PA1516 -	0,34	0,21	0,27
PA1517 -	0,29	0,26	0,46
PA1518 -	-0,31	-0,16	-0,13
PA1520 -	0,39	0,15	0,04
PA1521 -	-0,13	0,31	1,01
PA1522 -	0,14	ND	ND
PA1523 xdhB	-0,46	ND	ND

ID – Gene [a]	TC [b]	PC [c]	PTV [d]
PA3451 -	-1,12	ND	ND
PA3452 mqoA	-1,45	-0,34	0,50
PA3453 -	-0,10	-0,34	-0,86
PA3454 -	-0,65	ND	ND
PA3455 -	-1,18	-0,37	0,11
PA3456 -	-0,15	-0,10	-0,14
PA3457 -	0,31	ND	ND
PA3458 -	-0,16	ND	ND
PA3459 -	-1,16	ND	ND
PA3463 -	0,13	ND	ND
PA3465 -	-0,57	ND	ND
PA3466 -	-0,31	-0,10	0,01
PA3468 -	-0,76	-0,44	-0,48
PA3469 -	-0,58	ND	ND
PA3470 -	-0,80	ND	ND
PA3471 -	-1,21	-0,54	-0,34
PA3472 -	0,05	ND	ND
PA3473 -	-0,86	ND	ND
PA3474 -	0,00	ND	ND
PA3475 pheC	-0,34	0,21	0,95
PA3476 rhlI	-0,55	ND	ND
PA3477 rhlR	-1,04	-0,40	-0,11
PA3478 rhlB	-1,58	ND	ND
PA3479 rhlA	-2,25	ND	ND
PA3480 -	0,00	-0,29	-0,83
PA3481 -	-0,60	-0,24	-0,09
PA3482 metG	-0,50	-0,12	0,15
PA3483 -	-0,34	-0,12	0,01
PA3484 -	-0,38	-0,25	-0,32
PA3485 -	-0,42	ND	ND
PA3486 -	-0,30	ND	ND
PA3487 pldA	-0,40	ND	ND
PA3488 -	-0,55	ND	ND
PA3489 -	-0,03	ND	ND
PA3490 -	-0,47	ND	ND
PA3491 -	-0,21	ND	ND
PA3492 -	-0,56	ND	ND
PA3493 -	-0,44	ND	ND
PA3494 -	-0,50	ND	ND
PA3495 nth	0,10	ND	ND
PA3496 -	1,11	ND	ND
PA3497 -	0,28	ND	ND
PA3498 -	-0,54	ND	ND
PA3515 -	0,00	ND	ND
PA3524 gloA1	-0,73	-0,59	-0,96
PA3525 argG	-0,22	-0,24	-0,47
PA3526 -	-0,44	-0,16	-0,01
PA3527 pyrC	-0,22	-0,08	0,00
PA3528 rnt	-0,18	0,08	0,40
PA3529 -	-0,65	-0,69	-1,31
PA3530 -	1,30	ND	ND
PA3531 bfrB	-1,23	-0,77	-0,96
PA3532 -	-0,77	ND	ND
PA3533 -	-1,27	-0,09	1,01
PA3535 -	-0,06	ND	ND
PA3536 -	-0,37	ND	ND
PA3537 argF	-0,83	-0,33	-0,10
PA3538 -	-0,69	-0,26	-0,04
PA3539 -	-0,52	-0,45	-0,77
PA3545 algG	-1,58	ND	ND
PA3552 arnB	-0,42	-0,41	-0,75
PA3553 arnC	-0,41	-0,08	0,19
PA3554 arnA	-0,24	-0,32	-0,67
PA3555 arnD	0,10	ND	ND
PA3556 arnT	-0,17	ND	ND
PA3557 arnE	-0,18	ND	ND
PA3558 arnF	-0,08	ND	ND
PA3559 -	-0,37	ND	ND
PA3560 fruA	0,04	-0,23	-0,70
PA3561 fruK	-0,06	0,31	0,94
PA3562 fruI	-0,11	0,28	0,90
PA3563 fruR	-0,39	0,09	0,64
PA3564 -	-0,75	ND	ND
PA3565 -	-1,09	ND	ND
PA3566 -	-0,90	-0,25	0,18
PA3567 -	-0,70	-0,25	-0,01

ID – Gene [a]	TC [b]	PC [c]	PTV [d]
PA5222 -	0,26	ND	ND
PA5223 ubiH	-0,06	-0,03	-0,03
PA5224 pepP	-0,27	-0,08	0,03
PA5225 -	-0,45	-0,02	0,38
PA5226 -	-0,73	ND	ND
PA5227 -	-0,62	0,04	0,74
PA5227.1 ssrS	0,46	ND	ND
PA5228 -	-0,30	ND	ND
PA5229 -	0,70	-0,19	-1,25
PA5230 -	0,95	ND	ND
PA5231 -	0,73	0,03	-0,64
PA5232 -	-0,19	-0,27	-0,57
PA5233 -	-0,52	ND	ND
PA5234 -	-0,61	-0,18	0,09
PA5235 glpT	-1,57	-0,34	0,60
PA5236 -	-0,51	-0,09	0,25
PA5237 -	-0,31	-0,11	0,00
PA5238 -	-0,29	ND	ND
PA5239 rho	-0,38	-0,19	-0,16
PA5240 trxA	-0,39	-0,20	-0,18
PA5241 ppx	-0,36	-0,19	-0,17
PA5242 ppk	-0,15	0,24	0,83
PA5243 hemB	-0,33	-0,14	-0,07
PA5244 -	0,01	ND	ND
PA5245 -	-0,47	0,00	0,45
PA5246 -	-0,17	ND	ND
PA5247 -	-0,15	ND	ND
PA5248 -	0,32	0,19	0,23
PA5249 -	-0,68	ND	ND
PA5250 -	0,15	ND	ND
PA5251 -	0,09	ND	ND
PA5252 -	0,07	-0,02	-0,13
PA5253 algP	-0,63	-0,36	-0,39
PA5254 -	-0,25	ND	ND
PA5255 algQ	-0,65	-0,13	0,30
PA5256 dsbH	0,16	ND	ND
PA5257 -	-0,07	0,01	0,09
PA5258 -	-0,05	-0,05	-0,09
PA5259 hemD	-0,06	0,25	0,77
PA5260 hemC	-0,39	0,05	0,54
PA5261 algR	-0,36	-0,33	-0,58
PA5262 algZ	-0,93	ND	ND
PA5263 argH	-0,44	-0,13	0,07
PA5264 -	-0,32	ND	ND
PA5265 -	-0,87	ND	ND
PA5267 hcpB	-0,29	ND	ND
PA5268 corA	-0,57	ND	ND
PA5269 -	-0,62	0,01	0,66
PA5270 -	-0,26	ND	ND
PA5271 -	-0,60	ND	ND
PA5272 cyaA	0,10	ND	ND
PA5273 -	-0,62	ND	ND
PA5274 rnk	0,07	-0,07	-0,27
PA5275 -	-0,58	0,17	1,06
PA5276 lppL	0,00	ND	ND
PA5277 lysA	-0,06	0,03	0,15
PA5278 dapF	-0,05	0,09	0,30
PA5279 -	-0,24	-0,13	-0,12
PA5280 sss	-0,23	ND	ND
PA5281 -	-0,04	ND	ND
PA5283 -	-0,58	ND	ND
PA5284 -	0,12	ND	ND
PA5285 -	0,05	0,13	0,33
PA5286 -	0,67	ND	ND
PA5287 amtB	-0,39	ND	ND
PA5288 glnK	-0,94	-0,66	-0,95
PA5289 -	-0,01	-0,14	-0,40
PA5290 -	-0,42	ND	ND
PA5291 -	-0,97	-0,65	-0,86
PA5292 pchP	-0,95	ND	ND
PA5293 -	-0,71	ND	ND
PA5294 -	-0,45	ND	ND
PA5295 -	-0,35	ND	ND
PA5296 rep	0,24	0,12	0,11
PA5298 -	0,46	0,24	0,22
PA5299 -	0,20	ND	ND

ID – Gene [a]	TC [b]	PC [c]	PTV[d]
PA1524 xdhA	-0,92	ND	ND
PA1526 -	0,22	0,57	1,41
PA1527 -	0,57	0,33	0,37
PA1528 zipA	0,28	0,35	0,73
PA1529 lig	0,21	0,35	0,78
PA1530 -	0,53	0,45	0,75
PA1530.1 ffs	0,18	ND	ND
PA1531 -	0,14	ND	ND
PA1532 dnaX	0,36	0,23	0,30
PA1533 -	0,14	0,28	0,65
PA1534 recR	0,30	0,07	-0,09
PA1535 -	0,00	ND	ND
PA1536 -	-0,11	ND	ND
PA1539 -	0,25	ND	ND
PA1540 -	0,30	ND	ND
PA1542 -	1,08	ND	ND
PA1543 apt	0,67	0,19	-0,13
PA1544 anr	0,51	-0,06	-0,67
PA1545 -	0,40	ND	ND
PA1546 hemN	0,68	0,04	-0,56
PA1547 -	0,58	ND	ND
PA1548 -	1,34	ND	ND
PA1549 -	0,97	ND	ND
PA1550 -	0,91	0,62	0,85
PA1551 -	0,83	0,78	1,40
PA1552 ccoP1	-0,12	0,39	1,23
PA1552.1 ccoQ1	0,05	0,74	2,07
PA1553 ccoO1	0,02	0,29	0,81
PA1554 ccoN1	-0,03	0,45	1,30
PA1555 ccoP2	3,09	0,28	-2,28
PA1555.1 ccoQ2	1,78	ND	ND
PA1556 ccoO2	2,84	0,09	-2,59
PA1557 ccoN2	1,76	ND	ND
PA1558 -	0,35	0,31	0,54
PA1559 -	0,42	ND	ND
PA1560 -	0,30	ND	ND
PA1561 aer	0,70	0,09	-0,43
PA1562 acnA	0,09	-0,07	-0,30
PA1563 -	0,61	0,10	-0,33
PA1564 -	0,15	0,17	0,32
PA1565 -	-0,43	0,18	0,93
PA1566 -	-1,00	ND	ND
PA1570 -	-0,36	ND	ND
PA1571 -	0,33	ND	ND
PA1572 -	-0,21	0,77	2,40
PA1573 -	0,43	ND	ND
PA1574 -	0,25	0,33	0,69
PA1575 -	0,00	ND	ND
PA1576 -	-0,10	0,05	0,25
PA1577 -	0,58	ND	ND
PA1578 -	0,00	ND	ND
PA1579 -	-0,74	0,03	0,84
PA1580 gltA	-0,51	-0,39	-0,60
PA1581 sdhC	0,46	0,02	-0,39
PA1582 sdhD	0,35	0,14	0,04
PA1583 sdhA	0,22	0,04	-0,10
PA1584 sdhB	0,12	0,02	-0,06
PA1585 sucA	0,23	0,24	0,46
PA1586 sucB	0,29	0,31	0,59
PA1587 lpdG	0,33	0,27	0,45
PA1588 sucC	0,52	0,49	0,87
PA1589 sucD	0,49	0,44	0,76
PA1590 braB	0,66	ND	ND
PA1591 -	1,89	0,35	-0,88
PA1592 -	0,57	ND	ND
PA1593 -	0,65	ND	ND
PA1594 -	0,63	ND	ND
PA1595 -	0,78	ND	ND
PA1596 htpG	0,35	0,13	0,02
PA1597 -	0,45	-0,15	-0,89
PA1598 -	0,22	ND	ND
PA1599 -	-0,47	ND	ND
PA1600 -	-2,46	-0,12	2,11
PA1601 -	-3,48	-0,58	1,84

ID – Gene [a]	TC [b]	PC [c]	PTV[d]
PA3570 mmsA	0,65	ND	ND
PA3571 mmsR	-0,83	ND	ND
PA3572 -	-0,65	ND	ND
PA3573 -	-0,30	ND	ND
PA3574 nalD	-0,33	-0,41	-0,82
PA3575 -	0,57	ND	ND
PA3576 -	-0,83	ND	ND
PA3577 -	-0,21	ND	ND
PA3578 -	-2,12	ND	ND
PA3579 -	-1,76	-0,23	1,10
PA3580 -	-1,80	ND	ND
PA3581 glpF	-2,04	ND	ND
PA3582 glpK	-1,93	1,44	6,02
PA3583 glpR	-1,06	-0,14	0,66
PA3584 glpD	-2,87	0,16	3,31
PA3585 glpM	1,22	ND	ND
PA3586 -	0,83	ND	ND
PA3587 metR	-1,29	ND	ND
PA3594 -	-0,74	ND	ND
PA3599 -	-0,71	ND	ND
PA3600 -	-1,24	ND	ND
PA3601 -	-1,18	ND	ND
PA3602 -	-0,90	-0,53	-0,59
PA3603 dgkA	0,14	ND	ND
PA3604 erdR	-0,09	-0,30	-0,75
PA3605 -	-0,43	ND	ND
PA3606 -	0,13	ND	ND
PA3607 potA	-0,23	-0,41	-0,93
PA3608 potB	-0,64	ND	ND
PA3609 potC	-0,40	ND	ND
PA3610 potD	-0,57	-0,14	0,17
PA3611 -	-0,03	-0,03	-0,04
PA3612 -	0,32	ND	ND
PA3613 -	0,12	-0,42	-1,31
PA3614 -	-1,48	-0,17	0,98
PA3615 -	-0,92	-0,16	0,48
PA3616 -	-0,79	ND	ND
PA3617 recA	-0,84	-0,63	-0,94
PA3618 -	-1,30	-0,36	0,28
PA3619 -	-1,14	ND	ND
PA3620 mutS	-0,54	-0,26	-0,21
PA3621 fdxA	-0,14	-0,22	-0,47
PA3621.1 rsmZ	0,92	ND	ND
PA3622 rpoS	-0,51	ND	ND
PA3623 -	-0,58	-0,18	0,06
PA3624 pcm	-0,31	-0,32	-0,60
PA3625 surE	-0,08	-0,23	-0,58
PA3626 -	-0,13	-0,14	-0,27
PA3627 ygbB	0,00	-0,07	-0,19
PA3628 -	-0,83	ND	ND
PA3629 adhC	-1,03	-0,44	-0,22
PA3630 -	-0,58	ND	ND
PA3631 -	-0,27	-0,01	0,24
PA3632 -	-0,02	ND	ND
PA3633 ygbP	0,02	-0,18	-0,53
PA3634 -	0,03	ND	ND
PA3635 eno	-0,05	-0,11	-0,25
PA3636 kdsA	0,13	0,09	0,13
PA3637 pyrG	0,26	-0,02	-0,31
PA3638 -	0,22	ND	ND
PA3639 accA	0,12	-0,34	-1,09
PA3640 dnaE	-0,25	-0,03	0,16
PA3641 -	-0,40	-0,21	-0,19
PA3642 rnhB	-0,33	-0,10	0,06
PA3643 lpxB	0,21	0,06	-0,04
PA3644 lpxA	-0,04	-0,12	-0,29
PA3645 fabZ	0,11	0,15	0,32
PA3646 lpxD	0,01	0,14	0,38
PA3647 -	-0,09	-0,10	-0,18
PA3648 opr86	-0,17	-0,36	-0,85
PA3649 -	0,12	0,08	0,11
PA3650 dxr	-0,01	-0,05	-0,14
PA3651 cdsA	0,05	ND	ND
PA3652 uppS	-0,14	-0,19	-0,40

ID – Gene [a]	TC [b]	PC [c]	PTV[d]
PA5300 cycB	0,10	0,12	0,26
PA5301 -	-0,23	0,03	0,33
PA5302 dadX	1,34	1,41	2,67
PA5303 -	1,33	0,82	0,99
PA5304 dadA	0,99	1,32	2,76
PA5305 -	-0,67	-0,14	0,26
PA5306 -	-0,40	ND	ND
PA5307 -	-0,55	ND	ND
PA5308 lrp	-0,80	-0,21	0,20
PA5309 -	-0,73	0,14	1,14
PA5310 -	0,21	ND	ND
PA5311 -	-1,13	ND	ND
PA5312 -	-1,49	0,20	2,07
PA5313 -	-1,18	-0,28	0,40
PA5314 -	-1,41	ND	ND
PA5315 rpmG	-0,29	-0,31	-0,60
PA5316 rpmB	-0,17	-0,13	-0,19
PA5316.1 -	0,17	ND	ND
PA5317 -	-0,84	-0,31	-0,04
PA5318 -	-0,82	ND	ND
PA5319 radC	-0,40	ND	ND
PA5320 coaC	-0,13	-0,07	-0,06
PA5321 dut	-0,13	0,21	0,72
PA5322 algC	-0,18	-0,09	-0,06
PA5323 argB	-0,09	-0,04	-0,01
PA5324 -	0,24	ND	ND
PA5329 -	-0,39	ND	ND
PA5330 -	0,52	-0,44	-1,78
PA5331 pyrE	1,06	-1,20	-4,48
PA5333 -	-0,08	ND	ND
PA5334 rph	-0,28	-0,12	-0,08
PA5335 -	-0,04	-0,04	-0,07
PA5336 gmk	-0,10	-0,06	-0,08
PA5337 rpoZ	-0,18	-0,26	-0,56
PA5338 spoT	-0,01	0,21	0,61
PA5339 -	-0,14	0,06	0,32
PA5340 -	0,04	-0,05	-0,18
PA5341 -	0,07	ND	ND
PA5342 -	-0,44	ND	ND
PA5343 -	-0,58	-0,25	-0,12
PA5344 oxyR	-0,50	-0,17	0,01
PA5345 recG	-0,47	-0,01	0,44
PA5346 -	-0,77	0,05	0,91
PA5347 -	0,23	ND	ND
PA5348 -	1,26	2,09	4,68
PA5349 -	-0,68	-0,45	-0,61
PA5350 rubA2	-0,29	ND	ND
PA5351 rubA1	-0,23	ND	ND
PA5356 glcC	-1,47	ND	ND
PA5357 -	-0,18	-0,07	-0,03
PA5358 ubiA	0,08	ND	ND
PA5359 -	-0,39	ND	ND
PA5360 phoB	0,13	0,20	0,43
PA5361 phoR	-0,29	-0,03	0,21
PA5362 -	-0,34	-0,09	0,08
PA5363 -	-0,82	ND	ND
PA5364 -	-0,58	0,01	0,59
PA5365 phoU	-0,63	0,13	0,99
PA5366 pstB	-0,44	0,24	1,13
PA5367 pstA	0,00	0,50	1,42
PA5368 pstC	0,00	0,21	0,59
PA5369 pstS	-0,22	0,28	1,02
PA5369.3 -	0,06	ND	ND
PA5369.4 -	0,20	ND	ND
PA5370 -	-0,06	ND	ND
PA5371 -	-0,20	ND	ND
PA5372 betA	-0,64	ND	ND
PA5373 betB	-0,94	-0,58	-0,70
PA5374 betI	-0,54	-0,28	-0,26
PA5376 -	-0,36	-0,41	-0,80
PA5377 -	-0,50	ND	ND
PA5378 -	-0,75	-0,33	-0,20
PA5380 gbdR	0,09	ND	ND
PA5381 -	0,19	ND	ND

ID – Gene [a]	TC [b]	PC [c]	PTV[d]
PA1602 -	**-3,44**	ND	ND
PA1603 -	0,42	ND	ND
PA1604 -	-0,49	ND	ND
PA1605 -	-0,87	ND	ND
PA1606 -	-0,29	ND	ND
PA1607 -	0,11	ND	ND
PA1608 -	0,66	0,50	0,77
PA1609 fabB	0,78	0,09	-0,54
PA1610 fabA	0,92	0,21	-0,32
PA1611 -	0,55	0,19	-0,02
PA1612 -	0,46	ND	ND
PA1613 -	0,34	ND	ND
PA1614 gpsA	0,38	0,14	0,00
PA1615 -	0,22	ND	ND
PA1616 -	0,33	0,59	**1,36**
PA1617 -	0,50	ND	ND
PA1618 -	-0,14	-0,02	0,09
PA1619 -	0,36	ND	ND
PA1621 -	0,49	ND	ND
PA1622 -	0,64	ND	ND
PA1623 -	-0,30	0,25	**1,00**
PA1624 -	0,28	0,27	0,48
PA1625 -	0,14	ND	ND
PA1626 -	0,00	ND	ND
PA1627 -	0,30	ND	ND
PA1628 -	0,42	ND	ND
PA1629 -	0,30	ND	ND
PA1630 -	0,09	ND	ND
PA1631 -	-0,42	ND	ND
PA1632 kdpF	**2,83**	ND	ND
PA1633 kdpA	**2,90**	ND	ND
PA1634 kdpB	**3,46**	**1,19**	-0,06
PA1635 kdpC	**3,00**	ND	ND
PA1636 kdpD	**1,91**	0,67	-0,01
PA1637 kdpE	0,68	ND	ND
PA1638 -	0,53	ND	ND
PA1639 -	-0,03	-0,21	-0,58
PA1640 -	0,07	0,10	0,22
PA1641 -	0,00	ND	ND
PA1642 selD	0,29	0,26	0,46
PA1643 -	0,42	0,27	0,34
PA1644 -	0,20	0,09	0,04
PA1645 -	0,05	ND	ND
PA1647 -	0,46	ND	ND
PA1649 -	0,00	ND	ND
PA1650 -	0,71	ND	ND
PA1651 -	0,84	ND	ND
PA1652 -	0,69	ND	ND
PA1653 -	0,52	ND	ND
PA1654 -	0,47	0,55	**1,09**
PA1655 -	0,56	0,24	0,14
PA1656 -	0,43	ND	ND
PA1657 -	0,61	-0,27	**-1,37**
PA1658 -	0,61	-0,38	**-1,71**
PA1659 -	**1,09**	ND	ND
PA1660 -	0,95	ND	ND
PA1661 -	**1,40**	ND	ND
PA1662 -	**1,00**	-0,03	**-1,07**
PA1663 -	**1,24**	ND	ND
PA1664 -	**1,54**	ND	ND
PA1665 -	**1,00**	ND	ND
PA1666 -	**1,40**	ND	ND
PA1667 -	0,88	ND	ND
PA1668 -	**1,00**	ND	ND
PA1669 -	**1,00**	ND	ND
PA1670 stp1	**1,22**	ND	ND
PA1671 stk1	0,79	ND	ND
PA1672 -	-0,30	ND	ND
PA1673 -	0,86	0,33	0,08
PA1674 folE2	0,57	0,21	0,03
PA1675 -	0,79	0,14	-0,38
PA1676 -	-0,19	ND	ND
PA1677 -	-0,80	-0,33	-0,14
PA1678 -	0,62	0,32	0,30
PA1679 -	0,27	ND	ND
PA1681 aroC	0,01	0,12	0,34

ID – Gene [a]	TC [b]	PC [c]	PTV[d]
PA3653 frr	-0,44	-0,35	-0,56
PA3654 pyrH	-0,33	-0,45	-0,95
PA3655 tsf	-0,29	-0,19	-0,25
PA3656 rpsB	-0,12	-0,09	-0,13
PA3657 map	-0,37	-0,28	-0,43
PA3658 glnD	-0,33	ND	ND
PA3659 -	-0,67	-0,27	-0,10
PA3660 -	**-1,28**	ND	ND
PA3661 -	0,00	ND	ND
PA3662 -	**-1,27**	ND	ND
PA3663 -	-0,53	ND	ND
PA3664 -	-0,58	-0,04	0,47
PA3665 -	-0,49	ND	ND
PA3666 dapD	-0,65	-0,40	-0,48
PA3667 -	-0,38	0,09	0,63
PA3668 -	-0,25	ND	ND
PA3669 -	0,00	ND	ND
PA3670 -	-0,14	ND	ND
PA3671 -	-0,45	ND	ND
PA3672 -	-0,10	-0,01	0,06
PA3673 plsB	-0,21	-0,26	-0,53
PA3674 -	-0,92	-0,55	-0,65
PA3675 -	-0,25	-0,19	-0,30
PA3676 -	0,23	ND	ND
PA3677 -	**1,06**	ND	ND
PA3678 -	-0,13	-0,10	-0,17
PA3679 -	-0,06	ND	ND
PA3680 -	-0,23	ND	ND
PA3681 -	0,00	ND	ND
PA3683 -	-0,39	ND	ND
PA3684 -	-0,42	ND	ND
PA3685 -	-0,27	-0,18	-0,24
PA3686 adk	**-1,18**	-0,56	-0,42
PA3687 ppc	-0,89	-0,40	-0,25
PA3688 -	-0,29	ND	ND
PA3689 -	-0,87	ND	ND
PA3690 -	-0,46	**1,02**	**3,36**
PA3691 -	**-1,02**	-0,11	0,71
PA3692 lptF	-0,69	-0,53	-0,80
PA3693 -	-0,33	ND	ND
PA3694 -	0,09	-0,07	-0,30
PA3695 -	-0,25	ND	ND
PA3696 -	-0,79	-0,23	0,13
PA3697 -	-0,67	ND	ND
PA3698 -	-0,69	ND	ND
PA3699 -	-0,27	ND	ND
PA3700 lysS	-0,19	-0,12	-0,15
PA3701 prfB	-0,20	ND	ND
PA3702 wspR	-0,30	-0,15	-0,12
PA3703 wspF	-0,33	ND	ND
PA3704 wspE	-0,45	0,05	0,59
PA3705 wspD	-0,42	-0,61	**-1,33**
PA3706 wspC	-0,33	-0,37	-0,72
PA3707 wspB	-0,31	ND	ND
PA3708 wspA	-0,53	-0,22	-0,09
PA3711 -	-0,35	ND	ND
PA3712 -	**-1,55**	ND	ND
PA3713 spdH	**-1,44**	-0,72	-0,60
PA3714 -	-0,92	ND	ND
PA3715 -	-0,39	-0,01	0,36
PA3716 -	-0,44	-0,46	-0,87
PA3717 -	-0,18	-0,19	-0,37
PA3719 armR	**-1,94**	ND	ND
PA3720 -	**-1,78**	ND	ND
PA3721 nalC	0,30	ND	ND
PA3722 -	0,72	ND	ND
PA3724 lasB	-0,87	ND	ND
PA3725 recJ	-0,40	-0,13	0,04
PA3726 -	-0,38	-0,19	-0,17
PA3727 -	-0,16	ND	ND
PA3728 -	-0,34	-0,24	-0,35
PA3729 -	-0,63	-0,37	-0,43
PA3730 -	-0,52	-0,05	0,38
PA3731 -	-0,53	-0,44	-0,72
PA3732 -	-0,59	-0,46	-0,71
PA3733 -	-0,31	-0,35	-0,69

ID – Gene [a]	TC [b]	PC [c]	PTV[d]
PA5382 -	-0,51	ND	ND
PA5389 cdhR	-0,65	ND	ND
PA5390 -	-0,29	ND	ND
PA5394 cls	-0,49	ND	ND
PA5396 -	**-1,17**	ND	ND
PA5397 -	**-1,46**	ND	ND
PA5402 -	**-1,28**	ND	ND
PA5403 -	-0,66	ND	ND
PA5404 -	**-1,49**	ND	ND
PA5405 -	**-1,78**	ND	ND
PA5406 -	-0,20	-0,31	-0,69
PA5407 -	-0,45	ND	ND
PA5408 -	-0,89	ND	ND
PA5409 -	**-1,35**	ND	ND
PA5410 gbcA	-0,65	ND	ND
PA5411 gbcB	-0,58	ND	ND
PA5412 -	-0,39	0,02	0,45
PA5413 ltaA	-0,18	0,19	0,73
PA5414 -	-0,30	0,09	0,55
PA5415 glyA1	**2,17**	ND	ND
PA5422 -	-0,41	-0,12	0,08
PA5423 -	0,02	0,42	**1,18**
PA5424 -	-0,34	ND	ND
PA5425 purK	-0,04	-0,12	-0,29
PA5426 purE	0,18	-0,09	-0,43
PA5427 adhA	-0,01	-0,12	-0,33
PA5428 -	-0,11	-0,01	0,09
PA5429 aspA	-0,05	0,60	**1,76**
PA5430 -	0,09	0,25	0,62
PA5431 -	-0,65	ND	ND
PA5432 -	0,42	ND	ND
PA5433 -	0,27	ND	ND
PA5434 mtr	0,40	ND	ND
PA5435 -	**1,69**	**1,05**	**1,29**
PA5436 -	**1,09**	0,88	**1,40**
PA5437 -	-0,20	-0,13	-0,18
PA5438 -	-0,35	-0,13	-0,01
PA5439 -	-0,08	ND	ND
PA5440 -	0,77	-0,05	-0,90
PA5441 -	-0,09	-0,02	0,03
PA5442 -	**1,68**	ND	ND
PA5443 uvrD	-0,38	-0,11	0,06
PA5444 -	-0,49	ND	ND
PA5445 -	**-4,28**	**-1,26**	0,68
PA5446 -	**2,21**	**1,10**	0,94
PA5447 wbpZ	**1,42**	ND	ND
PA5448 wbpY	0,23	ND	ND
PA5449 wbpX	0,73	ND	ND
PA5450 wzt	0,55	ND	ND
PA5451 wzm	0,69	ND	ND
PA5452 wbpW	0,26	ND	ND
PA5453 gmd	0,68	ND	ND
PA5454 rmd	-0,12	ND	ND
PA5455 -	0,31	ND	ND
PA5456 -	0,42	0,55	**1,14**
PA5457 -	0,50	0,32	0,40
PA5458 -	0,51	ND	ND
PA5459 -	0,62	0,80	**1,65**
PA5460 -	-0,36	ND	ND
PA5461 -	0,69	ND	ND
PA5462 -	0,10	ND	ND
PA5463 -	-0,17	ND	ND
PA5464 -	-0,25	ND	ND
PA5465 -	-0,56	ND	ND
PA5466 -	**-1,28**	ND	ND
PA5467 -	-0,95	ND	ND
PA5468 -	**-1,44**	ND	ND
PA5469 -	-0,70	ND	ND
PA5470 -	-0,63	ND	ND
PA5471 -	-0,19	ND	ND
PA5471.1 -	-0,19	ND	ND
PA5472 -	-0,58	-0,60	**-1,13**
PA5473 -	-0,79	ND	ND
PA5474 -	**-1,09**	ND	ND
PA5475 -	**-1,55**	-0,33	0,62
PA5476 citA	-0,89	ND	ND

ID – Gene [a]	TC [b]	PC [c]	PTV[d]
PA1682 -	0,25	ND	ND
PA1683 -	0,47	0,06	-0,31
PA1684 -	0,42	0,13	-0,06
PA1685 masA	0,64	0,19	-0,10
PA1686 alkA	0,23	0,27	0,54
PA1687 speE	0,42	-0,02	-0,48
PA1688 -	0,79	0,39	0,30
PA1689 -	0,37	0,63	**1,43**
PA1692 -	-0,31	ND	ND
PA1693 pscR	-0,35	ND	ND
PA1694 pscQ	-0,58	ND	ND
PA1695 pscP	-0,54	ND	ND
PA1696 pscO	**-1,54**	ND	ND
PA1697 -	**-1,03**	ND	ND
PA1698 popN	**-1,03**	ND	ND
PA1699 -	**-1,12**	ND	ND
PA1700 -	**-1,27**	ND	ND
PA1701 -	**-1,47**	ND	ND
PA1702 -	**-1,06**	ND	ND
PA1703 pcrD	**-1,24**	ND	ND
PA1704 pcrR	**-1,28**	ND	ND
PA1705 pcrG	**-1,64**	ND	ND
PA1706 pcrV	**-1,67**	ND	ND
PA1707 pcrH	**-1,50**	ND	ND
PA1708 popB	**-1,32**	ND	ND
PA1709 popD	**-1,27**	ND	ND
PA1710 exsC	-0,65	ND	ND
PA1711 exsE	-0,64	ND	ND
PA1712 exsB	-0,40	ND	ND
PA1713 exsA	-0,88	ND	ND
PA1714 exsD	-0,65	-0,77	**-1,54**
PA1715 pscB	-0,95	ND	ND
PA1716 pscC	-0,90	ND	ND
PA1717 pscD	-0,89	ND	ND
PA1718 pscE	**-1,03**	0,28	**1,82**
PA1719 pscF	**-1,30**	ND	ND
PA1720 pscG	-0,80	ND	ND
PA1721 pscH	**-1,16**	ND	ND
PA1722 pscI	**-1,08**	ND	ND
PA1723 pscJ	-0,80	ND	ND
PA1724 pscK	-0,68	ND	ND
PA1725 pscL	-0,58	ND	ND
PA1726 bglX	0,50	ND	ND
PA1727 mucR	0,76	ND	ND
PA1728 -	0,00	ND	ND
PA1729 -	0,09	ND	ND
PA1733 -	-0,24	ND	ND
PA1734 -	-0,63	ND	ND
PA1735 -	0,56	ND	ND
PA1736 -	0,65	ND	ND
PA1737 -	0,70	ND	ND
PA1738 -	-0,10	-0,07	-0,10
PA1740 -	-0,45	ND	ND
PA1741 -	-0,34	ND	ND
PA1742 -	-0,68	0,55	**2,23**
PA1746 -	0,32	0,19	0,23
PA1747 -	**1,09**	ND	ND
PA1748 -	0,39	0,15	0,03
PA1749 -	0,21	0,20	0,35
PA1750 -	0,26	0,11	0,04
PA1751 -	0,11	ND	ND
PA1752 -	0,46	0,07	-0,26
PA1753 -	0,79	ND	ND
PA1754 cysB	-0,09	0,00	0,10
PA1755 -	0,31	ND	ND
PA1756 cysH	**1,01**	0,50	0,42
PA1757 thrH	0,63	0,20	-0,05
PA1758 pabB	0,16	0,22	0,46
PA1759 -	0,61	ND	ND
PA1760 -	**1,28**	0,83	**1,07**
PA1761 -	0,92	ND	ND
PA1762 -	0,93	ND	ND
PA1763 -	0,29	ND	ND
PA1766 -	0,78	0,64	**1,04**
PA1767 -	0,74	0,57	0,87

ID – Gene [a]	TC [b]	PC [c]	PTV[d]
PA3735 thrC	-0,55	-0,48	-0,80
PA3736 hom	-0,39	-0,47	-0,96
PA3737 dsbC	-0,28	-0,18	-0,22
PA3738 xerD	-0,51	-0,16	0,06
PA3739 -	-0,78	ND	ND
PA3740 -	-0,55	ND	ND
PA3741 -	-0,42	ND	ND
PA3742 rplS	-0,23	-0,03	0,14
PA3743 trmD	0,20	0,02	-0,15
PA3744 rimM	0,26	-0,14	-0,66
PA3745 rpsP	0,15	-0,21	-0,73
PA3746 ffh	-0,19	-0,27	-0,59
PA3747 -	-0,33	ND	ND
PA3748 -	-0,14	0,06	0,32
PA3750 -	-0,13	ND	ND
PA3751 purT	-0,50	-0,31	-0,38
PA3752 -	-0,75	ND	ND
PA3753 -	-0,54	ND	ND
PA3754 -	**-1,16**	ND	ND
PA3755 -	**-1,05**	ND	ND
PA3756 -	-0,32	ND	ND
PA3757 -	-0,35	ND	ND
PA3758 -	-0,71	ND	ND
PA3759 -	-0,45	0,11	0,75
PA3760 -	-0,50	0,44	**1,76**
PA3761 -	-0,24	0,24	0,92
PA3762 -	**-2,17**	ND	ND
PA3763 purL	-0,08	-0,03	-0,02
PA3764 -	-0,43	ND	ND
PA3765 -	-0,37	-0,23	-0,29
PA3766 -	**-2,72**	ND	ND
PA3767 -	-0,07	ND	ND
PA3768 -	0,04	0,01	-0,01
PA3769 guaA	-0,30	-0,19	-0,22
PA3770 guaB	-0,15	-0,13	-0,22
PA3777 xseA	-0,21	ND	ND
PA3778 -	-0,15	ND	ND
PA3779 -	0,00	0,89	**2,54**
PA3780 -	-0,13	ND	ND
PA3781 -	0,13	ND	ND
PA3782 -	-0,37	ND	ND
PA3783 -	-0,44	ND	ND
PA3784 -	0,27	ND	ND
PA3785 -	0,87	0,63	0,93
PA3786 -	-0,21	ND	ND
PA3787 -	-0,30	-0,07	0,08
PA3788 -	-0,43	ND	ND
PA3789 -	-0,11	-0,36	-0,91
PA3790 oprC	0,39	-0,21	-0,98
PA3791 -	-0,08	ND	ND
PA3792 leuA	-0,73	-0,21	0,13
PA3793 -	-0,51	ND	ND
PA3794 -	-0,58	ND	ND
PA3795 -	-0,29	-0,06	0,13
PA3796 -	-0,03	ND	ND
PA3797 -	-0,57	ND	ND
PA3798 -	-0,62	-0,41	-0,56
PA3799 -	-0,16	-0,13	-0,22
PA3800 -	-0,28	-0,07	0,08
PA3801 -	-0,24	-0,06	0,08
PA3802 hisS	-0,03	0,01	0,06
PA3803 gcpE	0,01	-0,09	-0,26
PA3804 -	-0,03	-0,12	-0,31
PA3805 pilF	-0,06	-0,23	-0,58
PA3806 -	-0,33	0,01	0,36
PA3807 ndk	-0,42	-0,14	0,01
PA3808 -	-0,23	-0,01	0,19
PA3809 fdx2	-0,01	ND	ND
PA3810 hscA	-0,36	0,08	0,57
PA3811 hscB	-0,32	-0,06	0,15
PA3812 iscA	-0,32	0,07	0,51
PA3813 iscU	-0,43	-0,10	0,13
PA3814 iscS	-0,44	-0,16	-0,01
PA3815 iscR	0,03	0,05	0,12
PA3816 cysE	-0,66	-0,19	0,11

ID – Gene [a]	TC [b]	PC [c]	PTV[d]
PA5477 -	-0,15	ND	ND
PA5478 -	-0,07	ND	ND
PA5479 gltP	-0,36	-0,18	-0,16
PA5480 -	-0,42	ND	ND
PA5481 -	**-2,07**	ND	ND
PA5482 -	**-1,79**	ND	ND
PA5483 algB	-0,45	-0,03	0,36
PA5484 -	-0,50	-0,02	0,46
PA5485 ampDh2	-0,15	-0,14	-0,26
PA5486 -	-0,19	ND	ND
PA5487 -	0,14	0,06	0,03
PA5488 -	-0,03	ND	ND
PA5489 dsbA	-0,32	0,00	0,32
PA5490 cc4	0,15	0,19	0,39
PA5491 -	0,69	ND	ND
PA5492 -	-0,30	-0,01	0,28
PA5493 polA	-0,46	-0,21	-0,13
PA5494 -	-0,58	ND	ND
PA5495 thrB	-0,36	-0,11	0,06
PA5496 nrdJb	0,93	ND	ND
PA5497 nrdJa	-0,29	-0,17	-0,18
PA5498 -	0,06	-0,04	-0,17
PA5499 np20	-0,50	ND	ND
PA5500 znuC	-0,11	ND	ND
PA5501 znuB	0,00	ND	ND
PA5502 -	0,28	0,02	-0,22
PA5503 -	0,13	0,16	0,34
PA5504 -	-0,19	0,29	**1,01**
PA5505 -	-0,30	-0,03	0,21
PA5506 -	**-4,96**	0,76	**7,14**
PA5507 -	**-4,71**	0,87	**7,19**
PA5508 -	**-4,03**	0,80	**6,32**
PA5509 -	**-3,43**	0,81	**5,74**
PA5510 -	**-2,56**	0,44	**3,82**
PA5511 mifR	-0,29	-0,08	0,06
PA5512 mifS	-0,28	ND	ND
PA5513 poxA	0,31	ND	ND
PA5514 -	-0,58	ND	ND
PA5515 -	-0,78	ND	ND
PA5516 pdxY	-0,23	-0,17	-0,24
PA5517 -	0,05	ND	ND
PA5518 -	0,00	0,09	0,24
PA5519 -	0,35	ND	ND
PA5520 -	-0,63	ND	ND
PA5521 -	-0,52	0,14	0,93
PA5522 -	-0,42	0,14	0,81
PA5523 -	-0,55	0,33	**1,49**
PA5524 -	-0,51	ND	ND
PA5525 -	0,00	ND	ND
PA5526 -	-0,32	ND	ND
PA5527 -	0,07	ND	ND
PA5528 -	-0,37	0,25	**1,07**
PA5529 -	-0,65	ND	ND
PA5530 -	**5,37**	**1,83**	-0,16
PA5531 tonB1	0,28	**1,18**	**3,08**
PA5532 -	-0,66	ND	ND
PA5533 -	-0,43	ND	ND
PA5534 -	**-1,00**	ND	ND
PA5535 -	-0,87	ND	ND
PA5537 -	**-1,25**	ND	ND
PA5541 pyrQ	-0,65	ND	ND
PA5542 -	0,46	ND	ND
PA5543 -	0,24	ND	ND
PA5544 -	0,13	ND	ND
PA5545 -	-0,19	0,86	**2,64**
PA5546 -	-0,32	ND	ND
PA5547 -	-0,84	ND	ND
PA5548 -	-0,06	ND	ND
PA5549 glmS	0,09	-0,07	-0,30
PA5550 glmR	0,58	-0,06	-0,76
PA5551 -	0,06	ND	ND
PA5552 glmU	-0,10	0,10	0,38
PA5553 atpC	-0,14	0,16	0,60
PA5554 atpD	-0,22	0,15	0,66
PA5555 atpG	-0,16	0,17	0,65

ID – Gene [a]	TC [b]	PC [c]	PTV[d]	ID – Gene [a]	TC [b]	PC [c]	PTV[d]	ID – Gene [a]	TC [b]	PC [c]	PTV[d]
PA1768 -	**1,10**	0,44	0,16	PA3817 -	-0,46	-0,19	-0,08	PA5556 atpA	-0,20	0,19	0,74
PA1769 -	0,50	0,38	0,57	PA3818 -	0,18	-0,33	**-1,11**	PA5557 atpH	-0,11	0,18	0,62
PA1770 ppsA	0,47	0,30	0,40	PA3819 -	-0,10	-0,30	-0,76	PA5558 atpF	-0,15	0,23	0,81
PA1771 estX	0,69	0,58	0,96	PA3820 secF	-0,41	-0,11	0,08	PA5559 atpE	-0,15	ND	ND
PA1772 -	0,13	0,07	0,08	PA3821 secD	-0,37	-0,23	-0,27	PA5560 atpB	0,16	0,13	0,20
PA1773 cmaX	0,43	ND	ND	PA3822 -	-0,48	-0,28	-0,30	PA5561 atpI	0,75	ND	ND
PA1774 crfX	**1,15**	ND	ND	PA3823 tgt	-0,18	-0,06	0,01	PA5562 spoOJ	-0,38	-0,19	-0,16
PA1775 cmpX	**1,05**	0,31	-0,18	PA3824 queA	0,13	-0,10	-0,41	PA5563 soj	-0,40	-0,01	0,37
PA1776 sigX	0,86	0,24	-0,19	PA3824.1 -	0,85	ND	ND	PA5564 gidB	-0,17	-0,12	-0,18
PA1777 oprF	0,69	0,51	0,76	PA3825 -	-0,78	ND	ND	PA5565 gidA	-0,14	-0,11	-0,18
PA1778 cobA	0,49	ND	ND	PA3826 -	0,15	ND	ND	PA5566 -	**-1,14**	ND	ND
PA1787 acnB	0,30	0,22	0,32	PA3827 -	-0,17	-0,03	0,07	PA5567 -	-0,11	0,07	0,32
PA1788 -	**1,12**	0,46	0,20	PA3828 -	0,06	-0,08	-0,28	PA5568 -	-0,05	0,21	0,66
PA1789 -	-0,21	-0,01	0,18	PA3829 -	**-1,14**	ND	ND	PA5569 rnpA	0,04	0,00	-0,02
PA1790 -	**1,34**	0,38	-0,25	PA3830 -	-0,54	ND	ND	PA5570 rpmH	-0,10	0,12	0,45

a ID and name of the gene according *Pseudomonas* Genome Database [1]. **b** TC, transcript change **c** PC, protein change **d** PTV, postranscriptional variaton parameter. ND, not detected transcript or protein.

1. Winsor GL, Griffiths EJ, Lo R, Dhillon BK, Shay JA, Brinkman FS: **Enhanced annotations and features for comparing thousands of Pseudomonas Geneomes in the Pseudomonas genome database.** *Nucleic Acids Res* 2016, **44:**D646-653.
